# *Mycobacterium tuberculosis* bacillus induces pyroptosis in human lung fibroblasts

**DOI:** 10.1128/msphere.00110-25

**Published:** 2025-05-19

**Authors:** Takemasa Takii, Hiroyuki Yamada, Chihiro Motozono, Sho Yamasaki, Jordi B. Torrelles, Joanne Turner, Aoi Kimishima, Yukihiro Asami, Naoya Ohara, Shigeaki Hida, Hidetoshi Hayashi, Kikuo Onozaki

**Affiliations:** 1 Department of Mycobacterium Reference and Research, the Research Institute of Tuberculosis, Japan Anti-Tuberculosis Association, Kiyose, Tokyo, Japan; 2 Department of Hygienic Chemistry, Graduate School of Pharmaceutical Sciences, Nagoya City University 12963, Nagoya, Aichi, Japan; 3 Ōmura Satoshi Memorial Institute, Kitasato University 12877, Minato, Tokyo, Japan; 4 Department of Molecular Immunology, Research Institute for Microbial Diseases, The University of Osaka 13013, Suita, Osaka, Japan; 5 Division of Infection and immunity, Joint Research Center for Human Retrovirus, Kumamoto University 13205, Kumamoto, Kumamoto, Japan; 6 Laboratory of Molecular Immunology, Immunology Frontier Research Center, The University of Osaka 13013, Suita, Osaka, Japan; 7 Texas Biomedical Research Institute and International Center for the Advancement of Research & Education (I•CARE) 7075, San Antonio, Texas, USA; 8 Laboratory of Applied Microbial Chemistry, Ōmura Satoshi Memorial Institute, Kitasato University 12877, Minato, Tokyo, Japan; 9 Department of Oral Microbiology, Graduate School of Medicine, Density and Pharmaceutical Sciences, Okayama University 12997, Okayama, Okayama, Japan; 10 Department of Cell Signaling, Graduate School of Pharmaceutical Sciences, Nagoya City University 12963, Nagoya, Aichi, Japan; Washington University in St. Louis School of Medicine, St. Louis, Missouri, USA

**Keywords:** *Mycobacterium tuberculosis*, pyroptosis, caspase, RNA-Seq, cytokine, fibroblasts

## Abstract

We previously reported that live, but not dead, virulent *Mycobacterium tuberculosis* (*Mtb*) H37Rv bacilli induce cell death in human lung fibroblast cell lines, MRC-5, MRC-9, and TIG-1. Here, using two distinct *Mtb* strains from two different lineages (HN878 lineage 2 and H37Rv lineage 4), we confirmed cell death at day 2 after infection with a device that measures cell growth/cytotoxicity in real time (Maestro-Z [AXION]). *Mtb* bacilli uptake by the fibroblast was confirmed with a transmission electron microscope on day 2. Expressions of inflammatory cytokines and interleukin (IL)−1β, IL-6, and IL-8 were observed when exposed to live, but not dead bacteria. The cell death of fibroblasts induced by both *Mtb* strains tested was prevented by caspase-1/4 and NLRP3 inflammasome inhibitors, but not by caspase-3 and caspase-9 inhibitors. Therefore, we classified the fibroblast cell death by *Mtb* infection as pyroptosis. To investigate the biological and pathological relevance of fibroblast cell death by *Mtb* infection, we performed dual RNA-Seq analysis on *Mtb* within fibroblasts and *Mtb*-infected fibroblasts at day 2. In *Mtb* bacilli *tcrR*, *secE2*, *ahpD*, and *mazF8* genes were highly induced during infection. These genes play roles in survival in a hypoxic environment, production of a calcium-binding protein-inducing cytokine, and regulation of transcription in a toxin-antitoxin system. The gene expressions of IL-1β, IL-6, and IL-8, caspase-4, and NLRP3, but not of caspase-3 and caspase-9, were augmented in *Mtb* bacilli-infected fibroblasts. Taken together, our study suggests that *Mtb* bacilli attempt to survive in lung fibroblasts and that pyroptosis of the host fibroblasts activates the immune system against the infection.

**IMPORTANCE:**

The role of “non-classical immune cells,” that is, fibroblasts, epithelial cells, adipocytes, etc., except for the “classical immune cells,” that is, macrophages and lymphoid cells, is not well known in the infection of Mtb bacilli. We have previously found that live, but not dead, Mtb bacilli induce cell death in human lung fibroblasts, except in human macrophages and monocytes. The present study reveals that fibroblasts ingest Mtb bacilli the same as macrophages and that *in vivo* Mtb bacilli within fibroblasts attempt to survive in the host cells, and pyroptosis, including the production of inflammatory cytokines, is induced in the Mtb-infected fibroblasts. Our results suggest that pyroptosis of the host fibroblasts activates the immune system against the infection.

## INTRODUCTION

Tuberculosis (TB), the world’s largest bacterial infectious disease, is caused by the *Mycobacterium tuberculosis* (*Mtb*) bacillus. We previously reported that live, but not dead, *Mtb* exhibits cytotoxicity to normal diploid fibroblasts from fetal lung cell lines MRC-5, MRC-9, and TIG-1 (1, 2). The cytotoxicity of the reported pathogenic *Mycobacteria* is stronger than the cytotoxicity of *Mycobacterium avium*, a pathogen of opportunistic infectious disease, and *Mycobacterium bovis* BCG Pasteur, a TB vaccine strain (1). In our previous studies, both live and dead *Mtb* induced cell death in other human-derived “classical immune cell” lines, that is, U937, THP-1, and HL-60.

Recently, many studies have evaluated the role of cell types other than macrophages in *Mtb* infection. Nevertheless, the role of fibroblasts in the progression or convergence of TB is not well understood. Several researchers reported that fibroblasts produce many inflammatory cytokines, including large quantities of IL-6 (3) and IL-8/CXCL8 (4), when infected with *Mtb* bacilli (5, 6). Furthermore, fibroblasts cooperate with other immune cells by interacting with an integral component of granuloma structures formed in response to *Mtb* bacilli infection, and this interaction activates immune regulatory functions to control the infection (6).

In recent years, pyroptosis has been recognized as the third type of cell death besides apoptosis and necrosis (7). In this study, we investigated whether the cytotoxicity of live *Mtb* is related to pyroptosis by analyzing inhibitors of caspase-1/4 and -3 and NLRP3 inflammasome. Furthermore, we used dual RNA-Seq analysis (8) to analyze both bacteria and host cell factors involved in cytotoxicity and examined gene expression in *in vivo Mtb* bacilli (i.e., within the host cell) and the role of fibroblast cell death in response to *Mtb* infection.

## MATERIALS AND METHODS

### Inhibitors

The inhibitors for caspase-1/4 (VX-765, Adooq BioScience LLC., Irvine, CA, USA), caspase-3 (PAC-1, Selleck Chemicals LLC, Houston, TX, USA), caspase-9 (z-LEHD-fmk, Selleck Chemicals LLC), and NLRP3 inflammasome (MCC950, Adooq BioScience LLC) were dissolved in dimethyl sulfoxide (DMSO) as 10 mg/mL stock and stored at −20°C until use.

### Bacterial cultures and frozen stock, and preparation of dead bacteria

*Mtb* H37Rv (ATTC#27294) and *Mtb* HN878 (BEI Resources, NR-13647) were purchased from the American Type Culture Collection (Manassas, VA, USA) and obtained from NIH-supported BEI Resources, respectively. Bacterial cultures were prepared in Middlebrook 7H9 broth (Difco, Detroit, MI, USA) supplemented with 10% albumin dextrose catalase (5% bovine serum albumin [fraction V], 2% dextrose, and 0.004% bovine liver catalase; Difco) and 0.05% Tween 80 and were cultured at 37°C under static conditions. Bacteria were grown to an optical density of 0.6–0.8 at 530 nm. Then, the cultures were aliquoted and stored at −70°C until needed. The number of colony-forming units in the aliquots was determined by colony assays on Middlebrook 7H11 agar (Difco) supplemented with 10% oleic albumin dextrose catalase (0.05% oleic acid, 5% bovine serum albumin [fraction V], 2% dextrose, and 0.004% bovine liver catalase; Difco). The dead bacteria were prepared by boiling, 95°C for 30 min, or by treatment with antibiotics, 100 µg/mL isoniazid and 100 µg/mL streptomycin for 1 day.

### Cell culture

The tissue culture medium for human embryonic lung fibroblast cell lines, MRC-5 (normal diploid fibroblasts from male), MRC-9 (normal diploid fibroblasts from female), and TIG-1 (normal diploid fibroblasts from female) (JCRB, Tsukuba, Ibaraki, Japan) were maintained, Dulbecco’s modified Eagle medium (D-MEM; low glucose) with L-glutamine and phenol red (Wako Pure Chemical Industries, Ltd., Osaka, Japan), 100 µg/mL streptomycin (Meiji Seika Pharma Co., Ltd., Tokyo, Japan) and 100 units/mL penicillin G (Meiji Seika Pharma) and 5% heat-inactivated fetal bovine serum (FBS; Hyclone, GE Health Life Science, South Logan, UT, USA). The culture was maintained at 37°C in 5% CO_2_ in a 100 mm in diameter Falcon standard tissue culture dish (No. 353003, Thermo Fisher Scientific, Waltham, MA, USA).

### Transmission electron microscopy

Fibroblasts (2 × 10^6^ cells/dish) were cultured for 24 hours, and then incubated with 2 × 10^7^ bacilli for the indicated period. After infection, the cells were washed with PBS (-) twice and scraped off with a cell scraper. Then, they were suspended in 1 mL of 2.5% glutaraldehyde (TAAB Laboratories Equipment Ltd, UK) in 100 mM phosphate buffer (PB) with pH 7.4 and fixed overnight at 4°C. After centrifugation and rinsing with PB, the cell pellets were post-fixed with 1% osmium tetroxide (Heraeus, South Africa) for 1 hour at 4 °C. After centrifugation, the osmium solution was discarded, and the cells were subjected to dehydration with a grading ethanol series with repeated centrifugation at room temperature (RT). Next, the cells were suspended in a solution of ethanol and Spurr’s resin (1:1) overnight at RT, and in complete Spurr’s resin for 12 hours twice at RT and then embedded with Spurr’s resin at 70 °C for 2 days. Ultrathin sections of 80 nm thickness were cut with ARTOS 3D (Leica Mikrosysteme GmbH, Vienna, Austria), mounted on a SiN window chip (Cat.# 783131836, JEOL, Tokyo, Japan) (9), and stained with uranyl acetate and lead citrate. Transmission electron microscopic examinations were performed with a JEM-2100 Plus microscope operated at 120 kV acceleration voltage and equipped with a complementary metal-oxide semiconductor (CMOS) camera with 2,048 × 2,048 pixels.

### Acid-fast staining

Fibroblasts (2 × 10^5^ cells/well) were cultured on an 8-well chamber microscope glass slide (SCS-No. 8, Matsunami Glass Ind., Ltd., Osaka, Japan) for 24 hours, and then incubated with 2 × 10^6^ bacilli for the indicated period. Then, the cells were washed with 1 mL of PBS(−) twice and then fixed with 2.5% glutaraldehyde (TAAB Laboratories Equipment Ltd, UK) in PB at 4°C overnight. After rinsing with PB three times, the cells were stained with Ziehl-Neelsen.

### Confocal laser microscopy

Fibroblasts (1 × 10^3^ cell/mL) were cultured in a slide chamber (Matsunami Glass Ind. Ltd., Osaka, Japan) for 4 hours, and then enhanced green fluorescent protein expressing live *Mycobacterium bovis* BCG bacilli (1 × 10^4^ cfu/mL), kindly provided by Dr. T. Mukai (National Institute of Infectious Disease Japan, Higashi Murayama, Japan), was added. The chamber was washed with PBS(−) twice, and fresh culture medium was added. Thirty minutes before examination with confocal laser microscopy (Zeiss LSM-410, Oberkochen, Germany), lysosomes and the nuclear membrane of the fibroblast were stained with 50 nM of LysoTracker Red DND-99 (Thermo Fisher Scientific, Waltham, MA, USA), and 5 µg/mL of Hoechst 33342 (Dojindo, Kumamoto, Japan), respectively.

### Real-time measurement of cytotoxicity with Maestro-Z (AXION)

Before seeding fibroblasts on the 96-well plate (CytoView-Z, AXION, Atlanta, GA, USA) for Maestro-Z (AXION), the plate was coated with 100 µL of fibronectin (REF11051407001, Roche, Basel, Switzerland; 1 µg/mL) for 1 hour at RT. Then, the coating solution was removed, and 100 mL of the tissue culture medium was added, followed by adding 8 mL of sterile water on the plate reservoirs to increase the humidity. The plate was inserted into the chamber of the Maestro-Z to measure the media-only (MO) baseline. The cells were diluted with D-MEM 5% FBS containing 100 U/mL penicillin at 1.0 × 10^5^ cells/mL. Once the MO baseline was obtained, the culture medium was removed from the plate, and then 100 µL of the cell suspension was seeded on the plate. For tissue culture, the fibroblast-seeded plate was incubated within a chamber of a Maestro-Z or a CO_2_ incubator.

### Dual-RNA-Seq analysis

RNA was extracted according to the procedure described by Pisu et al*.* (8), using TRIzol reagents (Thermo Fisher Scientific, CA, USA) and mixing total RNA (eukaryotic + bacterial) from *Mtb-*infected MRC-5 fibroblasts at an optimal pathogen to host RNA ratio. As a reference, total RNA was extracted from cultured *Mtb* bacilli and uninfected MRC-5 fibroblasts. RNA-Seq and bioinformatics analysis were conducted by Macrogen (Kyoto, Japan).

### Real-time quantitative RT-PCR (qRT-PCR) and RNase protection assay

Total RNA was extracted from the fibroblasts using TRIzol reagents (Thermo Fisher Scientific, CA, USA) according to the manufacturer’s instructions. The quantity and quality of the extracted RNA were determined using the NanoVue plus spectrophotometer (GE Healthcare, Chicago, IL, USA). The isolated RNA was digested with DNase1 (Takara Bio Inc., Shiga, Japan) at 37°C for 30 min. For cDNA synthesis, 100 ng of RNA was used with PrimeScript RT master mix (Takara Bio Inc.). Real-time PCRs were performed in an Applied Biosystems StepOnePlus real-time PCR system with real-time PCR software version 2.3 (Applied Biosystems, Thermo Fisher, Foster City, CA, USA). Each reaction was performed in a total volume of 10 µL on a 48-well optical reaction plate (Applied Biosystems) containing 5 µL of TB Green Fast qPCR Mix (Takara Bio Inc.), 100 to 1,000 ng of cDNA (1/10 dilution), and two gene-specific primers at a final concentration of 0.5  µM each. The real-time cycling conditions were as follows: (i) 95°C for 30 s and (ii) 40 cycles at 95°C for 5 s and 60°C for 10 s. Melting curve analysis verified that each reaction contained a single PCR product. Reported gene expression levels were normalized to transcripts of GAPDH. We used primer sets of human GAPDH (5′-TGCCAACGTGTCGGTTGT-3′, 5′-TGTCATCATATTTGGCAGGTTT-3′), human IL-1β (5′-GGGCATCAAGGGCTACAA-3′, 5′-CTGTCCAGGCGGTAGAAGAT-3′), human IL-6 (5′-AGAAATCCCTCCTCGCCAAT-3′, 5′- AAATAGCGAACGGCCCTCA-3′), and human IL-8 (5′- TCTGCAGCTCTGTGTGAAGG-3′, 5′- ACTTCTCCACAACCCTCTGC-3′).

The expression of mRNA of inflammatory cytokines was measured by RNase protection assay kits (BD Biosciences Pharmingen, San Diego, CA, USA).

### Reporter gene assay

MRC-5 fibroblasts (3 × 10^5^ cells/well) were cultured in a six-well plate and transfected with reporter plasmids by the calcium phosphate method. The plasmids contained the 5′ upstream region of IL-6 or IL-8 genes, including the NF-κB-binding site conjugated to the luciferase gene provided kindly by Prof. N. Mukaida (Kanazawa Univ., Japan). As a reference for the plasmid transfection, cells were also transfected with another plasmid-encoding β-galactosidase gene. After transfection with these plasmids, the fibroblasts were cultured with either live or dead *Mtb* bacilli (10 bacilli per fibroblast) up to day 3. The results were assessed as the ratio of luciferase to β-galactosidase activity.

### Measurement of cytokine levels in the culture supernatant

The amounts of human interleukin-1β (IL-1β), IL-6, and IL-8 in the supernatants of fibroblasts were quantified by enzyme-linked immunosorbent assay (ELISA) using a BD OptEIA ELISA set (BD Bioscience, San Jose, CA, USA). IL-18 in the supernatant was quantified by human IL-18 ELISA kit (Medical & Biological Laboratories Co., LTD, Tokyo, Japan).

### Statistical analysis

**Fig 1:**
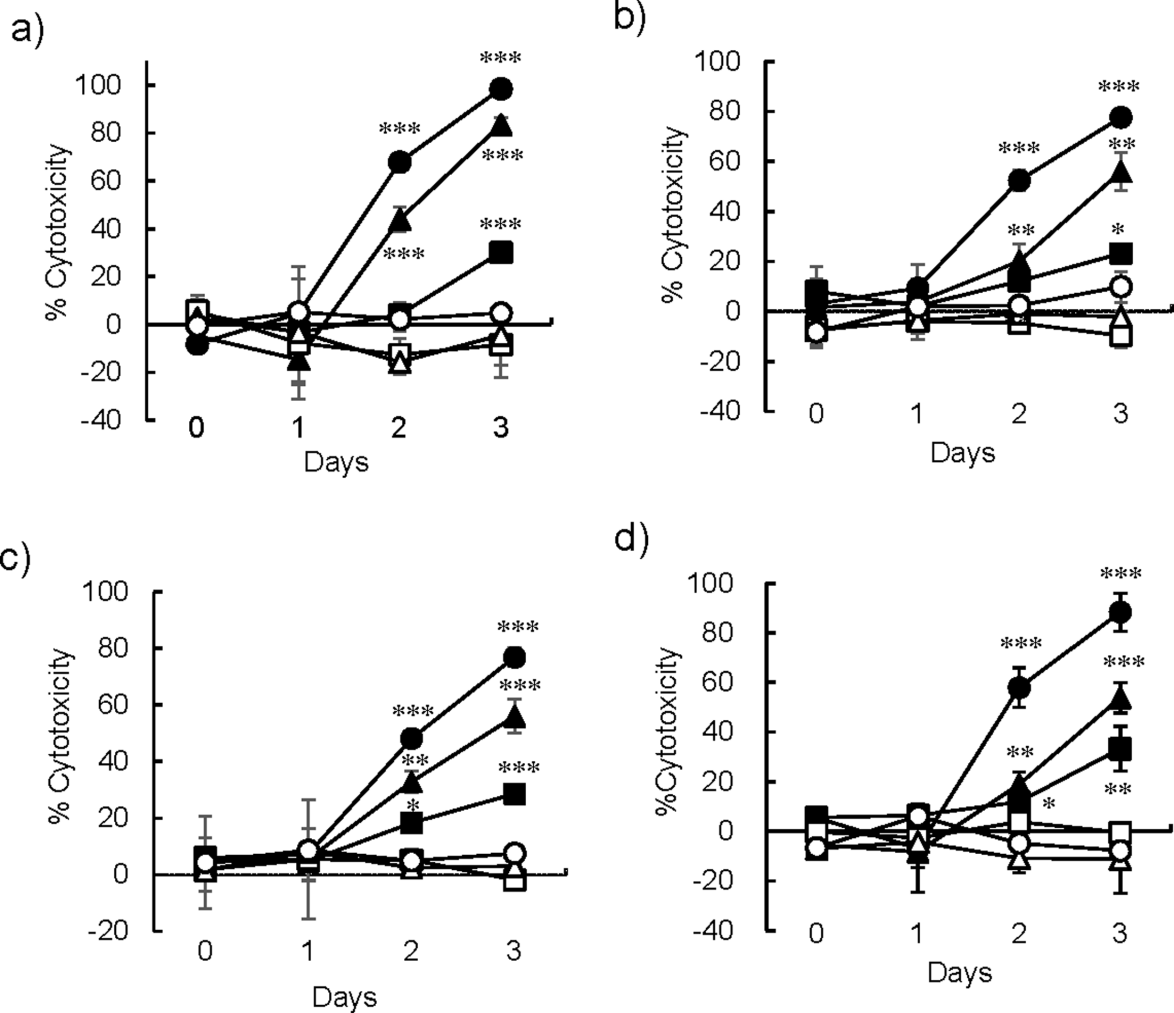
Time course of cytotoxicity induced by live, but not *Mycobacterium tuberculosis* (*Mtb*) bacilli in fibroblasts. Cytotoxicity was measured in real time with Maestro-Z (AXION), which measures the electrical resistance of the fibroblasts adhering to the bottom of an electrode embedded well using CytoView-Z 96-well plate (AXION). Fibroblasts (1 × 10^4^ cells/well) were incubated with live or heat-killed *Mycobacterium tuberculosis* (*Mtb*) H37Rv for 96 hours. Initially, 0–100 bacilli were added per fibroblast, (**a**) MRC-5, (**b**) MRC-9, and (c) TIG-1, respectively. (**d**) MRC-5 fibroblasts were incubated with live or heat-killed *Mtb* HN878 for 96 hours in the same manner. Solid and open shapes indicate the results of live and dead bacilli, respectively. Circle, triangle, and square indicate the number of bacteria added to the fibroblast: 100, 10, 1, respectively. Each experiment was conducted in triplicate. The statistical significance of the difference between the results of each experiment was analyzed by one-way analysis of variance, *P < 0.05, **P < 0.01, ***P < 0.001.

The statistical significance of the data sets, shown in Fig. 1, 3, 4, and 5; Fig. S3 and S5, was assessed by one-way analysis of variance with SigmaPlot (Systat Software, Inc., San Jose, CA, USA), and differences were considered significant at *P* < 0.05. Statistical analyses shown in Fig. 7 and 8 were performed by Macrogen (Kyoto, Japan) with Fold Change nbiomWorldTest using DESeq2 per comparison pair.

## RESULTS

### Early ingestion of *Mtb* H37Rv and *Mtb* HN878 by human fibroblast cell line induces cytotoxicity

We previously reported that the cytotoxic effect of live *Mtb* bacilli on MRC-5, MRC-9, and TIG-1, and lung epithelial cell A549 was observed 2 days after infection (1). In a previous study, we investigated live *Mtb*-induced cytotoxicity by measuring the clearance of an *Mtb*-infected cell line from a tissue culture plate; to do so, we assayed crystal violet staining and release of lactate dehydrogenase, a cytoplasmic enzyme from the infected cells (2). In the present study, we confirmed the cytotoxicity induced by live *Mtb* bacilli with the Maestro-Z (AXION), a device that assesses cytotoxicity in real time by measuring the electrical resistance of the fibroblasts adhering to the bottom of the well in a CytoView-Z 96-well plate (AXION), which is embedded in an electrode. Live, but not dead, *Mtb*, H37Rv lineage 4, induced cytotoxicity in MRC-5, MRC-9, and TIG-1 fibroblasts in a time- and bacterial number-dependent manner (Fig. 1a through c). The cytotoxicity by other strains of *Mtb* bacilli, HN878 lineage 2, was also induced in MRC-5 fibroblasts in the same manner (Fig. 1d).

**Fig 2:**
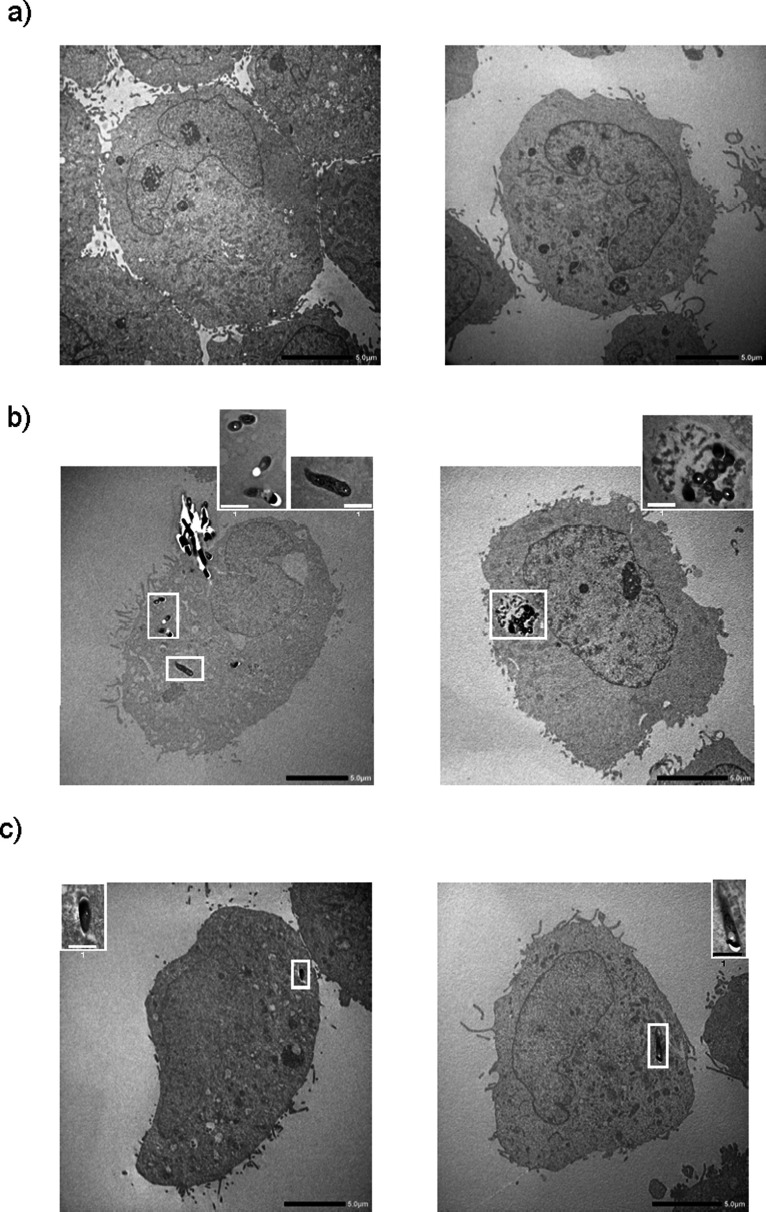
Uptake of *Mycobacterium tuberculosis* H37Rv bacilli by MRC-5 fibroblasts. MRC-5 fibroblasts (2 × 10^6^ cells/dish) were cultured in a 100 mm diameter tissue culture dish with live *Mycobacterium tuberculosis* (*Mtb*) H37Rv (2 × 10^7^ colony-forming units per dish) for the indicated period. After the incubation period, the cells were fixed with glutaraldehyde, and the dehydration steps were performed. After samples were composed with Spurr’s resin, ultrathin sections of each sample were cut to 80 nm thickness with ARTOS 3D (Leica Mikrosysteme GmbH, Vienna, Austria) and stained with uranyl acetate and lead citrate. Transmission electron microscopic examinations were performed with JEM-2100 Plus equipped with a complementary metal-oxide semiconductor CMOS camera with 2,048 x 2,048 pixels. Panels a, b, and c indicate the incubation time of *Mtb* H37Rv bacilli and MRC-5 fibroblasts on days 1, 2, and 3, respectively. The black bar shows the scale (5.0 µm). The bacilli in the fibroblast are surrounded by a white frame. An enlarged image of the bacilli cut horizontally is shown in the top right corner of panel b. The white bar shows the scale (1.0 µm).

*Mtb* H37Rv bacilli were located inside fibroblasts at days 2 and 3 (Fig. 2b and c). We confirmed the result by acid-fast staining in the same preparation sample (data not shown). We further confirmed the phagocytosis of *M. bovis* BCG bacilli by fibroblasts with a confocal laser microscope (Fig. S1) and also observed that *M. bovis* BCG killed by heat or the anti-mycobacterial drugs, isoniazid and streptomycin, were also inside fibroblasts (Fig. S2). These data indicate that fibroblasts can ingest both live and dead bacilli, but that only live *Mtb* bacilli induce cytotoxicity in fibroblasts.

### Effect of caspase and NLRP3 inflammasome inhibitors on the mycobacterial cytotoxicity against fibroblasts

**Fig 3:**
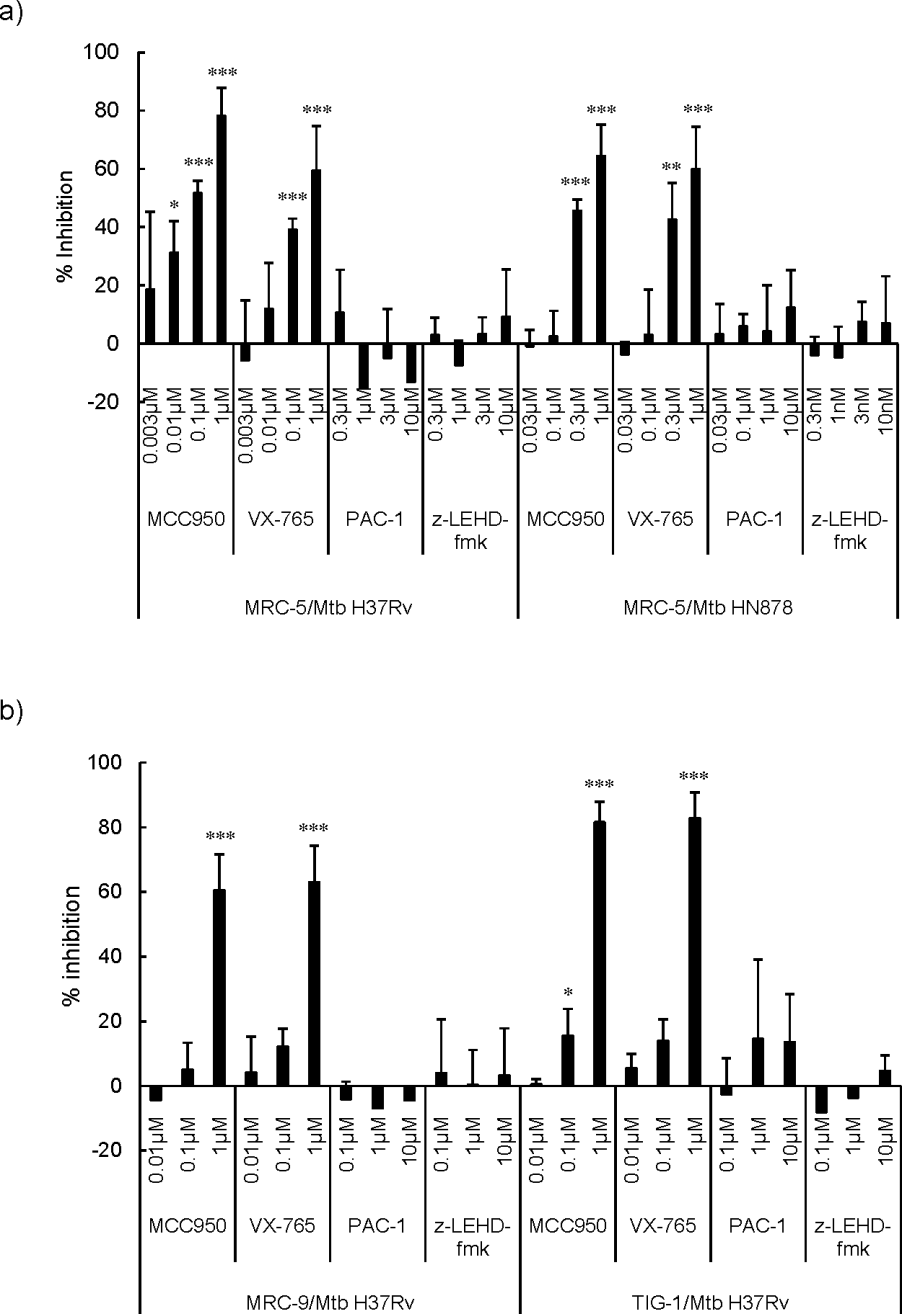
Effects of inhibitors for caspase-1/4, caspase-3, caspase-9, and NLRP3 inflammasome on live *Mycobacterium tuberculosis* (*Mtb*) bacilli-induced cytotoxicity to fibroblasts. (**a**) MRC-5 (1 × 10^4^ cells/well) fibroblasts were seeded on a fibronectin pre-coated CytoView-Z 96-well plate 4 hours before the addition of the inhibitors of caspase-1/4 (VX-765), caspase-3 (PAC-1), caspase-9 (z-LEHD-fmk), and NLRP3 inflammasome (MCC950), and 30 minutes later, the cells were infected with (a) live *Mycobacterium tuberculosis* (*Mtb*) H37Rv and *Mtb* HN878 bacilli (1 × 10^6^ CFU/well). Results are 2 days after incubation with the bacilli. The percentage of inhibition is shown in the panel, and row data are shown in Fig. S3. The error bars for negative numbers were excluded in panel **b**. MRC-9 and TIG-1 were infected with *Mtb* H37Rv, and the effects of inhibitors were investigated. Each experiment was performed in triplicates. The statistical significance of the difference between each experiment and the result of incubation with *Mtb* H37Rv or *Mtb* HN878 alone were analyzed by one-way analysis of variance, *P < 0.05, **P < 0.01, ***P < 0.001.

Next, we investigated the type of cell death using selective protease inhibitors, that is, the caspase-1/4 inhibitor VX-765 (Ki = 0.8 nM), the caspase-3 inhibitor PAC-1 (EC_50_ = 0.22 µM), the caspase-9 inhibitor z-LEHD-fmk (IC_50_ = 0.12 µM), and the NLRP3 inflammasome inhibitor MCC950 (IC_50_ = 7.5 nM). We observed that the caspase 1/4 and NLRP3 inflammasome inhibitors, but not caspase-3 and the caspase-9 inhibitors, were able to inhibit the cytotoxicity induced by *Mtb* H37Rv and *Mtb* HN878 in MRC-5 (Fig. 3a; Fig. S3). VX-765 and MCC950, but not PAC-1 and z-LEHD-fmk, also inhibited the cytotoxicity in MRC-9 and TIG-1 fibroblasts in the same manner (Fig. 3b). These results suggest that live *Mtb* induced pyroptosis (7), but not apoptosis in fibroblasts.

### Induction of the inflammatory cytokines, IL-1β, IL-6, and IL-8, but not IL-18, by fibroblasts after *Mtb* infection

**Fig 4:**
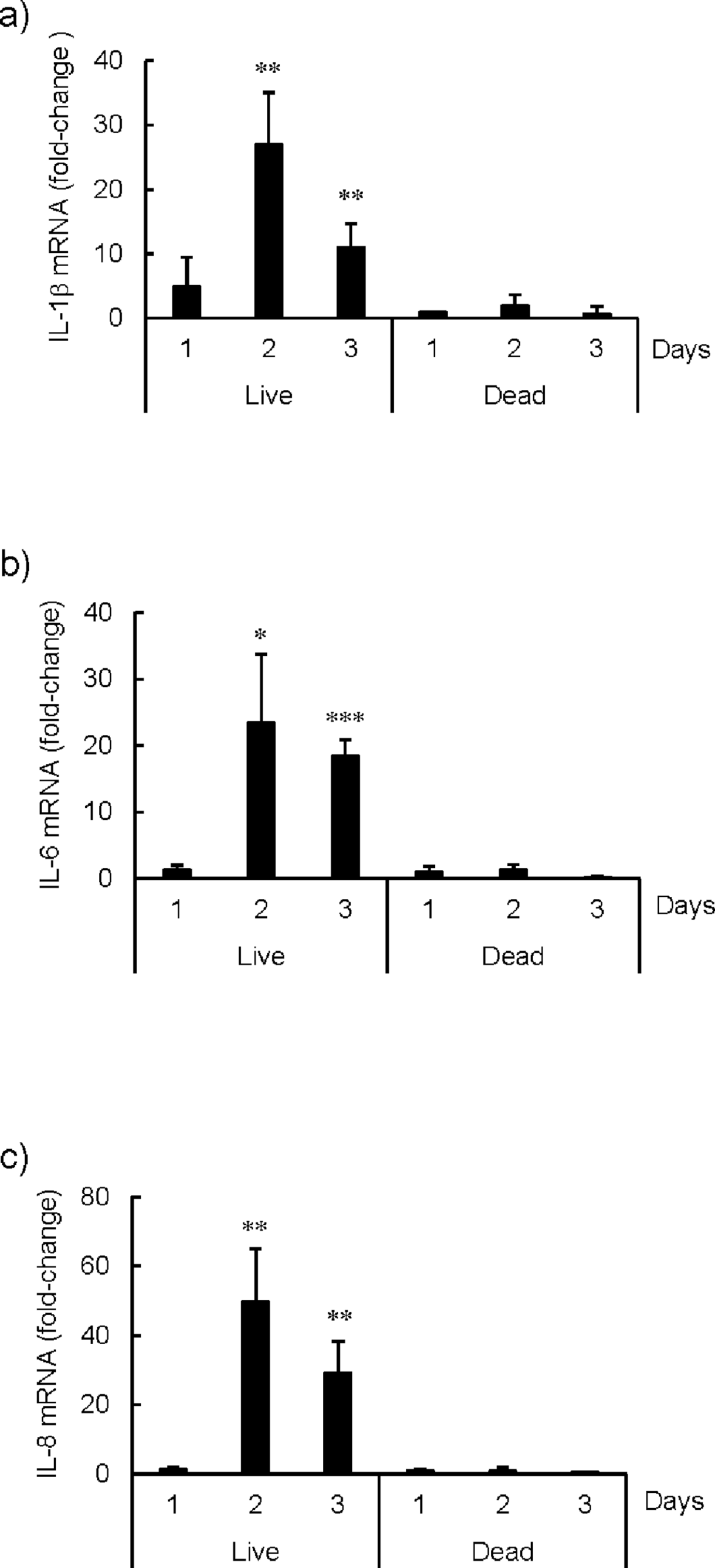
mRNA expression of IL-1β, IL-6, and IL-8 in MRC-5 fibroblasts induced by live, but not dead *Mycobacterium tuberculosis* H37Rv bacilli. MRC-5 fibroblasts (2 × 10^6^ cell/dish) were cultured in a 100 mm diameter tissue culture dish with either live or heat-killed *Mycobacterium tuberculosis* H37Rv bacilli (2 × 10^7^ bacilli/dish) for the indicated period. mRNA from MRC-5 fibroblasts was extracted with TRIzol reagents (Thermo Fisher Scientific). The mRNA expression of (a) IL-1β, (b) IL-6, and (c) IL-8 was measured by qRT-PCR using the specific primer sets. The statistical significance of the difference between each experiment and the result of incubation with dead bacilli at day 1 were analyzed by one-way analysis of variance, *P < 0.05, **P < 0.01, ***P < 0.001.

Inflammatory cytokines are produced by cells during pyroptosis. Fibroblasts were incubated with live or dead *Mtb* H37Rv for 3 days, and mRNA was extracted every day and evaluated by qRT-PCR. The mRNA expression of IL-1β (Fig. 4a), IL-6 (Fig. 4b), and IL-8 (Fig. 4c) was induced by live *Mtb* bacilli at day 2, and the inductions were sustained to day 3. Similar results were observed using RNase protection assay (Fig. S4).

**Fig 5:**
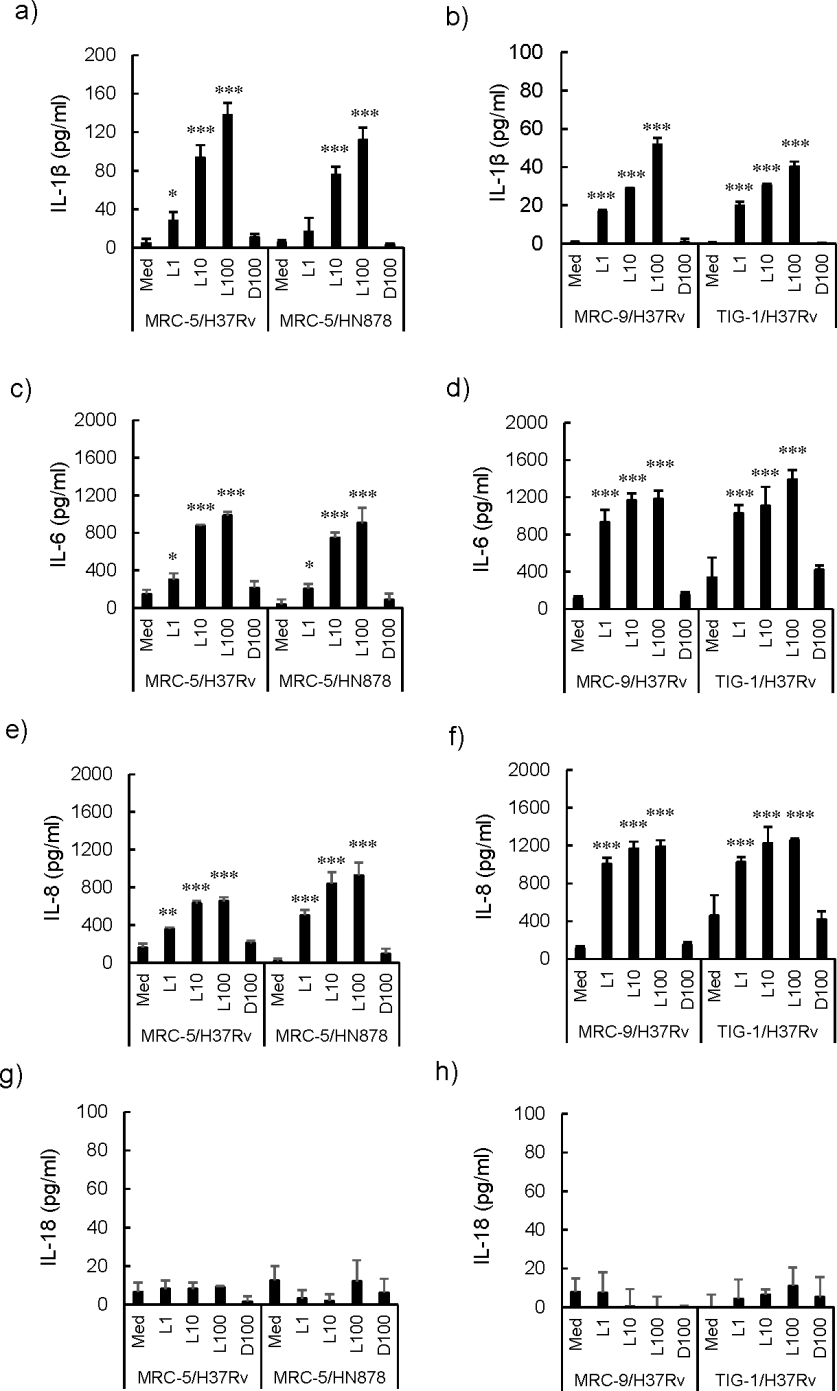
The production of IL-1β, IL-6, IL-8, and IL-18 from fibroblasts was induced by live, but not dead *Mycobacterium tuberculosis* (*Mtb*) bacilli. Fibroblasts (1 × 10^4^ cells/well) indicated in each panel were cultured in a 96-well plate with either live or heat-killed *Mycobacterium tuberculosis* (*Mtb*) H37Rv and/or *Mtb* HN878 bacilli for 3 days. The amounts of human (a, b) IL-1β, (c, d) IL-6, (e, f) IL-8, and (g, h) IL-18 in the supernatants of fibroblasts were quantified by ELISA. The Med column shows the results without the bacteria. L and D indicated live and dead bacilli, and the numbers, 1, 10, and 100, indicate bacterial number per the fibroblast. Each experiment was conducted in triplicate. The statistical significance of the difference between each experiment and Med result was analyzed by one-way analysis of variance, *P < 0.05, **P < 0.01, ***P < 0.001.

Because we believed that IL-1β, IL-6, and IL-8 were the biologically active cytokines produced by fibroblasts infected with *Mtb*, we measured the concentration of IL-1β, IL-6, and IL-8 proteins in supernatants of MRC-5, MRC-9, and TIG-1 fibroblasts by ELISA at day 3 (Fig. 5). We further investigated the gene expression of IL-6 and IL-8 by luciferase reporter assay and found that it was induced by live, but not dead, *Mtb* bacilli at day 2 and that the induction was sustained at day 3 (Fig. S5b and c, e, and f). The binding site of transcription factor NF-κB is located at 5′ upstream of inflammatory cytokine genes, such as IL-1β, IL-6, and IL-8, and NF-κB is known to activate the transcription of these genes during inflammation. The results of the reporter assay showed activation of NF-κB by live, but not dead *Mtb* bacilli infection at day 2 (Fig. S5f). These results suggest that *in vivo Mtb* bacilli-induced pyroptosis in fibroblasts was accompanied by the production of inflammatory cytokines.

IL-18, like IL-1β, is activated by caspase-1, but IL-18 was not detected in fibroblasts (Fig. 5; Table S2). These results suggest that IL-18 is not involved in cell death induced by live *Mtb*.

### Dual-RNA-Seq analysis of live *Mycobacterium tuberculosis* bacilli induced cytotoxicity in fibroblasts

To perform a comprehensive analysis of mRNA expression in fibroblasts infected with live *Mycobacterium tuberculosis* (*Mtb*) H37Rv bacilli, on day 2 we extracted mRNAs from both *in vitro Mtb* H37Rv bacilli in the fibroblasts and the fibroblasts infected with the *Mtb* bacilli, and then performed dual RNA-Seq analysis to evaluate mRNA expression in both *in vivo Mtb* bacilli and *Mtb*-infected host cells (8).

**Fig 6:**
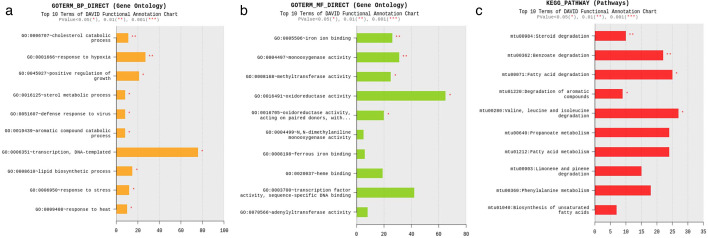
Dual RNA-Seq analysis of *in vivo Mycobacterium tuberculosis* (*Mtb*) bacilli. MRC-5 fibroblasts (2 × 10^6^ cells/dish) were co-cultured with *Mycobacterium tuberculosis* (*Mtb*) H37Rv bacilli (2 × 10^7^ CFU/dish) for 2 days. According to the reference method (8) for dual RNA-Seq analysis of the *Mtb* bacilli-infected macrophages, the host cells were solubilized with TRIsol solution. The lysate containing the particles of the *in vivo Mtb* bacilli was centrifuged, and the supernatant was used for RNA extraction and RNA-Seq analysis of the host cell. Fresh TRIsol solution and zirconia beads were added to the pellet, and bacterial bodies were broken up with a bead beater. After extracting the RNA fractions, the quality of nucleic acid was confirmed, and then RNA-Seq analysis was performed by Macrogen (Kyoto, Japan). (**a**) Gene ontology (GO) analysis of biological processes (BP), (**b**) GO analysis of molecular functions (MF), and (c) KEGG pathway analysis. Each experiment was performed in triplicates. The statistical significance of the difference between the result of each experiment and the result of *in vitro* cultured *Mtb* bacilli was analyzed by one-way analysis of variance, *P < 0.05, **P < 0.01.

First, we analyzed mRNA expression in *in vivo Mtb* bacilli. The gene ontology (GO) analysis of the biological processes of the *in vivo* bacilli showed that mRNAs from DNA-templated transcription were extensively upregulated compared with the *in vitro* cultured *Mtb* bacilli (Fig. 6a). The response to hypoxia and the positive regulation of growth were also augmented in the *in vivo* bacilli. The results of GO terms related to molecular function of the *in vivo Mtb* bacilli showed that oxidoreductase activity was highly upregulated in the *in vivo* bacilli compared with the *in vitro* bacilli (Fig. 6b). The expression of genes involved in iron binding and monooxygenase and methyltransferase activity was also augmented in the bacilli. These data suggest that *Mtb* bacilli incorporated into the fibroblasts survive in the hypoxic environment of the host cell.

Then, we further performed KEGG pathway analysis with the *in vivo Mtb* and found that the genes for benzoate, fatty acid, and steroid degradation were upregulated (Fig. 6c). *Mycobacterium*, *Rhodococcus,* and *Pseudomonas* are classified into steroid-degrading bacteria; thus, the augmentation of the gene expressions of steroid degradation was confirmed. The elevated gene expressions of benzoate and fatty acid degradation may correspond to the anaerobic biodegradation and acquisition of the carbon source in the hypoalimentation state inside the host cell.

**Fig 7:**
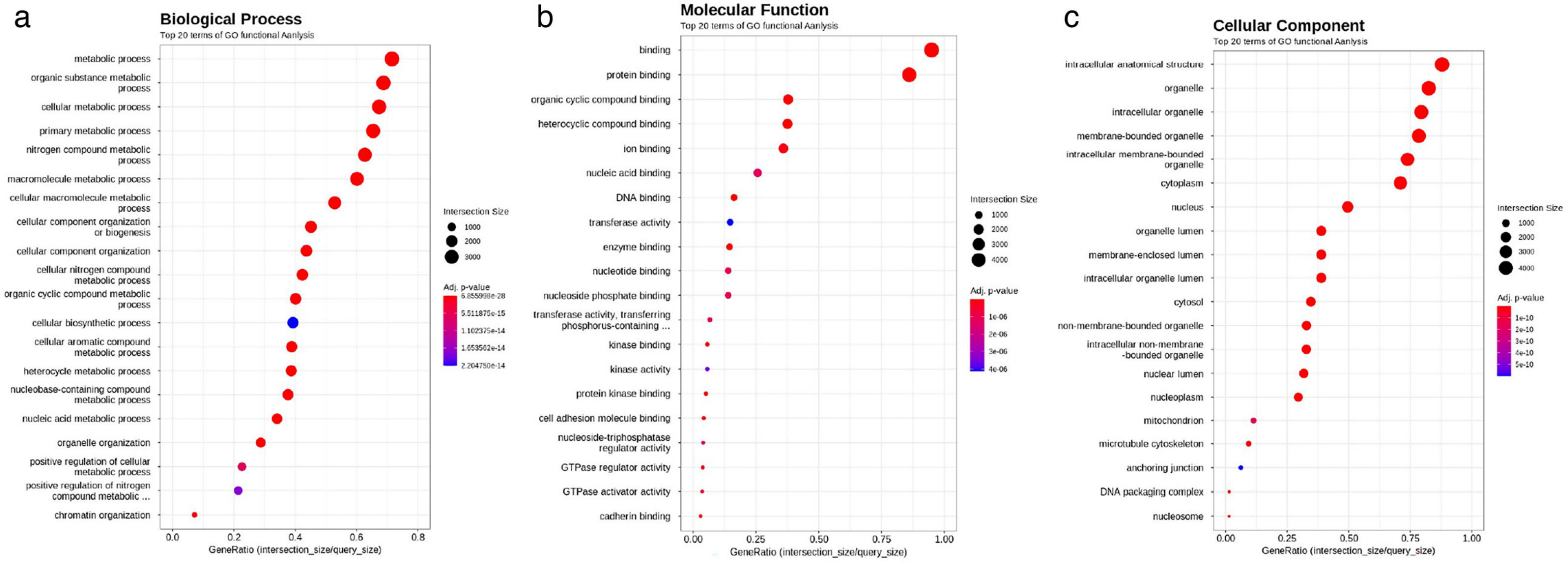
Dual RNA-Seq analysis of *Mycobacterium tuberculosis* (*Mtb*) bacilli-infected fibroblasts. The preparation of the RNA fraction is described in the legend to Fig. 6. (**a**) Gene ontology (GO) analysis of biological processes, (**b**) GO analysis of molecular functions, (c) GO analysis of cellular components. Each experiment was performed in triplicates.

Next, we performed a GO functional analysis of the host side, that is, we compared *Mtb*-infected and -uninfected fibroblasts. The analysis of biological processes showed upregulation of the genes for metabolic processes, including organic substances, cellular, primary, nitrogen compounds, and macromolecules (Fig. 7). Interestingly, the genes of binding and protein binding were strongly induced in the GO analysis of molecular function (Fig. 7b). Furthermore, the GO cellular component analysis showed that genes involved in intracellular anatomical structure, organelles, intercellular, membrane-bound organelles, and cytoplasm were highly induced in the *Mtb*-infected fibroblasts (Fig. 7c). These results were assumed to reflect changes in intracellular anatomical and subcellular organelle structure in the fibroblasts after incorporation of *Mtb* bacilli.

The top 50 differentially expressed genes in *in vivo* vs *in vitro Mtb* H37Rv are shown in Table 1. Transcriptional regulator TrcR (part of a two-component system), the protein translocase subunit SecE, alkyl hydroperoxide reductase AphD, NADH-quinone oxidoreductase subunit K, and toxin MazF8 genes were upregulated in the *in vivo Mtb* bacilli. These substances are important for survival in the fibroblasts, induction of cytotoxicity, and production of inflammatory cytokines by the host cell. The top 25 upregulated genes in *Mtb* H37Rv-infected fibroblasts are shown in Table 2. The dual RNA-Seq analysis confirmed that the IL-1β and IL-8/CXCL8 gene was also induced, with a 33.9- and 98.4-fold change (fc.) in the *Mtb*-infected vs -uninfected fibroblasts, respectively, at day 2 (Table 2). The IL-6 gene was also induced in the *Mtb*-infected fibroblasts (fc. 17.0; Table S2).

**TABLE 1:** Top 50 DEGs of *in vivo* vs *in vitro Mtb* H37Rv by RNA Seq analysis^*a*^^,^^*b*^

Gene symbol	Description	Gene biotype	Protein ID	Locus tag	*In vivo*/*in vitro Mtb* H37Rv.fc
alaT	tRNA-Ala	tRNA	–	Rvnt02	24.8
trcR	Two-component transcriptional regulator TrcR	protein_coding	NP_215549.1	Rv1033c	23.0
leuU	tRNA-Leu	tRNA	–	Rvnt22	21.8
secE2	Protein translocase subunit SecE	protein_coding	YP_177722.1	Rv0379	19.0
–	Diterpene synthase	protein_coding	NP_217895.1	Rv3378c	17.2
ahpD	Alkyl hydroperoxide reductase AphD	protein_coding	NP_216945.1	Rv2429	17.1
nuoK	NADH-quinone oxidoreductase subunit K	protein_coding	NP_217671.1	Rv3155	15.6
–	Hypothetical protein	protein_coding	NP_216787.1	Rv2271	15.5
–	Hypothetical protein	protein_coding	NP_216893.1	Rv2377c	14.5
–	Hypothetical protein	protein_coding	NP_215867.1	Rv1351	14.1
–	Hypothetical protein	protein_coding	NP_216326.1	Rv1810	13.9
mazF8	Toxin MazF8	protein_coding	NP_216790.1	Rv2274c	13.6
–	D-amino acid aminohydrolase	protein_coding	NP_217429.1	Rv2913c	−135.3
–	Transcriptional regulator	protein_coding	NP_215307.1	Rv0792c	−92.3
–	Hypothetical protein	protein_coding	NP_215306.1	Rv0791c	−82.2
–	Carotenoid cleavage oxygenase	protein_coding	NP_215168.1	Rv0654	−76.4
alkB	Transmembrane alkane 1-monooxygenase AlkB	protein_coding	NP_217769.1	Rv3252c	−76.3
–	Hypothetical protein	protein_coding	NP_215305.1	Rv0790c	−73.9
–	TetR family HTH-type transcriptional regulator	protein_coding	NP_217428.1	Rv2912c	−47.6
–	Hypothetical protein	protein_coding	NP_215352.1	Rv1813c	−45.8
–	Hypothetical protein	protein_coding	NP_215352.1	Rv0837c	−45.1
narX	Nitrate reductase-like protein NarX	protein_coding	NP_216548.1	Rv1736c	−37.8
acg	NAD(P)H nitroreductase	protein_coding	NP_216548.1	Rv2032	−35.8
–	Hypothetical protein	protein_coding	NP_217786.1	Rv3269	−30.8
hsp	Heat shock protein	protein_coding	NP_214765.1	Rv0251c	−30.5
aldA	Aldehyde dehydrogenase AldA	protein_coding	NP_215282.1	Rv0768	−29.4
ctpC	Manganese/zinc-exporting P-type ATPase	protein_coding	NP_217787.1	Rv3270	−27.6
–	NAD(P)H nitroreductase	protein_coding	NP_217647.3	Rv3131	−26.2
–	Hypothetical protein	protein_coding	NP_216982.1	Rv2466c	−25.9
cysD	Sulfate adenylyltransferase subunit 2	protein_coding	NP_215801.1	Rv1285	−25.7
hsaG	Acetaldehyde dehydrogenase	protein_coding	NP_218052.1	Rv3535c	−24.7
mmsA	Methylmalonate-semialdehyde dehydrogenase	protein_coding	NP_215267.1	Rv0753c	−24.7
–	Hypothetical protein	protein_coding	NP_214593.1	Rv0079	−22.4
–	Oxidoreductase	protein_coding	NP_215279.1	Rv0765c	−21.7
cyp135A1	Cytochrome P450 Cyp135A1	protein_coding	NP_214841.1	Rv0327c	−21.1
mcr11	Putative small regulatory RNA	ncRNA	–	RVnc0013	−20.3
–	Hypothetical protein	protein_coding	NP_218177.1	Rv3660c	−19.3
–	Hypothetical protein	protein_coding	NP_216546.1	Rv2030c	−18.9
tgs1	Diacylglycerol O-acyltransferase	protein_coding	NP_217646.1	Rv3130c	−18.5
PPE17	PPE family protein PPE17	protein_coding	YP_177791.1	Rv1168c	−18.4
clpB	Chaperone protein ClpB	protein_coding	NP_214898.1	Rv0384c	−18.0
–	HTH-type transcriptional regulator	protein_coding	NP_215281.1	Rv0767c	−17.7
–	Phage integrase	protein_coding	NP_216102.1	Rv1586c	−17.5
–	Dioxygenase	protein_coding	NP_217923.1	Rv3406	−17.4
–	Hypothetical protein	protein_coding	NP_218048.1	Rv3531c	−17.1
–	Oxidoreductase	protein_coding	NP_215283.1	Rv0769	−16.5
–	Hypothetical protein	protein_coding	NP_217980.1	Rv3463	−16.1
higB	Toxin HigB	protein_coding	NP_216471.2	Rv1955	−15.7
cydD	Cytochrome biosynthesis ABC transporter ATP-binding protein/permease CydD	protein_coding	NP_216137.1	Rv1621c	−14.9
mymT	Metallothionein	protein_coding	YP_004837046.2	Rv0186A	−14.3

^*a*^ DEG, differentially expressed gene; fc, fold change.

^*b*^ “–” represents genes that are nucleic acids and therefore do not have a protein ID.

**TABLE 2:** Top 25 upregulated genes on *Mtb* H37Rv-infected MRC-5 fibroblasts by RNA seq analysis

Gene symbol	Description	Gene biotype	Protein ID	Infected/un-infected MRC-5.fc
NEAT1	Nuclear paraspeckle assembly transcript 1	lncRNA	–^*a*^	144.3
ESM1	Endothelial cell-specific molecule 1	protein_coding	NP_001129076.1; NP_008967.1	130.9
CXCL8	C-X-C motif chemokine ligand 8	protein_coding	NP_000575.1; NP_001341769.1	98.4
LOC105369370	Uncharacterized LOC105369370	lncRNA	–	64.5
IFI44L	Interferon-induced protein 44 like	protein_coding	NP_001362575.1; NP_001362576.1; NP_001362577.1; NP_001362578.1; NP_001362579.1; NP_006811.2; XP_006710367.1	62.8
LOC107987083	Uncharacterized LOC107987083, transcript variant X2	lncRNA	–	48.6
IL1A	Interleukin 1 alpha	protein_coding	NP_000566.3; NP_001358483.1	46.3
MX2	MX dynamin-like GTPase 2	protein_coding	NP_002454.1; XP_005261040.1; XP_005261041.1; XP_011527873.1; XP_011527874.1; XP_011527875.1; XP_011527876.1; XP_024307848.1	45.1
HMGB1P3	High mobility group box 1 pseudogene 3	pseudogene	–	37.7
LOC105375914	Uncharacterized LOC105375914, transcript variant X6	lncRNA	–	34.5
VTA1P2	Vesicle trafficking 1 pseudogene 2	pseudogene	–	34.0
IL1B	Interleukin 1 beta	protein_coding	NP_000567.1; XP_016859477.1	33.9
MKRN5P	Makorin ring finger protein 5, pseudogene	pseudogene	–	30.8
OR6A2	Olfactory receptor family 6 subfamily A member 2	protein_coding	NP_003687.2	30.8
HAS2	Hyaluronan synthase 2	protein_coding	NP_005319.1	30.7
LOC105375247	Uncharacterized LOC105375247	lncRNA	–	29.2
RPL36AP42	Ribosomal protein L36a pseudogene 42	pseudogene	–	28.2
OR2AG2	Olfactory receptor family 2 subfamily AG member 2	protein_coding	NP_001004490.1	27.1
LOC105374179	Uncharacterized LOC105374179	lncRNA	–	27.0
LOC100129577	Mitochondrial carrier 1 pseudogene	pseudogene	–	26.7
ACTBP7	ACTB pseudogene 7	pseudogene	–	26.6
NUDCP1	Nuclear distribution C pseudogene 1	pseudogene	–	26.1
MIR8058	MicroRNA 8058	miRNA	–	25.8
RPL7AP42	Ribosomal protein L7a pseudogene 42	pseudogene	–	25.7
LOC105375310	Uncharacterized LOC105375310	lncRNA	–	25.3

^*a*^ “–” represents genes that are nucleic acids and therefore do not have a protein ID.

Fold change (fc.) of caspase-1 mRNA was not observed in the fibroblasts, but caspase-4 mRNA was induced in the fibroblasts by *Mtb* infection (fc. 5.9; Table S2). Fold change of caspase-9 mRNA, an initiator of apoptosis (10, 11), but not that of caspase-3, was observed in the fibroblasts and downregulated by *Mtb* infection (fc. −2.1; Table S2). NLRP3 mRNA was upregulated by *Mtb* infection (fc. 2.3; Table S2). These results indicated that NLRP3 and caspase-4 contribute to the cell death of the fibroblasts caused by the infection with *Mtb* bacilli.

## DISCUSSION

In this study, we first analyzed the model of host cell death induced by live *Mtb* bacilli with cell death analytic methods involving caspases and inflammasome inhibitors, and we discussed the role of fibroblasts in *Mtb* infection. We confirmed the cell death of three embryonic fibroblast cell lines derived from different origins, by live, but not dead *Mtb* bacilli, with a device for measuring cell growth/cytotoxicity in real time, Maestro-Z (AXION). Live *Mtb* bacilli induced cell death in fibroblasts in a time- and bacterial number-dependent manner; however, cell death was not observed on day 1 (Fig. 1), which was the same as in our previous study (2). Although we have reported the cytotoxicity of *Mtb* H37Rv, lineage 4, and clinical isolates of *Mtb* bacilli (1); in this study, we also confirmed that another *Mtb* strain, HN878 lineage 2, induced cytotoxicity in fibroblasts (Fig. 1D).

In their review, Randall et al. wrote the “non-classical immune cell” plays an important role in the host in case of *Mtb* infection (12). They summarized reports of interactions of *Mtb* with epithelial cells, endothelial cells, fibroblasts, adipocytes, glia, and neurons, which work with the “classical immune cells,” myeloid and lymphoid cells, to achieve an optimal immune outcome favoring the host. We previously reported that live *Mtb* induced marked cytotoxicity to the “non-classical immune cell,” human lung fibroblast cell lines MRC-5, MRC-9, and TIG-1 and the human lung epithelial cell line A549, compared with the “classical immune cell,” the human myeloid cell line HL-60 and lymphoid cell lines U937 and THP-1 (1, 2). Dead *Mtb* bacilli also induced cell death in HL-60, U937, and THP-1 cells, but not in fibroblasts and epithelial cells. In this study, we confirmed live, but not dead *Mtb* bacilli induced cytotoxicity to MRC-5, MRC-9, and TIG-1 fibroblasts (Fig. 1).

Fibroblasts also acted as macrophages and ingested both live and dead *Mtb* bacilli (Fig. 2; Fig. S1 and S2), and host cell death was observed 2 days after infection (Fig. 1). The expression of mRNA of the inflammatory cytokines, IL-1β, IL-6, and IL-8, was observed at day 2 (Fig. 4; Fig. S4). MRC-5, MRC-9, and TIG-1 fibroblast cell death induced by live *Mtb* bacilli 2 days after infection was specifically inhibited by VX-765, an inhibitor of caspase-1/4, and MCC950, an inhibitor of NLRP3 inflammasome, but not by PAC-1, a caspase-3 inhibitor, and z-LEHD-fmk, a caspase-9 inhibitor (Fig. 1 and 3). RNA-Seq analysis also revealed that mRNAs of NLRP3 and caspase-4, but not caspase-3, were induced in *Mtb*-infected fibroblasts. These results suggest that live, but not dead, *Mtb* bacilli induce fibroblast cell death by activating the inflammasome. Taken together, these results suggest that live *Mtb* bacilli induce pyroptosis (7) in fibroblasts and that pyroptosis due to fibroblast infection with *Mtb* bacilli promotes activation of immune system cells via signaling to “classical immune cells,” resulting in the pathogen being excluded by the host.

In this study, IL-18 was not detected (Fig. 5; Table S2), but other studies have shown that IL-18 was induced in pyroptosis in lipopolysaccharide-induced fibroblasts (13), suggesting that there may be another, as yet unknown, mechanism of cell death induced by live *Mtb*.

Other roles of fibroblasts in TB include acting as a reservoir of *Mtb* bacilli and supporting their active replication (14). Fibroblasts are also integrated into granulomas, where they control the *Mtb* infection by regulating immune responses (6). One study investigated the deposition of collagen produced by fibroblasts within granulomas in connection with the virulence of *Mtb* bacilli. Although H37Ra, an avirulent strain, induced collagen synthesis, H37Rv, a virulent strain, induced progressive collagen degradation via matrix metalloproteinases (MMPs), which promote cavity formation and diffusion of *Mtb* bacilli (15). Another study reported that *Mtb* bacilli upregulate MMP-1 in an NF-κb-dependent manner (16). In our study, dual RNA-Seq showed that NF-κB was activated (Fig. S5h) and MMP-1 was induced (fc. 3.0) by *Mtb* H37Rv infection (Table S2).

Next, we investigated the cyclopedic gene expression of *Mtb* in fibroblasts (Fig. 6) and *Mtb*-infected host cells (Fig. 7) at day 2 by the dual RNA-Seq method (8) because *Mtb* were ingested by fibroblasts, and host cell cytotoxicity appeared at day 2 after infection (Fig. 1 and 2). GO analysis of biological processes in *in vivo Mtb* bacilli in fibroblasts indicated that the expression of genes related to DNA-templated transcription and hypoxia response was highly induced compared with *in vitro Mtb* cultured in broth (Fig. 6a). GO analysis of molecular functions showed that the expressions of genes related to oxidoreductase activity, monooxygenase activity, and iron ion binding were augmented in *in vivo Mtb* (Fig. 6b). The KEGG pathway analysis showed that the expression of steroid degradation genes was induced in the *in vivo* bacilli, a finding that was supported by the classification into steroid-degrading bacteria, including *Mycobacterium*, *Rhodococcus,* and *Pseudomonas* (Fig. c). Expression of genes related to benzoate degradation was observed in anaerobic bacteria (Fig. 6c), which was related to the augmented expression of genes related to responses to hypoxia in the GO analysis of biological processes (Fig. 6a).

The bacillus, genus *Mycobacterium*, has a thick cell wall containing mycolic acid, which consists of a long-chain fatty acid. The bacilli are thought to obtain carbon by fatty acid degradation in the oligotrophic environment at the time of latent infection of the host cell. In addition, the bacilli require iron ions for a metabolic oxidation-reduction reaction. These results strongly suggest that *in vivo Mtb* survives within the host cell by adapting to the hypoxic oligotrophic environment (Fig. 6c).

The genes *trcR*, *secE2, ahpD*, and *mazF8* were highly induced in *in vivo Mtb* bacilli according to top 50 differentially expressed gene analysis (Table 1). The T-cell response regulator (TrcR) encoded by *tcrR* belongs to a two-component system and has been reported to bind an AT-rich sequence upstream of Rv1057, which was expressed during early infection of human macrophages (17). The biological function of Rv1057 is not well known, but a knockout study of Rv1057 found that it regulated the mycobacterium ESAT-6 secretion system (18). In our study, Rv1057 was not listed in the dual RNA-Seq analysis, so we assumed that TrcR regulated other genes related to the survival of the bacilli in MRC-5 fibroblasts. SecE2 is a calcium-binding protein (19) and an abundant protein and mRNA in *Mtb* and stimulates cytokine production from human peripheral blood mononuclear cells (20). The enzyme alkylhydroperoxidase D (*ahpD*) contributes to survival in the host cell by resisting host cell oxidative stress during infection, not only with *Mtb* (21–25) but also with other *Mycobacterium* (26, 27) and with *Streptococcus pneumoniae* (28) and *Legionella pneumophila* (29). MazF and RelE play a role as regulators of translocation by cleaving mRNA as a toxin-antitoxin (TA) family under unfavorable growth conditions in *E. coli* (30, 31). TA loci are conserved widely in prokaryotes, and the *Mtb* genome includes many more TA loci than other bacterial species; indeed, *Mtb* H37Rv and CDC1551 genomes include 38 and 26 TA loci, respectively (32). In our study, the expressions of *mazF* and *relE*, encoding a toxin RelE, genes were enhanced *in vivo* (by fc. 13.6 and 5.9, respectively), which have contributed to the survival of the *Mtb* bacilli in the host cells (Table S1).

On the other hand, the GO analysis of biological processes showed that the expressions of genes related to metabolism were generally increased in the host cell (Fig. 7a). The results of the GO analysis of molecular function on the host side indicated that the expression of genes related to binding and protein binding was highly induced by *Mtb* infection (Fig. 7b). The GO analysis of cellular components clearly indicated that the expression of genes related to intracellular anatomical structure, organelles, intracellular and membrane-bound organelles, and cytoplasm was highly induced by *Mtb* infection. These results strongly suggest that the structure of the cell membrane and intracellular organelles is changed in fibroblasts that take up the *Mtb* bacilli (Fig. 7c); this hypothesis is supported by morphological observation (Fig. 2; Fig. S1 and S2).

The list of the top 25 upregulated cytokine genes included IL-8/CXCL8 and IL-1β (Table 2). In addition, the genes of chemokines, such as CXCL1, CXCL2, and CXCL3, were also upregulated (fc. 18.7, 19.8, and 24.2, respectively; Table S2). The gene expressions of *IL-6*, an inflammatory cytokine, and *IRAK2* and *IL-6ST*, cytokine signaling molecules, were also induced in the infected cells (fc. 14.0, 3.2, and 2.3, respectively; Table S2). The gene expression of *has2* was highly upregulated in the infected cells (Table 2). Interestingly, a study of the toxicity of antibiotic levofloxacin found that apoptosis of fibroblast-like synoviocytes induced caspase-3 in the study of toxicity of levofloxacin (33). During the cell death caused by the antibiotic, *has2* gene expression and production of both IL-1 and IL-6 were downregulated. These results were opposite to our results but suggested that the cell death induced by *Mtb* bacilli infection is different from that caused by chemical compounds. Several researchers showed an anti-mycobacterial role of fibroblasts against *Mtb* infection in that the production of IL-8/CXCL8 by fibroblasts limited the growth of *in vivo Mtb* bacilli (5, 6). Our findings suggest that pyroptosis of fibroblasts contributes to host defenses against *Mtb* infection.

Findings about communication between fibroblasts and T cells through major histocompatibility complex (MHC-II) in *Mtb* infection are contradictory, that is, one study found augmentation of antigen presentation (34), whereas another found reduction of MHC-II expression (14). Because our RNA-Seq analysis showed that the MHC-II-related genes HLA-DPB1, encoding a MHC-II DP beta 1, and CIITA, encoding a MHC-II transactivator, were downregulated (fc. −2.0 and −2.5, respectively), it is unlikely that antigen presentation through MHC-II was not involved in the cell death of fibroblasts.

*Mtb* infection induced cell death in not only A549 but also HeLa cells, an epithelial cell line. The results of the experiments with the CRISPRi library (kindly provided by Prof. Yamasaki, Osaka University, Osaka, Japan) of HeLa cells suggested that caspase-3 contributes to host cell death by *Mtb* infection (unpublished data). Therefore, pyroptosis in fibroblasts may be different from apoptosis in other cells but may still play a role in defense against *Mtb* bacilli infection.

### Limitations of this study

This study examined the biological response to bacterial infection at the cellular level, and the relationship with other cells in *in vivo* immune responses needs to be investigated using other methods.

## SUPPLEMENTAL MATERIAL

Fig. S1 msphere.00110-25-s0001.tiff Fibroblast uptakes *Mycobacterium* bacilli.

Fig. S2 msphere.00110-25-s0002.tif Fibroblast uptakes both live and dead *Mycobacterium* bacilli.

Fig. S3 msphere.00110-25-s0003.tiff Effect of inhibitors for NLRP3 inflammasome, caspase-1/4, caspase-3, and caspase-9.

Fig. S4 msphere.00110-25-s0004.tiff mRNA expression of inflammatory cytokines.

Fig. S5 msphere.00110-25-s0005.tif The activation of transcription of IL-6 and IL-8 genes.

Table S1 msphere.00110-25-s0006.pdf DEG in *in vivo* vs *in vitro Mycobacterium tuberculosis*.

Table S2 msphere.00110-25-s0007.pdf DEG in MRC-5 fibroblasts infected vs uninfected with *Mycobacterium tuberculosis*.

ASM does not own the copyrights to Supplemental Material that may be linked to, or accessed through, an article. The authors have granted ASM a non-exclusive, world-wide license to publish the Supplemental Material files. Please contact the corresponding author directly for reuse.
