# Supplementary figures and images for: Mycobacterium tuberculosis bacillus induces pyroptosis in human lung fibroblasts
Source: mSphere. 2025 May 19;10(6):e00110-25. doi: 10.1128/msphere.00110-25 (PMC12188705; doi:10.1128/msphere.00110-25)

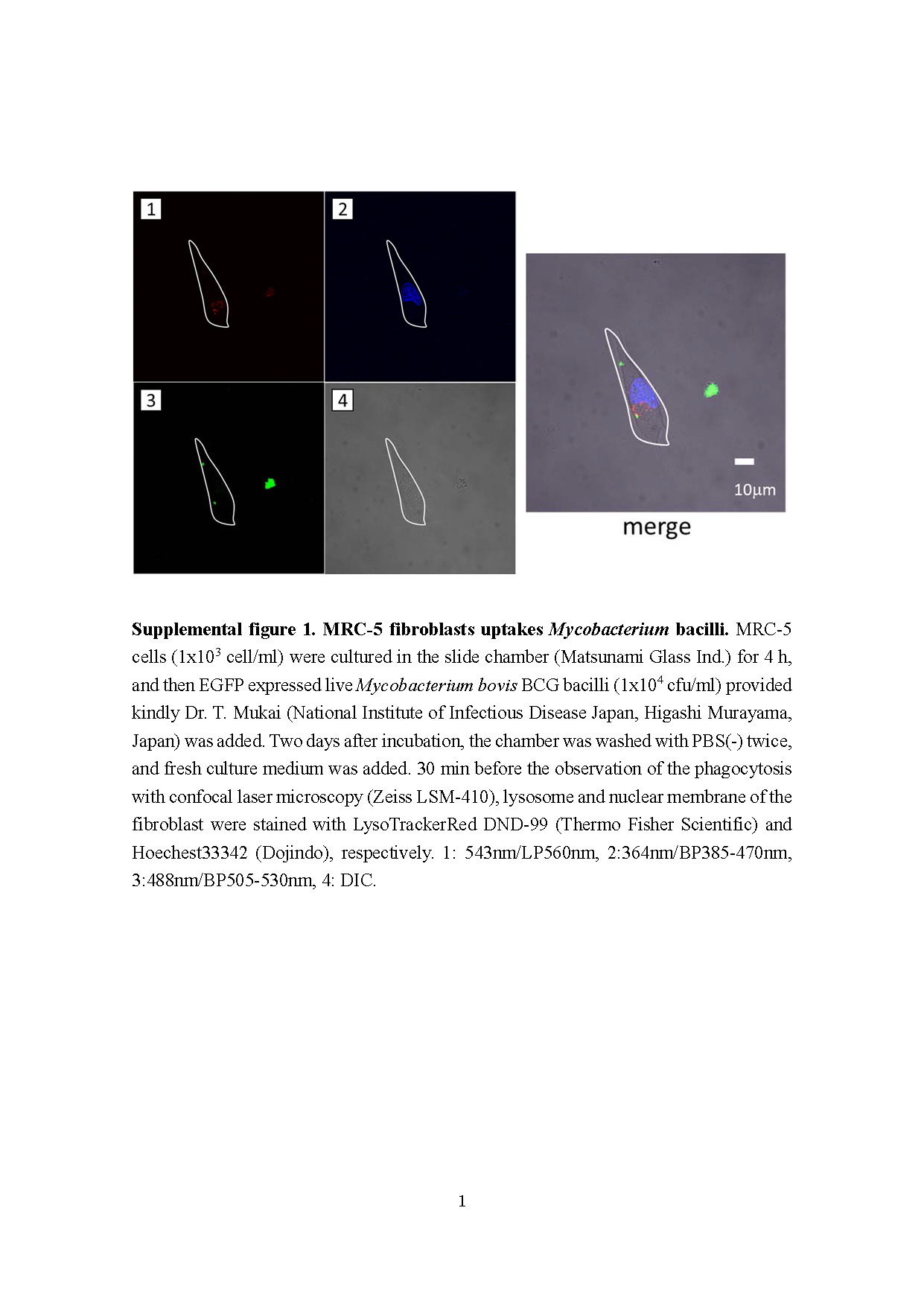

Supplement: Fig. S1 — Fibroblast uptakes Mycobacterium bacilli. [file msphere.00110-25-s0001.tiff]

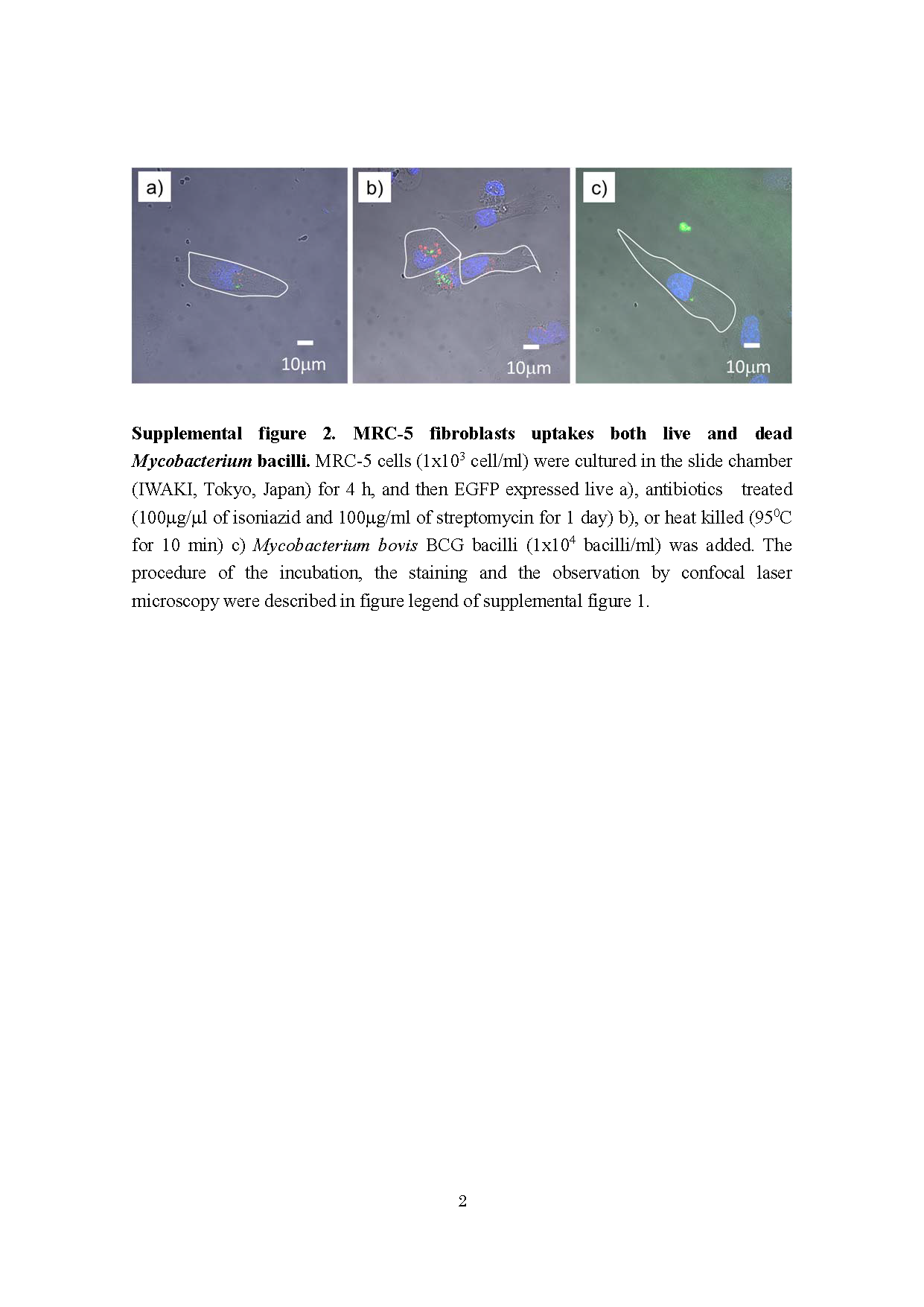

Supplement: Fig. S2 — Fibroblast uptakes both live and dead Mycobacterium bacilli. [file msphere.00110-25-s0002.tif]

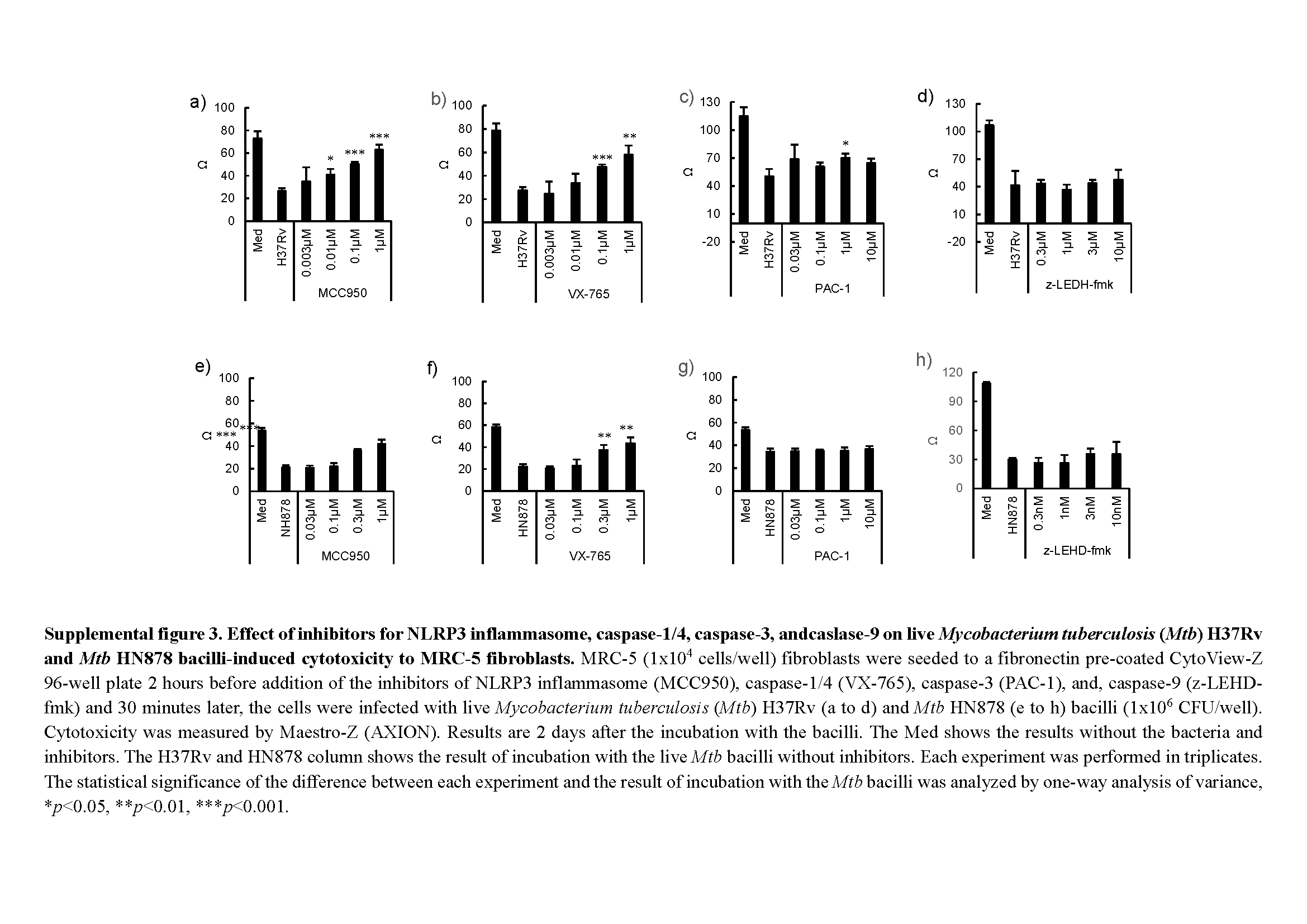

Supplement: Fig. S3 — Effect of inhibitors for NLRP3 inflammasome, caspase-1/4, caspase-3, and caspase-9. [file msphere.00110-25-s0003.tiff]

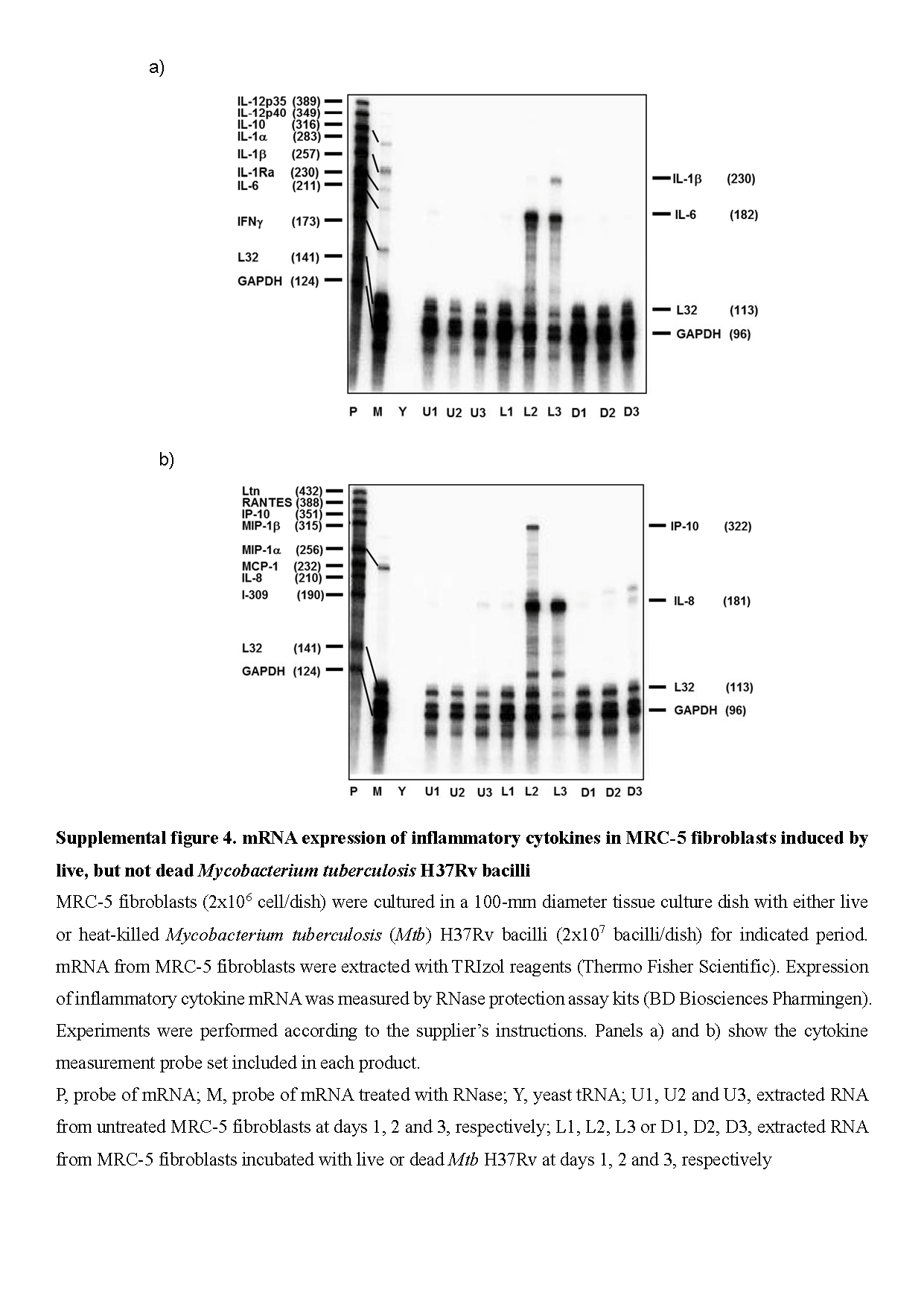

Supplement: Fig. S4 — mRNA expression of inflammatory cytokines. [file msphere.00110-25-s0004.tiff]
