# Supplementary material for: Mycobacterium tuberculosis bacillus induces pyroptosis in human lung fibroblasts
Source: mSphere. 2025 May 19;10(6):e00110-25. doi: 10.1128/msphere.00110-25 (PMC12188705; doi:10.1128/msphere.00110-25)
Supplement: Table S1 — DEG in in vivo vs in vitro Mycobacterium tuberculosis. [file msphere.00110-25-s0006.pdf]

Supplemental table 1.The differentially expressed genes in *in vivo* vs *in vitro Mycobacterium tuberculosis* H37Rv by RNA-Seq analysis

Gene_Symbol	Description	gene_biotype	Protein_ID	locus_tag	*in vivo/in vitro Mtb* H37Rv, .fc.
alaT	tRNA-Ala	tRNA	.	Rvnt02	24.802849
trcR	two component transcriptional regulator TrcR	protein_coding	NP_215549.1	Rv1033c	23.031411
leuU	tRNA-Leu	tRNA	.	Rvnt22	21.818790
secE2	protein translocase subunit SecE	protein_coding	YP_177722.1	Rv0379	18.985651
.	diterpene synthase	protein_coding	NP_217895.1	Rv3378c	17.226567
ahpD	alkyl hydroperoxide reductase AphD	protein_coding	NP_216945.1	Rv2429	17.146443
nuoK	NADH-quinone oxidoreductase subunit K	protein_coding	NP_217671.1	Rv3155	15.576579
.	hypothetical protein	protein_coding	NP_216787.1	Rv2271	15.473702
.	hypothetical protein	protein_coding	NP_216893.1	Rv2377c	14.536577
.	hypothetical protein	protein_coding	NP_215867.1	Rv1351	14.084116
.	hypothetical protein	protein_coding	NP_216326.1	Rv1810	13.894661
mazF8	toxin MazF8	protein_coding	NP_216790.1	Rv2274c	13.623012
pntAb	NAD(P) transhydrogenase subunit alpha PntAb	protein_coding	NP_214670.1	Rv0156	13.416302
rpmG2	50S ribosomal protein L33	protein_coding	YP_177630.1	Rv0634B	13.196506
thrT	tRNA-Thr	tRNA	.	Rvnt06	12.282840
metT	tRNA-Met	tRNA	.	Rvnt07	12.208965
.	hypothetical protein	protein_coding	NP_217476.1	Rv2960c	11.640723
ahpC	alkyl hydroperoxide reductase subunit AhpC	protein_coding	NP_216944.1	Rv2428	11.129833
.	hypothetical protein	protein_coding	NP_217472.1	Rv2956	10.886956
.	hypothetical protein	protein_coding	NP_216968.1	Rv2452c	10.785273
moaB1	pterin-4-alpha-carbinolamine dehydratase	protein_coding	YP_177926.1	Rv3110	10.759306
.	hypothetical protein	protein_coding	NP_216031.1	Rv1515c	10.758493
tesA	thioesterase TesA	protein_coding	NP_217444.1	Rv2928	10.751730
papA3	acyltransferase papA3	protein_coding	NP_215698.1	Rv1182	10.655143
vapB39	antitoxin VapB39	protein_coding	YP_177672.1	Rv2530A	10.502311
.	succinate dehydrogenase iron-sulfur subunit	protein_coding	NP_214761.1	Rv0247c	10.436349
vapB33	antitoxin VapB33	protein_coding	NP_215757.1	Rv1241	10.230090
vapB13	antitoxin VapB13	protein_coding	NP_216355.1	Rv1839c	10.222296
vapB15	antitoxin VapB15	protein_coding	NP_216525.1	Rv2009	9.876137
.	hypothetical protein	protein_coding	NP_216783.1	Rv2267c	9.793624
vapB35	antitoxin VapB35	protein_coding	YP_007410641.1	Rv1962A	9.711600
.	HTH-type transcriptional regulator KstR2	protein_coding	NP_218074.1	Rv3557c	9.668780
.	monooxygenase	protein_coding	NP_215407.1	Rv0892	9.478244
.	chitinase	protein_coding	NP_216503.1	Rv1987	9.362704
ileT	tRNA-Ile	tRNA	.	Rvnt01	9.299853
.	hypothetical protein	protein_coding	NP_217630.1	Rv3114	9.177521
rpsR1	30S ribosomal protein S18	protein_coding	YP_177688.1	Rv0055	9.047773
glyT	tRNA-Gly	tRNA	.	Rvnt32	8.876694
fadD30	long-chain-fatty-acid--AMP ligase FadD30	protein_coding	NP_214918.1	Rv0404	8.831906
moaD1	molybdenum cofactor biosynthesis protein MoaD	protein_coding	YP_177928.1	Rv3112	8.799897
.	hypothetical protein	protein_coding	NP_216948.1	Rv2432c	8.710993
.	hypothetical protein	protein_coding	NP_216017.1	Rv1501	8.615196
.	type B diterpene cyclase	protein_coding	NP_217894.1	Rv3377c	8.030195
.	hypothetical protein	protein_coding	NP_217445.1	Rv2929	8.015346
fadD21	fatty-acid--CoA ligase FadD21	protein_coding	NP_215701.1	Rv1185c	8.013268
rpsQ	30S ribosomal protein S17	protein_coding	NP_215224.1	Rv0710	7.972215
.	hypothetical protein	protein_coding	NP_216967.1	Rv2451	7.583218
hadC	(3R)-hydroxyacyl-ACP dehydratase subunit HadC	protein_coding	NP_215151.1	Rv0637	7.557938
.	hypothetical protein	protein_coding	YP_177629.1	Rv0634A	7.555253
.	hypothetical protein	protein_coding	NP_218336.1	Rv3819	7.456951
.	hypothetical protein	protein_coding	NP_215632.1	Rv1116	7.391729
umaA	mycolic acid synthase UmaA	protein_coding	YP_177729.1	Rv0469	7.361234
proU	tRNA-Pro	tRNA	.	Rvnt26	7.230015
lysU	tRNA-Lys	tRNA	.	Rvnt30	7.182788
papA1	acyltransferase	protein_coding	NP_218341.1	Rv3824c	7.149042
.	hypothetical protein	protein_coding	NP_216847.1	Rv2331	7.006863
.	cell division protein CrgA	protein_coding	NP_214525.1	Rv0011c	6.989187
.	hypothetical protein	protein_coding	NP_215982.1	Rv1466	6.978513
papA2	trehalose-2-sulfate acyltransferase	protein_coding	YP_178020.1	Rv3820c	6.968229
.	CRISPR-associated protein Csm6	protein_coding	NP_217334.1	Rv2818c	6.965228
.	hypothetical protein	protein_coding	NP_215080.1	Rv0566c	6.957764
argV	tRNA-Arg	tRNA	.	Rvnt18	6.927153
relB	antitoxin RelB	protein_coding	NP_215763.1	Rv1247c	6.858248
esxA	ESAT-6 protein EsxA	protein_coding	YP_178023.1	Rv3875	6.789914
moaC1	cyclic pyranopterin monophosphate synthase accessory	protein_coding	YP_177927.1	Rv3111	6.749470
vapB48	antitoxin VapB48	protein_coding	YP_007412423.1	Rv3697A	6.616326

.	hypothetical protein	protein_coding	NP_217734.1	Rv3218	6.588217
.	CRISPR type III-associated RAMP protein Csm5	protein_coding	NP_217335.1	Rv2819c	6.561559
.	adhesion component ABC transporter permease	protein_coding	NP_215502.1	Rv0987	6.536183
.	hypothetical protein	protein_coding	NP_216320.1	Rv1804c	6.522477
.	CRISPR type III-associated protein Csm2	protein_coding	NP_217338.1	Rv2822c	6.479103
moaD2	cyclic pyranopterin monophosphate synthase	protein_coding	NP_215383.1	Rv0868c	6.450529
mazE6	antitoxin MazE6	protein_coding	YP_007410673.1	Rv1991A	6.442637
lppA	lipoprotein LppA	protein_coding	NP_217059.1	Rv2543	6.347584
.	hypothetical protein	protein_coding	YP_177681.1	Rv2970A	6.342764
.	hypothetical protein	protein_coding	NP_216270.1	Rv1754c	6.332638
mazE4	antitoxin MazE4	protein_coding	NP_216010.1	Rv1494	6.308178
.	hypothetical protein	protein_coding	YP_009030027.1	Rv0115a	6.258527
.	hypothetical protein	protein_coding	NP_218045.1	Rv3528c	6.247672
ssr	10Sa RNA	misc_RNA	.	RVnc0046	6.221729
bioF2	8-amino-7-oxononanoate synthase	protein_coding	NP_214546.1	Rv0032	6.209574
mazF3	mRNA interferase MazF3	protein_coding	NP_215618.1	Rv1102c	6.184099
moaA1	cyclic pyranopterin monophosphate synthase	protein_coding	YP_177925.1	Rv3109	6.151751
.	glutaredoxin protein	protein_coding	YP_177941.1	Rv3198A	6.138835
rpmC	50S ribosomal protein L29	protein_coding	NP_215223.1	Rv0709	6.067248
sugC	sugar ABC transporter ATP-binding protein SugC	protein_coding	NP_215754.1	Rv1238	6.040018
vapB28	antitoxin VapB28	protein_coding	NP_215122.1	Rv0608	5.992746
.	integral membrane protein	protein_coding	NP_214822.1	Rv0308	5.980107
lrpA	transcriptional regulator LrpA	protein_coding	NP_217808.1	Rv3291c	5.948074
PPE1	PPE family protein PPE1	protein_coding	YP_177690.1	Rv0096	5.908978
.	membrane protein	protein_coding	NP_216419.1	Rv1903	5.902358
.	hypothetical protein	protein_coding	NP_216021.1	Rv1505c	5.896682
.	hypothetical protein	protein_coding	NP_214969.1	Rv0455c	5.846314
vapB43	antitoxin VapB43	protein_coding	NP_217387.1	Rv2871	5.827737
fadD23	long-chain-fatty-acid--CoA ligase FadD23	protein_coding	NP_218343.1	Rv3826	5.772770
.	excisionase	protein_coding	NP_218267.1	Rv3750c	5.697236
.	hypothetical protein	protein_coding	YP_177647.1	Rv1498A	5.695954
vapC44	ribonuclease VapC44	protein_coding	NP_217837.1	Rv3320c	5.573847
.	hypothetical protein	protein_coding	NP_216018.1	Rv1502	5.569232
.	integral membrane protein	protein_coding	NP_217239.1	Rv2723	5.471464
fcoT	fatty acyl CoA thioesterase FcoT	protein_coding	NP_214612.1	Rv0098	5.455987
.	ABC transporter permease	protein_coding	NP_216202.1	Rv1686c	5.441934
vapC36	ribonuclease VapC36	protein_coding	NP_216498.1	Rv1982c	5.436357
kgtP	.	other	.	Rv3476c	5.422084
.	rhamnosyl O-methyltransferase	protein_coding	NP_217475.1	Rv2959c	5.417904
.	CRISPR-associated endoribonuclease Cas2	protein_coding	NP_217332.1	Rv2816c	5.413283
acpM	meromycolate extension acyl carrier protein	protein_coding	NP_216760.1	Rv2244	5.409313
relE	toxin RelE	protein_coding	NP_215762.1	Rv1246c	5.386362
.	hypothetical protein	protein_coding	NP_217250.1	Rv2734	5.364584
.	hypothetical protein	protein_coding	NP_216517.1	Rv2001	5.342241
moeW	molybdopterin biosynthesis protein MoeW	protein_coding	NP_216854.1	Rv2338c	5.335586
.	hypothetical protein	protein_coding	NP_217324.1	Rv2808	5.335281
.	ferredoxin	protein_coding	NP_216302.1	Rv1786	5.333124
fadD18	fatty-acid--CoA ligase FadD18	protein_coding	NP_218030.1	Rv3513c	5.330385
mpt64	immunogenic protein Mpt64	protein_coding	NP_216496.1	Rv1980c	5.293718
hadA	(3R)-hydroxyacyl-ACP dehydratase subunit HadA	protein_coding	NP_215149.1	Rv0635	5.268616
proY	tRNA-Pro	tRNA	.	Rvnt40	5.185077
pckA	phosphoenolpyruvate carboxykinase	protein_coding	NP_214725.1	Rv0211	5.184945
.	antitoxin	protein_coding	NP_215424.1	Rv0909	5.157043
.	transcriptional regulator	protein_coding	NP_215409.1	Rv0894	5.130101
valT	tRNA-Val	tRNA	.	Rvnt31	5.097272
.	transcriptional regulator	protein_coding	NP_215875.1	Rv1359	5.094471
secG	protein-export membrane protein SecG	protein_coding	NP_215956.2	Rv1440	5.075483
.	hypothetical protein	protein_coding	NP_215503.1	Rv0988	5.057704
.	PemK-like protein	protein_coding	YP_007411812.1	Rv3098A	5.036054
trpT	tRNA-Trp	tRNA	.	Rvnt08	5.013887
.	hypothetical protein	protein_coding	NP_217294.1	Rv2778c	5.010643
whiB3	redox-responsive transcriptional regulator WhiB3	protein_coding	NP_217933.1	Rv3416	4.945571
pks9	polyketide synthase	protein_coding	NP_216180.1	Rv1664	4.933193
.	hypothetical protein	protein_coding	NP_218005.1	Rv3488	4.927084
lppX	lipoprotein LppX	protein_coding	NP_217461.1	Rv2945c	4.910416
.	transmembrane protein	protein_coding	NP_216644.1	Rv2128	4.881606
pks11	chalcone synthase	protein_coding	NP_216181.1	Rv1665	4.878984
moeB2	molybdenum cofactor biosynthesis protein MoeB	protein_coding	YP_177929.1	Rv3116	4.806564
PE25	PE family protein PE25	protein_coding	YP_177882.1	Rv2431c	4.802480
.	hypothetical protein	protein_coding	YP_177619.1	Rv0236A	4.765143
.	hypothetical protein	protein_coding	NP_216023.1	Rv1507c	4.741874

ureA	urease subunit gamma	protein_coding	NP_216364.1	Rv1848	4.732725
vapB19	antitoxin VapB19	protein_coding	NP_217063.1	Rv2547	4.727534
trcS	two component sensor histidine kinase TrcS	protein_coding	NP_215548.1	Rv1032c	4.714836
rplX	50S ribosomal protein L24	protein_coding	NP_215229.1	Rv0715	4.707429
cdh	CDP-diacylglycerol pyrophosphatase	protein_coding	NP_216805.1	Rv2289	4.703824
.	hypothetical protein	protein_coding	NP_216827.1	Rv2311	4.691469
.	CRISPR-associated endonuclease Cas1	protein_coding	NP_217333.1	Rv2817c	4.680412
.	hypothetical protein	protein_coding	NP_218264.1	Rv3747	4.644461
iniB	isoniazid inducible protein IniB	protein_coding	NP_214855.1	Rv0341	4.601577
.	monooxygenase	protein_coding	NP_215079.1	Rv0565c	4.599494
mmpL9	transmembrane transport protein MmpL9	protein_coding	NP_216855.1	Rv2339	4.597946
mmaA3	methoxy mycolic acid synthase MmaA3	protein_coding	NP_215157.1	Rv0643c	4.554796
lpqT	lipoprotein LpqT	protein_coding	NP_215532.1	Rv1016c	4.535502
.	hypothetical protein	protein_coding	NP_215346.1	Rv0831c	4.532681
.	hypothetical protein	protein_coding	NP_215465.1	Rv0950c	4.495636
serX	tRNA-Ser	tRNA	.	Rvnt42	4.477648
mmpL2	transmembrane transport protein MmpL2	protein_coding	NP_215021.1	Rv0507	4.475575
.	mycofactocin precursor	protein_coding	YP_007409348.2	Rv0691A	4.468208
relJ	antitoxin RelJ	protein_coding	NP_217874.1	Rv3357	4.465192
mmpS2	membrane protein MmpS2	protein_coding	NP_215020.1	Rv0506	4.454056
gmhA	phosphoheptose isomerase	protein_coding	NP_214627.1	Rv0113	4.451697
nrdF1	ribonucleoside-diphosphate reductase subunit beta NrdI	protein_coding	YP_177853.1	Rv1981c	4.435092
.	Mce associated protein	protein_coding	NP_218009.1	Rv3492c	4.430977
mku	non-homologous end joining protein Ku	protein_coding	NP_215452.1	Rv0937c	4.416881
.	hypothetical protein	protein_coding	NP_217752.1	Rv3235	4.404812
cmaA1	cyclopropane mycolic acid synthase CmaA	protein_coding	NP_217909.1	Rv3392c	4.391920
.	hypothetical protein	protein_coding	NP_216494.1	Rv1978	4.383199
.	nucleoside triphosphate pyrophosphohydrolase	protein_coding	NP_215537.1	Rv1021	4.355718
.	hypothetical protein	protein_coding	YP_007408717.1	Rv0078B	4.350092
lppB	lipoprotein LppB	protein_coding	NP_217060.1	Rv2544	4.339131
.	hypothetical protein	protein_coding	NP_216769.1	Rv2253	4.337772
.	hypothetical protein	protein_coding	NP_215648.1	Rv1132	4.335632
esxB	ESAT-6-like protein EsxB	protein_coding	NP_218391.1	Rv3874	4.333650
.	hypothetical protein	protein_coding	NP_216022.1	Rv1506c	4.326884
vapC8	ribonuclease VapC8	protein_coding	NP_215179.1	Rv0665	4.318914
.	hypothetical protein	protein_coding	YP_009030041.1	Rv2512a	4.315162
rpsO	30S ribosomal protein S15	protein_coding	NP_217301.1	Rv2785c	4.312756
relK	toxin RelK	protein_coding	NP_217875.1	Rv3358	4.308668
.	hypothetical protein	protein_coding	YP_009030028.1	Rv0997a	4.304417
.	hypothetical protein	protein_coding	NP_216025.1	Rv1509	4.298788
.	hypothetical protein	protein_coding	NP_218416.1	Rv3899c	4.271595
.	hypothetical protein	protein_coding	NP_215406.1	Rv0891c	4.270612
.	ATP-dependent Clp protease adapter protein ClpS	protein_coding	NP_215847.1	Rv1331	4.252118
vapB10	antitoxin VapB10	protein_coding	NP_215914.1	Rv1398c	4.246651
relF	antitoxin RelF	protein_coding	NP_217381.1	Rv2865	4.243819
.	glycosyltransferase	protein_coding	NP_216030.1	Rv1514c	4.240178
.	hypothetical protein	protein_coding	NP_216583.1	Rv2067c	4.234534
hadB	(3R)-hydroxyacyl-ACP dehydratase subunit HadB	protein_coding	NP_215150.1	Rv0636	4.227344
MTS1082	Putative small regulatory RNA	ncRNA	.	RVnc0035	4.203406
rplI	50S ribosomal protein L9	protein_coding	NP_214570.1	Rv0056	4.201328
.	S-adenosylmethionine-dependent methyltransferase	protein_coding	NP_215408.1	Rv0893c	4.173642
drrC	daunorubicin ABC transporter permease DrrC	protein_coding	NP_217454.1	Rv2938	4.151760
vapC33	ribonuclease VapC33	protein_coding	NP_215758.1	Rv1242	4.133971
trxA	thioredoxin TrxA	protein_coding	NP_215986.1	Rv1470	4.111319
.	hypothetical protein	protein_coding	NP_216240.1	Rv1724c	4.097771
.	hypothetical protein	protein_coding	NP_214571.1	Rv0057	4.084468
xthA	exodeoxyribonuclease III protein XthA	protein_coding	NP_214941.1	Rv0427c	4.071271
.	phosphatase	protein_coding	NP_217893.1	Rv3376	4.068955
vapC9	ribonuclease VapC9	protein_coding	NP_215475.1	Rv0960	4.065907
rpfC	resuscitation-promoting factor RpfC	protein_coding	NP_216400.1	Rv1884c	4.039355
.	cytochrome c oxidase polypeptide 4	protein_coding	NP_216715.1	Rv2199c	4.034017
iniA	isoniazid inductible protein IniA	protein_coding	NP_214856.1	Rv0342	4.033737
alaV	tRNA-Ala	tRNA	.	Rvnt15	4.033612
.	hypothetical protein	protein_coding	NP_216389.1	Rv1873	4.030320
.	two-component system transcriptional regulator	protein_coding	NP_216142.1	Rv1626	4.018026
.	transmembrane protein	protein_coding	NP_215070.1	Rv0556	4.004221
vapB37	antitoxin VapB37	protein_coding	NP_216620.1	Rv2104c	4.002745
.	phthiotriol/phenolphthiotriol dimycocerosates methyltra	protein_coding	NP_217468.1	Rv2952	3.994308
gca	GDP-mannose 4,6-dehydratase	protein_coding	NP_214626.1	Rv0112	3.992592
rplW	50S ribosomal protein L23	protein_coding	NP_215217.1	Rv0703	3.982502
.	hypothetical protein	protein_coding	NP_217705.1	Rv3189	3.965106

glyS	glycine--tRNA ligase	protein_coding	NP_216873.1	Rv2357c	3.952279
.	adhesion component ABC transporter ATP-binding pro	protein_coding	NP_215501.1	Rv0986	3.928995
mmpL4	transmembrane transport protein MmpL4	protein_coding	NP_214964.1	Rv0450c	3.923321
whiB1	transcriptional regulator WhiB1	protein_coding	NP_217735.1	Rv3219	3.902632
.	methyltransferase	protein_coding	NP_216014.1	Rv1498c	3.879419
fadE31	acyl-CoA dehydrogenase FadE31	protein_coding	NP_218079.1	Rv3562	3.877439
vapC21	ribonuclease VapC21	protein_coding	NP_217273.1	Rv2757c	3.874736
sodA	superoxide dismutase	protein_coding	NP_218363.1	Rv3846	3.863242
lppH	lipoprotein LppH	protein_coding	YP_177991.1	Rv3576	3.857366
.	hypothetical protein	protein_coding	NP_216852.1	Rv2336	3.852451
.	membrane protein insertion efficiency factor	protein_coding	NP_218439.1	Rv3922c	3.839399
mmpL1	transmembrane transport protein MmpL1	protein_coding	NP_214916.1	Rv0402c	3.833479
metV	tRNA-Met	tRNA	.	Rvnt24	3.828213
.	hypothetical protein	protein_coding	NP_216061.1	Rv1545	3.817662
.	hypothetical protein	protein_coding	YP_009030034.1	RV1984a	3.811637
ftsX	cell division protein FtsX	protein_coding	NP_217617.1	Rv3101c	3.810928
atpE	ATP synthase subunit C	protein_coding	NP_215821.1	Rv1305	3.805376
.	GCN5-like N-acetyltransferase	protein_coding	NP_217291.3	Rv2775	3.803346
secY	preprotein translocase SecY	protein_coding	NP_215246.1	Rv0732	3.797835
.	hypothetical protein	protein_coding	NP_214870.1	Rv0356c	3.795376
.	hypothetical protein	protein_coding	NP_215340.1	Rv0825c	3.775146
.	hypothetical protein	protein_coding	NP_216828.1	Rv2312	3.757133
lprN	Mce family lipoprotein LprN	protein_coding	NP_218012.1	Rv3495c	3.754758
leuX	tRNA-Leu	tRNA	.	Rvnt17	3.749485
.	phosphatase	protein_coding	NP_217629.1	Rv3113	3.747276
vapC32	ribonuclease VapC32	protein_coding	NP_215630.1	Rv1114	3.721015
TB16.3	hypothetical protein	protein_coding	NP_216701.1	Rv2185c	3.720780
.	membrane protein	protein_coding	NP_215045.1	Rv0531	3.717753
sigD	ECF RNA polymerase sigma factor SigD	protein_coding	NP_217931.1	Rv3414c	3.717214
purC	phosphoribosylaminoimidazole-succinocarboxamide sy	protein_coding	NP_215294.1	Rv0780	3.706136
sugB	sugar ABC transporter permease SugB	protein_coding	NP_215753.1	Rv1237	3.697373
.	hypothetical protein	protein_coding	NP_218283.1	Rv3766	3.690740
.	membrane protein	protein_coding	NP_216719.1	Rv2203	3.661388
.	hypothetical protein	protein_coding	NP_214655.1	Rv0141c	3.649225
.	dioxygenase	protein_coding	NP_215428.1	Rv0913c	3.635657
.	hypothetical protein	protein_coding	NP_217549.1	Rv3033	3.621012
canA	beta-carbonic anhydrase	protein_coding	NP_215800.1	Rv1284	3.617832
lprI	lipoprotein LprI	protein_coding	NP_216057.1	Rv1541c	3.613264
.	endopeptidase	protein_coding	NP_216706.1	Rv2190c	3.611634
tcrX	two component transcriptional regulator TcrX	protein_coding	NP_218282.1	Rv3765c	3.611076
.	membrane protein	protein_coding	NP_218374.1	Rv3857c	3.609735
.	4a-hydroxytetrahydrobiopterin dehydratase	protein_coding	YP_177641.1	Rv1159A	3.608388
.	hypothetical protein	protein_coding	NP_215644.1	Rv1128c	3.607148
mutT2	8-oxo-dGTP diphosphatase	protein_coding	NP_215676.1	Rv1160	3.592290
.	hypothetical protein	protein_coding	NP_218380.1	Rv3863	3.579399
esxC	ESAT-6 like protein EsxC	protein_coding	NP_218407.1	Rv3890c	3.578473
corA	magnesium and cobalt transport transmembrane proteir	protein_coding	NP_215755.1	Rv1239c	3.566997
cadI	cadmium inducible protein CadI	protein_coding	NP_217157.1	Rv2641	3.555699
.	hypothetical protein	protein_coding	NP_214938.1	Rv0424c	3.555035
rplN	50S ribosomal protein L14	protein_coding	NP_215228.1	Rv0714	3.554144
.	sugar ABC transporter permease	protein_coding	NP_216556.1	Rv2040c	3.522219
rpsS	30S ribosomal protein S19	protein_coding	NP_215219.1	Rv0705	3.508517
esxW	ESAT-6 like protein EsxW	protein_coding	NP_218137.1	Rv3620c	3.492902
mmpS4	membrane protein MmpS4	protein_coding	NP_214965.1	Rv0451c	3.483041
mscL	large-conductance ion mechanosensitive channel	protein_coding	NP_215500.1	Rv0985c	3.477124
.	hypothetical protein	protein_coding	YP_177649.1	Rv1508A	3.467544
.	oxidoreductase	protein_coding	NP_215776.1	Rv1260	3.445352
.	hypothetical protein	protein_coding	NP_214823.1	Rv0309	3.437253
TB15.3	iron-regulated universal stress protein	protein_coding	NP_216152.1	Rv1636	3.429218
.	hypothetical protein	protein_coding	NP_215193.1	Rv0679c	3.424149
glf	UDP-galactopyranose mutase	protein_coding	NP_218326.1	Rv3809c	3.415666
.	hypothetical protein	protein_coding	NP_217149.1	Rv2633c	3.415193
.	hypothetical protein	protein_coding	YP_177645.1	Rv1489	3.414447
.	GtrA family protein	protein_coding	NP_218306.1	Rv3789	3.405921
recF	DNA replication/repair protein RecF	protein_coding	NP_214517.1	Rv0003	3.400089
.	hypothetical protein	protein_coding	NP_215264.1	Rv0750	3.386955
.	IS2-like transposase	protein_coding	NP_215558.1	Rv1042c	3.386368
.	oxidoreductase	protein_coding	NP_214611.1	Rv0097	3.378361
.	hypothetical protein	protein_coding	NP_216296.1	Rv1780	3.373910
rpsF	30S ribosomal protein S6	protein_coding	NP_214567.1	Rv0053	3.371312
.	hypothetical protein	protein_coding	NP_216047.1	Rv1531	3.370604

.	hypothetical protein	protein_coding	NP_215872.1	Rv1356c	3.364685
.	membrane protein	protein_coding	NP_214562.1	Rv0048c	3.356074
smpB	SsrA-binding protein	protein_coding	NP_217616.1	Rv3100c	3.343782
arfA	peptidoglycan-binding protein ArfA	protein_coding	NP_215414.1	Rv0899	3.342907
.	hypothetical protein	protein_coding	NP_215371.1	Rv0856	3.342442
.	oxidoreductase	protein_coding	NP_218070.1	Rv3553	3.338862
glbO	hemoglobin GlbO	protein_coding	NP_216986.1	Rv2470	3.332613
.	membrane protein	protein_coding	YP_177969.1	Rv3395A	3.327182
sdhD	succinate dehydrogenase hydrophobic membrane ancho	protein_coding	NP_217834.1	Rv3317	3.326127
.	hypothetical protein	protein_coding	NP_215314.1	Rv0799c	3.324788
argA	L-glutamate alpha-N-acetyltranferase	protein_coding	NP_217263.1	Rv2747	3.315901
PPE41	PPE family protein PPE41	protein_coding	YP_177881.1	Rv2430c	3.306582
arsA	arsenic-transport integral membrane protein ArsA	protein_coding	NP_217200.1	Rv2684	3.304132
.	membrane protein	protein_coding	NP_215992.1	Rv1476	3.299570
rpfE	resuscitation-promoting factor RpfE	protein_coding	NP_216966.1	Rv2450c	3.298430
.	hypothetical protein	protein_coding	NP_217325.1	Rv2809	3.295869
.	anion transporter ATPase	protein_coding	NP_218196.1	Rv3679	3.291226
.	glutamine ABC transporter ATP-binding protein	protein_coding	NP_214587.1	Rv0073	3.290096
.	hypothetical protein	protein_coding	NP_218335.1	Rv3818	3.287247
hisT	tRNA-His	tRNA	.	Rvnt29	3.284391
greA	transcription elongation factor GreA	protein_coding	NP_215596.1	Rv1080c	3.276189
.	hypothetical protein	protein_coding	NP_217224.1	Rv2708c	3.276022
.	membrane protein	protein_coding	NP_216083.1	Rv1567c	3.270368
vapC13	ribonuclease VapC13	protein_coding	NP_216354.1	Rv1838c	3.242786
.	glyoxalase II	protein_coding	NP_215148.1	Rv0634c	3.242055
.	hypothetical protein	protein_coding	NP_214944.1	Rv0430	3.241435
.	hypothetical protein	protein_coding	NP_216277.1	Rv1761c	3.241105
vapB29	antitoxin VapB29	protein_coding	YP_007409270.1	Rv0616A	3.230976
.	hypothetical protein	protein_coding	NP_216491.1	Rv1975	3.227589
atpC	ATP synthase subunit epsilon	protein_coding	NP_215827.1	Rv1311	3.223635
parD2	antitoxin ParD2	protein_coding	YP_007410825.1	Rv2142A	3.219062
dnaB	replicative DNA helicase	protein_coding	NP_214572.1	Rv0058	3.211203
.	hypothetical protein	protein_coding	YP_009030039.1	Rv2386a	3.203372
cmaA2	cyclopropane mycolic acid synthase	protein_coding	NP_215017.1	Rv0503c	3.200501
sucD	succinyl-CoA ligase subunit alpha	protein_coding	NP_215467.1	Rv0952	3.198170
PE_PGRS55	PE-PGRS family protein PE_PGRS55	protein_coding	YP_177980.1	Rv3511	3.197550
.	anti-anti-sigma factor	protein_coding	NP_215030.1	Rv0516c	3.191996
PPE33	PPE family protein PPE33	protein_coding	YP_177845.1	Rv1809	3.191124
.	sugar ABC transporter permease	protein_coding	NP_216555.1	Rv2039c	3.179823
.	hypothetical protein	protein_coding	NP_216743.1	Rv2227	3.174243
PPE6	PPE family protein PPE6	protein_coding	YP_177715.1	Rv0305c	3.166212
.	membrane protein	protein_coding	NP_214993.1	Rv0479c	3.160215
lppE	lipoprotein LppE	protein_coding	NP_216397.1	Rv1881c	3.154024
ufaA1	cyclopropane-fatty-acyl-phospholipid synthase UfaA	protein_coding	NP_214961.1	Rv0447c	3.149544
lppM	lipoprotein LppM	protein_coding	NP_216687.1	Rv2171	3.140422
dinP	DNA polymerase IV 2	protein_coding	NP_217572.1	Rv3056	3.137431
.	hypothetical protein	protein_coding	YP_177665.1	Rv2307A	3.133413
.	membrane protein	protein_coding	NP_217036.1	Rv2520c	3.125982
.	HTH-type transcriptional regulator	protein_coding	NP_216332.1	Rv1816	3.125652
.	cyclase	protein_coding	NP_216951.1	Rv2435c	3.124183
rpsN1	30S ribosomal protein S14	protein_coding	YP_177747.1	Rv0717	3.117939
.	hypothetical protein	protein_coding	YP_009030031.1	Rv1155a	3.114599
.	CRISPR type III-associated RAMP protein Csm3	protein_coding	NP_217337.1	Rv2821c	3.108752
pitB	phosphate permease	protein_coding	NP_216797.1	Rv2281	3.084965
.	hypothetical protein	protein_coding	NP_215568.1	Rv1052	3.079563
mcr3	Putative small regulatory RNA	ncRNA	.	RVnc0021	3.078857
fadD29	long-chain-fatty-acid--AMP ligase FadD29	protein_coding	NP_217466.3	Rv2950c	3.075931
dipZ	integral membrane C-type cytochrome biogenesis prote	protein_coding	NP_217390.1	Rv2874	3.073129
PE36	PE family protein PE36	protein_coding	YP_178025.1	Rv3893c	3.070126
.	hypothetical protein	protein_coding	NP_217414.1	Rv2898c	3.067063
.	membrane protein	protein_coding	NP_216006.1	Rv1490	3.065798
vapC3	ribonuclease VapC3	protein_coding	NP_215063.1	Rv0549c	3.065485
.	hypothetical protein	protein_coding	YP_009030035.1	Rv2104a	3.065340
.	short-chain type dehydrogenase/reductase	protein_coding	NP_218066.1	Rv3549c	3.065005
.	hypothetical protein	protein_coding	NP_217828.1	Rv3311	3.064716
vapC31	ribonuclease VapC31	protein_coding	NP_215263.1	Rv0749	3.050734
.	hypothetical protein	protein_coding	YP_178004.1	Rv3678A	3.048243
lysS	lysine--tRNA ligase	protein_coding	NP_218115.1	Rv3598c	3.046055
.	hypothetical protein	protein_coding	NP_216399.1	Rv1883c	3.045199
mprA	two-component response regulator MrpA	protein_coding	NP_215496.2	Rv0981	3.021104
pks6	membrane bound polyketide synthase	protein_coding	NP_214919.1	Rv0405	3.018918

.	membrane protein	protein_coding	NP_215878.1	Rv1362c	3.004720
.	hypothetical protein	protein_coding	NP_217199.1	Rv2683	2.999360
vapC11	ribonuclease VapC11	protein_coding	NP_216077.1	Rv1561	2.987452
lpqQ	lipoprotein LpqQ	protein_coding	NP_215350.1	Rv0835	2.982527
vapB47	antitoxin VapB47	protein_coding	NP_217924.1	Rv3407	2.979896
vapB40	antitoxin VapB40	protein_coding	NP_217111.1	Rv2595	2.975538
cyp128	cytochrome P450 Cyp128	protein_coding	NP_216784.1	Rv2268c	2.972173
.	Mce associated protein	protein_coding	NP_214691.1	Rv0177	2.970598
.	L-gulono-1,4-lactone dehydrogenase	protein_coding	NP_216287.1	Rv1771	2.963757
pntB	NAD(P) transhydrogenase subunit beta PntB	protein_coding	NP_214671.1	Rv0157	2.963072
.	HTH-type transcriptional regulator	protein_coding	NP_214710.1	Rv0196	2.959062
fadD28	long-chain-fatty-acid--AMP ligase FadD28	protein_coding	NP_217457.1	Rv2941	2.956161
esxH	ESAT-6-like protein EsxH	protein_coding	NP_214802.1	Rv0288	2.955018
rpmH	50S ribosomal protein L34	protein_coding	NP_218441.1	Rv3924c	2.950113
dapC	N-succinyldiaminopimelate aminotransferase DapC	protein_coding	NP_215373.1	Rv0858c	2.949340
fadD34	fatty-acid--CoA ligase FadD34	protein_coding	YP_177686.1	Rv0035	2.947640
pstA1	phosphate ABC transporter permease PstA	protein_coding	NP_215445.2	Rv0930	2.947511
.	hypothetical protein	protein_coding	NP_216192.1	Rv1676	2.945711
.	cyclo(L-tyrosyl-L-tyrosyl) synthase	protein_coding	NP_216791.1	Rv2275	2.943047
vapB2	antitoxin VapB2	protein_coding	NP_214814.1	Rv0300	2.939502
glpD1	glycerol-3-phosphate dehydrogenase	protein_coding	NP_216765.1	Rv2249c	2.933448
.	hypothetical protein	protein_coding	NP_216278.1	Rv1762c	2.931803
atpF	ATP synthase subunit B	protein_coding	NP_215822.1	Rv1306	2.929492
.	hypothetical protein	protein_coding	NP_217251.1	Rv2735c	2.922543
rpsT	30S ribosomal protein S20	protein_coding	NP_216928.1	Rv2412	2.921832
.	transmembrane protein	protein_coding	NP_217794.1	Rv3277	2.920520
.	hypothetical protein	protein_coding	NP_216853.1	Rv2337c	2.919629
infA	translation initiation factor IF-1	protein_coding	NP_217979.1	Rv3462c	2.917125
.	toxin	protein_coding	NP_215425.1	Rv0910	2.913204
.	hypothetical protein	protein_coding	NP_218272.1	Rv3755c	2.910813
mmaA4	hydroxymycolate synthase MmaA4	protein_coding	NP_215156.1	Rv0642c	2.907716
.	hypothetical protein	protein_coding	NP_217443.1	Rv2927c	2.903926
mazF6	mRNA interferase MazF6	protein_coding	NP_216507.1	Rv1991c	2.902015
.	hypothetical protein	protein_coding	NP_215777.1	Rv1261c	2.873553
aroG	phospho-2-dehydro-3-deoxyheptonate aldolase AroG	protein_coding	NP_216694.1	Rv2178c	2.872552
vapC17	ribonuclease VapC17	protein_coding	NP_217043.1	Rv2527	2.867629
mmaA2	cyclopropane mycolic acid synthase CmaA	protein_coding	NP_215158.1	Rv0644c	2.860611
.	endonuclease NucS	protein_coding	NP_215837.1	Rv1321	2.851137
TB18.5	hypothetical protein	protein_coding	YP_177617.1	Rv0164	2.850206
.	hypothetical protein	protein_coding	NP_216347.1	Rv1831	2.845860
.	hypothetical protein	protein_coding	NP_216939.1	Rv2423	2.830802
sapM	acid phosphatase	protein_coding	NP_217827.1	Rv3310	2.829657
secA1	protein translocase subunit SecA	protein_coding	YP_177950.1	Rv3240c	2.819415
.	beta lactamase-like protein	protein_coding	NP_214920.1	Rv0406c	2.819335
.	hypothetical protein	protein_coding	NP_216516.1	Rv2000	2.809609
.	hypothetical protein	protein_coding	NP_214826.1	Rv0312	2.806828
.	S-adenosylmethionine-dependent methyltransferase	protein_coding	NP_216245.1	Rv1729c	2.803260
vapC48	ribonuclease VapC48	protein_coding	NP_218214.1	Rv3697c	2.802622
.	hypothetical protein	protein_coding	NP_216198.1	Rv1682	2.796833
.	hypothetical protein	protein_coding	NP_217007.1	Rv2491	2.778412
yajC	membrane protein secretion factor YajC	protein_coding	NP_217104.1	Rv2588c	2.776207
bacA	vitamin B12 transport ATP-binding protein BacA	protein_coding	NP_216335.1	Rv1819c	2.776106
.	hypothetical protein	protein_coding	NP_216804.1	Rv2288	2.774741
.	hypothetical protein	protein_coding	NP_216104.1	Rv1588c	2.774579
adk	adenylate kinase	protein_coding	NP_215247.1	Rv0733	2.773945
.	anti-sigma-D factor RsdA	protein_coding	NP_217930.1	Rv3413c	2.762547
PPE59	PPE family protein PPE59	protein_coding	YP_177973.1	Rv3429	2.755682
.	hypothetical protein	protein_coding	NP_217776.1	Rv3259	2.752872
.	antibiotic-resistance protein	protein_coding	NP_216819.1	Rv2303c	2.748180
hab	hydroxylaminobenzene mutase	protein_coding	NP_217594.1	Rv3078	2.741567
entC	isochorismate synthase	protein_coding	NP_217731.1	Rv3215	2.736700
.	HTH-type transcriptional regulator	protein_coding	NP_214986.1	Rv0472c	2.735162
eccA2	ESX-2 secretion system protein EccA	protein_coding	NP_218401.1	Rv3884c	2.733427
.	hypothetical protein	protein_coding	YP_178016.1	Rv3796	2.730119
.	hypothetical protein	protein_coding	NP_217591.1	Rv3075c	2.726300
fadE32	acyl-CoA dehydrogenase FadE32	protein_coding	NP_218080.1	Rv3563	2.723492
hisI	phosphoribosyl-AMP cyclohydrolase	protein_coding	NP_216638.2	Rv1606	2.719747
fdxC	ferredoxin FdxC	protein_coding	NP_215693.1	Rv1177	2.715409
.	transcriptional regulator	protein_coding	NP_214672.1	Rv0158	2.715004
prpC	methylcitrate synthase PrpC	protein_coding	NP_215647.1	Rv1131	2.702509
.	short-chain type dehydrogenase/reductase	protein_coding	NP_214662.1	Rv0148	2.702252

.	hypothetical protein	protein_coding	NP_217748.1	Rv3231c	2.695977
ndkA	nucleoside diphosphate kinase	protein_coding	NP_216961.1	Rv2445c	2.695672
.	transposase	protein_coding	NP_217307.1	Rv2791c	2.692301
.	hypothetical protein	protein_coding	NP_216029.1	Rv1513	2.691285
atpB	ATP synthase subunit A	protein_coding	NP_215820.1	Rv1304	2.690326
dprE2	decaprenylphosphoryl-D-2-keto erythropentose reducta	protein_coding	NP_218308.1	Rv3791	2.688172
.	hypothetical protein	protein_coding	NP_215670.1	Rv1154c	2.681554
.	nitrogen fixation related protein	protein_coding	NP_215981.1	Rv1465	2.681376
pup	ubiquitin-like protein Pup	protein_coding	NP_216627.1	Rv2111c	2.679441
thrV	tRNA-Thr	tRNA	.	Rvnt09	2.662730
dnaA	chromosomal replication initiator protein DnaA	protein_coding	NP_214515.1	Rv0001	2.662581
.	phage protein	protein_coding	NP_216100.1	Rv1584c	2.657780
.	hypothetical protein	protein_coding	NP_216934.1	Rv2418c	2.652699
cyp136	cytochrome P450 Cyp136	protein_coding	NP_217575.1	Rv3059	2.651887
.	HTH-type transcriptional regulator	protein_coding	NP_215405.1	Rv0890c	2.651587
.	hypothetical protein	protein_coding	NP_216820.1	Rv2304c	2.650920
vapB16	antitoxin VapB16	protein_coding	YP_007410918.1	Rv2231B	2.649805
tatD	deoxyribonuclease TatD	protein_coding	NP_215524.1	Rv1008	2.649383
.	hypothetical protein	protein_coding	NP_218419.1	Rv3902c	2.641876
cyp121	cytochrome P450 Cyp121	protein_coding	NP_216792.1	Rv2276	2.639336
.	hypothetical protein	protein_coding	NP_215955.1	Rv1439c	2.633258
pgsA3	CDP-diacylglycerol--glycerol-3-phosphate 3-phosphati	protein_coding	NP_217262.1	Rv2746c	2.632228
espR	ESX-1 transcriptional regulator EspR	protein_coding	NP_218366.1	Rv3849	2.621227
.	Lrp/AsnC family transcriptional regulator	protein_coding	NP_217295.2	Rv2779c	2.619508
vapB24	antitoxin VapB24	protein_coding	NP_214753.1	Rv0239	2.619042
.	adenylate cyclase	protein_coding	NP_215836.1	Rv1320c	2.615703
.	membrane protein	protein_coding	NP_218405.1	Rv3888c	2.614529
vapC28	ribonuclease VapC28	protein_coding	NP_215123.1	Rv0609	2.613680
.	macrolide ABC transporter ATP-binding protein	protein_coding	NP_216993.1	Rv2477c	2.609953
.	hypothetical protein	protein_coding	YP_177658.1	Rv2077A	2.609468
nuoD	NADH-quinone oxidoreductase subunit D	protein_coding	NP_217664.1	Rv3148	2.609263
vapB38	antitoxin VapB38	protein_coding	NP_217009.1	Rv2493	2.601053
echA20	enoyl-CoA hydratase EchA20	protein_coding	NP_218067.1	Rv3550	2.599853
.	hypothetical protein	protein_coding	NP_217287.1	Rv2771c	2.594087
.	peptidase	protein_coding	NP_214971.1	Rv0457c	2.594069
mtr	mycothione reductase	protein_coding	YP_177910.1	Rv2855	2.592910
.	transmembrane protein	protein_coding	NP_216788.1	Rv2272	2.591368
.	hypothetical protein	protein_coding	NP_218233.1	Rv3716c	2.591320
.	transmembrane protein	protein_coding	NP_216662.1	Rv2146c	2.585680
vapC40	ribonuclease VapC40	protein_coding	NP_217112.1	Rv2596	2.579644
lppR	lipoprotein LppR	protein_coding	NP_216919.1	Rv2403c	2.578549
pks4	polyketide beta-ketoacyl synthase	protein_coding	NP_215697.1	Rv1181	2.576807
.	hypothetical protein	protein_coding	NP_215060.1	Rv0546c	2.572203
.	diacylglycerol kinase	protein_coding	NP_216768.1	Rv2252	2.570377
aspB	aspartate aminotransferase AspB	protein_coding	NP_218082.1	Rv3565	2.567743
rplU	50S ribosomal protein L21	protein_coding	NP_216958.1	Rv2442c	2.564295
.	mycofactocin biosynthesis transcriptional regulator Mft	protein_coding	NP_215205.1	Rv0691c	2.550806
.	methyltransferase	protein_coding	NP_216219.4	Rv1703c	2.549338
.	PGL/p-HBAD biosynthesis glycosyltransferase	protein_coding	NP_217473.1	Rv2957	2.548456
.	hypothetical protein	protein_coding	NP_214974.1	Rv0460	2.547869
.	PGL/p-HBAD biosynthesis glycosyltransferase	protein_coding	NP_217474.1	Rv2958c	2.546845
htpG	chaperone protein HtpG	protein_coding	NP_216815.1	Rv2299c	2.544371
lppF	lipoprotein LppF	protein_coding	NP_216437.1	Rv1921c	2.542009
PE16	PE family protein PE16	protein_coding	YP_177810.1	Rv1430	2.540381
.	hypothetical protein	protein_coding	NP_217592.1	Rv3076	2.537638
bfrA	bacterioferritin BfrA	protein_coding	NP_216392.1	Rv1876	2.537554
.	hypothetical protein	protein_coding	NP_217045.1	Rv2529	2.534542
mrr	restriction system protein	protein_coding	NP_217044.1	Rv2528c	2.533752
dnaN	DNA polymerase III subunit beta	protein_coding	NP_214516.1	Rv0002	2.531571
.	hypothetical protein	protein_coding	YP_004837058.1	Rv3032A	2.531454
fhaB	FHA domain-containing protein FhaB	protein_coding	NP_214533.1	Rv0019c	2.528992
.	integral membrane protein	protein_coding	NP_217872.1	Rv3355c	2.520706
dnaE1	DNA polymerase III subunit alpha	protein_coding	NP_216063.1	Rv1547	2.514842
.	50S ribosomal protein L28	protein_coding	YP_009030042.1	Rv2975a	2.512990
eccD1	ESX-1 secretion system protein EccD1	protein_coding	NP_218394.1	Rv3877	2.511724
tgs2	diacyglycerol O-acyltransferase	protein_coding	NP_218251.1	Rv3734c	2.510520
gmk	guanylate kinase	protein_coding	NP_215905.1	Rv1389	2.505142
cyp139	cytochrome P450 Cyp139	protein_coding	NP_216182.1	Rv1666c	2.504475
.	hypothetical protein	protein_coding	NP_217254.1	Rv2738c	2.501138
.	hypothetical protein	protein_coding	YP_007411905.1	Rv3190A	2.500701
PPE66	PPE family protein PPE66	protein_coding	YP_178009.1	Rv3738c	2.486765

.	integral membrane protein	protein_coding	NP_218338.1	Rv3821	2.484482
.	amino acid transporter	protein_coding	NP_215002.1	Rv0488	2.477636
.	hypothetical protein	protein_coding	NP_217706.1	Rv3190c	2.475904
papA5	phthiocerol/phthiodiolone dimycocerosyl transferase	protein_coding	NP_217455.1	Rv2939	2.472330
.	integral membrane protein	protein_coding	NP_216636.1	Rv2120c	2.470196
PE_PGRS20	PE-PGRS family protein PE_PGRS20	protein_coding	YP_177781.1	Rv1068c	2.466338
.	hypothetical protein	protein_coding	NP_214574.1	Rv0060	2.466315
.	hypothetical protein	protein_coding	NP_215440.1	Rv0925c	2.465654
.	membrane protein	protein_coding	NP_217115.1	Rv2599	2.465007
.	transcriptional regulator	protein_coding	NP_214556.1	Rv0042c	2.463516
.	hypothetical protein	protein_coding	YP_004837057.1	Rv2548A	2.462306
cfp2	low molecular weight antigen MTB12	protein_coding	NP_216892.1	Rv2376c	2.462140
.	hypothetical protein	protein_coding	NP_215594.1	Rv1078	2.460508
vapC30	ribonuclease VapC30	protein_coding	NP_215138.1	Rv0624	2.459235
mpt83	cell surface lipoprotein	protein_coding	NP_217389.1	Rv2873	2.456733
.	hypothetical protein	protein_coding	NP_215642.1	Rv1126c	2.452795
.	transcriptional regulator	protein_coding	NP_215990.1	Rv1474c	2.452644
sdhC	succinate dehydrogenase cytochrome B-556 subunit	protein_coding	NP_217833.1	Rv3316	2.452042
.	hypothetical protein	protein_coding	NP_215561.1	Rv1045	2.449352
.	monooxygenase	protein_coding	NP_215458.1	Rv0943c	2.449056
.	hypothetical protein	protein_coding	NP_215949.1	Rv1433	2.448899
.	glutamine ABC transporter permease	protein_coding	NP_217079.1	Rv2563	2.445980
asnB	asparagine synthetase	protein_coding	NP_216717.1	Rv2201	2.443722
.	integral membrane protein	protein_coding	NP_218146.1	Rv3629c	2.439336
mctB	copper transporter MctB	protein_coding	NP_216214.1	Rv1698	2.435612
.	GTP-binding protein	protein_coding	NP_215628.1	Rv1112	2.430443
treS	trehalose synthase/amylase TreS	protein_coding	NP_214640.1	Rv0126	2.428344
leuS	leucine--tRNA ligase	protein_coding	NP_214555.1	Rv0041	2.422768
prsA	ribose-phosphate pyrophosphokinase	protein_coding	NP_215533.1	Rv1017c	2.420944
pstS3	phosphate ABC transporter substrate-binding lipoprotei	protein_coding	YP_177768.1	Rv0928	2.417113
whiB2	transcriptional regulator WhiB2	protein_coding	NP_217777.1	Rv3260c	2.415650
treX	maltooligosyl trehalose synthase	protein_coding	YP_177821.1	Rv1564c	2.414645
mmpL10	transmembrane transport protein MmpL10	protein_coding	NP_215699.1	Rv1183	2.409460
cfp21	cutinase	protein_coding	NP_216500.1	Rv1984c	2.408900
PE_PGRS54	PE-PGRS family protein PE_PGRS54	protein_coding	YP_177979.1	Rv3508	2.403710
rpfA	resuscitation-promoting factor RpfA	protein_coding	NP_215382.1	Rv0867c	2.400706
.	AP-4-A phosphorylase	protein_coding	NP_217129.1	Rv2613c	2.399777
.	hypothetical protein	protein_coding	NP_216702.1	Rv2186c	2.399524
.	hypothetical protein	protein_coding	NP_217688.1	Rv3172c	2.390651
.	esterase	protein_coding	NP_216363.1	Rv1847	2.384611
rpsA	30S ribosomal protein S1	protein_coding	NP_216146.1	Rv1630	2.383276
.	hypothetical protein	protein_coding	NP_215528.1	Rv1012	2.383044
moaX	MoaD-MoaE fusion protein MoaX	protein_coding	YP_177959.1	Rv3323c	2.379410
pstC2	phosphate ABC transporter permease PstC	protein_coding	NP_215444.1	Rv0929	2.375793
.	hypothetical protein	protein_coding	NP_214636.1	Rv0122	2.374158
.	hypothetical protein	protein_coding	NP_215787.1	Rv1271c	2.369313
.	hypothetical protein	protein_coding	NP_214824.1	Rv0310c	2.368009
lppJ	lipoprotein LppJ	protein_coding	NP_216596.1	Rv2080	2.364213
.	hypothetical protein	protein_coding	YP_177939.1	Rv3196A	2.363009
.	glycosyltransferase	protein_coding	NP_216016.1	Rv1500	2.362382
mmpS1	membrane protein MmpS1	protein_coding	NP_214917.1	Rv0403c	2.361641
fadD3	fatty-acid--CoA ligase FadD3	protein_coding	NP_218078.1	Rv3561	2.359864
fabG3	3-alpha(or 20-beta)-hydroxysteroid dehydrogenase	protein_coding	NP_216518.1	Rv2002	2.358904
narK1	nitrate/nitrite transporter	protein_coding	NP_216845.1	Rv2329c	2.358232
.	permease	protein_coding	NP_216495.2	Rv1979c	2.353770
.	oxidoreductase	protein_coding	NP_214711.1	Rv0197	2.351013
PE3	PE family protein PE3	protein_coding	YP_177697.1	Rv0159c	2.350551
.	hypothetical protein	protein_coding	NP_217723.1	Rv3207c	2.339796
.	hypothetical protein	protein_coding	NP_214707.1	Rv0193c	2.339343
mazF5	toxin MazF5	protein_coding	NP_216458.1	Rv1942c	2.337928
.	hypothetical protein	protein_coding	NP_217726.1	Rv3210c	2.337230
.	transcriptional regulator	protein_coding	NP_214752.1	Rv0238	2.336934
deaD	ATP-dependent RNA helicase DeaD	protein_coding	NP_215769.1	Rv1253	2.333658
.	HTH-type transcriptional regulator	protein_coding	NP_216346.1	Rv1830	2.328251
.	hypothetical protein	protein_coding	NP_215633.1	Rv1117	2.327818
.	hypothetical protein	protein_coding	NP_216403.1	Rv1887	2.312350
aftC	alpha-(1->3)-arabinofuranosyltransferase	protein_coding	NP_217189.1	Rv2673	2.312186
.	hypothetical protein	protein_coding	NP_215512.1	Rv0997	2.311203
.	hypothetical protein	protein_coding	YP_177667.1	Rv2307D	2.308939
canB	carbonic anhydrase	protein_coding	NP_218105.1	Rv3588c	2.305737
PPE50	PPE family protein PPE50	protein_coding	YP_177934.1	Rv3135	2.301716

.	transmembrane protein	protein_coding	NP_215227.1	Rv0713	2.299033
.	hypothetical protein	protein_coding	NP_216764.1	Rv2248	2.293980
mbtL	acyl carrier protein MbtL	protein_coding	NP_215860.1	Rv1344	2.292356
ftsQ	cell division protein FtsQ	protein_coding	NP_216667.1	Rv2151c	2.291509
icd2	isocitrate dehydrogenase	protein_coding	NP_214580.1	Rv0066c	2.289231
.	hypothetical protein	protein_coding	NP_216294.1	Rv1778c	2.288482
.	hypothetical protein	protein_coding	YP_007408797.1	Rv0157A	2.287245
vapC27	ribonuclease VapC27	protein_coding	NP_215112.1	Rv0598c	2.285998
mazF9	mRNA interferase MazF9	protein_coding	NP_217317.1	Rv2801c	2.280182
.	membrane protein	protein_coding	NP_218149.1	Rv3632	2.279343
.	oxidoreductase	protein_coding	NP_218076.1	Rv3559c	2.274962
vapB41	antitoxin VapB41	protein_coding	YP_177673.1	Rv2601A	2.274521
.	transcriptional regulator	protein_coding	NP_216506.1	Rv1990c	2.273631
.	hypothetical protein	protein_coding	YP_177800.1	Rv1342c	2.272973
.	hypothetical protein	protein_coding	NP_217514.1	Rv2998	2.272715
.	sugar ABC transporter ATP-binding protein	protein_coding	NP_216554.1	Rv2038c	2.270211
rmlC	dTDP-4-dehydrorhamnose 3,5-epimerase	protein_coding	NP_217982.1	Rv3465	2.265116
.	cob(I)yrinic acid a,c-diamide adenosyltransferase	protein_coding	NP_215830.1	Rv1314c	2.264561
pdxH	pyridoxine/pyridoxamine 5'-phosphate oxidase	protein_coding	NP_217123.1	Rv2607	2.264502
echA9	enoyl-CoA hydratase EchA9	protein_coding	NP_215587.1	Rv1071c	2.263409
yrbE1B	membrane protein	protein_coding	NP_214682.1	Rv0168	2.263044
cyp141	cytochrome P450 Cyp141	protein_coding	NP_217637.1	Rv3121	2.260925
fadD31	fatty-acid--CoA ligase FadD31	protein_coding	NP_216441.2	Rv1925	2.260095
.	hypothetical protein	protein_coding	NP_216630.1	Rv2114	2.259289
.	hypothetical protein	protein_coding	NP_216200.1	Rv1684	2.257803
.	hypothetical protein	protein_coding	YP_004837056.2	Rv2237A	2.253455
.	transmembrane protein	protein_coding	NP_217795.1	Rv3278c	2.252265
.	hypothetical protein	protein_coding	NP_216435.1	Rv1919c	2.251070
.	hypothetical protein	protein_coding	NP_215944.1	Rv1428c	2.248518
mazE5	antitoxin MazE5	protein_coding	NP_216459.1	Rv1943c	2.246666
embR	transcriptional regulator EmbR	protein_coding	NP_215783.1	Rv1267c	2.243281
pirG	cell surface protein	protein_coding	NP_218327.1	Rv3810	2.241843
mce4A	Mce family protein Mce4A	protein_coding	YP_177977.1	Rv3499c	2.240310
.	hypothetical protein	protein_coding	NP_215541.1	Rv1025	2.240075
sucC	succinyl-CoA ligase subunit beta	protein_coding	NP_215466.1	Rv0951	2.239471
.	hypothetical protein	protein_coding	NP_218285.1	Rv3768	2.236765
.	membrane protein insertase YidC	protein_coding	NP_218438.1	Rv3921c	2.236613
.	hypothetical protein	protein_coding	NP_216917.2	Rv2401	2.234468
.	oxidoreductase	protein_coding	NP_216059.1	Rv1543	2.225549
.	hypothetical protein	protein_coding	NP_216466.1	Rv1950c	2.225238
fadE36	acyl-CoA dehydrogenase FadE36	protein_coding	NP_218278.1	Rv3761c	2.223843
.	CRISPR type III-associated RAMP protein Csm4	protein_coding	NP_217336.1	Rv2820c	2.222961
amt	ammonium transporter integral membrane protein	protein_coding	NP_217436.1	Rv2920c	2.222335
trpC	indole-3-glycerol phosphate synthase	protein_coding	NP_216127.1	Rv1611	2.220588
echA11	enoyl-CoA hydratase EchA11	protein_coding	NP_215657.1	Rv1141c	2.218747
.	chorismate pyruvate-lyase	protein_coding	NP_217465.1	Rv2949c	2.211390
bcpB	peroxiredoxin	protein_coding	NP_216124.1	Rv1608c	2.208486
.	transmembrane protein	protein_coding	NP_214642.1	Rv0128	2.207728
.	CRISPR-associated protein Cas10/Csm1	protein_coding	NP_217339.1	Rv2823c	2.206180
aceAb	isocitrate lyase AceAb	protein_coding	NP_216432.1	Rv1916	2.204777
mmaA1	mycolic acid methyltransferase MmaA1	protein_coding	NP_215159.1	Rv0645c	2.197200
ppk2	polyphosphate kinase	protein_coding	NP_217749.1	Rv3232c	2.195552
ctaE	cytochrome C oxidase subunit III	protein_coding	NP_216709.1	Rv2193	2.195532
.	hypothetical protein	protein_coding	NP_214706.1	Rv0192	2.193056
vapB5	antitoxin VapB5	protein_coding	NP_215140.1	Rv0626	2.185886
cobU	bifunctional cobinamide kinase/cobinamide phosphate ;	protein_coding	NP_214768.1	Rv0254c	2.181077
.	HTH-type transcriptional regulator	protein_coding	NP_215869.1	Rv1353c	2.179159
PPE36	PPE family protein PPE36	protein_coding	YP_177859.1	Rv2108	2.178417
.	hypothetical protein	protein_coding	NP_215576.1	Rv1060	2.177856
.	integral membrane protein	protein_coding	NP_217580.1	Rv3064c	2.177183
hisB	imidazole glycerol-phosphate dehydratase	protein_coding	NP_216117.1	Rv1601	2.174455
pgsA2	CDP-diacylglycerol--glycerol-3-phosphate 3-phosphati	protein_coding	NP_216338.1	Rv1822	2.170207
.	hydrolase	protein_coding	NP_216239.1	Rv1723	2.165796
parE1	toxin ParE1	protein_coding	NP_216475.1	Rv1959c	2.161532
gyrB	DNA gyrase subunit B	protein_coding	NP_214519.2	Rv0005	2.158804
moaR1	transcriptional regulator MoaR	protein_coding	NP_217640.1	Rv3124	2.157190
.	hypothetical protein	protein_coding	NP_217506.1	Rv2990c	2.156999
.	hypothetical protein	protein_coding	NP_215542.1	Rv1026	2.155985
treZ	malto-oligosyltrehalose trehalohydrolase	protein_coding	YP_177819.1	Rv1562c	2.155920
.	hypothetical protein	protein_coding	NP_215793.1	Rv1277	2.154956
.	hypothetical protein	protein_coding	NP_216988.1	Rv2472	2.153319

frr	ribosome recycling factor	protein_coding	NP_217398.1	Rv2882c	2.151343
nicT	nickel-transport integral membrane protein NicT	protein_coding	NP_217372.1	Rv2856	2.150549
glnB	nitrogen regulatory protein P-II	protein_coding	NP_217435.1	Rv2919c	2.148360
fadB2	3-hydroxybutyryl-CoA dehydrogenase	protein_coding	NP_214982.1	Rv0468	2.145264
.	membrane protein	protein_coding	NP_215200.1	Rv0686	2.144268
tesB1	acyl-CoA thioesterase II	protein_coding	NP_216134.1	Rv1618	2.143525
.	hypothetical protein	protein_coding	NP_215573.1	Rv1057	2.141963
.	hypothetical protein	protein_coding	NP_217093.1	Rv2577	2.140594
phoH1	phosphate starvation-inducible protein PhoH	protein_coding	YP_177874.1	Rv2368c	2.138931
cycA	D-serine/alanine/glycine transporter protein CycA	protein_coding	NP_216220.1	Rv1704c	2.138485
tatA	Sec-independent protein translocase membrane-bound p	protein_coding	NP_216610.1	Rv2094c	2.136943
cysO	sulfur carrier protein CysO	protein_coding	NP_215851.1	Rv1335	2.135167
.	hypothetical protein	protein_coding	NP_217076.1	Rv2560	2.133199
gcvH	glycine cleavage system protein H	protein_coding	NP_216342.1	Rv1826	2.127936
icd1	isocitrate dehydrogenase	protein_coding	NP_217856.1	Rv3339c	2.126257
cyp126	cytochrome P450 Cyp126	protein_coding	NP_215292.1	Rv0778	2.124607
rodA	cell division protein RodA	protein_coding	NP_214531.1	Rv0017c	2.120653
plcA	membrane-associated phospholipase A	protein_coding	NP_216867.1	Rv2351c	2.118949
.	transmembrane protein	protein_coding	NP_214878.1	Rv0364	2.115434
.	hypothetical protein	protein_coding	NP_217323.1	Rv2807	2.114664
mce4B	Mce family protein Mce4B	protein_coding	NP_218015.1	Rv3498c	2.113122
wag31	cell wall synthesis protein Wag31	protein_coding	NP_216661.1	Rv2145c	2.110221
.	hypothetical protein	protein_coding	NP_215710.1	Rv1194c	2.106355
.	decaprenyl diphosphate synthase	protein_coding	NP_216877.1	Rv2361c	2.104403
atpG	ATP synthase subunit gamma	protein_coding	NP_215825.1	Rv1309	2.104206
.	hypothetical protein	protein_coding	NP_215707.1	Rv1191	2.103938
MTS2975	Putative small regulatory RNA	ncRNA	.	RVnc0040	2.102133
.	CoA-transferase subunit alpha	protein_coding	NP_218068.1	Rv3551	2.100956
gyrA	DNA gyrase subunit A	protein_coding	NP_214520.1	Rv0006	2.100604
echA8	enoyl-CoA hydratase EchA8	protein_coding	NP_215586.1	Rv1070c	2.099110
.	hypothetical protein	protein_coding	NP_216445.1	Rv1929c	2.091001
whiB4	transcriptional regulator WhiB4	protein_coding	NP_218198.2	Rv3681c	2.077278
glnQ	glutamine ABC transporter ATP-binding protein	protein_coding	NP_217080.1	Rv2564	2.076827
mce1B	Mce family protein Mce1B	protein_coding	NP_214684.1	Rv0170	2.075984
.	hypothetical protein	protein_coding	NP_216082.1	Rv1566c	2.072571
rocA	pyrroline-5-carboxylate dehydrogenase RocA	protein_coding	NP_215703.1	Rv1187	2.072492
.	polyketide cyclase/dehydrase	protein_coding	NP_214602.1	Rv0088	2.072299
tesB2	acyl-CoA thioesterase II	protein_coding	NP_217121.1	Rv2605c	2.067320
.	hypothetical protein	protein_coding	NP_215403.1	Rv0888	2.066572
.	hypothetical protein	protein_coding	NP_215372.2	Rv0857	2.063941
pcaA	cyclopropane mycolic acid synthase	protein_coding	YP_177730.1	Rv0470c	2.045553
fecB	FeIII-dicitrate-binding periplasmic lipoprotein	protein_coding	NP_217560.1	Rv3044	2.042393
.	hypothetical protein	protein_coding	NP_218223.1	Rv3706c	2.036779
fadB5	oxidoreductase FadB	protein_coding	NP_216428.1	Rv1912c	2.036625
.	hypothetical protein	protein_coding	NP_215575.1	Rv1059	2.036038
TB9.4	hypothetical protein	protein_coding	YP_177943.1	Rv3208A	2.035087
.	3',5'-cyclic adenosine monophosphate phosphodiesteras	protein_coding	NP_215320.1	Rv0805	2.034088
pbpB	penicillin-binding membrane protein PbpB	protein_coding	NP_216679.1	Rv2163c	2.032551
.	transmembrane protein	protein_coding	NP_217709.1	Rv3193c	2.031132
.	S-adenosylmethionine-dependent methyltransferase	protein_coding	NP_218284.1	Rv3767c	2.029476
.	response regulator	protein_coding	NP_217659.1	Rv3143	2.022737
.	hypothetical protein	protein_coding	NP_216062.1	Rv1546	2.020342
.	transmembrane protein	protein_coding	NP_215511.1	Rv0996	2.019364
ppsD	phthiocerol synthesis polyketide synthase type I PpsD	protein_coding	NP_217450.1	Rv2934	2.018581
pcnA	poly(A) polymerase PcnA	protein_coding	YP_178026.1	Rv3907c	2.017518
fabG2	3-oxoacyl-ACP reductase FabG	protein_coding	NP_215866.1	Rv1350	2.014301
mtrA	two component DNA-binding response regulator MtrA	protein_coding	NP_217763.1	Rv3246c	2.013747
.	sugar transferase	protein_coding	NP_216032.2	Rv1516c	2.012564
.	hypothetical protein	protein_coding	NP_217148.1	Rv2632c	2.010625
rpsJ	30S ribosomal protein S10	protein_coding	NP_215214.1	Rv0700	2.009938
.	transmembrane protein	protein_coding	NP_218186.1	Rv3669	2.003946
.	transmembrane protein	protein_coding	NP_218296.1	Rv3779	2.003845
.	hypothetical protein	protein_coding	NP_216341.1	Rv1825	2.002825
.	transcriptional regulator	protein_coding	NP_216537.1	Rv2021c	-2.002090
PE_PGRS41	acid and phagosome regulated protein AprC	protein_coding	YP_177878.1	Rv2396	-2.002411
eccB5	ESX-5 type VII secretion system protein EccB5	protein_coding	NP_216298.1	Rv1782	-2.002839
.	TetR family transcriptional regulator	protein_coding	NP_217022.1	Rv2506	-2.006222
fadD9	fatty-acid--CoA ligase FadD9	protein_coding	NP_217106.1	Rv2590	-2.006482
cobT	nicotinate-nucleotide-dimethylbenzimidazol phosphoril	protein_coding	NP_216723.1	Rv2207	-2.009312
.	MFS-type transporter	protein_coding	NP_214705.1	Rv0191	-2.010501
clpC1	ATP-dependent protease ATP-binding subunit ClpC	protein_coding	YP_177995.1	Rv3596c	-2.011260

.	hypothetical protein	protein_coding	NP_214788.1	Rv0274	-2.011395
.	hypothetical protein	protein_coding	NP_216664.1	Rv2148c	-2.026435
cyp135B1	cytochrome P450 Cyp135B1	protein_coding	NP_215082.1	Rv0568	-2.026655
.	hypothetical protein	protein_coding	NP_216921.1	Rv2405	-2.028220
.	oxidoreductase	protein_coding	NP_217297.1	Rv2781c	-2.031046
bkdB	3-methyl-2-oxobutanoate dehydrogenase subunit beta	protein_coding	NP_217012.1	Rv2496c	-2.032976
.	transmembrane protein	protein_coding	NP_215427.1	Rv0912	-2.033168
.	acyltransferase	protein_coding	NP_215374.1	Rv0859	-2.035450
.	transposase	protein_coding	NP_217401.1	Rv2885c	-2.035584
.	oxidoreductase	protein_coding	NP_215075.1	Rv0561c	-2.035622
.	phosphotransferase	protein_coding	NP_218334.1	Rv3817	-2.041104
purL	phosphoribosylformylglycinamidine synthase 2	protein_coding	NP_215318.1	Rv0803	-2.042305
.	hypothetical protein	protein_coding	NP_216519.1	Rv2003c	-2.046329
trmD	tRNA (guanine-N1)-methyltransferase	protein_coding	NP_217422.1	Rv2906c	-2.049573
.	hypothetical protein	protein_coding	NP_217882.1	Rv3365c	-2.051218
.	hypothetical protein	protein_coding	NP_218349.1	Rv3832c	-2.061344
.	transcriptional regulator	protein_coding	NP_215258.1	Rv0744c	-2.062630
ephG	epoxide hydrolase	protein_coding	NP_217256.1	Rv2740	-2.064296
PPE25	PPE family protein PPE25	protein_coding	YP_177833.1	Rv1787	-2.067167
fadE7	acyl-CoA dehydrogenase FadE7	protein_coding	NP_214914.1	Rv0400c	-2.069052
cyp144	cytochrome P450 Cyp144	protein_coding	NP_216293.1	Rv1777	-2.074347
.	RNA pseudouridine synthase	protein_coding	NP_216227.1	Rv1711	-2.075737
folD	bifunctional methylenetetrahydrofolate dehydrogenase/	protein_coding	NP_217873.1	Rv3356c	-2.080236
.	transmembrane protein	protein_coding	NP_214940.1	Rv0426c	-2.082352
adhA	alcohol dehydrogenase A	protein_coding	NP_216378.1	Rv1862	-2.088596
alaS	alanine--tRNA ligase	protein_coding	NP_217071.1	Rv2555c	-2.089150
fdhF	formate dehydrogenase subunit alpha FdhF	protein_coding	NP_217416.1	Rv2900c	-2.089559
choD	cholesterol oxidase	protein_coding	NP_217926.1	Rv3409c	-2.089754
.	hypothetical protein	protein_coding	NP_217181.1	Rv2665	-2.090015
.	magnesium chelatase	protein_coding	NP_217366.1	Rv2850c	-2.093808
moaB2	pterin-4-alpha-carbinolamine dehydratase	protein_coding	NP_215499.1	Rv0984	-2.095079
cyp138	cytochrome P450 Cyp138	protein_coding	NP_214650.1	Rv0136	-2.096539
.	hypothetical protein	protein_coding	NP_214874.1	Rv0360c	-2.097933
mscR	S-nitrosomycothiol reductase MscR	protein_coding	NP_216775.1	Rv2259	-2.100696
PPE13	PPE family protein PPE13	protein_coding	YP_177764.1	Rv0878c	-2.102711
mycP1	membrane-anchored mycosin	protein_coding	NP_218400.1	Rv3883c	-2.111837
htpX	protease HtpX	protein_coding	NP_215077.1	Rv0563	-2.114110
.	hypothetical protein	protein_coding	NP_218200.1	Rv3683	-2.121555
.	hypothetical protein	protein_coding	NP_218270.3	Rv3753c	-2.128585
.	IS1536 family serine type transposase	protein_coding	NP_215119.2	Rv0605	-2.129234
.	transmembrane protein	protein_coding	NP_218121.2	Rv3604c	-2.129516
.	hypothetical protein	protein_coding	NP_216592.1	Rv2076c	-2.133543
.	bifunctional 2-hydroxyhepta-2,4-diene-1,7-dioate isom	protein_coding	NP_215454.1	Rv0939	-2.134678
.	hypothetical protein	protein_coding	NP_216599.1	Rv2083	-2.135384
PPE53	PPE family protein PPE53	protein_coding	YP_177937.1	Rv3159c	-2.140019
.	lipid carrier protein or keto acyl-CoA thiolase	protein_coding	NP_215429.1	Rv0914c	-2.140612
tmk	thymidylate kinase	protein_coding	NP_217764.1	Rv3247c	-2.143937
.	4-carboxymuconolactone decarboxylase	protein_coding	NP_215285.1	Rv0771	-2.145817
fmu	16S rRNA m5C967 methyltransferase	protein_coding	NP_215923.1	Rv1407	-2.148765
lpqR	lipoprotein LpqR	protein_coding	NP_215353.1	Rv0838	-2.149576
.	two component transcriptional regulator	protein_coding	NP_214709.1	Rv0195	-2.156375
.	hypothetical protein	protein_coding	NP_215559.1	Rv1043c	-2.157691
dnaQ	DNA polymerase III subunit epsilon	protein_coding	NP_218228.1	Rv3711c	-2.158846
.	hypothetical protein	protein_coding	NP_218433.1	Rv3916c	-2.162975
.	hypothetical protein	protein_coding	NP_218287.1	Rv3770c	-2.164063
.	mycofactocin biosynthesis glycosyltransferase MftF	protein_coding	NP_215210.1	Rv0696	-2.167783
atsA	arylsulfatase AtsA	protein_coding	NP_215225.1	Rv0711	-2.170475
.	antibiotic ABC transporter permease	protein_coding	NP_215972.1	Rv1456c	-2.170828
.	transcriptional regulator	protein_coding	NP_217505.1	Rv2989	-2.171888
lppD	lipoprotein LppD	protein_coding	NP_216415.1	Rv1899c	-2.173468
.	D-tyrosyl-tRNA(Tyr) deacylase	protein_coding	NP_216413.1	Rv1897c	-2.175450
.	hypothetical protein	protein_coding	NP_217773.1	Rv3256c	-2.175957
.	hypothetical protein	protein_coding	NP_218129.1	Rv3612c	-2.178448
.	transmembrane protein	protein_coding	NP_216713.1	Rv2197c	-2.181463
.	oxidoreductase	protein_coding	NP_214591.1	Rv0077c	-2.181781
.	hypothetical protein	protein_coding	NP_217216.1	Rv2700	-2.185165
pyrC	dihydroorotase	protein_coding	NP_215897.1	Rv1381	-2.187131
cmtR	HTH-type transcriptional regulator CmtR	protein_coding	NP_216510.1	Rv1994c	-2.187273
.	DNA integrity scanning protein DisA	protein_coding	NP_218103.1	Rv3586	-2.190131
.	magnesium chelatase	protein_coding	NP_215473.1	Rv0958	-2.194479
.	hypothetical protein	protein_coding	NP_216659.1	Rv2143	-2.196789

fprB	ferredoxin/ferredoxin--NADP reductase	protein_coding	NP_215401.1	Rv0886	-2.200413
.	hypothetical protein	protein_coding	NP_214550.1	Rv0036c	-2.210989
.	transcriptional activator protein	protein_coding	NP_215969.2	Rv1453	-2.211740
.	methyltransferase	protein_coding	YP_177958.1	Rv3322c	-2.212294
ribF	bifunctional riboflavin kinase /FMN adenylyltransferas	protein_coding	NP_217302.1	Rv2786c	-2.213295
thiO	thiamine biosynthesis oxidoreductase ThiO	protein_coding	NP_214929.1	Rv0415	-2.213767
sigH	ECF RNA polymerase sigma factor SigH	protein_coding	NP_217739.1	Rv3223c	-2.214044
proB	glutamate 5-kinase protein	protein_coding	NP_216955.1	Rv2439c	-2.223459
far	fatty-acid-CoA racemase	protein_coding	NP_215370.1	Rv0855	-2.226110
.	hypothetical protein	protein_coding	NP_215868.1	Rv1352	-2.228843
aac	aminoglycoside 2'-N-acetyltransferase	protein_coding	NP_214776.1	Rv0262c	-2.230270
.	hypothetical protein	protein_coding	NP_218413.1	Rv3896c	-2.237732
phoR	two component system response sensor kinase PhoR	protein_coding	NP_215272.1	Rv0758	-2.237927
PPE32	PPE family protein PPE32	protein_coding	YP_177844.1	Rv1808	-2.238882
.	hypothetical protein	protein_coding	NP_215806.1	Rv1290c	-2.241935
ilvG	acetolactate synthase large subunit IlvG	protein_coding	NP_216336.1	Rv1820	-2.244984
.	oxidoreductase	protein_coding	NP_214653.1	Rv0139	-2.248595
.	transmembrane protein	protein_coding	NP_214657.1	Rv0143c	-2.253733
.	antibiotic ABC transporter permease	protein_coding	NP_217203.1	Rv2687c	-2.256430
glyA2	serine hydroxymethyltransferase	protein_coding	NP_214584.1	Rv0070c	-2.261372
.	hypothetical protein	protein_coding	NP_216823.1	Rv2307c	-2.262323
hemA	glutamyl-tRNA reductase	protein_coding	NP_215023.1	Rv0509	-2.263185
.	hypothetical protein	protein_coding	NP_217431.1	Rv2915c	-2.267911
.	hypothetical protein	protein_coding	NP_217247.1	Rv2731	-2.268687
echA1	enoyl-CoA hydratase EchA1	protein_coding	NP_214736.1	Rv0222	-2.270544
pepA	serine protease PepA	protein_coding	NP_214639.1	Rv0125	-2.273048
egtC	amidohydrolase EgtC	protein_coding	NP_218219.1	Rv3702c	-2.273351
.	acyltransferase	protein_coding	NP_218332.1	Rv3815c	-2.277102
gshA	glutamate--cysteine ligase	protein_coding	NP_218221.1	Rv3704c	-2.283797
sahH	adenosylhomocysteinase	protein_coding	NP_217765.1	Rv3248c	-2.289158
accA1	acetyl/propionyl-CoA carboxylase subuit alpha	protein_coding	NP_217017.1	Rv2501c	-2.293384
menE	2-succinylbenzoic acid--CoA ligase	protein_coding	NP_215056.1	Rv0542c	-2.295199
pepC	M18 family aminopeptidase	protein_coding	NP_215315.1	Rv0800	-2.296900
metK	S-adenosylmethionine synthetase	protein_coding	NP_215908.1	Rv1392	-2.298480
.	acetyltransferase Pat	protein_coding	NP_215513.1	Rv0998	-2.305463
fadE16	acyl-CoA dehydrogenase FadE16	protein_coding	NP_216195.1	Rv1679	-2.307300
.	phosphoglycerate mutase	protein_coding	NP_218354.1	Rv3837c	-2.307441
mutB	methylmalonyl-CoA mutase large subunit	protein_coding	NP_216009.1	Rv1493	-2.308255
.	thioredoxin	protein_coding	NP_215040.1	Rv0526	-2.314545
.	diacylglycerol acyltransferase	protein_coding	NP_216276.1	Rv1760	-2.315933
PE_PGRS14	PE-PGRS family protein PE_PGRS14	protein_coding	YP_177761.1	Rv0834c	-2.320390
caeA	carboxylesterase A	protein_coding	NP_216740.1	Rv2224c	-2.327706
.	transposase	protein_coding	NP_218344.1	Rv3827c	-2.328929
lipZ	hydrolase	protein_coding	NP_216350.1	Rv1834	-2.339480
.	hypothetical protein	protein_coding	NP_217563.1	Rv3047c	-2.341475
cut4	cutinase	protein_coding	NP_217969.1	Rv3452	-2.342180
cysS1	cysteine--tRNA ligase	protein_coding	YP_177992.1	Rv3580c	-2.347752
rplD	50S ribosomal protein L4	protein_coding	NP_215216.1	Rv0702	-2.348277
.	membrane protein	protein_coding	NP_215520.1	Rv1004c	-2.350310
.	hypothetical protein	protein_coding	NP_215892.1	Rv1376	-2.355788
lpqK	lipoprotein LpqK	protein_coding	NP_214913.1	Rv0399c	-2.363559
atsB	arylsulfatase AtsB	protein_coding	NP_217816.1	Rv3299c	-2.372714
eccB4	ESX-4 secretion system protein EccB4	protein_coding	NP_217967.1	Rv3450c	-2.373300
fbpD	MPT51/MPB51 antigen	protein_coding	YP_178017.1	Rv3803c	-2.377197
dxs1	1-deoxy-D-xylulose 5-phosphate synthase	protein_coding	YP_177898.1	Rv2682c	-2.382280
sigF	RNA polymerase sigma factor SigF	protein_coding	NP_217803.1	Rv3286c	-2.384436
.	flavoprotein	protein_coding	NP_216333.1	Rv1817	-2.395679
aao	D-amino acid oxidase	protein_coding	NP_216421.1	Rv1905c	-2.396837
vapC47	ribonuclease VapC47	protein_coding	NP_217925.1	Rv3408	-2.399482
lprF	lipoprotein LprF	protein_coding	NP_215884.1	Rv1368	-2.399733
groEL2	molecular chaperone GroEL	protein_coding	NP_214954.1	Rv0440	-2.406610
.	integral membrane protein	protein_coding	NP_215470.1	Rv0955	-2.412723
.	DNA glycosylase	protein_coding	NP_216980.1	Rv2464c	-2.417290
serA1	D-3-phosphoglycerate dehydrogenase	protein_coding	YP_177916.1	Rv2996c	-2.421207
echA10	enoyl-CoA hydratase EchA10	protein_coding	NP_215658.1	Rv1142c	-2.429181
.	ATP-dependent DNA helicase	protein_coding	NP_217717.1	Rv3201c	-2.433677
devR	two component transcriptional regulator DevR	protein_coding	NP_217649.1	Rv3133c	-2.435487
PPE16	PPE family protein PPE16	protein_coding	YP_177790.1	Rv1135c	-2.436275
subI	sulfate ABC transporter substrate-binding lipoprotein S	protein_coding	NP_216916.1	Rv2400c	-2.440075
.	hypothetical protein	protein_coding	NP_215377.1	Rv0862c	-2.451044
murX	phospho-N-acetylmuramoyl-pentappeptidetransferase	protein_coding	NP_216672.1	Rv2156c	-2.454365

cstA	carbon starvation protein A	protein_coding	NP_217579.1	Rv3063	-2.456884
.	transmembrane protein	protein_coding	NP_217806.1	Rv3289c	-2.458532
pks1	polyketide synthase	protein_coding	NP_217462.1	Rv2946c	-2.469962
purT	phosphoribosylglycinamide formyltransferase PurT	protein_coding	NP_214903.1	Rv0389	-2.474046
.	hypothetical protein	protein_coding	NP_218216.1	Rv3699	-2.476202
.	hypothetical protein	protein_coding	NP_217490.1	Rv2974c	-2.480013
cysE	serine acetyltransferase	protein_coding	NP_216851.1	Rv2335	-2.481683
amiB1	amidase AmiB	protein_coding	YP_177956.1	Rv3306c	-2.485761
.	hypothetical protein	protein_coding	NP_217743.1	Rv3226c	-2.492730
.	integral membrane protein	protein_coding	NP_217788.1	Rv3271c	-2.502369
virS	HTH-type transcriptional regulator VirS	protein_coding	NP_217598.1	Rv3082c	-2.505775
.	benzoquinone methyltransferase	protein_coding	NP_215074.1	Rv0560c	-2.509008
ltp2	lipid transfer protein	protein_coding	NP_218057.1	Rv3540c	-2.509062
gpdA2	glycerol-3-phosphate dehydrogenase	protein_coding	NP_217498.1	Rv2982c	-2.512239
PPE26	PPE family protein PPE26	protein_coding	YP_177835.1	Rv1789	-2.514962
.	hypothetical protein	protein_coding	YP_177666.1	Rv2307B	-2.528399
mmpS5	membrane protein MmpS5	protein_coding	NP_215191.1	Rv0677c	-2.529637
ccdA	cytochrome C-type biogenesis protein CcdA	protein_coding	YP_177735.1	Rv0527	-2.531685
.	MFS-type transporter	protein_coding	NP_214551.1	Rv0037c	-2.535104
fadD8	fatty-acid--CoA ligase FadD8	protein_coding	NP_215065.1	Rv0551c	-2.543029
sigB	RNA polymerase sigma factor SigB	protein_coding	NP_217226.1	Rv2710	-2.557081
.	transmembrane protein	protein_coding	NP_215588.1	Rv1072	-2.559168
gid	16S rRNA (guanine(527)-N(7))-methyltransferase Rsm	protein_coding	NP_218436.2	Rv3919c	-2.566567
.	hypothetical protein	protein_coding	YP_007411850.1	Rv3136A	-2.567005
.	hypothetical protein	protein_coding	NP_215674.1	Rv1158c	-2.567927
plsC	bifunctional L-3-phosphoserine phosphatase/1-acyl-sn-	protein_coding	NP_216999.1	Rv2483c	-2.571712
.	hypothetical protein	protein_coding	NP_217029.1	Rv2513	-2.572004
.	hypothetical protein	protein_coding	NP_218367.1	Rv3850	-2.586117
rnhB	ribonuclease HII	protein_coding	NP_217418.1	Rv2902c	-2.587363
.	hypothetical protein	protein_coding	NP_217343.1	Rv2827c	-2.588175
.	hypothetical protein	protein_coding	NP_217721.1	Rv3205c	-2.591056
.	hypothetical protein	protein_coding	NP_215066.1	Rv0552	-2.592360
pncB1	nicotinic acid phosphoribosyltransferase PncB1	protein_coding	NP_215846.2	Rv1330c	-2.594414
PPE45	PPE family protein PPE45	protein_coding	YP_177913.1	Rv2892c	-2.594516
.	hypothetical protein	protein_coding	NP_217074.1	Rv2558	-2.601280
.	transcriptional regulator	protein_coding	NP_216289.1	Rv1773c	-2.603810
eis	enhanced intracellular survival protein	protein_coding	NP_216932.2	Rv2416c	-2.608282
gcpE	4-hydroxy-3-methylbut-2-en-1-yl diphosphate synthase	protein_coding	NP_217384.1	Rv2868c	-2.617249
.	hypothetical protein	protein_coding	NP_216830.1	Rv2314c	-2.622025
vapC24	ribonuclease VapC24	protein_coding	NP_214754.1	Rv0240	-2.631933
purF	amidophosphoribosyltransferase	protein_coding	NP_215323.1	Rv0808	-2.634046
nrdZ	vitamin B12-dependent ribonucleoside-diphosphate red	protein_coding	NP_215084.1	Rv0570	-2.643772
.	membrane protein	protein_coding	NP_215135.1	Rv0621	-2.648918
PPE54	PPE family protein PPE54	protein_coding	YP_177960.1	Rv3343c	-2.651242
PE8	PE family protein PE8	protein_coding	YP_177779.1	Rv1040c	-2.652298
.	hypothetical protein	protein_coding	NP_216813.1	Rv2297	-2.653513
.	hydratase	protein_coding	NP_214730.1	Rv0216	-2.663182
rpe	ribulose-phosphate 3-epimerase	protein_coding	NP_215924.1	Rv1408	-2.663229
PE_PGRS38	PE-PGRS family protein PE_PGRS38	protein_coding	YP_177865.1	Rv2162c	-2.667594
phyA	phytoene synthase	protein_coding	NP_217914.1	Rv3397c	-2.675786
.	hypothetical protein	protein_coding	NP_214698.1	Rv0184	-2.679005
PE_PGRS31	PE-PGRS family protein PE_PGRS31	protein_coding	YP_177832.1	Rv1768	-2.680455
pks12	polyketide synthase	protein_coding	NP_216564.2	Rv2048c	-2.683455
idsA1	multifunctional dimethylallyltransferase/geranyltranstra	protein_coding	YP_177970.1	Rv3398c	-2.689251
mshD	mycothiol acetyltransferase	protein_coding	NP_215334.1	Rv0819	-2.694207
pheA	prephenate dehydratase	protein_coding	NP_218355.1	Rv3838c	-2.700087
.	hypothetical protein	protein_coding	NP_217135.1	Rv2619c	-2.703070
ctpI	cation-transporter ATPase I	protein_coding	NP_214621.1	Rv0107c	-2.707062
metE	5-methyltetrahydropteroyltriglutamate--homocysteine r	protein_coding	NP_215649.1	Rv1133c	-2.708422
.	hypothetical protein	protein_coding	NP_217728.1	Rv3212	-2.708448
metC	O-acetylhomoserine sulfhydrylase	protein_coding	NP_217857.1	Rv3340	-2.709231
35kd_ag	hypothetical protein	protein_coding	YP_177903.1	Rv2744c	-2.718070
.	hypothetical protein	protein_coding	YP_177990.1	Rv3566A	-2.729325
.	prophage protease	protein_coding	NP_217167.1	Rv2651c	-2.742067
aftD	alpha-(1->3)-arabinofuranosyltransferase	protein_coding	NP_214750.1	Rv0236c	-2.742904
serB1	phosphoserine phosphatase SerB	protein_coding	YP_177732.1	Rv0505c	-2.745079
recN	DNA repair protein RecN	protein_coding	NP_216212.1	Rv1696	-2.749264
lhr	ATP-dependent helicase	protein_coding	NP_217813.1	Rv3296	-2.762148
.	ArsR family transcriptional regulator	protein_coding	NP_217158.1	Rv2642	-2.764352
.	HTH-type transcriptional regulator	protein_coding	NP_217611.1	Rv3095	-2.774545
.	short-chain type dehydrogenase/reductase	protein_coding	NP_216444.1	Rv1928c	-2.774855

.	MFS-type transporter	protein_coding	NP_215364.1	Rv0849	-2.776187
.	hypothetical protein	protein_coding	NP_217556.1	Rv3040c	-2.779957
hsaB	flavin-dependent monooxygenase reductase subunit Hs	protein_coding	NP_218084.1	Rv3567c	-2.782597
ppm1	polyprenol-monophosphomannose synthase	protein_coding	NP_216567.1	Rv2051c	-2.784798
.	transmembrane protein	protein_coding	NP_217996.2	Rv3479	-2.784979
.	hypothetical protein	protein_coding	NP_214723.1	Rv0209	-2.785150
hisF	imidazole glycerol phosphate synthase subunit HisF	protein_coding	NP_216121.1	Rv1605	-2.787770
hemB	delta-aminolevulinic acid dehydratase	protein_coding	NP_215026.1	Rv0512	-2.794036
.	diacylglycerol O-acyltransferase	protein_coding	NP_215941.1	Rv1425	-2.803667
mbtM	long-chain-fatty-acid--ACP ligase MbtM	protein_coding	NP_215861.1	Rv1345	-2.815520
.	hypothetical protein	protein_coding	NP_217212.1	Rv2696c	-2.817636
echA4	enoyl-CoA hydratase EchA4	protein_coding	NP_215187.1	Rv0673	-2.830763
.	transmembrane protein	protein_coding	NP_215194.1	Rv0680c	-2.833323
lat	L-lysine-epsilon aminotransferase	protein_coding	NP_217807.1	Rv3290c	-2.834217
.	hypothetical protein	protein_coding	NP_215212.1	Rv0698	-2.837856
.	hypothetical protein	protein_coding	NP_215083.1	Rv0569	-2.837890
ispD	2-C-methyl-D-erythritol 4-phosphate cytidylyltransfera	protein_coding	NP_218099.1	Rv3582c	-2.840152
bglS	beta-glucosidase BglS	protein_coding	NP_214700.1	Rv0186	-2.842049
.	monooxygenase	protein_coding	NP_214899.1	Rv0385	-2.849317
lpdA	NAD(P)H quinone reductase LpdA	protein_coding	NP_217820.1	Rv3303c	-2.852169
rpsK	30S ribosomal protein S11	protein_coding	NP_217976.1	Rv3459c	-2.854283
.	GCN5-like N-acetyltransferase	protein_coding	NP_217383.1	Rv2867c	-2.854392
ilvA	threonine dehydratase IlvA	protein_coding	NP_216075.1	Rv1559	-2.859591
pknA	serine/threonine-protein kinase PknA	protein_coding	NP_214529.1	Rv0015c	-2.865279
.	hypothetical protein	protein_coding	NP_217179.1	Rv2663	-2.868857
.	universal stress protein	protein_coding	NP_216521.1	Rv2005c	-2.882036
oppB	oligopeptide ABC transporter permease OppB	protein_coding	NP_215799.1	Rv1283c	-2.883209
.	hypothetical protein	protein_coding	NP_218044.1	Rv3527	-2.885037
hpx	non-heme haloperoxidase Hpx	protein_coding	NP_217687.1	Rv3171c	-2.889797
.	haloalkane dehalogenase	protein_coding	NP_216812.1	Rv2296	-2.898626
.	transmembrane protein	protein_coding	NP_215391.1	Rv0876c	-2.911349
.	hypothetical protein	protein_coding	NP_215126.1	Rv0612	-2.914457
accD3	acetyl-CoAcarboxylase carboxyl transferase subunit be	protein_coding	NP_215419.1	Rv0904c	-2.916301
echA16	enoyl-CoA hydratase EchA16	protein_coding	NP_217347.1	Rv2831	-2.917642
.	hypothetical protein	protein_coding	NP_216989.1	Rv2473	-2.923538
.	transposase	protein_coding	NP_215437.1	Rv0922	-2.936228
deoC	2-deoxyribose-5-phosphate aldolase	protein_coding	NP_214992.1	Rv0478	-2.940291
.	hypothetical protein	protein_coding	YP_004837048.2	Rv0397A	-2.942052
nadB	L-aspartate oxidase	protein_coding	NP_216111.1	Rv1595	-2.942880
.	oxidoreductase	protein_coding	NP_214597.1	Rv0083	-2.949874
engA	GTPase Der	protein_coding	NP_216229.1	Rv1713	-2.962016
.	oxidoreductase	protein_coding	NP_218258.1	Rv3741c	-2.968677
.	hypothetical protein	protein_coding	NP_216048.1	Rv1532c	-2.968846
.	RNA polymerase-binding protein RbpA	protein_coding	NP_216566.1	Rv2050	-2.972975
.	hypothetical protein	protein_coding	NP_217038.1	Rv2522c	-2.978269
.	multidrug ABC transporter ATPase/permease	protein_coding	NP_214708.1	Rv0194	-2.999082
.	hypothetical protein	protein_coding	NP_216700.1	Rv2184c	-3.006733
groEL1	chaperonin GroEL	protein_coding	NP_217934.1	Rv3417c	-3.015010
.	transmembrane protein	protein_coding	NP_217133.1	Rv2617c	-3.018876
dnaJ2	chaperone protein DnaJ	protein_coding	NP_216889.1	Rv2373c	-3.021518
fadD11	fatty-acid--CoA ligase FadD11	protein_coding	NP_216066.1	Rv1550	-3.025412
.	transmembrane protein	protein_coding	NP_214553.1	Rv0039c	-3.034403
lipY	triacylglycerol lipase Lip	protein_coding	YP_177924.1	Rv3097c	-3.035379
.	hypothetical protein	protein_coding	NP_214656.1	Rv0142	-3.037678
.	short-chain type oxidoreductase	protein_coding	NP_214998.1	Rv0484c	-3.039468
obg	GTPase Obg	protein_coding	NP_216956.1	Rv2440c	-3.044922
.	hypothetical protein	protein_coding	NP_215276.1	Rv0762c	-3.060271
.	threonylcarbamoyl-AMP synthase	protein_coding	NP_215817.1	Rv1301	-3.065106
rbfA	ribosome-binding factor RbfA	protein_coding	NP_217354.1	Rv2838c	-3.065604
kshA	3-ketosteroid-9-alpha-monooxygenase oxygenase subu	protein_coding	NP_218043.1	Rv3526	-3.067829
dnaE2	error-prone DNA polymerase	protein_coding	NP_217887.3	Rv3370c	-3.068870
lipI	lipase	protein_coding	NP_215916.1	Rv1400c	-3.073418
leuA	2-isopropylmalate synthase	protein_coding	NP_218227.3	Rv3710	-3.077252
thiL	thiamine-monophosphate kinase	protein_coding	NP_217493.1	Rv2977c	-3.077462
.	insertion sequence element IS1533 transposase	protein_coding	NP_217459.1	Rv2943	-3.081190
.	membrane protein	protein_coding	NP_215746.1	Rv1230c	-3.087801
.	reductase	protein_coding	NP_216385.1	Rv1869c	-3.093655
.	mycofactocin radical SAM maturase MftC	protein_coding	NP_215207.1	Rv0693	-3.104999
.	type III pantothenate kinase	protein_coding	NP_218117.1	Rv3600c	-3.131657
.	multidrug-efflux transporter	protein_coding	NP_216150.1	Rv1634	-3.135693
etgB	iron(II)-dependent oxidoreductase EgtB	protein_coding	NP_218220.1	Rv3703c	-3.144153

hsdS	type I restriction/modification system specificity detern	protein_coding	NP_217277.1	Rv2761c	-3.144374
.	3-oxoacyl-ACP reductase	protein_coding	NP_218019.1	Rv3502c	-3.148334
.	hypothetical protein	protein_coding	NP_215392.1	Rv0877	-3.154878
.	hypothetical protein	protein_coding	NP_217146.1	Rv2630	-3.156665
ctpF	cation transporter ATPase F	protein_coding	NP_216513.1	Rv1997	-3.161397
phoH2	phosphate starvation-inducible protein PsiH	protein_coding	NP_215611.1	Rv1095	-3.164167
.	hypothetical protein	protein_coding	NP_217562.1	Rv3046c	-3.169735
.	ABC transporter ATP-binding protein	protein_coding	NP_217557.1	Rv3041c	-3.187691
.	bifunctional oligoribonuclease/PAP phosphatase NrnA	protein_coding	NP_217353.1	Rv2837c	-3.196689
.	hypothetical protein	protein_coding	NP_217871.1	Rv3354	-3.197904
nei	endonuclease VIII	protein_coding	NP_217814.1	Rv3297	-3.203064
fadE23	acyl-CoA dehydrogenase FadE23	protein_coding	NP_217656.1	Rv3140	-3.203568
ruvB	Holliday junction ATP-dependent DNA helicase RuvB	protein_coding	NP_217108.1	Rv2592c	-3.205631
.	oxidoreductase	protein_coding	NP_215089.1	Rv0575c	-3.206374
.	hypothetical protein	protein_coding	NP_216591.1	Rv2075c	-3.216906
mpt53	soluble secreted antigen Mpt53	protein_coding	NP_217394.1	Rv2878c	-3.223111
esxL	ESAT-6 like protein EsxL	protein_coding	NP_215714.1	Rv1198	-3.226926
rplR	50S ribosomal protein L18	protein_coding	NP_215234.1	Rv0720	-3.239563
PE_PGRS51	PE-PGRS family protein PE_PGRS51	protein_coding	YP_177965.1	Rv3367	-3.242231
fgd2	F420-dependent glucose-6-phosphate dehydrogenase	protein_coding	NP_214646.1	Rv0132c	-3.244224
sigJ	ECF RNA polymerase sigma factor SigJ	protein_coding	NP_217845.1	Rv3328c	-3.253190
nrdB	ribonucleoside-diphosphate reductase subunit beta Nrdl	protein_coding	NP_214747.1	Rv0233	-3.258852
.	AsnC family transcriptional regulator	protein_coding	NP_216840.1	Rv2324	-3.260314
.	RNA-splicing ligase RtcB	protein_coding	NP_217147.2	Rv2631	-3.268315
.	hypothetical protein	protein_coding	NP_216534.1	Rv2018	-3.272613
.	DNA polymerase	protein_coding	NP_218161.1	Rv3644c	-3.272913
.	alpha-(1->6)-mannopyranosyltransferase	protein_coding	NP_215975.1	Rv1459c	-3.279685
udgB	uracil DNA glycosylase	protein_coding	NP_215775.1	Rv1259	-3.289892
recX	regulatory protein RecX	protein_coding	NP_217252.1	Rv2736c	-3.297692
.	hypothetical protein	protein_coding	NP_214777.1	Rv0263c	-3.310449
hemN	oxygen-independent coproporphyrinogen III oxidase	protein_coding	NP_216904.1	Rv2388c	-3.315450
.	trans-acting enoyl reductase	protein_coding	NP_216965.1	Rv2449c	-3.322590
.	integral membrane transport protein	protein_coding	YP_177949.1	Rv3236c	-3.332681
trpD	anthranilate phosphoribosyltransferase	protein_coding	NP_216708.1	Rv2192c	-3.339428
.	hypothetical protein	protein_coding	NP_216248.1	Rv1732c	-3.343591
.	oxidoreductase	protein_coding	NP_214582.1	Rv0068	-3.350438
.	hypothetical protein	protein_coding	NP_215506.1	Rv0991c	-3.359964
erm(37)	23S rRNA (adenine(2058)-N(6))-methyltransferase Err	protein_coding	NP_216504.1	Rv1988	-3.362392
radA	DNA repair protein RadA	protein_coding	NP_218102.1	Rv3585	-3.393074
hemD	uroporphyrin-III C-methyltransferase	protein_coding	YP_177733.1	Rv0511	-3.400129
.	oxidase	protein_coding	NP_215361.1	Rv0846c	-3.403930
.	hypothetical protein	protein_coding	NP_216677.1	Rv2161c	-3.410369
arsC	arsenic-transport integral membrane protein ArsC	protein_coding	NP_217159.1	Rv2643	-3.413044
.	transcriptional regulator	protein_coding	NP_215683.1	Rv1167c	-3.413591
.	hypothetical protein	protein_coding	NP_217679.1	Rv3163c	-3.423258
.	resolvase	protein_coding	NP_215436.1	Rv0921	-3.428076
.	transcriptional regulator	protein_coding	NP_214999.1	Rv0485	-3.431750
.	diacyglycerol O-acyltransferase	protein_coding	NP_217000.1	Rv2484c	-3.442487
PPE19	PPE family protein PPE19	protein_coding	YP_177801.1	Rv1361c	-3.447189
fadE5	acyl-CoA dehydrogenase FadE5	protein_coding	NP_214758.1	Rv0244c	-3.455098
.	FAD-containing monooxygenase MymA	protein_coding	NP_217599.1	Rv3083	-3.459017
PE13	PE family protein PE13	protein_coding	YP_177794.1	Rv1195	-3.472258
mesT	epoxide hydrolase MesT	protein_coding	YP_177938.1	Rv3176c	-3.474418
.	integral membrane protein	protein_coding	NP_215055.1	Rv0541c	-3.496211
.	hypothetical protein	protein_coding	NP_215341.1	Rv0826	-3.503354
.	HTH-type transcriptional regulator	protein_coding	YP_177808.1	Rv1395	-3.507917
.	transcriptional regulator	protein_coding	NP_214816.1	Rv0302	-3.511620
.	hypothetical protein	protein_coding	NP_218189.1	Rv3672c	-3.515325
.	hypothetical protein	protein_coding	NP_217817.1	Rv3300c	-3.530750
adhD	alcohol dehydrogenase D	protein_coding	NP_217602.1	Rv3086	-3.537244
.	oxidoreductase	protein_coding	NP_216372.1	Rv1856c	-3.545225
cysW	sulfate ABC transporter permease CysW	protein_coding	NP_216914.1	Rv2398c	-3.563451
.	hypothetical protein	protein_coding	NP_215085.1	Rv0571c	-3.564123
mbtI	salicylate synthase	protein_coding	YP_177877.1	Rv2386c	-3.581105
.	hypothetical protein	protein_coding	NP_215226.1	Rv0712	-3.587331
cyp142	cytochrome P450 monooxygenase Cyp142	protein_coding	NP_218035.1	Rv3518c	-3.600089
.	phage protein	protein_coding	NP_216098.1	Rv1582c	-3.602165
.	glutamine amidotransferase	protein_coding	NP_217375.1	Rv2859c	-3.627136
.	phage protein	protein_coding	NP_216094.1	Rv1578c	-3.630665
echA5	enoyl-CoA hydratase EchA5	protein_coding	YP_177745.1	Rv0675	-3.632192
.	transmembrane protein	protein_coding	NP_217248.1	Rv2732c	-3.639175

irtB	iron ABC transporter ATP-binding protein/permease Ir	protein_coding	NP_215865.1	Rv1349	-3.639684
.	hypothetical protein	protein_coding	NP_218089.1	Rv3572	-3.643007
purN	phosphoribosylglycinamide formyltransferase PurN	protein_coding	NP_215471.1	Rv0956	-3.665014
.	hypothetical protein	protein_coding	NP_216414.1	Rv1898	-3.669017
acrA1	acyl-CoA-reductase AcrA	protein_coding	NP_217908.1	Rv3391	-3.669061
glnD	bifunctional uridylyltransferase/uridylyl-removing enzy	protein_coding	NP_217434.1	Rv2918c	-3.703914
panC	pantothenate synthetase	protein_coding	NP_218119.1	Rv3602c	-3.706842
frdA	fumarate reductase flavoprotein subunit	protein_coding	NP_216068.1	Rv1552	-3.708380
gcp	O-sialoglycoprotein endopeptidase	protein_coding	NP_217936.1	Rv3419c	-3.716219
pepR	zinc protease	protein_coding	NP_217298.1	Rv2782c	-3.718347
trxB2	thioredoxin reductase	protein_coding	NP_218430.1	Rv3913	-3.721527
echA12	enoyl-CoA hydratase EchA12	protein_coding	NP_215988.1	Rv1472	-3.726158
.	hypothetical protein	protein_coding	NP_216410.1	Rv1894c	-3.730736
infB	translation initiation factor IF-2	protein_coding	NP_217355.1	Rv2839c	-3.736027
fadE12	acyl-CoA dehydrogenase fadE12	protein_coding	NP_215487.1	Rv0972c	-3.738239
.	hypothetical protein	protein_coding	NP_216232.1	Rv1716	-3.741494
mmpL5	transmembrane transport protein MmpL5	protein_coding	NP_215190.1	Rv0676c	-3.752759
.	hypothetical protein	protein_coding	NP_216532.1	Rv2016	-3.769195
argD	acetylornithine aminotransferase	protein_coding	NP_216171.1	Rv1655	-3.792122
ggtB	gamma-glutamyltranspeptidase precursor GgtB	protein_coding	NP_216910.1	Rv2394	-3.819276
.	hypothetical protein	protein_coding	NP_218144.1	Rv3627c	-3.827300
.	hypothetical protein	protein_coding	NP_216189.1	Rv1673c	-3.838542
fadE13	acyl-CoA dehydrogenase FadE13	protein_coding	NP_215490.1	Rv0975c	-3.847538
.	hypothetical protein	protein_coding	NP_216527.1	Rv2011c	-3.850936
.	transcriptional regulator	protein_coding	NP_216050.1	Rv1534	-3.867609
.	diacylglycerol O-acyltransferase	protein_coding	NP_217603.1	Rv3087	-3.869687
glgA	capsular glucan synthase	protein_coding	NP_215728.1	Rv1212c	-3.902527
cydC	cytochrome biosyntheisis ABC transporter ATP-bindin	protein_coding	NP_216136.1	Rv1620c	-3.902663
.	HTH-type transcriptional regulator	protein_coding	NP_215803.1	Rv1287	-3.916028
.	NAD-dependent oxidoreductase	protein_coding	NP_215201.1	Rv0687	-3.923578
PPE60	PE family protein PPE60	protein_coding	YP_177976.1	Rv3478	-3.963221
.	ATP-dependent DNA helicase	protein_coding	NP_217718.1	Rv3202c	-3.980831
kmtR	HTH-type transcriptional regulator KmtR	protein_coding	NP_215342.1	Rv0827c	-3.997159
truB	tRNA pseudouridine synthase B	protein_coding	NP_217309.1	Rv2793c	-4.001590
.	hypothetical protein	protein_coding	NP_217163.2	Rv2647	-4.017595
mutA	methylmalonyl-CoA mutase small subunit	protein_coding	NP_216008.1	Rv1492	-4.023983
fadE34	acyl-CoA dehydrogenase FadE34	protein_coding	NP_218090.1	Rv3573c	-4.024341
.	monooxygenase	protein_coding	NP_218135.1	Rv3618	-4.029884
.	hypothetical protein	protein_coding	NP_218222.1	Rv3705c	-4.030377
xseA	exodeoxyribonuclease VII large subunit	protein_coding	NP_215624.1	Rv1108c	-4.037827
birA	bifunctional biotin operon repressor/biotin--[acetyl-Co/	protein_coding	NP_217796.1	Rv3279c	-4.041206
leuD	3-isopropylmalate dehydratase small subunit	protein_coding	NP_217503.1	Rv2987c	-4.059905
.	transmembrane protein	protein_coding	NP_215042.1	Rv0528	-4.060437
fadE26	acyl-CoA dehydrogenase FadE26	protein_coding	NP_218021.1	Rv3504	-4.071096
.	macrolide ABC transporter ATP-binding protein	protein_coding	NP_215989.1	Rv1473	-4.097222
.	hypothetical protein	protein_coding	NP_215142.1	Rv0628c	-4.112241
fadB3	3-hydroxybutyryl-CoA dehydrogenase FadB	protein_coding	YP_177829.1	Rv1715	-4.123384
cysH	phosphoadenosine phosphosulfate reductase	protein_coding	NP_216908.1	Rv2392	-4.124449
fusA2	elongation factor G	protein_coding	NP_214634.1	Rv0120c	-4.125926
.	oxidoreductase	protein_coding	NP_215773.1	Rv1257c	-4.128871
.	methyltransferase	protein_coding	NP_215736.1	Rv1220c	-4.132883
accD2	acetyl-/propionyl-CoA carboxylase subunit beta	protein_coding	NP_215489.1	Rv0974c	-4.137373
PPE56	PPE family protein PPE56	protein_coding	YP_177964.1	Rv3350c	-4.149489
.	transcriptional regulator	protein_coding	NP_214746.1	Rv0232	-4.160878
PE4	PE family protein PE4	protein_coding	YP_177698.1	Rv0160c	-4.194059
.	rRNA small subunit methyltransferase E	protein_coding	NP_216888.1	Rv2372c	-4.207654
fadE27	acyl-CoA dehydrogenase FadE27	protein_coding	NP_218022.1	Rv3505	-4.214670
.	hypothetical protein	protein_coding	NP_215400.1	Rv0885	-4.222578
eccD4	ESX-4 secretion system protein EccD4	protein_coding	NP_217965.1	Rv3448	-4.230215
blaR	sensor-transducer protein BlaR	protein_coding	NP_216361.1	Rv1845c	-4.238062
.	hypothetical protein	protein_coding	NP_214778.1	Rv0264c	-4.239992
pepD	serine protease PepD	protein_coding	NP_215498.1	Rv0983	-4.280261
moeB1	adenylyltransferase/sulfurtransferase MoeZ	protein_coding	YP_177942.1	Rv3206c	-4.281968
.	oxidoreductase SadH	protein_coding	NP_217601.1	Rv3085	-4.282402
ideR	iron-dependent repressor and activator IdeR	protein_coding	NP_217227.1	Rv2711	-4.292580
.	nonspecific lipid-transfer protein	protein_coding	NP_216143.1	Rv1627c	-4.308860
murE	UDP-N-acetylmuramoylalanyl-D-glutamate--2,6-diami	protein_coding	NP_216674.1	Rv2158c	-4.311242
.	membrane protein	protein_coding	NP_215917.1	Rv1401	-4.321827
csoR	copper-sensing transcriptional repressor CsoR	protein_coding	NP_215482.1	Rv0967	-4.322594
.	hypothetical protein	protein_coding	NP_216109.1	Rv1593c	-4.331014
.	hypothetical protein	protein_coding	NP_216033.1	Rv1517	-4.334664

echA19	enoyl-CoA hydratase EchA19	protein_coding	NP_218033.1	Rv3516	-4.336470
hflX	GTP-binding protein HflX	protein_coding	NP_217241.1	Rv2725c	-4.365563
.	monooxygenase	protein_coding	NP_217565.1	Rv3049c	-4.370825
fadA2	acetyl-CoA acetyltransferase FadA	protein_coding	NP_214757.1	Rv0243	-4.392862
.	hypothetical protein	protein_coding	NP_216241.1	Rv1725c	-4.396323
PPE55	PPE family protein PPE55	protein_coding	YP_177963.1	Rv3347c	-4.399698
.	oxidoreductase	protein_coding	YP_177756.1	Rv0794c	-4.401884
.	aldehyde dehydrogenase	protein_coding	NP_214972.1	Rv0458	-4.409273
rseA	anti-sigma E factor RseA	protein_coding	NP_215738.1	Rv1222	-4.411200
cyp123	cytochrome P450 Cyp123	protein_coding	NP_215280.1	Rv0766c	-4.430200
.	integral membrane protein	protein_coding	NP_217245.1	Rv2729c	-4.446774
sugI	sugar-transport integral membrane protein SugI	protein_coding	NP_217848.1	Rv3331	-4.452486
.	hypothetical protein	protein_coding	NP_217258.1	Rv2742c	-4.491939
tsnR	23S rRNA methyltransferase TsnR	protein_coding	NP_216160.1	Rv1644	-4.517804
.	hypothetical protein	protein_coding	NP_218164.1	Rv3647c	-4.519238
.	hypothetical protein	protein_coding	NP_215192.1	Rv0678	-4.522636
.	hypothetical protein	protein_coding	NP_218120.1	Rv3603c	-4.523383
.	hypothetical protein	protein_coding	NP_216144.1	Rv1628c	-4.559025
.	hypothetical protein	protein_coding	NP_216407.1	Rv1891	-4.561009
panB	3-methyl-2-oxobutanoate hydroxymethyltransferase	protein_coding	NP_216741.1	Rv2225	-4.567616
.	hypothetical protein	protein_coding	NP_216291.1	Rv1775	-4.616702
sirA	sulfite reductase	protein_coding	NP_216907.1	Rv2391	-4.632024
.	methyltransferase	protein_coding	NP_215919.1	Rv1403c	-4.643844
cydA	cytochrome D ubiquinol oxidase subunit I CydA	protein_coding	YP_177824.1	Rv1623c	-4.656526
moaA2	molybdenum cofactor biosynthesis protein MoaA	protein_coding	NP_215384.1	Rv0869c	-4.657293
.	hypothetical protein	protein_coding	NP_215354.1	Rv0839	-4.668804
.	hypothetical protein	protein_coding	NP_215390.1	Rv0875c	-4.674581
fic	cell filamentation protein Fic	protein_coding	NP_218158.1	Rv3641c	-4.705259
.	hypothetical protein	protein_coding	NP_216886.1	Rv2370c	-4.708815
.	integral membrane protein	protein_coding	NP_217852.1	Rv3335c	-4.727981
.	aldehyde dehydrogenase	protein_coding	NP_214737.1	Rv0223c	-4.752124
ligB	DNA ligase	protein_coding	NP_217578.1	Rv3062	-4.762888
.	hypothetical protein	protein_coding	NP_215564.1	Rv1048c	-4.816304
.	helicase	protein_coding	NP_218166.1	Rv3649	-4.879521
apt	adenine phosphoribosyltransferase	protein_coding	NP_217100.1	Rv2584c	-4.895411
.	hypothetical protein	protein_coding	NP_215102.1	Rv0588	-4.896692
.	aminoglycoside phosphotransferase	protein_coding	NP_217684.1	Rv3168	-4.900745
fadE24	acyl-CoA dehydrogenase	protein_coding	NP_217655.1	Rv3139	-4.908524
mce2A	Mce family protein Mce2A	protein_coding	YP_177740.1	Rv0589	-4.944788
.	integral membrane protein	protein_coding	NP_216770.1	Rv2254c	-4.965641
.	hypothetical protein	protein_coding	NP_217032.2	Rv2516c	-4.968470
B11	Putative small regulatory RNA	ncRNA	.	RVnc0004	-4.977597
.	TetR family transcriptional regulator	protein_coding	NP_217676.1	Rv3160c	-4.998236
smtB	HTH-type transcriptional regulator SmtB	protein_coding	NP_216874.1	Rv2358	-5.052503
fadE29	acyl-CoA dehydrogenase FadE29	protein_coding	NP_218060.1	Rv3543c	-5.066928
PE20	PE family protein PE20	protein_coding	YP_177843.1	Rv1806	-5.118267
leuC	3-isopropylmalate dehydratase large subunit	protein_coding	NP_217504.1	Rv2988c	-5.118749
fabG4	3-oxoacyl-ACP reductase FabG	protein_coding	NP_214756.1	Rv0242c	-5.150422
.	hypothetical protein	protein_coding	NP_216985.1	Rv2469c	-5.152680
rplV	50S ribosomal protein L22	protein_coding	NP_215220.1	Rv0706	-5.181857
aofH	flavin-containing monoamine oxidase	protein_coding	NP_217686.1	Rv3170	-5.183869
mce1R	transcriptional regulator Mce1R	protein_coding	YP_177700.2	Rv0165c	-5.211122
dfrA	dihydrofolate reductase	protein_coding	NP_217279.1	Rv2763c	-5.216891
fxsA	transmembrane protein FxsA	protein_coding	NP_216569.1	Rv2053c	-5.220870
.	hypothetical protein	protein_coding	NP_218059.1	Rv3542c	-5.259212
.	aminotransferase	protein_coding	NP_215694.1	Rv1178	-5.285723
.	tRNA-dihydrouridine synthase	protein_coding	NP_215338.1	Rv0823c	-5.305218
mce2R	HTH-type transcriptional regulator Mce2R	protein_coding	NP_215100.1	Rv0586	-5.331961
hemL	glutamate-1-semialdehyde 2,1-aminomutase	protein_coding	NP_215038.1	Rv0524	-5.357642
prfA	peptide chain release factor PrfA	protein_coding	NP_215815.1	Rv1299	-5.377463
.	universal stress protein	protein_coding	NP_217650.1	Rv3134c	-5.438706
lpqS	lipoprotein LpqS	protein_coding	NP_215362.1	Rv0847	-5.449062
scoA	succinyl-CoA:3-ketoacid-CoA transferase subunit A	protein_coding	NP_217020.1	Rv2504c	-5.452464
.	hypothetical protein	protein_coding	NP_216984.1	Rv2468c	-5.467359
rrf	5S ribosomal RNA	rRNA	.	Rvnr03	-5.495215
.	hypothetical protein	protein_coding	NP_217685.1	Rv3169	-5.508849
che1	ferrochelatase	protein_coding	NP_216909.1	Rv2393	-5.510714
.	hypothetical protein	protein_coding	NP_216520.1	Rv2004c	-5.526795
clpC2	ATP-dependent protease ATP-binding subunit ClpC	protein_coding	YP_177897.1	Rv2667	-5.580373
mshA	D-inositol 3-phosphate glycosyltransferase	protein_coding	NP_215000.1	Rv0486	-5.606417
.	hypothetical protein	protein_coding	NP_216551.1	Rv2035	-5.612688

esxK	ESAT-6 like protein EsxK	protein_coding	NP_215713.1	Rv1197	-5.657114
.	hypothetical protein	protein_coding	NP_215304.1	Rv0789c	-5.683448
.	AraC family transcriptional regulator	protein_coding	NP_218350.1	Rv3833	-5.846748
.	transcriptional regulator	protein_coding	NP_215976.2	Rv1460	-5.869856
.	TetR/Acr family transcriptional regulator	protein_coding	NP_217689.1	Rv3173c	-5.877537
PPE20	PPE family protein PPE20	protein_coding	YP_177806.1	Rv1387	-5.892315
dnaK	chaperone protein DnaK	protein_coding	NP_214864.1	Rv0350	-5.958602
.	stearoyl-CoA 9-desaturase electron transfer protein	protein_coding	NP_217747.1	Rv3230c	-5.993920
.	MerR family transcriptional regulator	protein_coding	NP_217851.1	Rv3334	-6.039822
.	dehydrogenase	protein_coding	YP_177986.1	Rv3538	-6.111655
.	hypothetical protein	protein_coding	NP_215600.1	Rv1084	-6.127358
PE_PGRS15	PE-PGRS family protein PE_PGRS15	protein_coding	YP_177763.1	Rv0872c	-6.128130
higA	antitoxin HigA	protein_coding	NP_216472.1	Rv1956	-6.168735
.	hypothetical protein	protein_coding	NP_218003.1	Rv3486	-6.178880
.	integral membrane protein	protein_coding	NP_217209.1	Rv2693c	-6.183252
PE_PGRS33	PE-PGRS family protein PE_PGRS33	protein_coding	YP_177846.1	Rv1818c	-6.222133
aroF	chorismate synthase	protein_coding	NP_217056.1	Rv2540c	-6.224845
cysK2	cysteine synthase CysK	protein_coding	YP_177762.1	Rv0848	-6.260106
alkA	bifunctional regulatory protein/DNA repair enzyme All	protein_coding	NP_215833.1	Rv1317c	-6.286131
cyp130	cytochrome P450 Cyp130	protein_coding	NP_215772.1	Rv1256c	-6.373051
cyp125	steroid C26-monooxygenase	protein_coding	NP_218062.1	Rv3545c	-6.487102
hrp1	hypoxic response protein	protein_coding	NP_217142.1	Rv2626c	-6.519811
.	hypothetical protein	protein_coding	NP_214837.1	Rv0323c	-6.603925
pip	proline iminopeptidase	protein_coding	NP_215355.1	Rv0840c	-6.620491
.	monooxygenase	protein_coding	NP_216049.1	Rv1533	-6.745155
.	hypothetical protein	protein_coding	NP_217259.1	Rv2743c	-6.752746
clgR	transcriptional regulator ClgR	protein_coding	NP_217261.1	Rv2745c	-6.801079
pfkB	6-phosphofructokinase PfkB	protein_coding	NP_216545.1	Rv2029c	-6.822594
.	hypothetical protein	protein_coding	NP_216283.1	Rv1767	-6.832132
.	hypothetical protein	protein_coding	NP_217144.1	Rv2628	-6.857446
.	integral membrane protein	protein_coding	YP_177912.1	Rv2877c	-6.942598
cyp51	lanosterol 14-alpha demethylase	protein_coding	NP_215278.1	Rv0764c	-6.953062
fadE22	acyl-CoA dehydrogenase FadE22	protein_coding	NP_217577.1	Rv3061c	-6.957562
hsaF	4-hydroxy-2-oxovalerate aldolase	protein_coding	NP_218051.1	Rv3534c	-6.987645
.	hypothetical protein	protein_coding	NP_216528.1	Rv2012	-7.021934
.	multidrug-efflux transporter	protein_coding	NP_215774.1	Rv1258c	-7.035039
yrbE2A	hypothetical protein	protein_coding	NP_215101.1	Rv0587	-7.130373
.	Holliday junction resolvase	protein_coding	NP_217070.1	Rv2554c	-7.168063
.	universal stress protein	protein_coding	NP_216512.1	Rv1996	-7.247149
hsaC	extradiol dioxygenase	protein_coding	NP_218085.1	Rv3568c	-7.277029
.	hypothetical protein	protein_coding	NP_217643.1	Rv3127	-7.295846
.	methyltransferase	protein_coding	NP_215921.1	Rv1405c	-7.329341
.	hypothetical protein	protein_coding	NP_217031.1	Rv2515c	-7.338362
.	hypothetical protein	protein_coding	NP_214654.1	Rv0140	-7.349317
kstR	HTH-type transcriptional regulator KstR	protein_coding	NP_218091.3	Rv3574	-7.422297
.	prophage integrase	protein_coding	NP_217175.1	Rv2659c	-7.494636
.	oxidoreductase	protein_coding	NP_218047.1	Rv3530c	-7.495741
fadE28	acyl-CoA dehydrogenase FadE28	protein_coding	NP_218061.1	Rv3544c	-7.538321
hisD	histidinol dehydrogenase	protein_coding	NP_216115.1	Rv1599	-7.550061
.	hypothetical protein	protein_coding	NP_217610.1	Rv3094c	-7.638860
.	dioxygenase	protein_coding	NP_217677.1	Rv3161c	-7.718994
.	hypothetical protein	protein_coding	NP_216675.1	Rv2159c	-7.809125
ethA	monooxygenase EthA	protein_coding	NP_218371.1	Rv3854c	-7.913730
ctpV	copper-exporting ATPase	protein_coding	NP_215484.3	Rv0969	-7.955817
cysA1	sulfate ABC transporter ATP-binding protein CysA	protein_coding	YP_177879.1	Rv2397c	-7.989853
fadD19	acyl-CoA synthetase	protein_coding	YP_177983.1	Rv3515c	-8.049105
mutT3	8-oxo-dGTP diphosphatase	protein_coding	NP_214927.1	Rv0413	-8.056401
trxB1	thioredoxin	protein_coding	YP_177815.1	Rv1471	-8.063042
.	transcriptional regulator	protein_coding	NP_216533.1	Rv2017	-8.343943
fadE9	acyl-CoA dehydrogenase FadE9	protein_coding	NP_215266.1	Rv0752c	-8.457746
.	transcriptional regulator	protein_coding	NP_214842.1	Rv0328	-8.472990
kshB	3-ketosteroid-9-alpha-hydroxylase reductase subunit	protein_coding	NP_218088.1	Rv3571	-8.493903
.	zinc-binding alcohol dehydrogenase	protein_coding	NP_216411.1	Rv1895	-8.654822
mmsB	3-hydroxyisobutyrate dehydrogenase	protein_coding	NP_215265.1	Rv0751c	-8.820668
MTS1338	Putative small regulatory RNA	ncRNA	.	RVnc0036	-8.871165
.	hypothetical protein	protein_coding	NP_218046.1	Rv3529c	-8.876853
.	HTH-type transcriptional regulator	protein_coding	NP_215771.1	Rv1255c	-8.937804
.	TetR family transcriptional regulator	protein_coding	NP_217766.1	Rv3249c	-9.029208
fadA5	acetyl-CoA acetyltransferase FadA	protein_coding	NP_218063.1	Rv3546	-9.133523
kstD	3-oxosteroid 1-dehydrogenase	protein_coding	NP_218054.1	Rv3537	-9.159579
.	adenylyl-sulfate kinase	protein_coding	NP_215802.1	Rv1286	-9.211308

.	methyltransferase	protein_coding	NP_217859.1	Rv3342	-9.225817
narK2	nitrate/nitrite transporter	protein_coding	NP_216253.1	Rv1737c	-9.279788
sigE	ECF RNA polymerase sigma factor SigE	protein_coding	NP_215737.1	Rv1221	-9.415452
.	oxidoreductase	protein_coding	NP_216290.1	Rv1774	-9.512317
dnaJ1	chaperone protein DnaJ	protein_coding	YP_177719.1	Rv0352	-9.607662
lipX	lipase LipX	protein_coding	YP_177792.1	Rv1169c	-9.762349
.	dehydrogenase/reductase	protein_coding	NP_214845.1	Rv0331	-9.808081
.	short-chain type dehydrogenase/reductase	protein_coding	NP_215761.1	Rv1245c	-9.927697
nusB	N utilization substance protein B	protein_coding	NP_217049.1	Rv2533c	-10.065032
.	hypothetical protein	protein_coding	NP_215804.1	Rv1288	-10.145104
hsaE	hydratase	protein_coding	NP_218053.1	Rv3536c	-10.201059
.	transcriptional regulator	protein_coding	NP_215167.1	Rv0653c	-10.210821
hsaA	flavin-dependent monooxygenase oxygenase subunit H	protein_coding	NP_218087.1	Rv3570c	-10.322917
.	hypothetical protein	protein_coding	NP_217570.1	Rv3054c	-10.495542
grpE	stress response protein GrpE	protein_coding	NP_214865.1	Rv0351	-10.615788
PPE18	PPE family protein PPE18	protein_coding	YP_177795.1	Rv1196	-10.709284
.	hypothetical protein	protein_coding	NP_216254.1	Rv1738	-10.848301
.	transcriptional regulator	protein_coding	NP_216190.1	Rv1674c	-11.077935
.	hypothetical protein	protein_coding	NP_216552.1	Rv2036	-11.155129
.	hypothetical protein	protein_coding	NP_217143.1	Rv2627c	-11.226317
.	hypothetical protein	protein_coding	NP_217738.1	Rv3222c	-11.242645
rpiB	ribose-5-phosphate isomerase B	protein_coding	YP_177884.1	Rv2465c	-11.503055
whiB7	transcriptional regulator WhiB7	protein_coding	YP_177940.1	Rv3197A	-11.597078
hsaD	4,5-9,10-diseco-3-hydroxy-5,9,17-trioxoandrosta-1(10)	protein_coding	NP_218086.1	Rv3569c	-11.769131
PE_PGRS11	PE-PGRS family protein PE_PGRS11	protein_coding	YP_177752.1	Rv0754	-12.006339
.	dehydrogenase/reductase	protein_coding	NP_214817.1	Rv0303	-12.171236
TB31.7	universal stress protein	protein_coding	NP_217139.1	Rv2623	-12.495084
.	hypothetical protein	protein_coding	NP_216568.1	Rv2052c	-12.628277
.	transcriptional regulator	protein_coding	NP_217137.1	Rv2621c	-12.811087
.	oxidoreductase	protein_coding	NP_215284.1	Rv0770	-12.863793
fdxA	ferredoxin	protein_coding	NP_216523.1	Rv2007c	-12.913183
.	cation efflux system protein	protein_coding	NP_216541.1	Rv2025c	-12.990309
.	hypothetical protein	protein_coding	NP_216391.1	Rv1875	-13.115227
ethR	HTH-type transcriptional repressor EthR	protein_coding	NP_218372.1	Rv3855	-13.291907
mymT	metallothionein	protein_coding	YP_004837046.2	Rv0186A	-14.333487
cydD	cytochrome biosyntheisis ABC transporter ATP-bindin	protein_coding	NP_216137.1	Rv1621c	-14.947216
higB	toxin HigB	protein_coding	NP_216471.2	Rv1955	-15.675594
.	hypothetical protein	protein_coding	NP_217980.1	Rv3463	-16.148472
.	oxidoreductase	protein_coding	NP_215283.1	Rv0769	-16.545721
.	hypothetical protein	protein_coding	NP_218048.1	Rv3531c	-17.145137
.	dioxygenase	protein_coding	NP_217923.1	Rv3406	-17.350127
.	phage integrase	protein_coding	NP_216102.1	Rv1586c	-17.529553
.	HTH-type transcriptional regulator	protein_coding	NP_215281.1	Rv0767c	-17.723503
clpB	chaperone protein ClpB	protein_coding	NP_214898.1	Rv0384c	-18.014201
PPE17	PPE family protein PPE17	protein_coding	YP_177791.1	Rv1168c	-18.368344
tgs1	diacylglycerol O-acyltransferase	protein_coding	NP_217646.1	Rv3130c	-18.548621
.	hypothetical protein	protein_coding	NP_216546.1	Rv2030c	-18.857681
.	hypothetical protein	protein_coding	NP_218177.1	Rv3660c	-19.303645
mcr11	Putative small regulatory RNA	ncRNA	.	RVnc0013	-20.287756
cyp135A1	cytochrome P450 Cyp135A1	protein_coding	NP_214841.1	Rv0327c	-21.091900
.	oxidoreductase	protein_coding	NP_215279.1	Rv0765c	-21.739114
.	hypothetical protein	protein_coding	NP_214593.1	Rv0079	-22.364957
mmsA	methylmalonate-semialdehyde dehydrogenase	protein_coding	NP_215267.1	Rv0753c	-24.719979
hsaG	acetaldehyde dehydrogenase	protein_coding	NP_218052.1	Rv3535c	-24.738704
cysD	sulfate adenylyltransferase subunit 2	protein_coding	NP_215801.1	Rv1285	-25.684416
.	hypothetical protein	protein_coding	NP_216982.1	Rv2466c	-25.913469
.	NAD(P)H nitroreductase	protein_coding	NP_217647.3	Rv3131	-26.159731
ctpC	manganese/zinc-exporting P-type ATPase	protein_coding	NP_217787.1	Rv3270	-27.597964
aldA	aldehyde dehydrogenase AldA	protein_coding	NP_215282.1	Rv0768	-29.407597
hsp	heat shock protein	protein_coding	NP_214765.1	Rv0251c	-30.517574
.	hypothetical protein	protein_coding	NP_217786.1	Rv3269	-30.751262
acg	NAD(P)H nitroreductase	protein_coding	NP_216548.1	Rv2032	-35.847298
narX	nitrate reductase-like protein NarX	protein_coding	NP_216252.1	Rv1736c	-37.846421
.	hypothetical protein	protein_coding	NP_215352.1	Rv0837c	-45.070827
.	hypothetical protein	protein_coding	NP_216329.1	Rv1813c	-45.827441
.	TetR family HTH-type transcriptional regulator	protein_coding	NP_217428.1	Rv2912c	-47.607882
.	hypothetical protein	protein_coding	NP_215305.1	Rv0790c	-73.891963
alkB	transmembrane alkane 1-monooxygenase AlkB	protein_coding	NP_217769.1	Rv3252c	-76.325032
.	carotenoid cleavage oxygenase	protein_coding	NP_215168.1	Rv0654	-76.437277
.	hypothetical protein	protein_coding	NP_215306.1	Rv0791c	-82.189090
.	transcriptional regulator	protein_coding	NP_215307.1	Rv0792c	-92.322196

.	D-amino acid aminohydrolase	protein_coding	NP_217429.1	Rv2913c	-135.319852
