# Supplementary material for: Mycobacterium tuberculosis bacillus induces pyroptosis in human lung fibroblasts
Source: mSphere. 2025 May 19;10(6):e00110-25. doi: 10.1128/msphere.00110-25 (PMC12188705; doi:10.1128/msphere.00110-25)
Supplement: Table S2 — DEG in MRC-5 fibroblasts infected vs uninfected with Mycobacterium tuberculosis. [file msphere.00110-25-s0007.pdf]

Supplemental table 2.The differentially expressed genes in MRC-5 fibroblasts infected vs uninfected with *Mycobacterium tuberculosis* H37Rv by RNA-Seq analysis

Gene_Symbol	Description	gene_biotype	Protein_ID	infected/un-infected MRC5, fc.
NEAT1	nuclear paraspeckle assembly transcript 1	lncRNA	.	144.3
ESM1	endothelial cell specific molecule 1	protein_coding	NP_001129076.1;NP_008967.1	130.9
CXCL8	C-X-C motif chemokine ligand 8	protein_coding	NP_000575.1;NP_001341769.1	98.4
LOC105369370	uncharacterized LOC105369370	lncRNA	.	64.5
IFI44L	interferon induced protein 44 like	protein_coding	NP_001362575.1;NP_001362576.1;	62.8
LOC107987083	uncharacterized LOC107987083, transcript varian	lncRNA	.	48.6
IL1A	interleukin 1 alpha	protein_coding	NP_000566.3;NP_001358483.1	46.3
MX2	MX dynamin like GTPase 2	protein_coding	NP_002454.1;XP_005261040.1;XP_	45.1
HMGB1P3	high mobility group box 1 pseudogene 3	pseudogene	.	37.7
LOC105375914	uncharacterized LOC105375914, transcript varian	lncRNA	.	34.5
VTA1P2	vesicle trafficking 1 pseudogene 2	pseudogene	.	34.0
IL1B	interleukin 1 beta	protein_coding	NP_000567.1;XP_016859477.1	33.9
MKRN5P	makorin ring finger protein 5, pseudogene	pseudogene	.	30.8
OR6A2	olfactory receptor family 6 subfamily A member 2	protein_coding	NP_003687.2	30.8
HAS2	hyaluronan synthase 2	protein_coding	NP_005319.1	30.7
LOC105375247	uncharacterized LOC105375247	lncRNA	.	29.2
RPL36AP42	ribosomal protein L36a pseudogene 42	pseudogene	.	28.2
OR2AG2	olfactory receptor family 2 subfamily AG member	protein_coding	NP_001004490.1	27.1
LOC105374179	uncharacterized LOC105374179	lncRNA	.	27.0
LOC100129577	mitochondrial carrier 1 pseudogene	pseudogene	.	26.7
ACTBP7	ACTB pseudogene 7	pseudogene	.	26.6
NUDCP1	nuclear distribution C pseudogene 1	pseudogene	.	26.1
MIR8058	microRNA 8058	miRNA	.	25.8
RPL7AP42	ribosomal protein L7a pseudogene 42	pseudogene	.	25.7
LOC105375310	uncharacterized LOC105375310	lncRNA	.	25.3
YTHDF1P1	YTH domain family member 1 pseudogene 1	pseudogene	.	24.7
PTGS2	prostaglandin-endoperoxide synthase 2	protein_coding	NP_000954.1	24.3
CXCL3	C-X-C motif chemokine ligand 3	protein_coding	NP_002081.2	24.2
LOC101928446	uncharacterized LOC101928446	lncRNA	.	23.2
LOC390618	ribosomal protein L7 like 1 pseudogene	pseudogene	.	23.0
LOC112268323	uncharacterized LOC112268323	lncRNA	.	22.6
RPS15AP6	ribosomal protein S15a pseudogene 6	pseudogene	.	22.2
OAS1	2'-5'-oligoadenylate synthetase 1	protein_coding	NP_001027581.1;NP_001307080.1;	22.1
LOC105374433	uncharacterized LOC105374433, transcript varian	lncRNA	.	22.0
ASDURF	ASNSD1 upstream reading frame	protein_coding	NP_001340422.1;NP_001340423.1	21.9
LOC107984089	uncharacterized LOC107984089, transcript varian	lncRNA	.	21.8
LOC112268421	uncharacterized LOC112268421	lncRNA	.	21.8
LOC107985686	uncharacterized LOC107985686	lncRNA	.	21.7
GAPDHP71	glyceraldehyde-3-phosphate dehydrogenase pseud	pseudogene	.	21.5
OAS2	2'-5'-oligoadenylate synthetase 2	protein_coding	NP_001027903.1;NP_002526.2;NP_	21.4
RN7SL630P	RNA, 7SL, cytoplasmic 630, pseudogene	pseudogene	.	21.3
ADAM20	ADAM metallopeptidase domain 20	protein_coding	NP_003805.4;XP_005268208.1	21.3
LOC107986930	uncharacterized LOC107986930, transcript varian	lncRNA	.	21.2
LOC105374584	uncharacterized LOC105374584	lncRNA	.	20.9
LOC105378030	uncharacterized LOC105378030	lncRNA	.	20.8
LOC107984120	uncharacterized LOC107984120	lncRNA	.	20.6
RFX8	RFX family member 8, lacking RFX DNA binding	protein_coding	NP_001139136.2;NP_001354437.1;	19.9
LOC107986116	uncharacterized LOC107986116	lncRNA	.	19.9
CXCL2	C-X-C motif chemokine ligand 2	protein_coding	NP_002080.1	19.8
LOC105377291	uncharacterized LOC105377291, transcript varian	lncRNA	.	19.6
ZMIZ1-AS1	ZMIZ1 antisense RNA 1	lncRNA	.	19.4
LOC145474	uncharacterized LOC145474	lncRNA	.	19.3
PSG8-AS1	PSG8 antisense RNA 1	lncRNA	.	19.3
LOC105376591	uncharacterized LOC105376591, transcript varian	lncRNA	.	19.2
RPL35P6	ribosomal protein L35 pseudogene 6	pseudogene	.	19.2
CXCL1	C-X-C motif chemokine ligand 1	protein_coding	NP_001502.1	18.7
LOC107984275	uncharacterized LOC107984275	lncRNA	.	18.6
RPSAP29	ribosomal protein SA pseudogene 29	pseudogene	.	18.5
LOC112268455	uncharacterized LOC112268455, transcript varian	lncRNA	.	18.3
LOC107985939	uncharacterized LOC107985939	lncRNA	.	18.2
LOC105376991	uncharacterized LOC105376991	lncRNA	.	17.9
LOC107985684	uncharacterized LOC107985684, transcript varian	lncRNA	.	17.7
KCNIP4-IT1	KCNIP4 intronic transcript 1	lncRNA	.	17.5
LOC105369313	uncharacterized LOC105369313	lncRNA	.	17.4
PLCZ1	phospholipase C zeta 1	protein_coding	NP_001317698.1;NP_001317703.1;	17.3
LOC107984016	uncharacterized LOC107984016	lncRNA	.	17.3
LOC105378983	uncharacterized LOC105378983	lncRNA	.	17.3

LINC00310	long intergenic non-protein coding RNA 310	lncRNA	.	17.3
LOC107985599	uncharacterized LOC107985599	lncRNA	.	17.2
LOC646870	centrosomal protein 57kDa pseudogene	pseudogene	.	17.2
IL24	interleukin 24	protein_coding	NP_001172085.1;NP_001172086.1;	17.2
LOC107985363	uncharacterized LOC107985363	lncRNA	.	17.0
PPP4R4	protein phosphatase 4 regulatory subunit 4	protein_coding	NP_001335071.1;NP_001335072.1;	17.0
SAA2	serum amyloid A2	protein_coding	NP_001120852.1;NP_110381.2	16.9
DDIT3	DNA damage inducible transcript 3	protein_coding	NP_001181982.1;NP_001181983.1;	16.9
RANP4	RAN pseudogene 4	pseudogene	.	16.9
LOC102723409	uncharacterized LOC102723409, transcript varian	lncRNA	.	16.4
GPR52	G protein-coupled receptor 52	protein_coding	NP_005675.3	16.4
SNORD168	small nucleolar RNA, C/D box 168	snoRNA	.	16.4
LOC100420738	chromosome 9 open reading frame 78 pseudogene	pseudogene	.	16.3
BACH1-IT3	BACH1 intronic transcript 3	lncRNA	.	16.3
LOC728715	ovostatin homolog 2	lncRNA	.	16.2
PRO1804	uncharacterized LOC100133319	lncRNA	.	16.2
DPPA4P1	developmental pluripotency associated 4 pseudoge	pseudogene	.	16.2
LOC105371271	uncharacterized LOC105371271	lncRNA	.	16.2
LOC105372663	uncharacterized LOC105372663	lncRNA	.	16.0
TSNAX-DISC1	TSNAX-DISC1 readthrough (NMD candidate)	lncRNA	.	15.8
POU5F1P5	POU class 5 homeobox 1 pseudogene 5	ranscribed_pseudogen	.	15.7
LOC105371618	uncharacterized LOC105371618	lncRNA	.	15.6
LOC105369312	uncharacterized LOC105369312	lncRNA	.	15.4
LINC02742	long intergenic non-protein coding RNA 2742, tra	lncRNA	.	15.4
XIAPP3	X-linked inhibitor of apoptosis pseudogene 3	pseudogene	.	15.3
SLC2A3P2	solute carrier family 2 member 3 pseudogene 2	pseudogene	.	15.3
LOC112268110	uncharacterized LOC112268110	lncRNA	.	15.2
RNF152P1	ring finger protein 152 pseudogene 1	pseudogene	.	15.2
PPIAP61	peptidylprolyl isomerase A pseudogene 61	pseudogene	.	15.2
IL37	interleukin 37	protein_coding	NP_055254.2;NP_775294.1;NP_77	14.9
TNFSF14	TNF superfamily member 14	protein_coding	NP_001363816.1;NP_003798.2;NP_	14.8
LOC107987109	uncharacterized LOC107987109	lncRNA	.	14.7
TPTEP2-CSNK1E	TPTEP2-CSNK1E readthrough	protein_coding	NP_001276841.1	14.6
MIR421	microRNA 421	miRNA	.	14.6
LOC107987172	uncharacterized LOC107987172, transcript varian	lncRNA	.	14.5
STC1	stanniocalcin 1	protein_coding	NP_003146.1	14.5
LOC107986135	uncharacterized LOC107986135	lncRNA	.	14.3
LOC107984749	uncharacterized LOC107984749, transcript varian	lncRNA	.	14.3
STX18-IT1	STX18 intronic transcript 1	lncRNA	.	14.2
ASAP1-IT1	ASAP1 intronic transcript 1	lncRNA	.	14.2
TAS2R3	taste 2 receptor member 3	protein_coding	NP_058639.1	14.1
FAM155A-IT1	FAM155A intronic transcript 1	lncRNA	.	14.0
LOC107985783	uncharacterized LOC107985783, transcript varian	lncRNA	.	14.0
LOC102724070	.	pseudogene	.	14.0
IL6	interleukin 6	protein_coding	NP_000591.1;NP_001305024.1;NP_	14.0
LOC107984434	.	pseudogene	.	14.0
LOC105369949	uncharacterized LOC105369949, transcript varian	lncRNA	.	13.9
LOC107987052	uncharacterized LOC107987052	lncRNA	.	13.9
TAS2R30	taste 2 receptor member 30	protein_coding	NP_001091112.1	13.8
RPSAP6	ribosomal protein SA pseudogene 6	pseudogene	.	13.8
KCCAT198	renal clear cell carcinoma-associated transcript 19	lncRNA	.	13.6
LOC100422382	nocturnin pseudogene	pseudogene	.	13.5
PPP6CP	protein phosphatase 6 catalytic subunit pseudogen	pseudogene	.	13.5
LOC112268015	uncharacterized LOC112268015, transcript varian	misc_RNA	.	13.4
SNORA84	small nucleolar RNA, H/ACA box 84	snoRNA	.	13.3
LINC00294	long intergenic non-protein coding RNA 294	lncRNA	.	13.2
LOC105372768	uncharacterized LOC105372768, transcript varian	lncRNA	.	13.2
PPIEL	peptidylprolyl isomerase E like pseudogene	ranscribed_pseudogen	.	13.1
HMGB1P24	high mobility group box 1 pseudogene 24	pseudogene	.	13.1
RPL21P44	ribosomal protein L21 pseudogene 44	ranscribed_pseudogen	.	13.0
TPT1P6	tumor protein, translationally-controlled 1 pseudog	pseudogene	.	13.0
LOC105376025	uncharacterized LOC105376025, transcript varian	lncRNA	.	12.9
LOC390586	hydroxyacyl-CoA dehydrogenase pseudogene	pseudogene	.	12.8
LOC107986008	uncharacterized LOC107986008	lncRNA	.	12.8
MIR221	microRNA 221	miRNA	.	12.7
MIR29A	microRNA 29a	miRNA	.	12.7
LOC107987108	uncharacterized LOC107987108, transcript varian	lncRNA	.	12.6
LOC100996664	uncharacterized LOC100996664	lncRNA	.	12.6
TAS2R63P	taste 2 receptor member 63 pseudogene	pseudogene	.	12.6
LOC105374010	uncharacterized LOC105374010, transcript varian	lncRNA	.	12.6
LOC101928389	uncharacterized LOC101928389	lncRNA	.	12.6

LOC105375721	uncharacterized LOC105375721, transcript varian	lncRNA	.	12.5
DIRC3	disrupted in renal carcinoma 3	lncRNA	.	12.5
LNCRNA-ATB	long noncoding RNA activated by TGF-beta	lncRNA	.	12.4
LOC107984616	uncharacterized LOC107984616	lncRNA	.	12.3
TMEM156	transmembrane protein 156	protein_coding	NP_001290157.1;NP_079219.1;XP_	12.3
LOC100421468	ubiquitin like modifier activating enzyme 2 pseudo	pseudogene	.	12.2
LOC112268272	uncharacterized LOC112268272, transcript varian	lncRNA	.	12.2
LOC107985486	uncharacterized LOC107985486, transcript varian	lncRNA	.	12.2
LOC105379752	putative uncharacterized protein FLJ46204	protein_coding	XP_016868426.1	12.1
GVINP1	GTPase, very large interferon inducible pseudoger	ranscribed_pseudogen	.	12.0
LOC107986820	uncharacterized LOC107986820, transcript varian	lncRNA	.	12.0
ADCY8	adenylate cyclase 8	protein_coding	NP_001106.1;XP_005250826.1;XP_	12.0
LOC107986178	uncharacterized LOC107986178, transcript varian	lncRNA	.	11.9
LOC105374786	uncharacterized LOC105374786, transcript varian	lncRNA	.	11.9
CRTC1P1	CRTC1 pseudogene 1	pseudogene	.	11.9
AKR1B1P1	aldehyde reductase family 1 member B1 pseudoge	pseudogene	.	11.9
LOC105374167	uncharacterized LOC105374167, transcript varian	lncRNA	.	11.8
NDUFA9P1	NADH:ubiquinone oxidoreductase subunit A9 pse	pseudogene	.	11.8
ADTRP	androgen dependent TFPI regulating protein	protein_coding	NP_001137420.1;NP_116133.1;XP_	11.8
LOC100420214	rhomboid domain containing 1 pseudogene	pseudogene	.	11.8
LOC107986424	uncharacterized LOC107986424	lncRNA	.	11.8
LOC105378228	uncharacterized LOC105378228	lncRNA	.	11.7
LINC-PINT	long intergenic non-protein coding RNA, p53 indu	lncRNA	.	11.7
LOC105372621	uncharacterized LOC105372621	lncRNA	.	11.7
IFNE	interferon epsilon	protein_coding	NP_795372.1	11.6
ARL4AP5	.	pseudogene	.	11.6
LOC105369559	uncharacterized LOC105369559, transcript varian	lncRNA	.	11.5
C3orf35	chromosome 3 open reading frame 35	protein_coding	NP_001289760.1;NP_001289761.1;	11.5
ERC2-IT1	ERC2 intronic transcript 1	lncRNA	.	11.5
HNRNPH1P1	heterogeneous nuclear ribonucleoprotein H1 pseuc	pseudogene	.	11.5
SOX5	SRY-box transcription factor 5	protein_coding	NP_001248343.1;NP_001248344.1;	11.5
SLC7A14-AS1	SLC7A14 antisense RNA 1	lncRNA	.	11.4
MIR1206	microRNA 1206	miRNA	.	11.4
LOC107986485	uncharacterized LOC107986485	lncRNA	.	11.4
SLIT2-IT1	SLIT2 intronic transcript 1	lncRNA	.	11.4
TAS2R50	taste 2 receptor member 50	protein_coding	NP_795371.2	11.3
LOC112267967	uncharacterized LOC112267967	lncRNA	.	11.3
RPL31P46	ribosomal protein L31 pseudogene 46	pseudogene	.	11.3
RN7SL735P	RNA, 7SL, cytoplasmic 735, pseudogene	pseudogene	.	11.2
LOC107986061	uncharacterized LOC107986061	lncRNA	.	11.2
PTPRB	protein tyrosine phosphatase receptor type B	protein_coding	NP_001103224.1;NP_001193900.1;	11.2
TALAM1	TALAM1 transcript, MALAT1 antisense RNA	antisense_RNA	.	11.2
LINC01920	long intergenic non-protein coding RNA 1920	lncRNA	.	11.2
LOC105369354	uncharacterized LOC105369354	lncRNA	.	11.1
CBX5P1	chromobox 5 pseudogene 1	pseudogene	.	11.1
FCF1P2	FCF1 pseudogene 2	ranscribed_pseudogen	.	11.1
LOC100422561	receptor for activated C kinase 1 pseudogene	pseudogene	.	11.0
MIR583HG	MIR583 host gene	lncRNA	.	11.0
LOC107985725	uncharacterized LOC107985725	lncRNA	.	11.0
LOC107987112	uncharacterized LOC107987112	lncRNA	.	10.9
C1QTNF3-AMACR	C1QTNF3-AMACR readthrough (NMD candidate	lncRNA	.	10.9
PHBP2	prohibitin pseudogene 2	pseudogene	.	10.8
TAS2R64P	taste 2 receptor member 64 pseudogene	pseudogene	.	10.8
HNRNPA1P3	heterogeneous nuclear ribonucleoprotein A1 pseuc	pseudogene	.	10.8
KCNQ5-IT1	KCNQ5 intronic transcript 1	lncRNA	.	10.7
RPS27P19	ribosomal protein S27 pseudogene 19	pseudogene	.	10.7
RPS2P49	ribosomal protein S2 pseudogene 49	pseudogene	.	10.7
RPL39P19	ribosomal protein L39 pseudogene 19	pseudogene	.	10.7
LOC100421523	UDP-N-acetylglucosamine pyrophosphorylase 1 p	pseudogene	.	10.6
LOC107984581	uncharacterized LOC107984581	lncRNA	.	10.6
CZ1P-ASNS	CZ1P-ASNS readthrough	lncRNA	.	10.6
RPL36AP15	ribosomal protein L36a pseudogene 15	pseudogene	.	10.5
LOC107984406	uncharacterized LOC107984406	lncRNA	.	10.5
INSYN2B	inhibitory synaptic factor family member 2B	protein_coding	NP_001123363.1;NP_001333233.1;	10.4
GNG12-AS1	GNG12, DIRAS3 and WLS antisense RNA 1	lncRNA	.	10.4
LOC389602	uncharacterized LOC389602	lncRNA	.	10.4
LOC101928278	uncharacterized LOC101928278, transcript varian	lncRNA	.	10.4
LOC100996724	phosphodiesterase 4D interacting protein-like	ranscribed_pseudogen	.	10.4
BLID	BH3-like motif containing, cell death inducer	protein_coding	NP_001001786.2	10.3
LOC102467080	uncharacterized LOC102467080	lncRNA	.	10.3
LOC100420174	centrosomal protein 170kDa pseudogene	pseudogene	.	10.3

LOC105378477	uncharacterized LOC105378477, transcript varian	lncRNA	.	10.3
RPL36AP33	ribosomal protein L36a pseudogene 33	pseudogene	.	10.3
NMRAL2P	NmrA like redox sensor 2, pseudogene	ranscribed_pseudogen	.	10.2
LOC107985153	uncharacterized LOC107985153	lncRNA	.	10.2
ALG3P1	ALG3 pseudogene 1	pseudogene	.	10.2
RNY4P34	RNY4 pseudogene 34	pseudogene	.	10.2
RELN	reelin	protein_coding	NP_005036.2;NP_774959.1	10.1
SNORD170	small nucleolar RNA, C/D box 170	snoRNA	.	10.1
LOC105370135	uncharacterized LOC105370135	lncRNA	.	10.1
LOC503540	AKT interacting protein pseudogene	pseudogene	.	10.1
LOC107984329	uncharacterized LOC107984329	lncRNA	.	10.1
MIR548U	microRNA 548u	miRNA	.	10.1
LOC105373785	uncharacterized LOC105373785, transcript varian	lncRNA	.	10.0
INHBA	inhibin subunit beta A	protein_coding	NP_002183.1;XP_016867663.1;XP_	10.0
RN7SL127P	RNA, 7SL, cytoplasmic 127, pseudogene	pseudogene	.	9.9
ITGB1BP2	integrin subunit beta 1 binding protein 2	protein_coding	NP_001290206.1;NP_036410.1	9.9
LOC107986031	uncharacterized LOC107986031	lncRNA	.	9.9
LOC105377524	uncharacterized LOC105377524, transcript varian	lncRNA	.	9.8
SPP1	secreted phosphoprotein 1	protein_coding	NP_000573.1;NP_001035147.1;NP_	9.8
ND4L	NADH dehydrogenase subunit 4L	protein_coding	YP_003024034.1	9.8
LOC107986016	uncharacterized LOC107986016	lncRNA	.	9.8
LOC105370456	uncharacterized LOC105370456, transcript varian	lncRNA	.	9.8
BACH1-IT2	BACH1 intronic transcript 2	lncRNA	.	9.7
LOC107986365	uncharacterized LOC107986365	lncRNA	.	9.7
SERPINB2	serpin family B member 2	protein_coding	NP_001137290.1;NP_002566.1;XP_	9.6
LOC100421868	glucose-fructose oxidoreductase domain containin	pseudogene	.	9.6
RAB40AL	RAB40A like	protein_coding	NP_001027004.1	9.6
LOC101928636	uncharacterized LOC101928636, transcript varian	lncRNA	.	9.6
LOC100132154	ankyrin repeat domain 30B pseudogene, transcript	ranscribed_pseudogen	.	9.6
DDX60L	DExD/H-box 60 like	protein_coding	NP_001012985.2;NP_001278439.1;	9.5
XIRP2	xin actin binding repeat containing 2	protein_coding	NP_001073278.1;NP_001186072.1;	9.5
LOC105372571	uncharacterized LOC105372571, transcript varian	lncRNA	.	9.5
LOC112268021	uncharacterized LOC112268021, transcript varian	lncRNA	.	9.5
RN7SL141P	RNA, 7SL, cytoplasmic 141, pseudogene	pseudogene	.	9.5
SND1-IT1	SND1 intronic transcript 1	lncRNA	.	9.5
FLJ31104	uncharacterized LOC441072	lncRNA	.	9.4
LOC100421309	DEAD-box helicase 25 pseudogene	pseudogene	.	9.4
SNORD98	small nucleolar RNA, C/D box 98	snoRNA	.	9.4
NABP1-OT1	uncharacterized LOC105747689	lncRNA	.	9.4
MAGI1-IT1	MAGI1 intronic transcript 1	lncRNA	.	9.4
GCNA	germ cell nuclear acidic peptidase	protein_coding	NP_443189.1;XP_006724779.1;XP_	9.4
LOC100506178	uncharacterized LOC100506178	lncRNA	.	9.4
RPL7P18	ribosomal protein L7 pseudogene 18	pseudogene	.	9.3
LOC105376244	uncharacterized LOC105376244, transcript varian	lncRNA	.	9.3
TRBV12-4	T cell receptor beta variable 12-4	V_segment	.	9.3
LINC02100	long intergenic non-protein coding RNA 2100, tra	lncRNA	.	9.3
LOC105376212	uncharacterized LOC105376212, transcript varian	lncRNA	.	9.3
RNU4ATAC16P	RNA, U4atac small nuclear 16, pseudogene	pseudogene	.	9.3
LOC643015	nucleolar protein 11 pseudogene	pseudogene	.	9.3
TNPO1P1	transportin 1 pseudogene 1	pseudogene	.	9.3
SNCAIP	synuclein alpha interacting protein	protein_coding	NP_001229864.1;NP_001295029.1;	9.3
LOC107986970	uncharacterized LOC107986970	lncRNA	.	9.3
TAS2R43	taste 2 receptor member 43	protein_coding	NP_795365.2	9.3
RPS3AP38	RPS3A pseudogene 38	pseudogene	.	9.2
LOC101928096	uncharacterized LOC101928096	lncRNA	.	9.2
RN7SL473P	RNA, 7SL, cytoplasmic 473, pseudogene	pseudogene	.	9.2
GOLGA6L3	golgin A6 family like 3	protein_coding	NP_001297082.1	9.1
LOC105374494	uncharacterized LOC105374494	lncRNA	.	9.1
LDHAL6FP	lactate dehydrogenase A like 6F, pseudogene	pseudogene	.	9.1
RPS12P3	ribosomal protein S12 pseudogene 3	pseudogene	.	9.1
RN7SL288P	RNA, 7SL, cytoplasmic 288, pseudogene	pseudogene	.	9.1
NBPF8	NBPF member 8	protein_coding	NP_001032590.2	9.1
LOC105377473	uncharacterized LOC105377473, transcript varian	lncRNA	.	9.1
RN7SL220P	RNA, 7SL, cytoplasmic 220, pseudogene	pseudogene	.	9.1
RNF14P1	RNF14 pseudogene 1	pseudogene	.	9.0
LOC644285	uncharacterized LOC644285	lncRNA	.	9.0
CFAP299	cilia and flagella associated protein 299	protein_coding	NP_001193926.1;NP_689983.2;XP_	9.0
CDKL4	cyclin dependent kinase like 4	protein_coding	NP_001009565.1;NP_001333840.1;	9.0
LOC107985930	uncharacterized LOC107985930	lncRNA	.	9.0
LOC112267879	uncharacterized LOC112267879	lncRNA	.	9.0
RBMX2P3	RBMX2 pseudogene 3	pseudogene	.	8.9

LOC107984247	uncharacterized LOC107984247, transcript varian	lncRNA	.	8.9
SNORA116	small nucleolar RNA, H/ACA box 116	snoRNA	.	8.9
TAS2R14	taste 2 receptor member 14	protein_coding	NP_076411.1	8.8
LOC112267935	uncharacterized LOC112267935	lncRNA	.	8.8
LOC105370569	uncharacterized LOC105370569	lncRNA	.	8.8
RBBP4P1	RBBP4 pseudogene 1	pseudogene	.	8.8
TAS2R46	taste 2 receptor member 46	protein_coding	NP_795368.2	8.8
LOC107985522	uncharacterized LOC107985522	lncRNA	.	8.8
LOC107984546	uncharacterized LOC107984546	lncRNA	.	8.8
LOC105378031	uncharacterized LOC105378031, transcript varian	lncRNA	.	8.8
LOC107984805	uncharacterized LOC107984805, transcript varian	lncRNA	.	8.8
LOC645965	adipose differentiation-related protein pseudogene	pseudogene	.	8.8
SNORD116-28	small nucleolar RNA, C/D box 116-28	snoRNA	.	8.8
G3BP1P1	G3BP1 pseudogene 1	pseudogene	.	8.7
ARHGAP22	Rho GTPase activating protein 22	protein_coding	NP_001242953.1;NP_001242954.1;	8.7
TAS2R19	taste 2 receptor member 19	protein_coding	NP_795369.1	8.7
RPS12P14	ribosomal protein S12 pseudogene 14	pseudogene	.	8.7
LETM1P3	leucine zipper and EF-hand containing transmemb	pseudogene	.	8.7
SNX18P11	sorting nexin 18 pseudogene 11	pseudogene	.	8.7
LOC105375835	uncharacterized LOC105375835	lncRNA	.	8.7
RNFT1P1	ring finger protein, transmembrane 1 pseudogene	pseudogene	.	8.7
NPIPA9	nuclear pore complex interacting protein family, n	pseudogene	.	8.7
PGAM1P7	phosphoglycerate mutase 1 pseudogene 7	pseudogene	.	8.7
LOC107986948	uncharacterized LOC107986948	lncRNA	.	8.6
ITGBL1	integrin subunit beta like 1	protein_coding	NP_001258683.1;NP_001258684.1;	8.6
RPS20P9	ribosomal protein S20 pseudogene 9	pseudogene	.	8.6
GOLGA8B	golgin A8 family member B	protein_coding	NP_001018861.3	8.6
KYNU	kynureninase	protein_coding	NP_001028170.1;NP_001186170.1;	8.6
PHF5DP	PHD finger protein 5D pseudogene	pseudogene	.	8.6
AK3P3	adenylate kinase 3 pseudogene 3	pseudogene	.	8.6
ARF1P2	ADP ribosylation factor 1 pseudogene 2	pseudogene	.	8.6
SAT1	spermidine/spermine N1-acetyltransferase 1	protein_coding	NP_002961.1;XP_024308189.1	8.6
NBPF19	NBPF member 19	protein_coding	NP_001338294.1	8.6
RPL7AP22	ribosomal protein L7a pseudogene 22	pseudogene	.	8.6
USF1P1	upstream transcription factor 1 pseudogene 1	pseudogene	.	8.5
LOC105371224	uncharacterized LOC105371224, transcript varian	lncRNA	.	8.5
LOC105377872	uncharacterized LOC105377872, transcript varian	lncRNA	.	8.5
TAS2R13	taste 2 receptor member 13	protein_coding	NP_076409.1	8.5
DAD1P1	defender against cell death 1 pseudogene 1	pseudogene	.	8.5
LOC105377310	syncytin-1-like, transcript variant X2	protein_coding	XP_011530744.1	8.5
HTR7	5-hydroxytryptamine receptor 7	protein_coding	NP_000863.1;NP_062873.1;NP_06	8.5
LOC102724404	uncharacterized LOC102724404	lncRNA	.	8.5
LOC100422495	protein tyrosine phosphatase non-receptor type 4 p	pseudogene	.	8.5
FLJ42393	uncharacterized LOC401105	lncRNA	.	8.5
SMARCE1P1	SWI/SNF related, matrix associated, actin depend	pseudogene	.	8.4
RPL7L1P12	ribosomal protein L7 like 1 pseudogene 12	pseudogene	.	8.4
ZNF33BP1	zinc finger protein 33B pseudogene 1	ranscribed_pseudogen	.	8.4
TMEM200A	transmembrane protein 200A	protein_coding	NP_001245205.1;NP_001245206.1;	8.4
SLC35F4	solute carrier family 35 member F4	protein_coding	NP_001193849.1;NP_001293016.1;	8.4
NBPF26	NBPF member 26	protein_coding	NP_001338301.1	8.4
LOC105373873	uncharacterized LOC105373873, transcript varian	lncRNA	.	8.4
LOC100506076	uncharacterized LOC100506076	lncRNA	.	8.4
SMCO2	single-pass membrane protein with coiled-coil dor	protein_coding	NP_001138482.1;XP_011518936.1;	8.4
LOC105369228	uncharacterized LOC105369228	lncRNA	.	8.4
SETP6	SET pseudogene 6	pseudogene	.	8.4
LOC105373802	uncharacterized LOC105373802, transcript varian	lncRNA	.	8.4
SNRPCP19	small nuclear ribonucleoprotein polypeptide C pse	pseudogene	.	8.3
LINC01191	long intergenic non-protein coding RNA 1191	lncRNA	.	8.3
GOLGA8A	golgin A8 family member A	protein_coding	NP_001355000.1;NP_001355001.1;	8.3
KRT18P31	keratin 18 pseudogene 31	pseudogene	.	8.3
LOC100287290	cytokine receptor CRL2, transcript variant X9	misc_RNA	.	8.3
PGAM1P10	phosphoglycerate mutase 1 pseudogene 10	pseudogene	.	8.3
RNU2-5P	RNA, U2 small nuclear 5, pseudogene	pseudogene	.	8.3
LINC01762	long intergenic non-protein coding RNA 1762	lncRNA	.	8.3
LOC105375821	uncharacterized LOC105375821, transcript varian	lncRNA	.	8.3
DDX58	DExD/H-box helicase 58	protein_coding	NP_055129.2	8.3
OR5P2	olfactory receptor family 5 subfamily P member 2	protein_coding	NP_703145.1	8.3
RCSD1	RCSD domain containing 1	protein_coding	NP_001309852.1;NP_001309853.1;	8.3
KRT8P52	keratin 8 pseudogene 52	pseudogene	.	8.2
LOC644584	EWS RNA binding protein 1 pseudogene	pseudogene	.	8.2
TPRG1	tumor protein p63 regulated 1	protein_coding	NP_940887.1;XP_005247439.1;XP_	8.2

PA2G4P4	proliferation-associated 2G4 pseudogene 4	ranscribed_pseudogen	.	8.2
LOC107987022	.	pseudogene	.	8.2
CAVIN2-AS1	CAVIN2 and TMEFF2 antisense RNA 1, transcrip	lncRNA	.	8.2
RPS4XP17	ribosomal protein S4X pseudogene 17	pseudogene	.	8.2
RPL7AP20	ribosomal protein L7a pseudogene 20	pseudogene	.	8.1
LOC105376987	uncharacterized LOC105376987, transcript varian	lncRNA	.	8.1
OSTCP8	oligosaccharyltransferase complex subunit pseudo	pseudogene	.	8.1
RPSAP21	ribosomal protein SA pseudogene 21	pseudogene	.	8.1
FABP5P9	fatty acid binding protein 5 pseudogene 9	pseudogene	.	8.1
MRPL35P3	mitochondrial ribosomal protein L35 pseudogene	pseudogene	.	8.1
MIR3935	microRNA 3935	miRNA	.	8.1
LINC00431	long intergenic non-protein coding RNA 431	lncRNA	.	8.1
DNHD1	dynein heavy chain domain 1	protein_coding	NP_653267.2;NP_775860.3	8.0
LOC112268443	.	pseudogene	.	8.0
LRRC8C	leucine rich repeat containing 8 VRAC subunit C	protein_coding	NP_115646.3;XP_006711023.1;XP_	8.0
FTO-IT1	FTO intronic transcript 1	lncRNA	.	8.0
LOC100422414	proteasome (prosome, macropain) 26S subunit, no	pseudogene	.	8.0
CDRT3	CMT1A duplicated region transcript 3	lncRNA	.	8.0
LOC390933	PAT1 homolog 1, processing body mRNA decay f	pseudogene	.	8.0
LINC02728	long intergenic non-protein coding RNA 2728	lncRNA	.	8.0
PSG6	pregnancy specific beta-1-glycoprotein 6	protein_coding	NP_001027020.1;NP_002773.1	7.9
LOC107986515	uncharacterized LOC107986515	lncRNA	.	7.9
USP18	ubiquitin specific peptidase 18	protein_coding	NP_059110.2;XP_006724137.1	7.9
LOC112267930	uncharacterized LOC112267930	protein_coding	XP_024302045.1	7.9
LOC112268227	uncharacterized LOC112268227	lncRNA	.	7.9
LOC100131471	presenilins-associated rhomboid-like protein, mito	pseudogene	.	7.9
IGFN1	immunoglobulin like and fibronectin type III dom	protein_coding	NP_001158058.1;NP_001354770.1;	7.9
LOC105377148	uncharacterized LOC105377148, transcript varian	lncRNA	.	7.9
NBPF14	NBPF member 14	protein_coding	NP_056198.2	7.9
METTL21AP1	methyltransferase like 21A pseudogene 1	pseudogene	.	7.8
LOC105373644	uncharacterized LOC105373644	lncRNA	.	7.8
LOC112268063	uncharacterized LOC112268063, transcript varian	lncRNA	.	7.8
LOC107985349	uncharacterized LOC107985349, transcript varian	lncRNA	.	7.8
LOC112268214	uncharacterized LOC112268214	lncRNA	.	7.8
CD24P2	CD24 molecule pseudogene 2	pseudogene	.	7.7
LOC107984489	uncharacterized LOC107984489	lncRNA	.	7.7
NBPF20	NBPF member 20	protein_coding	NP_001265196.1	7.7
LOC105378779	uncharacterized LOC105378779	lncRNA	.	7.7
LOC112268086	uncharacterized LOC112268086	lncRNA	.	7.7
SNORD13E	small nucleolar RNA, C/D box 13E	snoRNA	.	7.7
RPL23AP34	ribosomal protein L23a pseudogene 34	pseudogene	.	7.7
UC.134	long non-coding RNA uc.134	lncRNA	.	7.7
PSMD6-AS2	PSMD6 antisense RNA 2	lncRNA	.	7.6
HDAC9	histone deacetylase 9	protein_coding	NP_001191073.1;NP_001191074.1;	7.6
LOC100421517	DnaJ heat shock protein family (Hsp40) member C	pseudogene	.	7.6
RPS16P5	ribosomal protein S16 pseudogene 5	ranscribed_pseudogen	.	7.6
MIR3140	microRNA 3140	miRNA	.	7.6
CAP2P1	cyclase associated actin cytoskeleton regulatory pr	pseudogene	.	7.6
LOC100128721	stromal cell derived factor 2 pseudogene	pseudogene	.	7.6
LOC105370924	uncharacterized LOC105370924	lncRNA	.	7.6
LOC107984127	uncharacterized LOC107984127	lncRNA	.	7.6
LOC105370461	uncharacterized LOC105370461, transcript varian	lncRNA	.	7.6
PCED1B	PC-esterase domain containing 1B	protein_coding	NP_001268358.1;NP_612380.1;XP_	7.6
LOC107986949	uncharacterized LOC107986949	lncRNA	.	7.5
FTX	FTX transcript, XIST regulator	lncRNA	.	7.5
LOC646358	DnaJ heat shock protein family (Hsp40) member E	pseudogene	.	7.5
FLACC1	flagellum associated containing coiled-coil domai	protein_coding	NP_001120863.1;NP_001276922.1;	7.5
LOC107986068	uncharacterized LOC107986068	lncRNA	.	7.5
EEF1A1P3	eukaryotic translation elongation factor 1 alpha 1 p	pseudogene	.	7.5
RPS26P7	ribosomal protein S26 pseudogene 7	pseudogene	.	7.5
LOC112268133	uncharacterized LOC112268133	lncRNA	.	7.5
PHBP13	prohibitin pseudogene 13	pseudogene	.	7.5
SLMO2-ATP5E	SLMO2-ATP5E readthrough	lncRNA	.	7.4
GCNT1P2	glucosaminyl (N-acetyl) transferase 1, core 2 pseu	pseudogene	.	7.4
SAMD9L	sterile alpha motif domain containing 9 like	protein_coding	NP_001290425.1;NP_001290426.1;	7.4
RPL31P27	ribosomal protein L31 pseudogene 27	pseudogene	.	7.4
LINC02803	long intergenic non-protein coding RNA 2803	lncRNA	.	7.4
MIR3939	microRNA 3939	miRNA	.	7.4
LOC105369194	uncharacterized LOC105369194, transcript varian	lncRNA	.	7.4
RUNX1-IT1	RUNX1 intronic transcript 1	lncRNA	.	7.4
TNFAIP3	TNF alpha induced protein 3	protein_coding	NP_001257436.1;NP_001257437.1;	7.4

LOC112268122	uncharacterized LOC112268122, transcript varian	lncRNA	.	7.4
GAPDHP43	glyceraldehyde 3 phosphate dehydrogenase pseud	pseudogene	.	7.4
RPS14P4	ribosomal protein S14 pseudogene 4	pseudogene	.	7.4
RPS2P44	ribosomal protein S2 pseudogene 44	pseudogene	.	7.3
RPS14P5	ribosomal protein S14 pseudogene 5	pseudogene	.	7.3
LOC105371954	uncharacterized LOC105371954, transcript varian	lncRNA	.	7.3
LOC644303	ATP-dependent RNA helicase DDX24-like	pseudogene	.	7.3
CTBP2P8	CTBP2 pseudogene 8	pseudogene	.	7.3
SP100	SP100 nuclear antigen	protein_coding	NP_001073860.1;NP_001193630.1;	7.3
SMG1P6	SMG1 pseudogene 6	ranscribed_pseudogen	.	7.3
LOC100288798	uncharacterized LOC100288798	lncRNA	.	7.3
GPR21	G protein-coupled receptor 21	protein_coding	NP_005285.1;XP_005251990.1	7.3
LOC100128007	Spi-C transcription factor (Spi-1/PU.1 related) pse	pseudogene	.	7.3
SNORD159	small nucleolar RNA, C/D box 159	snoRNA	.	7.3
LOC107985523	uncharacterized LOC107985523	lncRNA	.	7.2
LINC01619	long intergenic non-protein coding RNA 1619	lncRNA	.	7.2
SCG5	secretogranin V	protein_coding	NP_001138229.1;NP_003011.1	7.2
PDLIM1P4	PDZ and LIM domain 1 pseudogene 4	pseudogene	.	7.2
PNPT1P1	polyribonucleotide nucleotidyltransferase 1 pseud	pseudogene	.	7.2
LOC105377016	uncharacterized LOC105377016, transcript varian	lncRNA	.	7.2
LOC100506123	uncharacterized LOC100506123	lncRNA	.	7.2
RPSAP36	ribosomal protein SA pseudogene 36	pseudogene	.	7.1
SNORD53	small nucleolar RNA, C/D box 53	snoRNA	.	7.1
LOC107983976	uncharacterized LOC107983976	lncRNA	.	7.1
TAF9P3	TATA-box binding protein associated factor 9 pse	pseudogene	.	7.1
RPL23AP63	ribosomal protein L23a pseudogene 63	pseudogene	.	7.1
LOC107986673	uncharacterized LOC107986673	lncRNA	.	7.1
LOC105376095	uncharacterized LOC105376095, transcript varian	lncRNA	.	7.1
ARHGAP26-IT1	ARHGAP26 intronic transcript 1	lncRNA	.	7.1
HSPA8P4	heat shock protein family A (Hsp70) member 8 ps	pseudogene	.	7.1
EBF1	EBF transcription factor 1	protein_coding	NP_001277289.1;NP_001311030.1;	7.1
IFI27	interferon alpha inducible protein 27	protein_coding	NP_001275883.1;NP_001275886.1;	7.1
LYPLAL1-DT	LYPLAL1 divergent transcript	lncRNA	.	7.1
NFE2L3P2	nuclear factor, erythroid 2 like 3 pseudogene 2	pseudogene	.	7.1
LOC100128361	uncharacterized LOC100128361	lncRNA	.	7.1
RARB	retinoic acid receptor beta	protein_coding	NP_000956.2;NP_001277145.1;NP_	7.1
TIMM23B-AGAP6	TIMM23B-AGAP6 readthrough (NMD candidate)	lncRNA	.	7.1
PARP9	poly(ADP-ribose) polymerase family member 9	protein_coding	NP_001139574.1;NP_001139575.1;	7.0
XBP1P1	X-box binding protein 1 pseudogene 1	pseudogene	.	7.0
LINC00597	long intergenic non-protein coding RNA 597	lncRNA	.	7.0
FTH1P15	ferritin heavy chain 1 pseudogene 15	pseudogene	.	7.0
CFAP44	cilia and flagella associated protein 44	protein_coding	NP_001157968.1;NP_060808.2	7.0
RPL35AP9	ribosomal protein L35a pseudogene 9	pseudogene	.	7.0
LOC112268009	uncharacterized LOC112268009, transcript varian	lncRNA	.	7.0
RPL7AP16	ribosomal protein L7a pseudogene 16	pseudogene	.	7.0
LOC100421580	abraxas 1, BRCA1 A complex subunit pseudogene	pseudogene	.	7.0
PCAT1	prostate cancer associated transcript 1	lncRNA	.	7.0
IMMP2L	inner mitochondrial membrane peptidase subunit 2	protein_coding	NP_001231535.1;NP_001337888.1;	7.0
LINC02193	long intergenic non-protein coding RNA 2193	lncRNA	.	7.0
LOC105369212	uncharacterized LOC105369212	lncRNA	.	6.9
LOC101929594	uncharacterized LOC101929594, transcript varian	lncRNA	.	6.9
ARL2-SNX15	ARL2-SNX15 readthrough (NMD candidate)	lncRNA	.	6.9
PARP14	poly(ADP-ribose) polymerase family member 14	protein_coding	NP_060024.2;XP_011511230.1;XP_	6.9
LINC00622	long intergenic non-protein coding RNA 622	lncRNA	.	6.9
YWHABP2	tyrosine 3-monooxygenase/tryptophan 5-monooxy	pseudogene	.	6.9
SNORD53B	small nucleolar RNA, C/D box 53B	snoRNA	.	6.9
LINC02863	long intergenic non-protein coding RNA 2863, tra	lncRNA	.	6.9
GOLGA6L17P	golgin A6 family like 17, pseudogene	ranscribed_pseudogen	.	6.9
LOC107986254	uncharacterized LOC107986254	lncRNA	.	6.9
RPS12P5	ribosomal protein S12 pseudogene 5	pseudogene	.	6.9
LOC105371366	uncharacterized LOC105371366, transcript varian	lncRNA	.	6.9
RPL12P21	ribosomal protein L12 pseudogene 21	pseudogene	.	6.9
TEKT3	tektin 3	protein_coding	NP_114104.1;XP_011522290.1;XP_	6.9
RPL35P3	ribosomal protein L35 pseudogene 3	pseudogene	.	6.9
RPL17P38	ribosomal protein L17 pseudogene 38	pseudogene	.	6.9
EPSTI1	epithelial stromal interaction 1	protein_coding	NP_001002264.1;NP_001317472.1;	6.9
HMGN2P28	high mobility group nucleosomal binding domain	pseudogene	.	6.9
CNTNAP4	contactin associated protein family member 4	protein_coding	NP_001309107.1;NP_001309108.1;	6.8
LOC107986065	uncharacterized LOC107986065	lncRNA	.	6.8
LSM1P1	LSM1 homolog, mRNA degradation associated ps	pseudogene	.	6.8
RPL21P123	ribosomal protein L21 pseudogene 123	pseudogene	.	6.8

LOC105377116	uncharacterized LOC105377116, transcript varian	lncRNA	.	6.8
LOC107984960	uncharacterized LOC107984960, transcript varian	lncRNA	.	6.8
LOC107986028	uncharacterized LOC107986028	lncRNA	.	6.8
IFI44	interferon induced protein 44	protein_coding	NP_006408.3;XP_005270437.1;XP_	6.8
RPS12P16	ribosomal protein S12 pseudogene 16	pseudogene	.	6.8
MAD2L1P1	mitotic arrest deficient 2 like 1 pseudogene 1	pseudogene	.	6.8
CAPZA1P2	CAPZA1 pseudogene 2	pseudogene	.	6.8
LOC105369349	uncharacterized LOC105369349	lncRNA	.	6.8
MIR5693	microRNA 5693	miRNA	.	6.7
RPS27P7	ribosomal protein S27 pseudogene 7	pseudogene	.	6.7
HSPE1P17	heat shock protein family E (Hsp10) member 1 ps	pseudogene	.	6.7
C3orf52	chromosome 3 open reading frame 52	protein_coding	NP_001165218.1;NP_078892.3	6.7
LOC112268195	uncharacterized LOC112268195, transcript varian	lncRNA	.	6.7
RPL36AP24	ribosomal protein L36a pseudogene 24	pseudogene	.	6.7
LOC107986345	uncharacterized LOC107986345	lncRNA	.	6.7
LOC105378859	uncharacterized LOC105378859, transcript varian	lncRNA	.	6.7
RN7SL587P	RNA, 7SL, cytoplasmic 587, pseudogene	pseudogene	.	6.7
MIR623	microRNA 623	miRNA	.	6.7
RPS26P20	ribosomal protein S26 pseudogene 20	pseudogene	.	6.7
RN7SKP187	RN7SK pseudogene 187	pseudogene	.	6.7
LOC105373637	uncharacterized LOC105373637	lncRNA	.	6.7
LOC105369161	uncharacterized LOC105369161	lncRNA	.	6.7
LOC107984112	uncharacterized LOC107984112, transcript varian	lncRNA	.	6.7
LOC107986513	uncharacterized LOC107986513	lncRNA	.	6.7
ARMCX5-GPRASP2	ARMCX5-GPRASP2 readthrough	protein_coding	NP_001186747.1;NP_001337197.1;	6.7
ST13P11	ST13, Hsp70 interacting protein pseudogene 11	pseudogene	.	6.6
KRT18P18	keratin 18 pseudogene 18	pseudogene	.	6.6
LOC105378721	uncharacterized LOC105378721	lncRNA	.	6.6
LOC100128523	mitochondrial carrier 2 pseudogene	pseudogene	.	6.6
LRRC49	leucine rich repeat containing 49	protein_coding	NP_001185946.1;NP_001185947.1;	6.6
SNORD164	small nucleolar RNA, C/D box 164	snoRNA	.	6.6
HECW1-IT1	HECW1 intronic transcript 1	lncRNA	.	6.6
TMLHE-AS1	TMLHE antisense RNA 1	lncRNA	.	6.6
ERAP2	endoplasmic reticulum aminopeptidase 2	protein_coding	NP_001123612.1;NP_001316158.1;	6.6
MIR6124	microRNA 6124	miRNA	.	6.6
SNORA87	small nucleolar RNA, H/ACA box 87	snoRNA	.	6.6
LOC105369318	uncharacterized LOC105369318	lncRNA	.	6.6
SEM1	SEM1 26S proteasome complex subunit	protein_coding	NP_006295.1;XP_024302702.1	6.6
CD274	CD274 molecule	protein_coding	NP_001254635.1;NP_001300958.1;	6.6
LOC107986168	uncharacterized LOC107986168	lncRNA	.	6.6
NBPF10	NBPF member 10	protein_coding	NP_001034792.4;NP_001289300.1	6.6
LOC100127917	heat shock protein 90 alpha family class B membe	pseudogene	.	6.6
TNFRSF11B	TNF receptor superfamily member 11b	protein_coding	NP_002537.3	6.6
ALDH1L2	aldehyde dehydrogenase 1 family member L2	protein_coding	NP_001029345.2;XP_011536288.1;	6.5
DEPDC1P2	DEP domain containing 1 pseudogene 2	pseudogene	.	6.5
TTN	titin	protein_coding	NP_001243779.1;NP_001254479.2;	6.5
TAS2R4	taste 2 receptor member 4	protein_coding	NP_058640.1	6.5
R3HDM2P2	R3H domain containing 2 pseudogene 2	pseudogene	.	6.5
DUSP10	dual specificity phosphatase 10	protein_coding	NP_009138.1;XP_016855636.1	6.5
SLC2A3P1	solute carrier family 2 member 3 pseudogene 1	pseudogene	.	6.5
LOC100128803	Spi-C transcription factor (Spi-1/PU.1 related) pse	pseudogene	.	6.5
TBILA	TGF-beta induced lncRNA	lncRNA	.	6.5
TAS2R31	taste 2 receptor member 31	protein_coding	NP_795366.2	6.5
LOC107986347	uncharacterized LOC107986347	lncRNA	.	6.5
PELI2	pellino E3 ubiquitin protein ligase family member	protein_coding	NP_067078.1;XP_005267947.1;XP_	6.5
LOC107986153	uncharacterized LOC107986153	lncRNA	.	6.5
AP27P1-BPTFP1-KP	ARHGAP27P1-BPTFP1-KPNA2P3 readthrough,	ranscribed_pseudogen	.	6.4
DBI	diazepam binding inhibitor, acyl-CoA binding pro	protein_coding	NP_001073331.1;NP_001073332.1;	6.4
MIR5000	microRNA 5000	miRNA	.	6.4
DNAH12	dynein axonemal heavy chain 12	protein_coding	NP_001352957.1;NP_940966.2;XP_	6.4
SP110	SP110 nuclear body protein	protein_coding	NP_001171944.1;NP_001365371.1;	6.4
LOC105377374	uncharacterized LOC105377374, transcript varian	lncRNA	.	6.4
LOC105378936	uncharacterized LOC105378936	lncRNA	.	6.4
LINC01605	long intergenic non-protein coding RNA 1605	lncRNA	.	6.4
KRT18P19	keratin 18 pseudogene 19	pseudogene	.	6.4
PGK1P1	phosphoglycerate kinase 1 pseudogene 1	pseudogene	.	6.4
LOC107984580	uncharacterized LOC107984580	lncRNA	.	6.4
HMGN2P46	high mobility group nucleosomal binding domain	ranscribed_pseudogen	.	6.3
LOC107986707	uncharacterized LOC107986707	lncRNA	.	6.3
DOCK4	dedicator of cytokinesis 4	protein_coding	NP_001350469.1;NP_055520.3;XP_	6.3
PI15	peptidase inhibitor 15	protein_coding	NP_001311332.1;NP_056970.1	6.3

LOC107987174	uncharacterized LOC107987174	lncRNA	.	6.3
LOC107986195	uncharacterized LOC107986195	lncRNA	.	6.3
LOC105377684	uncharacterized LOC105377684, transcript varian	lncRNA	.	6.3
RPL7P7	ribosomal protein L7 pseudogene 7	pseudogene	.	6.3
LOC105369710	uncharacterized LOC105369710, transcript varian	lncRNA	.	6.3
CLEC2D	C-type lectin domain family 2 member D	protein_coding	NP_001004419.1;NP_001184246.1;	6.3
BIRC3	baculoviral IAP repeat containing 3	protein_coding	NP_001156.1;NP_892007.1;XP_02	6.3
BTF3P3	BTF3 pseudogene 3	pseudogene	.	6.3
LOC107984292	uncharacterized LOC107984292, transcript varian	lncRNA	.	6.3
SUMO2P6	SUMO2 pseudogene 6	pseudogene	.	6.3
LOC100130691	uncharacterized LOC100130691	lncRNA	.	6.3
TRBV13	T cell receptor beta variable 13	V_segment	.	6.3
LOC107984019	uncharacterized LOC107984019, transcript varian	lncRNA	.	6.3
SEC31B	SEC31 homolog B, COPII coat complex compone	protein_coding	NP_056305.1	6.3
DLGAP1-AS2	DLGAP1 antisense RNA 2	lncRNA	.	6.3
GLULP2	glutamate-ammonia ligase pseudogene 2	pseudogene	.	6.2
LINC02344	long intergenic non-protein coding RNA 2344	lncRNA	.	6.2
MIR3182	microRNA 3182	miRNA	.	6.2
HMGB3P28	high mobility group box 3 pseudogene 28	pseudogene	.	6.2
EML6	EMAP like 6	protein_coding	NP_001034842.2;XP_016859587.1;	6.2
DOCK9	dedicator of cytokinesis 9	protein_coding	NP_001123520.1;NP_001123521.1;	6.2
PHKA1P1	phosphorylase kinase regulatory subunit alpha 1 p	pseudogene	.	6.2
DDX60	DExD/H-box helicase 60	protein_coding	NP_060101.3;XP_011530405.1;XP_	6.2
ACTA2-AS1	ACTA2 antisense RNA 1	lncRNA	.	6.2
SMG1P4	SMG1 pseudogene 4	pseudogene	.	6.2
GPR135	G protein-coupled receptor 135	protein_coding	NP_072093.2;XP_016877085.1;XP_	6.2
LOC107986656	uncharacterized LOC107986656	lncRNA	.	6.2
LOC101927538	uncharacterized LOC101927538, transcript varian	lncRNA	.	6.2
SLC22A15	solute carrier family 22 member 15	protein_coding	NP_060890.2;XP_005271061.1;XP_	6.2
PCBP2P1	poly(rC) binding protein 2 pseudogene 1	pseudogene	.	6.1
TLK2P2	tousled like kinase 2 pseudogene 2	pseudogene	.	6.1
LOC101929709	uncharacterized LOC101929709	lncRNA	.	6.1
BMS1P2-AGAP9	BMS1P2-AGAP9 readthrough	ranscribed_pseudogen	.	6.1
LOC105370460	uncharacterized LOC105370460, transcript varian	lncRNA	.	6.1
LOC105377319	uncharacterized LOC105377319	lncRNA	.	6.1
SCARNA26B	small Cajal body-specific RNA 26B	guide_RNA	.	6.1
N4BP2L2-IT2	N4BPL2 intronic transcript 2	lncRNA	.	6.1
LOC105374717	uncharacterized LOC105374717	lncRNA	.	6.1
HERC2P2	hect domain and RLD 2 pseudogene 2	ranscribed_pseudogen	.	6.1
LOC107986457	uncharacterized LOC107986457	lncRNA	.	6.1
CDK8P2	cyclin dependent kinase 8 pseudogene 2	pseudogene	.	6.1
PLGLA	plasminogen like A (pseudogene)	ranscribed_pseudogen	.	6.1
TRAF1	TNF receptor associated factor 1	protein_coding	NP_001177874.1;NP_001177876.1;	6.1
LOC105377320	uncharacterized LOC105377320	lncRNA	.	6.1
SNORA50A	small nucleolar RNA, H/ACA box 50A	snoRNA	.	6.1
ITGA2	integrin subunit alpha 2	protein_coding	NP_002194.2	6.0
LOC105374438	uncharacterized LOC105374438, transcript varian	lncRNA	.	6.0
PRC1-AS1	PRC1 antisense RNA 1	lncRNA	.	6.0
ARHGAP24	Rho GTPase activating protein 24	protein_coding	NP_001020787.2;NP_001036134.1;	6.0
LOC105375284	uncharacterized LOC105375284, transcript varian	lncRNA	.	6.0
TMEM154	transmembrane protein 154	protein_coding	NP_689893.1;XP_011530018.1	6.0
CTSK	cathepsin K	protein_coding	NP_000387.1	6.0
LOC105370774	uncharacterized LOC105370774, transcript varian	lncRNA	.	6.0
LINC02615	long intergenic non-protein coding RNA 2615	lncRNA	.	6.0
SCARNA20	small Cajal body-specific RNA 20	guide_RNA	.	6.0
NR2F1-AS1	NR2F1 antisense RNA 1	lncRNA	.	6.0
GPR65	G protein-coupled receptor 65	protein_coding	NP_003599.2	6.0
TRIM66	tripartite motif containing 66	protein_coding	NP_055633.1;XP_006718460.1;XP_	6.0
LOC105371692	uncharacterized LOC105371692, transcript varian	lncRNA	.	6.0
WNT2B	Wnt family member 2B	protein_coding	NP_001278809.1;NP_004176.2;NP_	6.0
LOC105375933	uncharacterized LOC105375933, transcript varian	lncRNA	.	6.0
CATSPER2	cation channel sperm associated 2	protein_coding	NP_001269238.1;NP_001269239.1;	6.0
MIR2909	microRNA 2909	miRNA	.	6.0
RNU6-10P	RNA, U6 small nuclear 10, pseudogene	pseudogene	.	6.0
ABCF1-DT	ABCF1 divergent transcript	lncRNA	.	5.9
TNFSF4	TNF superfamily member 4	protein_coding	NP_001284491.1;NP_003317.1;XP_	5.9
ZCCHC7	zinc finger CCHC-type containing 7	protein_coding	NP_001276048.1;NP_001276049.1;	5.9
LINC01776	long intergenic non-protein coding RNA 1776	lncRNA	.	5.9
LOC107987128	uncharacterized LOC107987128	lncRNA	.	5.9
COPG2IT1	COPG2 imprinted transcript 1	lncRNA	.	5.9
LOC107986334	uncharacterized LOC107986334	lncRNA	.	5.9

TRIM69	tripartite motif containing 69	protein_coding	NP_001288073.1;NP_001288074.1;	5.9
LOC102724434	uncharacterized LOC102724434	lncRNA	.	5.9
SNORD4B	small nucleolar RNA, C/D box 4B	snoRNA	.	5.9
CASP4	caspase 4	protein_coding	NP_001216.1;NP_150649.1;XP_01	5.9
LOC100422232	Parkinsonism associated deglycase pseudogene	pseudogene	.	5.9
FKSG29	FKSG29	lncRNA	.	5.9
CCDC144CP	coiled-coil domain containing 144C, pseudogene	ranscribed_pseudogen	.	5.9
TNIP3	TNFAIP3 interacting protein 3	protein_coding	NP_001122315.2;NP_001231693.1;	5.8
CFAP161	cilia and flagella associated protein 161	protein_coding	NP_001340294.1;NP_775799.2;XP_	5.8
LOC102724830	uncharacterized LOC102724830, transcript varian	lncRNA	.	5.8
MIR3134	microRNA 3134	miRNA	.	5.8
SAA1	serum amyloid A1	protein_coding	NP_000322.2;NP_001171477.1;NP_	5.8
SAMD9	sterile alpha motif domain containing 9	protein_coding	NP_001180236.1;NP_060124.2	5.8
LOC102723862	uncharacterized LOC102723862, transcript varian	lncRNA	.	5.8
LOC107985727	.	pseudogene	.	5.8
RSC1A1	regulator of solute carriers 1	protein_coding	NP_006502.1	5.8
LOC105369601	uncharacterized LOC105369601, transcript varian	lncRNA	.	5.8
LOC285638	uncharacterized LOC285638	lncRNA	.	5.8
ACTBP4	ACTB pseudogene 4	pseudogene	.	5.8
HMGB3P9	high mobility group box 3 pseudogene 9	pseudogene	.	5.8
SNORA79	small nucleolar RNA, H/ACA box 79	snoRNA	.	5.8
LOC101927923	.	pseudogene	.	5.8
LINC00515	long intergenic non-protein coding RNA 515	lncRNA	.	5.8
TAS2R20	taste 2 receptor member 20	protein_coding	NP_795370.2	5.8
NBPF9	NBPF member 9	protein_coding	NP_001032764.2;NP_001264373.1	5.8
MALAT1	metastasis associated lung adenocarcinoma transc	lncRNA	.	5.8
PHLDA1	pleckstrin homology like domain family A membe	protein_coding	NP_031376.3	5.8
PMFBP1	polyamine modulated factor 1 binding protein 1	protein_coding	NP_001153685.1;NP_112583.2;XP_	5.8
LOC100419975	transmembrane protein 192 pseudogene	pseudogene	.	5.8
LINC02478	long intergenic non-protein coding RNA 2478	lncRNA	.	5.8
VEPH1	ventricular zone expressed PH domain containing	protein_coding	NP_001161383.1;NP_001161384.1;	5.8
COLEC10	collectin subfamily member 10	protein_coding	NP_001311024.1;NP_006429.2;XP_	5.8
OR4F3	olfactory receptor family 4 subfamily F member 3	protein_coding	NP_001005224.1;XP_016864827.1	5.8
HSPE1P11	heat shock protein family E (Hsp10) member 1 ps	pseudogene	.	5.8
LOC105374140	uncharacterized LOC105374140, transcript varian	lncRNA	.	5.8
SNORD94	small nucleolar RNA, C/D box 94	snoRNA	.	5.8
LOC105374897	uncharacterized LOC105374897	lncRNA	.	5.8
SNORA74C-2	small nucleolar RNA, H/ACA box 74C-2	snoRNA	.	5.7
LOC105377663	uncharacterized LOC105377663, transcript varian	lncRNA	.	5.7
LOC107984718	uncharacterized LOC107984718	lncRNA	.	5.7
LOC112268024	uncharacterized LOC112268024	lncRNA	.	5.7
KCNIP4	potassium voltage-gated channel interacting protei	protein_coding	NP_001030175.1;NP_001030176.1;	5.7
LOC105374128	uncharacterized LOC105374128, transcript varian	lncRNA	.	5.7
SNORD92	small nucleolar RNA, C/D box 92	snoRNA	.	5.7
B3GALT1	beta-1,3-galactosyltransferase 1	protein_coding	NP_066191.1;XP_005246988.1;XP_	5.7
CKS1BP4	CDC28 protein kinase regulatory subunit 1B pseu	pseudogene	.	5.7
LINC01355	long intergenic non-protein coding RNA 1355	lncRNA	.	5.7
SNX29P2	sorting nexin 29 pseudogene 2	ranscribed_pseudogen	.	5.7
LOC105374808	uncharacterized LOC105374808, transcript varian	lncRNA	.	5.7
LUCAT1	lung cancer associated transcript 1	lncRNA	.	5.7
LOC102724528	uncharacterized LOC102724528, transcript varian	lncRNA	.	5.7
VNN1	vanin 1	protein_coding	NP_004657.2	5.7
ZFPM2-AS1	ZFPM2 antisense RNA 1	lncRNA	.	5.7
HNRNPA1P2	heterogeneous nuclear ribonucleoprotein A1 pseu	pseudogene	.	5.7
CEP128	centrosomal protein 128	protein_coding	NP_689659.2;XP_011534792.1;XP_	5.7
LOC107984617	uncharacterized LOC107984617	lncRNA	.	5.7
LOC648927	lysine acetyltransferase 7 pseudogene	pseudogene	.	5.7
TBX19	T-box transcription factor 19	protein_coding	NP_005140.1	5.6
LOC105371711	uncharacterized LOC105371711	lncRNA	.	5.6
LINC02246	long intergenic non-protein coding RNA 2246	lncRNA	.	5.6
OR5P3	olfactory receptor family 5 subfamily P member 3	protein_coding	NP_703146.1	5.6
NRIP1	nuclear receptor interacting protein 1	protein_coding	NP_003480.2;XP_005261120.1;XP_	5.6
MIR8076	microRNA 8076	miRNA	.	5.6
HSP90B2P	heat shock protein 90 beta family member 2, pseu	ranscribed_pseudogen	.	5.6
MIR597	microRNA 597	miRNA	.	5.6
NBPF11	NBPF member 11	protein_coding	NP_001095133.3;NP_899228.4;XP_	5.6
MIR4451	microRNA 4451	miRNA	.	5.6
LOC101928516	uncharacterized LOC101928516	lncRNA	.	5.6
LOC100422627	TATA-box binding protein associated factor 4b ps	pseudogene	.	5.6
LOC102723872	uncharacterized LOC102723872, transcript varian	lncRNA	.	5.6
MMADHC-DT	MMADHC divergent transcript	lncRNA	.	5.6

RGS17P1	regulator of G protein signaling 17 pseudogene 1	pseudogene	.	5.6
LPAL2	lipoprotein(a) like 2, pseudogene	ranscribed_pseudogen	.	5.6
LOC105379025	uncharacterized LOC105379025, transcript varian	lncRNA	.	5.6
RPL11P3	ribosomal protein L11 pseudogene 3	pseudogene	.	5.6
ERC2	ELKS/RAB6-interacting/CAST family member 2	protein_coding	NP_056391.1;XP_016861627.1;XP_	5.6
ZC3H12C	zinc finger CCCH-type containing 12C	protein_coding	NP_203748.1;XP_005271772.1;XP_	5.6
HSPBAP1	HSPB1 associated protein 1	protein_coding	NP_001307657.1;NP_078886.2;XP_	5.6
LOC107986013	uncharacterized LOC107986013	lncRNA	.	5.6
LOC107984255	uncharacterized LOC107984255	lncRNA	.	5.6
LINC00641	long intergenic non-protein coding RNA 641	lncRNA	.	5.6
HERC2P3	hect domain and RLD 2 pseudogene 3	ranscribed_pseudogen	.	5.6
MIR569	microRNA 569	miRNA	.	5.6
LOC105376678	ovostatin-like	protein_coding	XP_011519205.1	5.5
LINC00852	long intergenic non-protein coding RNA 852	lncRNA	.	5.5
HSPD1P4	heat shock protein family D (Hsp60) member 1 ps	pseudogene	.	5.5
RND3	Rho family GTPase 3	protein_coding	NP_001241667.1;NP_005159.1	5.5
FGF5	fibroblast growth factor 5	protein_coding	NP_001278741.1;NP_004455.2;NP_	5.5
LL0XNC01-250H12.3	uncharacterized LL0XNC01-250H12.3, transcript	lncRNA	.	5.5
SNORD8	small nucleolar RNA, C/D box 8	snoRNA	.	5.5
LOC107986108	uncharacterized LOC107986108, transcript varian	lncRNA	.	5.5
NCKAP5-AS2	NCKAP5 antisense RNA 2	lncRNA	.	5.5
LOC105371505	uncharacterized LOC105371505	lncRNA	.	5.5
CCDC150	coiled-coil domain containing 150	protein_coding	NP_001074008.1;NP_001340268.1;	5.5
LOC107984312	uncharacterized LOC107984312	lncRNA	.	5.5
LOC101927400	uncharacterized LOC101927400, transcript varian	lncRNA	.	5.5
STPG4	sperm-tail PG-rich repeat containing 4	protein_coding	NP_001157033.1;NP_775920.1	5.5
LOC107986874	uncharacterized LOC107986874	lncRNA	.	5.5
LOC101927830	uncharacterized LOC101927830	lncRNA	.	5.5
LOC105375825	uncharacterized LOC105375825, transcript varian	lncRNA	.	5.5
SPRY4	sprouty RTK signaling antagonist 4	protein_coding	NP_001120968.1;NP_001280218.1;	5.5
LOC107986285	uncharacterized LOC107986285	lncRNA	.	5.5
MIR6758	microRNA 6758	miRNA	.	5.5
PLGLB2	plasminogen like B2	protein_coding	NP_002656.1	5.5
LOC105372964	uncharacterized LOC105372964	lncRNA	.	5.5
SMYD3	SET and MYND domain containing 3	protein_coding	NP_001161212.1;NP_001362891.1;	5.5
SMG1P3	SMG1 pseudogene 3	ranscribed_pseudogen	.	5.5
LRRC7-AS1	LRRC7 antisense RNA 1, transcript variant X3	lncRNA	.	5.4
SMG1P1	SMG1 pseudogene 1	ranscribed_pseudogen	.	5.4
LOC105378046	uncharacterized LOC105378046	lncRNA	.	5.4
LOC105374733	uncharacterized LOC105374733, transcript varian	lncRNA	.	5.4
LOC102725082	uncharacterized LOC102725082	lncRNA	.	5.4
SNORA75	small nucleolar RNA, H/ACA box 75	snoRNA	.	5.4
FAM247A	family with sequence similarity 247 member A	lncRNA	.	5.4
ZNNT1	ZNF706 neighboring transcript 1	lncRNA	.	5.4
LOC105379251	uncharacterized LOC105379251, transcript varian	lncRNA	.	5.4
NBPF15	NBPF member 15	protein_coding	NP_001164226.1;NP_775909.2	5.4
CTB-30L5.1	uncharacterized CTB-30L5.1	lncRNA	.	5.4
LOC105370567	uncharacterized LOC105370567	lncRNA	.	5.4
NONOP2	non-POU domain containing, octamer-binding pse	pseudogene	.	5.4
COILP2	coilin pseudogene 2	pseudogene	.	5.4
FENDRR	FOXF1 adjacent non-coding developmental regula	lncRNA	.	5.4
SETDB2-PHF11	SETDB2-PHF11 readthrough	protein_coding	NP_001307656.1	5.4
LOC105369487	uncharacterized LOC105369487	lncRNA	.	5.4
ATP6V1G2-DDX39B	ATP6V1G2-DDX39B readthrough (NMD candida	lncRNA	.	5.4
SLCO1B7	solute carrier organic anion transporter family mer	protein_coding	NP_001009562.3	5.4
GAPDHP14	glyceraldehyde-3-phosphate dehydrogenase pseud	pseudogene	.	5.4
LOC105377275	uncharacterized LOC105377275, transcript varian	lncRNA	.	5.4
SNORD56B	small nucleolar RNA, C/D box 56B	snoRNA	.	5.4
LOC105372470	uncharacterized LOC105372470	lncRNA	.	5.4
EREG	epiregulin	protein_coding	NP_001423.1	5.4
LINC00571	long intergenic non-protein coding RNA 571	lncRNA	.	5.4
NFKBIZ	NFKB inhibitor zeta	protein_coding	NP_001005474.1;NP_113607.1	5.4
GPATCH4	G-patch domain containing 4	protein_coding	NP_056405.2;NP_872620.1;XP_00	5.4
MIR1256	microRNA 1256	miRNA	.	5.4
LOC105376374	uncharacterized LOC105376374	lncRNA	.	5.4
LOC100505622	uncharacterized LOC100505622	lncRNA	.	5.4
LOC105377732	uncharacterized LOC105377732, transcript varian	lncRNA	.	5.4
RPL5P4	ribosomal protein L5 pseudogene 4	pseudogene	.	5.4
LOC105374352	uncharacterized LOC105374352	lncRNA	.	5.4
SLC15A2	solute carrier family 15 member 2	protein_coding	NP_001139470.1;NP_066568.3;XP_	5.4
LOC107986161	uncharacterized LOC107986161	lncRNA	.	5.3

SETP2	SET pseudogene 2	pseudogene	.	5.3
LOC101928236	uncharacterized LOC101928236, transcript varian	lncRNA	.	5.3
PPP4R1L	protein phosphatase 4 regulatory subunit 1 like (ps	ranscribed_pseudogen	.	5.3
LOC105375751	uncharacterized LOC105375751, transcript varian	lncRNA	.	5.3
TFEC	transcription factor EC	protein_coding	NP_001018068.1;NP_001231512.1;	5.3
MIR1207	microRNA 1207	miRNA	.	5.3
MIR4644	microRNA 4644	miRNA	.	5.3
TAS2R15P	taste 2 receptor member 15 pseudogene	pseudogene	.	5.3
TMCC3	transmembrane and coiled-coil domain family 3	protein_coding	NP_001287965.1;NP_065749.3	5.3
ATP6AP1L	ATPase H+ transporting accessory protein 1 like	protein_coding	NP_001017971.1;NP_001336300.1;	5.3
LOC101059954	uncharacterized LOC101059954	lncRNA	.	5.3
LOC107984865	uncharacterized LOC107984865	lncRNA	.	5.3
LOC101928041	.	pseudogene	.	5.3
ST13P22	ST13, Hsp70 interacting protein pseudogene 22	pseudogene	.	5.3
IFIH1	interferon induced with helicase C domain 1	protein_coding	NP_071451.2	5.3
HNRNPA1P53	heterogeneous nuclear ribonucleoprotein A1 pseuc	pseudogene	.	5.3
CCDC18-AS1	CCDC18 antisense RNA 1	lncRNA	.	5.3
NPPA-AS1	NPPA antisense RNA 1	antisense_RNA	.	5.3
CFAP69	cilia and flagella associated protein 69	protein_coding	NP_001034795.2;NP_001153610.1;	5.3
MCUR1P1	MCUR1 pseudogene 1	pseudogene	.	5.3
LURAP1L-AS1	LURAP1L antisense RNA 1	lncRNA	.	5.3
LOC105374181	uncharacterized LOC105374181, transcript varian	lncRNA	.	5.3
DNAH5	dynein axonemal heavy chain 5	protein_coding	NP_001360.1;XP_005248319.2;XP_	5.3
PDXDC2P-NPIPB14F	nuclear pore complex-interacting protein	ranscribed_pseudogen	.	5.3
LINC01204	long intergenic non-protein coding RNA 1204	lncRNA	.	5.3
CEP152	centrosomal protein 152	protein_coding	NP_001181927.1;NP_055800.2;XP_	5.2
MIR548L	microRNA 548l	miRNA	.	5.2
LPXN	leupaxin	protein_coding	NP_001137467.1;NP_001294880.1;	5.2
LOC105375634	uncharacterized LOC105375634, transcript varian	lncRNA	.	5.2
LOC105377272	uncharacterized LOC105377272	lncRNA	.	5.2
ABI3BP	ABI family member 3 binding protein	protein_coding	NP_001336258.2;NP_001336259.2;	5.2
LOC105373890	uncharacterized LOC105373890, transcript varian	lncRNA	.	5.2
LOC107986114	uncharacterized LOC107986114, transcript varian	lncRNA	.	5.2
LOC101927040	uncharacterized LOC101927040	lncRNA	.	5.2
LINC01359	long intergenic non-protein coding RNA 1359	lncRNA	.	5.2
MTVR2	mouse mammary tumor virus receptor homolog 2	ranscribed_pseudogen	.	5.2
NBPF12	NBPF member 12	protein_coding	NP_001265070.1;XP_024309211.1	5.2
LOC105372098	uncharacterized LOC105372098, transcript varian	lncRNA	.	5.2
LOC101929174	uncharacterized LOC101929174, transcript varian	lncRNA	.	5.2
PSG7	pregnancy specific beta-1-glycoprotein 7	protein_coding	NP_001193579.1;NP_002774.2	5.2
LOC107985354	uncharacterized LOC107985354	lncRNA	.	5.2
LOC105370500	uncharacterized LOC105370500, transcript varian	lncRNA	.	5.2
HS3ST3A1	heparan sulfate-glucosamine 3-sulfotransferase 3A	protein_coding	NP_006033.1;XP_011522416.1;XP_	5.2
FAM247D	family with sequence similarity 247 member D	lncRNA	.	5.2
FAM13A-AS1	FAM13A antisense RNA 1	lncRNA	.	5.2
LOC100421561	family with sequence similarity 133 member B ps	pseudogene	.	5.2
LOC107986101	.	pseudogene	.	5.2
ELF2P1	ELF2 pseudogene 1	pseudogene	.	5.2
UBA52P6	ubiquitin A-52 residue ribosomal protein fusion pr	pseudogene	.	5.2
LOC105373117	uncharacterized LOC105373117	lncRNA	.	5.2
LOC107986904	uncharacterized LOC107986904	lncRNA	.	5.2
LOC107984713	uncharacterized LOC107984713	lncRNA	.	5.2
LOC105375500	uncharacterized LOC105375500	lncRNA	.	5.2
LOC105376367	uncharacterized LOC105376367, transcript varian	lncRNA	.	5.1
MIR6733	hsa-miR-6733-3p	.	.	5.1
LOC107986069	uncharacterized LOC107986069	lncRNA	.	5.1
PRR13P2	proline rich 13 pseudogene 2	pseudogene	.	5.1
TXLNGY	taxilin gamma pseudogene, Y-linked	ranscribed_pseudogen	.	5.1
SNORD173	small nucleolar RNA, C/D box 173	snoRNA	.	5.1
CDC27P10	cell division cycle 27 pseudogene 10	ranscribed_pseudogen	.	5.1
RPS12P22	ribosomal protein S12 pseudogene 22	pseudogene	.	5.1
ADAMTS6	ADAM metallopeptidase with thrombospondin typ	protein_coding	NP_922932.2;XP_011541415.1;XP_	5.1
MIR616	microRNA 616	miRNA	.	5.1
SLCO1A2	solute carrier organic anion transporter family mer	protein_coding	NP_066580.1;NP_602307.1;XP_00	5.1
ACSL5	acyl-CoA synthetase long chain family member 5	protein_coding	NP_057318.2;NP_976313.1;NP_97	5.1
RGS4	regulator of G protein signaling 4	protein_coding	NP_001095915.1;NP_001106851.1;	5.1
LINC01322	long intergenic non-protein coding RNA 1322	lncRNA	.	5.1
VDAC1P8	voltage dependent anion channel 1 pseudogene 8	pseudogene	.	5.1
MAGI2	membrane associated guanylate kinase, WW and I	protein_coding	NP_001288057.1;NP_036433.2;XP_	5.1
SCYL2P1	SCYL2 pseudogene 1	pseudogene	.	5.1
PSG1	pregnancy specific beta-1-glycoprotein 1	protein_coding	NP_001171754.1;NP_001171755.1;	5.1

RPS15AP10	ribosomal protein S15a pseudogene 10	ranscribed_pseudogen	.	5.1
DIRC3-AS1	DIRC3 antisense RNA 1	lncRNA	.	5.1
LINC01583	long intergenic non-protein coding RNA 1583	lncRNA	.	5.1
LOC101929162	uncharacterized LOC101929162	lncRNA	.	5.1
PPM1AP1	protein phosphatase, Mg2+/Mn2+ dependent 1A p	pseudogene	.	5.1
CDC27P9	cell division cycle 27 pseudogene 9	ranscribed_pseudogen	.	5.1
LOC107985679	uncharacterized LOC107985679	lncRNA	.	5.1
WDR19	WD repeat domain 19	protein_coding	NP_001304853.1;NP_079408.3;XP_	5.1
HIVEP2	HIVEP zinc finger 2	protein_coding	NP_006725.3;XP_016866294.1;XP_	5.0
RCC2P7	regulator of chromosome condensation 2 pseudog	pseudogene	.	5.0
BCL2L1-AS1	BCL2L1 antisense RNA 1	lncRNA	.	5.0
PNISR	PNN interacting serine and arginine rich protein	protein_coding	NP_001309334.1;NP_001309335.1;	5.0
RANP3	RAN pseudogene 3	pseudogene	.	5.0
HP09053	uncharacterized LOC101929357	lncRNA	.	5.0
LOC105378698	uncharacterized LOC105378698	lncRNA	.	5.0
LOC107986010	uncharacterized LOC107986010	lncRNA	.	5.0
TLR8-AS1	TLR8 antisense RNA 1	lncRNA	.	5.0
ARHGAP25	Rho GTPase activating protein 25	protein_coding	NP_001007232.2;NP_001159748.1;	5.0
AHSA2P	activator of HSP90 ATPase homolog 2, pseudoger	ranscribed_pseudogen	.	5.0
LOC107987182	uncharacterized LOC107987182	lncRNA	.	5.0
LOC112268100	uncharacterized LOC112268100	lncRNA	.	5.0
SNORD136	small nucleolar RNA, C/D box 136	snoRNA	.	5.0
SNORA9	small nucleolar RNA, H/ACA box 9	snoRNA	.	5.0
LOC101930307	uncharacterized LOC101930307, transcript varian	protein_coding	XP_016870904.1;XP_016870906.1;	5.0
TXK	TXK tyrosine kinase	protein_coding	NP_003319.2;XP_011512049.1;XP_	5.0
LOC751603	tropomyosin 3 pseudogene	pseudogene	.	5.0
POU5F2	POU domain class 5, transcription factor 2	protein_coding	NP_694948.1	5.0
LOC105370849	golgin subfamily A member 2-like, transcript vari	protein_coding	XP_011520597.1;XP_016878276.1;	5.0
RSAD2	radical S-adenosyl methionine domain containing	protein_coding	NP_542388.2;XP_011508717.1	5.0
LOC112268051	uncharacterized LOC112268051, transcript varian	lncRNA	.	5.0
OR4F16	olfactory receptor family 4 subfamily F member 1	protein_coding	NP_001005277.1;XP_016857897.1;	5.0
PGM5P2	phosphoglucomutase 5 pseudogene 2	ranscribed_pseudogen	.	5.0
ETV1	ETS variant transcription factor 1	protein_coding	NP_001156619.1;NP_001156620.1;	5.0
MIR181A1HG	MIR181A1 host gene	lncRNA	.	5.0
PLAC8L1	PLAC8 like 1	protein_coding	NP_001025040.1;XP_005268438.1;	5.0
LOC101929269	uncharacterized LOC101929269	lncRNA	.	5.0
OVCH1	ovochymase 1	protein_coding	NP_001340108.1;XP_011518940.1;	5.0
LOC647253	ring finger protein 13 pseudogene	pseudogene	.	5.0
AGAP4	ArfGAP with GTPase domain, ankyrin repeat and	protein_coding	NP_001263272.2;NP_001278308.1;	5.0
LOC107985027	uncharacterized LOC107985027, transcript varian	lncRNA	.	4.9
PEG13	paternally expressed 13	lncRNA	.	4.9
LOC105378988	uncharacterized LOC105378988	lncRNA	.	4.9
SMC5-AS1	SMC5 antisense RNA 1 (head to head)	lncRNA	.	4.9
LOC107986273	uncharacterized LOC107986273	lncRNA	.	4.9
MIR151A	microRNA 151a	miRNA	.	4.9
LOC112268282	uncharacterized LOC112268282	lncRNA	.	4.9
CAPNS2	calpain small subunit 2	protein_coding	NP_115706.1	4.9
LOC107984138	serine/threonine-protein kinase SMG1-like, transc	protein_coding	XP_016879454.1;XP_016879455.1	4.9
SDCCAG8	SHH signaling and ciliogenesis regulator SDCCA	protein_coding	NP_001337175.1;NP_001337176.1;	4.9
LOC101927978	uncharacterized LOC101927978, transcript varian	lncRNA	.	4.9
CMYA5	cardiomyopathy associated 5	protein_coding	NP_705838.3;XP_016864701.1	4.9
LOC105376033	uncharacterized LOC105376033	lncRNA	.	4.9
LOC105371998	uncharacterized LOC105371998	lncRNA	.	4.9
LOC107984256	uncharacterized LOC107984256	lncRNA	.	4.9
LOC107986813	uncharacterized LOC107986813	lncRNA	.	4.9
CLEC4A	C-type lectin domain family 4 member A	protein_coding	NP_057268.1;NP_919429.2;NP_91	4.9
MIR4435-1	microRNA 4435-1	miRNA	.	4.9
LOC100129774	ATPase family AAA domain containing 1 pseudo	pseudogene	.	4.9
LOC107985726	uncharacterized LOC107985726	lncRNA	.	4.9
PRKAR1AP1	protein kinase cAMP-dependent type I regulatory	pseudogene	.	4.9
LOC105374894	uncharacterized LOC105374894, transcript varian	lncRNA	.	4.9
DISC1-IT1	DISC1 intronic transcript 1	lncRNA	.	4.9
LOC101926959	uncharacterized LOC101926959	lncRNA	.	4.8
GAS2	growth arrest specific 2	protein_coding	NP_001137302.1;NP_001338153.1;	4.8
ADH5P3	ADH5 pseudogene 3	pseudogene	.	4.8
LOC112267938	uncharacterized LOC112267938	lncRNA	.	4.8
UCN2	urocortin 2	protein_coding	NP_149976.1	4.8
PDE8A	phosphodiesterase 8A	protein_coding	NP_001230066.1;NP_002596.1;NP_	4.8
LOC101929200	uncharacterized LOC101929200, transcript varian	lncRNA	.	4.8
GUSBP4	GUSB pseudogene 4	ranscribed_pseudogen	.	4.8
LOC107984694	.	pseudogene	.	4.8

EEF1A1P19	eukaryotic translation elongation factor 1 alpha 1 p	pseudogene	.	4.8
ZNF705E	zinc finger protein 705E	protein_coding	NP_001265642.1	4.8
RPL36AP16	ribosomal protein L36a pseudogene 16	pseudogene	.	4.8
UTS2B	urotensin 2B	protein_coding	NP_937795.2;XP_011510933.1;XP_	4.8
UPF3AP3	UPF3A pseudogene 3	pseudogene	.	4.8
AMT	aminomethyltransferase	protein_coding	NP_000472.2;NP_001158182.1;NP_	4.8
LOC112268108	uncharacterized LOC112268108	lncRNA	.	4.8
NAIPP4	NAIP pseudogene 4	pseudogene	.	4.8
NOTCH2NLB	notch 2 N-terminal like B	protein_coding	NP_001350936.1;NP_001350937.1;	4.8
LOC101929141	coiled-coil domain-containing protein 144A-like	ranscribed_pseudogen	.	4.8
RPL17P50	ribosomal protein L17 pseudogene 50	pseudogene	.	4.8
TMEM161B-AS1	TMEM161B antisense RNA 1	lncRNA	.	4.8
SNORD132	small nucleolar RNA, C/D box 132	snoRNA	.	4.8
USP3-AS1	USP3 antisense RNA 1	lncRNA	.	4.8
FRMD3	FERM domain containing 3	protein_coding	NP_001231888.1;NP_001231889.1;	4.7
LINC00222	long intergenic non-protein coding RNA 222	lncRNA	.	4.7
MIR3679	microRNA 3679	miRNA	.	4.7
GOLGB1	golgin B1	protein_coding	NP_001243415.1;NP_001243416.1;	4.7
IFIT1P1	interferon induced protein with tetratricopeptide re	pseudogene	.	4.7
DHRS3	dehydrogenase/reductase 3	protein_coding	NP_001306154.1;NP_001311299.1;	4.7
LOC107985225	uncharacterized LOC107985225	lncRNA	.	4.7
CCDC144B	coiled-coil domain containing 144B (pseudogene)	ranscribed_pseudogen	.	4.7
REC8	REC8 meiotic recombination protein	protein_coding	NP_001041670.1;NP_005123.2;XP_	4.7
ST7-OT4	ST7 overlapping transcript 4	lncRNA	.	4.7
ACTG1P3	actin gamma 1 pseudogene 3	pseudogene	.	4.7
SH3BP5-AS1	SH3BP5 antisense RNA 1	lncRNA	.	4.7
LSMEM1	leucine rich single-pass membrane protein 1	protein_coding	NP_001127940.1;NP_872403.1;XP_	4.7
RPS29P18	ribosomal protein S29 pseudogene 18	pseudogene	.	4.7
FCRLA	Fc receptor like A	protein_coding	NP_001171795.2;NP_001171796.2;	4.7
PLA2G4C	phospholipase A2 group IVC	protein_coding	NP_001152794.1;NP_001152795.1;	4.7
HIGD1AP16	HIG1 hypoxia inducible domain family member 1	pseudogene	.	4.7
GCM1	glial cells missing transcription factor 1	protein_coding	NP_003634.2;XP_016866879.1	4.7
SNORD57	small nucleolar RNA, C/D box 57	snoRNA	.	4.7
LOC112268035	uncharacterized LOC112268035	lncRNA	.	4.7
LOC107985369	uncharacterized LOC107985369	lncRNA	.	4.7
LOC100133252	zinc finger protein 131 pseudogene	pseudogene	.	4.7
ABCA5	ATP binding cassette subfamily A member 5	protein_coding	NP_061142.2;NP_758424.1	4.7
ATP6	ATP synthase F0 subunit 6	protein_coding	YP_003024031.1	4.7
HNRNPA1P5	heterogeneous nuclear ribonucleoprotein A1 pseuc	pseudogene	.	4.7
LOC107986698	uncharacterized LOC107986698	lncRNA	.	4.7
AGAP12P	ArfGAP with GTPase domain, ankyrin repeat and	ranscribed_pseudogen	.	4.7
SHC4	SHC adaptor protein 4	protein_coding	NP_976224.3;XP_005254432.1	4.7
SOCS5P4	suppressor of cytokine signaling 5 pseudogene 4	pseudogene	.	4.7
POLR3DP1	RNA polymerase III subunit D pseudogene 1	pseudogene	.	4.7
MIATNB	MIAT neighbor	lncRNA	.	4.7
LOC107986020	uncharacterized LOC107986020	lncRNA	.	4.6
NSRP1P1	nuclear speckle splicing regulatory protein 1 pseuc	pseudogene	.	4.6
LOC105374127	uncharacterized LOC105374127	lncRNA	.	4.6
SCML1	Scm polycomb group protein like 1	protein_coding	NP_001032624.1;NP_001032625.1;	4.6
RPL21P48	ribosomal protein L21 pseudogene 48	pseudogene	.	4.6
LOC101928705	uncharacterized LOC101928705, transcript varian	lncRNA	.	4.6
SCARNA27	small Cajal body-specific RNA 27	guide_RNA	.	4.6
LOC112267910	NACHT, LRR and PYD domains-containing prote	protein_coding	XP_016885504.1	4.6
SOHLH2	spermatogenesis and oogenesis specific basic heli	protein_coding	NP_001269076.1;NP_060296.2	4.6
LRRC69	leucine rich repeat containing 69	protein_coding	NP_001123362.1;NP_001341399.1	4.6
GPRASP1	G protein-coupled receptor associated sorting prot	protein_coding	NP_001092880.1;NP_001092881.1;	4.6
RPL36A	ribosomal protein L36a	protein_coding	NP_066357.3	4.6
MIR4803	microRNA 4803	miRNA	.	4.6
LOC105376208	uncharacterized LOC105376208, transcript varian	lncRNA	.	4.6
TVP23CP2	TVP23C pseudogene 2	pseudogene	.	4.6
LOC100420050	DNA replication fork stabilization factor DONSO	pseudogene	.	4.6
TPM3P7	tropomyosin 3 pseudogene 7	pseudogene	.	4.6
LOC105374571	uncharacterized LOC105374571	lncRNA	.	4.6
LOC101927366	uncharacterized LOC101927366	lncRNA	.	4.6
LOC100422296	Parkinsonism associated deglycase pseudogene	pseudogene	.	4.6
TAS2R5	taste 2 receptor member 5	protein_coding	NP_061853.1	4.6
LOC112268450	uncharacterized LOC112268450	lncRNA	.	4.6
RNU6-32P	RNA, U6 small nuclear 32, pseudogene	pseudogene	.	4.6
TARID	TCF21 antisense RNA inducing promoter demeth	lncRNA	.	4.6
LOC107986779	uncharacterized LOC107986779	lncRNA	.	4.6
C2orf74	chromosome 2 open reading frame 74	protein_coding	NP_001137431.1;NP_001137432.1;	4.6

TFB1M	transcription factor B1, mitochondrial	protein_coding	NP_001337430.1;NP_001337431.1;	4.6
LOC392555	MAGE family member C2 pseudogene	pseudogene	.	4.6
FXNP2	frataxin pseudogene 2	pseudogene	.	4.6
FAP	fibroblast activation protein alpha	protein_coding	NP_001278736.1;NP_004451.2;XP_	4.6
LOC105377735	uncharacterized LOC105377735	lncRNA	.	4.6
LOC105370664	uncharacterized LOC105370664	lncRNA	.	4.6
DDX50P1	DEAD-box helicase 50 pseudogene 1	pseudogene	.	4.6
KCTD4	potassium channel tetramerization domain contain	protein_coding	NP_940686.2	4.6
KLF9-DT	KLF9 divergent transcript, transcript variant X7	lncRNA	.	4.6
SNORD56	small nucleolar RNA, C/D box 56	snoRNA	.	4.5
SKP1P1	S-phase kinase associated protein 1 pseudogene 1	pseudogene	.	4.5
LOC100131465	peroxisomal biogenesis factor 5 pseudogene	pseudogene	.	4.5
MUC19	mucin 19, oligomeric	protein_coding	NP_775871.2	4.5
DNAH3	dynein axonemal heavy chain 3	protein_coding	NP_001334815.1;NP_060009.1;XP_	4.5
SNORA30	small nucleolar RNA, H/ACA box 30	snoRNA	.	4.5
SNORD76	small nucleolar RNA, C/D box 76	snoRNA	.	4.5
PRH1-PRR4	PRH1-PRR4 readthrough	lncRNA	.	4.5
LOC105373893	uncharacterized LOC105373893, transcript variant	lncRNA	.	4.5
ELOCP19	elongin C pseudogene 19	pseudogene	.	4.5
LRP1B	LDL receptor related protein 1B	protein_coding	NP_061027.2;XP_016859830.1;XP_	4.5
SMG1P7	SMG1 pseudogene 7	ranscribed_pseudogen	.	4.5
LDHAP7	lactate dehydrogenase A pseudogene 7	pseudogene	.	4.5
RPL36AP37	ribosomal protein L36a pseudogene 37	pseudogene	.	4.5
SYNDIG1L	synapse differentiation inducing 1 like	protein_coding	NP_001099049.1;XP_016877089.1	4.5
ULK4	unc-51 like kinase 4	protein_coding	NP_001309429.1;NP_001309430.1;	4.5
RPL36AL	ribosomal protein L36a like	protein_coding	NP_000992.1	4.5
YWHAEP5	tyrosine 3-monooxygenase/tryptophan 5-monooxy	pseudogene	.	4.5
HMGB1P21	high mobility group box 1 pseudogene 21	pseudogene	.	4.5
LOC105370327	uncharacterized LOC105370327, transcript variant	lncRNA	.	4.5
CHSY3	chondroitin sulfate synthase 3	protein_coding	NP_787052.3;XP_005272039.1;XP_	4.5
BMS1P7	BMS1 pseudogene 7	pseudogene	.	4.5
SCARNA9	small Cajal body-specific RNA 9	guide_RNA	.	4.5
LOC100507477	uncharacterized LOC100507477	lncRNA	.	4.5
SNORD69	small nucleolar RNA, C/D box 69	snoRNA	.	4.5
PITRM1-AS1	PITRM1 antisense RNA 1	lncRNA	.	4.5
LOC112268150	uncharacterized LOC112268150	lncRNA	.	4.5
HSPA5	heat shock protein family A (Hsp70) member 5	protein_coding	NP_005338.1	4.5
MYPN	myopalladin	protein_coding	NP_001243196.1;NP_001243197.1;	4.5
SNRK-AS1	SNRK antisense RNA 1	lncRNA	.	4.5
CDC27P11	cell division cycle 27 pseudogene 11	ranscribed_pseudogen	.	4.5
HCG18	HLA complex group 18	lncRNA	.	4.5
HMGN1P24	high mobility group nucleosome binding domain 1	pseudogene	.	4.5
LOC105369791	uncharacterized LOC105369791, transcript variant	lncRNA	.	4.5
LINC00184	long intergenic non-protein coding RNA 184	lncRNA	.	4.5
LOC112268031	.	pseudogene	.	4.5
BTN3A1	butyrophilin subfamily 3 member A1	protein_coding	NP_001138480.1;NP_001138481.1;	4.5
LOC101929018	uncharacterized LOC101929018	lncRNA	.	4.5
LOC105374709	uncharacterized LOC105374709, transcript variant	lncRNA	.	4.5
STMN1P1	stathmin 1 pseudogene 1	pseudogene	.	4.5
LINC00470	long intergenic non-protein coding RNA 470	lncRNA	.	4.5
LINC01881	long intergenic non-protein coding RNA 1881	lncRNA	.	4.5
MIR4435-2	microRNA 4435-2	miRNA	.	4.5
LOC100506071	uncharacterized LOC100506071	lncRNA	.	4.5
LOC105369574	uncharacterized LOC105369574, transcript variant	lncRNA	.	4.5
FAM106B	family with sequence similarity 106 member B	protein_coding	NP_001335090.1	4.5
RPSAP26	ribosomal protein SA pseudogene 26	pseudogene	.	4.5
LOC101928152	uncharacterized LOC101928152, transcript variant	lncRNA	.	4.5
LOC102723759	uncharacterized LOC102723759, transcript variant	lncRNA	.	4.5
GOLGA6L9	golgin A6 family like 9	protein_coding	NP_001278349.1;NP_937824.3;XP_	4.5
MIR6079	microRNA 6079	miRNA	.	4.5
RPL23AP74	ribosomal protein L23a pseudogene 74	pseudogene	.	4.5
PCDHGB8P	protocadherin gamma subfamily B, 8 pseudogene	ranscribed_pseudogen	.	4.4
ND4	NADH dehydrogenase subunit 4	protein_coding	YP_003024035.1	4.4
CCDC17	coiled-coil domain containing 17	protein_coding	NP_001108410.2;NP_001177111.1;	4.4
DRP2	dystrophin related protein 2	protein_coding	NP_001164655.1;NP_001930.2;XP_	4.4
LOC101927488	uncharacterized LOC101927488	lncRNA	.	4.4
LOC105378410	uncharacterized LOC105378410, transcript variant	lncRNA	.	4.4
LOC107986346	.	pseudogene	.	4.4
LOC102929163	ADP ribosylation factor like GTPase 17A pseudog	pseudogene	.	4.4
LOC107986187	uncharacterized LOC107986187	lncRNA	.	4.4
SNORD58A	small nucleolar RNA, C/D box 58A	snoRNA	.	4.4

LOC107987183	uncharacterized LOC107987183	lncRNA	.	4.4
LOC105369568	uncharacterized LOC105369568, transcript varian	lncRNA	.	4.4
SNORD103A	small nucleolar RNA, C/D box 103A	snoRNA	.	4.4
LINC01411	long intergenic non-protein coding RNA 1411	lncRNA	.	4.4
ND5	NADH dehydrogenase subunit 5	protein_coding	YP_003024036.1	4.4
LOC101929710	uncharacterized LOC101929710	lncRNA	.	4.4
EEF1DP2	eukaryotic translation elongation factor 1 delta pse	pseudogene	.	4.4
NPIPA3	nuclear pore complex interacting protein family m	protein_coding	NP_001264252.1;XP_005255542.1;	4.4
LOC105370965	uncharacterized LOC105370965	lncRNA	.	4.4
SNORD67	small nucleolar RNA, C/D box 67	snoRNA	.	4.4
MYH3	myosin heavy chain 3	protein_coding	NP_002461.2;XP_011522172.1;XP_	4.4
LINC01929	long intergenic non-protein coding RNA 1929	lncRNA	.	4.4
LOC100506124	uncharacterized LOC100506124	lncRNA	.	4.4
LOC101929165	uncharacterized LOC101929165	lncRNA	.	4.4
NRP2	neuropilin 2	protein_coding	NP_003863.2;NP_061004.3;NP_95	4.4
GDNF-AS1	GDNF antisense RNA 1	lncRNA	.	4.4
LINC00342	long intergenic non-protein coding RNA 342	lncRNA	.	4.4
TRIML2	tripartite motif family like 2	protein_coding	NP_001290348.1;NP_775824.2;XP_	4.4
LOC148430	ribosomal protein S2 pseudogene	pseudogene	.	4.4
PLGLB1	plasminogen like B1	protein_coding	NP_001027564.1	4.3
DCAF13P3	DDB1 and CUL4 associated factor 13 pseudogene	ranscribed_pseudogen	.	4.3
KCNQ1OT1	KCNQ1 opposite strand/antisense transcript 1	lncRNA	.	4.3
ADAMTS12	ADAM metallopeptidase with thrombospondin ty	protein_coding	NP_001311440.1;NP_001311441.1;	4.3
AKAP9	A-kinase anchoring protein 9	protein_coding	NP_001366206.1;NP_005742.4;NP_	4.3
NAMPTP1	nicotinamide phosphoribosyltransferase pseudoge	pseudogene	.	4.3
HMGN1P28	high mobility group nucleosome binding domain 1	pseudogene	.	4.3
RN7SL608P	RNA, 7SL, cytoplasmic 608, pseudogene	pseudogene	.	4.3
ANKHD1	ankyrin repeat and KH domain containing 1	protein_coding	NP_001183959.1;NP_060217.1;NP_	4.3
ANAPC1P4	ANAPC1 pseudogene 4	ranscribed_pseudogen	.	4.3
POLR2KP1	RNA polymerase II subunit K pseudogene 1	pseudogene	.	4.3
LOC105373265	uncharacterized LOC105373265, transcript varian	lncRNA	.	4.3
HERC6	HECT and RLD domain containing E3 ubiquitin p	protein_coding	NP_001158608.1;NP_060382.3;XP_	4.3
LOC107986018	uncharacterized LOC107986018	lncRNA	.	4.3
LOC105371841	uncharacterized LOC105371841	lncRNA	.	4.3
LOC101927506	uncharacterized LOC101927506	lncRNA	.	4.3
LINC01409	long intergenic non-protein coding RNA 1409, tra	lncRNA	.	4.3
LOC107985156	uncharacterized LOC107985156	lncRNA	.	4.3
GLYATL1	glycine-N-acyltransferase like 1	protein_coding	NP_001207423.1;NP_001207425.1;	4.3
LINC00894	long intergenic non-protein coding RNA 894	lncRNA	.	4.3
RNF32-AS1	RNF32 antisense RNA 1	lncRNA	.	4.3
LOC100420981	nucleosome assembly protein 1 like 1 pseudogene	pseudogene	.	4.3
PSG8	pregnancy specific beta-1-glycoprotein 8	protein_coding	NP_001123639.1;NP_001123640.1;	4.3
LOC105374162	uncharacterized LOC105374162	lncRNA	.	4.3
LDB2	LIM domain binding 2	protein_coding	NP_001124306.1;NP_001281.1;NP_	4.3
SNORD63B	small nucleolar RNA, C/D box 63B	snoRNA	.	4.3
MARCHF11-AS1	MARCHF11 antisense RNA 1	lncRNA	.	4.3
CCDC191	coiled-coil domain containing 191	protein_coding	NP_001340695.1;NP_001340696.2;	4.3
LOC107985840	uncharacterized LOC107985840, transcript varian	lncRNA	.	4.3
CHORDC1P4	CHORDC1 pseudogene 4	ranscribed_pseudogen	.	4.3
MINAR1	membrane integral NOTCH2 associated receptor 1	protein_coding	NP_056021.1;XP_011519694.1;XP_	4.3
RALGAPA1P1	RALGAPA1 pseudogene 1	ranscribed_pseudogen	.	4.3
MIR942	microRNA 942	miRNA	.	4.3
TXN2P1	TXN2 pseudogene 1	pseudogene	.	4.3
LOC107984318	uncharacterized LOC107984318	lncRNA	.	4.3
LOC105375213	uncharacterized LOC105375213	lncRNA	.	4.3
LOC100506023	uncharacterized LOC100506023	lncRNA	.	4.3
NPIPA1	nuclear pore complex interacting protein family m	protein_coding	NP_008916.2	4.3
LOC105376626	uncharacterized LOC105376626, transcript varian	lncRNA	.	4.3
TRANK1	tetratricopeptide repeat and ankyrin repeat contain	protein_coding	NP_001316927.1;NP_055646.2;XP_	4.3
MTAPP2	methylthioadenosine phosphorylase pseudogene 2	pseudogene	.	4.3
BMS1P4-AGAP5	BMS1P4-AGAP5 readthough	ranscribed_pseudogen	.	4.3
LOC374443	C-type lectin domain family 2 member D pseudog	ranscribed_pseudogen	.	4.3
NPIPA2	nuclear pore complex interacting protein family m	protein_coding	NP_001264253.1;XP_005255549.1;	4.3
RPL7L1P6	ribosomal protein L7 like 1 pseudogene 6	pseudogene	.	4.3
LOC105377567	uncharacterized LOC105377567, transcript varian	lncRNA	.	4.3
LOC105371131	uncharacterized LOC105371131, transcript varian	lncRNA	.	4.3
TF	transferrin	protein_coding	NP_001054.2;NP_001341632.2;NP_	4.2
ANP32C	acidic nuclear phosphoprotein 32 family member	protein_coding	NP_036535.1	4.2
LOC105370322	uncharacterized LOC105370322	lncRNA	.	4.2
PTRH2	peptidyl-tRNA hydrolase 2	protein_coding	NP_001015509.1;NP_057161.1;XP_	4.2
BOD1L1	biorientation of chromosomes in cell division 1 lik	protein_coding	NP_683692.2;XP_005248207.1;XP_	4.2

MIR4774	microRNA 4774	miRNA	.	4.2
MIR3605	microRNA 3605	miRNA	.	4.2
RN7SKP185	RN7SK pseudogene 185	pseudogene	.	4.2
ZNF471	zinc finger protein 471	protein_coding	NP_001308697.1;NP_065864.2;XP_	4.2
NEDD4L	NEDD4 like E3 ubiquitin protein ligase	protein_coding	NP_001138436.1;NP_001138437.1;	4.2
RN7SL49P	RNA, 7SL, cytoplasmic 49, pseudogene	pseudogene	.	4.2
LOC105369958	uncharacterized LOC105369958, transcript varian	lncRNA	.	4.2
CD177	CD177 molecule	protein_coding	NP_065139.2;XP_016882510.1;XP_	4.2
LOC105369957	uncharacterized LOC105369957, transcript varian	lncRNA	.	4.2
TMEM178A	transmembrane protein 178A	protein_coding	NP_001161431.1;NP_689603.2;XP_	4.2
ATG12P1	autophagy related 12 pseudogene 1	pseudogene	.	4.2
LOC107986422	uncharacterized LOC107986422	lncRNA	.	4.2
LOC107984234	uncharacterized LOC107984234, transcript varian	lncRNA	.	4.2
VENTXP1	VENT homeobox pseudogene 1	ranscribed_pseudogen	.	4.2
PIN4	peptidylprolyl cis/trans isomerase, NIMA-interacti	protein_coding	NP_001164218.1;NP_006214.3	4.2
FBXO32	F-box protein 32	protein_coding	NP_001229392.1;NP_478136.1;NP_	4.2
CTSS	cathepsin S	protein_coding	NP_001186668.1;NP_004070.3	4.2
LOC107986997	uncharacterized LOC107986997	lncRNA	.	4.2
MYRFL	myelin regulatory factor like	protein_coding	NP_872336.2;XP_011537354.1;XP_	4.2
CHRNA1	cholinergic receptor nicotinic alpha 1 subunit	protein_coding	NP_000070.1;NP_001034612.1;XP_	4.2
SAMSN1	SAM domain, SH3 domain and nuclear localizatio	protein_coding	NP_001243299.1;NP_001273452.1;	4.2
LOC107986426	uncharacterized LOC107986426	lncRNA	.	4.2
KIF5C-AS1	KIF5C antisense RNA 1, transcript variant X1	lncRNA	.	4.2
EIF5A2P1	eukaryotic translation initiation factor 5A2 pseudo	pseudogene	.	4.2
NCAPGP1	non-SMC condensin I complex subunit G pseudog	pseudogene	.	4.2
DPY19L1P2	DPY19L1 pseudogene 2	ranscribed_pseudogen	.	4.2
RPS6KA5	ribosomal protein S6 kinase A5	protein_coding	NP_001309156.1;NP_001309157.1;	4.2
CENPJ	centromere protein J	protein_coding	NP_060921.3;XP_011533451.1;XP_	4.2
LOC112267899	uncharacterized LOC112267899	lncRNA	.	4.2
CAMKMT	calmodulin-lysine N-methyltransferase	protein_coding	NP_079042.1;XP_011531413.1;XP_	4.2
CDRT1	CMT1A duplicated region transcript 1	protein_coding	NP_001269469.1;NP_006373.2	4.2
TEC	tec protein tyrosine kinase	protein_coding	NP_003206.2;XP_011512039.1;XP_	4.2
BAGE2	BAGE family member 2	protein_coding	NP_872288.2	4.2
MPP4	membrane palmitoylated protein 4	protein_coding	NP_149055.2;XP_016860109.1	4.2
TTC17	tetratricopeptide repeat domain 17	protein_coding	NP_001294872.1;NP_001363454.1;	4.2
LARP4P	LARP4 pseudogene	pseudogene	.	4.2
LOC107987245	uncharacterized LOC107987245	lncRNA	.	4.2
LOC107986278	.	pseudogene	.	4.1
LOC105373891	uncharacterized LOC105373891, transcript varian	lncRNA	.	4.1
LOC105373774	uncharacterized LOC105373774	lncRNA	.	4.1
CCND1	cyclin D1	protein_coding	NP_444284.1	4.1
LOC100128494	uncharacterized LOC100128494	lncRNA	.	4.1
MIR155HG	MIR155 host gene	lncRNA	.	4.1
LOC100129573	tRNA methyltransferase 2 homolog B (S. cerevisia	pseudogene	.	4.1
LOC105375363	uncharacterized LOC105375363, transcript varian	lncRNA	.	4.1
USP32P3	ubiquitin specific peptidase 32 pseudogene 3	ranscribed_pseudogen	.	4.1
LOC107986064	uncharacterized LOC107986064, transcript varian	lncRNA	.	4.1
SMG1P5	SMG1 pseudogene 5	ranscribed_pseudogen	.	4.1
LOC644261	solute carrier family 25 member 53 pseudogene	pseudogene	.	4.1
NAIPP2	NAIP pseudogene 2	pseudogene	.	4.1
MAPK13	mitogen-activated protein kinase 13	protein_coding	NP_002745.1;XP_024302259.1	4.1
HNRNPA1P66	heterogeneous nuclear ribonucleoprotein A1 pseuc	pseudogene	.	4.1
MYEOV	myeloma overexpressed	protein_coding	NP_001280220.1;NP_001280223.1;	4.1
LTBP1	latent transforming growth factor beta binding pro	protein_coding	NP_000618.4;NP_001159736.2;NP_	4.1
DHDDS-AS1	DHDDS antisense RNA 1	lncRNA	.	4.1
LOC100996723	.	pseudogene	.	4.1
CLIC4P1	chloride intracellular channel 4 pseudogene 1	pseudogene	.	4.1
LOC105369850	uncharacterized LOC105369850, transcript varian	lncRNA	.	4.1
LOC105375522	uncharacterized LOC105375522, transcript varian	lncRNA	.	4.1
CRADD	CASP2 and RIPK1 domain containing adaptor wit	protein_coding	NP_001307028.1;NP_001307029.1;	4.1
LOC107984494	uncharacterized LOC107984494, transcript varian	lncRNA	.	4.1
NANOGP5	Nanog homeobox pseudogene 5	pseudogene	.	4.1
HSP90AB4P	heat shock protein 90 alpha family class B membe	ranscribed_pseudogen	.	4.1
SNORD70B	small nucleolar RNA, C/D box 70B	snoRNA	.	4.1
LOC112268141	.	pseudogene	.	4.1
ZNF101P2	zinc finger protein 101 pseudogene 2	pseudogene	.	4.1
HERC2P9	hect domain and RLD 2 pseudogene 9	ranscribed_pseudogen	.	4.1
NPIPB15	nuclear pore complex interacting protein family m	protein_coding	NP_001293023.1;XP_011521798.1;	4.1
LOC105376993	uncharacterized LOC105376993, transcript varian	lncRNA	.	4.1
MIR3916	microRNA 3916	miRNA	.	4.1
PSMA6P2	proteasome subunit alpha 6 pseudogene 2	pseudogene	.	4.1

CARD16	caspase recruitment domain family member 16	protein_coding	NP_001017534.1;NP_443121.1;XP_	4.1
GARS1-DT	GARS1 divergent transcript	lncRNA	.	4.1
RPL32P16	ribosomal protein L32 pseudogene 16	pseudogene	.	4.1
LOC105376214	uncharacterized LOC105376214, transcript varian	lncRNA	.	4.1
CCDC146	coiled-coil domain containing 146	protein_coding	NP_065930.2	4.1
SRPX2	sushi repeat containing protein X-linked 2	protein_coding	NP_055282.1	4.1
HSD17B7P2	hydroxysteroid 17-beta dehydrogenase 7 pseudoge	ranscribed_pseudogene	.	4.1
ANKDD1B	ankyrin repeat and death domain containing 1B	protein_coding	NP_001263642.1;XP_011541920.1;	4.1
SNORA49	small nucleolar RNA, H/ACA box 49	snoRNA	.	4.1
TNFRSF9	TNF receptor superfamily member 9	protein_coding	NP_001552.2;XP_006710681.1	4.1
BTN3A3	butyrophilin subfamily 3 member A3	protein_coding	NP_001229732.1;NP_008925.1;NP_	4.1
RP1	RP1 axonemal microtubule associated	protein_coding	NP_001362583.1;NP_006260.1;XP_	4.1
ZNF33B	zinc finger protein 33B	protein_coding	NP_001291962.1;NP_001291964.1;	4.1
LOC112268274	uncharacterized LOC112268274, transcript varian	lncRNA	.	4.1
LOC105371493	uncharacterized LOC105371493, transcript varian	lncRNA	.	4.1
LINC00189	long intergenic non-protein coding RNA 189	lncRNA	.	4.1
LOC105374715	uncharacterized LOC105374715	lncRNA	.	4.1
OTUD4P1	OTUD4 pseudogene 1	pseudogene	.	4.0
SNORD71	small nucleolar RNA, C/D box 71	snoRNA	.	4.0
NUTM2B-AS1	NUTM2B antisense RNA 1	lncRNA	.	4.0
FANCD2P1	FANCD2 pseudogene 1	pseudogene	.	4.0
SNORA54	small nucleolar RNA, H/ACA box 54	snoRNA	.	4.0
RAB1AP1	RAB1A pseudogene 1	pseudogene	.	4.0
LOC105375334	uncharacterized LOC105375334, transcript varian	lncRNA	.	4.0
PSMC3IP	PSMC3 interacting protein	protein_coding	NP_001242943.1;NP_001242944.1;	4.0
FMN1	formin 1	protein_coding	NP_001096654.1;NP_001264242.1;	4.0
KRT8P31	keratin 8 pseudogene 31	pseudogene	.	4.0
LNCOG	lncRNA osteogenesis associated	lncRNA	.	4.0
ADHFE1	alcohol dehydrogenase iron containing 1	protein_coding	NP_653251.2	4.0
SMG1P2	SMG1 pseudogene 2	ranscribed_pseudogene	.	4.0
LOC105373888	uncharacterized LOC105373888	lncRNA	.	4.0
GOLGA4	golgin A4	protein_coding	NP_001166184.1;NP_002069.2;XP_	4.0
NAIPP1	NAIP pseudogene 1	pseudogene	.	4.0
SLC25A27	solute carrier family 25 member 27	protein_coding	NP_001190980.1;NP_001190981.1;	4.0
HDAC1P2	histone deacetylase 1 pseudogene 2	pseudogene	.	4.0
LRRC37A2	leucine rich repeat containing 37 member A2	protein_coding	NP_001006608.2;XP_011523143.1;	4.0
LOC101928451	uncharacterized LOC101928451, transcript varian	lncRNA	.	4.0
LRP2BP	LRP2 binding protein	protein_coding	NP_001364369.1;NP_001364370.1;	4.0
WFDC3	WAP four-disulfide core domain 3	protein_coding	NP_542181.1;XP_011526855.1;XP_	4.0
LOC101928421	uncharacterized LOC101928421, transcript varian	lncRNA	.	4.0
LOC105374407	uncharacterized LOC105374407	lncRNA	.	4.0
NRP1	neuropilin 1	protein_coding	NP_001019799.2;NP_001019800.2;	4.0
LOC107987115	uncharacterized LOC107987115	lncRNA	.	4.0
ZNF432	zinc finger protein 432	protein_coding	NP_001309213.1;NP_001309214.1;	4.0
LOC102723512	uncharacterized LOC102723512	lncRNA	.	4.0
LOC105372767	uncharacterized LOC105372767	lncRNA	.	4.0
MIR590	microRNA 590	miRNA	.	4.0
KLRA1P	killer cell lectin like receptor A1, pseudogene	ranscribed_pseudogene	.	4.0
LOC101241901	chromosome 4 open reading frame 46 pseudogene	pseudogene	.	4.0
DUSP6	dual specificity phosphatase 6	protein_coding	NP_001937.2;NP_073143.2	4.0
SLC16A6	solute carrier family 16 member 6	protein_coding	NP_001167637.1;NP_004685.2;XP_	4.0
LINC02542	long intergenic non-protein coding RNA 2542	lncRNA	.	4.0
LOC107985082	leucine-rich repeat-containing protein 37A3-like	protein_coding	XP_016880999.1	4.0
SNORA55	small nucleolar RNA, H/ACA box 55	snoRNA	.	4.0
SAMD12-AS1	SAMD12 antisense RNA 1	lncRNA	.	4.0
RN7SL329P	RNA, 7SL, cytoplasmic 329, pseudogene	pseudogene	.	4.0
LOC101928044	.	pseudogene	.	4.0
LOC112268148	uncharacterized LOC112268148, transcript varian	lncRNA	.	4.0
LOC100419059	tubulin epsilon 1 pseudogene	pseudogene	.	4.0
KRT8P50	keratin 8 pseudogene 50	pseudogene	.	4.0
LOC105373608	uncharacterized LOC105373608, transcript varian	lncRNA	.	4.0
SNORC	secondary ossification center associated regulator	protein_coding	NP_001333049.1;NP_001333050.1;	4.0
PARGP1	poly(ADP-ribose) glycohydrolase pseudogene 1	ranscribed_pseudogene	.	4.0
ADGRV1	adhesion G protein-coupled receptor V1	protein_coding	NP_115495.3;XP_016865452.1;XP_	4.0
MIR4677	microRNA 4677	miRNA	.	4.0
AIM2	absent in melanoma 2	protein_coding	NP_001335176.1;NP_004824.1;XP_	4.0
RPEP3	ribulose-5-phosphate-3-epimerase pseudogene 3	pseudogene	.	4.0
MIR591	microRNA 591	miRNA	.	4.0
PARP8	poly(ADP-ribose) polymerase family member 8	protein_coding	NP_001171526.1;NP_001171527.1;	4.0
LOC100422441	heat shock protein 90kDa alpha family class B me	pseudogene	.	4.0
LOC107986630	uncharacterized LOC107986630	lncRNA	.	3.9

LINC02085	long intergenic non-protein coding RNA 2085	lncRNA	.	3.9
RNU6-890P	RNA, U6 small nuclear 890, pseudogene	pseudogene	.	3.9
RAD51B	RAD51 paralog B	protein_coding	NP_001308738.1;NP_001308739.1;	3.9
LOC105377576	uncharacterized LOC105377576, transcript varian	lncRNA	.	3.9
LOC105376369	uncharacterized LOC105376369	lncRNA	.	3.9
RPGR	retinitis pigmentosa GTPase regulator	protein_coding	NP_000319.1;NP_001030025.1;NP_	3.9
CFAP45	cilia and flagella associated protein 45	protein_coding	NP_036469.2	3.9
LOC107985002	uncharacterized LOC107985002	lncRNA	.	3.9
LOC105373459	uncharacterized LOC105373459, transcript varian	lncRNA	.	3.9
CCDC171	coiled-coil domain containing 171	protein_coding	NP_001334931.1;NP_001342476.1;	3.9
GSDMB	gasdermin B	protein_coding	NP_001035936.1;NP_001159430.1;	3.9
ITPKB-IT1	ITPKB intronic transcript 1	lncRNA	.	3.9
COX2	cytochrome c oxidase subunit II	protein_coding	YP_003024029.1	3.9
NKTR	natural killer cell triggering receptor	protein_coding	NP_001336053.1;NP_001336054.1;	3.9
LOC112268301	uncharacterized LOC112268301	lncRNA	.	3.9
COQ10BP1	coenzyme Q10B pseudogene 1	pseudogene	.	3.9
LOC105373713	uncharacterized LOC105373713	lncRNA	.	3.9
MIR10526	hsa-miR-10526-3p	.	.	3.9
TUBBP10	tubulin beta class I pseudogene 10	pseudogene	.	3.9
RBM14-RBM4	RBM14-RBM4 readthrough	protein_coding	NP_001185774.1;NP_001185775.1	3.9
SNORD116-22	small nucleolar RNA, C/D box 116-22	snoRNA	.	3.9
CBLB	Cbl proto-oncogene B	protein_coding	NP_001308715.1;NP_001308717.1;	3.9
LOC100335030	FGFR1 oncogene partner 2 pseudogene	ranscribed_pseudogen	.	3.9
LOC100421438	DnaJ heat shock protein family (Hsp40) member (	pseudogene	.	3.9
LOC107987081	uncharacterized LOC107987081	lncRNA	.	3.9
SPICE1	spindle and centriole associated protein 1	protein_coding	NP_001318007.1;NP_001318008.1;	3.9
LOC105377359	uncharacterized LOC105377359, transcript varian	lncRNA	.	3.9
HSPD1P11	heat shock protein family D (Hsp60) member 1 ps	pseudogene	.	3.9
LINC01138	long intergenic non-protein coding RNA 1138	lncRNA	.	3.9
CYCSP6	CYCS pseudogene 6	pseudogene	.	3.9
GPR75	G protein-coupled receptor 75	protein_coding	NP_006785.1	3.9
PHC2-AS1	PHC2 antisense RNA 1	lncRNA	.	3.9
MIR2278	microRNA 2278	miRNA	.	3.9
LOC101926907	uncharacterized LOC101926907	lncRNA	.	3.9
MIR1296	microRNA 1296	miRNA	.	3.9
DNER	delta/notch like EGF repeat containing	protein_coding	NP_620711.3;XP_005247007.1	3.9
LAMC2	laminin subunit gamma 2	protein_coding	NP_005553.2;NP_061486.2;XP_01(	3.9
CCDC168	coiled-coil domain containing 168	protein_coding	NP_001139669.1;XP_011519408.1	3.9
SNORD116-24	small nucleolar RNA, C/D box 116-24	snoRNA	.	3.9
UBE2Q2P2	ubiquitin conjugating enzyme E2 Q2 pseudogene 2	ranscribed_pseudogen	.	3.9
LOC105373172	uncharacterized LOC105373172, transcript varian	lncRNA	.	3.9
LOC100129052	N-alpha-acetyltransferase 40, NatD catalytic subu	pseudogene	.	3.9
LOC399975	uncharacterized LOC399975	lncRNA	.	3.9
SEC14L1P1	SEC14 like 1 pseudogene 1	ranscribed_pseudogen	.	3.9
LOC105369477	uncharacterized LOC105369477, transcript varian	lncRNA	.	3.9
GJA9-MYCBP	GJA9-MYCBP readthrough	lncRNA	.	3.9
NPTN-IT1	NPTN intronic transcript 1	lncRNA	.	3.9
PLCD4	phospholipase C delta 4	protein_coding	NP_116115.1;XP_005246970.1;XP_	3.9
SNORA23	small nucleolar RNA, H/ACA box 23	snoRNA	.	3.9
HNRNPA1P77	heterogeneous nuclear ribonucleoprotein A1 pseu	pseudogene	.	3.9
RPS26P28	ribosomal protein S26 pseudogene 28	pseudogene	.	3.9
UPF3B	UPF3B regulator of nonsense mediated mRNA de	protein_coding	NP_075386.1;NP_542199.1;XP_01(	3.9
ELOVL6	ELOVL fatty acid elongase 6	protein_coding	NP_001124193.1;NP_076995.1;XP_	3.9
KCNT2	potassium sodium-activated channel subfamily T r	protein_coding	NP_001274748.1;NP_001274749.1;	3.8
LOC101930129	uncharacterized LOC101930129, transcript varian	lncRNA	.	3.8
LOC105371637	uncharacterized LOC105371637	lncRNA	.	3.8
LINC02882	long intergenic non-protein coding RNA 2882	lncRNA	.	3.8
MAGOH2P	mago homolog 2, pseudogene	ranscribed_pseudogen	.	3.8
LOC105376090	uncharacterized LOC105376090, transcript varian	lncRNA	.	3.8
MICOS10P1	MICOS10 pseudogene 1	ranscribed_pseudogen	.	3.8
LOC112268242	.	pseudogene	.	3.8
AURKAP1	aurora kinase A pseudogene 1	ranscribed_pseudogen	.	3.8
LOC105377599	uncharacterized LOC105377599	lncRNA	.	3.8
LOC107985595	uncharacterized LOC107985595	lncRNA	.	3.8
LOC107987084	uncharacterized LOC107987084, transcript varian	lncRNA	.	3.8
CEP83	centrosomal protein 83	protein_coding	NP_001035858.1;NP_001333386.1;	3.8
LOC105370353	uncharacterized LOC105370353	lncRNA	.	3.8
PTX3	pentraxin 3	protein_coding	NP_002843.2	3.8
LOC112267939	uncharacterized LOC112267939	protein_coding	XP_024302052.1	3.8
LOC102723654	uncharacterized LOC102723654, transcript varian	lncRNA	.	3.8
BMS1P1	BMS1 pseudogene 1	ranscribed_pseudogen	.	3.8

EID3	EP300 interacting inhibitor of differentiation 3	protein_coding	NP_001008395.1	3.8
ANKRD49P1	ANKRD49 pseudogene 1	pseudogene	.	3.8
GAS5	growth arrest specific 5	lncRNA	.	3.8
CREBRF	CREB3 regulatory factor	protein_coding	NP_001161865.1;NP_001161866.1;	3.8
LOC105370504	uncharacterized LOC105370504, transcript varian	lncRNA	.	3.8
LOC101929201	uncharacterized LOC101929201	lncRNA	.	3.8
C1QTNF3	C1q and TNF related 3	protein_coding	NP_112207.1;NP_852100.3	3.8
PDCD1LG2	programmed cell death 1 ligand 2	protein_coding	NP_079515.2;XP_005251657.1	3.8
LOC100419161	nei like DNA glycosylase 2 pseudogene	pseudogene	.	3.8
RPS26P11	ribosomal protein S26 pseudogene 11	ranscribed_pseudogen	.	3.8
NPIPB8	nuclear pore complex interacting protein family m	protein_coding	NP_001297065.1;XP_016879111.1;	3.8
SRSF11P1	serine and arginine rich splicing factor 11 pseudog	pseudogene	.	3.8
NDUFAF4P1	NADH:ubiquinone oxidoreductase complex assen	ranscribed_pseudogen	.	3.8
LOC107984131	uncharacterized LOC107984131	lncRNA	.	3.8
LOC112268025	uncharacterized LOC112268025	lncRNA	.	3.8
NSRP1	nuclear speckle splicing regulatory protein 1	protein_coding	NP_001248396.1;NP_115517.1;XP_	3.8
LOC105373623	uncharacterized LOC105373623, transcript varian	lncRNA	.	3.8
LOC105371967	uncharacterized LOC105371967, transcript varian	lncRNA	.	3.8
KC6	keratoconus gene 6	lncRNA	.	3.8
LOC105373716	uncharacterized LOC105373716, transcript varian	lncRNA	.	3.8
FDPSP3	farnesyl diphosphate synthase pseudogene 3	pseudogene	.	3.8
LOC102724832	uncharacterized LOC102724832, transcript varian	lncRNA	.	3.8
LOC105373302	uncharacterized LOC105373302, transcript varian	lncRNA	.	3.8
LOC107986699	uncharacterized LOC107986699	lncRNA	.	3.8
UBE2FP1	UBE2F pseudogene 1	ranscribed_pseudogen	.	3.8
GNRH1	gonadotropin releasing hormone 1	protein_coding	NP_000816.4;NP_001076580.1	3.8
RPS6P1	ribosomal protein S6 pseudogene 1	pseudogene	.	3.8
IFIT3	interferon induced protein with tetratricopeptide re	protein_coding	NP_001026853.1;NP_001276687.1;	3.8
LOC107984781	uncharacterized LOC107984781	lncRNA	.	3.8
LOC105373215	uncharacterized LOC105373215, transcript varian	lncRNA	.	3.8
ANKRD44	ankyrin repeat domain 44	protein_coding	NP_001182073.1;NP_001354424.1;	3.8
TAF4B	TATA-box binding protein associated factor 4b	protein_coding	NP_001280654.1;NP_005631.1;XP_	3.8
TUT7	terminal uridylyl transferase 7	protein_coding	NP_001171988.1;NP_001172003.1;	3.8
LOC105369464	uncharacterized LOC105369464, transcript varian	lncRNA	.	3.8
SLFN5	schlafen family member 5	protein_coding	NP_001317112.1;NP_659412.3	3.8
GRM8	glutamate metabotropic receptor 8	protein_coding	NP_000836.2;NP_001120795.1;NP_	3.8
LOC107986637	uncharacterized LOC107986637	lncRNA	.	3.8
NOTCH2NLA	notch 2 N-terminal like A	protein_coding	NP_001350935.1;NP_982283.2	3.8
RPS29P21	ribosomal protein S29 pseudogene 21	pseudogene	.	3.8
ARHGAP11B	Rho GTPase activating protein 11B	protein_coding	NP_001034930.1	3.8
LOC107984791	uncharacterized LOC107984791	lncRNA	.	3.8
SNORD21	small nucleolar RNA, C/D box 21	snoRNA	.	3.8
PDE4DIP	phosphodiesterase 4D interacting protein	protein_coding	NP_001002810.1;NP_001002811.2;	3.8
LOC107986206	uncharacterized LOC107986206	lncRNA	.	3.8
RTCA-AS1	RTCA antisense RNA 1	lncRNA	.	3.8
GSAP	gamma-secretase activating protein	protein_coding	NP_001337825.1;NP_001337826.1;	3.8
RPL34P24	ribosomal protein L34 pseudogene 24	pseudogene	.	3.7
FAM111A-DT	FAM111A divergent transcript	lncRNA	.	3.7
LOC100129554	thyroid hormone receptor interactor 4 pseudogene	pseudogene	.	3.7
UBE2E2	ubiquitin conjugating enzyme E2 E2	protein_coding	NP_001357154.1;NP_001357155.1;	3.7
TRK-CTT6-1	tRNA-Lys	tRNA	.	3.7
CHMP4BP1	charged multivesicular body protein 4B pseudoger	pseudogene	.	3.7
LINC00632	long intergenic non-protein coding RNA 632	lncRNA	.	3.7
NAIP	NLR family apoptosis inhibitory protein	protein_coding	NP_001333799.1;NP_004527.2;NP_	3.7
AHI1	Abelson helper integration site 1	protein_coding	NP_001128302.1;NP_001128303.1;	3.7
LINC01176	long intergenic non-protein coding RNA 1176	lncRNA	.	3.7
ENTPD1	ectonucleoside triphosphate diphosphohydrolase 1	protein_coding	NP_001091645.1;NP_001157650.1;	3.7
LOC105374003	uncharacterized LOC105374003	lncRNA	.	3.7
GHRLOS	ghrelin opposite strand/antisense RNA	lncRNA	.	3.7
RPL13AP15	ribosomal protein L13a pseudogene 15	pseudogene	.	3.7
MIR374B	microRNA 374b	miRNA	.	3.7
LOC105377508	uncharacterized LOC105377508, transcript varian	lncRNA	.	3.7
LOC105379749	uncharacterized LOC105379749, transcript varian	lncRNA	.	3.7
LOC107984972	.	pseudogene	.	3.7
PRELID2	PRELI domain containing 2	protein_coding	NP_612501.3;NP_892005.1;NP_99	3.7
GSDMC	gasdermin C	protein_coding	NP_113603.1;XP_011515460.1;XP_	3.7
KCTD9P2	potassium channel tetramerization domain contain	pseudogene	.	3.7
RNU6-60P	RNA, U6 small nuclear 60, pseudogene	pseudogene	.	3.7
ATP2C2-AS1	ATP2C2 antisense RNA 1	lncRNA	.	3.7
DCAF4L1	DDB1 and CUL4 associated factor 4 like 1	protein_coding	NP_001025126.2	3.7
CHD2	chromodomain helicase DNA binding protein 2	protein_coding	NP_001036037.1;NP_001262.3	3.7

ATP1A1-AS1	ATP1A1 antisense RNA 1	lncRNA	.	3.7
LOC112268256	uncharacterized LOC112268256	lncRNA	.	3.7
LRRC37A4P	leucine rich repeat containing 37 member A4, pseu	ranscribed_pseudogene	.	3.7
LOC112267857	uncharacterized LOC112267857, transcript varian	protein_coding	XP_011534584.1;XP_016866834.1	3.7
ZCWPW2	zinc finger CW-type and PWWP domain containir	protein_coding	NP_001035522.1;NP_001311098.1;	3.7
MIR25	microRNA 25	miRNA	.	3.7
NOTCH2NLC	notch 2 N-terminal like C	protein_coding	NP_001350941.1;NP_001350942.1	3.7
LOC100216337	ADP ribosylation factor like GTPase 1 pseudogene	pseudogene	.	3.7
UTY	ubiquitously transcribed tetratricopeptide repeat cc	protein_coding	NP_001245178.1;NP_001245179.1;	3.7
TMSB15B-AS1	TMSB15B antisense RNA 1	lncRNA	.	3.7
LOC105372356	uncharacterized LOC105372356, transcript varian	lncRNA	.	3.7
LOC105374020	uncharacterized LOC105374020, transcript varian	lncRNA	.	3.7
LOC107984104	uncharacterized LOC107984104, transcript varian	protein_coding	XP_016883676.1;XP_016883677.1;	3.7
LOC102724584	uncharacterized LOC102724584	lncRNA	.	3.7
SNRPGP3	small nuclear ribonucleoprotein polypeptide G pse	pseudogene	.	3.7
ZNF407	zinc finger protein 407	protein_coding	NP_001139661.1;NP_001139662.1;	3.7
OSMR-AS1	OSMR antisense RNA 1 (head to head)	lncRNA	.	3.7
CYP1B1-AS1	CYP1B1 antisense RNA 1	lncRNA	.	3.7
LOC100422317	proteasome 26S subunit, ATPase 5 pseudogene	pseudogene	.	3.7
ZNF841	zinc finger protein 841	protein_coding	NP_001129971.1;NP_001308278.1;	3.7
LOC100288073	heat shock protein family A (Hsp70) member 5 ps	pseudogene	.	3.7
LOC107987147	uncharacterized LOC107987147	lncRNA	.	3.7
SNORD103B	small nucleolar RNA, C/D box 103B	snoRNA	.	3.7
LOC102724517	uncharacterized LOC102724517	lncRNA	.	3.7
LOC105373296	uncharacterized LOC105373296	lncRNA	.	3.6
CNIH3	cornichon family AMPA receptor auxiliary protein	protein_coding	NP_001309231.1;NP_001309232.1;	3.6
AGAP13P	ArfGAP with GTPase domain, ankyrin repeat and	ranscribed_pseudogene	.	3.6
LOC105369698	uncharacterized LOC105369698	lncRNA	.	3.6
ANAPC1P2	ANAPC1 pseudogene 2	ranscribed_pseudogene	.	3.6
LOC100422669	tyrosine 3-monooxygenase/tryptophan 5-monooxy	pseudogene	.	3.6
WAKMAR2	wound and keratinocyte migration associated lncR	lncRNA	.	3.6
DGKH	diacylglycerol kinase eta	protein_coding	NP_001191433.1;NP_001191434.1;	3.6
SLC4A4	solute carrier family 4 member 4	protein_coding	NP_001091954.1;NP_001128214.1;	3.6
LOC107985399	uncharacterized LOC107985399	lncRNA	.	3.6
LOC105379173	uncharacterized LOC105379173	lncRNA	.	3.6
LOC105371630	uncharacterized LOC105371630	lncRNA	.	3.6
FMR1-AS1	FMR1 antisense RNA 1	lncRNA	.	3.6
ERRFI1	ERBB receptor feedback inhibitor 1	protein_coding	NP_061821.1;XP_005263534.1;XP_	3.6
PHF11	PHD finger protein 11	protein_coding	NP_001035533.1;NP_001035534.1	3.6
LOC107985400	uncharacterized LOC107985400, transcript varian	lncRNA	.	3.6
LOC107985977	uncharacterized LOC107985977	lncRNA	.	3.6
CYTB	cytochrome b	protein_coding	YP_003024038.1	3.6
NPIPB9	nuclear pore complex interacting protein family m	protein_coding	NP_001274179.1;NP_001274180.1;	3.6
RRS1-AS1	RRS1 antisense RNA 1 (head to head)	lncRNA	.	3.6
EAF2	ELL associated factor 2	protein_coding	NP_001306970.1;NP_060926.2;XP_	3.6
LRRC37A	leucine rich repeat containing 37A	protein_coding	NP_055649.4	3.6
SPART-AS1	SPART antisense RNA 1	lncRNA	.	3.6
RNF207	ring finger protein 207	protein_coding	NP_997279.2;XP_011539741.1;XP_	3.6
EYA4	EYA transcriptional coactivator and phosphatase 4	protein_coding	NP_001287941.1;NP_001287942.1;	3.6
MIR7-1	microRNA 7-1	miRNA	.	3.6
USP9Y	ubiquitin specific peptidase 9 Y-linked	protein_coding	NP_004645.2;XP_016885567.1	3.6
TAF1	TATA-box binding protein associated factor 1	protein_coding	NP_001273003.2;NP_004597.3;NP_	3.6
LOC105374333	uncharacterized LOC105374333	lncRNA	.	3.6
BNC1	basonuclin 1	protein_coding	NP_001288135.1;NP_001708.3;XP_	3.6
LOC107985295	uncharacterized LOC107985295, transcript varian	lncRNA	.	3.6
NPIPB2	nuclear pore complex interacting protein family m	protein_coding	NP_001342443.1;XP_024306192.1;	3.6
SMG1	SMG1 nonsense mediated mRNA decay associated	protein_coding	NP_055907.3;XP_005255239.1;XP_	3.6
PTPRK	protein tyrosine phosphatase receptor type K	protein_coding	NP_001129120.1;NP_001278910.1;	3.6
HSD17B3	hydroxysteroid 17-beta dehydrogenase 3	protein_coding	NP_000188.1;XP_011516920.1;XP_	3.6
FOSL1P1	FOSL1 pseudogene 1	pseudogene	.	3.6
HIVEP1	HIVEP zinc finger 1	protein_coding	NP_002105.3;XP_011512848.1;XP_	3.6
GOLGA8N	golgin A8 family member N	protein_coding	NP_001269423.1	3.6
PPFIA2	PTPRF interacting protein alpha 2	protein_coding	NP_001207402.1;NP_001207403.1;	3.6
LOC100996442	uncharacterized LOC100996442, transcript varian	misc_RNA	.	3.6
PLCB4	phospholipase C beta 4	protein_coding	NP_000924.3;NP_001166117.1;NP_	3.6
MIR548AQ	hsa-miR-548aq-3p	.	.	3.6
LOC389473	chromosome 5 open reading frame 13 pseudogene	pseudogene	.	3.6
GBP3	guanylate binding protein 3	protein_coding	NP_001306108.1;NP_001306109.1;	3.6
MIR30C2	microRNA 30c-2	miRNA	.	3.6
DOCK5	dedicator of cytokinesis 5	protein_coding	NP_001309739.1;NP_079216.4	3.6
STRIP2	striatin interacting protein 2	protein_coding	NP_001127808.1;NP_065755.1;XP_	3.6

ITGA1	integrin subunit alpha 1	protein_coding	NP_852478.1	3.6
LOC105376301	uncharacterized LOC105376301	lncRNA	.	3.6
SP140L	SP140 nuclear body protein like	protein_coding	NP_001295091.1;NP_001295092.1;	3.6
AVIL	advillin	protein_coding	NP_006567.3;XP_016874199.1;XP_	3.6
IGIP	IgA inducing protein	protein_coding	NP_001007190.1	3.6
LOC107985780	uncharacterized LOC107985780	lncRNA	.	3.6
LOC107984361	uncharacterized LOC107984361, transcript varian	lncRNA	.	3.6
LOC107984391	uncharacterized LOC107984391	lncRNA	.	3.6
LOC107984583	uncharacterized LOC107984583	lncRNA	.	3.6
LOC107985263	uncharacterized LOC107985263	lncRNA	.	3.6
TCEA1P3	transcription elongation factor A1 pseudogene 3	pseudogene	.	3.6
SNORA7B	small nucleolar RNA, H/ACA box 7B	snoRNA	.	3.5
ERAP1	endoplasmic reticulum aminopeptidase 1	protein_coding	NP_001035548.1;NP_001185470.1;	3.5
TMEM225B	transmembrane protein 225B	protein_coding	NP_001182470.1;NP_001182471.1;	3.5
LINC02595	long intergenic non-protein coding RNA 2595	lncRNA	.	3.5
KIZ	kizuna centrosomal protein	protein_coding	NP_001156494.1;NP_001156495.1;	3.5
ATP8A2	ATPase phospholipid transporting 8A2	protein_coding	NP_001300670.1;NP_057613.4;XP_	3.5
KDR	kinase insert domain receptor	protein_coding	NP_002244.1	3.5
POC1B-GALNT4	POC1B-GALNT4 readthrough	protein_coding	NP_001186710.1;NP_001186711.1	3.5
LOC105369720	uncharacterized LOC105369720, transcript varian	lncRNA	.	3.5
CEP290	centrosomal protein 290	protein_coding	NP_079390.3;XP_011537058.1;XP_	3.5
PRORSD1P	prolyl-tRNA synthetase associated domain contair	ranscribed_pseudogen	.	3.5
CLIP4	CAP-Gly domain containing linker protein family	protein_coding	NP_001274456.1;NP_001274457.1;	3.5
RFX3	regulatory factor X3	protein_coding	NP_001269045.1;NP_001269046.1;	3.5
CCDC14	coiled-coil domain containing 14	protein_coding	NP_001295246.1;NP_001353264.1;	3.5
CD226	CD226 molecule	protein_coding	NP_001290547.1;NP_001290548.1;	3.5
LOC105374439	uncharacterized LOC105374439	lncRNA	.	3.5
LOC105372698	uncharacterized LOC105372698, transcript varian	misc_RNA	.	3.5
LOC100421358	CREB regulated transcription coactivator 2 pseud	pseudogene	.	3.5
LOC388572	uncharacterized LOC388572	lncRNA	.	3.5
LOC107986272	uncharacterized LOC107986272	lncRNA	.	3.5
WDFY2	WD repeat and FYVE domain containing 2	protein_coding	NP_443182.1;XP_011533216.1;XP_	3.5
LOC105373456	uncharacterized LOC105373456, transcript varian	lncRNA	.	3.5
NR2F2-AS1	NR2F2 antisense RNA 1	lncRNA	.	3.5
TPR	translocated promoter region, nuclear basket prote	protein_coding	NP_003283.2	3.5
SNORD54	small nucleolar RNA, C/D box 54	snoRNA	.	3.5
FAM157B	family with sequence similarity 157 member B	lncRNA	.	3.5
NR4A2	nuclear receptor subfamily 4 group A member 2	protein_coding	NP_006177.1;NP_775265.1;XP_00	3.5
LOC105372117	uncharacterized LOC105372117	lncRNA	.	3.5
SP140	SP140 nuclear body protein	protein_coding	NP_001005176.1;NP_001265380.1;	3.5
LOC112267866	uncharacterized LOC112267866, transcript varian	lncRNA	.	3.5
CTH	cystathionine gamma-lyase	protein_coding	NP_001177392.1;NP_001893.2;NP_	3.5
NPIPA5	nuclear pore complex interacting protein family m	protein_coding	NP_001264254.1;NP_001338129.1;	3.5
OFD1	OFD1 centriole and centriolar satellite protein	protein_coding	NP_001317138.1;NP_001317139.1;	3.5
LOC112268474	uncharacterized LOC112268474	lncRNA	.	3.5
MIR6755	microRNA 6755	miRNA	.	3.5
FNBP4	formin binding protein 4	protein_coding	NP_001305268.1;NP_056123.2;XP_	3.5
SLAMF7	SLAM family member 7	protein_coding	NP_001269517.1;NP_001269518.1;	3.5
RRAGB	Ras related GTP binding B	protein_coding	NP_001340940.1;NP_001340942.1;	3.5
ABCA1	ATP binding cassette subfamily A member 1	protein_coding	NP_005493.2;XP_005251830.1;XP_	3.5
NAV3	neuron navigator 3	protein_coding	NP_001019554.1;NP_055718.4;XP_	3.5
BAZ2B	bromodomain adjacent to zinc finger domain 2B	protein_coding	NP_001276904.1;NP_001316786.1;	3.5
VEGFC	vascular endothelial growth factor C	protein_coding	NP_005420.1	3.5
LOC105374187	uncharacterized LOC105374187, transcript varian	lncRNA	.	3.5
MAB21L3	mab-21 like 3	protein_coding	NP_689580.2;XP_011538925.1;XP_	3.5
LOC112267894	uncharacterized LOC112267894	lncRNA	.	3.5
IFIT1	interferon induced protein with tetratricopeptide re	protein_coding	NP_001257856.1;NP_001257857.1;	3.5
SNORA40	small nucleolar RNA, H/ACA box 40	snoRNA	.	3.5
LOC100288637	OTU deubiquitinase 7A pseudogene	ranscribed_pseudogen	.	3.5
SCARNA26A	small Cajal body-specific RNA 26A	guide_RNA	.	3.5
ABCC9	ATP binding cassette subfamily C member 9	protein_coding	NP_001364202.1;NP_001364203.1;	3.5
ZDHHC11	zinc finger DHHC-type containing 11	protein_coding	NP_079062.1;XP_016865358.1;XP_	3.5
LINC02804	long intergenic non-protein coding RNA 2804, tra	lncRNA	.	3.5
LOC107985967	uncharacterized LOC107985967	lncRNA	.	3.5
CCDC7	coiled-coil domain containing 7	protein_coding	NP_001021554.1;NP_001308044.1;	3.5
GABPB1-AS1	GABPB1 antisense RNA 1	lncRNA	.	3.5
TRMT11	tRNA methyltransferase 11 homolog	protein_coding	NP_001026882.2;NP_001337509.1;	3.5
FLJ31356	uncharacterized protein FLJ31356	lncRNA	.	3.5
KCNQ3	potassium voltage-gated channel subfamily Q mer	protein_coding	NP_001191753.1;NP_004510.1;XP_	3.5
PHEX	phosphate regulating endopeptidase homolog X-li	protein_coding	NP_000435.3;NP_001269683.1;XP_	3.4
LOC107986434	uncharacterized LOC107986434	lncRNA	.	3.4

LOC107985873	uncharacterized LOC107985873	lncRNA	.	3.4
ATF4	activating transcription factor 4	protein_coding	NP_001666.2;NP_877962.1;XP_01(	3.4
SLTM	SAFB like transcription modulator	protein_coding	NP_001013865.1;NP_079031.2;XP_	3.4
PPIP5K1P1	diphosphoinositol pentakisphosphate kinase 1 pseu	pseudogene	.	3.4
CFDP1	craniofacial development protein 1	protein_coding	NP_006315.1;XP_011521116.1;XP_	3.4
SNORD117	small nucleolar RNA, C/D box 117	snoRNA	.	3.4
LOC101928682	uncharacterized LOC101928682	lncRNA	.	3.4
ANKRD36C	ankyrin repeat domain 36C	protein_coding	NP_001297083.1	3.4
LOC101929964	uncharacterized LOC101929964	lncRNA	.	3.4
PTPRR	protein tyrosine phosphatase receptor type R	protein_coding	NP_001193944.1;NP_001193945.1;	3.4
LOC107984261	uncharacterized LOC107984261	lncRNA	.	3.4
MRPL42P5	mitochondrial ribosomal protein L42 pseudogene :	ranscribed_pseudogen	.	3.4
GREM1	gremlin 1, DAN family BMP antagonist	protein_coding	NP_001178251.1;NP_001178252.1;	3.4
SNORD103C	small nucleolar RNA, C/D box 103C	snoRNA	.	3.4
SGK1	serum/glucocorticoid regulated kinase 1	protein_coding	NP_001137148.1;NP_001137149.1;	3.4
IL20RB	interleukin 20 receptor subunit beta	protein_coding	NP_653318.2;XP_006713728.1;XP_	3.4
MTARC1	mitochondrial amidoxime reducing component 1	protein_coding	NP_073583.3;XP_011508202.1;XP_	3.4
RNU6ATAC	RNA, U6atac small nuclear (U12-dependent splici	snRNA	.	3.4
SUPT3H	SPT3 homolog, SAGA and STAGA complex com	protein_coding	NP_001248752.1;NP_001337253.1;	3.4
GOLGA6L10	golgin A6 family like 10	protein_coding	NP_001157937.2	3.4
DST	dystonin	protein_coding	NP_001138241.1;NP_001138242.1;	3.4
CD99P1	CD99 molecule pseudogene 1	ranscribed_pseudogen	.	3.4
MIR3175	microRNA 3175	miRNA	.	3.4
GOLGA6L4	golgin A6 family like 4	protein_coding	NP_001254465.2;XP_016877970.1;	3.4
ZNF888	zinc finger protein 888	protein_coding	NP_001297056.1;XP_005259508.1;	3.4
PAN2	poly(A) specific ribonuclease subunit PAN2	protein_coding	NP_001120932.2;NP_001159751.2;	3.4
ADAMTS16	ADAM metallopeptidase with thrombospondin typ	protein_coding	NP_620687.2	3.4
NR3C2	nuclear receptor subfamily 3 group C member 2	protein_coding	NP_000892.2;NP_001159576.1;NP_	3.4
PFDN2	prefoldin subunit 2	protein_coding	NP_036526.2;XP_011507926.1	3.4
WDR35	WD repeat domain 35	protein_coding	NP_001006658.1;NP_065830.2;XP_	3.4
LOC105373310	uncharacterized LOC105373310	lncRNA	.	3.4
LOC107987057	uncharacterized LOC107987057	lncRNA	.	3.4
RPS3AP12	RPS3A pseudogene 12	pseudogene	.	3.4
PINLYP	phospholipase A2 inhibitor and LY6/PLAUR dom	protein_coding	NP_001180550.2;NP_001180551.1;	3.4
TRIM55	tripartite motif containing 55	protein_coding	NP_149047.2;NP_908973.1;NP_90	3.4
LOC112267872	uncharacterized LOC112267872, transcript varian	lncRNA	.	3.4
SNORD19C	small nucleolar RNA, C/D box 19C	snoRNA	.	3.4
LNCSRLR	lncRNA sorafenib resistance in renal cell carcinon	lncRNA	.	3.4
SLC10A7	solute carrier family 10 member 7	protein_coding	NP_001025169.1;NP_001287771.1;	3.4
SUMO4	small ubiquitin like modifier 4	protein_coding	NP_001002255.1	3.4
ZRSR2	zinc finger CCCH-type, RNA binding motif and s	protein_coding	NP_005080.1;XP_005274654.2;XP_	3.4
UNC5C	unc-5 netrin receptor C	protein_coding	NP_003719.3;XP_005263378.1;XP_	3.4
LOC105375521	uncharacterized LOC105375521	lncRNA	.	3.4
VN1R101P	vomeronasal 1 receptor 101 pseudogene	pseudogene	.	3.4
LOC107986655	uncharacterized LOC107986655, transcript varian	lncRNA	.	3.4
SEMA3A	semaphorin 3A	protein_coding	NP_006071.1;XP_005250167.1;XP_	3.4
GMDS-DT	GMDS divergent transcript	lncRNA	.	3.4
RPL4P6	ribosomal protein L4 pseudogene 6	pseudogene	.	3.4
E2F7	E2F transcription factor 7	protein_coding	NP_976328.2;XP_011536268.1;XP_	3.4
GPRC5D-AS1	GPRC5D and HEBP1 antisense RNA 1	lncRNA	.	3.4
RPL7P49	ribosomal protein L7 pseudogene 49	pseudogene	.	3.4
NPIPB4	nuclear pore complex interacting protein family m	protein_coding	NP_001297077.1;XP_016878733.1;	3.4
PRKY	protein kinase Y-linked (pseudogene)	ranscribed_pseudogen	.	3.4
TSSK1B	testis specific serine kinase 1B	protein_coding	NP_114417.1	3.4
LOC100419073	nucleoporin 155kDa pseudogene	pseudogene	.	3.3
KANSL1L-AS1	KANSL1L antisense RNA 1	lncRNA	.	3.3
TAPT1-AS1	TAPT1 antisense RNA 1 (head to head)	lncRNA	.	3.3
LUC7L3	LUC7 like 3 pre-mRNA splicing factor	protein_coding	NP_001317259.1;NP_006098.2;NP_	3.3
LOC105372976	uncharacterized LOC105372976	lncRNA	.	3.3
SAP30L-AS1	SAP30L antisense RNA 1 (head to head)	lncRNA	.	3.3
LINC01004	long intergenic non-protein coding RNA 1004	lncRNA	.	3.3
AMPD3	adenosine monophosphate deaminase 3	protein_coding	NP_000471.1;NP_001020560.1;NP_	3.3
LOC112267861	uncharacterized LOC112267861, transcript varian	lncRNA	.	3.3
LOC107984192	uncharacterized LOC107984192	lncRNA	.	3.3
FAAH2	fatty acid amide hydrolase 2	protein_coding	NP_001340769.1;NP_001340770.1;	3.3
PDXDC2P	pyridoxal dependent decarboxylase domain contai	pseudogene	.	3.3
ZNF438	zinc finger protein 438	protein_coding	NP_001137238.1;NP_001137239.1;	3.3
LOC100419896	zinc finger protein 267 pseudogene	pseudogene	.	3.3
LOC100420429	ubiquitin specific peptidase 15 pseudogene	pseudogene	.	3.3
ATM	ATM serine/threonine kinase	protein_coding	NP_000042.3;NP_001338763.1;NP_	3.3
PHBP19	prohibitin pseudogene 19	pseudogene	.	3.3

LOC107985440	uncharacterized LOC107985440, transcript varian	lncRNA	.	3.3
LOC101927556	uncharacterized LOC101927556, transcript varian	lncRNA	.	3.3
CLHC1	clathrin heavy chain linker domain containing 1	protein_coding	NP_001129070.1;NP_001340708.1;	3.3
FAM167A	family with sequence similarity 167 member A	protein_coding	NP_444509.2;XP_005272455.1;XP_	3.3
LOC103156999	dynein light chain Tctex-type 1 pseudogene	pseudogene	.	3.3
LOC100132686	uncharacterized LOC100132686	lncRNA	.	3.3
EFL1	elongation factor like GTPase 1	protein_coding	NP_001035700.1;NP_001309773.1;	3.3
TYW1B	tRNA-yW synthesizing protein 1 homolog B	protein_coding	NP_001138912.2	3.3
DLGAP1-AS1	DLGAP1 antisense RNA 1	lncRNA	.	3.3
LOC107985367	uncharacterized LOC107985367	lncRNA	.	3.3
LOC102723376	uncharacterized LOC102723376	lncRNA	.	3.3
LOC105375384	uncharacterized LOC105375384	lncRNA	.	3.3
LOC107985230	uncharacterized LOC107985230	lncRNA	.	3.3
PHF5GP	PHD finger protein 5G pseudogene	pseudogene	.	3.3
LOC107984376	uncharacterized LOC107984376	lncRNA	.	3.3
C5orf66	chromosome 5 open reading frame 66	lncRNA	.	3.3
RPS14P3	ribosomal protein S14 pseudogene 3	ranscribed_pseudogen	.	3.3
CBWD6	COBW domain containing 6	protein_coding	NP_001078926.1;XP_016870508.1;	3.3
ZNF761	zinc finger protein 761	protein_coding	NP_001008401.3;NP_001276880.1;	3.3
HMBOX1	homeobox containing 1	protein_coding	NP_001129198.1;NP_001311311.1;	3.3
LRRC70	leucine rich repeat containing 70	protein_coding	NP_852607.3	3.3
LOC107986348	uncharacterized LOC107986348	lncRNA	.	3.3
ALG13	ALG13 UDP-N-acetylglucosaminyltransferase sul	protein_coding	NP_001034299.3;NP_001093392.1;	3.3
KDM4C	lysine demethylase 4C	protein_coding	NP_001140167.1;NP_001140168.1;	3.3
SNORD16	small nucleolar RNA, C/D box 16	snoRNA	.	3.3
MECOM	MDS1 and EVI1 complex locus	protein_coding	NP_001098547.3;NP_001098548.2;	3.3
ANKRD36	ankyrin repeat domain 36	protein_coding	NP_001341516.1;NP_940957.3;XP_	3.3
CMC1	C-X9-C motif containing 1	protein_coding	NP_001318114.1;NP_001318115.1;	3.3
C9orf24	chromosome 9 open reading frame 24	protein_coding	NP_001239124.1;NP_115985.2;NP_	3.3
LOC105370124	uncharacterized LOC105370124	lncRNA	.	3.3
IFI16	interferon gamma inducible protein 16	protein_coding	NP_001193496.1;NP_001351796.1;	3.3
CNTF	ciliary neurotrophic factor	protein_coding	NP_000605.1	3.3
EFHC1	EF-hand domain containing 1	protein_coding	NP_001165891.1;NP_060570.2	3.3
RPSAP16	ribosomal protein SA pseudogene 16	pseudogene	.	3.3
MRPS25	mitochondrial ribosomal protein S25	protein_coding	NP_071942.1	3.3
LOC105374748	uncharacterized LOC105374748, transcript varian	lncRNA	.	3.3
ITGB1-DT	ITGB1 divergent transcript, transcript variant X2	lncRNA	.	3.3
EVI2B	ecotropic viral integration site 2B	protein_coding	NP_006486.3;XP_005258003.1	3.3
LOC101927603	uncharacterized LOC101927603	lncRNA	.	3.3
LOC105372206	uncharacterized LOC105372206	lncRNA	.	3.3
RICTOR	RPTOR independent companion of MTOR compl	protein_coding	NP_001272368.1;NP_001272369.1;	3.3
LOC101929828	uncharacterized LOC101929828, transcript varian	lncRNA	.	3.3
LOC107986075	uncharacterized LOC107986075	lncRNA	.	3.3
PUM3	pumilio RNA binding family member 3	protein_coding	NP_055693.4	3.3
CCDC30	coiled-coil domain containing 30	protein_coding	NP_001074319.1;NP_001342153.1;	3.3
LOC107985481	uncharacterized LOC107985481	lncRNA	.	3.3
LINC01684	long intergenic non-protein coding RNA 1684	lncRNA	.	3.3
RNU1-16P	RNA, U1 small nuclear 16, pseudogene	pseudogene	.	3.3
ST7-OT3	ST7 overlapping transcript 3	lncRNA	.	3.3
NCKAP5	NCK associated protein 5	protein_coding	NP_997246.2;NP_997364.3;XP_00	3.3
SEC24D	SEC24 homolog D, COPII coat complex compone	protein_coding	NP_001304995.1;NP_055637.2;XP_	3.3
STARD9	StAR related lipid transfer domain containing 9	protein_coding	NP_065810.2;XP_011520133.1;XP_	3.3
LINC00672	long intergenic non-protein coding RNA 672	lncRNA	.	3.3
LOC107986127	uncharacterized LOC107986127	lncRNA	.	3.3
LOC100190986	uncharacterized LOC100190986	lncRNA	.	3.3
LOC102724857	uncharacterized LOC102724857, transcript varian	lncRNA	.	3.3
COX6C	cytochrome c oxidase subunit 6C	protein_coding	NP_004365.1;XP_016868509.1	3.2
LOC107985851	uncharacterized LOC107985851	lncRNA	.	3.2
LOC105370941	uncharacterized LOC105370941	lncRNA	.	3.2
SLC38A6	solute carrier family 38 member 6	protein_coding	NP_001166173.1;NP_722518.2;XP_	3.2
LOC107985057	uncharacterized LOC107985057	lncRNA	.	3.2
RYR2	ryanodine receptor 2	protein_coding	NP_001026.2;XP_006711865.1;XP_	3.2
XRRA1	X-ray radiation resistance associated 1	protein_coding	NP_001257309.1;NP_001257310.1;	3.2
LOC107984798	uncharacterized LOC107984798	lncRNA	.	3.2
SNX18P12	sorting nexin 18 pseudogene 12	pseudogene	.	3.2
IDS2	uncharacterized IDS2, transcript variant X1	lncRNA	.	3.2
RPL5P18	ribosomal protein L5 pseudogene 18	pseudogene	.	3.2
BCLAF3	BCLAF1 and THRAP3 family member 3	protein_coding	NP_001354703.1;NP_938020.2;XP_	3.2
TRPM7	transient receptor potential cation channel subfami	protein_coding	NP_001288141.1;NP_060142.3;XP_	3.2
THAP5P1	THAP domain containing 5 pseudogene 1	pseudogene	.	3.2
HCG20	HLA complex group 20	lncRNA	.	3.2

ASNS	asparagine synthetase (glutamine-hydrolyzing)	protein_coding	NP_001171546.1;NP_001171547.1;	3.2
DKFZP586I1420	uncharacterized protein DKFZp586I1420	ranscribed_pseudogen	.	3.2
TPM3P9	tropomyosin 3 pseudogene 9	ranscribed_pseudogen	.	3.2
LOC105374464	uncharacterized LOC105374464, transcript varian	lncRNA	.	3.2
NBPF25P	NBPF member 25, pseudogene	ranscribed_pseudogen	.	3.2
ZNF648	zinc finger protein 648	protein_coding	NP_001009992.1;XP_024309026.1;	3.2
ACTG1P14	actin gamma 1 pseudogene 14	pseudogene	.	3.2
ZNF137P	zinc finger protein 137, pseudogene	ranscribed_pseudogen	.	3.2
VPS13B	vacuolar protein sorting 13 homolog B	protein_coding	NP_056058.2;NP_060360.3;NP_68	3.2
FAM133CP	family with sequence similarity 133 member C, ps	ranscribed_pseudogen	.	3.2
GEMIN8	gem nuclear organelle associated protein 8	protein_coding	NP_001035944.1;NP_001035945.1;	3.2
FTH1P16	ferritin heavy chain 1 pseudogene 16	pseudogene	.	3.2
LOC105373218	uncharacterized LOC105373218, transcript varian	lncRNA	.	3.2
LOC102724740	uncharacterized LOC102724740, transcript varian	lncRNA	.	3.2
SLC13A3	solute carrier family 13 member 3	protein_coding	NP_001011554.1;NP_001180268.1;	3.2
LOC102724775	uncharacterized LOC102724775	lncRNA	.	3.2
LOC105377778	uncharacterized LOC105377778, transcript varian	lncRNA	.	3.2
RPL23P2	ribosomal protein L23 pseudogene 2	pseudogene	.	3.2
DNAH7	dynein axonemal heavy chain 7	protein_coding	NP_061720.2;XP_011509790.1;XP_	3.2
MIR98	microRNA 98	miRNA	.	3.2
RN7SL566P	RNA, 7SL, cytoplasmic 566, pseudogene	pseudogene	.	3.2
NAIPP3	NAIP pseudogene 3	pseudogene	.	3.2
ZNF731P	zinc finger protein 731, pseudogene	pseudogene	.	3.2
FAM71F2	family with sequence similarity 71 member F2	protein_coding	NP_001012457.3;NP_001122398.1;	3.2
LINC00941	long intergenic non-protein coding RNA 941	lncRNA	.	3.2
PRUNE2	prune homolog 2 with BCH domain	protein_coding	NP_001294976.1;NP_001294977.1;	3.2
ASAP1-IT2	ASAP1 intronic transcript 2	lncRNA	.	3.2
DNAH6	dynein axonemal heavy chain 6	protein_coding	NP_001361.1;XP_006712019.1;XP_	3.2
ZNRD1ASP	zinc ribbon domain containing 1 antisense, pseudo	ranscribed_pseudogen	.	3.2
LOC107983971	uncharacterized LOC107983971, transcript varian	lncRNA	.	3.2
LOC112268260	uncharacterized LOC112268260, transcript varian	protein_coding	XP_024307730.1;XP_024307731.1	3.2
TARBP1	TAR (HIV-1) RNA binding protein 1	protein_coding	NP_005637.3;XP_005273291.1;XP_	3.2
IGSF10	immunoglobulin superfamily member 10	protein_coding	NP_001171616.1;NP_001171617.1;	3.2
PPP1R12B	protein phosphatase 1 regulatory subunit 12B	protein_coding	NP_001161329.1;NP_001161330.1;	3.2
FABP3	fatty acid binding protein 3	protein_coding	NP_001307925.1;NP_004093.1;XP_	3.2
LINC02057	long intergenic non-protein coding RNA 2057	lncRNA	.	3.2
BMS1P2	BMS1 pseudogene 2	ranscribed_pseudogen	.	3.2
RPSAP13	ribosomal protein SA pseudogene 13	pseudogene	.	3.2
RPL18AP9	ribosomal protein L18a pseudogene	pseudogene	.	3.2
IQCH	IQ motif containing H	protein_coding	NP_001026885.2;NP_001271276.2;	3.2
IRAK2	interleukin 1 receptor associated kinase 2	protein_coding	NP_001561.3	3.2
IFIT2	interferon induced protein with tetratricopeptide re	protein_coding	NP_001538.4	3.2
LOC105373511	uncharacterized LOC105373511, transcript varian	lncRNA	.	3.2
SCARNA18	small Cajal body-specific RNA 18	guide_RNA	.	3.2
LTA	lymphotoxin alpha	protein_coding	NP_000586.2;NP_001153212.1;XP_	3.2
BMS1P14	BMS1 pseudogene 14	ranscribed_pseudogen	.	3.2
LOC107984485	uncharacterized LOC107984485	lncRNA	.	3.2
L3HYPDH	trans-L-3-hydroxyproline dehydratase	protein_coding	NP_001318087.1;NP_001318088.1;	3.2
ZNF192P1	zinc finger protein 192 pseudogene 1	ranscribed_pseudogen	.	3.2
LOC102724907	uncharacterized LOC102724907	lncRNA	.	3.2
RN7SL689P	RNA, 7SL, cytoplasmic 689, pseudogene	pseudogene	.	3.2
NPIPB13	nuclear pore complex interacting protein family, n	protein_coding	NP_001308821.1	3.2
LOC107985139	uncharacterized LOC107985139, transcript varian	lncRNA	.	3.2
MYSM1	Myb like, SWIRM and MPN domains 1	protein_coding	NP_001078956.1;XP_006710377.1;	3.2
HCP5B	HLA complex P5B	lncRNA	.	3.2
SGK2	serum/glucocorticoid regulated kinase 2	protein_coding	NP_001186193.1;NP_057360.2;NP_	3.1
SCFD2	sec1 family domain containing 2	protein_coding	NP_689753.2;XP_011532677.1;XP_	3.1
LOC105376266	uncharacterized LOC105376266	lncRNA	.	3.1
OLMALINC	oligodendrocyte maturation-associated long interg	lncRNA	.	3.1
RAC1P3	Rac family small GTPase 1 pseudogene 3	pseudogene	.	3.1
DNM3-IT1	DNM3 intronic transcript 1	lncRNA	.	3.1
CD163	CD163 molecule	protein_coding	NP_001357074.1;NP_001357075.1;	3.1
GRK5-IT1	GRK5 intronic transcript 1	lncRNA	.	3.1
SHTN1	shootin 1	protein_coding	NP_001120683.1;NP_001245227.1;	3.1
RPL7AP9	ribosomal protein L7a pseudogene 9	pseudogene	.	3.1
LOC107984387	uncharacterized LOC107984387	lncRNA	.	3.1
KDELC1P1	KDEL motif containing 1 pseudogene 1	pseudogene	.	3.1
CCNL2	cyclin L2	protein_coding	NP_001034666.1;NP_001307082.1;	3.1
HYPK	huntingtin interacting protein K	protein_coding	NP_001186814.1;NP_057484.4	3.1
SNRPF	small nuclear ribonucleoprotein polypeptide F	protein_coding	NP_003086.1	3.1
LOC100996643	methylenetetrahydrofolate dehydrogenase (NADP	ranscribed_pseudogen	.	3.1

ZC3H13	zinc finger CCCH-type containing 13	protein_coding	NP_001070256.1;NP_001317493.1;	3.1
LTB4R	leukotriene B4 receptor	protein_coding	NP_001137391.1;NP_858043.1	3.1
EMSY	EMSY transcriptional repressor, BRCA2 interacti	protein_coding	NP_001287871.1;NP_001287872.1;	3.1
HBP1	HMG-box transcription factor 1	protein_coding	NP_001231191.1;NP_036389.2;XP_	3.1
LAMA3	laminin subunit alpha 3	protein_coding	NP_000218.3;NP_001121189.2;NP_	3.1
CCDC93	coiled-coil domain containing 93	protein_coding	NP_061917.3;XP_006712663.1;XP_	3.1
RORA	RAR related orphan receptor A	protein_coding	NP_002934.1;NP_599022.1;NP_59	3.1
LOC105376236	uncharacterized LOC105376236, transcript varian	lncRNA	.	3.1
LOC105378929	uncharacterized LOC105378929	lncRNA	.	3.1
DNAJB9	DnaJ heat shock protein family (Hsp40) member E	protein_coding	NP_036460.1	3.1
C2orf92	chromosome 2 open reading frame 92	protein_coding	NP_001338297.1;XP_024308873.1;	3.1
EPG5	ectopic P-granules autophagy protein 5 homolog	protein_coding	NP_066015.2;XP_011524424.1;XP_	3.1
LINC00565	long intergenic non-protein coding RNA 565	lncRNA	.	3.1
3C1D7-LOC1001303	TBC1D7-LOC100130357 readthrough	protein_coding	NP_001305738.1	3.1
ZNF285	zinc finger protein 285	protein_coding	NP_001278417.1;NP_001278418.1;	3.1
LOC105376000	uncharacterized LOC105376000	lncRNA	.	3.1
LOC105372989	uncharacterized LOC105372989, transcript varian	lncRNA	.	3.1
LOC100506672	cytochrome c oxidase subunit 7C pseudogene	pseudogene	.	3.1
REL	REL proto-oncogene, NF-kB subunit	protein_coding	NP_001278675.1;NP_002899.1;XP_	3.1
CD58	CD58 molecule	protein_coding	NP_001138294.1;NP_001770.1;XP_	3.1
G0S2	G0/G1 switch 2	protein_coding	NP_056529.1	3.1
ANXA2R	annexin A2 receptor	protein_coding	NP_001014301.1;NP_001369281.1	3.1
GUSBP3	GUSB pseudogene 3	ranscribed_pseudogen	.	3.1
LOC105377806	uncharacterized LOC105377806	lncRNA	.	3.1
SPAG5-AS1	SPAG5 antisense RNA 1	lncRNA	.	3.1
NAMPT	nicotinamide phosphoribosyltransferase	protein_coding	NP_005737.1	3.1
DDX42	DEAD-box helicase 42	protein_coding	NP_031398.2;NP_987095.1;XP_00	3.1
SUGT1P2	SUGT1 pseudogene 2	pseudogene	.	3.1
SNORA48	small nucleolar RNA, H/ACA box 48	snoRNA	.	3.1
PSD3	pleckstrin and Sec7 domain containing 3	protein_coding	NP_001349748.1;NP_056125.3;NP_	3.1
WDPCP	WD repeat containing planar cell polarity effector	protein_coding	NP_001036157.1;NP_001340973.1;	3.1
LOC646214	p21 (RAC1) activated kinase 2 pseudogene	ranscribed_pseudogen	.	3.1
LOC107985373	uncharacterized LOC107985373	lncRNA	.	3.1
ZNF660	zinc finger protein 660	protein_coding	NP_775929.2	3.1
RPS3AP3	RPS3A pseudogene 3	pseudogene	.	3.1
LOC107985957	uncharacterized LOC107985957	lncRNA	.	3.1
LOC105375655	uncharacterized LOC105375655	lncRNA	.	3.1
ZNF638	zinc finger protein 638	protein_coding	NP_001014972.1;NP_001239541.1;	3.1
RTP4	receptor transporter protein 4	protein_coding	NP_071430.2	3.1
LOC107984210	uncharacterized LOC107984210, transcript varian	lncRNA	.	3.1
RRN3P1	RRN3 pseudogene 1	ranscribed_pseudogen	.	3.1
LOC107984993	uncharacterized LOC107984993	lncRNA	.	3.1
LRRC37A3	leucine rich repeat containing 37 member A3	protein_coding	NP_001290184.1;NP_955372.2	3.1
OR2L1P	olfactory receptor family 2 subfamily L member 1	ranscribed_pseudogen	.	3.1
CBWD3	COBW domain containing 3	protein_coding	NP_001278750.1;NP_001365042.1;	3.1
LOC107985675	uncharacterized LOC107985675, transcript varian	lncRNA	.	3.1
LOC105376844	uncharacterized LOC105376844, transcript varian	lncRNA	.	3.1
ARHGEF9	Cdc42 guanine nucleotide exchange factor 9	protein_coding	NP_001166950.1;NP_001166951.1;	3.1
RBM44	RNA binding motif protein 44	protein_coding	NP_001073973.2;XP_005246132.1;	3.1
SH3YL1	SH3 and SYLF domain containing 1	protein_coding	NP_001153069.1;NP_001269611.1;	3.1
LOC112268259	uncharacterized LOC112268259	lncRNA	.	3.1
PABPC1L	poly(A) binding protein cytoplasmic 1 like	protein_coding	NP_001359108.1	3.1
C2CD6	C2 calcium dependent domain containing 6	protein_coding	NP_001161688.1;NP_001161689.1;	3.1
ZNF782	zinc finger protein 782	protein_coding	NP_001001662.1;NP_001333920.1;	3.1
PDE4D	phosphodiesterase 4D	protein_coding	NP_001098101.1;NP_001159371.1;	3.1
LINC02564	long intergenic non-protein coding RNA 2564	lncRNA	.	3.1
ZC3H8	zinc finger CCCH-type containing 8	protein_coding	NP_115883.2;XP_016860596.1;XP_	3.1
ZNF778	zinc finger protein 778	protein_coding	NP_001188336.1;NP_001365810.1;	3.1
SHC3	SHC adaptor protein 3	protein_coding	NP_058544.3;XP_011517087.1;XP_	3.1
LOC107984984	uncharacterized LOC107984984, transcript varian	lncRNA	.	3.1
RPS3AP34	RPS3A pseudogene 34	pseudogene	.	3.1
MCOLN3	mucolipin 3	protein_coding	NP_001240622.1;NP_060768.8;XP_	3.1
ZNF682	zinc finger protein 682	protein_coding	NP_001070817.1;NP_149973.1;XP_	3.1
CLDN1	claudin 1	protein_coding	NP_066924.1	3.1
LOC105374296	uncharacterized LOC105374296	lncRNA	.	3.1
TUBE1	tubulin epsilon 1	protein_coding	NP_057346.1;XP_011534177.1;XP_	3.1
LOC105376041	uncharacterized LOC105376041, transcript varian	lncRNA	.	3.1
LOC105373887	uncharacterized LOC105373887	lncRNA	.	3.1
ZNF697	zinc finger protein 697	protein_coding	NP_001073939.1;XP_005271372.1;	3.1
GUSBP16	GUSB pseudogene 16	ranscribed_pseudogen	.	3.1
SNORA46	small nucleolar RNA, H/ACA box 46	snoRNA	.	3.1

PHF14	PHD finger protein 14	protein_coding	NP_001007158.1;NP_055475.2	3.1
KDM7A	lysine demethylase 7A	protein_coding	NP_085150.1;XP_011514889.1	3.1
LOC102723564	uncharacterized LOC102723564, transcript varian	lncRNA	.	3.1
LOC101929356	uncharacterized LOC101929356, transcript varian	lncRNA	.	3.1
LOC105376039	uncharacterized LOC105376039, transcript varian	lncRNA	.	3.0
TTLL5	tubulin tyrosine ligase like 5	protein_coding	NP_055887.3	3.0
SLC7A11	solute carrier family 7 member 11	protein_coding	NP_055146.1;XP_011530104.1	3.0
ALMS1-IT1	ALMS1 intronic transcript 1	lncRNA	.	3.0
PLSCR1	phospholipid scramblase 1	protein_coding	NP_001350801.1;NP_001350803.1;	3.0
ECI2-DT	ECI2 divergent transcript	lncRNA	.	3.0
PRR16	proline rich 16	protein_coding	NP_001287712.1;NP_001295016.1;	3.0
SVEP1	sushi, von Willebrand factor type A, EGF and pen	protein_coding	NP_699197.3	3.0
LOC105375942	uncharacterized LOC105375942	lncRNA	.	3.0
HMGN5	high mobility group nucleosome binding domain 5	protein_coding	NP_110390.1	3.0
PCCA	propionyl-CoA carboxylase subunit alpha	protein_coding	NP_000273.2;NP_001121164.1;NP_	3.0
RPSAP41	ribosomal protein SA pseudogene 41	pseudogene	.	3.0
MTHFD1L	methylenetetrahydrofolate dehydrogenase (NADP	protein_coding	NP_001229696.1;NP_001229697.1;	3.0
CEACAM1	CEA cell adhesion molecule 1	protein_coding	NP_001020083.1;NP_001171742.1;	3.0
FAM221A	family with sequence similarity 221 member A	protein_coding	NP_001120836.1;NP_001120837.1;	3.0
NOL8	nucleolar protein 8	protein_coding	NP_001243323.1;NP_001317651.1;	3.0
LYPLAL1	lysophospholipase like 1	protein_coding	NP_001287698.1;NP_001287699.1;	3.0
HECW2-AS1	HECW2 antisense RNA 1	lncRNA	.	3.0
LOC107985915	uncharacterized LOC107985915, transcript varian	lncRNA	.	3.0
SCART1	scavenger receptor family member expressed on T	ranscribed_pseudogen	.	3.0
WDR27	WD repeat domain 27	protein_coding	NP_001189479.1;NP_001337552.1;	3.0
MPDZ	multiple PDZ domain crumbs cell polarity comple	protein_coding	NP_001248335.1;NP_001248336.1;	3.0
LOC105379016	uncharacterized LOC105379016, transcript varian	lncRNA	.	3.0
USP53	ubiquitin specific peptidase 53	protein_coding	NP_001358324.1;NP_001358325.1;	3.0
NOXRED1	NADP dependent oxidoreductase domain containi	protein_coding	NP_001106946.1;XP_005267387.1;	3.0
NAP1L4P1	nucleosome assembly protein 1 like 4 pseudogene	pseudogene	.	3.0
TVP23C	trans-golgi network vesicle protein 23 homolog C	protein_coding	NP_001128508.1;NP_660344.2	3.0
UTP25	UTP25 small subunit processor component	protein_coding	NP_055203.4;XP_006711338.1;XP_	3.0
LOC112268225	uncharacterized LOC112268225	lncRNA	.	3.0
LOC100133315	XRCC1 N-terminal domain containing 1-like	protein_coding	NP_001362776.1;NP_001362777.1	3.0
GUSBP9	GUSB pseudogene 9	ranscribed_pseudogen	.	3.0
MIR1236	hsa-miR-1236-3p	.	.	3.0
LOC105376154	uncharacterized LOC105376154, transcript varian	lncRNA	.	3.0
SNHG28	small nucleolar RNA host gene 28	lncRNA	.	3.0
CTNNA1P1	catenin alpha 1 pseudogene 1	pseudogene	.	3.0
LOC105375924	uncharacterized LOC105375924, transcript varian	lncRNA	.	3.0
OXNAD1	oxidoreductase NAD binding domain containing 1	protein_coding	NP_001317599.1;NP_001317600.1;	3.0
CSPP1	centrosome and spindle pole associated protein 1	protein_coding	NP_001278268.1;NP_001350060.1;	3.0
TRBV7-3	T cell receptor beta variable 7-3	V_segment	.	3.0
CFH	complement factor H	protein_coding	NP_000177.2;NP_001014975.1;XP_	3.0
HELB	DNA helicase B	protein_coding	NP_001357214.1;NP_387467.2;XP_	3.0
GPCPD1	glycerophosphocholine phosphodiesterase 1	protein_coding	NP_062539.1;XP_005260815.1;XP_	3.0
C5orf63	chromosome 5 open reading frame 63	protein_coding	NP_001157950.1;NP_001157951.1;	3.0
PFKFB4	6-phosphofructo-2-kinase/fructose-2,6-biphosphat	protein_coding	NP_001304063.1;NP_001304064.1;	3.0
LINC02762	long intergenic non-protein coding RNA 2762	lncRNA	.	3.0
ACAT2	acetyl-CoA acetyltransferase 2	protein_coding	NP_001290182.1;NP_005882.2	3.0
REP15	RAB15 effector protein	protein_coding	NP_001025045.3	3.0
RABGAP1L	RAB GTPase activating protein 1 like	protein_coding	NP_001030307.1;NP_001230692.1;	3.0
PCBP2-OT1	PCBP2 overlapping transcript 1	lncRNA	.	3.0
DMTF1	cyclin D binding myb like transcription factor 1	protein_coding	NP_001135798.1;NP_001135799.1;	3.0
ZNF75D	zinc finger protein 75D	protein_coding	NP_001171992.1;NP_009062.2;XP_	3.0
RPL7AP66	ribosomal protein L7a pseudogene 66	pseudogene	.	3.0
LOC112267941	uncharacterized LOC112267941	lncRNA	.	3.0
LOC105371024	uncharacterized LOC105371024, transcript varian	lncRNA	.	3.0
MCEE	methylmalonyl-CoA epimerase	protein_coding	NP_115990.3;XP_005264670.1	3.0
LINC01572	long intergenic non-protein coding RNA 1572	lncRNA	.	3.0
LOC105376001	uncharacterized LOC105376001	lncRNA	.	3.0
ATP1B1P1	ATPase Na+/K+ transporting subunit beta 1 pseud	pseudogene	.	3.0
ATG16L2	autophagy related 16 like 2	protein_coding	NP_001305695.1;NP_203746.1;XP_	3.0
LRP8	LDL receptor related protein 8	protein_coding	NP_001018064.1;NP_004622.2;NP_	3.0
VPS13B-DT	VPS13B divergent transcript	lncRNA	.	3.0
RPS14P2	ribosomal protein S14 pseudogene 2	pseudogene	.	3.0
RBM6	RNA binding motif protein 6	protein_coding	NP_001161054.1;NP_001336119.1;	3.0
SETD5	SET domain containing 5	protein_coding	NP_001073986.1;NP_001278972.1;	3.0
FSD1L	fibronectin type III and SPRY domain containing	protein_coding	NP_001138785.1;NP_001274120.1;	3.0
ARMCX4	armadillo repeat containing X-linked 4	protein_coding	NP_001243084.2	3.0
LOC100652840	M-phase phosphoprotein 10 (U3 small nucleolar ri	pseudogene	.	3.0

VWDE	von Willebrand factor D and EGF domains	protein_coding	NP_001129396.1;NP_001333901.1;	3.0
ZBTB49	zinc finger and BTB domain containing 49	protein_coding	NP_001317554.1;NP_660334.3;XP_	3.0
MYH15	myosin heavy chain 15	protein_coding	NP_055796.1;XP_011510861.1;XP_	3.0
TASP1	taspase 1	protein_coding	NP_001310531.1;NP_001310532.1;	3.0
CNPY3-GNMT	CNPY3-GNMT readthrough	protein_coding	NP_001305785.1;NP_001305786.1;	3.0
NPIPB11	nuclear pore complex interacting protein family m	protein_coding	NP_001297066.2;XP_024306188.1;	3.0
LOC102724642	anaphase-promoting complex subunit 1-like	protein_coding	XP_024309048.1	3.0
LOC100130357	uncharacterized LOC100130357	lncRNA	.	3.0
LOC107986437	uncharacterized LOC107986437, transcript varian	lncRNA	.	3.0
FUNDC2P1	FUN14 domain containing 2 pseudogene 1	pseudogene	.	3.0
ZNF470	zinc finger protein 470	protein_coding	NP_001001668.3;XP_016882290.1	3.0
KIAA1109	KIAA1109	protein_coding	NP_056127.2;XP_005263339.1;XP_	3.0
LOC107985193	uncharacterized LOC107985193	lncRNA	.	3.0
PLPP3	phospholipid phosphatase 3	protein_coding	NP_003704.3	3.0
NDUFS5	NADH:ubiquinone oxidoreductase subunit S5	protein_coding	NP_001171908.1;NP_004543.1	3.0
AREG	amphiregulin	protein_coding	NP_001648.1	3.0
ISG20	interferon stimulated exonuclease gene 20	protein_coding	NP_001290162.1;NP_001290163.1;	3.0
SYNE2	spectrin repeat containing nuclear envelope protei	protein_coding	NP_055995.4;NP_878914.1;NP_87	3.0
ZNF767P	zinc finger family member 767, pseudogene	ranscribed_pseudogen	.	3.0
ANAPC1P3	ANAPC1 pseudogene 3	pseudogene	.	3.0
EGFEM1P	EGF like and EMI domain containing 1, pseudoge	ranscribed_pseudogen	.	3.0
LOC112268422	uncharacterized LOC112268422	lncRNA	.	3.0
MMP1	matrix metallopeptidase 1	protein_coding	NP_001139410.1;NP_002412.1	3.0
AGAP14P	ArfGAP with GTPase domain, ankyrin repeat and	ranscribed_pseudogen	.	3.0
LOC105377929	uncharacterized LOC105377929	lncRNA	.	3.0
ADAT2	adenosine deaminase tRNA specific 2	protein_coding	NP_001273188.1;NP_872309.2;XP_	3.0
NCF2	neutrophil cytosolic factor 2	protein_coding	NP_000424.2;NP_001121123.1;NP_	2.9
RBMS2P1	RNA binding motif single stranded interacting pro	pseudogene	.	2.9
POLQ	DNA polymerase theta	protein_coding	NP_955452.3	2.9
ANKRD44-IT1	ANKRD44 intronic transcript 1	lncRNA	.	2.9
TMEM39A	transmembrane protein 39A	protein_coding	NP_060736.1;XP_005247635.1;XP_	2.9
MSS51	MSS51 mitochondrial translational activator	protein_coding	NP_001019764.1	2.9
CD163L1	CD163 molecule like 1	protein_coding	NP_001284579.1;NP_777601.3;XP_	2.9
CHP1P2	CHP1 pseudogene 2	ranscribed_pseudogen	.	2.9
LOC731075	uncharacterized LOC731075, transcript variant X2	lncRNA	.	2.9
LOC100996333	G protein-coupled receptor 125 pseudogene	pseudogene	.	2.9
LOC105379034	uncharacterized LOC105379034, transcript varian	lncRNA	.	2.9
LOC105375513	uncharacterized LOC105375513, transcript varian	lncRNA	.	2.9
NPIPB10P	nuclear pore complex interacting protein family, n	pseudogene	.	2.9
CCR4	C-C motif chemokine receptor 4	protein_coding	NP_005499.1;XP_016861176.1	2.9
ATR	ATR serine/threonine kinase	protein_coding	NP_001175.2;NP_001341508.1;XP_	2.9
PDCD5	programmed cell death 5	protein_coding	NP_004699.1;XP_005259449.1	2.9
CSGALNACT2	chondroitin sulfate N-acetylgalactosaminyltransfe	protein_coding	NP_001306583.1;NP_001306585.1;	2.9
CATSPER2P1	cation channel sperm associated 2 pseudogene 1	ranscribed_pseudogen	.	2.9
LINC01599	long intergenic non-protein coding RNA 1599	lncRNA	.	2.9
MIG7	mig-7	lncRNA	.	2.9
NCEH1	neutral cholesterol ester hydrolase 1	protein_coding	NP_001139748.2;NP_001139749.1;	2.9
PARGP1-AGAP4	PARGP1-AGAP4 readthrough	ranscribed_pseudogen	.	2.9
PCID2	PCI domain containing 2	protein_coding	NP_001120674.1;NP_001120675.1;	2.9
LINC00670	long intergenic non-protein coding RNA 670	lncRNA	.	2.9
SEPTIN14P5	septin 14 pseudogene 5	pseudogene	.	2.9
MSANTD2	Myb/SANT DNA binding domain containing 2	protein_coding	NP_001294956.1;NP_001299848.1;	2.9
URGCP-MRPS24	URGCP-MRPS24 readthrough	protein_coding	NP_001191800.1	2.9
CWC27	CWC27 spliceosome associated cyclophilin	protein_coding	NP_001284573.1;NP_001284574.1;	2.9
TBC1D8B	TBC1 domain family member 8B	protein_coding	NP_060222.2;NP_942582.1;XP_00	2.9
LOC107986379	uncharacterized LOC107986379	lncRNA	.	2.9
PLA2G4A	phospholipase A2 group IVA	protein_coding	NP_001298122.2;NP_077734.2;XP_	2.9
RAB5CP2	RAB5C, member RAS oncogene family pseudoge	pseudogene	.	2.9
LOC107986718	uncharacterized LOC107986718	lncRNA	.	2.9
GUSBP15	GUSB pseudogene 15	ranscribed_pseudogen	.	2.9
TDRD7	tudor domain containing 7	protein_coding	NP_001289813.1;NP_055105.2;XP_	2.9
LRRFIP1	LRR binding FLII interacting protein 1	protein_coding	NP_001131022.1;NP_001131023.1;	2.9
LOC112268030	uncharacterized LOC112268030	lncRNA	.	2.9
HERPUD1	homocysteine inducible ER protein with ubiquitin	protein_coding	NP_001010989.1;NP_001259032.1;	2.9
CFB	complement factor B	protein_coding	NP_001701.2	2.9
CYP27B1	cytochrome P450 family 27 subfamily B member	protein_coding	NP_000776.1	2.9
PACRGL	parkin coregulated like	protein_coding	NP_001124199.1;NP_001245274.1;	2.9
PIGAP1	phosphatidylinositol glycan anchor biosynthesis cl	pseudogene	.	2.9
C11orf91	chromosome 11 open reading frame 91	protein_coding	NP_001160164.1;XP_016872542.1	2.9
LINC01179	long intergenic non-protein coding RNA 1179	lncRNA	.	2.9
ZNF792	zinc finger protein 792	protein_coding	NP_787068.3	2.9

LOC105376093	uncharacterized LOC105376093, transcript varian	lncRNA	.	2.9
SDHAP3	succinate dehydrogenase complex flavoprotein sul	ranscribed_pseudogen	.	2.9
RCAN1	regulator of calcineurin 1	protein_coding	NP_001272318.1;NP_001272320.2;	2.9
LOC100287036	uncharacterized LOC100287036	lncRNA	.	2.9
LOC107984492	uncharacterized LOC107984492	lncRNA	.	2.9
NBEA	neurobeachin	protein_coding	NP_001191126.1;NP_001366174.1;	2.9
ZNF84	zinc finger protein 84	protein_coding	NP_001120844.1;NP_001276900.1;	2.9
ANXA10	annexin A10	protein_coding	NP_009124.2;XP_011529873.1	2.9
CNST	consortin, connexin sorting protein	protein_coding	NP_001132931.1;NP_689822.2;XP_	2.9
CCDC15	coiled-coil domain containing 15	protein_coding	NP_079280.2;XP_016873835.1;XP_	2.9
TCP11L2	t-complex 11 like 2	protein_coding	NP_001273191.1;NP_689985.1;XP_	2.9
GAD1	glutamate decarboxylase 1	protein_coding	NP_000808.2;NP_038473.2;XP_00	2.9
AGAP10P	ArfGAP with GTPase domain, ankyrin repeat and	ranscribed_pseudogen	.	2.9
ABL2	ABL proto-oncogene 2, non-receptor tyrosine kin	protein_coding	NP_001129472.1;NP_001129473.1;	2.9
ASPH	aspartate beta-hydroxylase	protein_coding	NP_001158222.1;NP_001158223.1;	2.9
RPL7AP60	ribosomal protein L7a pseudogene 60	pseudogene	.	2.9
LOC105377271	uncharacterized LOC105377271, transcript varian	lncRNA	.	2.9
ZEB1	zinc finger E-box binding homeobox 1	protein_coding	NP_001121600.1;NP_001167564.1;	2.9
ADGRA3	adhesion G protein-coupled receptor A3	protein_coding	NP_660333.2;XP_005248194.1;XP_	2.9
SCAPER	S-phase cyclin A associated protein in the ER	protein_coding	NP_001139395.1;NP_001339938.1;	2.9
ZMAT1	zinc finger matrin-type 1	protein_coding	NP_001011657.2;NP_001269329.1;	2.9
SNORD30	small nucleolar RNA, C/D box 30	snoRNA	.	2.9
N6AMT1	N-6 adenine-specific DNA methyltransferase 1	protein_coding	NP_037372.4;NP_877426.4	2.9
LOC105373794	uncharacterized LOC105373794	lncRNA	.	2.9
TIMM10B	translocase of inner mitochondrial membrane 10B	protein_coding	NP_036324.1	2.9
NKAPP1	NFKB activating protein pseudogene 1	ranscribed_pseudogen	.	2.9
NABP1	nucleic acid binding protein 1	protein_coding	NP_001026886.1;NP_001241665.1	2.9
PWAR6	Prader Willi/Angelman region RNA 6	lncRNA	.	2.9
LOC645513	septin 7 pseudogene	ranscribed_pseudogen	.	2.9
LOC105369180	uncharacterized LOC105369180	lncRNA	.	2.9
LOC105369301	uncharacterized LOC105369301, transcript varian	lncRNA	.	2.9
COX10-AS1	COX10 antisense RNA 1	lncRNA	.	2.9
NPIPB12	nuclear pore complex interacting protein family m	protein_coding	NP_001342330.1	2.9
ZNF107	zinc finger protein 107	protein_coding	NP_001013768.1;NP_001269288.1;	2.9
SGIP1	SH3GL interacting endocytic adaptor 1	protein_coding	NP_001295132.1;NP_001337146.1;	2.9
PGAM1P8	phosphoglycerate mutase 1 pseudogene 8	pseudogene	.	2.9
AGAP1-IT1	AGAP1 intronic transcript 1	lncRNA	.	2.9
WRN	WRN RecQ like helicase	protein_coding	NP_000544.2;XP_011542941.1;XP_	2.9
MAST4	microtubule associated serine/threonine kinase fan	protein_coding	NP_001158136.1;NP_001277155.1;	2.9
STK36	serine/threonine kinase 36	protein_coding	NP_001230242.1;NP_001356352.1;	2.9
LOC107986704	.	pseudogene	.	2.9
AK5	adenylate kinase 5	protein_coding	NP_036225.2;NP_777283.1;XP_00	2.9
LOC100507379	RNA binding motif protein 4 pseudogene	pseudogene	.	2.9
LOC107985798	histone-lysine N-methyltransferase 2C-like	protein_coding	XP_024309039.1	2.9
SNORD36A	small nucleolar RNA, C/D box 36A	snoRNA	.	2.9
ZNF521	zinc finger protein 521	protein_coding	NP_001295154.1;NP_056276.1;XP_	2.9
DENND4A	DENN domain containing 4A	protein_coding	NP_001138295.1;NP_001307764.1;	2.9
TTTY14	testis-specific transcript, Y-linked 14	lncRNA	.	2.9
LOC105379250	uncharacterized LOC105379250	lncRNA	.	2.9
LOC100507103	uncharacterized LOC100507103, transcript varian	lncRNA	.	2.9
LGALSL-DT	LGALSL divergent transcript, transcript variant X	lncRNA	.	2.9
LINC01094	long intergenic non-protein coding RNA 1094	lncRNA	.	2.9
SEPTIN7P2	septin 7 pseudogene 2	ranscribed_pseudogen	.	2.9
LOC653513	phosphodiesterase 4D interacting protein-like	ranscribed_pseudogen	.	2.9
RAI14	retinoic acid induced 14	protein_coding	NP_001138992.1;NP_001138993.1;	2.9
CCDC82	coiled-coil domain containing 82	protein_coding	NP_001305665.1;NP_001305666.1;	2.9
GAPLINC	gastric adenocarcinoma associated, positive CD44	lncRNA	.	2.9
CEP192	centrosomal protein 192	protein_coding	NP_115518.3;XP_005258164.1;XP_	2.9
KIAA0825	KIAA0825	protein_coding	NP_001139150.1;NP_775936.1;XP_	2.9
EXOC4	exocyst complex component 4	protein_coding	NP_001032203.1;NP_068579.3;XP_	2.9
UTP14C	UTP14C small subunit processome component	protein_coding	NP_067677.4	2.8
ZNF202	zinc finger protein 202	protein_coding	NP_001288708.1;NP_001288709.1;	2.8
LOC283299	uncharacterized LOC283299	lncRNA	.	2.8
MCPH1	microcephalin 1	protein_coding	NP_001166045.1;NP_001166046.1;	2.8
LOC105371009	uncharacterized LOC105371009, transcript varian	lncRNA	.	2.8
ZNF385D	zinc finger protein 385D	protein_coding	NP_078973.1;XP_011532424.1;XP_	2.8
OAS3	2'-5'-oligoadenylate synthetase 3	protein_coding	NP_006178.2;XP_005253946.1;XP_	2.8
HOXB-AS1	HOXB cluster antisense RNA 1	lncRNA	.	2.8
RPL21P95	ribosomal protein L21 pseudogene 95	pseudogene	.	2.8
MLH3	mutL homolog 3	protein_coding	NP_001035197.1;NP_055196.2;XP_	2.8
HECW2	HECT, C2 and WW domain containing E3 ubiqui	protein_coding	NP_001291769.1;NP_001335697.1;	2.8

MCM3AP-AS1	MCM3AP antisense RNA 1	lncRNA	.	2.8
LOC105370841	uncharacterized LOC105370841	lncRNA	.	2.8
N4BP2L2	NEDD4 binding protein 2 like 2	protein_coding	NP_001265361.1;NP_001307765.1;	2.8
MACF1	microtubule actin crosslinking factor 1	protein_coding	NP_036222.3	2.8
GTF2IP23	general transcription factor IIi pseudogene 23	ranscribed_pseudogen	.	2.8
KRBOX4	KRAB box domain containing 4	protein_coding	NP_001123370.1;NP_001123371.1;	2.8
RNPC3	RNA binding region (RNP1, RRM) containing 3	protein_coding	NP_060089.1	2.8
LOC107985736	uncharacterized LOC107985736	lncRNA	.	2.8
R3HCC1L	R3H domain and coiled-coil containing 1 like	protein_coding	NP_001243548.2;NP_001243549.1;	2.8
DPY19L3	dpy-19 like C-mannosyltransferase 3	protein_coding	NP_001166245.1;NP_997208.2;XP_	2.8
SNORA58B	small nucleolar RNA, H/ACA box 58B	snoRNA	.	2.8
PAQR6	progestin and adipoQ receptor family member 6	protein_coding	NP_001259033.1;NP_001259034.1;	2.8
LOC105375434	uncharacterized LOC105375434, transcript varian	lncRNA	.	2.8
LETM2	leucine zipper and EF-hand containing transmemb	protein_coding	NP_001186588.1;NP_001186589.1;	2.8
COL4A3	collagen type IV alpha 3 chain	protein_coding	NP_000082.2;XP_005246334.1;XP_	2.8
ANKRD28	ankyrin repeat domain 28	protein_coding	NP_001182027.1;NP_001182028.1;	2.8
MIR31HG	MIR31 host gene	lncRNA	.	2.8
AGBL2	ATP/GTP binding protein like 2	protein_coding	NP_079059.2;XP_005253195.1;XP_	2.8
CCNB1IP1	cyclin B1 interacting protein 1	protein_coding	NP_067001.3;NP_878269.1;NP_87	2.8
PRPF18	pre-mRNA processing factor 18	protein_coding	NP_003666.1;XP_011518056.1;XP_	2.8
DISP1	dispatched RND transporter family member 1	protein_coding	NP_001337559.1;NP_001356523.1;	2.8
C1RL-AS1	C1RL antisense RNA 1	lncRNA	.	2.8
LOC102724078	uncharacterized LOC102724078, transcript varian	lncRNA	.	2.8
SYCP2L	synaptonemal complex protein 2 like	protein_coding	NP_001035364.2	2.8
API5P2	apoptosis inhibitor 5 pseudogene 2	pseudogene	.	2.8
LOC105379395	uncharacterized LOC105379395	lncRNA	.	2.8
LOC112268262	uncharacterized LOC112268262	lncRNA	.	2.8
LOC105376253	uncharacterized LOC105376253	lncRNA	.	2.8
ZZZ3	zinc finger ZZ-type containing 3	protein_coding	NP_001295166.1;NP_001363075.1;	2.8
ZNF195	zinc finger protein 195	protein_coding	NP_001123991.1;NP_001123992.1;	2.8
LOC107984986	uncharacterized LOC107984986	lncRNA	.	2.8
RPL13AP9	ribosomal protein L13a pseudogene 9	pseudogene	.	2.8
CEP95	centrosomal protein 95	protein_coding	NP_001303919.1;NP_612372.1;XP_	2.8
NR6A1	nuclear receptor subfamily 6 group A member 1	protein_coding	NP_001265475.1;NP_001480.3;NP_	2.8
LRBA	LPS responsive beige-like anchor protein	protein_coding	NP_001186211.2;NP_001351834.1;	2.8
LOC101929285	uncharacterized LOC101929285, transcript varian	lncRNA	.	2.8
NTAN1P2	N-terminal asparagine amidase pseudogene 2	pseudogene	.	2.8
PCBP1-AS1	PCBP1 antisense RNA 1	lncRNA	.	2.8
SMIM2-AS1	SMIM2 antisense RNA 1	lncRNA	.	2.8
LOC107986785	uncharacterized LOC107986785	lncRNA	.	2.8
ZSWIM6	zinc finger SWIM-type containing 6	protein_coding	NP_065979.1;XP_016865166.1;XP_	2.8
ZFPM2	zinc finger protein, FOG family member 2	protein_coding	NP_001349765.1;NP_001349766.1;	2.8
NPIPB6	nuclear pore complex interacting protein family m	protein_coding	NP_001269453.1;XP_005255798.1;	2.8
CEP135	centrosomal protein 135	protein_coding	NP_079285.2;XP_005265845.1;XP_	2.8
ENDOD1	endonuclease domain containing 1	protein_coding	NP_055851.1	2.8
FNDC3A	fibronectin type III domain containing 3A	protein_coding	NP_001073141.1;NP_001265367.1;	2.8
LCT	lactase	protein_coding	NP_002290.2;XP_016859577.1	2.8
ENTPD4	ectonucleoside triphosphate diphosphohydrolase 4	protein_coding	NP_001122402.1;NP_004892.1	2.8
LOC107986337	uncharacterized LOC107986337	lncRNA	.	2.8
LOC107986188	uncharacterized LOC107986188	lncRNA	.	2.8
LINC02569	long intergenic non-protein coding RNA 2569	lncRNA	.	2.8
SRSF11	serine and arginine rich splicing factor 11	protein_coding	NP_001177916.1;NP_001337534.1;	2.8
ITPR2	inositol 1,4,5-trisphosphate receptor type 2	protein_coding	NP_002214.2;XP_016874755.1;XP_	2.8
SPANXN3	SPANX family member N3	protein_coding	NP_001009609.2;XP_016884753.1	2.8
BTN2A3P	butyrophilin subfamily 2 member A3, pseudogene	ranscribed_pseudogen	.	2.8
OR2B6	olfactory receptor family 2 subfamily B member 6	protein_coding	NP_036499.1	2.8
SLC4A7	solute carrier family 4 member 7	protein_coding	NP_001245308.1;NP_001245309.1;	2.8
LTV1	LTV1 ribosome biogenesis factor	protein_coding	NP_001316882.1;NP_116249.2	2.8
FAM155A	family with sequence similarity 155 member A	protein_coding	NP_001073865.1;XP_011519411.1	2.8
RPS2P47	ribosomal protein S2 pseudogene 47	pseudogene	.	2.8
GALK2	galactokinase 2	protein_coding	NP_001001556.1;NP_001275959.1;	2.8
WDR70	WD repeat domain 70	protein_coding	NP_001332927.1;NP_001332928.1;	2.8
COX6A1P2	cytochrome c oxidase subunit 6A1 pseudogene 2	pseudogene	.	2.8
EMP1	epithelial membrane protein 1	protein_coding	NP_001414.1;XP_024304645.1	2.8
OGT	O-linked N-acetylglucosamine (GlcNAc) transfera	protein_coding	NP_858058.1;NP_858059.1;XP_01	2.8
POC1B-AS1	POC1B antisense RNA 1	lncRNA	.	2.8
DPH6	diphthamine biosynthesis 6	protein_coding	NP_001135444.1;NP_542381.1;XP_	2.8
SPG11	SPG11 vesicle trafficking associated, spatacsin	protein_coding	NP_001153699.1;NP_079413.3;XP_	2.8
PRDM5	PR/SET domain 5	protein_coding	NP_001287752.1;NP_001287753.1;	2.8
GOLGA6L5P	golgin A6 family like 5, pseudogene	ranscribed_pseudogen	.	2.8
LINC00630	long intergenic non-protein coding RNA 630	lncRNA	.	2.8

NFXL1	nuclear transcription factor, X-box binding like 1	protein_coding	NP_001265552.1;NP_001265553.1;	2.8
UBE2V1P1	ubiquitin conjugating enzyme E2 V1 pseudogene	pseudogene	.	2.8
TFPI2	tissue factor pathway inhibitor 2	protein_coding	NP_001257932.1;NP_001257933.1;	2.8
BRIP1	BRCA1 interacting protein C-terminal helicase 1	protein_coding	NP_114432.2;XP_011523634.1;XP_	2.8
LINC00882	long intergenic non-protein coding RNA 882	lncRNA	.	2.8
LOC105377449	uncharacterized LOC105377449	lncRNA	.	2.8
LOC112268239	uncharacterized LOC112268239	lncRNA	.	2.8
CTSL	cathepsin L	protein_coding	NP_001244900.1;NP_001244901.1;	2.8
HERC1	HECT and RLD domain containing E3 ubiquitin p	protein_coding	NP_003913.3;XP_016878188.1;XP_	2.8
ZNF215	zinc finger protein 215	protein_coding	NP_001341782.1;NP_001341783.1;	2.8
BICD1	BICD cargo adaptor 1	protein_coding	NP_001003398.1;NP_001341115.1;	2.8
LOC107984347	uncharacterized LOC107984347	lncRNA	.	2.8
LOC107985021	flocculation protein FLO11-like	protein_coding	XP_016880993.1	2.8
TRIM38	tripartite motif containing 38	protein_coding	NP_006346.1;XP_005248856.1;XP_	2.8
NRCAM	neuronal cell adhesion molecule	protein_coding	NP_001032209.1;NP_001180511.1;	2.8
ZNF37BP	zinc finger protein 37B, pseudogene	ranscribed_pseudogen	.	2.8
SREK1	splicing regulatory glutamic acid and lysine rich p	protein_coding	NP_001070667.1;NP_001257421.1;	2.8
SNORD5	small nucleolar RNA, C/D box 5	snoRNA	.	2.8
EFCAB2	EF-hand calcium binding domain 2	protein_coding	NP_001137415.1;NP_001277256.1;	2.8
C6orf163	chromosome 6 open reading frame 163	protein_coding	NP_001010868.2	2.8
LOC112268134	uncharacterized LOC112268134	lncRNA	.	2.8
LOC107984999	uncharacterized LOC107984999	lncRNA	.	2.8
SNHG21	small nucleolar RNA host gene 21	lncRNA	.	2.8
SYT14	synaptotagmin 14	protein_coding	NP_001139733.1;NP_001139734.1;	2.8
REV1	REV1 DNA directed polymerase	protein_coding	NP_001032961.1;NP_001308383.1;	2.7
SNTG2	syntrophin gamma 2	protein_coding	NP_061841.2;XP_011508668.1;XP_	2.7
PAX8-AS1	PAX8 antisense RNA 1	lncRNA	.	2.7
SCARNA18B	small Cajal body-specific RNA 18B	guide_RNA	.	2.7
PWAR5	Prader Willi/Angelman region RNA 5	lncRNA	.	2.7
JMJD1C	jumonji domain containing 1C	protein_coding	NP_001269877.1;NP_001305082.1;	2.7
SNORD19	small nucleolar RNA, C/D box 19	snoRNA	.	2.7
LOC107986160	uncharacterized LOC107986160	lncRNA	.	2.7
KRT18P61	keratin 18 pseudogene 61	pseudogene	.	2.7
PLEKHH2	pleckstrin homology, MyTH4 and FERM domain	protein_coding	NP_742066.2;XP_016858840.1;XP_	2.7
USP34	ubiquitin specific peptidase 34	protein_coding	NP_055524.3	2.7
GNL2	G protein nucleolar 2	protein_coding	NP_001310552.1;NP_001310553.1;	2.7
PRRC2C	proline rich coiled-coil 2C	protein_coding	NP_055987.2;XP_005245072.1;XP_	2.7
TAOK3	TAO kinase 3	protein_coding	NP_001333416.1;NP_001333417.1;	2.7
CCDC18	coiled-coil domain containing 18	protein_coding	NP_001293005.1;NP_001365133.1;	2.7
NCKAP1L	NCK associated protein 1 like	protein_coding	NP_001171905.1;NP_005328.2	2.7
GNL3LP1	G protein nucleolar 3 like pseudogene 1	pseudogene	.	2.7
LINC00243	long intergenic non-protein coding RNA 243	lncRNA	.	2.7
MLLT10	MLLT10 histone lysine methyltransferase DOT1L	protein_coding	NP_001182555.1;NP_001182556.1;	2.7
LOC112267940	uncharacterized LOC112267940	protein_coding	XP_024302053.1	2.7
CA5BP1-CA5B	CA5BP1-CA5B readthrough	lncRNA	.	2.7
MBP	myelin basic protein	protein_coding	NP_001020252.1;NP_001020261.1;	2.7
ZNF33A	zinc finger protein 33A	protein_coding	NP_001265099.1;NP_001265100.1;	2.7
ETS2	ETS proto-oncogene 2, transcription factor	protein_coding	NP_001243224.1;NP_005230.1;XP_	2.7
MIR100HG	mir-100-let-7a-2-mir-125b-1 cluster host gene	lncRNA	.	2.7
PLEKHA5	pleckstrin homology domain containing A5	protein_coding	NP_001137293.2;NP_001177789.2;	2.7
LINC00444	long intergenic non-protein coding RNA 444, tran	lncRNA	.	2.7
ZNF224	zinc finger protein 224	protein_coding	NP_001308574.1;NP_037530.2;XP_	2.7
ST13P4	ST13, Hsp70 interacting protein pseudogene 4	ranscribed_pseudogen	.	2.7
CEP170	centrosomal protein 170	protein_coding	NP_001035863.1;NP_001035864.1;	2.7
SNORA51	small nucleolar RNA, H/ACA box 51	snoRNA	.	2.7
KRT18P37	keratin 18 pseudogene 37	pseudogene	.	2.7
ANKRD36B	ankyrin repeat domain 36B	protein_coding	NP_001340266.1;NP_079466.3;XP_	2.7
WSB1	WD repeat and SOCS box containing 1	protein_coding	NP_001335279.1;NP_056441.6;NP_	2.7
GPATCH2L	G-patch domain containing 2 like	protein_coding	NP_001308955.1;NP_001308956.1;	2.7
SERPINB8	serpin family B member 8	protein_coding	NP_001027018.1;NP_001263419.1;	2.7
PIGL	phosphatidylinositol glycan anchor biosynthesis cl	protein_coding	NP_004269.1;XP_011522382.1;XP_	2.7
SNHG15	small nucleolar RNA host gene 15	lncRNA	.	2.7
NEMP2	nuclear envelope integral membrane protein 2	protein_coding	NP_001136117.1;XP_005246269.1;	2.7
SRGAP1	SLIT-ROBO Rho GTPase activating protein 1	protein_coding	NP_001333130.1;NP_065813.1;XP_	2.7
TRIO	trio Rho guanine nucleotide exchange factor	protein_coding	NP_009049.2;XP_011512409.1;XP_	2.7
MMP24-AS1-EDEM2	MMP24-AS1-EDEM2 readthrough	protein_coding	NP_001341937.1	2.7
BACH1	BTB domain and CNC homolog 1	protein_coding	NP_001177.1;NP_996749.1	2.7
RHEBL1	RHEB like 1	protein_coding	NP_001290055.1;NP_653194.1	2.7
LPIN1	lipin 1	protein_coding	NP_001248356.1;NP_001248357.1;	2.7
LOC105369820	uncharacterized LOC105369820, transcript varian	lncRNA	.	2.7
FGGY	FGGY carbohydrate kinase domain containing	protein_coding	NP_001106882.1;NP_001231643.1;	2.7

EVC	EvC ciliary complex subunit 1	protein_coding	NP_001293019.1;NP_001293021.1;	2.7
CIR1	corepressor interacting with RBPJ, CIR1	protein_coding	NP_004873.3	2.7
LOC105373182	uncharacterized LOC105373182, transcript varian	lncRNA	.	2.7
MCTP1	multiple C2 and transmembrane domain containin	protein_coding	NP_001002796.1;NP_001284706.1;	2.7
TMEM81	transmembrane protein 81	protein_coding	NP_976310.1	2.7
LOC107986871	ubiquitin-conjugating enzyme E2 variant 1 pseudo	ranscribed_pseudogene	.	2.7
SIPA1L1	signal induced proliferation associated 1 like 1	protein_coding	NP_001271174.1;NP_001271175.1;	2.7
FOXG1-AS1	FOXG1 antisense RNA 1	lncRNA	.	2.7
LOC102724851	uncharacterized LOC102724851, transcript varian	lncRNA	.	2.7
LOC107984754	uncharacterized LOC107984754	lncRNA	.	2.7
NAPB	NSF attachment protein beta	protein_coding	NP_001269947.1;NP_001269949.1;	2.7
LOC102724081	uncharacterized LOC102724081, transcript varian	lncRNA	.	2.7
IL7R	interleukin 7 receptor	protein_coding	NP_002176.2;XP_005248356.1	2.7
GUSBP14	GUSB pseudogene 14	ranscribed_pseudogene	.	2.7
KDM6A	lysine demethylase 6A	protein_coding	NP_001278344.1;NP_001278345.1;	2.7
TBC1D32	TBC1 domain family member 32	protein_coding	NP_001354688.1;NP_001354689.1;	2.7
ERN1	endoplasmic reticulum to nucleus signaling 1	protein_coding	NP_001424.3;XP_016879836.1;XP_	2.7
ZNF175	zinc finger protein 175	protein_coding	NP_009078.1;XP_016882738.1	2.7
MICU3	mitochondrial calcium uptake family member 3	protein_coding	NP_001336739.1;NP_859074.1;XP_	2.7
ARRDC3-AS1	ARRDC3 antisense RNA 1	lncRNA	.	2.7
CD55	CD55 molecule (Cromer blood group)	protein_coding	NP_000565.1;NP_001108224.1;NP_	2.7
MIR3064	microRNA 3064	miRNA	.	2.7
TSEN2	tRNA splicing endonuclease subunit 2	protein_coding	NP_001138864.1;NP_001138865.1;	2.7
LXN	latexin	protein_coding	NP_064554.3;XP_016862365.1	2.7
CPED1	cadherin like and PC-esterase domain containing 1	protein_coding	NP_001099003.1;NP_079189.4;XP_	2.7
LRIG3	leucine rich repeats and immunoglobulin like dom	protein_coding	NP_001129523.1;NP_700356.2;XP_	2.7
FANCB	FA complementation group B	protein_coding	NP_001018123.1;NP_001311091.1;	2.7
LOC101927687	uncharacterized LOC101927687, transcript varian	lncRNA	.	2.7
LOC102606465	uncharacterized LOC102606465	lncRNA	.	2.7
UACA	uveal autoantigen with coiled-coil domains and an	protein_coding	NP_001008225.1;NP_060473.2;XP_	2.7
TMEM256-PLSCR3	TMEM256-PLSCR3 readthrough (NMD candidate	lncRNA	.	2.7
INTS6L	integrator complex subunit 6 like	protein_coding	NP_001338530.1;NP_001338532.1;	2.7
GUSBP13	GUSB pseudogene 13	pseudogene	.	2.7
SAMD15	sterile alpha motif domain containing 15	protein_coding	NP_001010860.1	2.7
RSKR	ribosomal protein S6 kinase related	protein_coding	NP_001167574.1	2.7
RPL7P24	ribosomal protein L7 pseudogene 24	pseudogene	.	2.7
ZNF112	zinc finger protein 112	protein_coding	NP_001076804.1;NP_001335210.1;	2.7
LOC102724748	uncharacterized LOC102724748, transcript varian	lncRNA	.	2.7
SETX	senataxin	protein_coding	NP_001338456.1;NP_001338457.1;	2.7
DTX3L	deltex E3 ubiquitin ligase 3L	protein_coding	NP_612144.1	2.7
CEP170P1	centrosomal protein 170 pseudogene 1	ranscribed_pseudogene	.	2.7
CLDN12	claudin 12	protein_coding	NP_001172001.1;NP_001172002.1;	2.7
IRF2	interferon regulatory factor 2	protein_coding	NP_002190.2;XP_024309802.1;XP_	2.7
MAGOH	mago homolog, exon junction complex subunit	protein_coding	NP_002361.1	2.7
KIF27	kinesin family member 27	protein_coding	NP_001258856.1;NP_001258857.1;	2.7
ZNF235	zinc finger protein 235	protein_coding	NP_004225.3;XP_005259469.1	2.7
INPP4B	inositol polyphosphate-4-phosphatase type II B	protein_coding	NP_001095139.1;NP_001317969.1;	2.7
LMCD1-AS1	LMCD1 antisense RNA 1	lncRNA	.	2.7
WTAPP1	Wilms tumor 1 associated protein pseudogene 1	ranscribed_pseudogene	.	2.7
UBA6-AS1	UBA6 antisense RNA 1 (head to head)	lncRNA	.	2.7
LOC643454	adaptor related protein complex 3 sigma 1 subunit	pseudogene	.	2.7
OVCH2	ovochymase 2	protein_coding	NP_001354892.1;NP_937828.3;XP_	2.7
LINC01291	long intergenic non-protein coding RNA 1291	lncRNA	.	2.7
SNORD14C	small nucleolar RNA, C/D box 14C	snoRNA	.	2.7
COA1	cytochrome c oxidase assembly factor 1 homolog	protein_coding	NP_001308126.1;NP_001308127.1;	2.7
SNORD14D	small nucleolar RNA, C/D box 14D	snoRNA	.	2.7
GRAMD1B	GRAM domain containing 1B	protein_coding	NP_001273492.1;NP_001273493.1;	2.7
RTTN	rotatin	protein_coding	NP_001305449.1;NP_775901.3;XP_	2.7
DCLRE1C	DNA cross-link repair 1C	protein_coding	NP_001029027.1;NP_001029029.1;	2.7
MLKL	mixed lineage kinase domain like pseudokinase	protein_coding	NP_001135969.1;NP_689862.1;XP_	2.7
LOC107986363	uncharacterized LOC107986363	lncRNA	.	2.7
MTHFD2L	methylenetetrahydrofolate dehydrogenase (NADP	protein_coding	NP_001004346.2;NP_001138450.1;	2.7
HMGA2	high mobility group AT-hook 2	protein_coding	NP_001287847.1;NP_001287848.1;	2.7
PDK1	pyruvate dehydrogenase kinase 1	protein_coding	NP_001265478.1;NP_002601.1;XP_	2.6
CWF19L2	CWF19 like cell cycle control factor 2	protein_coding	NP_689647.2;XP_011540922.1;XP_	2.6
AGAP5	ArfGAP with GTPase domain, ankyrin repeat and	protein_coding	NP_001137472.1	2.6
LOC112267943	uncharacterized LOC112267943	lncRNA	.	2.6
EZH1	enhancer of zeste 1 polycomb repressive complex	protein_coding	NP_001308008.1;NP_001308010.1;	2.6
CDKN2B-AS1	CDKN2B antisense RNA 1	lncRNA	.	2.6
SNHG6	small nucleolar RNA host gene 6	lncRNA	.	2.6
PATL2	PAT1 homolog 2	protein_coding	NP_001138584.1;NP_001317212.1;	2.6

BTF3P16	basic transcription factor 3 pseudogene 16	pseudogene	.	2.6
PRH1	proline rich protein HaeIII subfamily 1	protein_coding	NP_001278243.1;NP_001278244.1	2.6
LOC107985558	uncharacterized LOC107985558	lncRNA	.	2.6
GTF2IP12	general transcription factor IIi pseudogene 12	ranscribed_pseudogen	.	2.6
FCF1P1	FCF1 pseudogene 1	pseudogene	.	2.6
LOC107986918	uncharacterized LOC107986918	lncRNA	.	2.6
TASOR2	transcription activation suppressor family member	protein_coding	NP_001308712.1;NP_001308713.1;	2.6
LOC105374037	uncharacterized LOC105374037	lncRNA	.	2.6
COX7B	cytochrome c oxidase subunit 7B	protein_coding	NP_001857.1	2.6
LOC107986304	uncharacterized LOC107986304	lncRNA	.	2.6
LOC105374968	uncharacterized LOC105374968	lncRNA	.	2.6
CDRT4	CMT1A duplicated region transcript 4	protein_coding	NP_001191406.1	2.6
LOC105376577	uncharacterized LOC105376577	lncRNA	.	2.6
ZBED6	zinc finger BED-type containing 6	protein_coding	NP_001167579.1	2.6
COG5	component of oligomeric golgi complex 5	protein_coding	NP_001154992.2;NP_001366440.1;	2.6
TTLL3	tubulin tyrosine ligase like 3	protein_coding	NP_001021100.3;NP_001352980.1	2.6
PFKFB1	6-phosphofructo-2-kinase/fructose-2,6-biphosphat	protein_coding	NP_001258733.1;NP_001258734.1;	2.6
RAPGEF4	Rap guanine nucleotide exchange factor 4	protein_coding	NP_001093867.1;NP_001269828.1;	2.6
PATJ	PATJ crumbs cell polarity complex component	protein_coding	NP_001337074.2;NP_795352.3;XP_	2.6
ZNF197-AS1	ZNF197 antisense RNA 1	lncRNA	.	2.6
ZNF169	zinc finger protein 169	protein_coding	NP_001288204.1;NP_003439.2;NP_	2.6
MAP7	microtubule associated protein 7	protein_coding	NP_001185537.1;NP_001185538.1;	2.6
TMF1	TATA element modulatory factor 1	protein_coding	NP_001350808.1;NP_009045.2	2.6
SNORD44	small nucleolar RNA, C/D box 44	snoRNA	.	2.6
PTPN2	protein tyrosine phosphatase non-receptor type 2	protein_coding	NP_001193942.1;NP_001295216.1;	2.6
FLI1	Fli-1 proto-oncogene, ETS transcription factor	protein_coding	NP_001161153.1;NP_001257939.1;	2.6
SRGN	serglycin	protein_coding	NP_001307982.1;NP_001307983.1;	2.6
FAM151B	family with sequence similarity 151 member B	protein_coding	NP_991111.2;XP_006714627.2;XP_	2.6
LOC105372990	uncharacterized LOC105372990	lncRNA	.	2.6
LRRC37A17P	leucine rich repeat containing 37 member A17, ps	pseudogene	.	2.6
LINC02777	long intergenic non-protein coding RNA 2777, tra	lncRNA	.	2.6
TM4SF1	transmembrane 4 L six family member 1	protein_coding	NP_055035.1;XP_016861874.1	2.6
APLF	aprataxin and PNKP like factor	protein_coding	NP_775816.1	2.6
RPL7AP39	ribosomal protein L7a pseudogene 39	pseudogene	.	2.6
DOCK10	dedicator of cytokinesis 10	protein_coding	NP_001277192.1;NP_001350691.1;	2.6
MIR4517	microRNA 4517	miRNA	.	2.6
TPM3P6	tropomyosin 3 pseudogene 6	pseudogene	.	2.6
SHPRH	SNF2 histone linker PHD RING helicase	protein_coding	NP_001036148.2;NP_001357256.1;	2.6
RNF139-AS1	RNF139 antisense RNA 1 (head to head)	lncRNA	.	2.6
ANTXR2	ANTXR cell adhesion molecule 2	protein_coding	NP_001139266.1;NP_001273709.1;	2.6
LINC01537	long intergenic non-protein coding RNA 1537	lncRNA	.	2.6
RIMKLB	ribosomal modification protein rimK like family n	protein_coding	NP_001284705.1;NP_001339196.1;	2.6
FARS2	phenylalanyl-tRNA synthetase 2, mitochondrial	protein_coding	NP_001305801.1;NP_001361804.1;	2.6
LOC101929230	uncharacterized LOC101929230, transcript varian	lncRNA	.	2.6
LOC105372631	uncharacterized LOC105372631	lncRNA	.	2.6
LOC105371312	uncharacterized LOC105371312	lncRNA	.	2.6
HDX	highly divergent homeobox	protein_coding	NP_001170949.1;NP_001170950.1;	2.6
ZNF143	zinc finger protein 143	protein_coding	NP_001269585.1;NP_001269586.1;	2.6
LYST	lysosomal trafficking regulator	protein_coding	NP_000072.2;NP_001288294.1;XP_	2.6
MRPL45P2	mitochondrial ribosomal protein L45 pseudogene	ranscribed_pseudogen	.	2.6
ZNF83	zinc finger protein 83	protein_coding	NP_001099019.1;NP_001099020.1;	2.6
KCNMA1	potassium calcium-activated channel subfamily M	protein_coding	NP_001014797.1;NP_001154824.1;	2.6
SYNE1	spectrin repeat containing nuclear envelope protei	protein_coding	NP_001334630.1;NP_001334631.1;	2.6
CCDC81	coiled-coil domain containing 81	protein_coding	NP_001149946.1;NP_068599.3;XP_	2.6
ZCCHC18	zinc finger CCHC-type containing 18	protein_coding	NP_001137450.1;XP_011529314.1	2.6
NDUFAF2	NADH:ubiquinone oxidoreductase complex assen	protein_coding	NP_777549.1	2.6
GCC2	GRIP and coiled-coil domain containing 2	protein_coding	NP_852118.2;XP_006712933.1;XP_	2.6
PPIC-AS1	PPIC antisense RNA 1, transcript variant X1	lncRNA	.	2.6
AP3M2	adaptor related protein complex 3 subunit mu 2	protein_coding	NP_001127768.1;NP_006794.1;XP_	2.6
C1orf162	chromosome 1 open reading frame 162	protein_coding	NP_001287763.1;NP_001287764.1;	2.6
ARMC2	armadillo repeat containing 2	protein_coding	NP_001273538.1;NP_115507.4;XP_	2.6
ZNF460	zinc finger protein 460	protein_coding	NP_001317551.1;NP_006626.3	2.6
ZNF700	zinc finger protein 700	protein_coding	NP_001258777.1;NP_653167.1	2.6
DLG1	discs large MAGUK scaffold protein 1	protein_coding	NP_001091894.1;NP_001191315.1;	2.6
ANKRD10	ankyrin repeat domain 10	protein_coding	NP_001273650.1;NP_060134.2;XP_	2.6
LOC100289361	uncharacterized LOC100289361	lncRNA	.	2.6
DMXL2	Dmx like 2	protein_coding	NP_001167587.1;NP_001167588.1;	2.6
NGDN	neuroguidin	protein_coding	NP_001036100.1;NP_056329.1	2.6
USP32P1	ubiquitin specific peptidase 32 pseudogene 1	ranscribed_pseudogen	.	2.6
AGPAT4	1-acylglycerol-3-phosphate O-acyltransferase 4	protein_coding	NP_064518.1;XP_005267110.1;XP_	2.6
CAPRIN2	caprin family member 2	protein_coding	NP_001002259.1;NP_001193785.1;	2.6

COLQ	collagen like tail subunit of asymmetric acetylchol	protein_coding	NP_005668.2;NP_536799.1;NP_53(	2.6
LOC105379052	uncharacterized LOC105379052, transcript varian	lncRNA	.	2.6
ACAD11	acyl-CoA dehydrogenase family member 11	protein_coding	NP_115545.3	2.6
BIRC6	baculoviral IAP repeat containing 6	protein_coding	NP_001365054.1;NP_057336.3;XP_	2.6
LINC00618	long intergenic non-protein coding RNA 618	lncRNA	.	2.6
CCDC169-SOHLH2	CCDC169-SOHLH2 readthrough	protein_coding	NP_001185839.1	2.6
GORAB	golgin, RAB6 interacting	protein_coding	NP_001139511.2;NP_001307181.1;	2.6
SLC24A2	solute carrier family 24 member 2	protein_coding	NP_001180217.1;NP_001362779.1;	2.6
LOC100421409	glucosamine-phosphate N-acetyltransferase 1 pseu	pseudogene	.	2.6
CNTRL	centriolin	protein_coding	NP_001317691.1;NP_001356821.1;	2.6
GAPDHP62	glyceraldehyde 3 phosphate dehydrogenase pseudo	pseudogene	.	2.6
MYCBP2	MYC binding protein 2	protein_coding	NP_055872.4;XP_005266356.1;XP_	2.6
PMAIP1	phorbol-12-myristate-13-acetate-induced protein 1	protein_coding	NP_001369544.1;NP_001369545.1;	2.6
ND1	NADH dehydrogenase subunit 1	protein_coding	YP_003024026.1	2.6
IFT172	intraflagellar transport 172	protein_coding	NP_056477.1;XP_005264311.1;XP_	2.6
ZNF800	zinc finger protein 800	protein_coding	NP_789784.2;XP_005250238.1;XP_	2.6
LINC01239	long intergenic non-protein coding RNA 1239	lncRNA	.	2.6
MBTD1	mbt domain containing 1	protein_coding	NP_060113.2;XP_005257524.1;XP_	2.6
LOC105379326	uncharacterized LOC105379326, transcript varian	lncRNA	.	2.6
TDRD6	tudor domain containing 6	protein_coding	NP_001010870.1;NP_001161831.1	2.6
LOC105371087	uncharacterized LOC105371087	lncRNA	.	2.6
CRYM-AS1	CRYM antisense RNA 1	lncRNA	.	2.6
LOC101929057	uncharacterized LOC101929057	lncRNA	.	2.6
SCARNA7	small Cajal body-specific RNA 7	guide_RNA	.	2.6
EIF2S2P4	eukaryotic translation initiation factor 2 subunit 2	pseudogene	.	2.6
PRKCE	protein kinase C epsilon	protein_coding	NP_005391.1;XP_005264485.1;XP_	2.6
YES1P1	YES1 pseudogene 1	pseudogene	.	2.6
CCDC66	coiled-coil domain containing 66	protein_coding	NP_001012524.4;NP_001135419.1;	2.6
LOC105373264	uncharacterized LOC105373264	lncRNA	.	2.6
LOC107986855	uncharacterized LOC107986855, transcript varian	lncRNA	.	2.6
LOC112267858	uncharacterized LOC112267858	lncRNA	.	2.6
CBWD5	COBW domain containing 5	protein_coding	NP_001020087.2;NP_001273764.1;	2.6
KMT2C	lysine methyltransferase 2C	protein_coding	NP_733751.2;XP_005250082.1;XP	2.6
TCEAL4	transcription elongation factor A like 4	protein_coding	NP_001006936.1;NP_001006938.1;	2.6
LOC101928344	uncharacterized LOC101928344, transcript varian	lncRNA	.	2.6
LOC105379003	uncharacterized LOC105379003, transcript varian	lncRNA	.	2.6
MIR924HG	MIR924 host gene	lncRNA	.	2.6
LOC102723694	uncharacterized LOC102723694, transcript varian	lncRNA	.	2.5
KIAA0753	KIAA0753	protein_coding	NP_001338154.1;NP_055619.2;XP_	2.5
RAD18	RAD18 E3 ubiquitin protein ligase	protein_coding	NP_064550.3;XP_016862362.1	2.5
NPIPB3	nuclear pore complex interacting protein family m	protein_coding	NP_569731.2	2.5
LOC107986072	uncharacterized LOC107986072	lncRNA	.	2.5
LOC107985997	uncharacterized LOC107985997	lncRNA	.	2.5
DNMBP	dynamin binding protein	protein_coding	NP_001305255.1;NP_001305256.1;	2.5
ND2	NADH dehydrogenase subunit 2	protein_coding	YP_003024027.1	2.5
LOC105374122	uncharacterized LOC105374122, transcript varian	lncRNA	.	2.5
UBR5-AS1	UBR5 antisense RNA 1	lncRNA	.	2.5
PIP5K1P1-CATSPER	PPIP5K1P1-CATSPER2 readthrough	ranscribed_pseudogen	.	2.5
LOC100419307	ribosomal protein L5 pseudogene	pseudogene	.	2.5
NCAM2	neural cell adhesion molecule 2	protein_coding	NP_001339520.1;NP_001339521.1;	2.5
DNAJC2	DnaJ heat shock protein family (Hsp40) member C	protein_coding	NP_001123359.1;NP_001349596.1;	2.5
LGSN	lengsin, lens protein with glutamine synthetase do	protein_coding	NP_001137412.1;NP_057655.2;XP_	2.5
GUSBP2	GUSB pseudogene 2	ranscribed_pseudogen	.	2.5
SYTL4	synaptotagmin like 4	protein_coding	NP_001123368.1;NP_001167539.1;	2.5
NOTCH2NLR	notch 2 N-terminal like R (pseudogene)	ranscribed_pseudogen	.	2.5
ADAMTSL4-AS1	ADAMTSL4 antisense RNA 1	lncRNA	.	2.5
NPIPP1	nuclear pore complex interacting protein pseudoge	pseudogene	.	2.5
SARNP	SAP domain containing ribonucleoprotein	protein_coding	NP_149073.1	2.5
ATP2B1	ATPase plasma membrane Ca2+ transporting 1	protein_coding	NP_001001323.1;NP_001353449.1;	2.5
CHMP4A	charged multivesicular body protein 4A	protein_coding	NP_054888.3	2.5
SFI1	SFI1 centrin binding protein	protein_coding	NP_001007468.1;NP_001245254.1;	2.5
AKNAD1	AKNA domain containing 1	protein_coding	NP_689976.2	2.5
SLCO1B3	solute carrier organic anion transporter family mer	protein_coding	NP_001336849.1;NP_062818.1	2.5
ALS2	alsin Rho guanine nucleotide exchange factor ALS	protein_coding	NP_001129217.1;NP_065970.2;XP_	2.5
HEATR5B	HEAT repeat containing 5B	protein_coding	NP_061897.1;XP_006712097.1;XP_	2.5
NBPF1	NBPF member 1	protein_coding	NP_060410.3	2.5
SPATA9	spermatogenesis associated 9	protein_coding	NP_001336232.1;NP_114158.2;XP_	2.5
ZGRF1	zinc finger GRF-type containing 1	protein_coding	NP_001337326.1;NP_060862.3;XP_	2.5
HDAC8	histone deacetylase 8	protein_coding	NP_001159890.1;NP_001159891.1;	2.5
ERI3-IT1	ERI3 intronic transcript 1	lncRNA	.	2.5
FBXL4	F-box and leucine rich repeat protein 4	protein_coding	NP_001265645.1;NP_036292.2;XP_	2.5

LOC101930370	uncharacterized LOC101930370	lncRNA	.	2.5
SUCLG2-AS1	SUCLG2 antisense RNA 1 (head to head)	lncRNA	.	2.5
TBC1D3G	TBC1 domain family member 3G	protein_coding	NP_001278391.1;XP_005276971.1	2.5
LINC01515	long intergenic non-protein coding RNA 1515	lncRNA	.	2.5
LOC107984459	uncharacterized LOC107984459	lncRNA	.	2.5
FOXP1	forkhead box P1	protein_coding	NP_001012523.1;NP_001231737.1;	2.5
MDN1	midasin AAA ATPase 1	protein_coding	NP_055426.1;XP_005248757.1;XP_	2.5
LEO1	LEO1 homolog, Paf1/RNA polymerase II comple:	protein_coding	NP_001273359.1;NP_001310832.1;	2.5
SNORA1	small nucleolar RNA, H/ACA box 1	snoRNA	.	2.5
ENTPD7	ectonucleoside triphosphate diphosphohydrolase 7	protein_coding	NP_001336891.1;NP_001336892.1;	2.5
CCT6P1	chaperonin containing TCP1 subunit 6 pseudogen	ranscribed_pseudogen	.	2.5
OTUD4	OTU deubiquitinase 4	protein_coding	NP_001096123.1;NP_001352986.1;	2.5
ZNF117	zinc finger protein 117	protein_coding	NP_056936.2	2.5
SLC22A1	solute carrier family 22 member 1	protein_coding	NP_003048.1;NP_694857.1;XP_00	2.5
STAG3L4	stromal antigen 3-like 4 (pseudogene)	ranscribed_pseudogen	.	2.5
VPS8	VPS8 subunit of CORVET complex	protein_coding	NP_001009921.1;NP_001336221.1;	2.5
LOC101927888	uncharacterized LOC101927888, transcript varian	lncRNA	.	2.5
ANKRD26	ankyrin repeat domain 26	protein_coding	NP_001242982.1;NP_055730.2;XP_	2.5
KIAA1328	KIAA1328	protein_coding	NP_001309256.1;NP_001340847.1;	2.5
FBXO38	F-box protein 38	protein_coding	NP_001258652.1;NP_110420.3;NP_	2.5
EEF1A1P10	eukaryotic translation elongation factor 1 alpha 1 p	pseudogene	.	2.5
ITPR1	inositol 1,4,5-trisphosphate receptor type 1	protein_coding	NP_001093422.2;NP_001161744.1;	2.5
BEST3	bestrophin 3	protein_coding	NP_001269542.1;NP_001269543.1;	2.5
XKR6	XK related 6	protein_coding	NP_775954.2;XP_011542123.1;XP_	2.5
LOC107986361	uncharacterized LOC107986361	lncRNA	.	2.5
DPY19L2	dpy-19 like 2	protein_coding	NP_776173.3;XP_006719415.1;XP_	2.5
ZNF133	zinc finger protein 133	protein_coding	NP_001076799.2;NP_001269924.1;	2.5
RNF169	ring finger protein 169	protein_coding	NP_001092108.1;XP_011543191.1	2.5
AGAP7P	ArfGAP with GTPase domain, ankyrin repeat and	ranscribed_pseudogen	.	2.5
LOC105373715	uncharacterized LOC105373715	lncRNA	.	2.5
KHDC4	KH domain containing 4, pre-mRNA splicing fact	protein_coding	NP_055764.2	2.5
RBM5	RNA binding motif protein 5	protein_coding	NP_005769.1;XP_006712980.1;XP_	2.5
SNORD77	small nucleolar RNA, C/D box 77	snoRNA	.	2.5
POLA1	DNA polymerase alpha 1, catalytic subunit	protein_coding	NP_001317289.1;NP_001365232.1;	2.5
NIPSNAP3B	nipsnap homolog 3B	protein_coding	NP_060846.2;XP_011517141.1	2.5
PTBP2	polypyrimidine tract binding protein 2	protein_coding	NP_001287914.1;NP_001287915.1;	2.5
LRGUK	leucine rich repeats and guanylate kinase domain c	protein_coding	NP_001352629.1;NP_001352630.1;	2.5
TARS1	threonyl-tRNA synthetase 1	protein_coding	NP_001245366.1;NP_001245367.1;	2.5
HSP90B1	heat shock protein 90 beta family member 1	protein_coding	NP_003290.1	2.5
LOC105376037	uncharacterized LOC105376037	lncRNA	.	2.5
ZBTB38	zinc finger and BTB domain containing 38	protein_coding	NP_001073881.2;NP_001337028.1;	2.5
MOV10L1	Mov10 like RISC complex RNA helicase 1	protein_coding	NP_001157576.1;NP_001157577.1;	2.5
LOC105375523	uncharacterized LOC105375523, transcript varian	lncRNA	.	2.5
CCNL1	cyclin L1	protein_coding	NP_001295114.1;NP_064703.1;XP_	2.5
LINC01145	long intergenic non-protein coding RNA 1145	lncRNA	.	2.5
MAML2	mastermind like transcriptional coactivator 2	protein_coding	NP_115803.1;XP_011541325.1;XP_	2.5
ROS1	ROS proto-oncogene 1, receptor tyrosine kinase	protein_coding	NP_001365820.1;NP_001365831.1;	2.5
MGA	MAX dimerization protein MGA	protein_coding	NP_001074010.2;NP_001157745.1;	2.5
RPS7P3	ribosomal protein S7 pseudogene 3	pseudogene	.	2.5
VTI1A	vesicle transport through interaction with t-SNAR	protein_coding	NP_001305132.1;NP_001305134.1;	2.5
LTF	lactotransferrin	protein_coding	NP_001186078.1;NP_001308050.1;	2.5
LOC105369225	uncharacterized LOC105369225, transcript varian	lncRNA	.	2.5
BRCA1	BRCA1 DNA repair associated	protein_coding	NP_009225.1;NP_009228.2;NP_00	2.5
IKBKB	inhibitor of nuclear factor kappa B kinase subunit	protein_coding	NP_001177649.2;NP_001229707.1;	2.5
UBAP1L	ubiquitin associated protein 1 like	protein_coding	NP_001157164.1;XP_011519849.1;	2.5
WARS1	tryptophanyl-tRNA synthetase 1	protein_coding	NP_004175.2;NP_776049.1;NP_99	2.5
IARS1	isoleucyl-tRNA synthetase 1	protein_coding	NP_001361228.1;NP_001361229.1;	2.5
DUXAP9	double homeobox A pseudogene 9	ranscribed_pseudogen	.	2.5
EMC3-AS1	EMC3 antisense RNA 1	lncRNA	.	2.5
LOC102724093	golgin subfamily A member 6-like protein 4	ranscribed_pseudogen	.	2.5
LACC1	laccase domain containing 1	protein_coding	NP_001121775.1;NP_001337567.1;	2.5
ANKRD12	ankyrin repeat domain 12	protein_coding	NP_001077094.1;NP_001190985.1;	2.5
SETD4	SET domain containing 4	protein_coding	NP_001007260.1;NP_001007262.1;	2.5
ANAPC1P1	anaphase promoting complex subunit 1 pseudogen	ranscribed_pseudogen	.	2.5
CCNT2	cyclin T2	protein_coding	NP_001232.1;NP_001307677.1;NP_	2.5
TDP1	tyrosyl-DNA phosphodiesterase 1	protein_coding	NP_001008744.1;NP_001317134.1;	2.5
SPRY3	sprouty RTK signaling antagonist 3	protein_coding	NP_001291919.1;NP_005831.1	2.4
RPL11	ribosomal protein L11	protein_coding	NP_000966.2;NP_001186731.1	2.4
KANTR	KDM5C adjacent transcript	lncRNA	.	2.4
UGGT2	UDP-glucose glycoprotein glucosyltransferase 2	protein_coding	NP_064506.3;XP_011519396.1;XP_	2.4
LOC107984011	uncharacterized LOC107984011	lncRNA	.	2.4

PARP12	poly(ADP-ribose) polymerase family member 12	protein_coding	NP_073587.1;XP_005250095.1;XP_	2.4
AKAP12	A-kinase anchoring protein 12	protein_coding	NP_001357275.1;NP_005091.2;NP_	2.4
PDZK1P1	PDZ domain containing 1 pseudogene 1	ranscribed_pseudogen	.	2.4
DDX31	DEAD-box helicase 31	protein_coding	NP_001309269.1;NP_001309270.1;	2.4
CDH13	cadherin 13	protein_coding	NP_001207417.1;NP_001207418.1;	2.4
RN7SL481P	RNA, 7SL, cytoplasmic 481, pseudogene	pseudogene	.	2.4
PPP1R21	protein phosphatase 1 regulatory subunit 21	protein_coding	NP_001129101.1;NP_001180404.1;	2.4
INVS	inversin	protein_coding	NP_001305310.1;NP_001305311.1;	2.4
TMEM161B	transmembrane protein 161B	protein_coding	NP_001275936.1;NP_001275937.1;	2.4
ERO1B	endoplasmic reticulum oxidoreductase 1 beta	protein_coding	NP_063944.3;XP_016857323.1;XP_	2.4
DARS-AS1	DARS antisense RNA 1	lncRNA	.	2.4
FUT8	fucosyltransferase 8	protein_coding	NP_001358462.1;NP_001358463.1;	2.4
NFAT5	nuclear factor of activated T cells 5	protein_coding	NP_001106649.1;NP_001354638.1;	2.4
SEPTIN14P14	septin 14 pseudogene 14	pseudogene	.	2.4
TTC21A	tetratricopeptide repeat domain 21A	protein_coding	NP_001098983.2;NP_001353828.1;	2.4
KCNQ5	potassium voltage-gated channel subfamily Q mer	protein_coding	NP_001153602.1;NP_001153604.1;	2.4
ZNF343	zinc finger protein 343	protein_coding	NP_001269424.1;NP_001269425.1;	2.4
STX16-NPEPL1	STX16-NPEPL1 readthrough (NMD candidate)	lncRNA	.	2.4
PLAU	plasminogen activator, urokinase	protein_coding	NP_001138503.2;NP_001306120.2;	2.4
ZNF354B	zinc finger protein 354B	protein_coding	NP_478137.1	2.4
NDUFAF7	NADH:ubiquinone oxidoreductase complex assem	protein_coding	NP_001077415.1;NP_001336953.1;	2.4
PRDX2P3	peroxiredoxin 2 pseudogene 3	pseudogene	.	2.4
SNORD19B	small nucleolar RNA, C/D box 19B	snoRNA	.	2.4
NAALADL2	N-acetylated alpha-linked acidic dipeptidase like 2	protein_coding	NP_996898.2;XP_006713623.1;XP_	2.4
ZNF701	zinc finger protein 701	protein_coding	NP_001166126.1;NP_060730.2;XP_	2.4
LOC105378077	uncharacterized LOC105378077	lncRNA	.	2.4
TAF1A	TATA-box binding protein associated factor, RNA	protein_coding	NP_001188465.1;NP_005672.1;NP_	2.4
MORF4L2-AS1	MORF4L2 antisense RNA 1	lncRNA	.	2.4
IFRD1	interferon related developmental regulator 1	protein_coding	NP_001007246.1;NP_001184008.1;	2.4
MTHFD2	methylenetetrahydrofolate dehydrogenase (NADP	protein_coding	NP_006627.2;XP_006711987.1	2.4
LOC220729	succinate dehydrogenase complex flavoprotein sul	ranscribed_pseudogen	.	2.4
ZNF875	zinc finger protein 875	protein_coding	NP_001316690.1;NP_001316691.1;	2.4
LOC100130283	uncharacterized LOC100130283	lncRNA	.	2.4
ICE1	interactor of little elongation complex ELL subuni	protein_coding	NP_056140.1;XP_011512301.1;XP_	2.4
LOC102724117	golgin subfamily A member 6-like protein 4	ranscribed_pseudogen	.	2.4
ZNF564	zinc finger protein 564	protein_coding	NP_659413.1	2.4
LOC107984431	uncharacterized LOC107984431	lncRNA	.	2.4
ENTPD5	ectonucleoside triphosphate diphosphohydrolase 5	protein_coding	NP_001240.1;NP_001308913.1;NP_	2.4
SDHCP3	succinate dehydrogenase complex subunit C pseuc	pseudogene	.	2.4
LAP3	leucine aminopeptidase 3	protein_coding	NP_056991.2	2.4
TTC13	tetratricopeptide repeat domain 13	protein_coding	NP_001116307.2;NP_001363436.1;	2.4
FAM157A	family with sequence similarity 157 member A	lncRNA	.	2.4
TENT2	terminal nucleotidyltransferase 2	protein_coding	NP_001107865.1;NP_001107866.1;	2.4
COG3	component of oligomeric golgi complex 3	protein_coding	NP_113619.3;XP_011533568.1;XP_	2.4
NFKB1	nuclear factor kappa B subunit 1	protein_coding	NP_001158884.1;NP_001306155.1;	2.4
LAMA1	laminin subunit alpha 1	protein_coding	NP_005550.2	2.4
DUSP5	dual specificity phosphatase 5	protein_coding	NP_004410.3	2.4
RPS24	ribosomal protein S24	protein_coding	NP_001017.1;NP_001135754.1;NP_	2.4
KDM3A	lysine demethylase 3A	protein_coding	NP_001140160.1;NP_060903.2;XP_	2.4
VEGFA	vascular endothelial growth factor A	protein_coding	NP_001020537.2;NP_001020538.2;	2.4
DDX12P	DEAD/H-box helicase 12, pseudogene	ranscribed_pseudogen	.	2.4
LOC107986622	uncharacterized LOC107986622	lncRNA	.	2.4
MTIF3	mitochondrial translational initiation factor 3	protein_coding	NP_001159733.1;NP_001159734.1;	2.4
ZC3H15	zinc finger CCCH-type containing 15	protein_coding	NP_060941.2;XP_016859991.1	2.4
SNAI2	snail family transcriptional repressor 2	protein_coding	NP_003059.1	2.4
SNORA8	small nucleolar RNA, H/ACA box 8	snoRNA	.	2.4
CD83	CD83 molecule	protein_coding	NP_001035370.1;NP_001238830.1;	2.4
UBN2	ubinuclein 2	protein_coding	NP_775840.3;XP_005250306.2;XP_	2.4
RPL23AP32	ribosomal protein L23a pseudogene 32	ranscribed_pseudogen	.	2.4
RPL26	ribosomal protein L26	protein_coding	NP_000978.1;NP_001302459.1;NP_	2.4
HMGB1P5	high mobility group box 1 pseudogene 5	pseudogene	.	2.4
LCP1	lymphocyte cytosolic protein 1	protein_coding	NP_002289.2;XP_005266431.1	2.4
PRPF4B	pre-mRNA processing factor 4B	protein_coding	NP_003904.3;XP_011513272.1;XP_	2.4
ZNF267	zinc finger protein 267	protein_coding	NP_001252517.2;NP_003405.4	2.4
TBRG1	transforming growth factor beta regulator 1	protein_coding	NP_116200.2;XP_005271766.1;XP_	2.4
YPEL2	yippee like 2	protein_coding	NP_001005404.1;XP_016880110.1	2.4
BDP1	B double prime 1, subunit of RNA polymerase III	protein_coding	NP_060899.2;XP_011541813.1;XP_	2.4
ADPRM	ADP-ribose/CDP-alcohol diphosphatase, mangane	protein_coding	NP_064618.3	2.4
LOC105374795	uncharacterized LOC105374795	lncRNA	.	2.4
FAM227A	family with sequence similarity 227 member A	protein_coding	NP_001013669.1;NP_001277959.1;	2.4
TLK2P1	tousled like kinase 2 pseudogene 1	pseudogene	.	2.4

NUCB2	nucleobindin 2	protein_coding	NP_001317156.1;NP_001339590.1;	2.4
LINC02535	long intergenic non-protein coding RNA 2535	lncRNA	.	2.4
SPIDR	scaffold protein involved in DNA repair	protein_coding	NP_001073863.1;NP_001269845.1;	2.4
MTHFS	methenyltetrahydrofolate synthetase	protein_coding	NP_001186687.1;NP_006432.1	2.4
ENOSF1	enolase superfamily member 1	protein_coding	NP_001305689.1;NP_001340994.1;	2.4
LNP1	leukemia NUP98 fusion partner 1	protein_coding	NP_001078920.1	2.4
TRIP11	thyroid hormone receptor interactor 11	protein_coding	NP_001308780.1;NP_004230.2;XP_	2.4
ZSCAN30	zinc finger and SCAN domain containing 30	protein_coding	NP_001106205.1;NP_001159484.1;	2.4
THOC2	THO complex 2	protein_coding	NP_001075019.1;XP_016885151.1;	2.4
SERPINB7	serpin family B member 7	protein_coding	NP_001035237.1;NP_001248759.1;	2.4
MTAP	methylthioadenosine phosphorylase	protein_coding	NP_002442.2	2.4
LINC01410	long intergenic non-protein coding RNA 1410	lncRNA	.	2.4
LOC107984567	uncharacterized LOC107984567	lncRNA	.	2.4
RPAP2	RNA polymerase II associated protein 2	protein_coding	NP_079089.2;XP_005271280.1;XP_	2.4
POLK	DNA polymerase kappa	protein_coding	NP_001332850.1;NP_001332851.1;	2.4
B2M	beta-2-microglobulin	protein_coding	NP_004039.1;XP_005254606.1	2.4
TBC1D15	TBC1 domain family member 15	protein_coding	NP_001139685.2;NP_001139686.1;	2.4
LOC107985201	uncharacterized LOC107985201	lncRNA	.	2.4
RASA2	RAS p21 protein activator 2	protein_coding	NP_001290174.1;NP_001290175.1;	2.4
VPS13D	vacuolar protein sorting 13 homolog D	protein_coding	NP_056193.2;NP_060626.2	2.4
ZFP37	ZFP37 zinc finger protein	protein_coding	NP_001269444.1;NP_001269447.1;	2.4
KDM5D	lysine demethylase 5D	protein_coding	NP_001140177.1;NP_001140178.1;	2.4
PPFIBP1	PPFIA binding protein 1	protein_coding	NP_001185844.1;NP_001185845.2;	2.4
DIAPH2	diaphanous related formin 2	protein_coding	NP_006720.1;NP_009293.1	2.4
KCNJ15	potassium inwardly rectifying channel subfamily J	protein_coding	NP_001263364.1;NP_001263365.1;	2.4
CCNH	cyclin H	protein_coding	NP_001186118.1;NP_001230.1;NP_	2.4
DPY19L1P1	DPY19L1 pseudogene 1	ranscribed_pseudogen	.	2.4
PNN	pinin, desmosome associated protein	protein_coding	NP_002678.3	2.4
LOC153684	uncharacterized LOC153684	lncRNA	.	2.4
RAD50	RAD50 double strand break repair protein	protein_coding	NP_005723.2	2.4
LNPEP	leucyl and cystinyl aminopeptidase	protein_coding	NP_005566.2;NP_787116.2;XP_02	2.4
LOC107985188	uncharacterized LOC107985188	lncRNA	.	2.4
RBM39	RNA binding motif protein 39	protein_coding	NP_001229528.1;NP_001229529.1;	2.4
OR10V2P	olfactory receptor family 10 subfamily V member	ranscribed_pseudogen	.	2.4
TTC41P	tetratricopeptide repeat domain 41, pseudogene	ranscribed_pseudogen	.	2.4
GLRX	glutaredoxin	protein_coding	NP_001112362.1;NP_001230587.1;	2.4
RANBP17	RAN binding protein 17	protein_coding	NP_075048.1;XP_011532929.1;XP_	2.4
UFL1-AS1	UFL1 antisense RNA 1, transcript variant X4	lncRNA	.	2.4
CEBPG	CCAAT enhancer binding protein gamma	protein_coding	NP_001239225.1;NP_001797.1;XP_	2.4
CNTLN	centlein	protein_coding	NP_001107867.1;NP_001273913.1;	2.4
LOC112267915	uncharacterized LOC112267915	lncRNA	.	2.4
VPS13C	vacuolar protein sorting 13 homolog C	protein_coding	NP_001018098.1;NP_060154.3;NP_	2.4
ZNF41	zinc finger protein 41	protein_coding	NP_001311068.1;NP_001311069.1;	2.4
ZNF37A	zinc finger protein 37A	protein_coding	NP_001007095.1;NP_001171572.1;	2.4
LOC100505642	chromosome 9 open reading frame 85 pseudogene	pseudogene	.	2.4
NPIPA7	nuclear pore complex interacting protein family m	protein_coding	NP_001269436.1;XP_005255792.1;	2.4
PLCXD2	phosphatidylinositol specific phospholipase C X d	protein_coding	NP_001172035.1;NP_695000.1	2.4
LIPG	lipase G, endothelial type	protein_coding	NP_001294935.1;NP_006024.1;XP_	2.4
ADGRB3	adhesion G protein-coupled receptor B3	protein_coding	NP_001695.2	2.4
ENTPD1-AS1	ENTPD1 antisense RNA 1	lncRNA	.	2.4
WARS2-AS1	WARS2 antisense RNA 1	lncRNA	.	2.4
SNORD74	small nucleolar RNA, C/D box 74	snoRNA	.	2.4
POLI	DNA polymerase iota	protein_coding	NP_001338539.1;NP_001338540.1;	2.4
SEPTIN14P2	septin 14 pseudogene 2	pseudogene	.	2.3
ZNF331	zinc finger protein 331	protein_coding	NP_001073375.1;NP_001073376.1;	2.3
SLFN13	schlafen family member 13	protein_coding	NP_653283.3;XP_005257979.1;XP_	2.3
ETS1	ETS proto-oncogene 1, transcription factor	protein_coding	NP_001137292.1;NP_001155894.1;	2.3
PIKFYVE	phosphoinositide kinase, FYVE-type zinc finger c	protein_coding	NP_001171471.1;NP_055855.2;NP_	2.3
RIC8B	RIC8 guanine nucleotide exchange factor B	protein_coding	NP_001317074.1;NP_001317075.1;	2.3
LINC01358	long intergenic non-protein coding RNA 1358	lncRNA	.	2.3
TRNR	tRNA-Arg	tRNA	.	2.3
ZNF507	zinc finger protein 507	protein_coding	NP_001129628.1;NP_055725.2	2.3
CRYBG3	crystallin beta-gamma domain containing 3	protein_coding	NP_705833.3;XP_005247174.1	2.3
BCKDHB	branched chain keto acid dehydrogenase E1 subun	protein_coding	NP_000047.1;NP_001305904.1;NP_	2.3
EFNA5	ephrin A5	protein_coding	NP_001953.1;XP_006714628.1;XP_	2.3
TRPV1	transient receptor potential cation channel subfami	protein_coding	NP_061197.4;NP_542435.2;NP_54	2.3
PPIAP2	peptidylprolyl isomerase A pseudogene 2	pseudogene	.	2.3
LOC642846	DEAD/H (Asp-Glu-Ala-Asp/His) box polypeptide	lncRNA	.	2.3
TTC21B	tetratricopeptide repeat domain 21B	protein_coding	NP_079029.3;XP_006712824.1;XP_	2.3
KTN1	kinectin 1	protein_coding	NP_001072989.1;NP_001072990.1;	2.3
UST-AS2	UST antisense RNA 2	lncRNA	.	2.3

NEDD4	NEDD4 E3 ubiquitin protein ligase	protein_coding	NP_001271267.1;NP_001271268.1;	2.3
ZNF519	zinc finger protein 519	protein_coding	NP_660330.2	2.3
CFAP53	cilia and flagella associated protein 53	protein_coding	NP_659457.2;XP_024306868.1	2.3
EYS	eyes shut homolog	protein_coding	NP_001136272.1;NP_001136273.1;	2.3
MET	MET proto-oncogene, receptor tyrosine kinase	protein_coding	NP_000236.2;NP_001120972.1;NP_	2.3
KCNAB3	potassium voltage-gated channel subfamily A regu	protein_coding	NP_004723.2;XP_011522370.1;XP_	2.3
MIR5047	microRNA 5047	miRNA	.	2.3
ATP5F1E	ATP synthase F1 subunit epsilon	protein_coding	NP_008817.1	2.3
ESF1	ESF1 nucleolar pre-rRNA processing protein hom	protein_coding	NP_001263309.1;NP_057733.2;XP_	2.3
BAZ1A	bromodomain adjacent to zinc finger domain 1A	protein_coding	NP_038476.2;NP_872589.1;XP_01	2.3
MEF2C	myocyte enhancer factor 2C	protein_coding	NP_001124477.1;NP_001180276.1;	2.3
LOC107984814	uncharacterized LOC107984814, transcript varian	protein_coding	XP_016879409.1	2.3
RPGRIP1L	RPGRIP1 like	protein_coding	NP_001121369.1;NP_001295263.1;	2.3
FIG4	FIG4 phosphoinositide 5-phosphatase	protein_coding	NP_055660.1;XP_011534583.1;XP_	2.3
MSH5	mutS homolog 5	protein_coding	NP_002432.1;NP_079535.4;NP_75	2.3
ZNF670	zinc finger protein 670	protein_coding	NP_001191149.1;NP_149990.1	2.3
GALNT7	polypeptide N-acetylgalactosaminyltransferase 7	protein_coding	NP_001362528.1;NP_001362529.1;	2.3
SUCLG2	succinate-CoA ligase GDP-forming subunit beta	protein_coding	NP_001171070.1;NP_003839.2;XP_	2.3
RNF6	ring finger protein 6	protein_coding	NP_005968.1;NP_898864.1;NP_89	2.3
GABBR1	gamma-aminobutyric acid type B receptor subunit	protein_coding	NP_001305982.1;NP_001461.1;NP_	2.3
SNORA62	small nucleolar RNA, H/ACA box 62	snoRNA	.	2.3
MIA3	MIA SH3 domain ER export factor 3	protein_coding	NP_001287796.1;NP_001310991.1;	2.3
FBXO30-DT	FBXO30 divergent transcript	lncRNA	.	2.3
KBTBD2	kelch repeat and BTB domain containing 2	protein_coding	NP_056298.2;XP_005249753.1;XP_	2.3
SCN8A	sodium voltage-gated channel alpha subunit 8	protein_coding	NP_001171455.1;NP_001317189.1;	2.3
PTPDC1	protein tyrosine phosphatase domain containing 1	protein_coding	NP_001240758.1;NP_001240759.1;	2.3
ZC3HAV1	zinc finger CCCH-type containing, antiviral 1	protein_coding	NP_001350420.1;NP_064504.2;NP_	2.3
WDR43	WD repeat domain 43	protein_coding	NP_055946.1	2.3
SLC27A3	solute carrier family 27 member 3	protein_coding	NP_001304858.3;NP_077306.3	2.3
AVL9	AVL9 cell migration associated	protein_coding	NP_055875.1;XP_005249725.1;XP_	2.3
GLYATL2	glycine-N-acyltransferase like 2	protein_coding	NP_659453.3;XP_011543111.1;XP_	2.3
SCYL3	SCY1 like pseudokinase 3	protein_coding	NP_065156.5;NP_851607.2;XP_00	2.3
LOC107986649	uncharacterized LOC107986649, transcript varian	lncRNA	.	2.3
LOC100507336	uncharacterized LOC100507336, transcript varian	lncRNA	.	2.3
MON2	MON2 homolog, regulator of endosome-to-Golgi	protein_coding	NP_001265398.1;NP_001265399.1;	2.3
L3MBTL3	L3MBTL histone methyl-lysine binding protein 3	protein_coding	NP_001007103.1;NP_001333479.1;	2.3
SLC25A26	solute carrier family 25 member 26	protein_coding	NP_001158268.1;NP_001337920.1;	2.3
ZNF814	zinc finger protein 814	protein_coding	NP_001138461.1;XP_016882686.1	2.3
TPK1	thiamin pyrophosphokinase 1	protein_coding	NP_001035947.1;NP_001337808.1;	2.3
ATXN7L1	ataxin 7 like 1	protein_coding	NP_001305158.1;NP_065776.1;NP	2.3
TGDS	TDP-glucose 4,6-dehydratase	protein_coding	NP_001291359.1;NP_055120.1;XP_	2.3
BTN3A2	butyrophilin subfamily 3 member A2	protein_coding	NP_001184175.1;NP_001184176.1;	2.3
ME3-DT	ME3 divergent transcript, transcript variant X2	lncRNA	.	2.3
ATP10D	ATPase phospholipid transporting 10D (putative)	protein_coding	NP_065186.3;XP_005248176.1;XP_	2.3
ZC3H6	zinc finger CCCH-type containing 6	protein_coding	NP_940983.2;XP_006712582.1	2.3
RNMT	RNA guanine-7 methyltransferase	protein_coding	NP_001295192.1;NP_001365061.1;	2.3
AKAP3	A-kinase anchoring protein 3	protein_coding	NP_001265238.2;NP_006413.3;XP_	2.3
ZNF721	zinc finger protein 721	protein_coding	NP_597731.2	2.3
HMGCR	3-hydroxy-3-methylglutaryl-CoA reductase	protein_coding	NP_000850.1;NP_001124468.1;NP_	2.3
PRDM8	PR/SET domain 8	protein_coding	NP_001092873.1;NP_064611.3;XP_	2.3
TRIT1	tRNA isopentenyltransferase 1	protein_coding	NP_001299620.1;NP_001299621.1;	2.3
CHIC2	cysteine rich hydrophobic domain 2	protein_coding	NP_036242.1;XP_006714100.1;XP_	2.3
LRCH1	leucine rich repeats and calponin homology domai	protein_coding	NP_001157683.2;NP_001157685.2;	2.3
LOC101927439	uncharacterized LOC101927439, transcript varian	lncRNA	.	2.3
TRMO	tRNA methyltransferase O	protein_coding	NP_001317654.1;NP_001358586.1;	2.3
NLRP3	NLR family pyrin domain containing 3	protein_coding	NP_001073289.2;NP_001120933.2;	2.3
CUBN	cubilin	protein_coding	NP_001072.2;XP_011518010.1;XP_	2.3
PTPRM	protein tyrosine phosphatase receptor type M	protein_coding	NP_001098714.1;NP_001365071.1;	2.3
FNIP1	folliculin interacting protein 1	protein_coding	NP_001008738.3;NP_001333042.2;	2.3
SPATA5	spermatogenesis associated 5	protein_coding	NP_001304728.1;NP_001332785.1;	2.3
GUSBP1	GUSB pseudogene 1	ranscribed_pseudogen	.	2.3
C1S	complement C1s	protein_coding	NP_001333779.1;NP_001725.1;NP_	2.3
GBP1	guanylate binding protein 1	protein_coding	NP_002044.2;XP_024302119.1	2.3
JPX	JPX transcript, XIST activator	lncRNA	.	2.3
LOC107985678	uncharacterized LOC107985678	protein_coding	XP_016885496.1	2.3
ELF2	E74 like ETS transcription factor 2	protein_coding	NP_001263386.1;NP_001263387.1;	2.3
HCG17	HLA complex group 17	lncRNA	.	2.3
MIR17HG	miR-17-92a-1 cluster host gene	lncRNA	.	2.3
CENPP	centromere protein P	protein_coding	NP_001012267.1;NP_001273898.1;	2.3
LOC107986798	uncharacterized LOC107986798	lncRNA	.	2.3
ARGLU1	arginine and glutamate rich 1	protein_coding	NP_060481.3	2.3

ANKRD50	ankyrin repeat domain 50	protein_coding	NP_001161354.1;NP_065070.1;XP_	2.3
NT5E	5'-nucleotidase ecto	protein_coding	NP_001191742.1;NP_002517.1	2.3
LOC101929506	uncharacterized LOC101929506	lncRNA	.	2.3
CDK5RAP2	CDK5 regulatory subunit associated protein 2	protein_coding	NP_001011649.1;NP_001258968.1;	2.3
PIK3C3	phosphatidylinositol 3-kinase catalytic subunit typ	protein_coding	NP_001294949.1;NP_002638.2;XP_	2.3
SEMA3C	semaphorin 3C	protein_coding	NP_001337049.1;NP_001337050.1;	2.3
PSPC1	paraspeckle component 1	protein_coding	NP_001035879.1;NP_001341837.1;	2.3
SBF2	SET binding factor 2	protein_coding	NP_112224.1;XP_005253211.1;XP_	2.3
GRIK2	glutamate ionotropic receptor kainate type subunit	protein_coding	NP_001159719.1;NP_068775.1;NP_	2.3
PDP1	pyruvate dehyrogenase phosphatase catalytic subu	protein_coding	NP_001155251.1;NP_001155252.1;	2.3
TDRD3	tudor domain containing 3	protein_coding	NP_001139542.1;NP_001139543.1;	2.3
DCUN1D2	defective in cullin neddylation 1 domain containin	protein_coding	NP_001014305.1;XP_016876132.1;	2.3
SEC22B3P	SEC22 homolog B3, pseudogene	ranscribed_pseudogen	.	2.3
CPT1B	carnitine palmitoyltransferase 1B	protein_coding	NP_001138606.1;NP_001138607.1;	2.3
LSG1	large 60S subunit nuclear export GTPase 1	protein_coding	NP_060855.2;XP_016862285.1	2.3
DHX35	DEAH-box helicase 35	protein_coding	NP_001177738.1;NP_068750.2;XP_	2.3
PPP1R1C	protein phosphatase 1 regulatory inhibitor subunit	protein_coding	NP_001074014.1;NP_001248353.1;	2.3
RSRC2	arginine and serine rich coiled-coil 2	protein_coding	NP_075388.2;XP_005253658.1;XP_	2.3
SNORD2	small nucleolar RNA, C/D box 2	snoRNA	.	2.3
ZNF330	zinc finger protein 330	protein_coding	NP_001278931.1;NP_055302.1;XP_	2.3
LOC102724805	uncharacterized LOC102724805, transcript varian	lncRNA	.	2.3
ASH1L	ASH1 like histone lysine methyltransferase	protein_coding	NP_001353106.1;NP_060959.2;XP_	2.3
LRRC28	leucine rich repeat containing 28	protein_coding	NP_001271329.1;NP_001308604.1;	2.3
CASP8AP2	caspase 8 associated protein 2	protein_coding	NP_001131139.1;NP_001131140.1;	2.3
WDR7-OT1	WDR7 overlapping transcript 1	lncRNA	.	2.3
CHD9	chromodomain helicase DNA binding protein 9	protein_coding	NP_001295248.1;NP_001339056.1;	2.3
WIPI1	WD repeat domain, phosphoinositide interacting 1	protein_coding	NP_001307701.1;NP_060453.3;XP_	2.3
DDX10	DEAD-box helicase 10	protein_coding	NP_004389.2	2.3
RPS6KC1	ribosomal protein S6 kinase C1	protein_coding	NP_001129610.1;NP_001274147.1;	2.3
EML5	EMAP like 5	protein_coding	NP_899243.1;XP_006720133.1;XP_	2.3
MTRF1	mitochondrial translation release factor 1	protein_coding	NP_001341002.1;NP_001341003.1;	2.3
PARP6	poly(ADP-ribose) polymerase family member 6	protein_coding	NP_001310444.1;NP_001310445.1;	2.3
BPTF	bromodomain PHD finger transcription factor	protein_coding	NP_004450.3;NP_872579.2;XP_00	2.3
TTF2	transcription termination factor 2	protein_coding	NP_003585.3;XP_005271334.1;XP_	2.3
LOC112268167	.	pseudogene	.	2.3
LGR5	leucine rich repeat containing G protein-coupled r	protein_coding	NP_001264155.1;NP_001264156.1;	2.3
SMC6	structural maintenance of chromosomes 6	protein_coding	NP_001135758.1;NP_078900.1;XP_	2.3
POLD3	DNA polymerase delta 3, accessory subunit	protein_coding	NP_001350526.1;NP_006582.1;XP	2.3
TBC1D3E	TBC1 domain family member 3E	protein_coding	NP_001278395.1;XP_006722317.1	2.3
TFPI	tissue factor pathway inhibitor	protein_coding	NP_001027452.1;NP_001305870.1;	2.3
LINC01909	long intergenic non-protein coding RNA 1909	lncRNA	.	2.3
IL12A	interleukin 12A	protein_coding	NP_000873.2;NP_001341511.1;NP_	2.3
DPH6-DT	DPH6 divergent transcript	lncRNA	.	2.3
TFAP2E	transcription factor AP-2 epsilon	protein_coding	NP_848643.2;XP_016856628.1	2.3
RPL23AP4	ribosomal protein L23a pseudogene 4	pseudogene	.	2.3
LOC101929823	uncharacterized LOC101929823, transcript varian	lncRNA	.	2.3
GALC	galactosylceramidase	protein_coding	NP_000144.2;NP_001188330.1;NP_	2.3
IL6ST	interleukin 6 signal transducer	protein_coding	NP_001177910.1;NP_001351204.1;	2.3
USP32	ubiquitin specific peptidase 32	protein_coding	NP_115971.2;XP_011523673.1;XP_	2.3
PSG5	pregnancy specific beta-1-glycoprotein 5	protein_coding	NP_001123486.1;NP_002772.3;XP	2.3
CDK17	cyclin dependent kinase 17	protein_coding	NP_001163935.1;NP_002586.2;XP	2.3
GTF2IRD2	GTF2I repeat domain containing 2	protein_coding	NP_001268376.1;NP_001355229.1;	2.3
CASC9	cancer susceptibility 9	lncRNA	.	2.3
ZNF780B	zinc finger protein 780B	protein_coding	NP_001005851.1;XP_005258647.1;	2.3
KLF7	Kruppel like factor 7	protein_coding	NP_001257871.1;NP_001257872.1;	2.3
MYO5A	myosin VA	protein_coding	NP_000250.3;NP_001135967.2;NP_	2.3
MTERF4	mitochondrial transcription termination factor 4	protein_coding	NP_001317108.1;NP_001317109.1;	2.3
EPC1	enhancer of polycomb homolog 1	protein_coding	NP_001258933.1;NP_001258948.1;	2.2
SLC4A5	solute carrier family 4 member 5	protein_coding	NP_067019.3;NP_597812.1	2.2
SNORD27	small nucleolar RNA, C/D box 27	snoRNA	.	2.2
LOC105372675	uncharacterized LOC105372675, transcript varian	lncRNA	.	2.2
ATG7	autophagy related 7	protein_coding	NP_001129503.2;NP_001138384.1;	2.2
FRMD4A	FERM domain containing 4A	protein_coding	NP_001305265.1;NP_001305266.1;	2.2
THG1L	tRNA-histidine guanylyltransferase 1 like	protein_coding	NP_001304753.1;NP_001304754.1;	2.2
IVNS1ABP	influenza virus NS1A binding protein	protein_coding	NP_006460.2;XP_005244900.1;XP_	2.2
SERGEF	secretion regulating guanine nucleotide exchange	protein_coding	NP_036271.1	2.2
FBXW7	F-box and WD repeat domain containing 7	protein_coding	NP_001013433.1;NP_001243998.1;	2.2
PHF3	PHD finger protein 3	protein_coding	NP_001277188.1;NP_001277189.1;	2.2
LOC107985026	.	pseudogene	.	2.2
ZNF44	zinc finger protein 44	protein_coding	NP_001157748.1;NP_001340478.1;	2.2
TYRO3P	TYRO3P protein tyrosine kinase pseudogene	ranscribed_pseudogen	.	2.2

MILR1	mast cell immunoglobulin like receptor 1	protein_coding	NP_001078892.1;NP_001278245.1;	2.2
NEK11	NIMA related kinase 11	protein_coding	NP_001139475.1;NP_001308149.1;	2.2
NHLRC3	NHL repeat containing 3	protein_coding	NP_001012772.1;NP_001017370.1	2.2
ZBTB21	zinc finger and BTB domain containing 21	protein_coding	NP_001091872.1;NP_001091873.1;	2.2
ZNF236	zinc finger protein 236	protein_coding	NP_001293018.1;NP_031371.3;XP_	2.2
ATP8	ATP synthase F0 subunit 8	protein_coding	YP_003024030.1	2.2
LINC00893	long intergenic non-protein coding RNA 893	lncRNA	.	2.2
SETD6	SET domain containing 6, protein lysine methyltra	protein_coding	NP_001153777.1;NP_079136.2;XP_	2.2
NSUN6	NOP2/Sun RNA methyltransferase 6	protein_coding	NP_001338044.1;NP_001338045.1;	2.2
PBDC1	polysaccharide biosynthesis domain containing 1	protein_coding	NP_001287817.1;NP_057584.2	2.2
NF1	neurofibromin 1	protein_coding	NP_000258.1;NP_001035957.1;NP_	2.2
SNORA67	small nucleolar RNA, H/ACA box 67	snoRNA	.	2.2
XRN2	5'-3' exoribonuclease 2	protein_coding	NP_001304889.1;NP_036387.2;XP_	2.2
TSC22D1	TSC22 domain family member 1	protein_coding	NP_001230726.1;NP_001230727.1;	2.2
KNTC1	kinetochore associated 1	protein_coding	NP_055523.1;XP_006719769.1;XP_	2.2
MDM4	MDM4 regulator of p53	protein_coding	NP_001191100.1;NP_001191101.1;	2.2
ARID4A	AT-rich interaction domain 4A	protein_coding	NP_002883.3;NP_075376.2;NP_07:	2.2
RESF1	retroelement silencing factor 1	protein_coding	NP_060639.4;XP_005253462.1;XP_	2.2
APBB2	amyloid beta precursor protein binding family B m	protein_coding	NP_001159522.1;NP_001159523.1;	2.2
TNRC6A	trinucleotide repeat containing adaptor 6A	protein_coding	NP_001317449.1;NP_001338779.1;	2.2
RPS6KB1	ribosomal protein S6 kinase B1	protein_coding	NP_001258971.1;NP_001258972.1;	2.2
SNORD47	small nucleolar RNA, C/D box 47	snoRNA	.	2.2
PTPRG-AS1	PTPRG antisense RNA 1	lncRNA	.	2.2
NUMB	NUMB endocytic adaptor protein	protein_coding	NP_001005743.1;NP_001005744.1;	2.2
EIF2AK3	eukaryotic translation initiation factor 2 alpha kina	protein_coding	NP_001300844.1;NP_004827.4;XP_	2.2
HIF1A-AS3	HIF1A antisense RNA 3	lncRNA	.	2.2
DDHD1	DDHD domain containing 1	protein_coding	NP_001153619.1;NP_001153620.1;	2.2
CEP63	centrosomal protein 63	protein_coding	NP_001035842.1;NP_001035843.1;	2.2
RIOK1	RIO kinase 1	protein_coding	NP_001335123.1;NP_113668.2;XP_	2.2
LINC00472	long intergenic non-protein coding RNA 472	lncRNA	.	2.2
FTCDNL1	formiminotransferase cyclodeaminase N-terminal	protein_coding	NP_001337782.1;NP_001337783.1;	2.2
RPL37	ribosomal protein L37	protein_coding	NP_000988.1	2.2
THAP9-AS1	THAP9 antisense RNA 1	lncRNA	.	2.2
GAB1	GRB2 associated binding protein 1	protein_coding	NP_002030.2;NP_997006.1;XP_00	2.2
MSANTD3-TMEFF1	MSANTD3-TMEFF1 readthrough	protein_coding	NP_001185741.1	2.2
ANK2	ankyrin 2	protein_coding	NP_001120965.1;NP_001139.3;NP_	2.2
INO80D	INO80 complex subunit D	protein_coding	NP_060229.3;XP_011509671.1;XP_	2.2
KLHL38	kelch like family member 38	protein_coding	NP_001075144.2	2.2
MAP3K7CL	MAP3K7 C-terminal like	protein_coding	NP_001273546.1;NP_001273547.1;	2.2
ADCY2	adenylate cyclase 2	protein_coding	NP_065433.2;XP_011512244.1	2.2
FAM107B	family with sequence similarity 107 member B	protein_coding	NP_001269624.1;NP_001269625.1;	2.2
RPL17P6	ribosomal protein L17 pseudogene 6	pseudogene	.	2.2
FBXO25	F-box protein 25	protein_coding	NP_036305.2;NP_904356.1;NP_90	2.2
BBS2	Bardet-Biedl syndrome 2	protein_coding	NP_001364385.1;NP_114091.4	2.2
RNF213	ring finger protein 213	protein_coding	NP_001243000.2;NP_066005.2;XP_	2.2
LOC105374811	uncharacterized LOC105374811, transcript varian	lncRNA	.	2.2
ZFP69B	ZFP69 zinc finger protein B	protein_coding	NP_001356494.1;NP_075558.2;XP_	2.2
ZNF140	zinc finger protein 140	protein_coding	NP_001287705.1;NP_001287706.1;	2.2
OVOS2	alpha-2-macroglobulin like 1 pseudogene	ranscribed_pseudogen	.	2.2
LINC02832	long intergenic non-protein coding RNA 2832	lncRNA	.	2.2
PVT1	Pvt1 oncogene	lncRNA	.	2.2
CHD6	chromodomain helicase DNA binding protein 6	protein_coding	NP_115597.3;XP_005260630.1;XP_	2.2
HEXA-AS1	HEXA antisense RNA 1	lncRNA	.	2.2
UCHL3	ubiquitin C-terminal hydrolase L3	protein_coding	NP_001257881.1;NP_005993.1;XP_	2.2
WDFY3	WD repeat and FYVE domain containing 3	protein_coding	NP_055806.2;XP_005262915.1;XP_	2.2
DSCR9	Down syndrome critical region 9	lncRNA	.	2.2
PARP4	poly(ADP-ribose) polymerase family member 4	protein_coding	NP_006428.2;XP_011533233.1;XP_	2.2
TMTC2	transmembrane O-mannosyltransferase targeting c	protein_coding	NP_001307250.1;NP_001307251.1;	2.2
ZNF441	zinc finger protein 441	protein_coding	NP_689568.2	2.2
THUMPD3-AS1	THUMPD3 antisense RNA 1	lncRNA	.	2.2
WASHC4	WASH complex subunit 4	protein_coding	NP_001280569.1;NP_056090.1;XP_	2.2
MBD5	methyl-CpG binding domain protein 5	protein_coding	NP_001365049.1;NP_060798.2;XP_	2.2
SLC11A2	solute carrier family 11 member 2	protein_coding	NP_000608.1;NP_001167596.1;NP_	2.2
NSUN5P1	NSUN5 pseudogene 1	ranscribed_pseudogen	.	2.2
PPP1R9A	protein phosphatase 1 regulatory subunit 9A	protein_coding	NP_001159632.1;NP_001159633.1;	2.2
STAT1	signal transducer and activator of transcription 1	protein_coding	NP_009330.1;NP_644671.1;XP_00	2.2
LOC100126784	uncharacterized LOC100126784	lncRNA	.	2.2
TIFA	TRAF interacting protein with forkhead associatec	protein_coding	NP_443096.1	2.2
LOC107986198	uncharacterized LOC107986198	lncRNA	.	2.2
LINC01670	long intergenic non-protein coding RNA 1670	lncRNA	.	2.2
CFAP57	cilia and flagella associated protein 57	protein_coding	NP_001161437.1;NP_001182760.2;	2.2

NCOR1	nuclear receptor corepressor 1	protein_coding	NP_001177367.1;NP_001177369.1;	2.2
LOC107987272	uncharacterized LOC107987272	lncRNA	.	2.2
EVI2A	ecotropic viral integration site 2A	protein_coding	NP_001003927.1;NP_055025.2	2.2
MYNN	myoneurin	protein_coding	NP_001172047.1;NP_001172048.1;	2.2
ZFYVE16	zinc finger FYVE-type containing 16	protein_coding	NP_001098721.2;NP_001271165.2;	2.2
ZNF550	zinc finger protein 550	protein_coding	NP_001264019.1;NP_001264020.1;	2.2
CASP4LP	caspase 4 like, pseudogene	ranscribed_pseudogen	.	2.2
LOC105374298	uncharacterized LOC105374298, transcript varian	lncRNA	.	2.2
ZNF804A	zinc finger protein 804A	protein_coding	NP_919226.1	2.2
LYAR	Ly1 antibody reactive	protein_coding	NP_001139197.1;NP_060286.1;XP_	2.2
SCLT1	sodium channel and clathrin linker 1	protein_coding	NP_001287826.1;NP_001287827.1;	2.2
NIPBL	NIPBL cohesin loading factor	protein_coding	NP_056199.2;NP_597677.2;XP_00	2.2
FBXL2	F-box and leucine rich repeat protein 2	protein_coding	NP_001336245.1;NP_001336248.1;	2.2
LOC101927610	uncharacterized LOC101927610, transcript varian	lncRNA	.	2.2
LOC102723480	uncharacterized LOC102723480, transcript varian	lncRNA	.	2.2
MTRR	5-methyltetrahydrofolate-homocysteine methyltra	protein_coding	NP_001351369.1;NP_001351370.1;	2.2
RSRC1	arginine and serine rich coiled-coil 1	protein_coding	NP_001258763.1;NP_001258767.1;	2.2
LINC02454	long intergenic non-protein coding RNA 2454	lncRNA	.	2.2
LONRF2	LON peptidase N-terminal domain and ring finger	protein_coding	NP_001358712.1;NP_940863.3	2.2
JMY	junction mediating and regulatory protein, p53 cof	protein_coding	NP_689618.4;XP_005248487.1;XP_	2.2
LOC100288069	uncharacterized LOC100288069	lncRNA	.	2.2
RAB3IP	RAB3A interacting protein	protein_coding	NP_001019818.1;NP_001265331.1;	2.2
ARL17B	ADP ribosylation factor like GTPase 17B	protein_coding	NP_001034172.3;NP_001096624.1;	2.2
ZNF565	zinc finger protein 565	protein_coding	NP_001035939.1;NP_001353117.1;	2.2
MB21D2	Mab-21 domain containing 2	protein_coding	NP_848591.2	2.2
LOC107986465	uncharacterized LOC107986465	lncRNA	.	2.2
SYF2	SYF2 pre-mRNA splicing factor	protein_coding	NP_056299.1;NP_997053.1	2.2
SLC25A37	solute carrier family 25 member 37	protein_coding	NP_001304741.1;NP_001304742.1;	2.2
BNC2	basonuclin 2	protein_coding	NP_001304868.1;NP_001304869.1;	2.2
ARMC9	armadillo repeat containing 9	protein_coding	NP_001258395.2;NP_001278585.2;	2.2
RGS5	regulator of G protein signaling 5	protein_coding	NP_001182232.1;NP_001241677.1;	2.2
NCK1	NCK adaptor protein 1	protein_coding	NP_001177725.1;NP_001278928.1;	2.2
SEC22B2P	SEC22 homolog B2, pseudogene	ranscribed_pseudogen	.	2.2
FANCL	FA complementation group L	protein_coding	NP_001108108.1;NP_001361544.1;	2.2
SELENOS	selenoprotein S	protein_coding	NP_060915.2;NP_982298.2	2.2
KLF12	Kruppel like factor 12	protein_coding	NP_009180.3;XP_005266308.1;XP_	2.2
C9orf72	C9orf72-SMCR8 complex subunit	protein_coding	NP_001242983.1;NP_060795.1;NP_	2.2
RNU6-9	RNA, U6 small nuclear 9	snRNA	.	2.2
ZBTB20	zinc finger and BTB domain containing 20	protein_coding	NP_001157814.1;NP_001157815.1;	2.2
HMGCS1	3-hydroxy-3-methylglutaryl-CoA synthase 1	protein_coding	NP_001091742.1;NP_001311148.1;	2.2
GOLGA8O	golgin A8 family member O	protein_coding	NP_001264237.1;XP_011520290.1;	2.2
TLR4	toll like receptor 4	protein_coding	NP_003257.1;NP_612564.1;NP_61	2.2
ZNF337-AS1	ZNF337 antisense RNA 1	lncRNA	.	2.2
USP3	ubiquitin specific peptidase 3	protein_coding	NP_001243631.1;NP_006528.2;XP_	2.2
RAPGEF6	Rap guanine nucleotide exchange factor 6	protein_coding	NP_001157858.1;NP_001157859.1;	2.2
STC2	stanniocalcin 2	protein_coding	NP_003705.1	2.2
CCDC144A	coiled-coil domain containing 144A	protein_coding	NP_001368929.1;NP_055510.1;XP_	2.2
ST3GAL3	ST3 beta-galactoside alpha-2,3-sialyltransferase 3	protein_coding	NP_001257388.1;NP_001257389.1;	2.2
GTF2IRD2B	GTF2I repeat domain containing 2B	protein_coding	NP_001003795.1;NP_001355230.1;	2.2
ANAPC10	anaphase promoting complex subunit 10	protein_coding	NP_001243635.1;NP_001243636.1;	2.2
APTR	Alu-mediated CDKN1A/p21 transcriptional regula	lncRNA	.	2.2
ZNF860	zinc finger protein 860	protein_coding	NP_001131146.2;XP_016861788.1	2.2
PLEKHA1	pleckstrin homology domain containing A1	protein_coding	NP_001001974.1;NP_001182537.1;	2.2
PCNX2	pecanex 2	protein_coding	NP_001315536.1;NP_055616.3;XP_	2.2
LOC107985869	uncharacterized LOC107985869	lncRNA	.	2.2
HMGN1P1	high mobility group nucleosome binding domain 1	pseudogene	.	2.2
ARFGEF1	ADP ribosylation factor guanine nucleotide excha	protein_coding	NP_006412.2;XP_005251191.1;XP_	2.2
USP54	ubiquitin specific peptidase 54	protein_coding	NP_001307366.1;NP_001307370.1;	2.2
GTDC1	glycosyltransferase like domain containing 1	protein_coding	NP_001006637.1;NP_001158101.1;	2.2
EDN1	endothelin 1	protein_coding	NP_001161791.1;NP_001946.3;XP_	2.2
CLCN5	chloride voltage-gated channel 5	protein_coding	NP_000075.1;NP_001121370.1;NP_	2.2
PRMT9	protein arginine methyltransferase 9	protein_coding	NP_001291387.1;NP_001337070.1;	2.2
LOC107987192	uncharacterized LOC107987192	lncRNA	.	2.2
FKBP15	FKBP prolyl isomerase 15	protein_coding	NP_056073.1;XP_006717081.1;XP_	2.2
ACACA	acetyl-CoA carboxylase alpha	protein_coding	NP_942131.1;NP_942133.1;NP_94	2.2
SNHG1	small nucleolar RNA host gene 1	lncRNA	.	2.2
OARD1	O-acyl-ADP-ribose deacylase 1	protein_coding	NP_001316613.1;NP_001316614.1;	2.2
KIRREL1-IT1	KIRREL1 intronic transcript 1	lncRNA	.	2.2
PAXBP1	PAX3 and PAX7 binding protein 1	protein_coding	NP_037461.2;NP_057715.2;XP_00	2.2
ZDHHC6	zinc finger DHHC-type palmitoyltransferase 6	protein_coding	NP_001290063.1;NP_001338011.1;	2.2
HERC4	HECT and RLD domain containing E3 ubiquitin p	protein_coding	NP_001265114.1;NP_001265115.1;	2.2

ZFY	zinc finger protein Y-linked	protein_coding	NP_001138747.1;NP_001138748.1;	2.2
SNORA18	small nucleolar RNA, H/ACA box 18	snoRNA	.	2.2
NUP35	nucleoporin 35	protein_coding	NP_001274513.1;NP_001274514.1;	2.2
BRWD1	bromodomain and WD repeat domain containing 1	protein_coding	NP_001007247.1;NP_061836.2;NP_	2.2
AKAP13	A-kinase anchoring protein 13	protein_coding	NP_001257475.1;NP_006729.4;NP_	2.2
LOC643562	Rho GTPase activating protein 21 pseudogene	pseudogene	.	2.1
ARID2	AT-rich interaction domain 2	protein_coding	NP_001334768.1;NP_689854.2;XP_	2.1
SSUH2	ssu-2 homolog	protein_coding	NP_001243677.1;NP_001243678.1;	2.1
ARHGAP29	Rho GTPase activating protein 29	protein_coding	NP_001315593.1;NP_001315594.1;	2.1
SNORD29	small nucleolar RNA, C/D box 29	snoRNA	.	2.1
TMEM135	transmembrane protein 135	protein_coding	NP_001162195.1;NP_075069.3;XP_	2.1
IWS1	interacts with SUPT6H, CTD assembly factor 1	protein_coding	NP_060439.2;XP_005263764.1;XP_	2.1
RABEP1	rabaptin, RAB GTPase binding effector protein 1	protein_coding	NP_001077054.1;NP_001278510.1;	2.1
DCP1A	decapping mRNA 1A	protein_coding	NP_001277133.1;NP_001277134.1;	2.1
LOC107985897	uncharacterized LOC107985897	lncRNA	.	2.1
LINS1	lines homolog 1	protein_coding	NP_001035706.2;NP_001339436.1;	2.1
ZFC3H1	zinc finger C3H1-type containing	protein_coding	NP_659419.3	2.1
RCBTB1	RCC1 and BTB domain containing protein 1	protein_coding	NP_001339429.1;NP_001339430.1;	2.1
SP2-AS1	SP2 antisense RNA 1	lncRNA	.	2.1
SNRPE	small nuclear ribonucleoprotein polypeptide E	protein_coding	NP_001291393.1;NP_001315566.1;	2.1
ZDHHC11B	zinc finger DHHC-type containing 11B	protein_coding	NP_001338232.1;XP_016865599.1;	2.1
ZNF266	zinc finger protein 266	protein_coding	NP_001258243.1;NP_001357303.1;	2.1
C22orf46	chromosome 22 open reading frame 46	ranscribed_pseudogen	.	2.1
ADAM17	ADAM metallopeptidase domain 17	protein_coding	NP_001369706.1;NP_001369707.1;	2.1
HNRNPA2B1	heterogeneous nuclear ribonucleoprotein A2/B1	protein_coding	NP_002128.1;NP_112533.1;XP_00	2.1
TRMT13	tRNA methyltransferase 13 homolog	protein_coding	NP_061956.2;XP_005271002.1;XP_	2.1
LRRN1	leucine rich repeat neuronal 1	protein_coding	NP_001311117.1;NP_001311118.1;	2.1
OPHN1	oligophrenin 1	protein_coding	NP_002538.1;XP_005262327.1;XP_	2.1
SLC9B2	solute carrier family 9 member B2	protein_coding	NP_001287683.1;NP_001287685.1;	2.1
SNORA6	small nucleolar RNA, H/ACA box 6	snoRNA	.	2.1
TET2	tet methylcytosine dioxygenase 2	protein_coding	NP_001120680.1;NP_060098.3;XP_	2.1
HDAC2-AS2	HDAC2 and HS3ST5 antisense RNA 2	lncRNA	.	2.1
SCAT1	S-phase cancer associated transcript 1	lncRNA	.	2.1
CEP295	centrosomal protein 295	protein_coding	NP_203753.1;XP_005274423.1;XP_	2.1
ZNF81	zinc finger protein 81	protein_coding	NP_001365081.1;NP_001365082.1;	2.1
CFAP58	cilia and flagella associated protein 58	protein_coding	NP_001008723.1	2.1
NDUFB1	NADH:ubiquinone oxidoreductase subunit B1	protein_coding	NP_004536.3	2.1
DPP4	dipeptidyl peptidase 4	protein_coding	NP_001366533.1;NP_001366534.1;	2.1
ANKDD1A	ankyrin repeat and death domain containing 1A	protein_coding	NP_874362.3	2.1
ZNF720	zinc finger protein 720	protein_coding	NP_001124385.1	2.1
ZNF131	zinc finger protein 131	protein_coding	NP_001284477.1;NP_001317633.1;	2.1
GTF2H1	general transcription factor IIH subunit 1	protein_coding	NP_001135779.1;NP_005307.1;XP_	2.1
MRRF	mitochondrial ribosome recycling factor	protein_coding	NP_001166983.1;NP_001333268.1;	2.1
ARHGAP26	Rho GTPase activating protein 26	protein_coding	NP_001129080.1;NP_001336476.1;	2.1
AUH	AU RNA binding methylglutaconyl-CoA hydratas	protein_coding	NP_001293119.1;NP_001338360.1;	2.1
UBR5	ubiquitin protein ligase E3 component n-recognin	protein_coding	NP_001269802.1;NP_056986.2;XP	2.1
IQCB1	IQ motif containing B1	protein_coding	NP_001018864.2;NP_001018865.2;	2.1
UBR4	ubiquitin protein ligase E3 component n-recognin	protein_coding	NP_065816.2;XP_011539410.2;XP_	2.1
SLC35E1P1	solute carrier family 35 member E1 pseudogene 1	pseudogene	.	2.1
LOC105376718	uncharacterized LOC105376718	lncRNA	.	2.1
TSGA10	testis specific 10	protein_coding	NP_001335941.1;NP_001335942.1;	2.1
ZFAND2A	zinc finger AN1-type containing 2A	protein_coding	NP_001352310.1;NP_001352312.1;	2.1
SPRY2	sprouty RTK signaling antagonist 2	protein_coding	NP_001305465.1;NP_001305466.1;	2.1
IQGAP1	IQ motif containing GTPase activating protein 1	protein_coding	NP_003861.1	2.1
KRT8P33	keratin 8 pseudogene 33	pseudogene	.	2.1
APOL3	apolipoprotein L3	protein_coding	NP_055164.1;NP_085147.1;NP_66	2.1
LOC107984421	uncharacterized LOC107984421, transcript varian	lncRNA	.	2.1
LOC105373645	uncharacterized LOC105373645	lncRNA	.	2.1
TM4SF19-TCTEX1D	TM4SF19-TCTEX1D2 readthrough (NMD candic	lncRNA	.	2.1
ACTR3B	actin related protein 3B	protein_coding	NP_001035225.1;NP_001337869.1;	2.1
DZANK1	double zinc ribbon and ankyrin repeat domains 1	protein_coding	NP_001092877.1;NP_001338612.1;	2.1
LINC02018	long intergenic non-protein coding RNA 2018	lncRNA	.	2.1
MANF	mesencephalic astrocyte derived neurotrophic fact	protein_coding	NP_006001.5	2.1
TMEM67	transmembrane protein 67	protein_coding	NP_001135773.1;NP_714915.3;XP_	2.1
NBEAL1	neurobeachin like 1	protein_coding	NP_001107604.1;NP_001364955.1;	2.1
GPR89B	G protein-coupled receptor 89B	protein_coding	NP_001337109.1;NP_001337110.1;	2.1
NSD3	nuclear receptor binding SET domain protein 3	protein_coding	NP_060248.2;NP_075447.1	2.1
MED23	mediator complex subunit 23	protein_coding	NP_001257450.1;NP_001257451.1;	2.1
RP9	RP9 pre-mRNA splicing factor	protein_coding	NP_976033.1;XP_011513770.1	2.1
ZNF600	zinc finger protein 600	protein_coding	NP_001308795.1;NP_001308796.1;	2.1
LOC107985942	uncharacterized LOC107985942	lncRNA	.	2.1

UIMC1	ubiquitin interaction motif containing 1	protein_coding	NP_001186226.1;NP_001186227.1;	2.1
ZNF473	zinc finger protein 473	protein_coding	NP_001006657.1;NP_001295353.1;	2.1
CDC26	cell division cycle 26	protein_coding	NP_644815.1;XP_016870062.1;XP_	2.1
ENOX2	ecto-NOX disulfide-thiol exchanger 2	protein_coding	NP_001268665.1;NP_001369445.1;	2.1
PRDM10	PR/SET domain 10	protein_coding	NP_001354819.1;NP_001354820.1;	2.1
ZNF26	zinc finger protein 26	protein_coding	NP_001243208.1;NP_001243209.1;	2.1
BMS1P20	BMS1 pseudogene 20	ranscribed_pseudogen	.	2.1
TSC1	TSC complex subunit 1	protein_coding	NP_000359.1;NP_001155898.1;NP_	2.1
ZNF182	zinc finger protein 182	protein_coding	NP_001007089.1;NP_001171570.1;	2.1
PLB1	phospholipase B1	protein_coding	NP_001164056.1;NP_694566.4;XP_	2.1
NLGN3	neuroligin 3	protein_coding	NP_001160132.1;NP_001308205.1;	2.1
CEP112	centrosomal protein 112	protein_coding	NP_001032402.1;NP_001186094.1;	2.1
RWDD2A	RWD domain containing 2A	protein_coding	NP_001309264.1;NP_001309265.1;	2.1
PMS1	PMS1 homolog 1, mismatch repair system compo	protein_coding	NP_000525.1;NP_001121615.1;NP_	2.1
MYOSLID-AS1	MYOSLID antisense RNA 1	lncRNA	.	2.1
RPS4Y1	ribosomal protein S4 Y-linked 1	protein_coding	NP_000999.1	2.1
SPIN2A	spindlin family member 2A	protein_coding	NP_061876.3;XP_005262073.3;XP_	2.1
NEMF	nuclear export mediator factor	protein_coding	NP_001288661.1;NP_004704.3;XP_	2.1
CCAR1	cell division cycle and apoptosis regulator 1	protein_coding	NP_001269888.1;NP_001269889.1;	2.1
MIA2	MIA SH3 domain ER export factor 2	protein_coding	NP_001234917.1;NP_001234918.1;	2.1
MCF2L2	MCF.2 cell line derived transforming sequence-lik	protein_coding	NP_055893.3;XP_011510887.1;XP_	2.1
SRSF5	serine and arginine rich splicing factor 5	protein_coding	NP_001034554.1;NP_001307143.1;	2.1
LOC107985360	uncharacterized LOC107985360	lncRNA	.	2.1
DENND11	DENN domain containing 11	protein_coding	NP_001073861.1;XP_024302611.1	2.1
RAB6D	RAB6D, member RAS oncogene family	protein_coding	NP_001071105.1	2.1
RUFY3	RUN and FYVE domain containing 3	protein_coding	NP_001032519.1;NP_001124181.1;	2.1
CLSPN	claspin	protein_coding	NP_001177410.1;NP_001317419.1;	2.1
NSMCE2	NSE2 (MMS21) homolog, SMC5-SMC6 complex	protein_coding	NP_001336414.1;NP_001336415.1;	2.1
ALPK3	alpha kinase 3	protein_coding	NP_065829.4	2.1
GNL3	G protein nucleolar 3	protein_coding	NP_055181.3;NP_996561.1;NP_99	2.1
LARP1B	La ribonucleoprotein 1B	protein_coding	NP_001265533.1;NP_001337460.1;	2.1
ZDBF2	zinc finger DBF-type containing 2	protein_coding	NP_001272478.1;NP_001356583.1;	2.1
EIF4G3	eukaryotic translation initiation factor 4 gamma 3	protein_coding	NP_001185730.1;NP_001185731.1;	2.1
ZNF165	zinc finger protein 165	protein_coding	NP_001363420.1;NP_001363421.1;	2.1
AGO4	argonaute RISC component 4	protein_coding	NP_060099.2;XP_005270635.1;XP_	2.1
NMNAT2	nicotinamide nucleotide adenylyltransferase 2	protein_coding	NP_055854.1;NP_733820.1;XP_02	2.1
EIF3A	eukaryotic translation initiation factor 3 subunit A	protein_coding	NP_003741.1	2.1
ARHGAP18	Rho GTPase activating protein 18	protein_coding	NP_277050.2;XP_005267269.2;XP_	2.1
OSMR	oncostatin M receptor	protein_coding	NP_001161827.1;NP_001310433.1;	2.1
ST3GAL5	ST3 beta-galactoside alpha-2,3-sialyltransferase 5	protein_coding	NP_001035902.1;NP_001341152.1;	2.1
PIGA	phosphatidylinositol glycan anchor biosynthesis cl	protein_coding	NP_002632.1;NP_065206.3;XP_01	2.1
LOC105378756	uncharacterized LOC105378756, transcript varian	lncRNA	.	2.1
DOCK2	dedicator of cytokinesis 2	protein_coding	NP_004937.1;XP_005265887.1;XP_	2.1
RAB13	RAB13, member RAS oncogene family	protein_coding	NP_001258967.1;NP_002861.1;XP_	2.1
BTF3L4P2	basic transcription factor 3 like 4 pseudogene 2	pseudogene	.	2.1
C8orf48	chromosome 8 open reading frame 48	protein_coding	NP_001007091.2	2.1
MOK	MOK protein kinase	protein_coding	NP_001258940.1;NP_001317163.1;	2.1
LOC105374746	uncharacterized LOC105374746	lncRNA	.	2.1
ETV5	ETS variant transcription factor 5	protein_coding	NP_004445.1	2.1
SNORD110	small nucleolar RNA, C/D box 110	snoRNA	.	2.1
DYNC2I1	dynein 2 intermediate chain 1	protein_coding	NP_001337843.1;NP_001337844.1;	2.1
RBBP6	RB binding protein 6, ubiquitin ligase	protein_coding	NP_008841.2;NP_061173.1;NP_11	2.1
HMGB1P6	high mobility group box 1 pseudogene 6	pseudogene	.	2.1
COL12A1	collagen type XII alpha 1 chain	protein_coding	NP_004361.3;NP_542376.2;XP_01	2.1
EXOG	exo/endonuclease G	protein_coding	NP_001138936.1;NP_005098.2;XP_	2.1
EPGN	epithelial mitogen	protein_coding	NP_001257918.1;NP_001257919.1;	2.1
LOC729291	uncharacterized LOC729291	lncRNA	.	2.1
ABCA11P	ATP binding cassette subfamily A member 11, ps	ranscribed_pseudogen	.	2.1
GS1-124K5.11	RAB guanine nucleotide exchange factor 1 pseud	ranscribed_pseudogen	.	2.1
LOC107986411	uncharacterized LOC107986411	lncRNA	.	2.1
KLHL24	kelch like family member 24	protein_coding	NP_001336342.1;NP_001336343.1;	2.1
SECISBP2	SECIS binding protein 2	protein_coding	NP_001269617.1;NP_001269618.1;	2.1
BTN2A1	butyrophilin subfamily 2 member A1	protein_coding	NP_001184162.1;NP_001184163.1;	2.1
AK9	adenylate kinase 9	protein_coding	NP_001138600.2;NP_001316531.1;	2.1
ZNF674	zinc finger protein 674	protein_coding	NP_001034980.1;NP_001139763.1;	2.1
ZNF57	zinc finger protein 57	protein_coding	NP_001306012.1;NP_775751.1;XP_	2.1
PID1	phosphotyrosine interaction domain containing 1	protein_coding	NP_001094288.1;NP_001317085.1;	2.1
HMGN1P18	high mobility group nucleosome binding domain 1	pseudogene	.	2.1
AP1G2	adaptor related protein complex 1 subunit gamma	protein_coding	NP_001269403.1;NP_001269404.1;	2.1
MED12L	mediator complex subunit 12L	protein_coding	NP_443728.3;XP_006713550.1;XP_	2.1
CKMT2-AS1	CKMT2 antisense RNA 1	lncRNA	.	2.1

SRBD1	S1 RNA binding domain 1	protein_coding	NP_060549.4;XP_011531248.1;XP_	2.1
FAM204BP	family with sequence similarity 204 member B, ps	pseudogene	.	2.1
SLC35G2	solute carrier family 35 member G2	protein_coding	NP_001091068.1;NP_001091069.1;	2.1
RMDN2	regulator of microtubule dynamics 2	protein_coding	NP_001164262.1;NP_001164263.1;	2.1
THAP12P7	THAP domain containing 12 pseudogene 7	pseudogene	.	2.1
LOC105374724	uncharacterized LOC105374724, transcript varian	lncRNA	.	2.1
IK	IK cytokine	protein_coding	NP_006074.2	2.1
REV3L	REV3 like, DNA directed polymerase zeta catalyti	protein_coding	NP_001273360.1;NP_001273361.1;	2.1
BTAF1	B-TFIID TATA-box binding protein associated fa	protein_coding	NP_003963.1;XP_011538628.1;XP_	2.1
CDHR3	cadherin related family member 3	protein_coding	NP_001288090.1;NP_689963.2;XP_	2.1
CFLAR	CASP8 and FADD like apoptosis regulator	protein_coding	NP_001120655.1;NP_001120656.1;	2.1
LOC107986350	uncharacterized LOC107986350	lncRNA	.	2.1
LOC105375785	uncharacterized LOC105375785, transcript varian	lncRNA	.	2.1
EXOC2	exocyst complex component 2	protein_coding	NP_060773.3;XP_016866507.1;XP_	2.1
ZNF551	zinc finger protein 551	protein_coding	NP_001257867.1;NP_612356.2	2.1
HLCS	holocarboxylase synthetase	protein_coding	NP_000402.3;NP_001229713.1;NP_	2.1
CHRNA10	cholinergic receptor nicotinic alpha 10 subunit	protein_coding	NP_001289963.1;NP_001289964.1;	2.1
MITD1	microtubule interacting and trafficking domain co	protein_coding	NP_001307346.1;NP_001307347.1;	2.1
PSMD7	proteasome 26S subunit, non-ATPase 7	protein_coding	NP_002802.2	2.1
FNDC3B	fibronectin type III domain containing 3B	protein_coding	NP_001128567.1;NP_073600.3;XP_	2.1
FAM156B	family with sequence similarity 156 member B	protein_coding	NP_001093154.1;NP_001308107.1;	2.1
NBAS	NBAS subunit of NRZ tethering complex	protein_coding	NP_056993.2;XP_011508659.1;XP_	2.1
UQCRB	ubiquinol-cytochrome c reductase binding protein	protein_coding	NP_001186904.1;NP_001241681.1;	2.1
DPYD	dihydropyrimidine dehydrogenase	protein_coding	NP_000101.2;NP_001153773.1;XP_	2.1
TM4SF19	transmembrane 4 L six family member 19	protein_coding	NP_001191826.1;NP_001191827.1;	2.1
ARL13B	ADP ribosylation factor like GTPase 13B	protein_coding	NP_001167621.1;NP_001167622.1;	2.1
LINC01588	long intergenic non-protein coding RNA 1588	lncRNA	.	2.1
GEN1	GEN1 Holliday junction 5' flap endonuclease	protein_coding	NP_001123481.3;NP_872431.5;XP_	2.1
MRPS31	mitochondrial ribosomal protein S31	protein_coding	NP_005821.2	2.1
LOC105372553	uncharacterized LOC105372553	lncRNA	.	2.1
NASP	nuclear autoantigenic sperm protein	protein_coding	NP_001182122.1;NP_002473.2;NP_	2.1
ACSL4	acyl-CoA synthetase long chain family member 4	protein_coding	NP_001305438.1;NP_001305439.1;	2.1
GOLGA5	golgin A5	protein_coding	NP_005104.4;XP_011535722.1	2.1
DYRK1A	dual specificity tyrosine phosphorylation regulatec	protein_coding	NP_001334650.1;NP_001334651.1;	2.1
SNX24	sorting nexin 24	protein_coding	NP_054754.1;XP_005272029.1;XP_	2.1
BBS4	Bardet-Biedl syndrome 4	protein_coding	NP_001239607.1;NP_001307594.1;	2.1
ZNF852	zinc finger protein 852	protein_coding	NP_001274278.1	2.1
FAM66D	family with sequence similarity 66 member D	lncRNA	.	2.1
ZNF736	zinc finger protein 736	protein_coding	NP_001164376.1;NP_001281184.1	2.1
ELP2	elongator acetyltransferase complex subunit 2	protein_coding	NP_001229804.1;NP_001229805.1;	2.1
ZBED3-AS1	ZBED3 antisense RNA 1	lncRNA	.	2.1
ABCC2	ATP binding cassette subfamily C member 2	protein_coding	NP_000383.2;XP_006717693.1;XP_	2.1
LOC100421597	peptidylprolyl isomerase like 4 pseudogene	pseudogene	.	2.1
AGGF1P3	angiogenic factor with G-patch and FHA domains	pseudogene	.	2.0
RPS10P19	ribosomal protein S10 pseudogene 19	pseudogene	.	2.0
SIGLEC15	sialic acid binding Ig like lectin 15	protein_coding	NP_998767.1	2.0
CEP162	centrosomal protein 162	protein_coding	NP_001273135.1;NP_055710.2;XP_	2.0
LOC105377826	uncharacterized LOC105377826, transcript varian	lncRNA	.	2.0
CLK1	CDC like kinase 1	protein_coding	NP_001155879.1;NP_004062.2	2.0
LOC107986998	uncharacterized LOC107986998	lncRNA	.	2.0
NAA35	N-alpha-acetyltransferase 35, NatC auxiliary subu	protein_coding	NP_001308810.1;NP_001308811.1;	2.0
ZBTB43	zinc finger and BTB domain containing 43	protein_coding	NP_001129248.1;NP_054726.1;XP_	2.0
METTL3	methyltransferase like 3	protein_coding	NP_062826.2;XP_006720269.1;XP_	2.0
POGLUT1	protein O-glucosyltransferase 1	protein_coding	NP_689518.1;XP_006713768.1;XP_	2.0
SLC28A3	solute carrier family 28 member 3	protein_coding	NP_001186562.1;NP_071410.1;XP_	2.0
CFAP36	cilia and flagella associated protein 36	protein_coding	NP_001269690.1;NP_542398.3;XP_	2.0
MAP3K4	mitogen-activated protein kinase kinase kinase 4	protein_coding	NP_001278887.1;NP_001288001.1;	2.0
LOC101927932	uncharacterized LOC101927932	lncRNA	.	2.0
MARF1	meiosis regulator and mRNA stability factor 1	protein_coding	NP_001171927.1;NP_001171928.1;	2.0
DYNC2H1	dynein cytoplasmic 2 heavy chain 1	protein_coding	NP_001073932.1;NP_001368.2;XP_	2.0
LOC105372210	uncharacterized LOC105372210	lncRNA	.	2.0
ZNF226	zinc finger protein 226	protein_coding	NP_001027544.1;NP_001027545.1;	2.0
ST3GAL6	ST3 beta-galactoside alpha-2,3-sialyltransferase 6	protein_coding	NP_001258071.1;NP_001258074.1;	2.0
BEND7	BEN domain containing 7	protein_coding	NP_001094382.1;NP_001356792.1;	2.0
IL18BP	interleukin 18 binding protein	protein_coding	NP_001034748.1;NP_001034749.1;	2.0
LEKR1	leucine, glutamate and lysine rich 1	protein_coding	NP_001004316.2;NP_001180212.1;	2.0
MGC12916	uncharacterized protein MGC12916	lncRNA	.	2.0
LARS1	leucyl-tRNA synthetase 1	protein_coding	NP_001304893.1;NP_001304894.1;	2.0
MORC3	MORC family CW-type zinc finger 3	protein_coding	NP_001307374.1;NP_001307375.1;	2.0
WTAP	WT1 associated protein	protein_coding	NP_001257460.1;NP_001257461.1;	2.0
THSD7B	thrombospondin type 1 domain containing 7B	protein_coding	NP_001303278.1;XP_016860538.1	2.0

GPR68	G protein-coupled receptor 68	protein_coding	NP_001171147.1;NP_001335366.1;	2.0
ZNF69	zinc finger protein 69	protein_coding	NP_001308092.1;NP_001351659.1;	2.0
TYW1	tRNA-yW synthesizing protein 1 homolog	protein_coding	NP_060734.2;XP_011514674.1;XP_	2.0
ZUP1	zinc finger containing ubiquitin peptidase 1	protein_coding	NP_001348118.1;NP_001348119.1;	2.0
CHMP5	charged multivesicular body protein 5	protein_coding	NP_001182465.1;NP_057494.3	2.0
CDCP1	CUB domain containing protein 1	protein_coding	NP_073753.3;NP_835488.1;XP_01	2.0
CDH12	cadherin 12	protein_coding	NP_001304156.1;NP_001304157.1;	2.0
ALMS1	ALMS1 centrosome and basal body associated prc	protein_coding	NP_001365383.1;NP_055935.4	2.0
GON4L	gon-4 like	protein_coding	NP_001269785.1;NP_001269787.1;	2.0
CYLD	CYLD lysine 63 deubiquitinase	protein_coding	NP_001035814.1;NP_001035877.1;	2.0
KRIT1	KRIT1 ankyrin repeat containing	protein_coding	NP_001013424.1;NP_001337598.1;	2.0
NEDD9	neural precursor cell expressed, developmentally c	protein_coding	NP_001135865.1;NP_001257962.1;	2.0
SEC22B	SEC22 homolog B, vesicle trafficking protein	protein_coding	NP_004883.3;XP_016858345.1	2.0
CCDC169	coiled-coil domain containing 169	protein_coding	NP_001138453.1;NP_001138454.1;	2.0
LOC105379337	uncharacterized LOC105379337	lncRNA	.	2.0
TRPS1	transcriptional repressor GATA binding 1	protein_coding	NP_001269831.1;NP_001269832.1;	2.0
TES	testin LIM domain protein	protein_coding	NP_056456.1;NP_690042.1;XP_00	2.0
DNTTIP2	deoxynucleotidyltransferase terminal interacting p	protein_coding	NP_055412.2	2.0
NPIPB5	nuclear pore complex interacting protein family m	protein_coding	NP_001129337.1	2.0
TBCK	TBC1 domain containing kinase	protein_coding	NP_001156907.2;NP_001156908.2;	2.0
SNORD15B	small nucleolar RNA, C/D box 15B	snoRNA	.	2.0
DICER1	dicer 1, ribonuclease III	protein_coding	NP_001182502.1;NP_001258211.1;	2.0
SH2B3	SH2B adaptor protein 3	protein_coding	NP_001278353.1;NP_005466.1;XP_	2.0
RPL23AP21	ribosomal protein L23a pseudogene 21	pseudogene	.	2.0
EPB41L4A-AS1	EPB41L4A antisense RNA 1	lncRNA	.	2.0
ATF2	activating transcription factor 2	protein_coding	NP_001243019.1;NP_001243020.1;	2.0
N4BP2	NEDD4 binding protein 2	protein_coding	NP_001305288.1;NP_060647.2;XP_	2.0
AKAP10	A-kinase anchoring protein 10	protein_coding	NP_001317081.1;NP_009133.2	2.0
KLHL2	kelch like family member 2	protein_coding	NP_001154993.1;NP_001154994.1;	2.0
ZMYM6	zinc finger MYM-type containing 6	protein_coding	NP_009098.3	2.0
AKAP6	A-kinase anchoring protein 6	protein_coding	NP_004265.3;XP_011535680.1;XP_	2.0
KRT34	keratin 34	protein_coding	NP_066293.2;XP_011523095.1	2.0
FMNL2	formin like 2	protein_coding	NP_443137.2;XP_005246320.1;XP_	2.0
LEPR	leptin receptor	protein_coding	NP_001003679.1;NP_001003680.1;	2.0
PAK1IP1	PAK1 interacting protein 1	protein_coding	NP_060376.2;XP_005249261.1;XP_	2.0
RSPH4A	radial spoke head component 4A	protein_coding	NP_001010892.1;NP_001155136.1;	2.0
EZH2	enhancer of zeste 2 polycomb repressive complex	protein_coding	NP_001190176.1;NP_001190177.1;	2.0
ITGA11	integrin subunit alpha 11	protein_coding	NP_001004439.1;XP_005254285.1;	2.0
ASAH2	N-acylsphingosine amidohydrolase 2	protein_coding	NP_001137446.1;NP_063946.2;XP_	2.0
PNPT1	polyribonucleotide nucleotidyltransferase 1	protein_coding	NP_149100.2;XP_005264686.1;XP_	2.0
GIGYF2	GRB10 interacting GYF protein 2	protein_coding	NP_001096616.1;NP_001096617.1;	2.0
EPB41L2	erythrocyte membrane protein band 4.1 like 2	protein_coding	NP_001129026.1;NP_001129027.1;	2.0
NDNF	neuron derived neurotrophic factor	protein_coding	NP_078850.3;XP_024309980.1;XP_	2.0
PPIB	peptidylprolyl isomerase B	protein_coding	NP_000933.1	2.0
KCNJ2	potassium inwardly rectifying channel subfamily J	protein_coding	NP_000882.1	2.0
UTP14A	UTP14A small subunit processome component	protein_coding	NP_001159693.1;NP_006640.2;XP_	2.0
NFE2L3	nuclear factor, erythroid 2 like 3	protein_coding	NP_004280.5	2.0
LOC107987150	uncharacterized LOC107987150	lncRNA	.	2.0
FTO	FTO alpha-ketoglutarate dependent dioxygenase	protein_coding	NP_001073901.1;NP_001350820.1;	2.0
LOC107984350	.	pseudogene	.	2.0
NBPF3	NBPF member 3	protein_coding	NP_001243345.1;NP_001243346.1;	2.0
TOPORS	TOP1 binding arginine/serine rich protein, E3 ubic	protein_coding	NP_001182551.1;NP_005793.2	2.0
RGPD6	RANBP2 like and GRIP domain containing 6	protein_coding	NP_001032955.1;NP_001116835.1;	2.0
POPDC3	popeye domain containing 3	protein_coding	NP_071756.2;XP_011534369.1;XP_	2.0
ACP6	acid phosphatase 6, lysophosphatidic	protein_coding	NP_001310554.1;NP_057445.4;XP_	2.0
CLCN6	chloride voltage-gated channel 6	protein_coding	NP_001243888.2;NP_001277.2	2.0
TNFRSF21	TNF receptor superfamily member 21	protein_coding	NP_055267.1;XP_016866233.1	-2.0
RILPL2	Rab interacting lysosomal protein like 2	protein_coding	NP_659495.1;XP_011536314.1	-2.0
ASCC2	activating signal cointegrator 1 complex subunit 2	protein_coding	NP_001229835.1;NP_001356849.1;	-2.0
BSCL2	BSCL2 lipid droplet biogenesis associated, seipin	protein_coding	NP_001116427.1;NP_001124174.2;	-2.0
LOC154761	family with sequence similarity 115, member C ps	ranscribed_pseudogen	.	-2.0
PMF1-BGLAP	PMF1-BGLAP readthrough	protein_coding	NP_001186590.1;NP_001186591.1;	-2.0
ZNF608	zinc finger protein 608	protein_coding	NP_065798.2;XP_005272094.1;XP_	-2.0
CYB5RL	cytochrome b5 reductase like	protein_coding	NP_001026842.2;NP_001340282.1;	-2.0
STX5	syntaxin 5	protein_coding	NP_001231595.1;NP_001317223.1;	-2.0
RNF10	ring finger protein 10	protein_coding	NP_001317403.1;NP_055683.3;XP_	-2.0
GATAD2B	GATA zinc finger domain containing 2B	protein_coding	NP_065750.1;XP_005245421.1;XP_	-2.0
BSDC1	BSD domain containing 1	protein_coding	NP_001137360.1;NP_001137361.1;	-2.0
PRKACB	protein kinase cAMP-activated catalytic subunit b	protein_coding	NP_001229786.1;NP_001229787.1;	-2.0
CRTC3	CREB regulated transcription coactivator 3	protein_coding	NP_001036039.1;NP_073606.3;XP_	-2.0
DHFRP1	dihydrofolate reductase pseudogene 1	pseudogene	.	-2.0

SNORA73B	small nucleolar RNA, H/ACA box 73B	snoRNA	.	-2.0
SMPDL3B	sphingomyelin phosphodiesterase acid like 3B	protein_coding	NP_001009568.1;NP_001291508.1;	-2.0
ARL16	ADP ribosylation factor like GTPase 16	protein_coding	NP_001035114.1;NP_001316537.1;	-2.0
LGALSL	galectin like	protein_coding	NP_054900.2	-2.0
LINC02709	long intergenic non-protein coding RNA 2709	lncRNA	.	-2.0
HLA-DPB1	major histocompatibility complex, class II, DP bet	protein_coding	NP_002112.3	-2.0
TMEM42	transmembrane protein 42	protein_coding	NP_653239.1	-2.0
DAP	death associated protein	protein_coding	NP_001278892.1;NP_004385.1	-2.0
SPRED3	sprouty related EVH1 domain containing 3	protein_coding	NP_001035987.1;XP_006723282.1;	-2.0
SEPHS1	selenophosphate synthetase 1	protein_coding	NP_001182531.1;NP_001182533.1;	-2.0
C4orf3	chromosome 4 open reading frame 3	protein_coding	NP_001001701.2;NP_001163801.1	-2.0
CIAO2B	cytosolic iron-sulfur assembly component 2B	protein_coding	NP_057146.1	-2.0
NAA40	N-alpha-acetyltransferase 40, NatD catalytic subu	protein_coding	NP_001287729.1;NP_079047.2;XP_	-2.0
SDHA	succinate dehydrogenase complex flavoprotein sul	protein_coding	NP_001281261.1;NP_001317687.1;	-2.0
SGSM2	small G protein signaling modulator 2	protein_coding	NP_001091979.1;NP_001333629.1;	-2.0
MAGEA12	MAGE family member A12	protein_coding	NP_001159858.1;NP_001159859.1;	-2.0
RDH10	retinol dehydrogenase 10	protein_coding	NP_742034.1	-2.0
ENTR1	endosome associated trafficking regulator 1	protein_coding	NP_001034796.1;NP_001034797.1;	-2.0
RASSF1	Ras association domain family member 1	protein_coding	NP_001193886.1;NP_009113.3;NP_	-2.0
LCMT2	leucine carboxyl methyltransferase 2	protein_coding	NP_055608.2	-2.0
C12orf57	chromosome 12 open reading frame 57	protein_coding	NP_001288763.1;NP_001288765.1;	-2.0
ZNF185	zinc finger protein 185 with LIM domain	protein_coding	NP_001171577.1;NP_001171578.1;	-2.0
CCDC9B	coiled-coil domain containing 9B	protein_coding	NP_997263.2	-2.0
DIXDC1	DIX domain containing 1	protein_coding	NP_001033043.1;NP_001265471.1;	-2.0
FAM89A	family with sequence similarity 89 member A	protein_coding	NP_940954.1	-2.0
OIP5	Opa interacting protein 5	protein_coding	NP_001304789.1;NP_009211.1	-2.0
ZSCAN22	zinc finger and SCAN domain containing 22	protein_coding	NP_001308045.1;NP_001308046.1;	-2.0
NELFE	negative elongation factor complex member E	protein_coding	NP_002895.3;XP_006715268.1;XP_	-2.0
GLB1L	galactosidase beta 1 like	protein_coding	NP_001273352.1;NP_001273356.1;	-2.0
CAMKK2	calcium/calmodulin dependent protein kinase kina	protein_coding	NP_001257414.1;NP_001257415.1;	-2.0
TTC30A	tetratricopeptide repeat domain 30A	protein_coding	NP_689488.3	-2.0
TMPO-AS1	TMPO antisense RNA 1	lncRNA	.	-2.0
TMEM220	transmembrane protein 220	protein_coding	NP_001004313.1;NP_001317068.1;	-2.0
GNL1	G protein nucleolar 1 (putative)	protein_coding	NP_005266.2;XP_005249072.1	-2.0
AK4	adenylate kinase 4	protein_coding	NP_001005353.1;NP_001317545.1;	-2.0
PXN-AS1	PXN antisense RNA 1	lncRNA	.	-2.0
HAX1	HCLS1 associated protein X-1	protein_coding	NP_001018238.1;NP_006109.2	-2.0
RMDN3	regulator of microtubule dynamics 3	protein_coding	NP_001291731.1;NP_001310823.1;	-2.0
FILIP1	filamin A interacting protein 1	protein_coding	NP_001276916.1;NP_001287795.1;	-2.0
PRKAR2A	protein kinase cAMP-dependent type II regulatory	protein_coding	NP_001308911.1;NP_001308912.1;	-2.0
ARL4C	ADP ribosylation factor like GTPase 4C	protein_coding	NP_001269360.1;NP_005728.2	-2.0
PSPH	phosphoserine phosphatase	protein_coding	NP_001357432.1;NP_001357433.1;	-2.0
HIP1	huntingtin interacting protein 1	protein_coding	NP_001230127.1;NP_001369373.1;	-2.1
TSR2	TSR2 ribosome maturation factor	protein_coding	NP_001333718.1;NP_001333719.1;	-2.1
CRAMP1	cramped chromatin regulator homolog 1	protein_coding	NP_065876.3	-2.1
FNTB	farnesyltransferase, CAAX box, beta	protein_coding	NP_002019.1	-2.1
F2RL2	coagulation factor II thrombin receptor like 2	protein_coding	NP_001243495.1;NP_004092.1	-2.1
MDFIC	MyoD family inhibitor domain containing	protein_coding	NP_001159817.1;NP_001159818.3;	-2.1
TOMM22	translocase of outer mitochondrial membrane 22	protein_coding	NP_064628.1	-2.1
SGSH	N-sulfoglucosamine sulfohydrolase	protein_coding	NP_000190.1;NP_001339850.1;NP_	-2.1
IRS2	insulin receptor substrate 2	protein_coding	NP_003740.2	-2.1
PPCS	phosphopantothenoylcysteine synthetase	protein_coding	NP_001070915.1;NP_001274435.1;	-2.1
HMGN2	high mobility group nucleosomal binding domain	protein_coding	NP_005508.1	-2.1
LOC105373077	uncharacterized LOC105373077	lncRNA	.	-2.1
CCDC92	coiled-coil domain containing 92	protein_coding	NP_001291886.1;NP_001291887.1;	-2.1
ZNF491	zinc finger protein 491	protein_coding	NP_689569.2;XP_005259787.1	-2.1
H3P6	H3 histone pseudogene 6	ranscribed_pseudogen	.	-2.1
EFTUD2	elongation factor Tu GTP binding domain contain	protein_coding	NP_001136077.1;NP_001245282.1;	-2.1
RNF11	ring finger protein 11	protein_coding	NP_055187.1	-2.1
HIRIP3	HIRA interacting protein 3	protein_coding	NP_001184252.1;NP_003600.2	-2.1
LRRC57	leucine rich repeat containing 57	protein_coding	NP_694992.2;XP_011519725.1;XP_	-2.1
TNFAIP1	TNF alpha induced protein 1	protein_coding	NP_066960.1;XP_016880482.1	-2.1
LINC02021	long intergenic non-protein coding RNA 2021	lncRNA	.	-2.1
KDM2A	lysine demethylase 2A	protein_coding	NP_001243334.1;NP_036440.1;XP_	-2.1
TM7SF3	transmembrane 7 superfamily member 3	protein_coding	NP_057635.1;XP_005253448.1;XP_	-2.1
UBE2D2	ubiquitin conjugating enzyme E2 D2	protein_coding	NP_003330.1;NP_862821.1;XP_01	-2.1
ODC1	ornithine decarboxylase 1	protein_coding	NP_001274117.1;NP_001274118.1;	-2.1
ELOVL1	ELOVL fatty acid elongase 1	protein_coding	NP_001243328.1;NP_001243330.1;	-2.1
RAB4A	RAB4A, member RAS oncogene family	protein_coding	NP_001258927.1;NP_004569.2	-2.1
ABTB2	ankyrin repeat and BTB domain containing 2	protein_coding	NP_665803.2	-2.1
LAMTOR4	late endosomal/lysosomal adaptor, MAPK and M	protein_coding	NP_001008396.1;NP_001305165.1;	-2.1

HINT3	histidine triad nucleotide binding protein 3	protein_coding	NP_612638.3	-2.1
SNRNP25	small nuclear ribonucleoprotein U11/U12 subunit	protein_coding	NP_078847.2	-2.1
GALNT4	polypeptide N-acetylgalactosaminyltransferase 4	protein_coding	NP_003765.2	-2.1
KIF9-AS1	KIF9 antisense RNA 1	lncRNA	.	-2.1
EIF4EBP2	eukaryotic translation initiation factor 4E binding p	protein_coding	NP_004087.1	-2.1
CHP1	calcineurin like EF-hand protein 1	protein_coding	NP_009167.1;XP_016877368.1	-2.1
UBE2C	ubiquitin conjugating enzyme E2 C	protein_coding	NP_001268670.1;NP_001268671.1;	-2.1
GYPC	glycophorin C (Gerbich blood group)	protein_coding	NP_001243513.1;NP_002092.1;NP_	-2.1
VPS26B	VPS26, retromer complex component B	protein_coding	NP_443107.1;XP_011540867.1	-2.1
CCDC117	coiled-coil domain containing 117	protein_coding	NP_001271192.1;NP_001271193.1;	-2.1
BYSL	bystin like	protein_coding	NP_004044.3	-2.1
CMTM4	CKLF like MARVEL transmembrane domain con	protein_coding	NP_848933.1;NP_852662.1;XP_01	-2.1
TNS3	tensin 3	protein_coding	NP_073585.8;XP_011513778.1;XP_	-2.1
ALDH2	aldehyde dehydrogenase 2 family member	protein_coding	NP_000681.2;NP_001191818.1	-2.1
GCSH	glycine cleavage system protein H	protein_coding	NP_004474.2;XP_016878625.1;XP_	-2.1
POMZP3	POM121 and ZP3 fusion	protein_coding	NP_036362.3;NP_694537.1	-2.1
PEX26	peroxisomal biogenesis factor 26	protein_coding	NP_001121121.1;NP_001186248.1;	-2.1
DESI1	desumoylating isopeptidase 1	protein_coding	NP_056519.1;XP_005261628.1	-2.1
MPRIP	myosin phosphatase Rho interacting protein	protein_coding	NP_001351645.2;NP_055949.2;NP_	-2.1
MYLIP	myosin regulatory light chain interacting protein	protein_coding	NP_037394.2;XP_005249089.1;XP_	-2.1
ZKSCAN3	zinc finger with KRAB and SCAN domains 3	protein_coding	NP_001229823.1;NP_001229824.1;	-2.1
MBOAT1	membrane bound O-acyltransferase domain contai	protein_coding	NP_001073949.1;XP_006715062.1;	-2.1
NCOA5	nuclear receptor coactivator 5	protein_coding	NP_001335077.1;NP_001335078.1;	-2.1
MIF-AS1	MIF antisense RNA 1	lncRNA	.	-2.1
ITGA5	integrin subunit alpha 5	protein_coding	NP_002196.4;XP_024304738.1	-2.1
PPP2CB	protein phosphatase 2 catalytic subunit beta	protein_coding	NP_001009552.1	-2.1
ZFYVE28	zinc finger FYVE-type containing 28	protein_coding	NP_001166127.1;NP_001166128.1;	-2.1
AKIRIN1	akirin 1	protein_coding	NP_001129747.1;NP_078871.1	-2.1
MRPS6	mitochondrial ribosomal protein S6	protein_coding	NP_115865.1	-2.1
MGAT2	alpha-1,6-mannosyl-glycoprotein 2-beta-N-acetylg	protein_coding	NP_002399.1	-2.1
RREB1	ras responsive element binding protein 1	protein_coding	NP_001003698.1;NP_001003699.1;	-2.1
CDC42BPB	CDC42 binding protein kinase beta	protein_coding	NP_006026.3;XP_005268284.1;XP_	-2.1
EIF4E3	eukaryotic translation initiation factor 4E family m	protein_coding	NP_001128121.1;NP_001128122.1;	-2.1
CASP9	caspase 9	protein_coding	NP_001220.2;NP_001264983.1;NP_	-2.1
NREP	neuronal regeneration related protein	protein_coding	NP_001135946.1;NP_001135947.1;	-2.1
POU4F1	POU class 4 homeobox 1	protein_coding	NP_006228.3	-2.1
SRSF2	serine and arginine rich splicing factor 2	protein_coding	NP_001182356.1;NP_003007.2;XP_	-2.1
MAGEA1	MAGE family member A1	protein_coding	NP_004979.3	-2.1
COX16	cytochrome c oxidase assembly factor COX16	protein_coding	NP_001191019.1;NP_057552.1	-2.1
BET1L	Bet1 golgi vesicular membrane trafficking protein	protein_coding	NP_001092257.1;NP_057610.2	-2.1
SLC3A2	solute carrier family 3 member 2	protein_coding	NP_001012680.1;NP_001012682.1;	-2.1
CEP164	centrosomal protein 164	protein_coding	NP_001258862.1;NP_055771.4;XP_	-2.1
PIK3R3	phosphoinositide-3-kinase regulatory subunit 3	protein_coding	NP_001107644.1;NP_001290357.1;	-2.1
PEBP1	phosphatidylethanolamine binding protein 1	protein_coding	NP_002558.1	-2.1
FAM50A	family with sequence similarity 50 member A	protein_coding	NP_004690.1	-2.1
CYP27C1	cytochrome P450 family 27 subfamily C member	protein_coding	NP_001001665.3;NP_001354430.1;	-2.1
C12orf75	chromosome 12 open reading frame 75	protein_coding	NP_001138671.1	-2.1
PERP	p53 apoptosis effector related to PMP22	protein_coding	NP_071404.2;XP_024302288.1	-2.1
ARHGAP44	Rho GTPase activating protein 44	protein_coding	NP_001308093.1;NP_001308095.1;	-2.1
SLC7A2	solute carrier family 7 member 2	protein_coding	NP_001008539.3;NP_001158243.1;	-2.1
DHX38	DEAH-box helicase 38	protein_coding	NP_054722.2;XP_005256326.1;XP_	-2.1
ANKS3	ankyrin repeat and sterile alpha motif domain cont	protein_coding	NP_001229858.1;NP_001295018.1;	-2.1
PM20D2	peptidase M20 domain containing 2	protein_coding	NP_001010853.1;XP_005248718.1;	-2.1
TMEM214	transmembrane protein 214	protein_coding	NP_001077059.1;NP_060197.4;XP_	-2.1
AKIRIN2	akirin 2	protein_coding	NP_060534.1	-2.1
PROCR	protein C receptor	protein_coding	NP_006395.2;XP_011526798.1	-2.1
AR	androgen receptor	protein_coding	NP_000035.2;NP_001011645.1;NP_	-2.1
PHC1	polyhomeotic homolog 1	protein_coding	NP_004417.2;XP_005253391.1;XP_	-2.1
RPS5	ribosomal protein S5	protein_coding	NP_001000.2	-2.1
DDX56	DEAD-box helicase 56	protein_coding	NP_001244118.1;NP_061955.1	-2.1
APOBEC3G	apolipoprotein B mRNA editing enzyme catalytic	protein_coding	NP_001336365.1;NP_001336366.1;	-2.1
PHC2	polyhomeotic homolog 2	protein_coding	NP_001317417.1;NP_004418.2;NP_	-2.1
TMPO	thymopoietin	protein_coding	NP_001027454.1;NP_001027455.1;	-2.1
BOLA3	bolA family member 3	protein_coding	NP_001030582.1;NP_997717.2	-2.1
MCIDAS	multiciliate differentiation and DNA synthesis ass	protein_coding	NP_001177716.1;XP_016864928.1	-2.1
LOC101929054	uncharacterized LOC101929054	lncRNA	.	-2.1
DNMT3B	DNA methyltransferase 3 beta	protein_coding	NP_001193984.1;NP_001193985.1;	-2.1
PTPN1	protein tyrosine phosphatase non-receptor type 1	protein_coding	NP_001265547.1;NP_002818.1	-2.1
SH3BP4	SH3 domain binding protein 4	protein_coding	NP_001358231.1;NP_001358232.1;	-2.1
HMCES	5-hydroxymethylcytosine binding, ES cell specific	protein_coding	NP_001006109.1;NP_001350810.1;	-2.1
ACACB	acetyl-CoA carboxylase beta	protein_coding	NP_001084.3;XP_006719430.1;XP_	-2.1

LOC729086	VOPP1, WBP1/VOPP1 family member pseudoge	pseudogene	.	-2.1
RANBP10	RAN binding protein 10	protein_coding	NP_001307167.1;NP_001307168.1;	-2.1
EIF3F	eukaryotic translation initiation factor 3 subunit F	protein_coding	NP_003745.1	-2.1
GGT8P	gamma-glutamyltransferase 8 pseudogene	ranscribed_pseudogen	.	-2.1
HACD1	3-hydroxyacyl-CoA dehydratase 1	protein_coding	NP_055056.3;XP_005252698.1	-2.1
STK32C	serine/threonine kinase 32C	protein_coding	NP_001305807.1;NP_001305808.1;	-2.1
ATP5MC1	ATP synthase membrane subunit c locus 1	protein_coding	NP_001002027.1;NP_005166.1	-2.1
TSPAN13	tetraspanin 13	protein_coding	NP_055214.1	-2.1
CYSLTR1	cysteinyl leukotriene receptor 1	protein_coding	NP_001269115.1;NP_001269116.1;	-2.1
NOP14-AS1	NOP14 antisense RNA 1	lncRNA	.	-2.1
GALT	galactose-1-phosphate uridylyltransferase	protein_coding	NP_000146.2;NP_001245261.1	-2.1
PSMF1	proteasome inhibitor subunit 1	protein_coding	NP_001310336.1;NP_001310337.1;	-2.1
FBXO10	F-box protein 10	protein_coding	NP_036298.2;XP_005251496.1;XP_	-2.1
QSOX2	quiescin sulfhydryl oxidase 2	protein_coding	NP_859052.3	-2.1
MIEF1	mitochondrial elongation factor 1	protein_coding	NP_001291493.1;NP_061881.2;XP_	-2.1
PANK2-AS1	PANK2 antisense RNA 1	lncRNA	.	-2.1
EML2	EMAP like 2	protein_coding	NP_001180197.1;NP_001180198.1;	-2.1
PHC1P1	polyhomeotic homolog 1 pseudogene 1	pseudogene	.	-2.1
SLC25A19	solute carrier family 25 member 19	protein_coding	NP_001119593.1;NP_001119594.1;	-2.1
F8	coagulation factor VIII	protein_coding	NP_000123.1;NP_063916.1	-2.1
KIRREL3	kirre like nephrin family adhesion molecule 3	protein_coding	NP_001155179.1;NP_001288026.1;	-2.1
ADAM19	ADAM metallopeptidase domain 19	protein_coding	NP_150377.1;XP_016865498.1	-2.1
FAU	FAU ubiquitin like and ribosomal protein S30 fusi	protein_coding	NP_001988.1	-2.1
BMP5	bone morphogenetic protein 5	protein_coding	NP_001316683.1;NP_001316685.1;	-2.1
IFT140	intraflagellar transport 140	protein_coding	NP_055529.2;XP_005255782.1;XP_	-2.1
PARP3	poly(ADP-ribose) polymerase family member 3	protein_coding	NP_001003931.3;NP_001357168.1;	-2.1
KIF21B	kinesin family member 21B	protein_coding	NP_001239029.1;NP_001239031.1;	-2.1
PIGBOS1	PIGB opposite strand 1	protein_coding	NP_001295350.1;NP_001295351.1;	-2.1
RMI2	RecQ mediated genome instability 2	protein_coding	NP_689521.1	-2.1
CIB1	calcium and integrin binding 1	protein_coding	NP_001264693.1;NP_006375.2;XP_	-2.1
ATP6V1E2	ATPase H+ transporting V1 subunit E2	protein_coding	NP_001304992.1;NP_001358210.1;	-2.1
FCRLB	Fc receptor like B	protein_coding	NP_001002901.1;NP_001275758.1;	-2.1
DCAF7	DDB1 and CUL4 associated factor 7	protein_coding	NP_005819.3	-2.1
PAF1	PAF1 homolog, Paf1/RNA polymerase II complex	protein_coding	NP_001243755.1;NP_061961.2	-2.1
INAFM2	InaF motif containing 2	protein_coding	NP_001288197.1;XP_011519451.1	-2.1
MMGT1	membrane magnesium transporter 1	protein_coding	NP_001316929.1;NP_775741.1	-2.1
PTPRQ	protein tyrosine phosphatase receptor type Q	protein_coding	NP_001138498.1;XP_016874762.1;	-2.1
RPL7L1	ribosomal protein L7 like 1	protein_coding	NP_001353410.1;NP_940888.3	-2.1
TMEM64	transmembrane protein 64	protein_coding	NP_001008495.2;NP_001139745.1	-2.1
LMAN2L	lectin, mannose binding 2 like	protein_coding	NP_001135764.1;NP_001309275.1;	-2.1
ACVR1B	activin A receptor type 1B	protein_coding	NP_004293.1;NP_064732.3;NP_06	-2.1
LINC01615	long intergenic non-protein coding RNA 1615	lncRNA	.	-2.1
PCDH7	protocadherin 7	protein_coding	NP_001166994.1;NP_002580.2;NP_	-2.1
TMEM171	transmembrane protein 171	protein_coding	NP_001154814.1;NP_775761.4;XP_	-2.1
HM13	histocompatibility minor 13	protein_coding	NP_110416.1;NP_848695.1;NP_84	-2.1
FDX1	ferredoxin 1	protein_coding	NP_004100.1	-2.1
S100A16	S100 calcium binding protein A16	protein_coding	NP_001303936.1;NP_001303937.1;	-2.1
PDPK1	3-phosphoinositide dependent protein kinase 1	protein_coding	NP_001248745.1;NP_002604.1;NP_	-2.1
TOP1MT	DNA topoisomerase I mitochondrial	protein_coding	NP_001245375.1;NP_001245376.1;	-2.2
KCNK6	potassium two pore domain channel subfamily K r	protein_coding	NP_004814.1;XP_011525829.1;XP_	-2.2
ANPEP	alanyl aminopeptidase, membrane	protein_coding	NP_001141.2;NP_001368852.1;NP_	-2.2
C17orf58	chromosome 17 open reading frame 58	protein_coding	NP_001369288.1;NP_858041.2;NP_	-2.2
WIPF2	WAS/WASL interacting protein family member 2	protein_coding	NP_573571.1;XP_005257140.1;XP_	-2.2
PRKCD	protein kinase C delta	protein_coding	NP_001303256.1;NP_001341605.1;	-2.2
SLC43A1	solute carrier family 43 member 1	protein_coding	NP_001185739.1;NP_003618.1;XP_	-2.2
FAH	fumarylacetoacetate hydrolase	protein_coding	NP_000128.1;NP_001361306.1;NP_	-2.2
LOC112268437	uncharacterized protein FLJ45252	protein_coding	XP_024309047.1	-2.2
GINS1	GINS complex subunit 1	protein_coding	NP_066545.3;XP_016883651.1	-2.2
KCTD10	potassium channel tetramerization domain contain	protein_coding	NP_001304324.1;NP_001304328.1;	-2.2
LOC112268190	uncharacterized LOC112268190	lncRNA	.	-2.2
INKA2	inka box actin regulator 2	protein_coding	NP_061972.1;NP_945120.1;XP_01	-2.2
ZDHHC9	zinc finger DHHC-type palmitoyltransferase 9	protein_coding	NP_001008223.1;NP_057116.2;XP_	-2.2
POLR1A	RNA polymerase I subunit A	protein_coding	NP_056240.2	-2.2
NGRN	neugrin, neurite outgrowth associated	protein_coding	NP_001028260.2	-2.2
TBC1D25	TBC1 domain family member 25	protein_coding	NP_001335191.1;NP_001335192.1;	-2.2
VCP	valosin containing protein	protein_coding	NP_001341856.1;NP_001341857.1;	-2.2
SMAP2	small ArfGAP2	protein_coding	NP_001185907.1;NP_001185908.1;	-2.2
DNMT1	DNA methyltransferase 1	protein_coding	NP_001124295.1;NP_001305659.1;	-2.2
DCST1-AS1	DCST1 antisense RNA 1	lncRNA	.	-2.2
HACD2	3-hydroxyacyl-CoA dehydratase 2	protein_coding	NP_001316712.1;NP_001316713.1;	-2.2
TAFA2	TAFA chemokine like family member 2	protein_coding	NP_848634.1;XP_011536574.1;XP_	-2.2

MAP3K12	mitogen-activated protein kinase kinase kinase 12	protein_coding	NP_001180440.1;NP_006292.3;XP_	-2.2
TYMS	thymidylate synthetase	protein_coding	NP_001062.1;NP_001341796.1;NP_	-2.2
CREBBP	CREB binding protein	protein_coding	NP_001073315.1;NP_004371.2;XP_	-2.2
TAF10	TATA-box binding protein associated factor 10	protein_coding	NP_006275.1	-2.2
RRP7BP	ribosomal RNA processing 7 homolog B, pseudog	ranscribed_pseudogen	.	-2.2
TMEM39B	transmembrane protein 39B	protein_coding	NP_001306606.1;NP_001306607.1;	-2.2
GSR	glutathione-disulfide reductase	protein_coding	NP_000628.2;NP_001182031.1;NP_	-2.2
SPRING1	SREBF pathway regulator in golgi 1	protein_coding	NP_001340552.1;NP_001340553.1;	-2.2
LOC100129534	small nuclear ribonucleoprotein polypeptide N pse	ranscribed_pseudogen	.	-2.2
SPOCD1	SPOC domain containing 1	protein_coding	NP_001268916.1;NP_001268917.1;	-2.2
LDLR	low density lipoprotein receptor	protein_coding	NP_000518.1;NP_001182727.1;NP_	-2.2
LSM2	LSM2 homolog, U6 small nuclear RNA and mRN	protein_coding	NP_067000.1	-2.2
GINM1	glycoprotein integral membrane 1	protein_coding	NP_620140.1	-2.2
PEA15	proliferation and apoptosis adaptor protein 15	protein_coding	NP_001284505.1;NP_001284506.1;	-2.2
LOC100996437	uncharacterized LOC100996437	lncRNA	.	-2.2
PHB	prohibitin	protein_coding	NP_001268425.1;NP_001268426.1;	-2.2
TMEM150A	transmembrane protein 150A	protein_coding	NP_001026908.1;NP_001356846.1;	-2.2
ZDHHC7	zinc finger DHHC-type palmitoyltransferase 7	protein_coding	NP_001139020.1;NP_060210.2;XP_	-2.2
PCDHB13	protocadherin beta 13	protein_coding	NP_061756.1	-2.2
SNHG7	small nucleolar RNA host gene 7	lncRNA	.	-2.2
KLHL36	kelch like family member 36	protein_coding	NP_001290380.1;NP_079007.2;XP_	-2.2
DSTNP2	DSTN pseudogene 2	ranscribed_pseudogen	.	-2.2
PIGO	phosphatidylinositol glycan anchor biosynthesis cl	protein_coding	NP_001188413.1;NP_116023.2;NP_	-2.2
MMP2	matrix metallopeptidase 2	protein_coding	NP_001121363.1;NP_001289437.1;	-2.2
TUBA1B	tubulin alpha 1b	protein_coding	NP_006073.2	-2.2
DDIT4	DNA damage inducible transcript 4	protein_coding	NP_061931.1	-2.2
RNF135	ring finger protein 135	protein_coding	NP_001171921.1;NP_115698.3;NP_	-2.2
HASPIN	histone H3 associated protein kinase	protein_coding	NP_114171.2	-2.2
SMARCC2	SWI/SNF related, matrix associated, actin depend	protein_coding	NP_001123892.1;NP_001317217.1;	-2.2
GSS	glutathione synthetase	protein_coding	NP_000169.1;NP_001309423.1;NP_	-2.2
RHPN2	rhophilin Rho GTPase binding protein 2	protein_coding	NP_149094.3	-2.2
MPC2	mitochondrial pyruvate carrier 2	protein_coding	NP_001137146.1;NP_056230.1;XP_	-2.2
SPATA18	spermatogenesis associated 18	protein_coding	NP_001284537.1;NP_001333031.1;	-2.2
PBK	PDZ binding kinase	protein_coding	NP_001265874.1;NP_001349969.1;	-2.2
ATPAF1	ATP synthase mitochondrial F1 complex assembly	protein_coding	NP_001036011.2;NP_001230657.1;	-2.2
RRAGD	Ras related GTP binding D	protein_coding	NP_067067.1;XP_005248812.1;XP_	-2.2
COMMD7	COMM domain containing 7	protein_coding	NP_001092809.1;NP_444269.2;XP_	-2.2
RPS15	ribosomal protein S15	protein_coding	NP_001009.1;NP_001295155.1	-2.2
VRK3	VRK serine/threonine kinase 3	protein_coding	NP_001020949.1;NP_001295349.1;	-2.2
TBC1D14	TBC1 domain family member 14	protein_coding	NP_001106832.1;NP_001106834.1;	-2.2
DEGS1	delta 4-desaturase, sphingolipid 1	protein_coding	NP_001308470.1;NP_001308471.1;	-2.2
ZSWIM1	zinc finger SWIM-type containing 1	protein_coding	NP_542170.3;XP_005260667.1;XP_	-2.2
TIMM22	translocase of inner mitochondrial membrane 22	protein_coding	NP_037469.2	-2.2
WDR91	WD repeat domain 91	protein_coding	NP_001349665.1;NP_001349666.1;	-2.2
DDB2	damage specific DNA binding protein 2	protein_coding	NP_000098.1;NP_001287663.1	-2.2
LOC100129034	uncharacterized LOC100129034	lncRNA	.	-2.2
AGAP1	ArfGAP with GTPase domain, ankyrin repeat and	protein_coding	NP_001032208.1;NP_001231817.1;	-2.2
LINC00963	long intergenic non-protein coding RNA 963	lncRNA	.	-2.2
H2BC4	H2B clustered histone 4	protein_coding	NP_001368918.1;NP_003517.2	-2.2
C1orf198	chromosome 1 open reading frame 198	protein_coding	NP_001129966.1;NP_001129967.1;	-2.2
WWC1	WW and C2 domain containing 1	protein_coding	NP_001155133.1;NP_001155134.1;	-2.2
ABCF3	ATP binding cassette subfamily F member 3	protein_coding	NP_001338227.1;NP_001338228.1;	-2.2
AKAP5	A-kinase anchoring protein 5	protein_coding	NP_004848.3	-2.2
FAM168A	family with sequence similarity 168 member A	protein_coding	NP_001272979.1;NP_001272980.1;	-2.2
NISCH	nischarin	protein_coding	NP_001263222.2;NP_001263223.2;	-2.2
EZR	ezrin	protein_coding	NP_001104547.1;NP_003370.2;XP_	-2.2
SKA1	spindle and kinetochore associated complex subun	protein_coding	NP_001034624.1;NP_659497.1	-2.2
TTC7B	tetratricopeptide repeat domain 7B	protein_coding	NP_001010854.1;NP_001307350.1;	-2.2
CSF1	colony stimulating factor 1	protein_coding	NP_000748.4;NP_757349.2;NP_75	-2.2
LIPH	lipase H	protein_coding	NP_640341.1;XP_006713592.1;XP_	-2.2
HOXB6	homeobox B6	protein_coding	NP_001356326.1;NP_061825.2;XP_	-2.2
PSD4	pleckstrin and Sec7 domain containing 4	protein_coding	NP_036587.2;XP_005263691.1;XP_	-2.2
WIPI2	WD repeat domain, phosphoinositide interacting 2	protein_coding	NP_001028690.1;NP_001028691.1;	-2.2
SNHG11	small nucleolar RNA host gene 11	lncRNA	.	-2.2
PAXIP1-AS1	PAXIP1 antisense RNA 1 (head to head)	lncRNA	.	-2.2
SUMO2P17	SUMO2 pseudogene 17	pseudogene	.	-2.2
ZCCHC14	zinc finger CCHC-type containing 14	protein_coding	NP_055959.2;XP_005255915.2;XP_	-2.2
TMED9	transmembrane p24 trafficking protein 9	protein_coding	NP_059980.2	-2.2
ALDH6A1	aldehyde dehydrogenase 6 family member A1	protein_coding	NP_001265522.1;NP_001265523.1;	-2.2
LARP1	La ribonucleoprotein 1, translational regulator	protein_coding	NP_001354642.1;NP_001354643.1;	-2.2
MRPL30	mitochondrial ribosomal protein L30	protein_coding	NP_660213.1	-2.2

CYB5D2	cytochrome b5 domain containing 2	protein_coding	NP_001241684.1;NP_001241685.1;	-2.2
TMEM71	transmembrane protein 71	protein_coding	NP_001138625.1;NP_001351814.1;	-2.2
CENPA	centromere protein A	protein_coding	NP_001035891.1;NP_001800.1	-2.2
RAB30	RAB30, member RAS oncogene family	protein_coding	NP_001272988.1;NP_001272989.1;	-2.2
OLR1	oxidized low density lipoprotein receptor 1	protein_coding	NP_001166103.1;NP_001166104.1;	-2.2
NPHP4	nephrocystin 4	protein_coding	NP_001278522.1;NP_001278523.1;	-2.2
DDX41	DEAD-box helicase 41	protein_coding	NP_001308661.1;NP_001308759.1;	-2.2
MEGF9	multiple EGF like domains 9	protein_coding	NP_001073966.2	-2.2
RPL13A	ribosomal protein L13a	protein_coding	NP_001257420.1;NP_036555.1	-2.2
DSG3	desmoglein 3	protein_coding	NP_001935.2;XP_011524152.1	-2.2
PRR7	proline rich 7, synaptic	protein_coding	NP_001167572.1;NP_001167573.1;	-2.2
LMTK2	lemur tyrosine kinase 2	protein_coding	NP_055731.2;XP_011514283.1	-2.2
DNAJA3	DnaJ heat shock protein family (Hsp40) member A	protein_coding	NP_001128582.1;NP_001273445.1;	-2.2
PCBP2	poly(rC) binding protein 2	protein_coding	NP_001092090.1;NP_001122383.1;	-2.2
LOC105370809	uncharacterized LOC105370809	lncRNA	.	-2.2
CAVIN2	caveolae associated protein 2	protein_coding	NP_004648.1	-2.2
SNAI3-AS1	SNAI3 antisense RNA 1	lncRNA	.	-2.2
PLEKHH1	pleckstrin homology, MyTH4 and FERM domain	protein_coding	NP_065766.1;XP_011535311.1;XP_	-2.2
HNRNPD	heterogeneous nuclear ribonucleoprotein D	protein_coding	NP_001003810.1;NP_002129.2;NP_	-2.2
LDLRAD3	low density lipoprotein receptor class A domain co	protein_coding	NP_001291192.1;NP_001291193.1;	-2.2
LINC01137	long intergenic non-protein coding RNA 1137	lncRNA	.	-2.2
DUSP3	dual specificity phosphatase 3	protein_coding	NP_004081.1	-2.2
SSU72	SSU72 homolog, RNA polymerase II CTD phospl	protein_coding	NP_054907.1	-2.2
ADGRL3	adhesion G protein-coupled receptor L3	protein_coding	NP_001309175.1;NP_001309331.1;	-2.2
TGM2	transglutaminase 2	protein_coding	NP_001310245.1;NP_001310246.1;	-2.2
RTEL1	regulator of telomere elongation helicase 1	protein_coding	NP_001269938.1;NP_001269939.1;	-2.2
TCFL5	transcription factor like 5	protein_coding	NP_001288655.1;NP_006593.2;XP_	-2.2
CDS1	CDP-diacylglycerol synthase 1	protein_coding	NP_001254.2;XP_005262744.1;XP_	-2.2
MTMR14	myotubularin related protein 14	protein_coding	NP_001070993.1;NP_001070994.1;	-2.2
ITGB5	integrin subunit beta 5	protein_coding	NP_001341693.1;NP_001341694.1;	-2.2
KYAT1	kynurenine aminotransferase 1	protein_coding	NP_001116143.1;NP_001116144.1;	-2.3
BHLHE41	basic helix-loop-helix family member e41	protein_coding	NP_110389.1	-2.3
SAT2	spermidine/spermine N1-acetyltransferase family 1	protein_coding	NP_001307774.1;NP_001307775.1;	-2.3
NADSYN1	NAD synthetase 1	protein_coding	NP_060631.2	-2.3
DPYSL2	dihydropyrimidinase like 2	protein_coding	NP_001184222.1;NP_001231533.1;	-2.3
KIAA1191	KIAA1191	protein_coding	NP_001073152.1;NP_001073153.1;	-2.3
CHRFAM7A	CHRNA7 (exons 5-10) and FAM7A (exons A-E)	protein_coding	NP_647536.1;NP_683709.1;XP_00	-2.3
ALAD	aminolevulinate dehydratase	protein_coding	NP_000022.3;NP_001003945.1;NP_	-2.3
AMBRA1	autophagy and beclin 1 regulator 1	protein_coding	NP_001254711.1;NP_001254712.1;	-2.3
PARTICL	promoter of MAT2A antisense radiation-induced c	lncRNA	.	-2.3
SLC25A4	solute carrier family 25 member 4	protein_coding	NP_001142.2	-2.3
ACSF2	acyl-CoA synthetase family member 2	protein_coding	NP_001275897.1;NP_001275898.1;	-2.3
AURKB	aurora kinase B	protein_coding	NP_001243763.1;NP_001271455.1;	-2.3
PDCD2L	programmed cell death 2 like	protein_coding	NP_001340362.1;NP_115722.1	-2.3
AKAP8L	A-kinase anchoring protein 8 like	protein_coding	NP_001278407.1;NP_055186.3	-2.3
KAZN	kazrin, periplakin interacting protein	protein_coding	NP_001017999.1;NP_001018000.1;	-2.3
PGRMC2	progesterone receptor membrane component 2	protein_coding	NP_006311.3;XP_011529835.1	-2.3
SEMA4F	ssemaphorin 4F	protein_coding	NP_001258590.1;NP_001258591.1;	-2.3
SAMD4B	sterile alpha motif domain containing 4B	protein_coding	NP_001290543.1;NP_060498.2;XP_	-2.3
SF3B2	splicing factor 3b subunit 2	protein_coding	NP_006833.2;XP_005273783.1;XP_	-2.3
MPP7	membrane palmitoylated protein 7	protein_coding	NP_001305099.1;NP_775767.2;XP_	-2.3
SNORA94	small nucleolar RNA, H/ACA box 94	snoRNA	.	-2.3
SSTR1	somatostatin receptor 1	protein_coding	NP_001040.1	-2.3
PDE12	phosphodiesterase 12	protein_coding	NP_001309105.1;NP_001309106.1;	-2.3
TECPR2	tectonin beta-propeller repeat containing 2	protein_coding	NP_001166102.1;NP_055659.2	-2.3
MRPL11	mitochondrial ribosomal protein L11	protein_coding	NP_057134.1;NP_733934.1;NP_73	-2.3
DNAJC11	DnaJ heat shock protein family (Hsp40) member C	protein_coding	NP_060668.2;XP_016857234.1	-2.3
RELL1	RELT like 1	protein_coding	NP_001078868.1;NP_001078869.1;	-2.3
BAZ2A	bromodomain adjacent to zinc finger domain 2A	protein_coding	NP_001287834.1;NP_001338085.1;	-2.3
BCAP31	B cell receptor associated protein 31	protein_coding	NP_001132913.1;NP_001132929.1;	-2.3
PIF1	PIF1 5'-to-3' DNA helicase	protein_coding	NP_001273425.1;NP_001273426.1;	-2.3
H2BC5	H2B clustered histone 5	protein_coding	NP_066407.1;NP_619790.1;XP_00	-2.3
NXT1	nuclear transport factor 2 like export factor 1	protein_coding	NP_037380.1	-2.3
RPS7P1	ribosomal protein S7 pseudogene 1	pseudogene	.	-2.3
LITAF	lipopolysaccharide induced TNF factor	protein_coding	NP_001129944.1;NP_001129945.1;	-2.3
QDPR	quinoid dihydropteridine reductase	protein_coding	NP_000311.2;NP_001293069.1	-2.3
CLCN4	chloride voltage-gated channel 4	protein_coding	NP_001243873.1;NP_001821.2	-2.3
CGNL1	cingulin like 1	protein_coding	NP_001239264.1;NP_116255.2;XP_	-2.3
DENND2C	DENN domain containing 2C	protein_coding	NP_001243333.1;NP_940861.3	-2.3
OBSCN	obscurin, cytoskeletal calmodulin and titin-interac	protein_coding	NP_001092093.2;NP_001258152.2;	-2.3
ARL6IP1	ADP ribosylation factor like GTPase 6 interacting	protein_coding	NP_001300787.1;NP_055976.1	-2.3

DUSP4	dual specificity phosphatase 4	protein_coding	NP_001385.1;NP_476499.1;XP_01	-2.3
MAP4	microtubule associated protein 4	protein_coding	NP_001127836.1;NP_002366.2;NP_	-2.3
COPS7A	COP9 signalosome subunit 7A	protein_coding	NP_001157565.1;NP_001157566.1;	-2.3
STAT5B	signal transducer and activator of transcription 5B	protein_coding	NP_036580.2;XP_005257683.1;XP_	-2.3
USP21	ubiquitin specific peptidase 21	protein_coding	NP_001014443.1;NP_001306776.1;	-2.3
LOC653303	proprotein convertase subtilisin/kexin type 7 pseu	pseudogene	.	-2.3
HLA-A	major histocompatibility complex, class I, A	protein_coding	NP_002107.3	-2.3
POFUT1	protein O-fucosyltransferase 1	protein_coding	NP_056167.1;NP_758436.1	-2.3
EMP3	epithelial membrane protein 3	protein_coding	NP_001300834.1;NP_001416.1;XP_	-2.3
NFKB2	nuclear factor kappa B subunit 2	protein_coding	NP_001070962.1;NP_001248332.1;	-2.3
SQSTM1	sequestosome 1	protein_coding	NP_001135770.1;NP_001135771.1;	-2.3
JMJD8	jumonji domain containing 8	protein_coding	NP_001005920.3;NP_001310847.2;	-2.3
ELAC2	elaC ribonuclease Z 2	protein_coding	NP_001159434.1;NP_060597.4;NP_	-2.3
SLC2A11	solute carrier family 2 member 11	protein_coding	NP_001020109.1;NP_001020110.1;	-2.3
RUNDC1	RUN domain containing 1	protein_coding	NP_001308310.2;NP_775102.3;XP_	-2.3
PSG9	pregnancy specific beta-1-glycoprotein 9	protein_coding	NP_001288636.1;NP_001288637.1;	-2.3
HOMER3-AS1	HOMER3 antisense RNA 1	lncRNA	.	-2.3
ANKRD33B	ankyrin repeat domain 33B	protein_coding	NP_001157912.1;XP_005248400.1;	-2.3
CDK10	cyclin dependent kinase 10	protein_coding	NP_001092003.2;NP_001153839.1;	-2.3
H2BC11	H2B clustered histone 11	protein_coding	NP_066402.2	-2.3
POLR2J2	RNA polymerase II subunit J2	protein_coding	NP_116581.3	-2.3
TNFRSF11A	TNF receptor superfamily member 11a	protein_coding	NP_001257878.1;NP_001257879.1;	-2.3
GPR108	G protein-coupled receptor 108	protein_coding	NP_001073921.1;NP_064556.1;XP_	-2.3
SESN2	sestrin 2	protein_coding	NP_113647.1	-2.3
LOC105378179	uncharacterized LOC105378179, transcript varian	lncRNA	.	-2.3
2P1-UPK3BP1-PMS2	DTX2P1-UPK3BP1-PMS2P11 readthrough, trans	ranscribed_pseudogen	.	-2.3
ZNF335	zinc finger protein 335	protein_coding	NP_071378.1;XP_005260561.1;XP_	-2.3
MRTO4	MRT4 homolog, ribosome maturation factor	protein_coding	NP_057267.2;XP_006710738.1	-2.3
ZFYVE19	zinc finger FYVE-type containing 19	protein_coding	NP_001070736.1;NP_001245349.1;	-2.3
C16orf91	chromosome 16 open reading frame 91	protein_coding	NP_001258980.1	-2.3
UPP1	uridine phosphorylase 1	protein_coding	NP_001274355.1;NP_001274357.1;	-2.3
TRIM56	tripartite motif containing 56	protein_coding	NP_112223.1;XP_011514891.1	-2.3
PEX11B	peroxisomal biogenesis factor 11 beta	protein_coding	NP_001171724.1;NP_003837.1	-2.3
WDR1	WD repeat domain 1	protein_coding	NP_005103.2;NP_059830.1;XP_01	-2.3
DIPK2A	divergent protein kinase domain 2A	protein_coding	NP_001127942.1;NP_001350873.1;	-2.3
RPL7AP6	ribosomal protein L7a pseudogene 6	pseudogene	.	-2.3
TMEM185B	transmembrane protein 185B	protein_coding	NP_077026.2	-2.3
SNHG30	small nucleolar RNA host gene 30	lncRNA	.	-2.3
PPP1R11	protein phosphatase 1 regulatory inhibitor subunit	protein_coding	NP_068778.1;XP_006715237.1	-2.3
CENPH	centromere protein H	protein_coding	NP_075060.1	-2.3
GALNT6	polypeptide N-acetylgalactosaminyltransferase 6	protein_coding	NP_009141.2;XP_005268664.1;XP_	-2.3
BAIAP2L1	BAR/IMD domain containing adaptor protein 2 lik	protein_coding	NP_061330.2	-2.3
PABPN1	poly(A) binding protein nuclear 1	protein_coding	NP_001347480.1;NP_001347481.1;	-2.3
SEC14L2	SEC14 like lipid binding 2	protein_coding	NP_001191133.1;NP_001278861.1;	-2.3
SIX1	SIX homeobox 1	protein_coding	NP_005973.1;XP_016877091.1	-2.3
CLIC6	chloride intracellular channel 6	protein_coding	NP_001303938.1;NP_444507.1;XP_	-2.3
TMEM47	transmembrane protein 47	protein_coding	NP_113630.1	-2.3
ZBTB9	zinc finger and BTB domain containing 9	protein_coding	NP_689948.1	-2.3
FARP1	FERM, ARH/RhoGEF and pleckstrin domain prot	protein_coding	NP_001001715.2;NP_001273768.1;	-2.3
DERPC	DERPC proline and glycine rich nuclear protein	protein_coding	NP_001002847.1;NP_001035234.2;	-2.3
THSD1	thrombospondin type 1 domain containing 1	protein_coding	NP_061146.1;NP_954872.1	-2.3
PLEKHM1	pleckstrin homology and RUN domain containing	protein_coding	NP_001339754.1;NP_055613.1;XP_	-2.3
BLCAP	BLCAP apoptosis inducing factor	protein_coding	NP_001161292.1;NP_001161293.1;	-2.3
FAM83D	family with sequence similarity 83 member D	protein_coding	NP_112181.3;XP_016883577.1	-2.3
GGCX	gamma-glutamyl carboxylase	protein_coding	NP_000812.2;NP_001135741.1;NP_	-2.3
LOC100129434	uncharacterized LOC100129434	lncRNA	.	-2.3
SPPL3	signal peptide peptidase like 3	protein_coding	NP_620584.2;XP_011536227.1	-2.3
ANKRD23	ankyrin repeat domain 23	protein_coding	NP_659431.5	-2.3
CEP19	centrosomal protein 19	protein_coding	NP_001366397.1;NP_001366398.1;	-2.3
COPRS	coordinator of PRMT5 and differentiation stimula	protein_coding	NP_001317105.1;NP_060875.2	-2.3
MUTYH	mutY DNA glycosylase	protein_coding	NP_001041636.2;NP_001041637.1;	-2.3
ZNF212	zinc finger protein 212	protein_coding	NP_036388.2	-2.3
FTH1P2	ferritin heavy chain 1 pseudogene 2	pseudogene	.	-2.3
NUAK1	NUAK family kinase 1	protein_coding	NP_055655.1	-2.3
FTL	ferritin light chain	protein_coding	NP_000137.2;XP_024307215.1	-2.3
DHODH	dihydroorotate dehydrogenase (quinone)	protein_coding	NP_001352.2;XP_005255884.1;XP_	-2.3
LOC100506411	uncharacterized LOC100506411, transcript varian	lncRNA	.	-2.3
RNASET2	ribonuclease T2	protein_coding	NP_003721.2;XP_016866886.1;XP_	-2.3
CDCA8	cell division cycle associated 8	protein_coding	NP_001243804.1;NP_060571.1	-2.3
ISG15	ISG15 ubiquitin like modifier	protein_coding	NP_005092.1	-2.3
NR2F2	nuclear receptor subfamily 2 group F member 2	protein_coding	NP_001138627.1;NP_001138628.1;	-2.3

PDF	peptide deformylase, mitochondrial	protein_coding	NP_071736.1	-2.4
VIPR1	vasoactive intestinal peptide receptor 1	protein_coding	NP_001238811.1;NP_001238812.1;	-2.4
PTCH1	patched 1	protein_coding	NP_000255.2;NP_001077071.1;NP_	-2.4
PRSS53	serine protease 53	protein_coding	NP_001034592.1;XP_011544118.1;	-2.4
LINC00667	long intergenic non-protein coding RNA 667	lncRNA	.	-2.4
PIP4K2B	phosphatidylinositol-5-phosphate 4-kinase type 2 l	protein_coding	NP_003550.1;XP_011523628.1;XP_	-2.4
EPS15L1	epidermal growth factor receptor pathway substrat	protein_coding	NP_001245303.1;NP_001245304.1;	-2.4
CNTROB	centrobin, centriole duplication and spindle asseml	protein_coding	NP_001032221.1;NP_001317053.1;	-2.4
TMEM185A	transmembrane protein 185A	protein_coding	NP_001167563.1;NP_001269231.1;	-2.4
KLLN	killin, p53 regulated DNA replication inhibitor	protein_coding	NP_001119521.1	-2.4
APEX2	apurinic/apyrimidinic endodeoxyribonuclease 2	protein_coding	NP_001258677.1;NP_055296.2	-2.4
CHAF1A	chromatin assembly factor 1 subunit A	protein_coding	NP_005474.2;XP_011525907.1;XP_	-2.4
GLYCTK	glycerate kinase	protein_coding	NP_001138423.1;NP_660305.2;XP_	-2.4
ZNF101	zinc finger protein 101	protein_coding	NP_001287878.1;NP_149981.2;XP_	-2.4
MIEN1	migration and invasion enhancer 1	protein_coding	NP_001317135.1;NP_115715.3	-2.4
SF3B5	splicing factor 3b subunit 5	protein_coding	NP_112577.1	-2.4
PPP1R35-AS1	PPP1R35 antisense RNA 1, transcript variant X2	lncRNA	.	-2.4
HAUS2	HAUS augmin like complex subunit 2	protein_coding	NP_001123919.1;NP_001310558.1;	-2.4
STAT3	signal transducer and activator of transcription 3	protein_coding	NP_001356441.1;NP_001356442.1;	-2.4
GOLGA3	golgin A3	protein_coding	NP_001166028.1;NP_005886.2;XP_	-2.4
LOC107985944	uncharacterized LOC107985944	lncRNA	.	-2.4
FXYD5	FXYD domain containing ion transport regulator 5	protein_coding	NP_001158077.1;NP_001307841.1;	-2.4
ARMCX2	armadillo repeat containing X-linked 2	protein_coding	NP_001269160.1;NP_055597.1;NP_	-2.4
EEF1G	eukaryotic translation elongation factor 1 gamma	protein_coding	NP_001395.1	-2.4
IL17RA	interleukin 17 receptor A	protein_coding	NP_001276834.1;NP_055154.3	-2.4
C6orf136	chromosome 6 open reading frame 136	protein_coding	NP_001103408.1;NP_001154848.1;	-2.4
EBPL	EBP like	protein_coding	NP_001265565.1;NP_115954.1	-2.4
MEF2D	myocyte enhancer factor 2D	protein_coding	NP_001258558.1;NP_005911.1;XP_	-2.4
POC1A	POC1 centriolar protein A	protein_coding	NP_001155052.1;NP_001155053.1;	-2.4
PITPNC1	phosphatidylinositol transfer protein cytoplasmic 1	protein_coding	NP_036549.2;NP_858057.1;XP_00	-2.4
MECP2	methyl-CpG binding protein 2	protein_coding	NP_001104262.1;NP_001303266.1;	-2.4
FAM210B	family with sequence similarity 210 member B	protein_coding	NP_543011.2	-2.4
BICD2	BICD cargo adaptor 2	protein_coding	NP_001003800.1;NP_056065.1;XP_	-2.4
ENAH	ENAH actin regulator	protein_coding	NP_001008493.1;NP_001364410.1;	-2.4
CCDC51	coiled-coil domain containing 51	protein_coding	NP_001243893.1;NP_001243894.1;	-2.4
WDR4	WD repeat domain 4	protein_coding	NP_001247403.1;NP_001247404.1;	-2.4
MIR193BHG	MIR193B host gene	lncRNA	.	-2.4
SLC16A2	solute carrier family 16 member 2	protein_coding	NP_006508.2	-2.4
FAM86C2P	family with sequence similarity 86 member C2, ps	ranscribed_pseudogen	.	-2.4
DHRS2	dehydrogenase/reductase 2	protein_coding	NP_001305764.1;NP_005785.1;NP_	-2.4
ZNF689	zinc finger protein 689	protein_coding	NP_612456.1	-2.4
CCDC190	coiled-coil domain containing 190	protein_coding	NP_848645.3;XP_005245182.1;XP_	-2.4
VILL	villin like	protein_coding	NP_001357193.1;NP_001357194.1;	-2.4
LOC105372480	uncharacterized LOC105372480	lncRNA	.	-2.4
EMP2	epithelial membrane protein 2	protein_coding	NP_001415.1;XP_006720927.1	-2.4
LOC107985423	uncharacterized LOC107985423	lncRNA	.	-2.4
FAM222B	family with sequence similarity 222 member B	protein_coding	NP_001070966.1;NP_001275560.1;	-2.4
FAM193B	family with sequence similarity 193 member B	protein_coding	NP_001177875.1;NP_001353427.1;	-2.4
TXNL4A	thioredoxin like 4A	protein_coding	NP_001290400.1;NP_001292486.1;	-2.4
DNTTIP1	deoxynucleotidyltransferase terminal interacting p	protein_coding	NP_443183.1;XP_024307591.1	-2.4
PRXL2C	peroxiredoxin like 2C	protein_coding	NP_714542.1;XP_005251840.1;XP_	-2.4
KMT5A	lysine methyltransferase 5A	protein_coding	NP_001311433.1;NP_001311434.1;	-2.4
CCN1	cellular communication network factor 1	protein_coding	NP_001545.2	-2.4
CDH4	cadherin 4	protein_coding	NP_001239267.1;NP_001239268.1;	-2.4
PLAC8	placenta associated 8	protein_coding	NP_001124187.1;NP_001124188.1;	-2.4
GNG10	G protein subunit gamma 10	protein_coding	NP_001017998.1;NP_001185593.1	-2.4
CAMK2N1	calcium/calmodulin dependent protein kinase II in	protein_coding	NP_061054.2	-2.4
RUNX1T1	RUNX1 partner transcriptional co-repressor 1	protein_coding	NP_001185554.1;NP_001185555.1;	-2.4
CCDC84-DT	CCDC84 divergent transcript	lncRNA	.	-2.4
AP4M1	adaptor related protein complex 4 subunit mu 1	protein_coding	NP_001350600.1;NP_004713.2;XP_	-2.4
AFMID	arylformamidase	protein_coding	NP_001010982.2;NP_001138998.1;	-2.4
UROD	uroporphyrinogen decarboxylase	protein_coding	NP_000365.3	-2.4
ABHD4	abhydrolase domain containing 4, N-acyl phospho	protein_coding	NP_071343.2;XP_005268043.1	-2.4
SH3D21	SH3 domain containing 21	protein_coding	NP_001156002.1;NP_078952.4;XP_	-2.4
RNF220	ring finger protein 220	protein_coding	NP_001306885.1;NP_001306886.1;	-2.4
FAM86FP	family with sequence similarity 86 member F, pse	ranscribed_pseudogen	.	-2.4
PORCN	porcupine O-acyltransferase	protein_coding	NP_001269096.1;NP_073736.2;NP_	-2.4
NID1	nidogen 1	protein_coding	NP_002499.2;XP_011542497.1	-2.4
ZNF592	zinc finger protein 592	protein_coding	NP_055445.2;XP_005255053.1;XP_	-2.4
BRPF1	bromodomain and PHD finger containing 1	protein_coding	NP_001003694.1;NP_001305978.1;	-2.4
TRIM35	tripartite motif containing 35	protein_coding	NP_001291424.1;NP_001349742.1;	-2.4

BRPF3	bromodomain and PHD finger containing 3	protein_coding	NP_056510.2;XP_005249067.1;XP_	-2.4
TMEM236	transmembrane protein 236	protein_coding	NP_001092314.1;XP_011517928.1;	-2.4
SCARNA12	small Cajal body-specific RNA 12	guide_RNA	.	-2.4
C19orf12	chromosome 19 open reading frame 12	protein_coding	NP_001026896.2;NP_001242975.1;	-2.4
VIM	vimentin	protein_coding	NP_003371.2;XP_006717563.1	-2.4
MINPP1	multiple inositol-polyphosphate phosphatase 1	protein_coding	NP_001171588.1;NP_001171589.1;	-2.4
GBA	glucosylceramidase beta	protein_coding	NP_000148.2;NP_001005741.1;NP_	-2.4
LHX4	LIM homeobox 4	protein_coding	NP_203129.1;XP_011508407.1;XP_	-2.4
PI4KB	phosphatidylinositol 4-kinase beta	protein_coding	NP_001185702.1;NP_001185703.1;	-2.4
CLDN11	claudin 11	protein_coding	NP_001171985.1;NP_005593.2	-2.4
SHROOM3	shroom family member 3	protein_coding	NP_065910.3	-2.4
ATXN7L2	ataxin 7 like 2	protein_coding	NP_001337103.1;NP_001337104.1;	-2.4
LOC105376707	uncharacterized LOC105376707	lncRNA	.	-2.4
ZNF681	zinc finger protein 681	protein_coding	NP_612143.2	-2.4
CNOT11	CCR4-NOT transcription complex subunit 11	protein_coding	NP_060016.3	-2.4
TMEM217	transmembrane protein 217	protein_coding	NP_001156372.1;NP_001273330.1;	-2.4
LOC102724378	uncharacterized LOC102724378	lncRNA	.	-2.4
SERTAD4	SERTA domain containing 4	protein_coding	NP_001341102.1;NP_001362357.1;	-2.4
GPN2	GPN-loop GTPase 2	protein_coding	NP_060536.3	-2.4
CCDC115	coiled-coil domain containing 115	protein_coding	NP_001308047.1;NP_001308048.2;	-2.4
NDFIP1	Nedd4 family interacting protein 1	protein_coding	NP_085048.1	-2.4
SPARC	secreted protein acidic and cysteine rich	protein_coding	NP_001296372.1;NP_001296373.1;	-2.4
ENO2	enolase 2	protein_coding	NP_001966.1	-2.4
MXRA7	matrix remodeling associated 7	protein_coding	NP_001008528.1;NP_001008529.1;	-2.4
AMFR	autocrine motility factor receptor	protein_coding	NP_001135.3;NP_001310440.1;NP_	-2.4
RNF122	ring finger protein 122	protein_coding	NP_079063.2;XP_016869335.1	-2.4
SP2	Sp2 transcription factor	protein_coding	NP_003101.3;XP_006722086.1;XP_	-2.4
SPOCK2	SPARC (osteonectin), cwcv and kazal like domair	protein_coding	NP_001127906.1;NP_001231879.1;	-2.4
TPI1P1	triosephosphate isomerase 1 pseudogene 1	pseudogene	.	-2.5
IQCN	IQ motif containing N	protein_coding	NP_001138776.1;NP_001138777.1;	-2.5
DND1	DND microRNA-mediated repression inhibitor 1	protein_coding	NP_919225.1	-2.5
BPHL	biphenyl hydrolase like	protein_coding	NP_001289706.1;NP_004323.2	-2.5
MRPL2	mitochondrial ribosomal protein L2	protein_coding	NP_001287777.1;NP_057034.2;XP_	-2.5
CD14	CD14 molecule	protein_coding	NP_000582.1;NP_001035110.1;NP_	-2.5
PLEKHN1	pleckstrin homology domain containing N1	protein_coding	NP_001153656.1;NP_001354481.1;	-2.5
LLPH-DT	LLPH divergent transcript	lncRNA	.	-2.5
MCM7	minichromosome maintenance complex componer	protein_coding	NP_001265524.1;NP_005907.3;NP_	-2.5
CTSB	cathepsin B	protein_coding	NP_001304166.1;NP_001899.1;NP_	-2.5
ATP8B2	ATPase phospholipid transporting 8B2	protein_coding	NP_001005855.1;NP_001354863.1;	-2.5
LOC105373159	uncharacterized LOC105373159	lncRNA	.	-2.5
LOC101927604	uncharacterized LOC101927604	lncRNA	.	-2.5
ICAM3	intercellular adhesion molecule 3	protein_coding	NP_001307534.1;NP_001307535.1;	-2.5
SCARNA21	small Cajal body-specific RNA 21	guide_RNA	.	-2.5
KCNH1	potassium voltage-gated channel subfamily H mer	protein_coding	NP_002229.1;NP_758872.1;XP_01(	-2.5
DMAC2	distal membrane arm assembly complex 2	protein_coding	NP_001161339.1;NP_001161340.1;	-2.5
LPCAT4	lysophosphatidylcholine acyltransferase 4	protein_coding	NP_705841.2;XP_006720517.1;XP_	-2.5
MRPL20-AS1	MRPL20 antisense RNA 1	lncRNA	.	-2.5
RRBP1	ribosome binding protein 1	protein_coding	NP_001036041.2;NP_001352542.1;	-2.5
TACO1	translational activator of cytochrome c oxidase I	protein_coding	NP_057444.2	-2.5
H4-16	H4 histone 16	protein_coding	NP_778224.1	-2.5
PPP1R3D	protein phosphatase 1 regulatory subunit 3D	protein_coding	NP_006233.1	-2.5
BTG3-AS1	BTG3 antisense RNA 1	lncRNA	.	-2.5
FIS1	fission, mitochondrial 1	protein_coding	NP_057152.2	-2.5
YPEL3	yippee like 3	protein_coding	NP_001138996.1;NP_113665.3	-2.5
HMGN2P4	high mobility group nucleosomal binding domain	pseudogene	.	-2.5
VOPP1	VOPP1 WW domain binding protein	protein_coding	NP_001271211.1;NP_001271212.1;	-2.5
PCK2	phosphoenolpyruvate carboxykinase 2, mitochond	protein_coding	NP_001018083.2;NP_001278485.1;	-2.5
RASSF2	Ras association domain family member 2	protein_coding	NP_055552.1;NP_739580.1;XP_00:	-2.5
PRR5L	proline rich 5 like	protein_coding	NP_001153639.1;NP_001153640.1;	-2.5
DTD2	D-aminoacyl-tRNA deacylase 2	protein_coding	NP_542395.1	-2.5
TNC	tenascin C	protein_coding	NP_002151.2;XP_005252029.1;XP_	-2.5
GINS4	GINS complex subunit 4	protein_coding	NP_115712.1;XP_005273716.1	-2.5
KIAA1143P1	KIAA1143 pseudogene 1	pseudogene	.	-2.5
OSBPL7	oxysterol binding protein like 7	protein_coding	NP_665741.1;XP_016879611.1;XP_	-2.5
TRNF	tRNA-Phe	tRNA	.	-2.5
DDX28	DEAD-box helicase 28	protein_coding	NP_060850.2	-2.5
NFE2L1	nuclear factor, erythroid 2 like 1	protein_coding	NP_001317190.1;NP_001317191.1;	-2.5
YBEY	ybeY metalloendoribonuclease	protein_coding	NP_001006114.1;NP_001300951.1;	-2.5
PAMR1	peptidase domain containing associated with musc	protein_coding	NP_001001991.1;NP_001269604.1;	-2.5
TMEM101	transmembrane protein 101	protein_coding	NP_001291742.1;NP_001291743.1;	-2.5
CERS2	ceramide synthase 2	protein_coding	NP_071358.1;NP_859530.1;XP_01	-2.5

ANXA11	annexin A11	protein_coding	NP_001148.1;NP_001265336.1;NP_	-2.5
IGFBP5	insulin like growth factor binding protein 5	protein_coding	NP_000590.1	-2.5
LOC107984658	uncharacterized LOC107984658	lncRNA	.	-2.5
ZNF385B	zinc finger protein 385B	protein_coding	NP_001106868.1;NP_001106869.1;	-2.5
CDKN2B	cyclin dependent kinase inhibitor 2B	protein_coding	NP_004927.2;NP_511042.1	-2.5
MRPL43	mitochondrial ribosomal protein L43	protein_coding	NP_001295325.1;NP_115488.2;NP_	-2.5
MEDAG	mesenteric estrogen dependent adipogenesis	protein_coding	NP_116238.3;XP_016876290.1	-2.5
CIITA	class II major histocompatibility complex transacti	protein_coding	NP_000237.2;NP_001273331.1;NP_	-2.5
TRG-GCC1-5	tRNA-Gly	tRNA	.	-2.5
RASSF5	Ras association domain family member 5	protein_coding	NP_872604.1;NP_872605.1;NP_87	-2.5
SRCAP	Snf2 related CREBBP activator protein	protein_coding	NP_006653.2	-2.5
ATG4B	autophagy related 4B cysteine peptidase	protein_coding	NP_037457.3;NP_847896.1;XP_00	-2.5
GTF3C2	general transcription factor IIIC subunit 2	protein_coding	NP_001030598.1;NP_001305838.1;	-2.5
SPAG4	sperm associated antigen 4	protein_coding	NP_001304860.1;NP_003107.1;XP_	-2.5
H6PD	hexose-6-phosphate dehydrogenase/glucose 1-deh	protein_coding	NP_001269516.1;NP_004276.2;XP_	-2.5
SCARNA10	small Cajal body-specific RNA 10	guide_RNA	.	-2.5
CAMK1D	calcium/calmodulin dependent protein kinase ID	protein_coding	NP_001337961.1;NP_065130.1;NP_	-2.5
PABPC4	poly(A) binding protein cytoplasmic 4	protein_coding	NP_001129125.1;NP_001129126.1;	-2.5
S100A10	S100 calcium binding protein A10	protein_coding	NP_002957.1	-2.5
ITPA	inosine triphosphatase	protein_coding	NP_001254552.1;NP_001311165.1;	-2.5
DNAJB1	DnaJ heat shock protein family (Hsp40) member I	protein_coding	NP_001287843.1;NP_001300893.1;	-2.5
UNG	uracil DNA glycosylase	protein_coding	NP_003353.1;NP_550433.1	-2.5
TUBBP1	tubulin beta pseudogene 1	pseudogene	.	-2.5
PGP	phosphoglycolate phosphatase	protein_coding	NP_001035830.1	-2.5
GPKOW	G-patch domain and KOW motifs	protein_coding	NP_056513.2;XP_016884904.1	-2.5
ZNF786	zinc finger protein 786	protein_coding	NP_689624.2	-2.5
MZF1-AS1	MZF1 antisense RNA 1	lncRNA	.	-2.5
KCTD2	potassium channel tetramerization domain contain	protein_coding	NP_056168.1	-2.5
RRP1B	ribosomal RNA processing 1B	protein_coding	NP_055871.1	-2.5
DCTN2	dynactin subunit 2	protein_coding	NP_001248341.1;NP_001248342.1;	-2.5
IGFBP6	insulin like growth factor binding protein 6	protein_coding	NP_002169.1	-2.5
C1orf115	chromosome 1 open reading frame 115	protein_coding	NP_078985.3	-2.5
ETFBKMT	electron transfer flavoprotein subunit beta lysine n	protein_coding	NP_001129335.1;NP_001129336.1;	-2.5
PLEKHA2	pleckstrin homology domain containing A2	protein_coding	NP_067636.1;XP_011542907.1;XP_	-2.5
EDF1	endothelial differentiation related factor 1	protein_coding	NP_001268226.1;NP_001268227.1;	-2.5
NNMT	nicotinamide N-methyltransferase	protein_coding	NP_001358974.1;NP_001358975.1;	-2.5
FTSJ1	FtsJ RNA 2'-O-methyltransferase 1	protein_coding	NP_001269086.1;NP_036412.1;NP_	-2.5
CD34	CD34 molecule	protein_coding	NP_001020280.1;NP_001764.1	-2.5
NR4A1	nuclear receptor subfamily 4 group A member 1	protein_coding	NP_001189162.1;NP_001189163.1;	-2.5
CSNK2B	casein kinase 2 beta	protein_coding	NP_001269314.1;NP_001311.3	-2.5
ARHGEF39	Rho guanine nucleotide exchange factor 39	protein_coding	NP_116207.2;XP_011516359.1;XP_	-2.5
ABCC10	ATP binding cassette subfamily C member 10	protein_coding	NP_001185863.1;NP_001337447.1;	-2.5
CLCF1	cardiotrophin like cytokine factor 1	protein_coding	NP_001159684.1;NP_037378.1	-2.5
LOC339803	uncharacterized LOC339803	lncRNA	.	-2.5
TAP1	transporter 1, ATP binding cassette subfamily B m	protein_coding	NP_000584.3;NP_001278951.1	-2.5
PIH1D1	PIH1 domain containing 1	protein_coding	NP_060386.1;XP_024307336.1;XP_	-2.5
H2BC12	H2B clustered histone 12	protein_coding	NP_001299582.1;NP_542160.1	-2.5
MON1B	MON1 homolog B, secretory trafficking associate	protein_coding	NP_001273568.1;NP_001273569.1;	-2.5
LOC643576	phosphoglycerate mutase 1 (brain) pseudogene	pseudogene	.	-2.5
DNAJC14	DnaJ heat shock protein family (Hsp40) member C	protein_coding	NP_115740.5	-2.5
SMPD2	sphingomyelin phosphodiesterase 2	protein_coding	NP_003071.2;XP_005267166.1;XP_	-2.5
RNVU1-18	RNA, variant U1 small nuclear 18	snRNA	.	-2.5
TRERF1	transcriptional regulating factor 1	protein_coding	NP_001284502.1;NP_277037.1;XP_	-2.5
MAPK4	mitogen-activated protein kinase 4	protein_coding	NP_001278968.1;NP_001278969.1;	-2.5
RAB11B-AS1	RAB11B antisense RNA 1	lncRNA	.	-2.5
TRAM2	translocation associated membrane protein 2	protein_coding	NP_036420.1;XP_011513307.1	-2.5
SIM1	SIM bHLH transcription factor 1	protein_coding	NP_001361698.1;NP_005059.2;XP_	-2.5
HMGN2P41	high mobility group nucleosomal binding domain	pseudogene	.	-2.5
PICK1	protein interacting with PRKCA 1	protein_coding	NP_001034672.1;NP_001034673.1;	-2.5
TUBA1C	tubulin alpha 1c	protein_coding	NP_001290043.1;NP_001290044.1;	-2.5
FAM8A1	family with sequence similarity 8 member A1	protein_coding	NP_057339.1	-2.5
HNRNPM	heterogeneous nuclear ribonucleoprotein M	protein_coding	NP_001284347.1;NP_005959.2;NP_	-2.5
UBN1	ubinuclein 1	protein_coding	NP_001072982.1;NP_001275585.1;	-2.5
CAPZB	capping actin protein of muscle Z-line subunit beta	protein_coding	NP_001193469.1;NP_001193470.1;	-2.5
ZNF723	zinc finger protein 723	protein_coding	NP_001336655.1;XP_024307420.1;	-2.5
ICMT	isoprenylcysteine carboxyl methyltransferase	protein_coding	NP_036537.1;XP_011539442.1	-2.5
PARS2	prolyl-tRNA synthetase 2, mitochondrial	protein_coding	NP_689481.2	-2.5
TRMT1	tRNA methyltransferase 1	protein_coding	NP_001129507.1;NP_001136026.1;	-2.5
LOC105374118	uncharacterized LOC105374118	lncRNA	.	-2.5
CYB561D1	cytochrome b561 family member D1	protein_coding	NP_001127872.1;NP_001127874.1;	-2.5
TCAF1	TRPM8 channel associated factor 1	protein_coding	NP_001193867.2;NP_001193870.1;	-2.5

SYVN1	synoviolin 1	protein_coding	NP_115807.1;NP_757385.1;XP_01	-2.5
HMGN2P7	high mobility group nucleosomal binding domain	pseudogene	.	-2.5
LATS2	large tumor suppressor kinase 2	protein_coding	NP_055387.2;XP_005266399.1;XP_	-2.5
AEN	apoptosis enhancing nuclease	protein_coding	NP_073604.3;XP_005255023.1;XP_	-2.5
DCAF11	DDB1 and CUL4 associated factor 11	protein_coding	NP_001156956.1;NP_079506.3;NP_	-2.5
UPK3BL1	uroplakin 3B like 1	protein_coding	NP_001107875.1	-2.5
MAST2	microtubule associated serine/threonine kinase 2	protein_coding	NP_001306174.1;NP_001311249.1;	-2.5
SESN3	sestrin 3	protein_coding	NP_001258523.1;NP_653266.2;XP_	-2.5
FOSL2	FOS like 2, AP-1 transcription factor subunit	protein_coding	NP_005244.1;XP_005264288.1;XP_	-2.5
TLE1	TLE family member 1, transcriptional corepressor	protein_coding	NP_001290032.1;NP_001290033.1;	-2.5
SDF2L1	stromal cell derived factor 2 like 1	protein_coding	NP_071327.2	-2.5
GEMIN4	gem nuclear organelle associated protein 4	protein_coding	NP_056536.2;XP_005256724.1;XP_	-2.5
WASH7P	WASP family homolog 7, pseudogene	ranscribed_pseudogene	.	-2.5
SLC5A6	solute carrier family 5 member 6	protein_coding	NP_066918.2;XP_006712191.1;XP_	-2.5
CHST7	carbohydrate sulfotransferase 7	protein_coding	NP_063939.2	-2.5
HIGD2A	HIG1 hypoxia inducible domain family member 2	protein_coding	NP_620175.1	-2.5
REEP1	receptor accessory protein 1	protein_coding	NP_001158202.1;NP_001158203.1;	-2.5
ELP5	elongator acetyltransferase complex subunit 5	protein_coding	NP_056177.3;NP_981958.1;NP_98	-2.5
TCAF1P1	TRPM8 channel associated factor 1 pseudogene 1	pseudogene	.	-2.5
C17orf49	chromosome 17 open reading frame 49	protein_coding	NP_001136270.1;NP_001136271.1;	-2.5
SOWAHC	sosondowah ankyrin repeat domain family membe	protein_coding	NP_075392.2	-2.5
ITPKB	inositol-trisphosphate 3-kinase B	protein_coding	NP_002212.3;XP_005273177.1;XP_	-2.5
RPL28	ribosomal protein L28	protein_coding	NP_000982.2;NP_001129606.1;NP_	-2.6
NEK6	NIMA related kinase 6	protein_coding	NP_001138473.1;NP_001159639.1;	-2.6
GPS2	G protein pathway suppressor 2	protein_coding	NP_004480.1	-2.6
SLC38A7	solute carrier family 38 member 7	protein_coding	NP_001295313.1;NP_001356537.1;	-2.6
EIF3B	eukaryotic translation initiation factor 3 subunit B	protein_coding	NP_001032360.1;NP_001349720.1;	-2.6
LYRM7	LYR motif containing 7	protein_coding	NP_001280664.1;NP_859056.2	-2.6
SKOR1	SKI family transcriptional corepressor 1	protein_coding	NP_001352844.1	-2.6
ZNF570	zinc finger protein 570	protein_coding	NP_001287922.1;NP_001308920.1;	-2.6
NARF	nuclear prelamin A recognition factor	protein_coding	NP_001033707.1;NP_001077077.1;	-2.6
DDX51	DEAD-box helicase 51	protein_coding	NP_778236.2;XP_011536558.1	-2.6
HMBS	hydroxymethylbilane synthase	protein_coding	NP_000181.2;NP_001019553.1;NP_	-2.6
CRISPLD2	cysteine rich secretory protein LCCL domain cont	protein_coding	NP_113664.1;XP_005256247.1;XP_	-2.6
QARS1	glutaminyl-tRNA synthetase 1	protein_coding	NP_001259002.1;NP_005042.1;XP_	-2.6
ADGRG1	adhesion G protein-coupled receptor G1	protein_coding	NP_001139242.1;NP_001139243.1;	-2.6
SHB	SH2 domain containing adaptor protein B	protein_coding	NP_003019.2	-2.6
LARP1P1	LARP1 pseudogene 1	pseudogene	.	-2.6
GM2A	GM2 ganglioside activator	protein_coding	NP_000396.2;NP_001161079.1	-2.6
CINP	cyclin dependent kinase 2 interacting protein	protein_coding	NP_001306975.1;NP_116019.1	-2.6
DGCR11	DiGeorge syndrome critical region gene 11	lncRNA	.	-2.6
BIN3	bridging integrator 3	protein_coding	NP_001349975.1;NP_061158.1;XP_	-2.6
PBX1	PBX homeobox 1	protein_coding	NP_001191890.1;NP_001191892.1;	-2.6
GAPDH	glyceraldehyde-3-phosphate dehydrogenase	protein_coding	NP_001243728.1;NP_001276674.1;	-2.6
TRMU	tRNA 5-methylaminomethyl-2-thiouridylate meth	protein_coding	NP_001269711.1;NP_001269712.1;	-2.6
YPEL1	yippee like 1	protein_coding	NP_037445.1	-2.6
CYTH2	cytohesin 2	protein_coding	NP_004219.3;NP_059431.1;XP_00	-2.6
SCUBE3	signal peptide, CUB domain and EGF like domain	protein_coding	NP_001290065.1;NP_689966.2;XP_	-2.6
HHLA3	HERV-H LTR-associating 3	lncRNA	.	-2.6
NMB	neuromedin B	protein_coding	NP_066563.2;NP_995580.1;XP_01	-2.6
PARM1	prostate androgen-regulated mucin-like protein 1	protein_coding	NP_056208.2;XP_011530135.1	-2.6
STAT6	signal transducer and activator of transcription 6	protein_coding	NP_001171549.1;NP_001171550.1;	-2.6
CDK20	cyclin dependent kinase 20	protein_coding	NP_001034892.1;NP_001164110.1;	-2.6
LGALS3	galectin 3	protein_coding	NP_001344607.1;NP_002297.2	-2.6
PGM1	phosphoglucomutase 1	protein_coding	NP_001166289.1;NP_001166290.1;	-2.6
LIG1	DNA ligase 1	protein_coding	NP_000225.1;NP_001275992.1;NP_	-2.6
RN7SK	RNA component of 7SK nuclear ribonucleoprotei	snRNA	.	-2.6
MAN1B1	mannosidase alpha class 1B member 1	protein_coding	NP_057303.2;XP_006717008.1;XP_	-2.6
TLCD1	TLC domain containing 1	protein_coding	NP_001153879.1;NP_612472.1;XP_	-2.6
DFFA	DNA fragmentation factor subunit alpha	protein_coding	NP_004392.1;NP_998731.1	-2.6
SIRT3	sirtuin 3	protein_coding	NP_001017524.1;NP_001357239.1;	-2.6
SNX12	sorting nexin 12	protein_coding	NP_001243114.1;NP_001243115.1;	-2.6
COL8A1	collagen type VIII alpha 1 chain	protein_coding	NP_001841.2;NP_065084.2	-2.6
AKAP17A	A-kinase anchoring protein 17A	protein_coding	NP_005079.2	-2.6
TMEM80	transmembrane protein 80	protein_coding	NP_001035928.2;NP_001263182.1;	-2.6
DDRGK1	DDRGK domain containing 1	protein_coding	NP_076424.1	-2.6
ADAP1	ArfGAP with dual PH domains 1	protein_coding	NP_001271237.1;NP_001271238.1;	-2.6
POLR2L	RNA polymerase II subunit L	protein_coding	NP_066951.1	-2.6
PFKFB3	6-phosphofructo-2-kinase/fructose-2,6-biphosphat	protein_coding	NP_001138915.1;NP_001269559.1;	-2.6
CCDC61	coiled-coil domain containing 61	protein_coding	NP_001254652.1;XP_005259248.1;	-2.6
TNNT1	troponin T1, slow skeletal type	protein_coding	NP_001119604.1;NP_001119605.1;	-2.6

PSMG3	proteasome assembly chaperone 3	protein_coding	NP_001127812.1;NP_115678.1;XP_	-2.6
HCG15	HLA complex group 15	lncRNA	.	-2.6
CAV2	caveolin 2	protein_coding	NP_001193676.1;NP_001193677.1;	-2.6
CASC3	CASC3 exon junction complex subunit	protein_coding	NP_031385.2;XP_005257220.1	-2.6
FOXK2	forkhead box K2	protein_coding	NP_004505.2;XP_024306507.1	-2.6
FTH1	ferritin heavy chain 1	protein_coding	NP_002023.2	-2.6
DCPS	decapping enzyme, scavenger	protein_coding	NP_001337165.1;NP_054745.1	-2.6
LOC101928868	uncharacterized LOC101928868	lncRNA	.	-2.6
PCDHB11	protocadherin beta 11	protein_coding	NP_061754.1	-2.6
COL5A2	collagen type V alpha 2 chain	protein_coding	NP_000384.2;XP_011508875.1	-2.6
LINC01275	long intergenic non-protein coding RNA 1275, tra	lncRNA	.	-2.6
FOXJ2	forkhead box J2	protein_coding	NP_060886.1;XP_011519062.1;XP_	-2.6
H2AC8	H2A clustered histone 8	protein_coding	NP_066390.1	-2.6
SLC2A1-AS1	SLC2A1 antisense RNA 1	lncRNA	.	-2.6
CCDC142	coiled-coil domain containing 142	protein_coding	NP_001352504.1;NP_116168.3	-2.6
ZNF486	zinc finger protein 486	protein_coding	NP_443084.2	-2.6
C11orf24	chromosome 11 open reading frame 24	protein_coding	NP_001287842.1;NP_071733.1;XP_	-2.6
SFT2D3	SFT2 domain containing 3	protein_coding	NP_116129.3	-2.6
GADD45B	growth arrest and DNA damage inducible beta	protein_coding	NP_056490.2;XP_016882311.1	-2.6
FOXG1	forkhead box G1	protein_coding	NP_005240.3	-2.6
ABAT	4-aminobutyrate aminotransferase	protein_coding	NP_000654.2;NP_001120920.1;NP_	-2.6
MOCS3	molybdenum cofactor synthesis 3	protein_coding	NP_055299.1	-2.6
RGS12	regulator of G protein signaling 12	protein_coding	NP_002917.1;NP_937870.1;NP_93	-2.6
SYNM	synemin	protein_coding	NP_056101.5;NP_663780.2;XP_01	-2.6
PI4K2A	phosphatidylinositol 4-kinase type 2 alpha	protein_coding	NP_060895.1	-2.6
PPP1R3C	protein phosphatase 1 regulatory subunit 3C	protein_coding	NP_005389.1	-2.6
MTCL1	microtubule crosslinking factor 1	protein_coding	NP_001365134.1;NP_001365135.1;	-2.6
RRAD	RRAD, Ras related glycolysis inhibitor and calciu	protein_coding	NP_001122322.1;NP_004156.1	-2.6
CHRNB1	cholinergic receptor nicotinic beta 1 subunit	protein_coding	NP_000738.2	-2.6
UCN	urocortin	protein_coding	NP_003344.1	-2.6
NCAPD2	non-SMC condensin I complex subunit D2	protein_coding	NP_055680.3	-2.6
C2CD2L	C2CD2 like	protein_coding	NP_001277403.1;NP_001369540.1;	-2.6
WASH8P	WAS protein family homolog 8, pseudogene	ranscribed_pseudogen	.	-2.6
CGN	cingulin	protein_coding	NP_065821.1;XP_005245422.1	-2.6
MIR1244-2	microRNA 1244-2	miRNA	.	-2.6
ARID1A	AT-rich interaction domain 1A	protein_coding	NP_006006.3;NP_624361.1	-2.6
PRKAR2B	protein kinase cAMP-dependent type II regulatory	protein_coding	NP_002727.2;XP_011514700.1;XP_	-2.6
ELAVL1	ELAV like RNA binding protein 1	protein_coding	NP_001410.2	-2.6
CLTA	clathrin light chain A	protein_coding	NP_001070145.1;NP_001171689.1;	-2.6
SURF6	surfeit 6	protein_coding	NP_001265871.1;NP_006744.2	-2.6
KHDRBS3	KH RNA binding domain containing, signal transc	protein_coding	NP_006549.1;XP_005250814.1;XP_	-2.6
P4HB	prolyl 4-hydroxylase subunit beta	protein_coding	NP_000909.2;XP_024306545.1	-2.7
TAF4	TATA-box binding protein associated factor 4	protein_coding	NP_003176.2	-2.7
TGM1	transglutaminase 1	protein_coding	NP_000350.1	-2.7
UPK3BL2	uroplakin 3B like 2	protein_coding	NP_001350435.1	-2.7
SMKR1	small lysine rich protein 1	protein_coding	NP_001182172.1;XP_024302388.1	-2.7
BASP1	brain abundant membrane attached signal protein	protein_coding	NP_001258535.1;NP_006308.3	-2.7
ROBO4	roundabout guidance receptor 4	protein_coding	NP_001288017.1;NP_061928.4;XP_	-2.7
CEP20	centrosomal protein 20	protein_coding	NP_001291426.1;NP_001291427.1;	-2.7
KIF3C	kinesin family member 3C	protein_coding	NP_002245.4;XP_005264356.2	-2.7
SERTAD4-AS1	SERTAD4 antisense RNA 1	lncRNA	.	-2.7
IGFBP3	insulin like growth factor binding protein 3	protein_coding	NP_000589.2;NP_001013416.1	-2.7
MTG1	mitochondrial ribosome associated GTPase 1	protein_coding	NP_612393.2	-2.7
ATRIP	ATR interacting protein	protein_coding	NP_001257951.1;NP_001257952.1;	-2.7
URGCP	upregulator of cell proliferation	protein_coding	NP_001071131.1;NP_001071132.1;	-2.7
WASH9P	WAS protein family homolog 9, pseudogene	ranscribed_pseudogen	.	-2.7
HLA-B	major histocompatibility complex, class I, B	protein_coding	NP_005505.2	-2.7
AAR2	AAR2 splicing factor	protein_coding	NP_001258803.1;NP_056326.2;XP_	-2.7
PPRC1	PPARG related coactivator 1	protein_coding	NP_001275656.1;NP_001275657.1;	-2.7
POFUT2	protein O-fucosyltransferase 2	protein_coding	NP_056042.1;NP_598368.2;XP_00	-2.7
FKBPL	FKBP prolyl isomerase like	protein_coding	NP_071393.2	-2.7
BANF1P3	BANF1 pseudogene 3	pseudogene	.	-2.7
KHDRBS1	KH RNA binding domain containing, signal transc	protein_coding	NP_001258807.1;NP_006550.1	-2.7
LOC107984895	uncharacterized LOC107984895	lncRNA	.	-2.7
MKRN3	makorin ring finger protein 3	protein_coding	NP_005655.1	-2.7
SORL1	sortilin related receptor 1	protein_coding	NP_003096.2	-2.7
LOC107986925	uncharacterized LOC107986925, transcript varian	lncRNA	.	-2.7
UBXN2A	UBX domain protein 2A	protein_coding	NP_859064.2;XP_005264223.1;XP_	-2.7
PADI1	peptidyl arginine deiminase 1	protein_coding	NP_037490.2;XP_011539609.1;XP_	-2.7
SH3PXD2A	SH3 and PX domains 2A	protein_coding	NP_001352008.1;NP_055446.2	-2.7
STXBP1	syntaxin binding protein 1	protein_coding	NP_001027392.1;NP_001361235.1;	-2.7

SMG5	SMG5 nonsense mediated mRNA decay factor	protein_coding	NP_001310543.1;NP_001310544.1;	-2.7
MARCKSP1	myristoylated alanine rich protein kinase C substra	pseudogene	.	-2.7
CDPF1	cysteine rich DPF motif domain containing 1	protein_coding	NP_997210.3;XP_011528262.1;XP_	-2.7
PIGT	phosphatidylinositol glycan anchor biosynthesis cl	protein_coding	NP_001171657.1;NP_001171658.1;	-2.7
C1R	complement C1r	protein_coding	NP_001341275.1;NP_001724.4	-2.7
ADCY7	adenylate cyclase 7	protein_coding	NP_001105.1;NP_001272986.1;XP_	-2.7
C21orf58	chromosome 21 open reading frame 58	protein_coding	NP_001273391.1;NP_001273392.1;	-2.7
LOC642969	phosphoglycerate mutase 1 (brain) pseudogene	pseudogene	.	-2.7
VAMP5	vesicle associated membrane protein 5	protein_coding	NP_006625.1	-2.7
LSS	lanosterol synthase	protein_coding	NP_001001438.1;NP_001138908.1;	-2.7
MAML1	mastermind like transcriptional coactivator 1	protein_coding	NP_055572.1	-2.7
TUBAP2	tubulin alpha pseudogene 2	pseudogene	.	-2.7
TMEM40	transmembrane protein 40	protein_coding	NP_001271335.1;NP_001271336.1;	-2.7
GABARAP	GABA type A receptor-associated protein	protein_coding	NP_009209.1	-2.7
PKM	pyruvate kinase M1/2	protein_coding	NP_001193725.1;NP_001193726.1;	-2.7
LINC01311	long intergenic non-protein coding RNA 1311	lncRNA	.	-2.7
TDGP1	thymine-DNA glycosylase pseudogene 1	pseudogene	.	-2.7
DBF4B	DBF4 zinc finger B	protein_coding	NP_079380.2;NP_663696.1;XP_00(	-2.7
NUTM2E	NUT family member 2E	protein_coding	NP_001342192.1;XP_024303718.1;	-2.7
FGFR1	fibroblast growth factor receptor 1	protein_coding	NP_001167534.1;NP_001167535.1;	-2.7
WWC3	WWC family member 3	protein_coding	NP_056506.2	-2.7
PCDHGA10	protocadherin gamma subfamily A, 10	protein_coding	NP_061736.1;NP_114479.1	-2.7
FRZB	frizzled related protein	protein_coding	NP_001454.2	-2.7
LSM10	LSM10, U7 small nuclear RNA associated	protein_coding	NP_116270.1	-2.7
TENM2	teneurin transmembrane protein 2	protein_coding	NP_001073897.2;NP_001116151.1;	-2.7
CORO6	coronin 6	protein_coding	NP_001338230.1;NP_001338231.1;	-2.7
MTG2	mitochondrial ribosome associated GTPase 2	protein_coding	NP_056481.1;XP_005260450.1;XP_	-2.7
ZNF76	zinc finger protein 76	protein_coding	NP_001278961.1;NP_003418.2;XP_	-2.7
EIF4HP1	eukaryotic translation initiation factor 4H pseudog	pseudogene	.	-2.7
IPO5P1	importin 5 pseudogene 1	ranscribed_pseudogen	.	-2.7
ENO1	enolase 1	protein_coding	NP_001188412.1;NP_001340275.1;	-2.7
KCTD1	potassium channel tetramerization domain contain	protein_coding	NP_001129677.1;NP_001136202.1;	-2.7
HLA-H	major histocompatibility complex, class I, H (pseu	ranscribed_pseudogen	.	-2.7
NAPRT	nicotinate phosphoribosyltransferase	protein_coding	NP_001273758.1;NP_001350074.1;	-2.7
PCDHGA6	protocadherin gamma subfamily A, 6	protein_coding	NP_061742.1;NP_114475.1	-2.7
ERAL1	Era like 12S mitochondrial rRNA chaperone 1	protein_coding	NP_001304914.1;NP_001304915.1;	-2.7
LOC105369663	uncharacterized LOC105369663	lncRNA	.	-2.7
LINC02475	long intergenic non-protein coding RNA 2475	lncRNA	.	-2.7
MFN2	mitofusin 2	protein_coding	NP_001121132.1;NP_055689.1;XP_	-2.7
PTTG1IP	PTTG1 interacting protein	protein_coding	NP_001273751.1;NP_004330.1	-2.7
WBP1L	WW domain binding protein 1 like	protein_coding	NP_001077382.1;NP_060257.4;XP	-2.7
BATF3	basic leucine zipper ATF-like transcription factor	protein_coding	NP_061134.1;XP_016857172.1	-2.7
ARF3	ADP ribosylation factor 3	protein_coding	NP_001650.1;XP_005268913.1;XP	-2.7
CNTNAP1	contactin associated protein 1	protein_coding	NP_003623.1;XP_005257805.1;XP_	-2.7
ZNF70	zinc finger protein 70	protein_coding	NP_068735.1;XP_024308037.1;XP	-2.7
TLL1	tolloid like 1	protein_coding	NP_001191689.1;NP_036596.3;XP	-2.7
EEPD1	endonuclease/exonuclease/phosphatase family dor	protein_coding	NP_085139.2;XP_016868146.1;XP_	-2.7
SMAD7	SMAD family member 7	protein_coding	NP_001177750.1;NP_001177751.1;	-2.7
ZNF513	zinc finger protein 513	protein_coding	NP_001188388.1;NP_653232.3;XP_	-2.7
NHP2	NHP2 ribonucleoprotein	protein_coding	NP_001030005.1;NP_060308.1	-2.7
KLF5	Kruppel like factor 5	protein_coding	NP_001273747.1;NP_001721.2	-2.7
TUB	TUB bipartite transcription factor	protein_coding	NP_003311.2;NP_813977.1;XP_00	-2.7
HIVEP3	HIVEP zinc finger 3	protein_coding	NP_001121186.1;NP_078779.2;XP_	-2.7
AQP11	aquaporin 11	protein_coding	NP_001350406.1;NP_766627.1	-2.7
UBALD2	UBA like domain containing 2	protein_coding	NP_872371.1	-2.7
NDUFC2	NADH:ubiquinone oxidoreductase subunit C2	protein_coding	NP_001190983.1;NP_001190984.1;	-2.7
SCLY	selenocysteine lyase	protein_coding	NP_057594.5	-2.7
GRHPR	glyoxylate and hydroxypyruvate reductase	protein_coding	NP_036335.1;XP_005251688.1;XP_	-2.7
GALM	galactose mutarotase	protein_coding	NP_620156.1;XP_011530842.1	-2.7
SLITRK3	SLIT and NTRK like family member 3	protein_coding	NP_001305739.1;NP_001305740.1;	-2.7
SLC12A8	solute carrier family 12 member 8	protein_coding	NP_001182412.2;NP_078904.4	-2.7
SERINC2	serine incorporator 2	protein_coding	NP_001185966.1;NP_001185967.1;	-2.7
TCAF2	TRPM8 channel associated factor 2	protein_coding	NP_001123498.2;NP_001350467.1;	-2.7
DOCK3	dedicator of cytokinesis 3	protein_coding	NP_004938.1;XP_005264971.1;XP_	-2.7
SKP2	S-phase kinase associated protein 2	protein_coding	NP_001230049.1;NP_005974.2;NP_	-2.7
FBXO31	F-box protein 31	protein_coding	NP_001269612.1;NP_079011.3	-2.7
MRPS18B	mitochondrial ribosomal protein S18B	protein_coding	NP_054765.1;XP_024302176.1	-2.7
LPCAT3	lysophosphatidylcholine acyltransferase 3	protein_coding	NP_005759.4;XP_024304561.1	-2.7
DHCR7	7-dehydrocholesterol reductase	protein_coding	NP_001157289.1;NP_001351.2;XP_	-2.7
ZDHHC16	zinc finger DHHC-type palmitoyltransferase 16	protein_coding	NP_001274732.1;NP_001274733.1;	-2.7
TUBGCP6	tubulin gamma complex associated protein 6	protein_coding	NP_065194.3	-2.7

PCDHB12	protocadherin beta 12	protein_coding	NP_061755.1	-2.7
TTLL1	tubulin tyrosine ligase like 1	protein_coding	NP_036395.1;XP_006724288.1;XP_	-2.8
MSL3P1	MSL complex subunit 3 pseudogene 1	ranscribed_pseudogen	.	-2.8
PITX2	paired like homeodomain 2	protein_coding	NP_000316.2;NP_001191326.1;NP_	-2.8
PAM16	presequence translocase associated motor 16	protein_coding	NP_057153.8	-2.8
FLAD1	flavin adenine dinucleotide synthetase 1	protein_coding	NP_001171820.1;NP_001171821.1;	-2.8
KDM2B	lysine demethylase 2B	protein_coding	NP_001005366.1;NP_115979.3;XP_	-2.8
KIT	KIT proto-oncogene, receptor tyrosine kinase	protein_coding	NP_000213.1;NP_001087241.1;XP_	-2.8
PLBD2	phospholipase B domain containing 2	protein_coding	NP_001153199.1;NP_775813.2;XP_	-2.8
LOC100506253	uncharacterized LOC100506253	lncRNA	.	-2.8
EFEMP1	EGF containing fibulin extracellular matrix protei	protein_coding	NP_001034437.1;NP_001034438.1;	-2.8
EDIL3-DT	EDIL3 divergent transcript, transcript variant X1	lncRNA	.	-2.8
PPIL2	peptidylprolyl isomerase like 2	protein_coding	NP_001304925.1;NP_055152.1;NP_	-2.8
CTSH	cathepsin H	protein_coding	NP_001306066.1;NP_004381.2;XP_	-2.8
PTK2B	protein tyrosine kinase 2 beta	protein_coding	NP_004094.3;NP_775266.1;NP_77	-2.8
EPHB2	EPH receptor B2	protein_coding	NP_001296121.1;NP_001296122.1;	-2.8
CAPN6	calpain 6	protein_coding	NP_055104.2	-2.8
S100A5	S100 calcium binding protein A5	protein_coding	NP_002953.2;XP_011508162.1;XP_	-2.8
HK1	hexokinase 1	protein_coding	NP_000179.2;NP_001309293.1;NP_	-2.8
NEU1	neuraminidase 1	protein_coding	NP_000425.1	-2.8
LOC100506083	uncharacterized LOC100506083	lncRNA	.	-2.8
ADPRHL1	ADP-ribosylhydrolase like 1	protein_coding	NP_612439.2;NP_954631.1	-2.8
SLC35E1	solute carrier family 35 member E1	protein_coding	NP_079157.3	-2.8
RRS1	ribosome biogenesis regulator 1 homolog	protein_coding	NP_055984.1	-2.8
CPNE1	copine 1	protein_coding	NP_001185792.1;NP_003906.2;NP_	-2.8
TMEM187	transmembrane protein 187	protein_coding	NP_003483.1;XP_011529501.1	-2.8
KRT10	keratin 10	protein_coding	NP_000412.4;NP_001366295.1	-2.8
PGAM1	phosphoglycerate mutase 1	protein_coding	NP_001304008.1;NP_002620.1	-2.8
SPECC1L-ADORA2A	SPECC1L-ADORA2A readthrough (NMD candid	lncRNA	.	-2.8
SRSF9	serine and arginine rich splicing factor 9	protein_coding	NP_003760.1	-2.8
H2BC18	H2B clustered histone 18	protein_coding	NP_001019770.1;NP_001154806.1	-2.8
RBM24	RNA binding motif protein 24	protein_coding	NP_001137413.1;NP_001137414.1;	-2.8
MTA2	metastasis associated 1 family member 2	protein_coding	NP_001317221.1;NP_004730.2	-2.8
B9D2	B9 domain containing 2	protein_coding	NP_085055.2;XP_011525651.1;XP_	-2.8
VPS16	VPS16 core subunit of CORVET and HOPS comp	protein_coding	NP_072097.2;NP_536338.1	-2.8
SMAD9	SMAD family member 9	protein_coding	NP_001120689.1;NP_001365550.1;	-2.8
ZNF517	zinc finger protein 517	protein_coding	NP_001304865.1;NP_998770.2;XP_	-2.8
LYSMD2	LysM domain containing 2	protein_coding	NP_001137389.1;NP_001350898.1;	-2.8
GSE1	Gse1 coiled-coil protein	protein_coding	NP_001127945.1;NP_001265113.1;	-2.8
LRP11	LDL receptor related protein 11	protein_coding	NP_116221.3;XP_011534496.1;XP_	-2.8
WNT5B	Wnt family member 5B	protein_coding	NP_110402.2;NP_116031.1;XP_02	-2.8
MRGBP	MRG domain binding protein	protein_coding	NP_060740.1	-2.8
EPHA2	EPH receptor A2	protein_coding	NP_001316019.1;NP_004422.2;XP_	-2.8
HEXIM1	HEXIM P-TEFb complex subunit 1	protein_coding	NP_006451.1	-2.8
DHTKD1	dehydrogenase E1 and transketolase domain conta	protein_coding	NP_061176.4	-2.8
CCDC69	coiled-coil domain containing 69	protein_coding	NP_056436.2	-2.8
CDCA3	cell division cycle associated 3	protein_coding	NP_001284531.1;NP_001284532.1;	-2.8
HOXA1	homeobox A1	protein_coding	NP_005513.2;NP_705873.3	-2.8
YBX3	Y-box binding protein 3	protein_coding	NP_001138898.1;NP_003642.3;XP_	-2.8
FAM171A1	family with sequence similarity 171 member A1	protein_coding	NP_001010924.1;XP_011517680.1;	-2.8
FBL	fibrillarin	protein_coding	NP_001427.2;XP_005258708.1;XP_	-2.8
SDK1	sidekick cell adhesion molecule 1	protein_coding	NP_001073121.1;NP_689957.3;XP_	-2.8
TMEM127	transmembrane protein 127	protein_coding	NP_001180233.1;NP_060319.1;XP_	-2.8
LOC102725121	DEAD/H-box helicase 11 pseudogene	ranscribed_pseudogen	.	-2.8
SMARCD3	SWI/SNF related, matrix associated, actin depend	protein_coding	NP_001003801.1;NP_001003802.1;	-2.8
FBF1	Fas binding factor 1	protein_coding	NP_001306122.1	-2.8
TEPSIN	TEPSIN adaptor related protein complex 4 access	protein_coding	NP_001350693.1;NP_653280.1;XP_	-2.8
LOC107987293	uncharacterized LOC107987293	lncRNA	.	-2.8
CTNS	cystinosin, lysosomal cystine transporter	protein_coding	NP_001026851.2;NP_001361421.1;	-2.8
LRRK1	leucine rich repeat kinase 1	protein_coding	NP_078928.3;XP_005255036.1;XP_	-2.8
CNPY3	canopy FGF signaling regulator 3	protein_coding	NP_001305771.1;NP_001305774.1;	-2.8
RCC2	regulator of chromosome condensation 2	protein_coding	NP_001129676.1;NP_061185.1	-2.8
HMGN2P3	high mobility group nucleosomal binding domain	pseudogene	.	-2.8
PIP4P1	phosphatidylinositol-4,5-bisphosphate 4-phosphat	protein_coding	NP_001094284.1;NP_653169.2;XP_	-2.8
RERE	arginine-glutamic acid dipeptide repeats	protein_coding	NP_001036146.1;NP_001036147.1;	-2.8
GPT2	glutamic--pyruvic transaminase 2	protein_coding	NP_001135938.1;NP_597700.1;XP_	-2.8
NPM3	nucleophosmin/nucleoplasmin 3	protein_coding	NP_008924.1	-2.8
CDCA4	cell division cycle associated 4	protein_coding	NP_060425.2;NP_663747.1	-2.8
DAGLB	diacylglycerol lipase beta	protein_coding	NP_001136408.1;NP_631918.3	-2.8
RP1L1	RP1 like 1	protein_coding	NP_849188.4	-2.8
POLDIP3	DNA polymerase delta interacting protein 3	protein_coding	NP_001265586.1;NP_001349981.1;	-2.8

MMACHC	metabolism of cobalamin associated C	protein_coding	NP_001317469.1;NP_056321.2;XP_	-2.8
TRUB2	TruB pseudouridine synthase family member 2	protein_coding	NP_001316790.1;NP_001316791.1;	-2.8
PIGZ	phosphatidylinositol glycan anchor biosynthesis cl	protein_coding	NP_079439.2;XP_011511492.1;XP_	-2.8
TP73-AS1	TP73 antisense RNA 1	lncRNA	.	-2.8
TOR3A	torsin family 3 member A	protein_coding	NP_071766.2	-2.8
FAM161B	FAM161 centrosomal protein B	protein_coding	NP_689658.3;XP_011534777.1	-2.8
PYM1	PYM homolog 1, exon junction complex associate	protein_coding	NP_001137325.1;NP_115721.1	-2.8
LOC105374491	uncharacterized LOC105374491, transcript variant	lncRNA	.	-2.8
SOX4	SRY-box transcription factor 4	protein_coding	NP_003098.1	-2.8
PRECSIT	p53 regulated carcinoma associated Stat3 activatin	lncRNA	.	-2.9
VKORC1	vitamin K epoxide reductase complex subunit 1	protein_coding	NP_001298240.1;NP_076869.1;NP_	-2.9
CUTA	cutA divalent cation tolerance homolog	protein_coding	NP_001014433.2;NP_001014837.1;	-2.9
SETP14	SET pseudogene 14	pseudogene	.	-2.9
ANXA6	annexin A6	protein_coding	NP_001146.2;NP_001180473.1;NP_	-2.9
PGAP2	post-GPI attachment to proteins 2	protein_coding	NP_001138910.1;NP_001243164.1;	-2.9
AXIN2	axin 2	protein_coding	NP_001350742.1;NP_004646.3;XP_	-2.9
FNBP1	formin binding protein 1	protein_coding	NP_001350684.1;NP_055848.1;XP_	-2.9
LOC112268124	uncharacterized LOC112268124	lncRNA	.	-2.9
PDAP1	PDGFA associated protein 1	protein_coding	NP_055706.1	-2.9
WDR54	WD repeat domain 54	protein_coding	NP_001307752.1;NP_001307753.1;	-2.9
PCDHB10	protocadherin beta 10	protein_coding	NP_061753.1	-2.9
MZT1	mitotic spindle organizing protein 1	protein_coding	NP_001065243.1	-2.9
MAGED2	MAGE family member D2	protein_coding	NP_055414.2;NP_803182.1;NP_95	-2.9
EID2B	EP300 interacting inhibitor of differentiation 2B	protein_coding	NP_689574.1	-2.9
EEF2K	eukaryotic elongation factor 2 kinase	protein_coding	NP_037434.2;XP_016878682.1;XP_	-2.9
TMEM53	transmembrane protein 53	protein_coding	NP_001287675.1;NP_001287676.1;	-2.9
CPNE7	copine 7	protein_coding	NP_055242.1;NP_705900.1;XP_01	-2.9
RNASEK-C17orf49	RNASEK-C17orf49 readthrough	lncRNA	.	-2.9
LTBP3	latent transforming growth factor beta binding pro	protein_coding	NP_001123616.1;NP_001157738.1;	-2.9
SLC25A33	solute carrier family 25 member 33	protein_coding	NP_115691.1;XP_005263560.1;XP_	-2.9
PBX2	PBX homeobox 2	protein_coding	NP_002577.2	-2.9
AP4S1	adaptor related protein complex 4 subunit sigma 1	protein_coding	NP_001121598.1;NP_001241655.1;	-2.9
POLG	DNA polymerase gamma, catalytic subunit	protein_coding	NP_001119603.1;NP_002684.1	-2.9
ZGLP1	zinc finger GATA like protein 1	protein_coding	NP_001096637.1	-2.9
TSPAN14	tetraspanin 14	protein_coding	NP_001121781.1;NP_001338195.1;	-2.9
ZNRF1	zinc and ring finger 1	protein_coding	NP_115644.1;XP_011521694.1;XP_	-2.9
RPS19	ribosomal protein S19	protein_coding	NP_001013.1;NP_001308412.1;NP_	-2.9
LOC107986985	uncharacterized LOC107986985	lncRNA	.	-2.9
RASAL2-AS1	RASAL2 antisense RNA 1	lncRNA	.	-2.9
TMEM169	transmembrane protein 169	protein_coding	NP_001135782.1;NP_001135783.1;	-2.9
TFAP2A-AS1	TFAP2A antisense RNA 1	lncRNA	.	-2.9
ADCY6	adenylate cyclase 6	protein_coding	NP_056085.1;XP_006719273.1;XP_	-2.9
HNRNPAB	heterogeneous nuclear ribonucleoprotein A/B	protein_coding	NP_004490.2;NP_112556.2	-2.9
RHBDF1	rhomboid 5 homolog 1	protein_coding	NP_071895.3;XP_005255551.1;XP_	-2.9
RGS9	regulator of G protein signaling 9	protein_coding	NP_001075424.1;NP_001159405.1;	-2.9
BAMBI	BMP and activin membrane bound inhibitor	protein_coding	NP_036474.1	-2.9
LINC01786	long intergenic non-protein coding RNA 1786, tra	lncRNA	.	-2.9
DHX16	DEAH-box helicase 16	protein_coding	NP_001157711.1;NP_001350444.1;	-2.9
ERGIC1	endoplasmic reticulum-golgi intermediate compart	protein_coding	NP_001026881.1;XP_011532899.1;	-2.9
NR1D1	nuclear receptor subfamily 1 group D member 1	protein_coding	NP_068370.1	-2.9
ORMDL3	ORMDL sphingolipid biosynthesis regulator 3	protein_coding	NP_001307730.1;NP_001307731.1;	-2.9
LOXL3	lysyl oxidase like 3	protein_coding	NP_001276093.1;NP_001276094.1;	-2.9
FAM214B	family with sequence similarity 214 member B	protein_coding	NP_001304920.1;NP_079458.2;XP_	-2.9
SNORA73A	small nucleolar RNA, H/ACA box 73A	snoRNA	.	-2.9
DUSP19	dual specificity phosphatase 19	protein_coding	NP_001135786.1;NP_001308448.1;	-2.9
PKP2	plakophilin 2	protein_coding	NP_001005242.2;NP_004563.2	-2.9
TMEM147	transmembrane protein 147	protein_coding	NP_001229526.1;NP_001229527.1;	-2.9
DMAC1	distal membrane arm assembly complex 1	protein_coding	NP_001304987.1;NP_001304988.1;	-2.9
TIE1	tyrosine kinase with immunoglobulin like and EGI	protein_coding	NP_001240286.1;NP_005415.1;XP_	-2.9
SH3BP2	SH3 domain binding protein 2	protein_coding	NP_001116153.1;NP_001139327.1;	-2.9
NOL12	nucleolar protein 12	protein_coding	NP_077289.1	-2.9
PSENEN	presenilin enhancer, gamma-secretase subunit	protein_coding	NP_001268461.1;NP_758844.1	-2.9
LOC100129215	uncharacterized LOC100129215	lncRNA	.	-2.9
MED9	mediator complex subunit 9	protein_coding	NP_060489.1	-2.9
LOXL2	lysyl oxidase like 2	protein_coding	NP_002309.1	-2.9
CACNB1	calcium voltage-gated channel auxiliary subunit be	protein_coding	NP_000714.3;NP_954855.1;NP_95	-2.9
NUDT1	nudix hydrolase 1	protein_coding	NP_001354482.1;NP_001354483.1;	-2.9
SMARCD1	SWI/SNF related, matrix associated, actin depende	protein_coding	NP_003067.3;NP_620710.2;XP_00	-2.9
PLCE1-AS1	PLCE1 antisense RNA 1	lncRNA	.	-2.9
NPLOC4	NPL4 homolog, ubiquitin recognition factor	protein_coding	NP_001356627.1;NP_060391.2;XP_	-2.9
ACTR1A	actin related protein 1A	protein_coding	NP_005727.1	-2.9

RCAN3	RCAN family member 3	protein_coding	NP_001238906.1;NP_001238907.1;	-2.9
NOL9	nucleolar protein 9	protein_coding	NP_078930.4;XP_005263550.1;XP_	-2.9
SGF29	SAGA complex associated factor 29	protein_coding	NP_612423.1;XP_016878383.1	-2.9
UBQLN4	ubiquilin 4	protein_coding	NP_001291271.1;NP_064516.2;XP_	-2.9
POU3F2	POU class 3 homeobox 2	protein_coding	NP_005595.2	-2.9
RAP1GAP2	RAP1 GTPase activating protein 2	protein_coding	NP_001093868.1;NP_001316987.1;	-2.9
DPH1	diphthamide biosynthesis 1	protein_coding	NP_001333503.1;NP_001333504.1;	-2.9
EIF4H	eukaryotic translation initiation factor 4H	protein_coding	NP_071496.1;NP_114381.1	-2.9
CHRAC1	chromatin accessibility complex subunit 1	protein_coding	NP_059140.1	-2.9
IGHMBP2	immunoglobulin mu DNA binding protein 2	protein_coding	NP_002171.2;XP_005274031.1;XP_	-2.9
WWP2	WW domain containing E3 ubiquitin protein ligas	protein_coding	NP_001257382.1;NP_001257383.1;	-2.9
KLC4	kinesin light chain 4	protein_coding	NP_001275963.1;NP_001275964.1;	-2.9
CDK4	cyclin dependent kinase 4	protein_coding	NP_000066.1	-2.9
HOXB7	homeobox B7	protein_coding	NP_004493.3	-2.9
SYT17	synaptotagmin 17	protein_coding	NP_001295086.1;NP_001317438.1;	-2.9
PRMT7	protein arginine methyltransferase 7	protein_coding	NP_001171753.1;NP_001276947.1;	-2.9
ZFP36L2	ZFP36 ring finger protein like 2	protein_coding	NP_008818.3	-2.9
BCL7B	BAF chromatin remodeling complex subunit BCL	protein_coding	NP_001184173.1;NP_001287990.1;	-2.9
GORASP1	golgi reassembly stacking protein 1	protein_coding	NP_001265718.1;NP_001265719.1;	-2.9
ABCC6	ATP binding cassette subfamily C member 6	protein_coding	NP_001072996.1;NP_001162.5;NP_	-2.9
ILF3	interleukin enhancer binding factor 3	protein_coding	NP_001131145.1;NP_004507.2;NP_	-2.9
RCC1	regulator of chromosome condensation 1	protein_coding	NP_001041659.1;NP_001041660.1;	-2.9
TCN2	transcobalamin 2	protein_coding	NP_000346.2;NP_001171655.1	-3.0
WASH3P	WASP family homolog 3, pseudogene	ranscribed_pseudogen	.	-3.0
C3orf33	chromosome 3 open reading frame 33	protein_coding	NP_001295158.1;NP_775928.1;XP_	-3.0
HEBP1	heme binding protein 1	protein_coding	NP_057071.2	-3.0
USB1	U6 snRNA biogenesis phosphodiesterase 1	protein_coding	NP_001182231.1;NP_001191840.1;	-3.0
TGIF2	TGFB induced factor homeobox 2	protein_coding	NP_001186442.1;NP_001186443.1;	-3.0
RAB5IF	RAB5 interacting factor	protein_coding	NP_001186463.1;NP_001361107.1;	-3.0
MAN2A2	mannosidase alpha class 2A member 2	protein_coding	NP_001307906.1;NP_006113.2;XP_	-3.0
STK10	serine/threonine kinase 10	protein_coding	NP_005981.3;XP_016865277.1	-3.0
RELT	RELT TNF receptor	protein_coding	NP_116260.2;NP_689408.1;XP_01	-3.0
CCDC157	coiled-coil domain containing 157	protein_coding	NP_001017437.2;NP_001305263.2;	-3.0
ANAPC11	anaphase promoting complex subunit 11	protein_coding	NP_001002244.1;NP_001002245.1;	-3.0
DLG4	discs large MAGUK scaffold protein 4	protein_coding	NP_001122299.1;NP_001308003.1;	-3.0
LOC107984577	uncharacterized LOC107984577	lncRNA	.	-3.0
TPI1	triosephosphate isomerase 1	protein_coding	NP_000356.1;NP_001152759.1;NP_	-3.0
MAFG	MAF bZIP transcription factor G	protein_coding	NP_002350.1;NP_116100.2	-3.0
H2BC8	H2B clustered histone 8	protein_coding	NP_003509.1	-3.0
FN3KRP	fructosamine 3 kinase related protein	protein_coding	NP_078895.2;XP_024306716.1	-3.0
SLC45A4	solute carrier family 45 member 4	protein_coding	NP_001073900.1;NP_001273575.1;	-3.0
PRPF6	pre-mRNA processing factor 6	protein_coding	NP_036601.2;XP_006723832.1	-3.0
NOL3	nucleolar protein 3	protein_coding	NP_001171986.1;NP_001263236.1;	-3.0
GXYLT2	glucoside xylosyltransferase 2	protein_coding	NP_001073862.1;XP_011532369.1;	-3.0
RASSF4	Ras association domain family member 4	protein_coding	NP_114412.2;XP_005271890.1;XP_	-3.0
SARDH	sarcosine dehydrogenase	protein_coding	NP_001128179.1;NP_009032.2;XP_	-3.0
TP53INP2	tumor protein p53 inducible nuclear protein 2	protein_coding	NP_001316358.1;NP_001316359.1;	-3.0
CLPB	caseinolytic mitochondrial matrix peptidase chape	protein_coding	NP_001245321.1;NP_001245322.1;	-3.0
ARRDC2	arrestin domain containing 2	protein_coding	NP_001020775.1;NP_001273755.1;	-3.0
C1orf226	chromosome 1 open reading frame 226	protein_coding	NP_001078844.1;NP_001128712.1	-3.0
RNASEH2A	ribonuclease H2 subunit A	protein_coding	NP_006388.2	-3.0
MINDY1	MINDY lysine 48 deubiquitinase 1	protein_coding	NP_001035307.2;NP_001156730.2;	-3.0
RWDD4P2	RWD domain containing 4 pseudogene 2	pseudogene	.	-3.0
HDAC6	histone deacetylase 6	protein_coding	NP_001308154.1;NP_001308155.1;	-3.0
TPRG1L	tumor protein p63 regulated 1 like	protein_coding	NP_877429.2	-3.0
SYNE3	spectrin repeat containing nuclear envelope family	protein_coding	NP_001350621.1;NP_689805.3;XP_	-3.0
FLJ42969	uncharacterized LOC441374	lncRNA	.	-3.0
LOC101929124	uncharacterized LOC101929124	lncRNA	.	-3.0
PCDH18	protocadherin 18	protein_coding	NP_001287757.1;NP_061908.1;XP_	-3.0
ABCC1	ATP binding cassette subfamily C member 1	protein_coding	NP_004987.2;XP_011520799.1;XP_	-3.0
SUGP1	SURP and G-patch domain containing 1	protein_coding	NP_757386.2;XP_005260059.1;XP_	-3.0
BMP4	bone morphogenetic protein 4	protein_coding	NP_001193.2;NP_001334841.1;NP_	-3.0
KIRREL1	kirre like nephrin family adhesion molecule 1	protein_coding	NP_001273278.1;NP_060710.3;XP_	-3.0
CENPBD1	CENPB DNA-binding domain containing 1	protein_coding	NP_659476.2	-3.0
FANCG	FA complementation group G	protein_coding	NP_004620.1	-3.0
DCAKD	dephospho-CoA kinase domain containing	protein_coding	NP_001122103.1;NP_001275583.1;	-3.0
FAHD2B	fumarylacetoacetate hydrolase domain containing	protein_coding	NP_001307777.1;NP_001307778.1;	-3.0
UQCRFS1P1	ubiquinol-cytochrome c reductase, Rieske iron-sul	pseudogene	.	-3.0
MOCS1	molybdenum cofactor synthesis 1	protein_coding	NP_001068566.1;NP_001345458.1;	-3.0
FOXD2-AS1	FOXD2 adjacent opposite strand RNA 1	lncRNA	.	-3.0
DIO2	iodothyronine deiodinase 2	protein_coding	NP_000784.3;NP_001311391.2;NP_	-3.0

H2AC17	H2A clustered histone 17	protein_coding	NP_003505.1	-3.0
MEGF10	multiple EGF like domains 10	protein_coding	NP_001243474.1;NP_001295048.1;	-3.0
HTRA2	HtrA serine peptidase 2	protein_coding	NP_001308656.1;NP_001308657.1;	-3.0
GFOD2	glucose-fructose oxidoreductase domain containin	protein_coding	NP_001230579.1;NP_110446.3;XP_	-3.0
KRT7	keratin 7	protein_coding	NP_005547.3;XP_011536627.1;XP_	-3.0
ADO	2-aminoethanethiol dioxygenase	protein_coding	NP_116193.2	-3.0
PRMT1	protein arginine methyltransferase 1	protein_coding	NP_001193971.1;NP_001527.3;NP_	-3.0
F8A1	coagulation factor VIII associated 1	protein_coding	NP_036283.2	-3.0
PIGX	phosphatidylinositol glycan anchor biosynthesis cl	protein_coding	NP_001159776.1;NP_060331.3	-3.0
ABT1	activator of basal transcription 1	protein_coding	NP_037507.1	-3.0
ILRUN	inflammation and lipid regulator with UBA-like ar	protein_coding	NP_073595.2;NP_077270.1;XP_00	-3.0
FBXL8	F-box and leucine rich repeat protein 8	protein_coding	NP_060848.2	-3.0
PADI3	peptidyl arginine deiminase 3	protein_coding	NP_057317.2;XP_011539873.1;XP_	-3.0
TUSC2	tumor suppressor 2, mitochondrial calcium regulat	protein_coding	NP_009206.1	-3.0
H2AC7	H2A clustered histone 7	protein_coding	NP_066409.1	-3.0
XKR8	XK related 8	protein_coding	NP_060523.2;XP_011539981.1;XP_	-3.0
TNPO2	transportin 2	protein_coding	NP_001129667.1;NP_001129668.1;	-3.0
ARFRP1	ADP ribosylation factor related protein 1	protein_coding	NP_001128230.1;NP_001254473.1;	-3.0
MAP3K3	mitogen-activated protein kinase kinase kinase 3	protein_coding	NP_001317360.1;NP_001350697.1;	-3.0
CGAS	cyclic GMP-AMP synthase	protein_coding	NP_612450.2	-3.0
IFT27	intraflagellar transport 27	protein_coding	NP_001171172.1;NP_001349932.1;	-3.0
CTSA	cathepsin A	protein_coding	NP_000299.3;NP_001121167.1;NP_	-3.0
LOC105373748	uncharacterized LOC105373748	lncRNA	.	-3.0
CTBP1-DT	CTBP1 divergent transcript	lncRNA	.	-3.0
SHMT1	serine hydroxymethyltransferase 1	protein_coding	NP_001268715.1;NP_004160.3;NP_	-3.0
RANGRF	RAN guanine nucleotide release factor	protein_coding	NP_001171272.1;NP_001171273.1;	-3.0
LOC105374868	uncharacterized LOC105374868	lncRNA	.	-3.0
NR1H3	nuclear receptor subfamily 1 group H member 3	protein_coding	NP_001123573.1;NP_001123574.1;	-3.0
PKD1L2	polycystin 1 like 2 (gene/pseudogene)	protein_coding	NP_001070248.1;NP_001265352.1;	-3.0
POMT1	protein O-mannosyltransferase 1	protein_coding	NP_001070833.1;NP_001070834.1;	-3.0
C12orf43	chromosome 12 open reading frame 43	protein_coding	NP_001273120.1;NP_001273121.1;	-3.0
IFI30	IFI30 lysosomal thiol reductase	protein_coding	NP_006323.2	-3.0
LOC105369783	uncharacterized LOC105369783	lncRNA	.	-3.0
HELZ2	helicase with zinc finger 2	protein_coding	NP_001032412.2;NP_208384.3;XP_	-3.0
EMC8	ER membrane protein complex subunit 8	protein_coding	NP_001135760.1;NP_006058.1;XP_	-3.0
PPP5C	protein phosphatase 5 catalytic subunit	protein_coding	NP_001191213.1;NP_006238.1;XP_	-3.0
PRPF19	pre-mRNA processing factor 19	protein_coding	NP_055317.1	-3.0
LHX9	LIM homeobox 9	protein_coding	NP_001014434.1;NP_001357142.1;	-3.0
ARPC4	actin related protein 2/3 complex subunit 4	protein_coding	NP_001020130.1;NP_001020131.1;	-3.0
RNASEK	ribonuclease K	protein_coding	NP_001004333.3	-3.0
ARAP3	ArfGAP with RhoGAP domain, ankyrin repeat and	protein_coding	NP_071926.4;XP_005268554.1;XP_	-3.0
MYL6B	myosin light chain 6B	protein_coding	NP_001186558.1;NP_002466.1	-3.1
CDC37	cell division cycle 37, HSP90 cochaperone	protein_coding	NP_008996.1;XP_011525954.1	-3.1
EIF3FP3	eukaryotic translation initiation factor 3 subunit F	pseudogene	.	-3.1
GMPPA	GDP-mannose pyrophosphorylase A	protein_coding	NP_001361223.1;NP_001361224.1;	-3.1
CBR3	carbonyl reductase 3	protein_coding	NP_001227.1;XP_011528074.1	-3.1
RETREG3	reticulophagy regulator family member 3	protein_coding	NP_835227.1;XP_011522742.1	-3.1
KDM5C	lysine demethylase 5C	protein_coding	NP_001140174.1;NP_001269551.1;	-3.1
SSPN	sarcospan	protein_coding	NP_001129295.1;NP_005077.2;XP_	-3.1
NDUFAF8	NADH:ubiquinone oxidoreductase complex assem	protein_coding	NP_001079990.1;NP_001340331.1;	-3.1
SLC2A1	solute carrier family 2 member 1	protein_coding	NP_006507.2	-3.1
RELA-DT	RELA divergent transcript, transcript variant X3	lncRNA	.	-3.1
TUBB6	tubulin beta 6 class V	protein_coding	NP_001290453.1;NP_001290454.1;	-3.1
RRNAD1	ribosomal RNA adenine dimethylase domain conta	protein_coding	NP_001136032.1;NP_057081.3;XP_	-3.1
KIFC2	kinesin family member C2	protein_coding	NP_001356698.1;NP_665697.1;XP_	-3.1
EID2	EP300 interacting inhibitor of differentiation 2	protein_coding	NP_694964.3	-3.1
GREB1	growth regulating estrogen receptor binding 1	protein_coding	NP_055483.2;NP_149081.1;NP_68	-3.1
DHRS11	dehydrogenase/reductase 11	protein_coding	NP_077284.2;XP_005257715.1;XP_	-3.1
HPCAL1	hippocalcin like 1	protein_coding	NP_001245286.1;NP_001245287.1;	-3.1
ZNF341	zinc finger protein 341	protein_coding	NP_001269862.1;NP_001269864.1;	-3.1
SURF1	SURF1 cytochrome c oxidase assembly factor	protein_coding	NP_001267716.1;NP_003163.1;XP_	-3.1
RPS6KL1	ribosomal protein S6 kinase like 1	protein_coding	NP_001357181.1;NP_001357182.1;	-3.1
MRPS16	mitochondrial ribosomal protein S16	protein_coding	NP_057149.1	-3.1
SHOC1	shortage in chiasmata 1	protein_coding	NP_001074020.3;NP_001365140.1;	-3.1
SARM1	sterile alpha and TIR motif containing 1	protein_coding	NP_055892.2	-3.1
SNAPC4	small nuclear RNA activating complex polypeptid	protein_coding	NP_003077.2;XP_005266153.1;XP_	-3.1
COG8	component of oligomeric golgi complex 8	protein_coding	NP_001361800.1;NP_001366190.1;	-3.1
SMG9	SMG9 nonsense mediated mRNA decay factor	protein_coding	NP_061981.2;XP_005259114.1;XP_	-3.1
TCF19	transcription factor 19	protein_coding	NP_001070979.1;NP_001305837.1;	-3.1
CREB3	cAMP responsive element binding protein 3	protein_coding	NP_006359.3	-3.1
MAGEF1	MAGE family member F1	protein_coding	NP_071432.2	-3.1

DNAJB5	DnaJ heat shock protein family (Hsp40) member I	protein_coding	NP_001128476.3;NP_001128477.1;	-3.1
PPP1R13B	protein phosphatase 1 regulatory subunit 13B	protein_coding	NP_056131.2;XP_005267544.1;XP_	-3.1
THTPA	thiamine triphosphatase	protein_coding	NP_001119811.1;NP_001242991.1;	-3.1
SLC2A10	solute carrier family 2 member 10	protein_coding	NP_110404.1;XP_011527362.1;XP_	-3.1
LOC105374101	uncharacterized LOC105374101	lncRNA	.	-3.1
EMILIN2	elastin microfibril interfacer 2	protein_coding	NP_114437.2;XP_016881527.1	-3.1
RGS3	regulator of G protein signaling 3	protein_coding	NP_001263189.1;NP_001263190.1;	-3.1
FIBP	FGF1 intracellular binding protein	protein_coding	NP_004205.2;NP_942600.1;XP_02	-3.1
SENP3	SUMO specific peptidase 3	protein_coding	NP_056485.2	-3.1
ACOT2	acyl-CoA thioesterase 2	protein_coding	NP_001351106.1;NP_001351107.1;	-3.1
C10orf62	chromosome 10 open reading frame 62	protein_coding	NP_001009997.2	-3.1
SIN3B	SIN3 transcription regulator family member B	protein_coding	NP_001284524.1;NP_001284526.1;	-3.1
BUB1B-PAK6	BUB1B-PAK6 readthrough	protein_coding	NP_001122100.1;NP_001122101.1	-3.1
SIAH3	siah E3 ubiquitin protein ligase family member 3	protein_coding	NP_942146.2	-3.1
CALHM5	calcium homeostasis modulator family member 5	protein_coding	NP_714922.1	-3.1
LOC102724159	periodic tryptophan protein 2 homolog	protein_coding	NP_001355167.1	-3.1
MRAS	muscle RAS oncogene homolog	protein_coding	NP_001078518.1;NP_001239019.1;	-3.1
DENND2A	DENN domain containing 2A	protein_coding	NP_001304981.1;NP_001304982.1;	-3.1
SLC1A2	solute carrier family 1 member 2	protein_coding	NP_001182657.1;NP_001239581.1;	-3.1
CHCHD5	coiled-coil-helix-coiled-coil-helix domain contain	protein_coding	NP_001291282.1;NP_001291283.1;	-3.1
PMF1	polyamine modulated factor 1	protein_coding	NP_001186582.1;NP_001186583.1;	-3.1
TOGARAM2	TOG array regulator of axonemal microtubules 2	protein_coding	NP_001308467.1;NP_001308468.1;	-3.1
EIF1AXP1	EIF1AX pseudogene 1	pseudogene	.	-3.1
ZC3H12A	zinc finger CCCH-type containing 12A	protein_coding	NP_001310479.1;NP_001310480.1;	-3.1
H2BC7	H2B clustered histone 7	protein_coding	NP_003513.1	-3.1
VDAC2P5	VDAC2 pseudogene 5	pseudogene	.	-3.1
LOC100507507	uncharacterized LOC100507507	lncRNA	.	-3.1
NAA38	N-alpha-acetyltransferase 38, NatC auxiliary subu	protein_coding	NP_001307853.1;NP_001307854.1;	-3.1
PML	PML nuclear body scaffold	protein_coding	NP_002666.1;NP_150241.2;NP_15	-3.1
MZF1	myeloid zinc finger 1	protein_coding	NP_001253962.1;NP_003413.2;NP_	-3.1
IDH3G	isocitrate dehydrogenase (NAD(+)) 3 non-catalyti	protein_coding	NP_004126.1;NP_777358.1	-3.1
TMEM205	transmembrane protein 205	protein_coding	NP_001138888.1;NP_001308041.1;	-3.1
CRY2	cryptochrome circadian regulator 2	protein_coding	NP_001120929.1;NP_066940.3	-3.1
H2BC3	H2B clustered histone 3	protein_coding	NP_066406.1	-3.1
KDM7A-DT	KDM7A divergent transcript	lncRNA	.	-3.1
EHD4	EH domain containing 4	protein_coding	NP_644670.1	-3.1
EIF4EBP1	eukaryotic translation initiation factor 4E binding	protein_coding	NP_004086.1	-3.1
RNF227	ring finger protein 227	protein_coding	NP_001345628.1	-3.1
LOC102724397	uncharacterized LOC102724397, transcript varian	lncRNA	.	-3.1
EGR1	early growth response 1	protein_coding	NP_001955.1	-3.1
NCKIPSD	NCK interacting protein with SH3 domain	protein_coding	NP_057537.1;NP_909119.1;XP_01	-3.1
LOC105371763	uncharacterized LOC105371763	lncRNA	.	-3.1
TUFT1	tuftelin 1	protein_coding	NP_001119809.1;NP_001288246.1;	-3.1
CHST2	carbohydrate sulfotransferase 2	protein_coding	NP_004258.2	-3.2
TARS2	threonyl-tRNA synthetase 2, mitochondrial	protein_coding	NP_001258824.1;NP_001258825.1;	-3.2
RN7SL4P	RNA, 7SL, cytoplasmic 4, pseudogene	pseudogene	.	-3.2
FANCF	FA complementation group F	protein_coding	NP_073562.1	-3.2
NLE1	notchless homolog 1	protein_coding	NP_001014445.1;NP_060566.2;XP_	-3.2
MAGED1	MAGE family member D1	protein_coding	NP_001005332.1;NP_001005333.1;	-3.2
WDR6	WD repeat domain 6	protein_coding	NP_001307475.1;NP_001307476.1;	-3.2
PDLIM1	PDZ and LIM domain 1	protein_coding	NP_066272.1;XP_011538632.1	-3.2
CSNK1D	casein kinase 1 delta	protein_coding	NP_001350678.1;NP_001884.2;NP_	-3.2
LOXL4	lysyl oxidase like 4	protein_coding	NP_115587.6;XP_005270273.1;XP_	-3.2
OSER1-DT	OSER1 divergent transcript	lncRNA	.	-3.2
MGLL	monoglyceride lipase	protein_coding	NP_001003794.1;NP_001243514.1;	-3.2
TPRA1	transmembrane protein adipocyte associated 1	protein_coding	NP_001129525.1;NP_001136118.1;	-3.2
PIGS	phosphatidylinositol glycan anchor biosynthesis cl	protein_coding	NP_149975.1	-3.2
GCN1	GCN1 activator of EIF2AK4	protein_coding	NP_006827.1	-3.2
PGGHG	protein-glucosylgalactosylhydroxylysine glucosid	protein_coding	NP_079368.3;XP_011518685.1;XP_	-3.2
H1-0	H1.0 linker histone	protein_coding	NP_005309.1	-3.2
MRPL57	mitochondrial ribosomal protein L57	protein_coding	NP_076931.1;XP_016876229.1	-3.2
HMGN2P17	high mobility group nucleosomal binding domain	pseudogene	.	-3.2
BRD1	bromodomain containing 1	protein_coding	NP_001291737.1;NP_001291738.1;	-3.2
THPO	thrombopoietin	protein_coding	NP_000451.1;NP_001171068.1;NP_	-3.2
FAXC	failed axon connections homolog, metaxin like GS	protein_coding	NP_001333459.1;NP_001333460.1;	-3.2
AP1M2	adaptor related protein complex 1 subunit mu 2	protein_coding	NP_001287816.1;NP_005489.2;XP_	-3.2
OAF	out at first homolog	protein_coding	NP_848602.1	-3.2
SNRPN	small nuclear ribonucleoprotein polypeptide N	protein_coding	NP_001336383.1;NP_001336384.1;	-3.2
DYSF	dysferlin	protein_coding	NP_001123927.1;NP_001124448.1;	-3.2
FAM174A	family with sequence similarity 174 member A	protein_coding	NP_940909.1;XP_006714663.1	-3.2
TMEM219	transmembrane protein 219	protein_coding	NP_001077082.1;NP_001356617.1;	-3.2

STARD8	StAR related lipid transfer domain containing 8	protein_coding	NP_001135975.1;NP_001135976.1;	-3.2
ADD1	adducin 1	protein_coding	NP_001110.2;NP_001273574.1;NP_	-3.2
LRPAP1	LDL receptor related protein associated protein 1	protein_coding	NP_002328.1	-3.2
NSMCE3	NSE3 homolog, SMC5-SMC6 complex componer	protein_coding	NP_619649.1	-3.2
LRATD1	LRAT domain containing 1	protein_coding	NP_001356293.1;NP_660158.2	-3.2
TLN1	talin 1	protein_coding	NP_006280.3	-3.2
GPLD1	glycosylphosphatidylinositol specific phospholipa:	protein_coding	NP_001494.2;XP_016866242.1;XP_	-3.2
PRR11	proline rich 11	protein_coding	NP_060774.2;XP_024306596.1	-3.2
FAM13C	family with sequence similarity 13 member C	protein_coding	NP_001001971.1;NP_001137245.1;	-3.2
FBLN5	fibulin 5	protein_coding	NP_006320.2;XP_005267324.1;XP_	-3.2
RIOX1	ribosomal oxygenase 1	protein_coding	NP_078920.2	-3.2
AGBL5	ATP/GTP binding protein like 5	protein_coding	NP_001030584.1;NP_068603.4;XP_	-3.2
ECM1	extracellular matrix protein 1	protein_coding	NP_001189787.1;NP_004416.2;NP_	-3.2
HCG11	HLA complex group 11	lncRNA	.	-3.2
LINC00665	long intergenic non-protein coding RNA 665	lncRNA	.	-3.2
DUSP8P5	dual specificity phosphatase 8 pseudogene 5	pseudogene	.	-3.2
DAXX	death domain associated protein	protein_coding	NP_001135441.1;NP_001135442.1;	-3.2
LOC107985497	uncharacterized LOC107985497	lncRNA	.	-3.2
HLA-E	major histocompatibility complex, class I, E	protein_coding	NP_005507.3;XP_016866296.1;XP_	-3.2
LOC642361	uncharacterized LOC642361	lncRNA	.	-3.2
TOMM40L	translocase of outer mitochondrial membrane 40 li	protein_coding	NP_001273302.1;NP_001273303.1;	-3.2
RTL6	retrotransposon Gag like 6	protein_coding	NP_115663.2	-3.2
WDR83	WD repeat domain 83	protein_coding	NP_001093207.1;NP_115708.1	-3.2
MEIS3P1	Meis homeobox 3 pseudogene 1	ranscribed_pseudogen	.	-3.2
WDR55	WD repeat domain 55	protein_coding	NP_060176.2;XP_005268526.1;XP_	-3.2
ERI3	ERI1 exoribonuclease family member 3	protein_coding	NP_001288627.1;NP_001288628.1;	-3.2
H4C15	H4 clustered histone 15	protein_coding	NP_001029249.1;XP_024307476.1;	-3.2
BSN	bassoon presynaptic cytomatrix protein	protein_coding	NP_003449.2	-3.2
PNRC1	proline rich nuclear receptor coactivator 1	protein_coding	NP_006804.1	-3.2
SFXN3	sideroflexin 3	protein_coding	NP_112233.2	-3.2
L3MBTL2	L3MBTL histone methyl-lysine binding protein 2	protein_coding	NP_113676.2;XP_011528722.1;XP_	-3.2
TBX2-AS1	TBX2 antisense RNA 1	lncRNA	.	-3.2
PPAN	peter pan homolog	protein_coding	NP_001333068.1;NP_001333070.1;	-3.2
GUCY1A2	guanylate cyclase 1 soluble subunit alpha 2	protein_coding	NP_000846.1;NP_001243353.1	-3.2
H2BC9	H2B clustered histone 9	protein_coding	NP_003515.1	-3.2
DIP2C	disco interacting protein 2 homolog C	protein_coding	NP_055789.1;XP_005252483.1;XP_	-3.2
CCDC167	coiled-coil domain containing 167	protein_coding	NP_612502.1	-3.2
FRS3	fibroblast growth factor receptor substrate 3	protein_coding	NP_006644.1;XP_011512556.1;XP_	-3.2
ATP6V0E2-AS1	ATP6V0E2 antisense RNA 1	lncRNA	.	-3.2
PRKRIP1	PRKR interacting protein 1	protein_coding	NP_078929.1	-3.2
TXLNA	taxilin alpha	protein_coding	NP_001363786.1;NP_001363787.1;	-3.2
PMPCA	peptidase, mitochondrial processing subunit alpha	protein_coding	NP_001269873.1;NP_001269875.1;	-3.2
ACSF3	acyl-CoA synthetase family member 3	protein_coding	NP_001120686.1;NP_001230208.1;	-3.2
LOC100652758	uncharacterized LOC100652758	lncRNA	.	-3.2
B4GALT3	beta-1,4-galactosyltransferase 3	protein_coding	NP_001186802.1;NP_001186803.1;	-3.2
COQ8A	coenzyme Q8A	protein_coding	NP_064632.2;XP_005273258.1;XP_	-3.2
ACADVL	acyl-CoA dehydrogenase very long chain	protein_coding	NP_000009.1;NP_001029031.1;NP_	-3.2
H1-9P	H1.9 linker histone, pseudogene	ranscribed_pseudogen	.	-3.2
NAXE	NAD(P)HX epimerase	protein_coding	NP_658985.2;XP_016855808.1	-3.2
TBC1D2	TBC1 domain family member 2	protein_coding	NP_001254500.1;NP_001254501.1;	-3.2
DXO	decapping exoribonuclease	protein_coding	NP_001358134.1;NP_001358135.1;	-3.2
PRKAR2A-AS1	PRKAR2A antisense RNA 1	lncRNA	.	-3.2
LOC105376917	uncharacterized LOC105376917	lncRNA	.	-3.2
ARSL	arylsulfatase L	protein_coding	NP_000038.2;NP_001269557.1;NP_	-3.3
TANGO2	transport and golgi organization 2 homolog	protein_coding	NP_001270035.1;NP_001270045.1;	-3.3
CPT2	carnitine palmitoyltransferase 2	protein_coding	NP_000089.1;NP_001317518.1	-3.3
LOC728688	ubiquitin like with PHD and ring finger domains 1	pseudogene	.	-3.3
JCAD	junctional cadherin 5 associated	protein_coding	NP_001336930.1;NP_001336950.1;	-3.3
ARFGAP2	ADP ribosylation factor GTPase activating proteir	protein_coding	NP_001229761.1;NP_115765.2;XP_	-3.3
TMEM185AP1	transmembrane protein 185A pseudogene 1	pseudogene	.	-3.3
HYAL1	hyaluronidase 1	protein_coding	NP_149349.2;NP_695013.1;NP_69:	-3.3
RAET1G	retinoic acid early transcript 1G	protein_coding	NP_001001788.2;XP_011534102.1;	-3.3
NDE1	nudE neurodevelopment protein 1	protein_coding	NP_001137451.1;NP_060138.1;XP_	-3.3
INPP5A	inositol polyphosphate-5-phosphatase A	protein_coding	NP_001307971.1;NP_005530.3;XP_	-3.3
LOXL1-AS1	LOXL1 antisense RNA 1	lncRNA	.	-3.3
DDX39A	DExD-box helicase 39A	protein_coding	NP_005795.2;XP_006722669.1;XP_	-3.3
ALG1	ALG1 chitobiosyldiphosphodolichol beta-mannos	protein_coding	NP_001317433.1;NP_061982.3;XP_	-3.3
HDDC3	HD domain containing 3	protein_coding	NP_001273380.1;NP_940929.1	-3.3
FAHD1	fumarylacetoacetate hydrolase domain containing	protein_coding	NP_001018114.1;NP_001135870.1;	-3.3
SMUG1	single-strand-selective monofunctional uracil-DN	protein_coding	NP_001230716.1;NP_001230717.1;	-3.3
LOC100130992	uncharacterized LOC100130992	lncRNA	.	-3.3

RPS9	ribosomal protein S9	protein_coding	NP_001004.2;NP_001308630.1;NP_	-3.3
PXDN	peroxidasin	protein_coding	NP_036425.1;XP_005264764.1;XP_	-3.3
ENGASE	endo-beta-N-acetylglucosaminidase	protein_coding	NP_001036038.1;XP_006722080.1;	-3.3
GPR4	G protein-coupled receptor 4	protein_coding	NP_005273.1;XP_016882096.1;XP_	-3.3
MFGE8	milk fat globule EGF and factor V/VIII domain co	protein_coding	NP_001108086.1;NP_001297248.1;	-3.3
SNORA32	small nucleolar RNA, H/ACA box 32	snoRNA	.	-3.3
MAN2B2	mannosidase alpha class 2B member 2	protein_coding	NP_001278967.1;NP_056089.1	-3.3
RGP1	RGP1 homolog, RAB6A GEF complex partner 1	protein_coding	NP_001073965.2	-3.3
CBS	cystathionine beta-synthase	protein_coding	NP_000062.1;NP_001171479.1;NP_	-3.3
STK35	serine/threonine kinase 35	protein_coding	NP_543026.2;XP_011527476.1	-3.3
SEC16A	SEC16 homolog A, endoplasmic reticulum export	protein_coding	NP_001263347.1;NP_055681.1;XP_	-3.3
TRIM16	tripartite motif containing 16	protein_coding	NP_001335048.1;NP_001335049.1;	-3.3
HMGN2P2	high mobility group nucleosomal binding domain	pseudogene	.	-3.3
ZNF71	zinc finger protein 71	protein_coding	NP_001357143.1;NP_001357144.1;	-3.3
RN7SKP80	RN7SK pseudogene 80	pseudogene	.	-3.3
H2AC6	H2A clustered histone 6	protein_coding	NP_003503.1	-3.3
XXYLT1	xyloside xylosyltransferase 1	protein_coding	NP_001294998.1;NP_689744.3;XP_	-3.3
DLX1	distal-less homeobox 1	protein_coding	NP_001033582.1;NP_835221.2	-3.3
H2BU1	H2B.U histone 1	protein_coding	NP_778225.1	-3.3
LOC107987082	.	pseudogene	.	-3.3
DDIT4L	DNA damage inducible transcript 4 like	protein_coding	NP_660287.1	-3.3
PCDHGB7	protocadherin gamma subfamily B, 7	protein_coding	NP_061750.1;NP_115272.1	-3.3
RBCK1	RANBP2-type and C3HC4-type zinc finger contai	protein_coding	NP_001310885.1;NP_001310887.1;	-3.3
PLD2	phospholipase D2	protein_coding	NP_001230037.1;NP_002654.3;XP_	-3.3
CEP68	centrosomal protein 68	protein_coding	NP_001306029.1;NP_001306030.1;	-3.3
CKAP4	cytoskeleton associated protein 4	protein_coding	NP_006816.2	-3.3
GTPBP1	GTP binding protein 1	protein_coding	NP_004277.2;XP_011528839.1;XP_	-3.3
B3GNT9	UDP-GlcNAc:betaGal beta-1,3-N-acetylglucosam	protein_coding	NP_171608.2	-3.3
ZMIZ2	zinc finger MIZ-type containing 2	protein_coding	NP_001287888.1;NP_113637.3;NP_	-3.3
STING1	stimulator of interferon response cGAMP interacto	protein_coding	NP_001288667.1;NP_001354187.1;	-3.3
KIFC1	kinesin family member C1	protein_coding	NP_002254.2;XP_011512887.1;XP_	-3.3
SMARCD2	SWI/SNF related, matrix associated, actin depende	protein_coding	NP_001091896.1;NP_001317368.1;	-3.3
VARS2	valyl-tRNA synthetase 2, mitochondrial	protein_coding	NP_001161205.1;NP_001161206.1;	-3.3
TMEM177	transmembrane protein 177	protein_coding	NP_001098668.1;NP_001098669.1;	-3.3
CEBPZOS	CEBPZ opposite strand	protein_coding	NP_001309302.1;NP_001309303.1;	-3.3
MTLN	mitoregulin	protein_coding	NP_001342180.1;XP_024308519.1	-3.3
ACAA1	acetyl-CoA acyltransferase 1	protein_coding	NP_001123882.1;NP_001598.1;XP_	-3.3
TGFB3	transforming growth factor beta 3	protein_coding	NP_001316867.1;NP_001316868.1;	-3.3
ITGA3	integrin subunit alpha 3	protein_coding	NP_002195.1;XP_005257365.1	-3.3
H2BS1	H2B.S histone 1	protein_coding	NP_059141.1	-3.3
PWP2	PWP2 small subunit processome component	protein_coding	NP_005040.2;XP_011527969.1	-3.3
RRP1	ribosomal RNA processing 1	protein_coding	NP_003674.1;XP_016883974.1;XP_	-3.3
MDC1	mediator of DNA damage checkpoint 1	protein_coding	NP_055456.2;XP_005249549.1;XP_	-3.3
PEF1	penta-EF-hand domain containing 1	protein_coding	NP_001346580.1;NP_036524.1;XP_	-3.3
PPP6R2	protein phosphatase 6 regulatory subunit 2	protein_coding	NP_001229827.1;NP_001229828.1;	-3.3
IBA57	iron-sulfur cluster assembly factor IBA57	protein_coding	NP_001010867.1;NP_001297256.1	-3.3
ALG12	ALG12 alpha-1,6-mannosyltransferase	protein_coding	NP_077010.1;XP_011528673.1;XP_	-3.3
E2F4	E2F transcription factor 4	protein_coding	NP_001941.2	-3.3
SSBP3	single stranded DNA binding protein 3	protein_coding	NP_001009955.1;NP_060540.2;NP_	-3.3
HOXA5	homeobox A5	protein_coding	NP_061975.2	-3.3
PRDX5	peroxiredoxin 5	protein_coding	NP_001345440.2;NP_001345445.1;	-3.3
TRIM26	tripartite motif containing 26	protein_coding	NP_001229712.1;NP_003440.1;XP_	-3.3
PGPEP1	pyroglutamyl-peptidase I	protein_coding	NP_001287856.1;NP_001295295.1;	-3.3
CDCA5	cell division cycle associated 5	protein_coding	NP_542399.1;XP_005273790.1;XP_	-3.3
AMOT	angiomotin	protein_coding	NP_001106962.1;NP_573572.1;XP_	-3.3
CROCC	ciliary rootlet coiled-coil, rootletin	protein_coding	NP_055490.4;XP_006711121.1;XP_	-3.3
TBCD	tubulin folding cofactor D	protein_coding	NP_005984.3;XP_005256453.1;XP_	-3.3
OGG1	8-oxoguanine DNA glycosylase	protein_coding	NP_001341577.1;NP_001341578.1;	-3.3
ADCY3	adenylate cyclase 3	protein_coding	NP_001307542.1;NP_001364057.1;	-3.3
TTPA	alpha tocopherol transfer protein	protein_coding	NP_000361.1;XP_006716531.1	-3.3
MYO9B	myosin IXB	protein_coding	NP_001123537.1;NP_004136.2	-3.3
CDH6	cadherin 6	protein_coding	NP_001349364.1;NP_004923.1;XP_	-3.3
STEAP3	STEAP3 metalloreductase	protein_coding	NP_001008410.1;NP_060704.2;NP_	-3.3
LINC00638	long intergenic non-protein coding RNA 638	lncRNA	.	-3.3
TMOD1	tropomodulin 1	protein_coding	NP_001159588.1;NP_003266.1;XP_	-3.3
KDM8	lysine demethylase 8	protein_coding	NP_001138820.1;NP_079049.2;XP_	-3.4
LOC107986558	uncharacterized LOC107986558	lncRNA	.	-3.4
IRS1	insulin receptor substrate 1	protein_coding	NP_005535.1	-3.4
SLC25A35	solute carrier family 25 member 35	protein_coding	NP_001307799.1;NP_001307800.1;	-3.4
FCSK	fucose kinase	protein_coding	NP_659496.2;XP_006721225.1;XP_	-3.4
CDKN1B	cyclin dependent kinase inhibitor 1B	protein_coding	NP_004055.1	-3.4

TXN2	thioredoxin 2	protein_coding	NP_036605.2;XP_006724289.1;XP_	-3.4
SH3BGRL3	SH3 domain binding glutamate rich protein like 3	protein_coding	NP_112576.1	-3.4
VAC14	VAC14 component of PIKFYVE complex	protein_coding	NP_001338086.1;NP_060522.3;XP_	-3.4
SHMT2	serine hydroxymethyltransferase 2	protein_coding	NP_001159828.1;NP_001159829.1;	-3.4
HNRNPA1P38	heterogeneous nuclear ribonucleoprotein A1pseud	pseudogene	.	-3.4
MRPS24	mitochondrial ribosomal protein S24	protein_coding	NP_114403.1	-3.4
RBM15B	RNA binding motif protein 15B	protein_coding	NP_037418.3	-3.4
KCNH7	potassium voltage-gated channel subfamily H mer	protein_coding	NP_150375.2;NP_775185.1;XP_01	-3.4
ACP2	acid phosphatase 2, lysosomal	protein_coding	NP_001289418.1;NP_001289419.1;	-3.4
CENPT	centromere protein T	protein_coding	NP_079358.3;XP_011521647.1;XP_	-3.4
RALY	RALY heterogeneous nuclear ribonucleoprotein	protein_coding	NP_031393.2;NP_057951.1;XP_00	-3.4
ABHD12	abhydrolase domain containing 12, lysophospholip	protein_coding	NP_001035937.1;NP_056415.1;XP_	-3.4
GLIPR2	GLI pathogenesis related 2	protein_coding	NP_001273939.1;NP_001273940.1;	-3.4
JPT1	Jupiter microtubule associated homolog 1	protein_coding	NP_001002032.1;NP_001002033.1;	-3.4
RRP12	ribosomal RNA processing 12 homolog	protein_coding	NP_001138586.1;NP_001271266.1;	-3.4
REXO4	REX4 homolog, 3'-5' exonuclease	protein_coding	NP_001266278.1;NP_001266279.1;	-3.4
CHRNA7	cholinergic receptor nicotinic alpha 7 subunit	protein_coding	NP_000737.1;NP_001177384.1;XP_	-3.4
EXOSC6	exosome component 6	protein_coding	NP_478126.1	-3.4
AFAP1L2	actin filament associated protein 1 like 2	protein_coding	NP_001001936.1;NP_001274753.1;	-3.4
NUDC	nuclear distribution C, dynein complex regulator	protein_coding	NP_006591.1;XP_011538832.1;XP_	-3.4
CCS	copper chaperone for superoxide dismutase	protein_coding	NP_005116.1	-3.4
HINT2	histidine triad nucleotide binding protein 2	protein_coding	NP_115982.1;XP_024303470.1;XP_	-3.4
NDRG1	N-myc downstream regulated 1	protein_coding	NP_001128714.1;NP_001245361.1;	-3.4
HOXB2	homeobox B2	protein_coding	NP_002136.1;XP_005257332.1	-3.4
PRICKLE1	prickle planar cell polarity protein 1	protein_coding	NP_001138353.1;NP_001138354.1;	-3.4
SLC9A3-AS1	SLC9A3 antisense RNA 1	lncRNA	.	-3.4
LOC107984433	uncharacterized LOC107984433	lncRNA	.	-3.4
NONOP1	non-POU domain containing, octamer-binding pse	pseudogene	.	-3.4
NUTM2A	NUT family member 2A	protein_coding	NP_001092808.1;XP_011538424.1;	-3.4
ADM	adrenomedullin	protein_coding	NP_001115.1	-3.4
TRIM3	tripartite motif containing 3	protein_coding	NP_001234935.1;NP_001234936.1;	-3.4
LRSAM1	leucine rich repeat and sterile alpha motif containi	protein_coding	NP_001005373.1;NP_001005374.1;	-3.4
SNURF	SNRPN upstream reading frame	protein_coding	NP_005669.2;NP_073715.1	-3.4
BRI3BP	BRI3 binding protein	protein_coding	NP_542193.3;XP_011536242.1	-3.4
CAMKK1	calcium/calmodulin dependent protein kinase kina	protein_coding	NP_115670.1;NP_757343.2;NP_75	-3.4
EHD3	EH domain containing 3	protein_coding	NP_055415.1;XP_011531108.1	-3.4
DPF2	double PHD fingers 2	protein_coding	NP_001317237.1;NP_006259.1;XP_	-3.4
TMBIM1	transmembrane BAX inhibitor motif containing 1	protein_coding	NP_001308356.1;NP_001308357.1;	-3.4
LOC100505501	uncharacterized LOC100505501	lncRNA	.	-3.4
FRAT2	FRAT regulator of WNT signaling pathway 2	protein_coding	NP_036215.1	-3.4
MCAT	malonyl-CoA-acyl carrier protein transacylase	protein_coding	NP_055322.1;NP_775738.3;XP_01	-3.4
DNAJB12	DnaJ heat shock protein family (Hsp40) member B	protein_coding	NP_001002762.3;NP_001352009.1;	-3.4
ABCA7	ATP binding cassette subfamily A member 7	protein_coding	NP_061985.2;XP_006722679.1;XP_	-3.4
S100A3	S100 calcium binding protein A3	protein_coding	NP_002951.1	-3.4
YIPF3	Yip1 domain family member 3	protein_coding	NP_056203.2	-3.4
ANGPTL2	angiopoietin like 2	protein_coding	NP_036230.1;XP_006717093.1	-3.4
ALDH5A1	aldehyde dehydrogenase 5 family member A1	protein_coding	NP_001071.1;NP_001355883.1;NP_	-3.4
TRIM52-AS1	TRIM52 antisense RNA 1 (head to head)	lncRNA	.	-3.4
LINP1	lncRNA in non-homologous end joining pathway	lncRNA	.	-3.5
TUBB	tubulin beta class I	protein_coding	NP_001280141.1;NP_001280142.1;	-3.5
CDAN1	codanin 1	protein_coding	NP_612486.2;XP_005254233.1;XP_	-3.5
TTC7A	tetratricopeptide repeat domain 7A	protein_coding	NP_001275880.1;NP_001275882.1;	-3.5
CPSF4	cleavage and polyadenylation specific factor 4	protein_coding	NP_001075028.1;NP_001305089.1;	-3.5
C19orf47	chromosome 19 open reading frame 47	protein_coding	NP_001243369.1;NP_001243370.1;	-3.5
GTF3C1	general transcription factor IIIC subunit 1	protein_coding	NP_001273171.1;NP_001511.2;XP_	-3.5
B4GALNT1	beta-1,4-N-acetyl-galactosaminyltransferase 1	protein_coding	NP_001263397.1;NP_001263398.1;	-3.5
TPD52L2	TPD52 like 2	protein_coding	NP_001230820.1;NP_001230821.1;	-3.5
BCL7A	BAF chromatin remodeling complex subunit BCL	protein_coding	NP_001019979.1;NP_066273.1	-3.5
LOC105379599	uncharacterized LOC105379599, transcript varian	lncRNA	.	-3.5
NAIF1	nuclear apoptosis inducing factor 1	protein_coding	NP_931045.1	-3.5
DBNL	drebrin like	protein_coding	NP_001014436.1;NP_001116428.1;	-3.5
SFXN5	sideroflexin 5	protein_coding	NP_001317329.1;NP_001317330.1;	-3.5
SLC41A3	solute carrier family 41 member 3	protein_coding	NP_001008485.2;NP_001008486.2;	-3.5
RPL18AP6	ribosomal protein L18a pseudogene 6	pseudogene	.	-3.5
RN7SL832P	RNA, 7SL, cytoplasmic 832, pseudogene	ranscribed_pseudogen	.	-3.5
LENG1	leukocyte receptor cluster member 1	protein_coding	NP_077292.2	-3.5
VGLL2	vestigial like family member 2	protein_coding	NP_703154.1;NP_872586.1;XP_00	-3.5
MAU2	MAU2 sister chromatid cohesion factor	protein_coding	NP_056144.3;XP_005259894.2;XP_	-3.5
MRPL37	mitochondrial ribosomal protein L37	protein_coding	NP_001317531.1;NP_057575.2	-3.5
SLC29A1	solute carrier family 29 member 1 (Augustine bloc	protein_coding	NP_001071643.1;NP_001071645.1;	-3.5
GNA12	G protein subunit alpha 12	protein_coding	NP_001269369.1;NP_001269370.1;	-3.5

NUTM2B	NUT family member 2B	protein_coding	NP_001265424.1	-3.5
UBXN1	UBX domain protein 1	protein_coding	NP_001273006.1;NP_001273007.1;	-3.5
EIF3FP2	eukaryotic translation initiation factor 3 subunit F	pseudogene	.	-3.5
C9orf40	chromosome 9 open reading frame 40	protein_coding	NP_060468.2	-3.5
DMAP1	DNA methyltransferase 1 associated protein 1	protein_coding	NP_001029195.1;NP_001029196.1;	-3.5
ABR	ABR activator of RhoGEF and GTPase	protein_coding	NP_001083.2;NP_001153218.1;NP_	-3.5
GGA1	golgi associated, gamma adaptin ear containing, A	protein_coding	NP_001001560.1;NP_001166158.1;	-3.5
EPAS1	endothelial PAS domain protein 1	protein_coding	NP_001421.2;XP_011531000.1	-3.5
EXTL3	exostosin like glycosyltransferase 3	protein_coding	NP_001431.1;XP_011542742.1;XP_	-3.5
NANOS1	nanos C2HC-type zinc finger 1	protein_coding	NP_955631.1	-3.5
TRAP1	TNF receptor associated protein 1	protein_coding	NP_001258978.1;NP_057376.2;XP_	-3.5
WDR5	WD repeat domain 5	protein_coding	NP_060058.1;NP_438172.1;XP_00	-3.5
UBOX5	U-box domain containing 5	protein_coding	NP_001254513.1;NP_055763.1;NP_	-3.5
RTL10	retrotransposon Gag like 10	protein_coding	NP_078903.3	-3.5
SUMF2	sulfatase modifying factor 2	protein_coding	NP_001035933.3;NP_001035934.3;	-3.5
C6orf226	chromosome 6 open reading frame 226	protein_coding	NP_001008739.1	-3.5
JRK	Jrk helix-turn-helix protein	protein_coding	NP_001070995.2;NP_001266281.1;	-3.5
ZMIZ1	zinc finger MIZ-type containing 1	protein_coding	NP_065071.1;XP_005270044.1;XP_	-3.5
GATA3	GATA binding protein 3	protein_coding	NP_001002295.1;NP_002042.1;XP_	-3.5
DOC2A	double C2 domain alpha	protein_coding	NP_001268991.1;NP_001268992.1;	-3.5
NUMA1	nuclear mitotic apparatus protein 1	protein_coding	NP_001273490.1;NP_006176.2;XP_	-3.5
TEX261	testis expressed 261	protein_coding	NP_653183.2	-3.5
PNMA1	PNMA family member 1	protein_coding	NP_006020.4	-3.5
DHRS4L2	dehydrogenase/reductase 4 like 2	protein_coding	NP_001180564.1;NP_001180565.1;	-3.5
TBCC	tubulin folding cofactor C	protein_coding	NP_003183.2	-3.5
LOC105372709	uncharacterized LOC105372709	lncRNA	.	-3.5
NT5C3B	5'-nucleotidase, cytosolic IIIB	protein_coding	NP_443167.4;XP_006721732.1;XP_	-3.5
LOC101929691	uncharacterized LOC101929691, transcript varian	lncRNA	.	-3.5
PHF23	PHD finger protein 23	protein_coding	NP_001271446.1;NP_001271447.1;	-3.5
ANKRD13D	ankyrin repeat domain 13D	protein_coding	NP_001334830.1;NP_997237.2;XP_	-3.5
FLVCR2	FLVCR heme transporter 2	protein_coding	NP_001182212.1;NP_060261.2	-3.5
LINC01123	long intergenic non-protein coding RNA 1123	lncRNA	.	-3.5
PRICKLE4	prickle planar cell polarity protein 4	protein_coding	NP_037529.3	-3.5
EIF6	eukaryotic translation initiation factor 6	protein_coding	NP_001254739.1;NP_002203.1;NP_	-3.5
TCF25	transcription factor 25	protein_coding	NP_055787.1;XP_005256354.1;XP_	-3.5
MYDGF	myeloid derived growth factor	protein_coding	NP_061980.1;XP_016882476.1	-3.5
LSM14B	LSM family member 14B	protein_coding	NP_653304.2;XP_005260359.1;XP_	-3.5
SLC29A2	solute carrier family 29 member 2	protein_coding	NP_001287797.1;NP_001287798.1;	-3.5
PRRC2B	proline rich coiled-coil 2B	protein_coding	NP_037450.2	-3.5
CUL7	cullin 7	protein_coding	NP_001161842.2;NP_001361801.1;	-3.5
SPRYD3	SPRY domain containing 3	protein_coding	NP_116229.1	-3.5
CBSL	cystathionine beta-synthase like	protein_coding	NP_001308002.1;NP_001340935.1;	-3.5
SAP30	Sin3A associated protein 30	protein_coding	NP_003855.1	-3.6
H4C13	H4 clustered histone 13	protein_coding	NP_003537.1	-3.6
HSD17B10	hydroxysteroid 17-beta dehydrogenase 10	protein_coding	NP_001032900.1;NP_004484.1	-3.6
CITED2	Cbp/p300 interacting transactivator with Glu/Asp	protein_coding	NP_001161860.1;NP_001161861.2;	-3.6
LOC105372275	uncharacterized LOC105372275	lncRNA	.	-3.6
LRRC46	leucine rich repeat containing 46	protein_coding	NP_219481.1;XP_005257832.1	-3.6
LOC102724334	histone H2B type F-S-like	protein_coding	XP_006723981.1	-3.6
ALKBH6	alkB homolog 6	protein_coding	NP_001284630.1;NP_116267.3;NP_	-3.6
KCNE1B	potassium voltage-gated channel subfamily E regu	protein_coding	NP_001316994.1;NP_001356798.1;	-3.6
SNX33	sorting nexin 33	protein_coding	NP_001305075.1;NP_695003.1	-3.6
HSD17B14	hydroxysteroid 17-beta dehydrogenase 14	protein_coding	NP_057330.2;XP_005259026.1;XP_	-3.6
H2AJ	H2A.J histone	protein_coding	NP_808760.1	-3.6
RAB32	RAB32, member RAS oncogene family	protein_coding	NP_006825.1	-3.6
RPLP1P6	ribosomal protein lateral stalk subunit P1 pseudog	pseudogene	.	-3.6
SCNN1A	sodium channel epithelial 1 subunit alpha	protein_coding	NP_001029.1;NP_001153047.1;NP_	-3.6
YKT6	YKT6 v-SNARE homolog	protein_coding	NP_001350607.1;NP_006546.1;XP_	-3.6
GALNS	galactosamine (N-acetyl)-6-sulfatase	protein_coding	NP_000503.1;NP_001310472.1;NP_	-3.6
SMYD5	SMYD family member 5	protein_coding	NP_006053.2	-3.6
TMEM19	transmembrane protein 19	protein_coding	NP_060749.2	-3.6
PNPO	pyridoxamine 5'-phosphate oxidase	protein_coding	NP_060599.1;XP_005257557.1;XP_	-3.6
LOC102723591	uncharacterized LOC102723591, transcript varian	lncRNA	.	-3.6
SNRPA	small nuclear ribonucleoprotein polypeptide A	protein_coding	NP_004587.1	-3.6
SH3RF3	SH3 domain containing ring finger 3	protein_coding	NP_001092759.1;XP_011509411.1;	-3.6
TJAP1	tight junction associated protein 1	protein_coding	NP_001139488.1;NP_001139489.1;	-3.6
TMEM198	transmembrane protein 198	protein_coding	NP_001005209.1;NP_001290027.1;	-3.6
ARHGAP27	Rho GTPase activating protein 27	protein_coding	NP_001269219.1;NP_777579.2;NP_	-3.6
ZNF710	zinc finger protein 710	protein_coding	NP_940928.2;XP_005254963.1;XP_	-3.6
ASMTL	acetylserotonin O-methyltransferase like	protein_coding	NP_001166944.1;NP_001166945.1;	-3.6
LOC107984289	Ras homolog, mTORC1 binding pseudogene	pseudogene	.	-3.6

MAP2K6	mitogen-activated protein kinase kinase 6	protein_coding	NP_001317379.1;NP_002749.2;XP_	-3.6
LOC105372401	uncharacterized LOC105372401	lncRNA	.	-3.6
PCED1A	PC-esterase domain containing 1A	protein_coding	NP_001258097.1;NP_073597.2;XP_	-3.6
GEMIN7	gem nuclear organelle associated protein 7	protein_coding	NP_001007270.1;NP_001007271.1;	-3.6
RABGGTA	Rab geranylgeranyltransferase subunit alpha	protein_coding	NP_004572.3;NP_878256.1	-3.6
JTB	jumping translocation breakpoint	protein_coding	NP_006685.1	-3.6
WDR90	WD repeat domain 90	protein_coding	NP_660337.3;XP_016878512.1;XP_	-3.6
FOLR1	folate receptor alpha	protein_coding	NP_000793.1;NP_057936.1;NP_05	-3.6
CNNM3	cyclin and CBS domain divalent metal cation trans	protein_coding	NP_060093.3;NP_951060.1;XP_01	-3.6
MAVS	mitochondrial antiviral signaling protein	protein_coding	NP_001193420.1;NP_065797.2	-3.6
ISL2	ISL LIM homeobox 2	protein_coding	NP_665804.1	-3.6
TMEM179B	transmembrane protein 179B	protein_coding	NP_001350528.1;NP_001350529.1;	-3.6
INPP5K	inositol polyphosphate-5-phosphatase K	protein_coding	NP_001129114.1;NP_057616.2;NP_	-3.6
FOXC1	forkhead box C1	protein_coding	NP_001444.2	-3.6
MAGED4	MAGE family member D4	protein_coding	NP_001092270.1;NP_001258990.1;	-3.6
ECHS1	enoyl-CoA hydratase, short chain 1	protein_coding	NP_004083.3	-3.6
DNMT3A	DNA methyltransferase 3 alpha	protein_coding	NP_001307821.1;NP_001307822.1;	-3.6
CTDSP2	CTD small phosphatase 2	protein_coding	NP_005721.3;XP_005268613.1	-3.6
POLR2I	RNA polymerase II subunit I	protein_coding	NP_006224.1	-3.6
EGLN3	egl-9 family hypoxia inducible factor 3	protein_coding	NP_001295032.1;NP_071356.1	-3.6
PHB2	prohibitin 2	protein_coding	NP_001138303.1;NP_001254629.1	-3.6
LPP-AS2	LPP antisense RNA 2	lncRNA	.	-3.6
MAGED4B	MAGE family member D4B	protein_coding	NP_001229291.1;NP_110428.2;NP_	-3.6
DVL3	dishevelled segment polarity protein 3	protein_coding	NP_004414.3;XP_005247229.1;XP_	-3.6
MAP6D1	MAP6 domain containing 1	protein_coding	NP_079147.1	-3.6
SELENOW	selenoprotein W	protein_coding	NP_003000.1	-3.6
FTLP14	ferritin light chain pseudogene 14	pseudogene	.	-3.6
CDK2AP1	cyclin dependent kinase 2 associated protein 1	protein_coding	NP_001257362.1;NP_001257363.1;	-3.6
PLXNA3	plexin A3	protein_coding	NP_059984.3;XP_005274762.2;XP_	-3.6
POLR2A	RNA polymerase II subunit A	protein_coding	NP_000928.1	-3.6
EHBP1L1	EH domain binding protein 1 like 1	protein_coding	NP_001092879.1;NP_001338016.1;	-3.7
PACS1	phosphofurin acidic cluster sorting protein 1	protein_coding	NP_060496.2;XP_011543464.1;XP_	-3.7
SEMA4B	semaphorin 4B	protein_coding	NP_001310958.1;NP_001310959.1;	-3.7
SNORD157	small nucleolar RNA, C/D box 157	snoRNA	.	-3.7
ACOX3	acyl-CoA oxidase 3, pristanoyl	protein_coding	NP_001095137.1;NP_001362712.1;	-3.7
LOC728975	uncharacterized LOC728975	lncRNA	.	-3.7
RHBDF2	rhomboid 5 homolog 2	protein_coding	NP_001005498.2;NP_001363157.1;	-3.7
PCDHGB2	protocadherin gamma subfamily B, 2	protein_coding	NP_061746.1;NP_115267.1	-3.7
LOC105375798	uncharacterized LOC105375798, transcript varian	lncRNA	.	-3.7
EXOC7	exocyst complex component 7	protein_coding	NP_001013861.1;NP_001138769.1;	-3.7
CRELD2	cysteine rich with EGF like domains 2	protein_coding	NP_001128573.1;NP_001271246.1;	-3.7
SIRPA	signal regulatory protein alpha	protein_coding	NP_001035111.1;NP_001035112.1;	-3.7
AVPI1	arginine vasopressin induced 1	protein_coding	NP_068378.2;XP_016871983.1	-3.7
RTL8A	retrotransposon Gag like 8A	protein_coding	NP_001071640.1;NP_001127793.1	-3.7
NFKBID	NFKB inhibitor delta	protein_coding	NP_001308760.1;NP_001352634.1;	-3.7
GDI1	GDP dissociation inhibitor 1	protein_coding	NP_001484.1	-3.7
LPCAT1	lysophosphatidylcholine acyltransferase 1	protein_coding	NP_079106.3;XP_005248430.1;XP_	-3.7
NUAK2	NUAK family kinase 2	protein_coding	NP_112214.3;XP_005245572.1	-3.7
SREBF2	sterol regulatory element binding transcription fac	protein_coding	NP_004590.2;XP_006724373.1;XP_	-3.7
AP5S1	adaptor related protein complex 5 subunit sigma 1	protein_coding	NP_001191375.1;NP_001191376.1;	-3.7
ALDH1B1	aldehyde dehydrogenase 1 family member B1	protein_coding	NP_000683.3;XP_011516104.1	-3.7
MZT2A	mitotic spindle organizing protein 2A	protein_coding	NP_001078834.1;XP_005263799.1;	-3.7
CTC-338M12.4	uncharacterized LOC101928649	lncRNA	.	-3.7
ZNHIT1	zinc finger HIT-type containing 1	protein_coding	NP_006340.1;XP_011514041.1	-3.7
AACS	acetoacetyl-CoA synthetase	protein_coding	NP_001306768.1;NP_001306769.1;	-3.7
PCIF1	PDX1 C-terminal inhibiting factor 1	protein_coding	NP_071387.1;XP_011527282.1;XP_	-3.7
SLC38A5	solute carrier family 38 member 5	protein_coding	NP_277053.2;XP_005272751.2;XP_	-3.7
TADA2B	transcriptional adaptor 2B	protein_coding	NP_689506.2;XP_011511897.1	-3.7
LOC727751	golgin A2 pseudogene	ranscribed_pseudogen	.	-3.7
LRRC8E	leucine rich repeat containing 8 VRAC subunit E	protein_coding	NP_001255213.1;NP_001255214.1;	-3.7
SLX4	SLX4 structure-specific endonuclease subunit	protein_coding	NP_115820.2;XP_011521017.1;XP_	-3.7
DNASE2	deoxyribonuclease 2, lysosomal	protein_coding	NP_001366.1	-3.7
PSMB10	proteasome 20S subunit beta 10	protein_coding	NP_002792.1	-3.7
PFN1	profilin 1	protein_coding	NP_001362920.1;NP_005013.1	-3.7
EMC9	ER membrane protein complex subunit 9	protein_coding	NP_001333803.1;NP_001333804.1;	-3.7
PRDM11	PR/SET domain 11	protein_coding	NP_001243624.1;NP_001243625.1;	-3.7
WDR13	WD repeat domain 13	protein_coding	NP_001159898.1;NP_001334146.1;	-3.7
PAGR1	PAXIP1 associated glutamate rich protein 1	protein_coding	NP_078792.1	-3.7
MAP3K14	mitogen-activated protein kinase kinase kinase 14	protein_coding	NP_003945.2;XP_011523743.1	-3.7
PPM1M	protein phosphatase, Mg2+/Mn2+ dependent 1M	protein_coding	NP_001116342.1;NP_653242.3;XP_	-3.7
DRG2	developmentally regulated GTP binding protein 2	protein_coding	NP_001317073.1;NP_001379.1;XP_	-3.7

DAAM2	dishevelled associated activator of morphogenesis	protein_coding	NP_001188356.1;NP_056160.2;XP_	-3.7
MAPK8IP3	mitogen-activated protein kinase 8 interacting prot	protein_coding	NP_001035529.1;NP_001305781.1;	-3.7
EEF1AKMT4	EEF1A lysine methyltransferase 4	protein_coding	NP_115707.2	-3.7
RNF31	ring finger protein 31	protein_coding	NP_001297261.1;NP_060469.4	-3.7
BOC	BOC cell adhesion associated, oncogene regulated	protein_coding	NP_001288790.1;NP_001288796.1;	-3.7
SPATA2	spermatogenesis associated 2	protein_coding	NP_001129245.1;NP_006029.1;XP_	-3.7
MDH2	malate dehydrogenase 2	protein_coding	NP_001269332.1;NP_001269333.1;	-3.7
KAT2A	lysine acetyltransferase 2A	protein_coding	NP_001363156.1;NP_066564.2;XP_	-3.7
WDR46	WD repeat domain 46	protein_coding	NP_001157739.1;NP_005443.3;XP_	-3.7
PKNOX2	PBX/knotted 1 homeobox 2	protein_coding	NP_001369252.1;NP_001369253.1;	-3.7
CRTC2	CREB regulated transcription coactivator 2	protein_coding	NP_859066.1;XP_005245003.1;XP_	-3.7
FADS2	fatty acid desaturase 2	protein_coding	NP_001268430.1;NP_001268431.1;	-3.7
PIAS3	protein inhibitor of activated STAT 3	protein_coding	NP_006090.2	-3.7
IMPDH1P10	inosine monophosphate dehydrogenase 1 pseudog	pseudogene	.	-3.7
TYSND1	trypsin domain containing 1	protein_coding	NP_001035363.1;NP_775826.2	-3.7
IPO13	importin 13	protein_coding	NP_055467.3;XP_024306837.1;XP_	-3.8
ACOT11	acyl-CoA thioesterase 11	protein_coding	NP_056362.1;NP_671517.1	-3.8
KIAA1656	KIAA1656 protein	lncRNA	.	-3.8
PLPPR4	phospholipid phosphatase related 4	protein_coding	NP_001159724.1;NP_055654.2;XP_	-3.8
TMEM79	transmembrane protein 79	protein_coding	NP_115699.1	-3.8
IL6R	interleukin 6 receptor	protein_coding	NP_000556.1;NP_001193795.1;NP_	-3.8
DENND4B	DENN domain containing 4B	protein_coding	NP_001354395.1;NP_055671.2;XP_	-3.8
TMEM203	transmembrane protein 203	protein_coding	NP_444273.1	-3.8
FAHD2CP	fumarylacetoacetate hydrolase domain containing	ranscribed_pseudogen	.	-3.8
OR1F1	olfactory receptor family 1 subfamily F member 1	protein_coding	NP_001357568.1;NP_001357569.1;	-3.8
FMNL1	formin like 1	protein_coding	NP_005883.3;XP_006722125.1;XP_	-3.8
HEXD	hexosaminidase D	protein_coding	NP_001317471.1;NP_001356416.1;	-3.8
LOC105376326	uncharacterized LOC105376326	lncRNA	.	-3.8
LOC100287015	uncharacterized LOC100287015	lncRNA	.	-3.8
NRBP2	nuclear receptor binding protein 2	protein_coding	NP_848659.2;XP_016868867.1;XP_	-3.8
LY6K	lymphocyte antigen 6 family member K	protein_coding	NP_001153826.1;NP_001153827.1;	-3.8
HMGN2P5	high mobility group nucleosomal binding domain	pseudogene	.	-3.8
ZSWIM9	zinc finger SWIM-type containing 9	protein_coding	NP_955373.3;XP_005259506.1;XP_	-3.8
PKD1P3	polycystin 1, transient receptor potential channel i	pseudogene	.	-3.8
FAM98C	family with sequence similarity 98 member C	protein_coding	NP_001338604.1;NP_777565.3;XP_	-3.8
HAUS7	HAUS augmin like complex subunit 7	protein_coding	NP_059988.3	-3.8
HOXA11-AS	HOXA11 antisense RNA	lncRNA	.	-3.8
NOL6	nucleolar protein 6	protein_coding	NP_075068.2;NP_631981.2;XP_01	-3.8
MAEA	macrophage erythroblast attacher, E3 ubiquitin lig	protein_coding	NP_001017405.1;NP_001284359.1;	-3.8
H4C14	H4 clustered histone 14	protein_coding	NP_003539.1	-3.8
DNPEP	aspartyl aminopeptidase	protein_coding	NP_001306045.1;NP_001306046.1;	-3.8
KXD1	KxDL motif containing 1	protein_coding	NP_001165419.1;NP_001165420.1;	-3.8
AIFM2	apoptosis inducing factor mitochondria associated	protein_coding	NP_001185625.1;NP_116186.1	-3.8
LOC105376010	uncharacterized LOC105376010, transcript varian	lncRNA	.	-3.8
INAVA	innate immunity activator	protein_coding	NP_001136041.1;NP_001354218.1;	-3.8
JUN	Jun proto-oncogene, AP-1 transcription factor sub	protein_coding	NP_002219.1	-3.8
ZBTB48	zinc finger and BTB domain containing 48	protein_coding	NP_001265576.1;NP_001265577.1;	-3.8
RPP25L	ribonuclease P/MRP subunit p25 like	protein_coding	NP_680544.1;NP_680545.1	-3.8
COLEC12	collectin subfamily member 12	protein_coding	NP_569057.2;XP_011524043.1	-3.8
AP2M1	adaptor related protein complex 2 subunit mu 1	protein_coding	NP_001020376.1;NP_001298127.1;	-3.8
TMEM143	transmembrane protein 143	protein_coding	NP_001290467.1;NP_001290468.1;	-3.8
TLNRD1	talin rod domain containing 1	protein_coding	NP_072088.1	-3.8
TMEM63B	transmembrane protein 63B	protein_coding	NP_001305721.1;NP_060896.1;XP_	-3.8
PRRG4	proline rich and Gla domain 4	protein_coding	NP_076986.1;XP_006718376.3;XP_	-3.8
CLPTM1L	CLPTM1 like	protein_coding	NP_110409.2;XP_011512446.1;XP_	-3.8
PLOD1	procollagen-lysine,2-oxoglutarate 5-dioxygenase 1	protein_coding	NP_000293.2;NP_001303249.1	-3.8
PKD1P2	polycystin 1, transient receptor potential channel i	pseudogene	.	-3.8
NBEAL2	neurobeachin like 2	protein_coding	NP_001352045.1;NP_055990.1;XP_	-3.8
IRX3	iroquois homeobox 3	protein_coding	NP_077312.2;XP_005256196.1	-3.8
HOXB4	homeobox B4	protein_coding	NP_076920.1	-3.8
TTC38	tetratricopeptide repeat domain 38	protein_coding	NP_060401.3;XP_011528561.1;XP_	-3.8
RPTOR	regulatory associated protein of MTOR complex 1	protein_coding	NP_001156506.1;NP_065812.1	-3.8
ASIC1	acid sensing ion channel subunit 1	protein_coding	NP_001086.2;NP_001243759.1;NP_	-3.8
NTMT1	N-terminal Xaa-Pro-Lys N-methyltransferase 1	protein_coding	NP_001273725.1;NP_001273726.1;	-3.8
RDH13	retinol dehydrogenase 13	protein_coding	NP_001139443.1;NP_612421.1;XP_	-3.8
MAMSTR	MEF2 activating motif and SAP domain containin	protein_coding	NP_001124387.1;NP_001284682.1;	-3.8
IVD	isovaleryl-CoA dehydrogenase	protein_coding	NP_001152980.2;NP_001341526.1;	-3.8
CCDC107	coiled-coil domain containing 107	protein_coding	NP_001182129.1;NP_001182130.1;	-3.8
DEAF1	DEAF1 transcription factor	protein_coding	NP_001280563.1;NP_001354319.1;	-3.8
TNFAIP2	TNF alpha induced protein 2	protein_coding	NP_001358149.1;NP_001358150.1;	-3.8
GOLGA2P7	GOLGA2 pseudogene 7	ranscribed_pseudogen	.	-3.8

CCDC137	coiled-coil domain containing 137	protein_coding	NP_954981.1;XP_011523040.1;XP_	-3.8
GTF2IRD1	GTF2I repeat domain containing 1	protein_coding	NP_001186136.1;NP_005676.3;NP_	-3.8
BDKRB1	bradykinin receptor B1	protein_coding	NP_000701.2	-3.8
LOC105372836	uncharacterized LOC105372836, transcript varian	protein_coding	XP_011528114.1	-3.8
LOC105378949	collagen alpha-2(VIII) chain	lncRNA	.	-3.8
RAC2	Rac family small GTPase 2	protein_coding	NP_002863.1;XP_006724349.1	-3.8
PROSER2	proline and serine rich 2	protein_coding	NP_694988.3;XP_011517740.1;XP_	-3.9
ARID3A	AT-rich interaction domain 3A	protein_coding	NP_005215.1;XP_005259570.1;XP_	-3.9
RAD9A	RAD9 checkpoint clamp component A	protein_coding	NP_001230153.1;NP_004575.1;XP_	-3.9
ZFAND2B	zinc finger AN1-type containing 2B	protein_coding	NP_001257927.1;NP_001257928.1;	-3.9
PANK4	pantothenate kinase 4 (inactive)	protein_coding	NP_060686.3;XP_011540010.2	-3.9
DHRS4	dehydrogenase/reductase 4	protein_coding	NP_001269916.1;NP_001269917.1;	-3.9
CNNM4	cyclin and CBS domain divalent metal cation trans	protein_coding	NP_064569.3;XP_005263971.1;XP_	-3.9
RNASEH2C	ribonuclease H2 subunit C	protein_coding	NP_115569.2	-3.9
COTL1	coactosin like F-actin binding protein 1	protein_coding	NP_066972.1	-3.9
SLC4A3	solute carrier family 4 member 3	protein_coding	NP_001313488.1;NP_005061.3;NP_	-3.9
TUBA4A	tubulin alpha 4a	protein_coding	NP_001265481.1;NP_005991.1;XP_	-3.9
VPS37B	VPS37B subunit of ESCRT-I	protein_coding	NP_078943.1;XP_005253679.1;XP_	-3.9
PLEKHG5	pleckstrin homology and RhoGEF domain contain	protein_coding	NP_001036128.2;NP_001036129.1;	-3.9
SEPTIN4	septin 4	protein_coding	NP_001033793.3;NP_001185642.1;	-3.9
DPH2	diphthamide biosynthesis 2	protein_coding	NP_001034678.1;NP_001306094.1;	-3.9
DDX11L2	DEAD/H-box helicase 11 like 2 (pseudogene)	ranscribed_pseudogen	.	-3.9
CYP26B1	cytochrome P450 family 26 subfamily B member	protein_coding	NP_001264671.1;NP_063938.1;XP_	-3.9
DNAJC22	DnaJ heat shock protein family (Hsp40) member C	protein_coding	NP_001291873.1;NP_079178.2;XP_	-3.9
TUBG1	tubulin gamma 1	protein_coding	NP_001061.2;XP_024306672.1	-3.9
WASF2	WASP family member 2	protein_coding	NP_001188333.1;NP_008921.1	-3.9
DLG5	discs large MAGUK scaffold protein 5	protein_coding	NP_004738.3;XP_005270333.1;XP_	-3.9
SHC1	SHC adaptor protein 1	protein_coding	NP_001123512.1;NP_001123513.1;	-3.9
LOC107984338	uncharacterized LOC107984338	lncRNA	.	-3.9
F8A3	coagulation factor VIII associated 3	protein_coding	NP_001007525.1	-3.9
TSHZ1	teashirt zinc finger homeobox 1	protein_coding	NP_001295139.1;NP_005777.3;XP_	-3.9
TSPYL1	TSPY like 1	protein_coding	NP_003300.1	-3.9
AHCY	adenosylhomocysteinase	protein_coding	NP_000678.1;NP_001155238.1;NP_	-3.9
IRF5	interferon regulatory factor 5	protein_coding	NP_001092097.2;NP_001092099.1;	-3.9
GPATCH3	G-patch domain containing 3	protein_coding	NP_071361.2	-3.9
FLG-AS1	FLG antisense RNA 1	lncRNA	.	-3.9
SDC2	syndecan 2	protein_coding	NP_002989.2;XP_011515514.1;XP_	-3.9
IQSEC2	IQ motif and Sec7 domain ArfGEF 2	protein_coding	NP_001104595.1;NP_001230126.1;	-3.9
PRRT1	proline rich transmembrane protein 1	protein_coding	NP_001350709.1;NP_085154.3;XP_	-3.9
THAP11	THAP domain containing 11	protein_coding	NP_065190.2	-3.9
TNIP2	TNFAIP3 interacting protein 2	protein_coding	NP_001154999.1;NP_001278945.1;	-3.9
CD2BP2	CD2 cytoplasmic tail binding protein 2	protein_coding	NP_001230575.1;NP_006101.1	-3.9
ELL	elongation factor for RNA polymerase II	protein_coding	NP_006523.1;XP_011526632.2;XP_	-3.9
DTWD2	DTW domain containing 2	protein_coding	NP_001295010.1;NP_775937.1;XP_	-3.9
NAGA	alpha-N-acetylgalactosaminidase	protein_coding	NP_000253.1;NP_001349777.1;NP_	-3.9
GALE	UDP-galactose-4-epimerase	protein_coding	NP_000394.2;NP_001008217.1;NP_	-3.9
SPOUT1	SPOUT domain containing methyltransferase 1	protein_coding	NP_057474.2;XP_016870293.1	-3.9
NADK	NAD kinase	protein_coding	NP_001185922.1;NP_001185923.1;	-3.9
PPARD	peroxisome proliferator activated receptor delta	protein_coding	NP_001165289.1;NP_001165290.1;	-3.9
AUP1	AUP1 lipid droplet regulating VLDL assembly fac	protein_coding	NP_853553.1	-3.9
TMEM223	transmembrane protein 223	protein_coding	NP_001073970.1	-3.9
CHFR	checkpoint with forkhead and ring finger domains	protein_coding	NP_001154816.1;NP_001154817.1;	-3.9
GRB2	growth factor receptor bound protein 2	protein_coding	NP_002077.1;NP_987102.1	-3.9
COMMD4	COMM domain containing 4	protein_coding	NP_001271306.1;NP_001271307.1;	-3.9
TMUB2	transmembrane and ubiquitin like domain containi	protein_coding	NP_001070142.1;NP_001317164.1;	-3.9
OAZ2	ornithine decarboxylase antizyme 2	protein_coding	NP_001288231.1;NP_002528.1	-4.0
MRPL34	mitochondrial ribosomal protein L34	protein_coding	NP_001369271.1;NP_001369272.1;	-4.0
RNPEP	arginyl aminopeptidase	protein_coding	NP_001306111.1;NP_001306112.1;	-4.0
PCSK9	proprotein convertase subtilisin/kexin type 9	protein_coding	NP_777596.2	-4.0
MRPS18A	mitochondrial ribosomal protein S18A	protein_coding	NP_001180272.1;NP_060605.1;XP_	-4.0
RNPS1	RNA binding protein with serine rich domain 1	protein_coding	NP_001273554.1;NP_001273555.1;	-4.0
TOB2P1	transducer of ERBB2, 2 pseudogene 1	ranscribed_pseudogen	.	-4.0
AMIGO1	adhesion molecule with Ig like domain 1	protein_coding	NP_065754.2;XP_011540114.1	-4.0
H4C3	H4 clustered histone 3	protein_coding	NP_003533.1	-4.0
NRXN2	neurexin 2	protein_coding	NP_001363191.1;NP_001363192.1;	-4.0
MRPS2	mitochondrial ribosomal protein S2	protein_coding	NP_001358330.1;NP_057118.1	-4.0
FKBP10	FKBP prolyl isomerase 10	protein_coding	NP_068758.3;XP_011523401.1;XP_	-4.0
ARHGEF25	Rho guanine nucleotide exchange factor 25	protein_coding	NP_001104740.2;NP_001334862.2;	-4.0
STOML2	stomatin like 2	protein_coding	NP_001273960.1;NP_001273961.1;	-4.0
HPS1	HPS1 biogenesis of lysosomal organelles complex	protein_coding	NP_000186.2;NP_001298274.1;NP_	-4.0
KRT18	keratin 18	protein_coding	NP_000215.1;NP_954657.1	-4.0

ZNF500	zinc finger protein 500	protein_coding	NP_001290379.1;NP_067678.1;XP_	-4.0
LHPP	phospholysine phosphohistidine inorganic pyropho	protein_coding	NP_001161352.1;NP_001305260.1;	-4.0
ANKRD34A	ankyrin repeat domain 34A	protein_coding	NP_001034977.1	-4.0
GSTM4	glutathione S-transferase mu 4	protein_coding	NP_000841.1;NP_671489.1;XP_01	-4.0
UBXN11	UBX domain protein 11	protein_coding	NP_001070730.1;NP_663320.2;NP_	-4.0
FZD7	frizzled class receptor 7	protein_coding	NP_003498.1	-4.0
EVL	Enah/Vasp-like	protein_coding	NP_001317150.1;NP_057421.1;XP_	-4.0
RMND5B	required for meiotic nuclear division 5 homolog B	protein_coding	NP_001275723.1;NP_001275724.1;	-4.0
ZNF395	zinc finger protein 395	protein_coding	NP_061130.1	-4.0
PIK3CD	phosphatidylinositol-4,5-bisphosphate 3-kinase ca	protein_coding	NP_001337163.1;NP_001337164.1;	-4.0
RETREG2	reticulophagy regulator family member 2	protein_coding	NP_001308038.1;NP_001308039.1;	-4.0
LOC107987294	uncharacterized LOC107987294, transcript varian	lncRNA	.	-4.0
POM121	POM121 transmembrane nucleoporin	protein_coding	NP_001244119.1;NP_001354539.1;	-4.0
CENPV	centromere protein V	protein_coding	NP_859067.2	-4.0
MED15	mediator complex subunit 15	protein_coding	NP_001003891.1;NP_001280163.1;	-4.0
SKIV2L	Ski2 like RNA helicase	protein_coding	NP_008860.4;XP_011513117.1	-4.0
RPLP0P6	ribosomal protein lateral stalk subunit P0 pseudog	pseudogene	.	-4.0
PLD6	phospholipase D family member 6	protein_coding	NP_849158.2;XP_016879799.2	-4.0
ORAI3	ORAI calcium release-activated calcium modulato	protein_coding	NP_689501.1	-4.0
RNU4-2	RNA, U4 small nuclear 2	snRNA	.	-4.0
NENF	neudesin neurotrophic factor	protein_coding	NP_037481.1	-4.0
APOBEC3F	apolipoprotein B mRNA editing enzyme catalytic	protein_coding	NP_001006667.1;NP_660341.2;XP_	-4.0
OGFOD2	2-oxoglutarate and iron dependent oxygenase dom	protein_coding	NP_001291762.1;NP_001291763.1;	-4.0
DEF8	differentially expressed in FDCP 8 homolog	protein_coding	NP_001229745.1;NP_001229746.1;	-4.0
DCTPP1	dCTP pyrophosphatase 1	protein_coding	NP_077001.1	-4.0
LOC101929479	golgin A2 pseudogene	ranscribed_pseudogen	.	-4.0
APOOL	apolipoprotein O like	protein_coding	NP_940852.3;XP_011529161.1;XP_	-4.0
SNORD88B	small nucleolar RNA, C/D box 88B	snoRNA	.	-4.0
CDKN2AIPNL	CDKN2A interacting protein N-terminal like	protein_coding	NP_542387.1;XP_011542011.1	-4.0
TP53I3	tumor protein p53 inducible protein 3	protein_coding	NP_001193731.1;NP_004872.2;NP_	-4.0
PLEKHA4	pleckstrin homology domain containing A4	protein_coding	NP_001154826.1;NP_065955.2;XP_	-4.0
SLC48A1	solute carrier family 48 member 1	protein_coding	NP_060312.2;XP_005269073.1;XP_	-4.0
JAG2	jagged canonical Notch ligand 2	protein_coding	NP_002217.3;NP_660142.1	-4.0
CDK9	cyclin dependent kinase 9	protein_coding	NP_001252.1	-4.0
FRMD6-AS1	FRMD6 antisense RNA 1	lncRNA	.	-4.0
SIPA1L3	signal induced proliferation associated 1 like 3	protein_coding	NP_055888.1;XP_005258728.1;XP_	-4.0
SIK1B	salt inducible kinase 1B (putative)	protein_coding	NP_001307572.1;XP_016883697.1	-4.0
IGFL2-AS1	IGFL2 antisense RNA 1	lncRNA	.	-4.0
PES1	pescadillo ribosomal biogenesis factor 1	protein_coding	NP_001230154.1;NP_001269256.1;	-4.0
HMOX2	heme oxygenase 2	protein_coding	NP_001120676.1;NP_001120677.1;	-4.1
LOC105372296	uncharacterized LOC105372296	lncRNA	.	-4.1
RAB31	RAB31, member RAS oncogene family	protein_coding	NP_006859.2;XP_016881017.1;XP_	-4.1
HAUS5	HAUS augmin like complex subunit 5	protein_coding	NP_056117.1;XP_011524986.1	-4.1
TBC1D24	TBC1 domain family member 24	protein_coding	NP_001186036.1;NP_065756.1;XP_	-4.1
OTUB1	OTU deubiquitinase, ubiquitin aldehyde binding 1	protein_coding	NP_060140.2	-4.1
UCKL1	uridine-cytidine kinase 1 like 1	protein_coding	NP_001180308.1;NP_001340404.1;	-4.1
FADD	Fas associated via death domain	protein_coding	NP_003815.1	-4.1
UCK1	uridine-cytidine kinase 1	protein_coding	NP_001129426.1;NP_001248379.1;	-4.1
GPANK1	G-patch domain and ankyrin repeats 1	protein_coding	NP_001186166.1;NP_001186167.1;	-4.1
DCTN1	dynactin subunit 1	protein_coding	NP_001128512.1;NP_001128513.1;	-4.1
TWIST2	twist family bHLH transcription factor 2	protein_coding	NP_001258822.1;NP_476527.1	-4.1
ZNF696	zinc finger protein 696	protein_coding	NP_112157.2	-4.1
OPA3	outer mitochondrial membrane lipid metabolism re	protein_coding	NP_001017989.2;NP_079412.1;XP_	-4.1
GLMP	glycosylated lysosomal membrane protein	protein_coding	NP_001243533.1;NP_001243534.1;	-4.1
PTPN18	protein tyrosine phosphatase non-receptor type 18	protein_coding	NP_001135842.1;NP_055184.2;XP_	-4.1
LEMD2	LEM domain nuclear envelope protein 2	protein_coding	NP_001137416.1;NP_001335638.1;	-4.1
MYCL	MYCL proto-oncogene, bHLH transcription facto	protein_coding	NP_001028253.1;NP_001028254.2;	-4.1
FBXL12	F-box and leucine rich repeat protein 12	protein_coding	NP_001303865.1;NP_001303866.1;	-4.1
TMEM92	transmembrane protein 92	protein_coding	NP_001161687.1;NP_694961.2;XP_	-4.1
SERPING1	serpin family G member 1	protein_coding	NP_000053.2;NP_001027466.1	-4.1
ST3GAL4	ST3 beta-galactoside alpha-2,3-sialyltransferase 4	protein_coding	NP_001241686.1;NP_001241687.1;	-4.1
ANKRD39	ankyrin repeat domain 39	protein_coding	NP_057550.3	-4.1
KCNJ12	potassium inwardly rectifying channel subfamily J	protein_coding	NP_066292.2;XP_005256682.1;XP_	-4.1
TMEM147-AS1	TMEM147 antisense RNA 1	lncRNA	.	-4.1
RGL2	ral guanine nucleotide dissociation stimulator like	protein_coding	NP_001230667.1;NP_004752.1;XP_	-4.1
HMGN1P38	high mobility group nucleosome binding domain 1	pseudogene	.	-4.1
RMRP	RNA component of mitochondrial RNA processin	RNase_MRP_RNA	.	-4.1
DNAJC5	DnaJ heat shock protein family (Hsp40) member C	protein_coding	NP_079495.1;XP_011527350.1;XP_	-4.1
PHF1	PHD finger protein 1	protein_coding	NP_002627.2;NP_077084.2;XP_01	-4.1
AHNAK2	AHNAK nucleoprotein 2	protein_coding	NP_001337858.1;NP_612429.2;XP_	-4.1
SLC35A2	solute carrier family 35 member A2	protein_coding	NP_001027460.1;NP_001035963.1;	-4.1

WDR62	WD repeat domain 62	protein_coding	NP_001077430.1;NP_775907.4;XP_	-4.1
H2BC15	H2B clustered histone 15	protein_coding	NP_003511.1	-4.1
OTUD5	OTU deubiquitinase 5	protein_coding	NP_001129629.1;NP_001129630.1;	-4.1
POP7	POP7 homolog, ribonuclease P/MRP subunit	protein_coding	NP_005828.2	-4.1
SYMPK	symplekin	protein_coding	NP_004810.2;XP_005259343.1;XP_	-4.1
SYPL2	synaptophysin like 2	protein_coding	NP_001035799.1;XP_011539585.1;	-4.1
CAMTA2	calmodulin binding transcription activator 2	protein_coding	NP_001164637.1;NP_001164638.1;	-4.1
TSPAN15	tetraspanin 15	protein_coding	NP_001338192.1;NP_036471.1;XP_	-4.1
ACOT8	acyl-CoA thioesterase 8	protein_coding	NP_005460.2;XP_005260296.1;XP_	-4.1
PIM2	Pim-2 proto-oncogene, serine/threonine kinase	protein_coding	NP_006866.2	-4.1
C6orf47	chromosome 6 open reading frame 47	protein_coding	NP_067007.3	-4.1
VPS37C	VPS37C subunit of ESCRT-I	protein_coding	NP_060436.4;XP_005274134.1;XP_	-4.1
NAA10	N-alpha-acetyltransferase 10, NatA catalytic subu	protein_coding	NP_001243048.1;NP_001243049.1;	-4.1
MAMDC4	MAM domain containing 4	protein_coding	NP_996803.2	-4.1
MUL1	mitochondrial E3 ubiquitin protein ligase 1	protein_coding	NP_078820.2;XP_011540439.1	-4.1
QSOX1	quiescin sulfhydryl oxidase 1	protein_coding	NP_001004128.1;NP_002817.2	-4.1
C9orf116	chromosome 9 open reading frame 116	protein_coding	NP_001041730.1;NP_653255.1	-4.1
ZSWIM4	zinc finger SWIM-type containing 4	protein_coding	NP_001354763.1;NP_075560.2;XP_	-4.1
TKFC	triokinase and FMN cyclase	protein_coding	NP_001338905.1;NP_001338906.1;	-4.1
GLRX5	glutaredoxin 5	protein_coding	NP_057501.2	-4.1
ZNF746	zinc finger protein 746	protein_coding	NP_001156946.1;NP_001350446.1;	-4.1
ERCC1	ERCC excision repair 1, endonuclease non-catalyt	protein_coding	NP_001159521.1;NP_001356337.1;	-4.1
PDXK	pyridoxal kinase	protein_coding	NP_001317959.1;NP_003672.1;XP_	-4.2
ABL1	ABL proto-oncogene 1, non-receptor tyrosine kin	protein_coding	NP_005148.2;NP_009297.2	-4.2
CCDC159	coiled-coil domain containing 159	protein_coding	NP_001073972.2;XP_006722706.1;	-4.2
ABHD15	abhydrolase domain containing 15	protein_coding	NP_937790.2	-4.2
MFSD5	major facilitator superfamily domain containing 5	protein_coding	NP_001164261.1;NP_116278.3;XP_	-4.2
LOC388242	SAGA complex associated factor 29 pseudogene	ranscribed_pseudogen	.	-4.2
ARHGEF40	Rho guanine nucleotide exchange factor 40	protein_coding	NP_001265458.1;NP_001265459.1;	-4.2
HSPB8	heat shock protein family B (small) member 8	protein_coding	NP_055180.1	-4.2
FBXL14	F-box and leucine rich repeat protein 14	protein_coding	NP_689654.1;XP_016874364.1;XP_	-4.2
KMT5AP1	KMT5A pseudogene 1	pseudogene	.	-4.2
ATF6B	activating transcription factor 6 beta	protein_coding	NP_001129625.1;NP_004372.3	-4.2
HLA-C	major histocompatibility complex, class I, C	protein_coding	NP_002108.4	-4.2
UBA1	ubiquitin like modifier activating enzyme 1	protein_coding	NP_003325.2;NP_695012.1;XP_00	-4.2
TMEM120B	transmembrane protein 120B	protein_coding	NP_001074294.2;XP_024304619.1;	-4.2
DHX34	DExH-box helicase 34	protein_coding	NP_055496.2;XP_005259500.1;XP_	-4.2
IMP3	IMP U3 small nucleolar ribonucleoprotein 3	protein_coding	NP_060755.1	-4.2
WHRN	whirlin	protein_coding	NP_001077354.2;NP_001166896.1;	-4.2
DALRD3	DALR anticodon binding domain containing 3	protein_coding	NP_001009996.1;NP_001263334.1;	-4.2
CCNQ	cyclin Q	protein_coding	NP_001124469.1;NP_689487.2;XP_	-4.2
PREB	prolactin regulatory element binding	protein_coding	NP_001317413.1;NP_001317414.1;	-4.2
BMP1	bone morphogenetic protein 1	protein_coding	NP_001190.1;NP_006120.1	-4.2
HGS	hepatocyte growth factor-regulated tyrosine kinas	protein_coding	NP_004703.1	-4.2
LYPD1	LY6/PLAUR domain containing 1	protein_coding	NP_001070895.1;NP_001308163.1;	-4.2
EEFSEC	eukaryotic elongation factor, selenocysteine-tRNA	protein_coding	NP_068756.2;XP_005247753.1;XP_	-4.2
GIPR	gastric inhibitory polypeptide receptor	protein_coding	NP_000155.1;NP_001295347.1;XP_	-4.2
DOLPP1	dolichyldiphosphatase 1	protein_coding	NP_001129389.1;NP_065171.2	-4.2
SURF4	surfeit 4	protein_coding	NP_001267717.1;NP_001267718.1;	-4.2
TEN1	TEN1 subunit of CST complex	protein_coding	NP_001106795.2	-4.2
TLCD4	TLC domain containing 4	protein_coding	NP_001186608.1;NP_689700.1	-4.2
FLOT1	flotillin 1	protein_coding	NP_001305804.1;NP_005794.1;XP_	-4.2
INPPL1	inositol polyphosphate phosphatase like 1	protein_coding	NP_001558.3;XP_005274036.1;XP_	-4.2
CXXC1	CXXC finger protein 1	protein_coding	NP_001095124.1;NP_055408.2;XP_	-4.2
ZNF324	zinc finger protein 324	protein_coding	NP_055162.1;XP_005258770.1	-4.2
DENND2B	DENN domain containing 2B	protein_coding	NP_001363424.1;NP_001363425.1;	-4.2
EIF4G1	eukaryotic translation initiation factor 4 gamma 1	protein_coding	NP_001181875.2;NP_001181876.1;	-4.2
USP20	ubiquitin specific peptidase 20	protein_coding	NP_001008563.2;NP_001103773.2;	-4.2
SLITRK5	SLIT and NTRK like family member 5	protein_coding	NP_056382.1;XP_005254095.1;XP_	-4.2
SUPT5H	SPT5 homolog, DSIF elongation factor subunit	protein_coding	NP_001104490.1;NP_001124296.1;	-4.2
SYNC	syncoilin, intermediate filament protein	protein_coding	NP_001155180.2;NP_110413.3;XP_	-4.2
GOLGA2P10	GOLGA2 pseudogene 10	ranscribed_pseudogen	.	-4.2
LOC107985687	translation initiation factor IF-2-like	protein_coding	XP_016885495.1	-4.2
SNN	stannin	protein_coding	NP_003489.1;XP_016879230.1;XP_	-4.2
MED25	mediator complex subunit 25	protein_coding	NP_001365284.1;NP_112235.2	-4.2
A1BG-AS1	A1BG antisense RNA 1	lncRNA	.	-4.3
HOXC5	homeobox C5	protein_coding	NP_061826.1	-4.3
CTDNEP1	CTD nuclear envelope phosphatase 1	protein_coding	NP_001137247.1;NP_056158.2	-4.3
FA2H	fatty acid 2-hydroxylase	protein_coding	NP_077282.3;XP_011521619.1;XP_	-4.3
HHIP	hedgehog interacting protein	protein_coding	NP_071920.1;XP_005263235.1;XP_	-4.3
NDUFA6-DT	NDUFA6 divergent transcript	lncRNA	.	-4.3

ZNF821	zinc finger protein 821	protein_coding	NP_001188481.1;NP_001188482.1;	-4.3
PCDHB16	protocadherin beta 16	protein_coding	NP_066008.2	-4.3
ZCCHC3	zinc finger CCHC-type containing 3	protein_coding	NP_149080.2	-4.3
NCK2	NCK adaptor protein 2	protein_coding	NP_001004720.1;NP_001004722.1;	-4.3
SV2A	synaptic vesicle glycoprotein 2A	protein_coding	NP_001265648.1;NP_001315603.1;	-4.3
LOC105377294	uncharacterized LOC105377294, transcript varian	lncRNA	.	-4.3
ZIC2	Zic family member 2	protein_coding	NP_009060.2	-4.3
RAB6B	RAB6B, member RAS oncogene family	protein_coding	NP_001350882.1;NP_057661.3	-4.3
LOC105372501	uncharacterized LOC105372501, transcript varian	lncRNA	.	-4.3
MINK1	misshapen like kinase 1	protein_coding	NP_001020108.1;NP_001308165.1;	-4.3
ZCCHC24	zinc finger CCHC-type containing 24	protein_coding	NP_699198.2;XP_011537754.1	-4.3
JAK3	Janus kinase 3	protein_coding	NP_000206.2;XP_011526292.1;XP_	-4.3
CD99L2	CD99 molecule like 2	protein_coding	NP_001171737.1;NP_001229543.1;	-4.3
CABLES2	Cdk5 and Abl enzyme substrate 2	protein_coding	NP_112492.2	-4.3
ARF1	ADP ribosylation factor 1	protein_coding	NP_001019397.1;NP_001019398.1;	-4.3
MSX1	msh homeobox 1	protein_coding	NP_002439.2	-4.3
WASH2P	WASP family homolog 2, pseudogene	ranscribed_pseudogen	.	-4.3
TAF1C	TATA-box binding protein associated factor, RNA	protein_coding	NP_001230085.2;NP_001230086.1;	-4.3
SCD5	stearoyl-CoA desaturase 5	protein_coding	NP_001032671.2;NP_079182.2	-4.3
SIK1	salt inducible kinase 1	protein_coding	NP_775490.2;XP_011527776.1	-4.3
LRRC75A	leucine rich repeat containing 75A	protein_coding	NP_001107039.1;NP_997270.2;XP_	-4.3
PCBP3	poly(rC) binding protein 3	protein_coding	NP_001123613.1;NP_001335167.1;	-4.3
AGPAT3	1-acylglycerol-3-phosphate O-acyltransferase 3	protein_coding	NP_001032642.1;NP_001356807.1;	-4.3
SLC22A23	solute carrier family 22 member 23	protein_coding	NP_001273384.1;NP_001273385.1;	-4.3
MAPKAPK2	MAPK activated protein kinase 2	protein_coding	NP_004750.1;NP_116584.2;XP_00	-4.3
ERBB3	erb-b2 receptor tyrosine kinase 3	protein_coding	NP_001005915.1;NP_001973.2	-4.3
GALNT2	polypeptide N-acetylgalactosaminyltransferase 2	protein_coding	NP_001278795.1;NP_004472.1;XP_	-4.3
IER5	immediate early response 5	protein_coding	NP_057629.2	-4.3
FAM53C	family with sequence similarity 53 member C	protein_coding	NP_001129119.1;NP_001337123.1;	-4.3
TRPC6	transient receptor potential cation channel subfami	protein_coding	NP_004612.2;XP_011541270.1;XP_	-4.3
SELPLG	selectin P ligand	protein_coding	NP_001193538.1;NP_002997.2	-4.3
TTC9	tetratricopeptide repeat domain 9	protein_coding	NP_056166.1	-4.3
PYCR2	pyrroline-5-carboxylate reductase 2	protein_coding	NP_001258610.1;NP_037460.2	-4.3
CLSTN3	calsyntenin 3	protein_coding	NP_055533.2;XP_006719226.1	-4.3
PMEL	premelanosome protein	protein_coding	NP_001186982.1;NP_001186983.1;	-4.3
TYK2	tyrosine kinase 2	protein_coding	NP_003322.3;XP_011526547.1;XP_	-4.3
LOC105372705	uncharacterized LOC105372705	lncRNA	.	-4.3
UNKL	unk like zinc finger	protein_coding	NP_001032202.1;NP_001180317.2;	-4.3
MORN1	MORN repeat containing 1	protein_coding	NP_001287989.1;NP_079124.1	-4.3
H2BC21	H2B clustered histone 21	protein_coding	NP_003519.1	-4.3
COL16A1	collagen type XVI alpha 1 chain	protein_coding	NP_001847.3;XP_005270538.1;XP_	-4.3
MCAM	melanoma cell adhesion molecule	protein_coding	NP_006491.2;XP_016873248.1;XP_	-4.3
ZMYM3	zinc finger MYM-type containing 3	protein_coding	NP_001164633.1;NP_001164634.1;	-4.3
ETV4	ETS variant transcription factor 4	protein_coding	NP_001073143.1;NP_001248366.1;	-4.4
PCDHB8	protocadherin beta 8	protein_coding	NP_061993.3	-4.4
IP6K1	inositol hexakisphosphate kinase 1	protein_coding	NP_001006115.1;NP_001229758.1;	-4.4
ACBD7	acyl-CoA binding domain containing 7	protein_coding	NP_001034933.1	-4.4
PRPF31	pre-mRNA processing factor 31	protein_coding	NP_056444.3;XP_006723200.1	-4.4
BORCS8	BLOC-1 related complex subunit 8	protein_coding	NP_001139255.1;NP_001139256.1	-4.4
CUEDC1	CUE domain containing 1	protein_coding	NP_001258804.1;NP_001278954.1;	-4.4
FKRP	fukutin related protein	protein_coding	NP_001034974.1;NP_077277.1;XP_	-4.4
GLTP	glycolipid transfer protein	protein_coding	NP_057517.1;XP_011536736.1	-4.4
CDKN2D	cyclin dependent kinase inhibitor 2D	protein_coding	NP_001791.1;NP_524145.1	-4.4
VAMP2	vesicle associated membrane protein 2	protein_coding	NP_001317054.1;NP_055047.2	-4.4
C14orf132	chromosome 14 open reading frame 132	protein_coding	NP_001239436.1;NP_001269392.1;	-4.4
SPHK1	sphingosine kinase 1	protein_coding	NP_001136073.1;NP_001136074.1;	-4.4
TRNN	tRNA-Asn	tRNA	.	-4.4
ATP6V0B	ATPase H+ transporting V0 subunit b	protein_coding	NP_001034546.1;NP_001281262.1;	-4.4
MLLT6	MLLT6, PHD finger containing	protein_coding	NP_005928.2	-4.4
AJUBA	ajuba LIM protein	protein_coding	NP_001276026.1;NP_116265.1;NP_	-4.4
LINC00537	long intergenic non-protein coding RNA 537	lncRNA	.	-4.4
MARCKS	myristoylated alanine rich protein kinase C substra	protein_coding	NP_002347.5	-4.4
LAMTOR1	late endosomal/lysosomal adaptor, MAPK and MT	protein_coding	NP_060377.1	-4.4
PPP2R3B	protein phosphatase 2 regulatory subunit B"beta	protein_coding	NP_037371.2;XP_011544479.1	-4.4
IFFO1	intermediate filament family orphan 1	protein_coding	NP_001034759.1;NP_001180386.1;	-4.4
HNRNPUL1	heterogeneous nuclear ribonucleoprotein U like 1	protein_coding	NP_001287945.1;NP_001308137.1;	-4.4
ZNF576	zinc finger protein 576	protein_coding	NP_001138819.1;NP_077303.1	-4.4
RHOC	ras homolog family member C	protein_coding	NP_001036143.1;NP_001036144.1;	-4.4
WASF4P	WASP family member 4, pseudogene	pseudogene	.	-4.4
FLJ20021	uncharacterized LOC90024	lncRNA	.	-4.4
CAD	carbamoyl-phosphate synthetase 2, aspartate trans	protein_coding	NP_001293008.1;NP_004332.2;XP_	-4.4

MTCH1	mitochondrial carrier 1	protein_coding	NP_001258570.1;NP_055156.1;XP_	-4.4
PFKP	phosphofructokinase, platelet	protein_coding	NP_001229268.1;NP_001309996.1;	-4.4
EEF2KMT	eukaryotic elongation factor 2 lysine methyltransfe	protein_coding	NP_001275958.1;NP_958802.1;NP_	-4.4
TMEM104	transmembrane protein 104	protein_coding	NP_001308193.1;NP_060198.3;XP_	-4.4
SPTB	spectrin beta, erythrocytic	protein_coding	NP_001020029.1;NP_001342365.1;	-4.4
PPP1R26	protein phosphatase 1 regulatory subunit 26	protein_coding	NP_055626.3;XP_005263468.1;XP_	-4.4
NT5M	5',3'-nucleotidase, mitochondrial	protein_coding	NP_064586.1;XP_005256788.1;XP_	-4.4
AKR7A2	aldo-keto reductase family 7 member A2	protein_coding	NP_001307908.1;NP_003680.2	-4.4
CREG2	cellular repressor of E1A stimulated genes 2	protein_coding	NP_722578.1;XP_011509079.1;XP_	-4.4
KTI12	KTI12 chromatin associated homolog	protein_coding	NP_612426.1	-4.4
SH3BP5L	SH3 binding domain protein 5 like	protein_coding	NP_001309391.1;NP_001309392.1;	-4.4
FBXO17	F-box protein 17	protein_coding	NP_079183.4;NP_680474.1	-4.4
RUNX3	RUNX family transcription factor 3	protein_coding	NP_001026850.1;NP_001307601.1;	-4.4
SPSB4	splA/ryanodine receptor domain and SOCS box cc	protein_coding	NP_543138.1;XP_016862998.1	-4.4
ZNRF3	zinc and ring finger 3	protein_coding	NP_001193927.1;NP_115549.2;XP_	-4.4
BCL2L1	BCL2 like 1	protein_coding	NP_001182.1;NP_001304848.1;NP_	-4.4
STON2	stonin 2	protein_coding	NP_001243359.1;NP_001353778.1;	-4.4
EXOSC10-AS1	EXOSC10 antisense RNA 1	lncRNA	.	-4.4
AAAS	aladin WD repeat nucleoporin	protein_coding	NP_001166937.1;NP_056480.1;XP_	-4.5
TRIOBP	TRIO and F-actin binding protein	protein_coding	NP_001034230.1;NP_008963.3;NP_	-4.5
SLC66A1	solute carrier family 66 member 1	protein_coding	NP_001035214.1;NP_001035215.1;	-4.5
ESPL1	extra spindle pole bodies like 1, separase	protein_coding	NP_036423.4;XP_006719768.1;XP_	-4.5
LOC102724135	uncharacterized LOC102724135	ranscribed_pseudogen	.	-4.5
DEDD2	death effector domain containing 2	protein_coding	NP_001257543.1;NP_001257544.1;	-4.5
MRM1	mitochondrial rRNA methyltransferase 1	protein_coding	NP_079140.2;XP_005257751.1;XP_	-4.5
ADPRS	ADP-ribosylserine hydrolase	protein_coding	NP_060295.1;XP_011539938.1	-4.5
AHRR	aryl-hydrocarbon receptor repressor	protein_coding	NP_001364165.1;NP_001364168.1	-4.5
TRIP10	thyroid hormone receptor interactor 10	protein_coding	NP_001275891.1;NP_001275892.1;	-4.5
WASH6P	WASP family homolog 6, pseudogene	pseudogene	.	-4.5
PRELID1P6	PRELID1 pseudogene 6	pseudogene	.	-4.5
ETHE1	ETHE1 persulfide dioxygenase	protein_coding	NP_001307796.1;NP_001307797.1;	-4.5
C19orf54	chromosome 19 open reading frame 54	protein_coding	NP_001340734.1;NP_001340735.1;	-4.5
ROM1	retinal outer segment membrane protein 1	protein_coding	NP_000318.2	-4.5
ZBTB17	zinc finger and BTB domain containing 17	protein_coding	NP_001229813.1;NP_001274532.1;	-4.5
CSRP1	cysteine and glycine rich protein 1	protein_coding	NP_001180499.1;NP_001180500.1;	-4.5
TREX1	three prime repair exonuclease 1	protein_coding	NP_009179.2;NP_338599.1	-4.5
AARS2	alanyl-tRNA synthetase 2, mitochondrial	protein_coding	NP_065796.2;XP_005249302.1;XP_	-4.5
PDE1A	phosphodiesterase 1A	protein_coding	NP_001003683.1;NP_001245241.1;	-4.5
RN7SL3	RNA component of signal recognition particle 7SI	scRNA	.	-4.5
PPOX	protoporphyrinogen oxidase	protein_coding	NP_000300.1;NP_001116236.1;NP_	-4.5
MIF4GD	MIF4G domain containing	protein_coding	NP_001229427.1;NP_001229429.1;	-4.5
ATP6V0D1	ATPase H+ transporting V0 subunit d1	protein_coding	NP_004682.2;XP_011521746.1;XP_	-4.5
STX1B	syntaxin 1B	protein_coding	NP_443106.1;XP_016878382.1	-4.5
MED22	mediator complex subunit 22	protein_coding	NP_598395.1;NP_852468.1	-4.5
PCDHGB1	protocadherin gamma subfamily B, 1	protein_coding	NP_061745.1;NP_115266.1	-4.5
CPNE2	copine 2	protein_coding	NP_689940.3	-4.5
COLGALT1	collagen beta(1-O)galactosyltransferase 1	protein_coding	NP_078932.2;XP_005260137.1;XP_	-4.5
SLC8B1	solute carrier family 8 member B1	protein_coding	NP_001317395.1;NP_001345274.1;	-4.5
CYFIP2	cytoplasmic FMR1 interacting protein 2	protein_coding	NP_001032410.1;NP_001278650.1;	-4.5
ATG101	autophagy related 101	protein_coding	NP_001092143.1;NP_068753.2;XP	-4.5
INPP5E	inositol polyphosphate-5-phosphatase E	protein_coding	NP_001305431.1;NP_063945.2;XP	-4.5
CYTH3	cytohesin 3	protein_coding	NP_001354509.1;NP_001354510.1;	-4.5
GLI2	GLI family zinc finger 2	protein_coding	NP_001358200.1;NP_001361282.1;	-4.5
SNAP47	synaptosome associated protein 47	protein_coding	NP_001310859.1;NP_001310860.1;	-4.5
TBC1D28	TBC1 domain family member 28	protein_coding	NP_001034486.2;XP_011522083.1;	-4.5
TRIM62	tripartite motif containing 62	protein_coding	NP_001317412.1;NP_060677.2;XP_	-4.5
CLCN2	chloride voltage-gated channel 2	protein_coding	NP_001164558.1;NP_001164559.1;	-4.5
CBR1	carbonyl reductase 1	protein_coding	NP_001273718.1;NP_001748.1	-4.5
PCBP1	poly(rC) binding protein 1	protein_coding	NP_006187.2	-4.5
DNASE1L1	deoxyribonuclease 1 like 1	protein_coding	NP_001009932.1;NP_001009933.1;	-4.5
POM121C	POM121 transmembrane nucleoporin C	protein_coding	NP_001092885.2	-4.5
PPP1R14BP3	protein phosphatase 1 regulatory inhibitor subunit	pseudogene	.	-4.5
RNF123	ring finger protein 123	protein_coding	NP_071347.2;XP_006713348.1;XP_	-4.5
SLC6A9	solute carrier family 6 member 9	protein_coding	NP_001020016.1;NP_001248309.1;	-4.5
TAZ	tafazzin	protein_coding	NP_000107.1;NP_001290394.1;NP_	-4.5
SPR	sepiapterin reductase	protein_coding	NP_003115.1	-4.5
TMEM120A	transmembrane protein 120A	protein_coding	NP_001304732.1;NP_001350391.1;	-4.5
SCAMP2	secretory carrier membrane protein 2	protein_coding	NP_001307707.1;NP_005688.2;XP_	-4.5
PHF19	PHD finger protein 19	protein_coding	NP_001009936.1;NP_001273769.1;	-4.5
SIGMAR1	sigma non-opioid intracellular receptor 1	protein_coding	NP_001269134.1;NP_001269135.1;	-4.5
CLU	clusterin	protein_coding	NP_001822.3	-4.5

ZBTB11-AS1	ZBTB11 antisense RNA 1	lncRNA	.	-4.5
MID1IP1	MID1 interacting protein 1	protein_coding	NP_001092260.1;NP_001092261.1;	-4.5
ZMYND19	zinc finger MYND-type containing 19	protein_coding	NP_612471.1;XP_005266109.1;XP_	-4.5
ATXN2L	ataxin 2 like	protein_coding	NP_001295159.1;NP_009176.2;NP_	-4.5
SLC37A4	solute carrier family 37 member 4	protein_coding	NP_001157749.1;NP_001157750.1;	-4.5
PHF13	PHD finger protein 13	protein_coding	NP_722519.2;XP_011539064.1	-4.5
GSG1	germ cell associated 1	protein_coding	NP_001074023.1;NP_001074024.1;	-4.6
NIPSNAP1	nipsnap homolog 1	protein_coding	NP_001189431.1;NP_003625.2	-4.6
PHPT1	phosphohistidine phosphatase 1	protein_coding	NP_001129333.1;NP_001274271.1;	-4.6
FBLIM1	filamin binding LIM protein 1	protein_coding	NP_001019386.1;NP_001019387.1;	-4.6
ABHD16A	abhydrolase domain containing 16A, phospholipas	protein_coding	NP_001170986.1;NP_066983.1	-4.6
PCDHGA12	protocadherin gamma subfamily A, 12	protein_coding	NP_003726.1;NP_115265.1	-4.6
TSPOAP1-AS1	TSPOAP1, SUPT4H1 and RNF43 antisense RNA	lncRNA	.	-4.6
IRF7	interferon regulatory factor 7	protein_coding	NP_001563.2;NP_004020.1;NP_00	-4.6
ZNF815P	zinc finger protein 815, pseudogene	ranscribed_pseudogen	.	-4.6
CANT1	calcium activated nucleotidase 1	protein_coding	NP_001153244.1;NP_001153245.1;	-4.6
JMJD7	jumonji domain containing 7	protein_coding	NP_001108104.1	-4.6
LINC00619	long intergenic non-protein coding RNA 619	lncRNA	.	-4.6
KPTN	kaptin, actin binding protein	protein_coding	NP_001278225.1;NP_008990.2;XP_	-4.6
GPI	glucose-6-phosphate isomerase	protein_coding	NP_000166.2;NP_001171651.1;NP_	-4.6
SLC25A29	solute carrier family 25 member 29	protein_coding	NP_001034444.1;NP_001278742.1;	-4.6
LOC105379152	uncharacterized LOC105379152	lncRNA	.	-4.6
CERK	ceramide kinase	protein_coding	NP_073603.2;XP_016884398.1	-4.6
MCM2	minichromosome maintenance complex componer	protein_coding	NP_004517.2;XP_024309299.1	-4.6
GMPPB	GDP-mannose pyrophosphorylase B	protein_coding	NP_037466.2;NP_068806.2	-4.6
POMGNT2	protein O-linked mannose N-acetylglucosaminyltr	protein_coding	NP_116195.2;XP_005265572.1;XP_	-4.6
TMEM132A	transmembrane protein 132A	protein_coding	NP_060340.2;NP_821174.1;XP_01	-4.6
ACTG1P25	actin gamma 1 pseudogene 25	ranscribed_pseudogen	.	-4.6
TPST2	tyrosylprotein sulfotransferase 2	protein_coding	NP_001008566.1;NP_001349851.1;	-4.6
STOML1	stomatin like 1	protein_coding	NP_001243601.1;NP_001243602.1;	-4.6
LOC440300	chondroitin sulfate proteoglycan 4 pseudogene	ranscribed_pseudogen	.	-4.6
TMEM121B	transmembrane protein 121B	protein_coding	NP_001156551.1;NP_114096.1;XP_	-4.6
TCIRG1	T cell immune regulator 1, ATPase H+ transportin	protein_coding	NP_001337988.1;NP_006010.2;NP_	-4.6
KIF22	kinesin family member 22	protein_coding	NP_001243198.1;NP_001243199.1;	-4.6
DTX4	deltex E3 ubiquitin ligase 4	protein_coding	NP_001287656.1;NP_055992.1;XP_	-4.6
TROAP	trophinin associated protein	protein_coding	NP_001094090.1;NP_001265253.1;	-4.6
IDUA	alpha-L-iduronidase	protein_coding	NP_000194.2;NP_001350505.1;XP_	-4.6
POLL	DNA polymerase lambda	protein_coding	NP_001167555.1;NP_001167556.1;	-4.6
H3P16	H3 histone pseudogene 16	pseudogene	.	-4.6
LMAN2	lectin, mannose binding 2	protein_coding	NP_006807.1	-4.6
NDUFAF3	NADH:ubiquinone oxidoreductase complex assem	protein_coding	NP_951032.1;NP_951033.1;NP_95	-4.6
SIDT2	SID1 transmembrane family member 2	protein_coding	NP_001035545.1	-4.6
MOB2	MOB kinase activator 2	protein_coding	NP_001165694.1;NP_443731.2	-4.6
LINC00863	long intergenic non-protein coding RNA 863	lncRNA	.	-4.6
DPM2	dolichyl-phosphate mannosyltransferase subunit 2	protein_coding	NP_001365365.1;NP_001365366.1;	-4.7
ENTPD6	ectonucleoside triphosphate diphosphohydrolase 6	protein_coding	NP_001107561.1;NP_001238.3;NP_	-4.7
PRCC	proline rich mitotic checkpoint control factor	protein_coding	NP_005964.3;XP_005245370.1;XP_	-4.7
GUCD1	guanylyl cyclase domain containing 1	protein_coding	NP_001271180.1;NP_001271181.1;	-4.7
SMARCA4	SWI/SNF related, matrix associated, actin depende	protein_coding	NP_001122316.1;NP_001122317.1;	-4.7
GCSHP5	glycine cleavage system protein H pseudogene 5	pseudogene	.	-4.7
ATP6AP1	ATPase H+ transporting accessory protein 1	protein_coding	NP_001174.2;XP_011529481.1	-4.7
TSPAN2	tetraspanin 2	protein_coding	NP_001295244.1;NP_001295245.1;	-4.7
ACTN1	actinin alpha 1	protein_coding	NP_001093.1;NP_001123476.1;NP_	-4.7
CAPG	capping actin protein, gelsolin like	protein_coding	NP_001243068.1;NP_001243069.1;	-4.7
GNAO1	G protein subunit alpha o1	protein_coding	NP_066268.1;NP_620073.2;XP_01	-4.7
PITPNM2	phosphatidylinositol transfer protein membrane as	protein_coding	NP_001287730.1;NP_065896.1;XP_	-4.7
UBAC1	UBA domain containing 1	protein_coding	NP_057256.2	-4.7
LINC00685	long intergenic non-protein coding RNA 685	lncRNA	.	-4.7
CFL1	cofilin 1	protein_coding	NP_005498.1	-4.7
NAPA	NSF attachment protein alpha	protein_coding	NP_003818.2;XP_011525738.1;XP_	-4.7
MRPL49	mitochondrial ribosomal protein L49	protein_coding	NP_004918.1	-4.7
SF3B4	splicing factor 3b subunit 4	protein_coding	NP_005841.1	-4.7
SKIDA1	SKI/DACH domain containing 1	protein_coding	NP_997254.3;XP_005252507.1;XP_	-4.7
GTF2F1	general transcription factor IIF subunit 1	protein_coding	NP_002087.2;XP_024307243.1	-4.7
SART1	spliceosome associated factor 1, recruiter of U4/U	protein_coding	NP_005137.1;XP_011543646.1;XP_	-4.7
AP2A2	adaptor related protein complex 2 subunit alpha 2	protein_coding	NP_001229766.1;NP_036437.1;XP_	-4.7
PHF2	PHD finger protein 2	protein_coding	NP_005383.3;XP_005252108.1;XP_	-4.7
PKIG	cAMP-dependent protein kinase inhibitor gamma	protein_coding	NP_001268373.1;NP_001268374.1;	-4.7
LMF1	lipase maturation factor 1	protein_coding	NP_001338946.1;NP_001338947.1;	-4.7
WFDC21P	WAP four-disulfide core domain 21, pseudogene	ranscribed_pseudogen	.	-4.7
MAPKAPK3	MAPK activated protein kinase 3	protein_coding	NP_001230854.1;NP_001230855.1;	-4.7

RPL18	ribosomal protein L18	protein_coding	NP_000970.1;NP_001257419.1	-4.7
SF3A1	splicing factor 3a subunit 1	protein_coding	NP_005868.1	-4.7
MARCHF9	membrane associated ring-CH-type finger 9	protein_coding	NP_612405.2	-4.7
MGAT5B	alpha-1,6-mannosylglycoprotein 6-beta-N-acetylg	protein_coding	NP_001186101.1;NP_653278.2;NP_	-4.7
TIMM13	translocase of inner mitochondrial membrane 13	protein_coding	NP_036590.1	-4.7
NUP62	nucleoporin 62	protein_coding	NP_001180286.1;NP_036478.2;NP_	-4.7
PCDHB9	protocadherin beta 9	protein_coding	NP_061992.3	-4.7
ZNF593	zinc finger protein 593	protein_coding	NP_056955.2	-4.7
PYGO2	pygopus family PHD finger 2	protein_coding	NP_612157.1	-4.7
TBRG4	transforming growth factor beta regulator 4	protein_coding	NP_001248763.1;NP_004740.2;NP_	-4.7
ALG3	ALG3 alpha-1,3- mannosyltransferase	protein_coding	NP_001006942.1;NP_005778.1;XP_	-4.7
CDK16	cyclin dependent kinase 16	protein_coding	NP_001163931.1;NP_006192.1;NP_	-4.7
SLC25A25	solute carrier family 25 member 25	protein_coding	NP_001006642.1;NP_001006643.1;	-4.8
ECH1	enoyl-CoA hydratase 1	protein_coding	NP_001389.2;XP_016881937.1;XP_	-4.8
ILF3-DT	ILF3 divergent transcript	lncRNA	.	-4.8
PAQR7	progestin and adipoQ receptor family member 7	protein_coding	NP_848509.1;XP_005245803.1;XP_	-4.8
FEM1A	fem-1 homolog A	protein_coding	NP_061178.1	-4.8
TIMP2	TIMP metallopeptidase inhibitor 2	protein_coding	NP_003246.1	-4.8
CCM2	CCM2 scaffold protein	protein_coding	NP_001025006.1;NP_001161406.1;	-4.8
BLVRB	biliverdin reductase B	protein_coding	NP_000704.1	-4.8
C19orf48	chromosome 19 open reading frame 48	protein_coding	NP_001277078.1;NP_001277079.1;	-4.8
INCENP	inner centromere protein	protein_coding	NP_001035784.1;NP_064623.2;XP_	-4.8
SPACA6P-AS	SPACA6P antisense RNA	lncRNA	.	-4.8
BIRC5	baculoviral IAP repeat containing 5	protein_coding	NP_001012270.1;NP_001012271.1;	-4.8
PCDHGA9	protocadherin gamma subfamily A, 9	protein_coding	NP_061744.1;NP_114478.1	-4.8
ELAPOR1	endosome-lysosome associated apoptosis and auto	protein_coding	NP_001253977.2;NP_001271281.1;	-4.8
IER2	immediate early response 2	protein_coding	NP_004898.2	-4.8
H2BC10	H2B clustered histone 10	protein_coding	NP_003516.1	-4.8
B9D1	B9 domain containing 1	protein_coding	NP_001230402.1;NP_001308143.1;	-4.8
PATZ1	POZ/BTB and AT hook containing zinc finger 1	protein_coding	NP_055138.2;NP_114439.1;NP_11	-4.8
ASPHD2	aspartate beta-hydroxylase domain containing 2	protein_coding	NP_065170.2	-4.8
IRGQ	immunity related GTPase Q	protein_coding	NP_001007562.1;XP_005258572.1	-4.8
PRR14	proline rich 14	protein_coding	NP_001307393.1;NP_076936.1	-4.8
CD2BP2-DT	CD2BP2 divergent transcript, transcript variant X	lncRNA	.	-4.8
CYSRT1	cysteine rich tail 1	protein_coding	NP_945352.4	-4.8
ABCB6	ATP binding cassette subfamily B member 6 (Lan	protein_coding	NP_001336757.1;NP_005680.1	-4.8
PLEKHF1	pleckstrin homology and FYVE domain containin	protein_coding	NP_077286.3;XP_005259313.1;XP_	-4.8
MYH9	myosin heavy chain 9	protein_coding	NP_002464.1	-4.8
SPPL2B	signal peptide peptidase like 2B	protein_coding	NP_001070706.1;NP_694533.1;XP_	-4.8
SMARCB1	SWI/SNF related, matrix associated, actin depend	protein_coding	NP_001007469.1;NP_001304875.1;	-4.8
UBE2J2	ubiquitin conjugating enzyme E2 J2	protein_coding	NP_477515.2;NP_919296.1;NP_91	-4.8
LINC00205	long intergenic non-protein coding RNA 205	lncRNA	.	-4.8
SPACA9	sperm acrosome associated 9	protein_coding	NP_001303826.1;NP_001303827.1;	-4.8
ABTB1	ankyrin repeat and BTB domain containing 1	protein_coding	NP_115937.1;NP_742024.1;XP_00	-4.8
KMT2D	lysine methyltransferase 2D	protein_coding	NP_003473.3;XP_006719677.1;XP_	-4.8
TUBA3FP	tubulin alpha 3f pseudogene	ranscribed_pseudogen	.	-4.8
PRMT6	protein arginine methyltransferase 6	protein_coding	NP_060607.2	-4.8
PLEKHO1	pleckstrin homology domain containing O1	protein_coding	NP_001291651.1;NP_001291652.1;	-4.8
TRIM41	tripartite motif containing 41	protein_coding	NP_291027.3;NP_963921.1;XP_00	-4.8
ASL	argininosuccinate lyase	protein_coding	NP_000039.2;NP_001020114.1;NP_	-4.8
LOC613038	SAGA complex associated factor 29 pseudogene	ranscribed_pseudogen	.	-4.8
ABHD11	abhydrolase domain containing 11	protein_coding	NP_001138836.1;NP_001287987.1;	-4.8
AIP	aryl hydrocarbon receptor interacting protein	protein_coding	NP_001289888.1;NP_001289889.1;	-4.8
H3C12	H3 clustered histone 12	protein_coding	NP_003526.1	-4.8
UBL7	ubiquitin like 7	protein_coding	NP_001273668.1;NP_001273669.1;	-4.8
TMEM86A	transmembrane protein 86A	protein_coding	NP_699178.1	-4.8
PMVK	phosphomevalonate kinase	protein_coding	NP_001309940.1;NP_001309941.1;	-4.9
ADGRE5	adhesion G protein-coupled receptor E5	protein_coding	NP_001020331.1;NP_001775.2;NP_	-4.9
CYP27A1	cytochrome P450 family 27 subfamily A member	protein_coding	NP_000775.1;XP_016858977.1	-4.9
COQ4	coenzyme Q4	protein_coding	NP_001292871.2;NP_057119.3;XP_	-4.9
PXMP4	peroxisomal membrane protein 4	protein_coding	NP_009169.3;NP_899634.1	-4.9
ZBED1	zinc finger BED-type containing 1	protein_coding	NP_001164606.1;NP_001164607.1;	-4.9
LIX1L	limb and CNS expressed 1 like	protein_coding	NP_714924.1;XP_016855805.1	-4.9
PLCXD1	phosphatidylinositol specific phospholipase C X d	protein_coding	NP_001357299.1;NP_001357300.1;	-4.9
SPSB1	splA/ryanodine receptor domain and SOCS box cc	protein_coding	NP_079382.2	-4.9
TOM1L2	target of myb1 like 2 membrane trafficking protei	protein_coding	NP_001028723.1;NP_001076437.1;	-4.9
MIDEAS	mitotic deacetylase associated SANT domain prot	protein_coding	NP_001036783.1;NP_001354639.1;	-4.9
CDK18	cyclin dependent kinase 18	protein_coding	NP_002587.2;NP_997667.1;NP_99	-4.9
BTBD6	BTB domain containing 6	protein_coding	NP_150374.2;XP_005268245.1	-4.9
THAP3	THAP domain containing 3	protein_coding	NP_001182681.1;NP_001182682.1;	-4.9
FZD1	frizzled class receptor 1	protein_coding	NP_003496.1	-4.9

CYB561A3	cytochrome b561 family member A3	protein_coding	NP_001154924.1;NP_001154926.1;	-4.9
PPP1R18	protein phosphatase 1 regulatory subunit 18	protein_coding	NP_001128342.1;NP_597728.1	-4.9
LAMP1	lysosomal associated membrane protein 1	protein_coding	NP_005552.3;XP_011535796.1	-4.9
TAPBP	TAP binding protein	protein_coding	NP_003181.3;NP_757345.2;NP_75'	-4.9
RANBP3	RAN binding protein 3	protein_coding	NP_001287794.1;NP_003615.2;NP_	-4.9
FAM219A	family with sequence similarity 219 member A	protein_coding	NP_001171869.1;NP_001171870.1;	-4.9
DPP3	dipeptidyl peptidase 3	protein_coding	NP_001243599.1;NP_005691.2;NP_	-4.9
LIN37	lin-37 DREAM MuvB core complex component	protein_coding	NP_001356709.1;NP_061977.1	-4.9
PXMP2	peroxisomal membrane protein 2	protein_coding	NP_061133.1	-4.9
LRRC8A	leucine rich repeat containing 8 VRAC subunit A	protein_coding	NP_001120716.1;NP_001120717.1;	-4.9
HOXC4	homeobox C4	protein_coding	NP_055435.2;NP_705897.1	-4.9
B3GALT4	beta-1,3-galactosyltransferase 4	protein_coding	NP_003773.1	-4.9
ETFB	electron transfer flavoprotein subunit beta	protein_coding	NP_001014763.1;NP_001976.1;XP_	-4.9
GNAI2	G protein subunit alpha i2	protein_coding	NP_001159897.1;NP_001269546.1;	-4.9
APEH	acylaminoacyl-peptide hydrolase	protein_coding	NP_001631.3;XP_005265154.1;XP_	-4.9
CLSTN1	calsyntenin 1	protein_coding	NP_001009566.1;NP_001289812.1;	-4.9
POLDIP2	DNA polymerase delta interacting protein 2	protein_coding	NP_001277074.1;NP_056399.1	-4.9
TIMM44	translocase of inner mitochondrial membrane 44	protein_coding	NP_006342.2	-4.9
BMP8B	bone morphogenetic protein 8b	protein_coding	NP_001711.2;XP_005271206.1;XP_	-4.9
B3GNT3	UDP-GlcNAc:betaGal beta-1,3-N-acetylglucosam	protein_coding	NP_055071.2;XP_011525928.1	-4.9
RTKN	rhotekin	protein_coding	NP_001015055.1;NP_001015056.1;	-4.9
GTSE1	G2 and S-phase expressed 1	protein_coding	NP_057510.5;XP_005261684.1;XP_	-4.9
WASH4P	WASP family homolog 4, pseudogene	pseudogene	.	-4.9
TRP-AGG2-6	tRNA-Pro	tRNA	.	-4.9
TRNP1	TMF1 regulated nuclear protein 1	protein_coding	NP_001013664.2;XP_005245924.1	-4.9
UQCRC1	ubiquinol-cytochrome c reductase core protein 1	protein_coding	NP_003356.2	-4.9
FAM117A	family with sequence similarity 117 member A	protein_coding	NP_110429.1;XP_016880671.1	-4.9
DOLK	dolichol kinase	protein_coding	NP_055723.1	-4.9
CENPM	centromere protein M	protein_coding	NP_001002876.1;NP_001103685.1;	-5.0
LRRC47	leucine rich repeat containing 47	protein_coding	NP_065761.1	-5.0
NFKBIL1	NFKB inhibitor like 1	protein_coding	NP_001138433.1;NP_001138434.1;	-5.0
MCM5	minichromosome maintenance complex componer	protein_coding	NP_006730.2;XP_006724305.1	-5.0
COMMD5	COMM domain containing 5	protein_coding	NP_001074472.1;NP_001074473.1;	-5.0
STK40	serine/threonine kinase 40	protein_coding	NP_001269475.1;NP_001269476.1;	-5.0
CD70	CD70 molecule	protein_coding	NP_001243.1;NP_001317261.1	-5.0
CMIP	c-Maf inducing protein	protein_coding	NP_085132.1;NP_938204.2;XP_00:	-5.0
RING1	ring finger protein 1	protein_coding	NP_002922.2	-5.0
EFR3B	EFR3 homolog B	protein_coding	NP_001306028.1;NP_055786.1;XP_	-5.0
BABAM1	BRISC and BRCA1 A complex member 1	protein_coding	NP_001028721.1;NP_001275685.1;	-5.0
SZRD1	SUZ RNA binding domain containing 1	protein_coding	NP_001108072.1;NP_001258798.1	-5.0
FOXM1	forkhead box M1	protein_coding	NP_001230017.1;NP_001230018.1;	-5.0
A1BG	alpha-1-B glycoprotein	protein_coding	NP_570602.2	-5.0
RARRES2	retinoic acid receptor responder 2	protein_coding	NP_002880.1;XP_016867980.1	-5.0
TLE3	TLE family member 3, transcriptional corepressor	protein_coding	NP_001098662.1;NP_001269908.1;	-5.0
ARPC1B	actin related protein 2/3 complex subunit 1B	protein_coding	NP_005711.1;XP_006715888.1;XP_	-5.0
PINK1	PTEN induced kinase 1	protein_coding	NP_115785.1	-5.0
PLEKHB1	pleckstrin homology domain containing B1	protein_coding	NP_001123505.1;NP_001123506.1;	-5.0
C17orf97	chromosome 17 open reading frame 97	protein_coding	NP_001013694.4	-5.0
RAB34	RAB34, member RAS oncogene family	protein_coding	NP_001136096.2;NP_001136097.2;	-5.0
KLHL21	kelch like family member 21	protein_coding	NP_001311238.1;NP_055666.2	-5.0
RAB5C	RAB5C, member RAS oncogene family	protein_coding	NP_001238968.1;NP_004574.2;NP_	-5.0
LRP1	LDL receptor related protein 1	protein_coding	NP_002323.2;XP_016874792.1	-5.0
LOC105371912	uncharacterized LOC105371912	lncRNA	.	-5.0
H4C2	H4 clustered histone 2	protein_coding	NP_003535.1	-5.0
CMTM3	CKLF like MARVEL transmembrane domain con	protein_coding	NP_001350847.1;NP_001350852.1;	-5.0
FTH1P7	ferritin heavy chain 1 pseudogene 7	pseudogene	.	-5.0
FITM2	fat storage inducing transmembrane protein 2	protein_coding	NP_001073941.1	-5.0
CCAR2	cell cycle and apoptosis regulator 2	protein_coding	NP_001349997.1;NP_001349998.1;	-5.0
KIAA0513	KIAA0513	protein_coding	NP_001273494.1;NP_001273495.1;	-5.0
PRAF2	PRA1 domain family member 2	protein_coding	NP_009144.1	-5.0
CES2	carboxylesterase 2	protein_coding	NP_001352334.1;NP_001352335.1;	-5.0
C7orf26	chromosome 7 open reading frame 26	protein_coding	NP_001289968.1;NP_076972.2;XP_	-5.0
RELL2	RELT like 2	protein_coding	NP_001123501.1;NP_776189.3;XP_	-5.0
ZNF324B	zinc finger protein 324B	protein_coding	NP_997278.2;XP_005258972.1;XP_	-5.0
TMEM38A	transmembrane protein 38A	protein_coding	NP_076979.1	-5.0
AK1	adenylate kinase 1	protein_coding	NP_000467.1;NP_001305050.1;NP_	-5.0
RAB40B	RAB40B, member RAS oncogene family	protein_coding	NP_006813.1;XP_005256391.1;XP_	-5.0
USP22	ubiquitin specific peptidase 22	protein_coding	NP_056091.1;XP_005256632.1	-5.1
CDK5	cyclin dependent kinase 5	protein_coding	NP_001157882.1;NP_004926.1	-5.1
SH3PXD2B	SH3 and PX domains 2B	protein_coding	NP_001017995.1;NP_001295104.1;	-5.1
CST3	cystatin C	protein_coding	NP_000090.1;NP_001275543.1	-5.1

LAMB2	laminin subunit beta 2	protein_coding	NP_002283.3;XP_005265184.1	-5.1
TMEM222	transmembrane protein 222	protein_coding	NP_115501.2	-5.1
TAF6L	TATA-box binding protein associated factor 6 like	protein_coding	NP_006464.1;XP_005273771.1;XP_	-5.1
FSCN2	fascin actin-bundling protein 2, retinal	protein_coding	NP_001070650.1;NP_036550.1;XP_	-5.1
KCNAB2	potassium voltage-gated channel subfamily A regu	protein_coding	NP_001186789.1;NP_001186790.1;	-5.1
LOC105372952	uncharacterized LOC105372952, transcript varian	lncRNA	.	-5.1
TSKU	tsukushi, small leucine rich proteoglycan	protein_coding	NP_001245139.1;NP_001305406.1;	-5.1
STIM1	stromal interaction molecule 1	protein_coding	NP_001264890.1;NP_001264891.1;	-5.1
CCDC183-AS1	CCDC183 antisense RNA 1	lncRNA	.	-5.1
EBI3	Epstein-Barr virus induced 3	protein_coding	NP_005746.2;XP_011525921.1	-5.1
TBC1D13	TBC1 domain family member 13	protein_coding	NP_001273701.1;NP_060671.3;XP_	-5.1
NUTM2D	NUT family member 2D	protein_coding	NP_001009610.1;NP_001369233.1	-5.1
ZNF653	zinc finger protein 653	protein_coding	NP_620138.2	-5.1
HOXC9	homeobox C9	protein_coding	NP_008828.1	-5.1
SNORD3A	small nucleolar RNA, C/D box 3A	snoRNA	.	-5.1
GCDH	glutaryl-CoA dehydrogenase	protein_coding	NP_000150.1;NP_039663.1	-5.1
ABHD14B	abhydrolase domain containing 14B	protein_coding	NP_001139786.1;NP_001241682.1;	-5.1
LRRC20	leucine rich repeat containing 20	protein_coding	NP_001265140.1;NP_001265141.1;	-5.1
AP5B1	adaptor related protein complex 5 subunit beta 1	protein_coding	NP_612377.4	-5.1
GMEB2	glucocorticoid modulatory element binding protei	protein_coding	NP_036516.1;XP_005260259.1;XP_	-5.1
ADRB1	adrenoceptor beta 1	protein_coding	NP_000675.1	-5.1
ZNF672	zinc finger protein 672	protein_coding	NP_079112.1;XP_005270393.1	-5.1
ALKBH5	alkB homolog 5, RNA demethylase	protein_coding	NP_060228.3	-5.1
RPL18A	ribosomal protein L18a	protein_coding	NP_000971.1	-5.1
BAG3	BAG cochaperone 3	protein_coding	NP_004272.2;XP_005270344.1	-5.1
ACO2	aconitase 2	protein_coding	NP_001089.1;XP_016884301.1;XP_	-5.1
PIM1	Pim-1 proto-oncogene, serine/threonine kinase	protein_coding	NP_001230115.1;NP_002639.1	-5.1
RAPGEF1	Rap guanine nucleotide exchange factor 1	protein_coding	NP_001291204.1;NP_001364864.1;	-5.1
H3C2	H3 clustered histone 2	protein_coding	NP_003528.1	-5.1
TRPT1	tRNA phosphotransferase 1	protein_coding	NP_001028850.2;NP_001153861.1;	-5.1
RPH3AL	rabphilin 3A like (without C2 domains)	protein_coding	NP_001177340.1;NP_001177341.1;	-5.1
FOXN3-AS1	FOXN3 antisense RNA 1	lncRNA	.	-5.1
H2BC6	H2B clustered histone 6	protein_coding	NP_003514.2	-5.1
HAGH	hydroxyacylglutathione hydrolase	protein_coding	NP_001035517.1;NP_001273178.1;	-5.1
CYB561D2	cytochrome b561 family member D2	protein_coding	NP_001278213.1;NP_008953.1	-5.2
ARHGEF37	Rho guanine nucleotide exchange factor 37	protein_coding	NP_001001669.2;XP_005268504.1;	-5.2
SNRNP70	small nuclear ribonucleoprotein U1 subunit 70	protein_coding	NP_001287998.1;NP_003080.2;XP_	-5.2
SLC26A11	solute carrier family 26 member 11	protein_coding	NP_001159819.1;NP_001159820.1;	-5.2
EEF1D	eukaryotic translation elongation factor 1 delta	protein_coding	NP_001123525.3;NP_001123527.1;	-5.2
KLF13	Kruppel like factor 13	protein_coding	NP_001289390.1;NP_057079.2	-5.2
TFE3	transcription factor binding to IGHM enhancer 3	protein_coding	NP_001269071.1;NP_006512.2;XP	-5.2
MED24	mediator complex subunit 24	protein_coding	NP_001072986.1;NP_001254726.1;	-5.2
RIPK4	receptor interacting serine/threonine kinase 4	protein_coding	NP_065690.2	-5.2
RAB11FIP3	RAB11 family interacting protein 3	protein_coding	NP_001135744.2;NP_001357330.1;	-5.2
MEST	mesoderm specific transcript	protein_coding	NP_001240829.1;NP_001240830.1;	-5.2
CTF1	cardiotrophin 1	protein_coding	NP_001136016.1;NP_001321.1;XP	-5.2
ASAP3	ArfGAP with SH3 domain, ankyrin repeat and PH	protein_coding	NP_001137250.1;NP_060177.2;XP_	-5.2
KCTD5	potassium channel tetramerization domain contain	protein_coding	NP_061865.1	-5.2
AXIN1	axin 1	protein_coding	NP_003493.1;NP_851393.1;XP_01	-5.2
HNRNPL	heterogeneous nuclear ribonucleoprotein L	protein_coding	NP_001005335.1;NP_001524.2;XP	-5.2
MSX2	msh homeobox 2	protein_coding	NP_001350555.1;NP_002440.2	-5.2
KLHL17	kelch like family member 17	protein_coding	NP_938073.1;XP_006710663.1;XP_	-5.2
SEMA4C	semaphorin 4C	protein_coding	NP_060259.4;XP_006712669.2;XP_	-5.2
PXDC1	PX domain containing 1	protein_coding	NP_899229.2;XP_011512695.1	-5.2
LOC105371480	uncharacterized LOC105371480	lncRNA	.	-5.2
GIGYF1	GRB10 interacting GYF protein 1	protein_coding	NP_001362688.1;NP_001362689.1;	-5.2
GNPTG	N-acetylglucosamine-1-phosphate transferase subu	protein_coding	NP_115909.1;XP_016879271.1;XP_	-5.2
HMGA1P3	high mobility group AT-hook 1 pseudogene 3	pseudogene	.	-5.2
SMPD4	sphingomyelin phosphodiesterase 4	protein_coding	NP_001164554.1;NP_060221.2;NP_	-5.2
CCDC71	coiled-coil domain containing 71	protein_coding	NP_075054.3	-5.2
WDR24	WD repeat domain 24	protein_coding	NP_115635.1;XP_011521001.1	-5.2
PPME1	protein phosphatase methylesterase 1	protein_coding	NP_001258522.1;NP_057231.1;XP_	-5.2
ZNF526	zinc finger protein 526	protein_coding	NP_001300962.1;NP_597701.1	-5.2
FKBP4	FKBP prolyl isomerase 4	protein_coding	NP_002005.1;XP_011519231.1	-5.2
H2BC17	H2B clustered histone 17	protein_coding	NP_003518.2	-5.2
HNRNPA0	heterogeneous nuclear ribonucleoprotein A0	protein_coding	NP_006796.1	-5.2
SLIT3	slit guidance ligand 3	protein_coding	NP_001258875.2;NP_003053.2;XP_	-5.2
TRIM11	tripartite motif containing 11	protein_coding	NP_660215.1;XP_011542587.1;XP_	-5.2
IGFBP7	insulin like growth factor binding protein 7	protein_coding	NP_001240764.1;NP_001544.1	-5.2
HOXC6	homeobox C6	protein_coding	NP_004494.1;NP_710160.1	-5.2
H2BC13	H2B clustered histone 13	protein_coding	NP_003510.1	-5.2

TSEN54	tRNA splicing endonuclease subunit 54	protein_coding	NP_997229.2	-5.2
POMGNT1	protein O-linked mannose N-acetylglucosaminyltr	protein_coding	NP_001230695.1;NP_001277058.1;	-5.2
MMP25-AS1	MMP25 antisense RNA 1	lncRNA	.	-5.3
TYMSOS	TYMS opposite strand	protein_coding	NP_001012734.2	-5.3
BRD3OS	BRD3 opposite strand	protein_coding	NP_001342185.1	-5.3
LOC100130027	uncharacterized LOC100130027	lncRNA	.	-5.3
VDR	vitamin D receptor	protein_coding	NP_000367.1;NP_001017535.1;NP_	-5.3
MRPL54	mitochondrial ribosomal protein L54	protein_coding	NP_758455.1	-5.3
OAZ1	ornithine decarboxylase antizyme 1	protein_coding	NP_001287949.1;NP_004143.1	-5.3
UHRF1	ubiquitin like with PHD and ring finger domains 1	protein_coding	NP_001041666.1;NP_001276979.1;	-5.3
PSCA	prostate stem cell antigen	protein_coding	NP_005663.2	-5.3
LOC653503	G protein subunit gamma 10 pseudogene	pseudogene	.	-5.3
CEL	carboxyl ester lipase	protein_coding	NP_001798.3	-5.3
FLII	FLII actin remodeling protein	protein_coding	NP_001243193.1;NP_001243194.1;	-5.3
HMGA1	high mobility group AT-hook 1	protein_coding	NP_001306006.1;NP_001306007.1;	-5.3
STK11IP	serine/threonine kinase 11 interacting protein	protein_coding	NP_443134.3;XP_011508828.1;XP_	-5.3
UBE2O	ubiquitin conjugating enzyme E2 O	protein_coding	NP_071349.3;XP_005257624.1;XP_	-5.3
MANEAL	mannosidase endo-alpha like	protein_coding	NP_001026910.1;NP_001106954.1;	-5.3
ADCY9	adenylate cyclase 9	protein_coding	NP_001107.2;XP_005255136.1;XP_	-5.3
CCNO	cyclin O	protein_coding	NP_066970.3	-5.3
IKBKG	inhibitor of nuclear factor kappa B kinase regulato	protein_coding	NP_001093326.2;NP_001093327.1;	-5.3
KIFC3	kinesin family member C3	protein_coding	NP_001123571.1;NP_001123572.1;	-5.3
AP1S1	adaptor related protein complex 1 subunit sigma 1	protein_coding	NP_001274.1	-5.3
ARHGEF19	Rho guanine nucleotide exchange factor 19	protein_coding	NP_694945.2;XP_011539008.1	-5.3
LDLRAP1	low density lipoprotein receptor adaptor protein 1	protein_coding	NP_056442.2;XP_006710622.1;XP_	-5.3
STX10	syntaxin 10	protein_coding	NP_001258538.1;NP_001258539.1;	-5.3
KIF7	kinesin family member 7	protein_coding	NP_940927.2;XP_011519833.1	-5.3
LINC00909	long intergenic non-protein coding RNA 909	lncRNA	.	-5.3
ASB16-AS1	ASB16 antisense RNA 1	lncRNA	.	-5.3
HECTD3	HECT domain E3 ubiquitin protein ligase 3	protein_coding	NP_078878.3;XP_024305571.1	-5.3
ECE1	endothelin converting enzyme 1	protein_coding	NP_001106818.1;NP_001106819.1;	-5.3
LOC100499489	uncharacterized LOC100499489	lncRNA	.	-5.3
MEPCE	methylphosphate capping enzyme	protein_coding	NP_001181919.1;NP_001181920.1;	-5.3
HDHD5	haloacid dehalogenase like hydrolase domain cont	protein_coding	NP_060299.4;NP_149061.1;XP_00	-5.3
VAV2	vav guanine nucleotide exchange factor 2	protein_coding	NP_001127870.1;NP_003362.2;XP_	-5.3
OGDH	oxoglutarate dehydrogenase	protein_coding	NP_001003941.1;NP_001158508.1;	-5.3
TEAD4	TEA domain transcription factor 4	protein_coding	NP_003204.2;NP_958849.1;NP_95	-5.3
IL11RA	interleukin 11 receptor subunit alpha	protein_coding	NP_001136256.1	-5.3
RN7SL2	RNA component of signal recognition particle 7SI	scRNA	.	-5.3
DNM2	dynamin 2	protein_coding	NP_001005360.1;NP_001005361.1;	-5.3
LOC101927093	uncharacterized LOC101927093	lncRNA	.	-5.3
YJU2	YJU2 splicing factor homolog	protein_coding	NP_060544.2	-5.3
CRTC1	CREB regulated transcription coactivator 1	protein_coding	NP_001091952.1;NP_056136.2;XP_	-5.4
MRPL55	mitochondrial ribosomal protein L55	protein_coding	NP_001308213.1;NP_852106.1;NP_	-5.4
RUSC2	RUN and SH3 domain containing 2	protein_coding	NP_001129471.1;NP_001317669.1;	-5.4
TUBGCP2	tubulin gamma complex associated protein 2	protein_coding	NP_001243546.1;NP_001243547.1;	-5.4
H2AC11	H2A clustered histone 11	protein_coding	NP_066408.1	-5.4
TADA3	transcriptional adaptor 3	protein_coding	NP_001265199.1;NP_006345.1;NP_	-5.4
MEX3A	mex-3 RNA binding family member A	protein_coding	NP_001087194.1	-5.4
GRAMD4	GRAM domain containing 4	protein_coding	NP_001353589.1;NP_055939.1;XP_	-5.4
TNFRSF1A	TNF receptor superfamily member 1A	protein_coding	NP_001056.1;NP_001333020.1;NP_	-5.4
ESYT1	extended synaptotagmin 1	protein_coding	NP_001171725.1;NP_056107.1	-5.4
H2AW	H2A.W histone	protein_coding	NP_254280.1	-5.4
TAOK2	TAO kinase 2	protein_coding	NP_001238972.1;NP_004774.1;NP_	-5.4
CCHCR1	coiled-coil alpha-helical rod protein 1	protein_coding	NP_001099033.1;NP_001099034.1;	-5.4
SCAMP3	secretory carrier membrane protein 3	protein_coding	NP_005689.2;NP_443069.1	-5.4
AP1M1	adaptor related protein complex 1 subunit mu 1	protein_coding	NP_001123996.1;NP_115882.1	-5.4
BRD4	bromodomain containing 4	protein_coding	NP_001317313.1;NP_001366220.1;	-5.4
RAB11FIP1	RAB11 family interacting protein 1	protein_coding	NP_001002814.2;NP_079427.4;XP_	-5.4
AP3D1	adaptor related protein complex 3 subunit delta 1	protein_coding	NP_001248755.1;NP_001361728.1;	-5.4
SLC39A4	solute carrier family 39 member 4	protein_coding	NP_001267486.1;NP_001361768.1;	-5.4
ANKRD54	ankyrin repeat domain 54	protein_coding	NP_001336782.1;NP_001350768.1;	-5.4
G6PC3	glucose-6-phosphatase catalytic subunit 3	protein_coding	NP_001306874.1;NP_612396.1;XP_	-5.4
ZBTB12	zinc finger and BTB domain containing 12	protein_coding	NP_862825.1;XP_011512685.2	-5.4
CCDC134	coiled-coil domain containing 134	protein_coding	NP_001291726.1;NP_001369275.1;	-5.4
TEX264	testis expressed 264, ER-phagy receptor	protein_coding	NP_001123356.1;NP_001230654.1;	-5.4
IFFO2	intermediate filament family orphan 2	protein_coding	NP_001129737.1;XP_011538932.1	-5.4
NEIL2	nei like DNA glycosylase 2	protein_coding	NP_001129218.1;NP_001129219.1;	-5.4
SH2B1	SH2B adaptor protein 1	protein_coding	NP_001139267.1;NP_001139268.1;	-5.4
C1QTNF1	C1q and TNF related 1	protein_coding	NP_112230.1;NP_699203.1;NP_94	-5.4
TSNARE1	t-SNARE domain containing 1	protein_coding	NP_001278860.1;NP_001350669.1;	-5.4

PRKD2	protein kinase D2	protein_coding	NP_001073349.1;NP_001073350.1;	-5.4
H3C6	H3 clustered histone 6	protein_coding	NP_001368928.1;NP_003523.1	-5.4
SPIRE2	spire type actin nucleation factor 2	protein_coding	NP_115827.1	-5.4
ACTR1B	actin related protein 1B	protein_coding	NP_005726.1;XP_005263911.1;XP_	-5.4
GET3	guided entry of tail-anchored proteins factor 3, AT	protein_coding	NP_001358417.1;NP_001358418.1;	-5.4
SURF2	surfeit 2	protein_coding	NP_001265857.1;NP_059973.4	-5.4
IGDCC4	immunoglobulin superfamily DCC subclass memb	protein_coding	NP_066013.1;XP_011520147.1;XP_	-5.4
RELA	RELA proto-oncogene, NF-kB subunit	protein_coding	NP_001138610.1;NP_001230913.1;	-5.4
MLXIP	MLX interacting protein	protein_coding	NP_055753.3;XP_006719353.1;XP_	-5.4
ABCB8	ATP binding cassette subfamily B member 8	protein_coding	NP_001269220.1;NP_001269221.1;	-5.4
KANK2	KN motif and ankyrin repeat domains 2	protein_coding	NP_001129663.1;NP_001316380.1;	-5.4
TMED1	transmembrane p24 trafficking protein 1	protein_coding	NP_006849.1;XP_006722694.1	-5.4
PRELID1	PRELI domain containing 1	protein_coding	NP_001258757.1;NP_037369.1	-5.4
PCDHB2	protocadherin beta 2	protein_coding	NP_061759.1	-5.4
TMEM200B	transmembrane protein 200B	protein_coding	NP_001003682.1;NP_001165339.1;	-5.5
CHPF2	chondroitin polymerizing factor 2	protein_coding	NP_001271224.1;NP_061888.1	-5.5
GPSM3	G protein signaling modulator 3	protein_coding	NP_001263430.1;NP_071390.1	-5.5
FANCE	FA complementation group E	protein_coding	NP_068741.1;XP_005248942.1;XP_	-5.5
VANGL2	VANGL planar cell polarity protein 2	protein_coding	NP_065068.1;XP_005245414.1;XP_	-5.5
RNF215	ring finger protein 215	protein_coding	NP_001017981.1	-5.5
IL32	interleukin 32	protein_coding	NP_001012649.1;NP_001012650.1;	-5.5
NDUFA11	NADH:ubiquinone oxidoreductase subunit A11	protein_coding	NP_001180304.1;NP_783313.1	-5.5
RALGDS	ral guanine nucleotide dissociation stimulator	protein_coding	NP_001035827.1;NP_001258703.1;	-5.5
MED26	mediator complex subunit 26	protein_coding	NP_004822.2	-5.5
PPP2R5D	protein phosphatase 2 regulatory subunit B'delta	protein_coding	NP_001257405.1;NP_006236.1;NP_	-5.5
FGFRL1	fibroblast growth factor receptor like 1	protein_coding	NP_001004356.1;NP_001004358.1;	-5.5
H2AC21	H2A clustered histone 21	protein_coding	NP_778235.1	-5.5
IDH2	isocitrate dehydrogenase (NADP(+)) 2	protein_coding	NP_001276839.1;NP_001277043.1;	-5.5
NDST1	N-deacetylase and N-sulfotransferase 1	protein_coding	NP_001287992.1;NP_001534.1	-5.5
LOC105371441	uncharacterized LOC105371441, transcript variant	lncRNA	.	-5.5
LOC284454	uncharacterized LOC284454	lncRNA	.	-5.5
MRPS26	mitochondrial ribosomal protein S26	protein_coding	NP_110438.1	-5.5
TIMM50	translocase of inner mitochondrial membrane 50	protein_coding	NP_001001563.2;NP_001316488.1;	-5.5
DNM1	dynamin 1	protein_coding	NP_001005336.1;NP_001275666.1;	-5.5
GSN	gelsolin	protein_coding	NP_000168.1;NP_001121134.1;NP_	-5.5
SGSM3	small G protein signaling modulator 3	protein_coding	NP_001288778.1;NP_001336968.1;	-5.5
ANO8	anoctamin 8	protein_coding	NP_066010.1;XP_016882537.1	-5.5
TMEM94	transmembrane protein 94	protein_coding	NP_001308077.1;NP_001308078.1;	-5.5
TNFRSF1B	TNF receptor superfamily member 1B	protein_coding	NP_001057.1;XP_011540362.1;XP_	-5.5
SLC16A14	solute carrier family 16 member 14	protein_coding	NP_689740.2;XP_005246410.1;XP_	-5.5
MYT1L	myelin transcription factor 1 like	protein_coding	NP_001289981.1;NP_001316773.1;	-5.5
RASA4DP	RAS p21 protein activator 4CD, pseudogene	ranscribed_pseudogen	.	-5.5
DNAAF5	dynein axonemal assembly factor 5	protein_coding	NP_060272.3;XP_024302581.1;XP_	-5.5
BRICD5	BRICHOS domain containing 5	protein_coding	NP_872369.2	-5.5
TCTA	T cell leukemia translocation altered	protein_coding	NP_071503.1	-5.5
MED29	mediator complex subunit 29	protein_coding	NP_001304699.2;NP_060062.2	-5.5
AP2S1	adaptor related protein complex 2 subunit sigma 1	protein_coding	NP_001288005.1;NP_001288007.1;	-5.5
VPS9D1	VPS9 domain containing 1	protein_coding	NP_004904.2;XP_005256386.1;XP_	-5.5
PROSER3	proline and serine rich 3	protein_coding	NP_001034976.2;NP_001354785.1;	-5.5
QTRT1	queuine tRNA-ribosyltransferase catalytic subunit	protein_coding	NP_112486.1	-5.5
ARAF	A-Raf proto-oncogene, serine/threonine kinase	protein_coding	NP_001243125.1;NP_001243126.1;	-5.5
CARHSP1	calcium regulated heat stable protein 1	protein_coding	NP_001035941.1;NP_001265189.1;	-5.5
GAK	cyclin G associated kinase	protein_coding	NP_001305063.1;NP_005246.2;XP_	-5.6
CYBC1	cytochrome b-245 chaperone 1	protein_coding	NP_001028218.1;NP_001093877.1;	-5.6
CHID1	chitinase domain containing 1	protein_coding	NP_001136146.1;NP_001136147.1;	-5.6
EIF3G	eukaryotic translation initiation factor 3 subunit G	protein_coding	NP_003746.2	-5.6
ARAP1	ArfGAP with RhoGAP domain, ankyrin repeat and	protein_coding	NP_001035207.1;NP_001128662.1;	-5.6
FRAT1	FRAT regulator of WNT signaling pathway 1	protein_coding	NP_005470.2	-5.6
CENPX	centromere protein X	protein_coding	NP_001257935.1;NP_001257936.1;	-5.6
XRCC1	X-ray repair cross complementing 1	protein_coding	NP_006288.2	-5.6
CNOT3	CCR4-NOT transcription complex subunit 3	protein_coding	NP_055331.1;XP_005278336.1;XP_	-5.6
FXR2	FMR1 autosomal homolog 2	protein_coding	NP_004851.2	-5.6
SLC35A4	solute carrier family 35 member A4	protein_coding	NP_542401.1	-5.6
ST6GALNAC6	ST6 N-acetylgalactosaminide alpha-2,6-sialyltrans	protein_coding	NP_001273928.1;NP_001273929.1;	-5.6
LIN7B	lin-7 homolog B, crumbs cell polarity complex con	protein_coding	NP_001295348.1;NP_071448.1;XP_	-5.6
KCNB1	potassium voltage-gated channel subfamily B men	protein_coding	NP_004966.1;XP_006723847.1;XP_	-5.6
RPL13	ribosomal protein L13	protein_coding	NP_000968.2;NP_001230060.1;NP_	-5.6
HDGF	heparin binding growth factor	protein_coding	NP_001119522.1;NP_001119523.1;	-5.6
HOXC8	homeobox C8	protein_coding	NP_073149.1	-5.6
TMEM189	transmembrane protein 189	protein_coding	NP_001155977.2;NP_954580.2	-5.6
MYG1	MYG1 exonuclease	protein_coding	NP_067653.4	-5.6

CAMK1	calcium/calmodulin dependent protein kinase I	protein_coding	NP_003647.1;XP_005265573.1;XP_	-5.6
RAB42	RAB42, member RAS oncogene family	protein_coding	NP_001180461.1;NP_689517.1;XP_	-5.6
TCOF1	treacle ribosome biogenesis factor 1	protein_coding	NP_000347.2;NP_001008657.1;NP_	-5.6
TRIM65	tripartite motif containing 65	protein_coding	NP_001243053.1;NP_775818.2;XP_	-5.6
EDC4	enhancer of mRNA decapping 4	protein_coding	NP_055144.3	-5.6
GATD3A	glutamine amidotransferase like class 1 domain co	protein_coding	NP_001307312.1;NP_001307313.1;	-5.6
MAD1L1	mitotic arrest deficient 1 like 1	protein_coding	NP_001013858.1;NP_001013859.1;	-5.6
THOC6	THO complex 6	protein_coding	NP_001135822.1;NP_001334632.1;	-5.6
SLC45A3	solute carrier family 45 member 3	protein_coding	NP_149093.1	-5.6
ROBO3	roundabout guidance receptor 3	protein_coding	NP_001357285.1;NP_001357286.1;	-5.6
ULK3	unc-51 like kinase 3	protein_coding	NP_001092906.3;NP_001271293.2;	-5.6
USP11	ubiquitin specific peptidase 11	protein_coding	NP_001358001.1;XP_005272731.1;	-5.7
PLEKHG2	pleckstrin homology and RhoGEF domain contain	protein_coding	NP_001338622.1;NP_001338623.1;	-5.7
DAZAP1	DAZ associated protein 1	protein_coding	NP_001338962.1;NP_001338963.1;	-5.7
POLRMTP1	RNA polymerase mitochondrial pseudogene 1	pseudogene	.	-5.7
FBXO46	F-box protein 46	protein_coding	NP_001073938.1;NP_001316561.1;	-5.7
AXL	AXL receptor tyrosine kinase	protein_coding	NP_001265528.1;NP_001690.2;NP_	-5.7
DRAP1	DR1 associated protein 1	protein_coding	NP_006433.2	-5.7
LINC00896	long intergenic non-protein coding RNA 896	lncRNA	.	-5.7
DEXI	Dexi homolog	protein_coding	NP_054734.2	-5.7
MYRF	myelin regulatory factor	protein_coding	NP_001120864.1;NP_037411.1;XP_	-5.7
PFAS	phosphoribosylformylglycinamidine synthase	protein_coding	NP_036525.1;XP_006721609.1;XP_	-5.7
AMDHD2	amidohydrolase domain containing 2	protein_coding	NP_001139287.1;NP_001317378.1;	-5.7
LDB1	LIM domain binding 1	protein_coding	NP_001106878.1;NP_001308541.1;	-5.7
FHDC1	FH2 domain containing 1	protein_coding	NP_001358045.1;NP_203751.2;XP_	-5.7
NABP2	nucleic acid binding protein 2	protein_coding	NP_076973.1;XP_005269204.1;XP_	-5.7
MUS81	MUS81 structure-specific endonuclease subunit	protein_coding	NP_001337212.1;NP_079404.3;XP_	-5.7
CAPNS1	calpain small subunit 1	protein_coding	NP_001003962.1;NP_001289561.1;	-5.7
SLC22A18	solute carrier family 22 member 18	protein_coding	NP_001302430.1;NP_001302431.1;	-5.7
PSRC1	proline and serine rich coiled-coil 1	protein_coding	NP_001005290.1;NP_001027462.1;	-5.7
ARID5A	AT-rich interaction domain 5A	protein_coding	NP_001306014.1;NP_001306016.1;	-5.7
LRP10	LDL receptor related protein 10	protein_coding	NP_001316155.1;NP_054764.2;XP_	-5.7
CCDC97	coiled-coil domain containing 97	protein_coding	NP_001333029.1;NP_443080.1;XP_	-5.7
NAGLU	N-acetyl-alpha-glucosaminidase	protein_coding	NP_000254.2;XP_006721983.1;XP_	-5.7
TBC1D9B	TBC1 domain family member 9B	protein_coding	NP_055858.2;NP_942568.2	-5.7
H2AC20	H2A clustered histone 20	protein_coding	NP_003508.1	-5.7
TEF	TEF transcription factor, PAR bZIP family membe	protein_coding	NP_001138870.1;NP_003207.1	-5.7
HIP1R	huntingtin interacting protein 1 related	protein_coding	NP_001290026.1;NP_001290028.1;	-5.7
KCTD15	potassium channel tetramerization domain contain	protein_coding	NP_001123466.1;NP_001123467.1;	-5.7
ASB13	ankyrin repeat and SOCS box containing 13	protein_coding	NP_078977.2	-5.7
MYORG	myogenesis regulating glycosidase (putative)	protein_coding	NP_065753.2;XP_011516268.1;XP_	-5.7
ACTG1	actin gamma 1	protein_coding	NP_001186883.1;NP_001605.1	-5.7
SLC25A23	solute carrier family 25 member 23	protein_coding	NP_077008.2;XP_011526576.1;XP_	-5.7
MAGEA2	MAGE family member A2	protein_coding	NP_001269430.1;NP_001269431.1;	-5.7
CDKN2A	cyclin dependent kinase inhibitor 2A	protein_coding	NP_000068.1;NP_001182061.1;NP_	-5.7
TBCB	tubulin folding cofactor B	protein_coding	NP_001272.2;NP_001287900.1	-5.8
MAGEA2B	MAGE family member A2B	protein_coding	NP_001308329.1;NP_001308330.1;	-5.8
TMEM9	transmembrane protein 9	protein_coding	NP_001275493.1;NP_001275494.1;	-5.8
MCRS1	microspherule protein 1	protein_coding	NP_001012300.1;NP_001265270.1;	-5.8
PARP10	poly(ADP-ribose) polymerase family member 10	protein_coding	NP_001304824.1;NP_116178.2;XP_	-5.8
EIF5AP4	eukaryotic translation initiation factor 5A pseudog	pseudogene	.	-5.8
ZFPM1	zinc finger protein, FOG family member 1	protein_coding	NP_722520.2;XP_011521214.1;XP_	-5.8
TRIR	telomerase RNA component interacting RNase	protein_coding	NP_001316667.1;NP_001316668.1;	-5.8
RNPS1P1	RNA binding protein with serine rich domain 1 ps	pseudogene	.	-5.8
NT5DC2	5'-nucleotidase domain containing 2	protein_coding	NP_001127703.1;NP_075059.1;XP_	-5.8
QPCTL	glutaminyl-peptide cyclotransferase like	protein_coding	NP_001156849.1;NP_060129.2;XP_	-5.8
BBC3	BCL2 binding component 3	protein_coding	NP_001120712.1;NP_001120713.1;	-5.8
DHX30	DExH-box helicase 30	protein_coding	NP_001317919.1;NP_055781.2;NP_	-5.8
MAD2L2	mitotic arrest deficient 2 like 2	protein_coding	NP_001120797.1;NP_006332.3;XP_	-5.8
POLM	DNA polymerase mu	protein_coding	NP_001271259.1;NP_001271260.1;	-5.8
ZNF74	zinc finger protein 74	protein_coding	NP_001243452.1;NP_001243453.1;	-5.8
ELOF1	elongation factor 1 homolog	protein_coding	NP_001350602.1;NP_001350603.1;	-5.8
BRD3	bromodomain containing 3	protein_coding	NP_031397.1;XP_006717354.1;XP_	-5.8
AP5Z1	adaptor related protein complex 5 subunit zeta 1	protein_coding	NP_001351787.1;NP_055670.1	-5.8
SOX13	SRY-box transcription factor 13	protein_coding	NP_005677.2;XP_005245680.1	-5.8
APH1A	aph-1 homolog A, gamma-secretase subunit	protein_coding	NP_001071096.1;NP_001230700.1;	-5.8
MTHFR	methylenetetrahydrofolate reductase	protein_coding	NP_001317287.1;NP_005948.3;XP_	-5.8
ZIC1	Zic family member 1	protein_coding	NP_003403.2	-5.8
SH3TC1	SH3 domain and tetratricopeptide repeats 1	protein_coding	NP_001305409.2;NP_061859.4;XP_	-5.8
USP19	ubiquitin specific peptidase 19	protein_coding	NP_001186089.1;NP_001186090.1;	-5.8
ARHGEF10L	Rho guanine nucleotide exchange factor 10 like	protein_coding	NP_001011722.2;NP_001306766.1;	-5.8

CHST3	carbohydrate sulfotransferase 3	protein_coding	NP_004264.2;XP_006718138.1;XP_	-5.8
PPT2	palmitoyl-protein thioesterase 2	protein_coding	NP_001191032.1;NP_005146.4;NP_	-5.8
BCL7C	BAF chromatin remodeling complex subunit BCL	protein_coding	NP_001273455.1;NP_004756.2;XP_	-5.8
C16orf74	chromosome 16 open reading frame 74	protein_coding	NP_996850.1	-5.8
NEURL4	neuralized E3 ubiquitin protein ligase 4	protein_coding	NP_001005408.1;NP_115818.2	-5.8
TSC2	TSC complex subunit 2	protein_coding	NP_000539.2;NP_001070651.1;NP_	-5.8
GLI4	GLI family zinc finger 4	protein_coding	NP_612474.1	-5.8
BRSK1	BR serine/threonine kinase 1	protein_coding	NP_115806.1;XP_005259384.1;XP_	-5.8
AMPD2	adenosine monophosphate deaminase 2	protein_coding	NP_001244290.1;NP_001295099.1;	-5.8
NUDT19	nudix hydrolase 19	protein_coding	NP_001099040.1;XP_016882295.1	-5.8
PPP4C	protein phosphatase 4 catalytic subunit	protein_coding	NP_001290432.1;NP_001290433.1;	-5.8
AGPAT1	1-acylglycerol-3-phosphate O-acyltransferase 1	protein_coding	NP_001358366.1;NP_001358367.1;	-5.8
MON1A	MON1 homolog A, secretory trafficking associate	protein_coding	NP_001135973.1;NP_115731.2;XP_	-5.9
ARFGAP1	ADP ribosylation factor GTPase activating protein	protein_coding	NP_001268411.1;NP_001268412.1;	-5.9
IQCE	IQ motif containing E	protein_coding	NP_001274428.1;NP_001274429.1;	-5.9
SRRT	serrate, RNA effector molecule	protein_coding	NP_001122324.1;NP_001122325.1;	-5.9
EMC6	ER membrane protein complex subunit 6	protein_coding	NP_001014764.1;NP_112588.1	-5.9
ARHGEF4	Rho guanine nucleotide exchange factor 4	protein_coding	NP_001354422.1;NP_001362829.1;	-5.9
SUFU	SUFU negative regulator of hedgehog signaling	protein_coding	NP_001171604.1;NP_057253.2;XP_	-5.9
LOC100996740	uncharacterized LOC100996740	lncRNA	.	-5.9
GADD45GIP1	GADD45G interacting protein 1	protein_coding	NP_443082.2	-5.9
C15orf39	chromosome 15 open reading frame 39	protein_coding	NP_056307.3	-5.9
WDTC1	WD and tetratricopeptide repeats 1	protein_coding	NP_001263181.1;NP_055838.2;XP_	-5.9
ITM2C	integral membrane protein 2C	protein_coding	NP_001012532.1;NP_001012534.1;	-5.9
SNORA5A	small nucleolar RNA, H/ACA box 5A	snoRNA	.	-5.9
EFNA4	ephrin A4	protein_coding	NP_005218.1;NP_872631.1;NP_87	-5.9
ARMC6	armadillo repeat containing 6	protein_coding	NP_001186125.1;NP_219483.1;XP_	-5.9
ANKRD52	ankyrin repeat domain 52	protein_coding	NP_775866.2;XP_011536499.1;XP_	-5.9
RPAP1	RNA polymerase II associated protein 1	protein_coding	NP_056355.2;XP_005254354.1	-5.9
HRAS	HRas proto-oncogene, GTPase	protein_coding	NP_001123914.1;NP_001304983.1;	-5.9
SNX17	sorting nexin 17	protein_coding	NP_001253988.1;NP_001253989.1;	-5.9
SMIM29	small integral membrane protein 29	protein_coding	NP_001008703.2;NP_001008704.2;	-5.9
DOT1L	DOT1 like histone lysine methyltransferase	protein_coding	NP_115871.1;XP_005259716.1;XP	-5.9
CCDC103	coiled-coil domain containing 103	protein_coding	NP_001245324.1;NP_001245325.1;	-5.9
CNPPD1	cyclin Pas1/PHO80 domain containing 1	protein_coding	NP_001308318.1;NP_001308319.2;	-5.9
PDK2	pyruvate dehydrogenase kinase 2	protein_coding	NP_001186827.1;NP_001186828.1;	-5.9
VPS4A	vacuolar protein sorting 4 homolog A	protein_coding	NP_037377.1	-5.9
LDOC1	LDOC1 regulator of NFKB signaling	protein_coding	NP_036449.1	-5.9
HDHD3	haloacid dehalogenase like hydrolase domain cont	protein_coding	NP_001291438.1;NP_001291439.1;	-5.9
HIC2	HIC ZBTB transcriptional repressor 2	protein_coding	NP_055909.2;XP_011528309.1;XP	-5.9
LZTR1	leucine zipper like transcription regulator 1	protein_coding	NP_006758.2	-6.0
ZBTB47	zinc finger and BTB domain containing 47	protein_coding	NP_660149.2	-6.0
ADAMTSL5	ADAMTS like 5	protein_coding	NP_001354126.1;NP_998769.2;XP_	-6.0
STARD3	StAR related lipid transfer domain containing 3	protein_coding	NP_001159409.1;NP_001159410.1;	-6.0
ACAP3	ArfGAP with coiled-coil, ankyrin repeat and PH d	protein_coding	NP_085152.2;XP_005244772.1;XP	-6.0
FAM50B	family with sequence similarity 50 member B	protein_coding	NP_036267.1;XP_016866218.1	-6.0
LRRC14	leucine rich repeat containing 14	protein_coding	NP_001258965.1;NP_055480.1;XP_	-6.0
KAZALD1	Kazal type serine peptidase inhibitor domain 1	protein_coding	NP_001306232.1;NP_112191.2;XP_	-6.0
DNAJC30	DnaJ heat shock protein family (Hsp40) member C	protein_coding	NP_115693.2	-6.0
CHST12	carbohydrate sulfotransferase 12	protein_coding	NP_001230723.1;NP_001230724.1;	-6.0
C1orf56	chromosome 1 open reading frame 56	protein_coding	NP_060330.2	-6.0
IRF2BPL	interferon regulatory factor 2 binding protein like	protein_coding	NP_078772.1	-6.0
SEMA3F	semaphorin 3F	protein_coding	NP_001305727.1;NP_001305729.1;	-6.0
NSUN5	NOP2/Sun RNA methyltransferase 5	protein_coding	NP_001161819.1;NP_001161820.1;	-6.0
DHRS13	dehydrogenase/reductase 13	protein_coding	NP_653284.2	-6.0
RPS2	ribosomal protein S2	protein_coding	NP_002943.2	-6.0
ZGPAT	zinc finger CCCH-type and G-patch domain conta	protein_coding	NP_001076582.1;NP_001182582.1;	-6.0
TMEM175	transmembrane protein 175	protein_coding	NP_001284352.1;NP_001284353.1;	-6.0
LOC107985388	pre-mRNA-splicing factor cwc22-like	protein_coding	XP_016883675.1	-6.0
ARHGAP1	Rho GTPase activating protein 1	protein_coding	NP_004299.1;XP_011518397.1;XP_	-6.0
NAA60	N-alpha-acetyltransferase 60, NatF catalytic subun	protein_coding	NP_001077069.1;NP_001077070.1;	-6.0
TBX2	T-box transcription factor 2	protein_coding	NP_005985.3	-6.0
MAN1B1-DT	MAN1B1 divergent transcript	lncRNA	.	-6.0
UBTF	upstream binding transcription factor	protein_coding	NP_001070151.1;NP_001070152.1;	-6.0
SLC35B2	solute carrier family 35 member B2	protein_coding	NP_001273438.1;NP_001273439.1;	-6.0
HMOX1	heme oxygenase 1	protein_coding	NP_002124.1	-6.0
PTPRS	protein tyrosine phosphatase receptor type S	protein_coding	NP_002841.3;NP_570923.2;NP_57(	-6.0
ZNF618	zinc finger protein 618	protein_coding	NP_001304969.1;NP_001304970.1;	-6.0
CSPG4P11	chondroitin sulfate proteoglycan 4 pseudogene 11	pseudogene	.	-6.0
C8orf58	chromosome 8 open reading frame 58	protein_coding	NP_001013864.1;NP_001185756.1;	-6.1
P4HTM	prolyl 4-hydroxylase, transmembrane	protein_coding	NP_808807.2;NP_808808.1	-6.1

ARRB2	arrestin beta 2	protein_coding	NP_001244257.1;NP_001244258.1;	-6.1
KIAA1614	KIAA1614	protein_coding	NP_066001.1	-6.1
RNF167	ring finger protein 167	protein_coding	NP_001307285.1;NP_001307286.1;	-6.1
PPP2R5B	protein phosphatase 2 regulatory subunit B'beta	protein_coding	NP_006235.1;XP_011543434.1;XP_	-6.1
GFUS	GDP-L-fucose synthase	protein_coding	NP_001304712.1;NP_003304.1;XP_	-6.1
FOXD1	forkhead box D1	protein_coding	NP_004463.1	-6.1
PEPD	peptidase D	protein_coding	NP_000276.2;NP_001159528.1;NP_	-6.1
RCE1	Ras converting CAAX endopeptidase 1	protein_coding	NP_001027450.1;NP_005124.1	-6.1
POLR1G	RNA polymerase I subunit G	protein_coding	NP_001284519.1;NP_036231.1	-6.1
OSGIN1	oxidative stress induced growth inhibitor 1	protein_coding	NP_892026.1	-6.1
LOC107987300	uncharacterized LOC107987300	lncRNA	.	-6.1
NRM	nurim	protein_coding	NP_001257636.1;NP_001257637.1;	-6.1
LOC100129503	uncharacterized LOC100129503	lncRNA	.	-6.1
FIZ1	FLT3 interacting zinc finger 1	protein_coding	NP_116225.2;XP_005259409.1;XP_	-6.1
NDUFV1	NADH:ubiquinone oxidoreductase core subunit V	protein_coding	NP_001159574.1;NP_009034.2	-6.1
MAP11	microtubule associated protein 11	protein_coding	NP_001290399.1;NP_060745.3	-6.1
AKT2	AKT serine/threonine kinase 2	protein_coding	NP_001229956.1;NP_001229957.1;	-6.1
ASF1B	anti-silencing function 1B histone chaperone	protein_coding	NP_060624.1	-6.1
TAB1	TGF-beta activated kinase 1 (MAP3K7) binding p	protein_coding	NP_006107.1;NP_705717.1	-6.1
LOC112268179	uncharacterized LOC112268179	lncRNA	.	-6.1
B3GALT6	beta-1,3-galactosyltransferase 6	protein_coding	NP_542172.2	-6.1
NIBAN3	niban apoptosis regulator 3	protein_coding	NP_001091994.1;NP_001308755.2;	-6.1
WBP2	WW domain binding protein 2	protein_coding	NP_001317428.1;NP_001335099.1;	-6.1
RNF5	ring finger protein 5	protein_coding	NP_008844.1	-6.1
AAMP	angio associated migratory cell protein	protein_coding	NP_001078.2;NP_001289474.1;XP_	-6.1
ITPKC	inositol-trisphosphate 3-kinase C	protein_coding	NP_079470.1;XP_016882813.1	-6.1
APOBEC3C	apolipoprotein B mRNA editing enzyme catalytic	protein_coding	NP_055323.2;XP_024307986.1	-6.1
BORCS8-MEF2B	BORCS8-MEF2B readthrough	protein_coding	NP_005910.1	-6.1
TRADD	TNFRSF1A associated via death domain	protein_coding	NP_001310481.1;NP_003780.1;XP_	-6.1
RHOG	ras homolog family member G	protein_coding	NP_001656.2;XP_005252973.1;XP_	-6.1
MACROD1	mono-ADP ribosylhydrolase 1	protein_coding	NP_054786.2;XP_005273996.1;XP_	-6.1
GDPD5	glycerophosphodiester phosphodiesterase domain	protein_coding	NP_001338096.1;NP_001338097.1;	-6.1
TUSC1	tumor suppressor candidate 1	protein_coding	NP_001004125.1	-6.2
H4C1	H4 clustered histone 1	protein_coding	NP_003529.1	-6.2
ANKRD11P2	ANKRD11 pseudogene 2	pseudogene	.	-6.2
ASB6	ankyrin repeat and SOCS box containing 6	protein_coding	NP_001189332.1;NP_060343.1;NP_	-6.2
DAPK3	death associated protein kinase 3	protein_coding	NP_001339.1;NP_001362587.1	-6.2
SUV39H1	suppressor of variegation 3-9 homolog 1	protein_coding	NP_001269095.1;NP_003164.1	-6.2
LINC01836	long intergenic non-protein coding RNA 1836	lncRNA	.	-6.2
MTX1	metaxin 1	protein_coding	NP_002446.3;NP_942584.2	-6.2
LOC105372824	uncharacterized LOC105372824	protein_coding	XP_011528113.1	-6.2
CASTOR1	cytosolic arginine sensor for mTORC1 subunit 1	protein_coding	NP_001032755.1	-6.2
ZFPL1	zinc finger protein like 1	protein_coding	NP_006773.2	-6.2
ZBTB3	zinc finger and BTB domain containing 3	protein_coding	NP_001350037.1;NP_001350038.1;	-6.2
ARL8A	ADP ribosylation factor like GTPase 8A	protein_coding	NP_001243058.1;NP_620150.1	-6.2
PROB1	proline rich basic protein 1	protein_coding	NP_001155018.1	-6.2
FAM131A	family with sequence similarity 131 member A	protein_coding	NP_001164564.1;NP_001353062.1;	-6.2
PITPNM3	PITPNM family member 3	protein_coding	NP_001159438.1;NP_112497.2;XP_	-6.2
BTG2	BTG anti-proliferation factor 2	protein_coding	NP_006754.1	-6.2
GSTP1	glutathione S-transferase pi 1	protein_coding	NP_000843.1	-6.2
PPAN-P2RY11	PPAN-P2RY11 readthrough	protein_coding	NP_001035754.1;NP_001185619.1	-6.2
AP1B1	adaptor related protein complex 1 subunit beta 1	protein_coding	NP_001118.3;NP_001159491.1;NP_	-6.2
E4F1	E4F transcription factor 1	protein_coding	NP_001275705.1;NP_001275707.1;	-6.2
ZFP36	ZFP36 ring finger protein	protein_coding	NP_003398.3	-6.3
CENPBD1P1	CENPB DNA-binding domains containing 1 pseu	transcribed_pseudogene	.	-6.3
PCGF2	polycomb group ring finger 2	protein_coding	NP_001356543.1;NP_001356544.1;	-6.3
FJX1	four-jointed box kinase 1	protein_coding	NP_055159.2	-6.3
CA11	carbonic anhydrase 11	protein_coding	NP_001208.2	-6.3
SLC35C1	solute carrier family 35 member C1	protein_coding	NP_001138737.1;NP_001138738.1;	-6.3
EML3	EMAP like 3	protein_coding	NP_001287722.1;NP_001287723.1;	-6.3
SLC66A2	solute carrier family 66 member 2	protein_coding	NP_001139815.1;NP_001139817.1;	-6.3
THOP1	thimet oligopeptidase 1	protein_coding	NP_003240.1;XP_011526530.1	-6.3
SCRN2	secernin 2	protein_coding	NP_001138495.1;NP_612364.2;XP_	-6.3
PLEKHO2	pleckstrin homology domain containing O2	protein_coding	NP_001181988.1;NP_079477.2	-6.3
LETM1	leucine zipper and EF-hand containing transmemb	protein_coding	NP_036450.1;XP_006713947.1	-6.3
LINC02809	long intergenic non-protein coding RNA 2809	lncRNA	.	-6.3
C1orf35	chromosome 1 open reading frame 35	protein_coding	NP_077295.1	-6.3
PNKD	PNKD metallo-beta-lactamase domain containing	protein_coding	NP_001070867.1;NP_056303.3;NP_	-6.3
NCDN	neurochondrin	protein_coding	NP_001014839.1;NP_001014841.1;	-6.3
ZNF629	zinc finger protein 629	protein_coding	NP_001073886.1;NP_001332899.1	-6.3
C11orf68	chromosome 11 open reading frame 68	protein_coding	NP_001129107.1;NP_113638.2	-6.3

RANGAP1	Ran GTPase activating protein 1	protein_coding	NP_001265580.1;NP_001304859.1;	-6.3
MVD	mevalonate diphosphate decarboxylase	protein_coding	NP_002452.1;XP_011521388.1;XP_	-6.3
MNT	MAX network transcriptional repressor	protein_coding	NP_064706.1;XP_011522170.1;XP_	-6.3
HDAC10	histone deacetylase 10	protein_coding	NP_001152758.1;NP_114408.3	-6.3
GTF3C5	general transcription factor IIIC subunit 5	protein_coding	NP_001116295.1;NP_001273638.1;	-6.3
IQSEC1	IQ motif and Sec7 domain ArfGEF 1	protein_coding	NP_001127854.1;NP_001317548.1;	-6.3
ALYREF	Aly/REF export factor	protein_coding	NP_005773.3	-6.3
ZER1	zyg-11 related cell cycle regulator	protein_coding	NP_001362883.1;NP_001362884.1;	-6.3
MARK2	microtubule affinity regulating kinase 2	protein_coding	NP_001034558.2;NP_001156768.1;	-6.3
TRI-AAT2-1	tRNA-Ile	tRNA	.	-6.3
ARHGAP39	Rho GTPase activating protein 39	protein_coding	NP_001295136.1;NP_001295137.1;	-6.3
TWIST1	twist family bHLH transcription factor 1	protein_coding	NP_000465.1	-6.3
TUT1	terminal uridylyl transferase 1, U6 snRNA-specifi	protein_coding	NP_001354835.1;NP_073741.3	-6.3
PRRT2	proline rich transmembrane protein 2	protein_coding	NP_001243371.1;NP_001243372.1;	-6.3
CIZ1	CDKN1A interacting zinc finger protein 1	protein_coding	NP_001124487.1;NP_001124488.1;	-6.3
ADAMTS4	ADAM metallopeptidase with thrombospondin typ	protein_coding	NP_001307265.1;NP_005090.3	-6.3
PKD1P5	polycystin 1, transient receptor potential channel i	pseudogene	.	-6.3
PMM1	phosphomannomutase 1	protein_coding	NP_002667.2;XP_005261695.1;XP_	-6.3
MRPL23	mitochondrial ribosomal protein L23	protein_coding	NP_066957.3;XP_006718334.1;XP_	-6.4
P2RX6	purinergic receptor P2X 6	protein_coding	NP_001153026.1;NP_001336803.1;	-6.4
POLR3H	RNA polymerase III subunit H	protein_coding	NP_001018060.1;NP_001018062.1;	-6.4
IFRD2	interferon related developmental regulator 2	protein_coding	NP_006755.4	-6.4
MARVELD1	MARVEL domain containing 1	protein_coding	NP_113672.1	-6.4
COMT	catechol-O-methyltransferase	protein_coding	NP_000745.1;NP_001128633.1;NP_	-6.4
SCARNA13	small Cajal body-specific RNA 13	guide_RNA	.	-6.4
SLC16A13	solute carrier family 16 member 13	protein_coding	NP_963860.1	-6.4
DDA1	DET1 and DDB1 associated 1	protein_coding	NP_076955.1;XP_024307469.1	-6.4
UNC45A	unc-45 myosin chaperone A	protein_coding	NP_001034764.1;NP_001310548.1;	-6.4
EPN2	epsin 2	protein_coding	NP_001096134.1;NP_055779.2;NP_	-6.4
CDK5R1	cyclin dependent kinase 5 regulatory subunit 1	protein_coding	NP_003876.1;XP_011523740.1;XP_	-6.4
RILP	Rab interacting lysosomal protein	protein_coding	NP_113618.2;XP_005256868.1;XP_	-6.4
BCKDHA	branched chain keto acid dehydrogenase E1 subun	protein_coding	NP_000700.1;NP_001158255.1	-6.4
EIF5A	eukaryotic translation initiation factor 5A	protein_coding	NP_001137232.1;NP_001137233.1;	-6.4
H4C4	H4 clustered histone 4	protein_coding	NP_003530.1	-6.4
PUF60	poly(U) binding splicing factor 60	protein_coding	NP_001129505.1;NP_001258025.1;	-6.4
MAP3K10	mitogen-activated protein kinase kinase kinase 10	protein_coding	NP_002437.2;XP_006723286.1;XP_	-6.4
ZBTB22	zinc finger and BTB domain containing 22	protein_coding	NP_001138810.1;NP_005444.4	-6.4
JADE2	jade family PHD finger 2	protein_coding	NP_001276913.1;NP_001276914.1;	-6.4
LGALS3BP	galectin 3 binding protein	protein_coding	NP_005558.1	-6.4
CDC42EP2	CDC42 effector protein 2	protein_coding	NP_006770.1	-6.4
FGD1	FYVE, RhoGEF and PH domain containing 1	protein_coding	NP_004454.2	-6.4
ARMC7	armadillo repeat containing 7	protein_coding	NP_001291200.1;NP_001291201.1;	-6.4
PTPRF	protein tyrosine phosphatase receptor type F	protein_coding	NP_001316066.1;NP_001316067.1;	-6.4
SLC12A4	solute carrier family 12 member 4	protein_coding	NP_001139433.1;NP_001139434.1;	-6.4
WNT9A	Wnt family member 9A	protein_coding	NP_003386.1;XP_011542573.1	-6.4
TKT	transketolase	protein_coding	NP_001055.1;NP_001128527.1;NP_	-6.4
SLC1A5	solute carrier family 1 member 5	protein_coding	NP_001138616.1;NP_001138617.1;	-6.4
ATP13A1	ATPase 13A1	protein_coding	NP_065143.2	-6.4
HYAL3	hyaluronidase 3	protein_coding	NP_001186958.1;NP_001186959.1;	-6.4
GATAD2A	GATA zinc finger domain containing 2A	protein_coding	NP_001287875.1;NP_001346560.1;	-6.4
TMEM109	transmembrane protein 109	protein_coding	NP_076997.1	-6.4
RFXANK	regulatory factor X associated ankyrin containing	protein_coding	NP_001265656.1;NP_001265657.1;	-6.4
IRF3	interferon regulatory factor 3	protein_coding	NP_001184051.1;NP_001184052.1;	-6.5
BAK1P1	BCL2 antagonist/killer 1 pseudogene 1	pseudogene	.	-6.5
KIF18B	kinesin family member 18B	protein_coding	NP_001251503.1;NP_001252506.1;	-6.5
CBX7	chromobox 7	protein_coding	NP_001333672.1;NP_001333673.1;	-6.5
RUSC1	RUN and SH3 domain containing 1	protein_coding	NP_001098673.1;NP_001098674.1;	-6.5
TUFM	Tu translation elongation factor, mitochondrial	protein_coding	NP_001352289.1;NP_003312.3	-6.5
ORAI1	ORAI calcium release-activated calcium modulato	protein_coding	NP_116179.2	-6.5
SEMA3B	semaphorin 3B	protein_coding	NP_001005914.1;NP_001276989.1;	-6.5
POLD4	DNA polymerase delta 4, accessory subunit	protein_coding	NP_001243799.1;NP_066996.3	-6.5
VPS28	VPS28 subunit of ESCRT-I	protein_coding	NP_057292.1;NP_898880.1;XP_00	-6.5
CEBPB	CCAAT enhancer binding protein beta	protein_coding	NP_001272807.1;NP_001272808.1;	-6.5
MIGA2	mitoguardin 2	protein_coding	NP_001316919.1;NP_116198.3;XP_	-6.5
GET4	guided entry of tail-anchored proteins factor 4	protein_coding	NP_057033.2	-6.5
RHBDD2	rhomboid domain containing 2	protein_coding	NP_001035546.1;NP_001035547.1;	-6.5
GPX1	glutathione peroxidase 1	protein_coding	NP_000572.2;NP_001316384.1;NP_	-6.5
TRAF4	TNF receptor associated factor 4	protein_coding	NP_004286.2;XP_011523806.1;XP_	-6.5
RAB15	RAB15, member RAS oncogene family	protein_coding	NP_001295083.1;NP_001317111.1;	-6.5
RNFT2	ring finger protein, transmembrane 2	protein_coding	NP_001103373.1;NP_001369195.1;	-6.5
EFHD2	EF-hand domain family member D2	protein_coding	NP_077305.2;XP_005246057.1	-6.5

PBXIP1	PBX homeobox interacting protein 1	protein_coding	NP_001304663.1;NP_001304664.1;	-6.5
RPS6KA1	ribosomal protein S6 kinase A1	protein_coding	NP_001006666.1;NP_001317370.1;	-6.5
TRAF2	TNF receptor associated factor 2	protein_coding	NP_066961.2;XP_011517276.1;XP_	-6.5
MYBBP1A	MYB binding protein 1a	protein_coding	NP_001099008.1;NP_055335.2;XP_	-6.5
LOC102724191	.	pseudogene	.	-6.5
ATP8B3	ATPase phospholipid transporting 8B3	protein_coding	NP_001171473.1;NP_620168.1;XP_	-6.5
EXOSC5	exosome component 5	protein_coding	NP_064543.3	-6.5
GATD1	glutamine amidotransferase like class 1 domain co	protein_coding	NP_001305747.1;NP_001305749.1;	-6.6
CISD3	CDGSH iron sulfur domain 3	protein_coding	NP_001129970.1	-6.6
IGFL2	IGF like family member 2	protein_coding	NP_001002915.2;NP_001128585.1;	-6.6
PCDHGC3	protocadherin gamma subfamily C, 3	protein_coding	NP_002579.2;NP_115778.1;NP_11	-6.6
STK25	serine/threonine kinase 25	protein_coding	NP_001258906.1;NP_001258907.1;	-6.6
TOB2	transducer of ERBB2, 2	protein_coding	NP_057356.1;XP_005261372.1;XP_	-6.6
MCOLN1	mucolipin 1	protein_coding	NP_065394.1	-6.6
RN7SL1	RNA component of signal recognition particle 7SI	scRNA	.	-6.6
FUT1	fucosyltransferase 1 (H blood group)	protein_coding	NP_000139.1;NP_001316806.1	-6.6
LRRC41	leucine rich repeat containing 41	protein_coding	NP_006360.3	-6.6
GTPBP3	GTP binding protein 3, mitochondrial	protein_coding	NP_001122327.1;NP_001182351.1;	-6.6
FRMD8	FERM domain containing 8	protein_coding	NP_001287761.1;NP_001287762.1;	-6.6
CPSF1	cleavage and polyadenylation specific factor 1	protein_coding	NP_037423.2;XP_006716611.1;XP_	-6.6
SNX21	sorting nexin family member 21	protein_coding	NP_001036097.1;NP_001036098.1;	-6.6
MGAT1	alpha-1,3-mannosyl-glycoprotein 2-beta-N-acetylg	protein_coding	NP_001108089.1;NP_001108090.1;	-6.6
MPDU1	mannose-P-dolichol utilization defect 1	protein_coding	NP_001317002.1;NP_004861.2;XP_	-6.6
ZNF764	zinc finger protein 764	protein_coding	NP_001166150.1;NP_219363.2	-6.6
AKNA	AT-hook transcription factor	protein_coding	NP_001304879.1;NP_001304881.1;	-6.6
H3C3	H3 clustered histone 3	protein_coding	NP_003522.1	-6.6
TP53	tumor protein p53	protein_coding	NP_000537.3;NP_001119584.1;NP_	-6.6
LAMA5	laminin subunit alpha 5	protein_coding	NP_005551.3;XP_006723859.1;XP_	-6.6
MYO7B	myosin VIIB	protein_coding	NP_001073996.1;XP_006712602.1;	-6.7
CCDC88C	coiled-coil domain containing 88C	protein_coding	NP_001073883.2;XP_005267748.1;	-6.7
PYGB	glycogen phosphorylase B	protein_coding	NP_002853.2	-6.7
DNAL4	dynein axonemal light chain 4	protein_coding	NP_005731.1	-6.7
COL4A2	collagen type IV alpha 2 chain	protein_coding	NP_001837.2	-6.7
METTL7B	methyltransferase like 7B	protein_coding	NP_689850.2	-6.7
G6PD	glucose-6-phosphate dehydrogenase	protein_coding	NP_000393.4;NP_001035810.1;NP_	-6.7
SDHAF1	succinate dehydrogenase complex assembly factor	protein_coding	NP_001036096.2	-6.7
XRCC3	X-ray repair cross complementing 3	protein_coding	NP_001093588.1;NP_001093589.1;	-6.7
PSMD3	proteasome 26S subunit, non-ATPase 3	protein_coding	NP_002800.2	-6.7
RIC8A	RIC8 guanine nucleotide exchange factor A	protein_coding	NP_001273063.1;NP_068751.4;XP_	-6.7
AGFG2	ArfGAP with FG repeats 2	protein_coding	NP_006067.3;XP_005250363.1;XP_	-6.7
C3orf18	chromosome 3 open reading frame 18	protein_coding	NP_001165211.1;NP_001165212.1;	-6.7
KIAA2013	KIAA2013	protein_coding	NP_612355.1	-6.7
PHLDB1	pleckstrin homology like domain family B membe	protein_coding	NP_001138230.1;NP_001138231.1;	-6.7
PXN	paxillin	protein_coding	NP_001074324.1;NP_001230685.1;	-6.7
ZNRD2	zinc ribbon domain containing 2	protein_coding	NP_001289953.1;NP_006387.1	-6.7
MRC2	mannose receptor C type 2	protein_coding	NP_006030.2;XP_011523845.1	-6.7
CYHR1	cysteine and histidine rich 1	protein_coding	NP_001123360.1;NP_001317547.1;	-6.7
ARRB1	arrestin beta 1	protein_coding	NP_004032.2;NP_064647.1;XP_01	-6.7
PLEKHG3	pleckstrin homology and RhoGEF domain contain	protein_coding	NP_001295076.1;XP_011534927.1;	-6.7
MAPK7	mitogen-activated protein kinase 7	protein_coding	NP_002740.2;NP_620601.1;NP_62(	-6.7
PPP1R3G	protein phosphatase 1 regulatory subunit 3G	protein_coding	NP_001138587.1	-6.7
ADAMTS2	ADAM metallopeptidase with thrombospondin tyƒ	protein_coding	NP_055059.2;NP_067610.1	-6.7
CSPG4P5	chondroitin sulfate proteoglycan 4 pseudogene 5	pseudogene	.	-6.7
RPUSD3	RNA pseudouridine synthase D3	protein_coding	NP_001136019.1;NP_001338665.1;	-6.7
JPH2	junctophilin 2	protein_coding	NP_065166.2;NP_787109.2;XP_00(	-6.7
EPB41L1	erythrocyte membrane protein band 4.1 like 1	protein_coding	NP_001245258.1;NP_001245259.1;	-6.7
PDLIM4	PDZ and LIM domain 4	protein_coding	NP_001124499.1;NP_003678.2;XP_	-6.8
PANX2	pannexin 2	protein_coding	NP_001153772.1;NP_443071.2	-6.8
CACNB3	calcium voltage-gated channel auxiliary subunit b	protein_coding	NP_000716.2;NP_001193844.1;NP_	-6.8
ZNF707	zinc finger protein 707	protein_coding	NP_001094068.1;NP_001094069.1;	-6.8
ENKD1	enkurin domain containing 1	protein_coding	NP_115516.1;XP_024306237.1;XP_	-6.8
MEX3B	mex-3 RNA binding family member B	protein_coding	NP_115622.2	-6.8
ZNF784	zinc finger protein 784	protein_coding	NP_976308.1	-6.8
LOC107987158	uncharacterized LOC107987158, transcript variant	protein_coding	XP_016874124.1;XP_016874125.1;	-6.8
PLXND1	plexin D1	protein_coding	NP_055918.3;XP_011510890.1;XP_	-6.8
DNAJC4	DnaJ heat shock protein family (Hsp40) member C	protein_coding	NP_001294909.1;NP_001294910.1;	-6.8
SLC9A1	solute carrier family 9 member A1	protein_coding	NP_003038.2;XP_011540323.1	-6.8
CSPG4P10	chondroitin sulfate proteoglycan 4 pseudogene 10	pseudogene	.	-6.8
ZDHHC18	zinc finger DHHC-type palmitoyltransferase 18	protein_coding	NP_115659.1	-6.8
SLC39A1	solute carrier family 39 member 1	protein_coding	NP_001258886.1;NP_001258887.1;	-6.8
CTIF	cap binding complex dependent translation initiati	protein_coding	NP_001135869.1;NP_055587.1;XP_	-6.8

PNKP	polynucleotide kinase 3'-phosphatase	protein_coding	NP_009185.2	-6.8
TNFSF9	TNF superfamily member 9	protein_coding	NP_003802.1	-6.8
BCL9	BCL9 transcription coactivator	protein_coding	NP_004317.2;XP_005273028.1;XP_	-6.8
DUS3L	dihydrouridine synthase 3 like	protein_coding	NP_001155091.1;NP_064560.2;XP_	-6.8
UCP2	uncoupling protein 2	protein_coding	NP_001368872.1;NP_001368873.1;	-6.8
SOX6	SRY-box transcription factor 6	protein_coding	NP_001139283.1;NP_001139291.2;	-6.8
LBX2-AS1	LBX2 antisense RNA 1	lncRNA	.	-6.8
LSM4	LSM4 homolog, U6 small nuclear RNA and mRN	protein_coding	NP_001239058.1;NP_036453.1	-6.8
RPL18AP3	ribosomal protein L18a pseudogene 3	pseudogene	.	-6.8
DOK4	docking protein 4	protein_coding	NP_001317485.1;NP_001356547.1;	-6.8
TECRP1	trans-2,3-enoyl-CoA reductase pseudogene 1	pseudogene	.	-6.8
TMEM8B	transmembrane protein 8B	protein_coding	NP_001036054.1;NP_001036055.2;	-6.9
MAP4K2	mitogen-activated protein kinase kinase kinase kin	protein_coding	NP_001294919.1;NP_004570.2;XP_	-6.9
MOGS	mannosyl-oligosaccharide glucosidase	protein_coding	NP_001139630.1;NP_006293.2	-6.9
RAB35	RAB35, member RAS oncogene family	protein_coding	NP_001161078.1;NP_006852.1;XP_	-6.9
AGAP2	ArfGAP with GTPase domain, ankyrin repeat and	protein_coding	NP_001116244.1;NP_055585.1;XP_	-6.9
TNS2	tensin 2	protein_coding	NP_056134.2;NP_736610.2;NP_93[illegible]	-6.9
CSRNP1	cysteine and serine rich nuclear protein 1	protein_coding	NP_001307488.1;NP_001307489.1;	-6.9
MPV17L2	MPV17 mitochondrial inner membrane protein lik	protein_coding	NP_116072.2	-6.9
TM7SF2	transmembrane 7 superfamily member 2	protein_coding	NP_001264162.1;NP_003264.2	-6.9
TRPM2	transient receptor potential cation channel subfami	protein_coding	NP_001307279.1;NP_001307280.1;	-6.9
LOC102723834	uncharacterized LOC102723834, transcript varian	lncRNA	.	-6.9
INO80E	INO80 complex subunit E	protein_coding	NP_001291491.1;NP_001291492.1;	-6.9
MRPS34	mitochondrial ribosomal protein S34	protein_coding	NP_001287829.1;NP_076425.1	-7.0
RCC1L	RCC1 like	protein_coding	NP_001268370.1;NP_001350376.1;	-7.0
FBXW9	F-box and WD repeat domain containing 9	protein_coding	NP_115677.2;XP_005260153.1	-7.0
MIB2	mindbomb E3 ubiquitin protein ligase 2	protein_coding	NP_001164157.2;NP_001164158.2;	-7.0
RUSF1	RUS family member 1	protein_coding	NP_073581.2;XP_016879059.1	-7.0
HOGA1	4-hydroxy-2-oxoglutarate aldolase 1	protein_coding	NP_001128142.1;NP_612422.2	-7.0
SLC25A6	solute carrier family 25 member 6	protein_coding	NP_001627.2	-7.0
GATD3B	glutamine amidotransferase like class 1 domain co	protein_coding	NP_001350687.1;NP_001350689.1;	-7.0
SLC35F6	solute carrier family 35 member F6	protein_coding	NP_060347.2	-7.0
ACTB	actin beta	protein_coding	NP_001092.1	-7.0
YDJC	YdjC chitooligosaccharide deacetylase homolog	protein_coding	NP_001017964.1;NP_001358279.1	-7.0
RIN3	Ras and Rab interactor 3	protein_coding	NP_001306916.1;NP_079108.3;XP	-7.0
RNF40	ring finger protein 40	protein_coding	NP_001193962.1;NP_001193963.1;	-7.0
PC	pyruvate carboxylase	protein_coding	NP_000911.2;NP_001035806.1;NP_	-7.0
MAP2K7	mitogen-activated protein kinase kinase 7	protein_coding	NP_001284484.1;NP_001284485.1;	-7.0
TNFRSF12A	TNF receptor superfamily member 12A	protein_coding	NP_057723.1	-7.0
PCDHB5	protocadherin beta 5	protein_coding	NP_056484.2	-7.0
MARCHF4	membrane associated ring-CH-type finger 4	protein_coding	NP_065865.1	-7.0
SLC35E4	solute carrier family 35 member E4	protein_coding	NP_001001479.1;NP_001305299.1;	-7.0
LOC112268269	uncharacterized LOC112268269	lncRNA	.	-7.0
ARHGEF18	Rho/Rac guanine nucleotide exchange factor 18	protein_coding	NP_001124427.2;NP_001354752.1;	-7.0
NELFA	negative elongation factor complex member A	protein_coding	NP_005654.4;XP_016864078.1	-7.0
PEX6	peroxisomal biogenesis factor 6	protein_coding	NP_000278.3;NP_001303242.1;XP	-7.0
UPF1	UPF1 RNA helicase and ATPase	protein_coding	NP_001284478.1;NP_002902.2;XP_	-7.0
PLEKHS1	pleckstrin homology domain containing S1	protein_coding	NP_001180363.1;NP_001180364.1;	-7.0
RNF166	ring finger protein 166	protein_coding	NP_001165286.1;NP_001165287.1;	-7.0
B3GAT3	beta-1,3-glucuronyltransferase 3	protein_coding	NP_001275650.1;NP_001275651.1;	-7.1
ANK1	ankyrin 1	protein_coding	NP_000028.3;NP_001135917.1;NP_	-7.1
TGFB2-OT1	TGFB2 overlapping transcript 1	lncRNA	.	-7.1
P3H3	prolyl 3-hydroxylase 3	protein_coding	NP_055077.2	-7.1
URM1	ubiquitin related modifier 1	protein_coding	NP_001129419.1;NP_001252511.1;	-7.1
H2AC15	H2A clustered histone 15	protein_coding	NP_003501.1	-7.1
TCF7	transcription factor 7	protein_coding	NP_001128323.2;NP_001333354.1;	-7.1
MAGEA6	MAGE family member A6	protein_coding	NP_005354.1;NP_787064.1	-7.1
POLD2	DNA polymerase delta 2, accessory subunit	protein_coding	NP_001120690.1;NP_001243808.1;	-7.1
LAGE3	L antigen family member 3	protein_coding	NP_006005.2	-7.1
CLN6	CLN6 transmembrane ER protein	protein_coding	NP_060352.1	-7.1
FOXO4	forkhead box O4	protein_coding	NP_001164402.1;NP_005929.2;XP_	-7.1
F8A2	coagulation factor VIII associated 2	protein_coding	NP_001007524.1	-7.1
APBA3	amyloid beta precursor protein binding family A n	protein_coding	NP_004877.1;XP_006723013.1;XP_	-7.1
PPP2R1A	protein phosphatase 2 scaffold subunit Aalpha	protein_coding	NP_001350585.1;NP_055040.2	-7.1
ITGA7	integrin subunit alpha 7	protein_coding	NP_001138468.1;NP_001138469.1;	-7.1
DAG1	dystroglycan 1	protein_coding	NP_001159400.2;NP_001171105.1;	-7.1
ZDHHC24	zinc finger DHHC-type containing 24	protein_coding	NP_001335500.1;NP_997223.1;XP_	-7.1
CMBL	carboxymethylenebutenolidase homolog	protein_coding	NP_620164.1	-7.1
H2BC14	H2B clustered histone 14	protein_coding	NP_003512.1	-7.1
ZNF687	zinc finger protein 687	protein_coding	NP_001291692.1;NP_001291693.1;	-7.1
SPEG	striated muscle enriched protein kinase	protein_coding	NP_001166947.1;NP_005867.3;XP_	-7.1

PCYT2	phosphate cytidylyltransferase 2, ethanolamine	protein_coding	NP_001171846.1;NP_001243362.1;	-7.1
DGAT1	diacylglycerol O-acyltransferase 1	protein_coding	NP_036211.2;XP_011515658.1	-7.1
KLF15	Kruppel like factor 15	protein_coding	NP_054798.1;XP_005247457.1;XP_	-7.1
ABHD14A	abhydrolase domain containing 14A	protein_coding	NP_056222.2	-7.1
SCARB1	scavenger receptor class B member 1	protein_coding	NP_001076428.1;NP_001354910.1;	-7.1
IMPA2	inositol monophosphatase 2	protein_coding	NP_055029.1;XP_011523961.1;XP_	-7.1
BRI3	brain protein I3	protein_coding	NP_001152963.1;NP_056194.1;XP_	-7.2
TIMM29	translocase of inner mitochondrial membrane 29	protein_coding	NP_612367.1	-7.2
NFATC4	nuclear factor of activated T cells 4	protein_coding	NP_001129494.1;NP_001185894.1;	-7.2
C11orf95	chromosome 11 open reading frame 95	protein_coding	NP_001138408.1;XP_024304430.1	-7.2
RPL8	ribosomal protein L8	protein_coding	NP_000964.1;NP_001304700.1;NP_	-7.2
ZMAT5	zinc finger matrin-type 5	protein_coding	NP_001003692.1;NP_001305058.1;	-7.2
NME4	NME/NM23 nucleoside diphosphate kinase 4	protein_coding	NP_001273362.1;NP_001273364.1;	-7.2
TOM1	target of myb1 membrane trafficking protein	protein_coding	NP_001129201.1;NP_001129202.1;	-7.2
LTBR	lymphotoxin beta receptor	protein_coding	NP_001257916.1;NP_002333.1;XP_	-7.2
ACBD4	acyl-CoA binding domain containing 4	protein_coding	NP_001129177.1;NP_001129178.1;	-7.2
TBC1D17	TBC1 domain family member 17	protein_coding	NP_001161694.1;NP_078958.2;XP_	-7.2
NAGPA	N-acetylglucosamine-1-phosphodiester alpha-N-ac	protein_coding	NP_057340.2;XP_011520819.1	-7.2
ERCC2	ERCC excision repair 2, TFIIH core complex helic	protein_coding	NP_000391.1;NP_001124339.1;XP_	-7.2
MAPK12	mitogen-activated protein kinase 12	protein_coding	NP_001290181.1;NP_002960.2	-7.2
SETD1A	SET domain containing 1A, histone lysine methylt	protein_coding	NP_055527.1;XP_005255780.1;XP_	-7.2
UBL4A	ubiquitin like 4A	protein_coding	NP_055050.1	-7.2
ZC3H4	zinc finger CCCH-type containing 4	protein_coding	NP_055983.1;XP_005258733.1;XP_	-7.2
KDM6B	lysine demethylase 6B	protein_coding	NP_001073893.1;NP_001335645.1;	-7.2
SIRT2	sirtuin 2	protein_coding	NP_001180215.1;NP_036369.2;NP_	-7.2
PDXP	pyridoxal phosphatase	protein_coding	NP_064711.1	-7.2
LOC108783654	uncharacterized LOC108783654	lncRNA	.	-7.2
EPOR	erythropoietin receptor	protein_coding	NP_000112.1	-7.3
CARD19	caspase recruitment domain family member 19	protein_coding	NP_001304939.1;NP_001304940.1;	-7.3
VAT1	vesicle amine transport 1	protein_coding	NP_006364.2	-7.3
IP6K3	inositol hexakisphosphate kinase 3	protein_coding	NP_001136355.1;NP_473452.2;XP_	-7.3
NR2F6	nuclear receptor subfamily 2 group F member 6	protein_coding	NP_005225.2	-7.3
CPZ	carboxypeptidase Z	protein_coding	NP_001014447.2;NP_001014448.1;	-7.3
CDKN2C	cyclin dependent kinase inhibitor 2C	protein_coding	NP_001253.1;NP_523240.1	-7.3
BAX	BCL2 associated X, apoptosis regulator	protein_coding	NP_001278357.1;NP_001278358.1;	-7.3
LPAR5	lysophosphatidic acid receptor 5	protein_coding	NP_001136433.1;NP_065133.1	-7.3
ZBTB4	zinc finger and BTB domain containing 4	protein_coding	NP_001122305.1;NP_065950.2;XP_	-7.3
FAM160B2	family with sequence similarity 160 member B2	protein_coding	NP_001341179.1;NP_001341180.1;	-7.3
S1PR2	sphingosine-1-phosphate receptor 2	protein_coding	NP_004221.3	-7.3
ZNF408	zinc finger protein 408	protein_coding	NP_001171680.1;NP_079017.1	-7.3
KLHDC3	kelch domain containing 3	protein_coding	NP_476502.1;XP_024302088.1	-7.3
ARRDC1	arrestin domain containing 1	protein_coding	NP_001304897.1;NP_689498.1;XP_	-7.3
MTA1	metastasis associated 1	protein_coding	NP_001190187.1;NP_004680.2;XP_	-7.3
GPR157	G protein-coupled receptor 157	protein_coding	NP_079256.4;XP_005263553.1;XP_	-7.3
CAPN10	calpain 10	protein_coding	NP_075571.2;NP_075573.3	-7.3
TEAD2	TEA domain transcription factor 2	protein_coding	NP_001243587.1;NP_001243588.1;	-7.3
H3C4	H3 clustered histone 4	protein_coding	NP_001363866.1;NP_003521.2	-7.3
KCNN4	potassium calcium-activated channel subfamily N	protein_coding	NP_002241.1;XP_005258939.1;XP_	-7.3
HDAC7	histone deacetylase 7	protein_coding	NP_001091886.1;NP_001295019.1;	-7.4
SPATA20	spermatogenesis associated 20	protein_coding	NP_001245301.1;NP_001245302.1;	-7.4
FAM207A	family with sequence similarity 207 member A	protein_coding	NP_001303912.1;NP_001303913.1;	-7.4
INTS11	integrator complex subunit 11	protein_coding	NP_001243385.1;NP_001243389.1;	-7.4
ARHGEF17	Rho guanine nucleotide exchange factor 17	protein_coding	NP_055601.2;XP_016874112.1;XP_	-7.4
SLC4A11	solute carrier family 4 member 11	protein_coding	NP_001167560.1;NP_001167561.1;	-7.4
IL17RC	interleukin 17 receptor C	protein_coding	NP_001190192.2;NP_001190193.1;	-7.4
GTPBP6	GTP binding protein 6 (putative)	protein_coding	NP_036359.3;XP_006724510.1;XP_	-7.4
ZNF316	zinc finger protein 316	protein_coding	NP_001265488.1;XP_006715693.1;	-7.4
AMIGO3	adhesion molecule with Ig like domain 3	protein_coding	NP_942015.1	-7.4
MMP17	matrix metallopeptidase 17	protein_coding	NP_057239.4;XP_011536657.1;XP_	-7.4
H2AC12	H2A clustered histone 12	protein_coding	NP_542163.1	-7.4
TOLLIP	toll interacting protein	protein_coding	NP_001305441.1;NP_001305443.1;	-7.4
RBM10	RNA binding motif protein 10	protein_coding	NP_001191395.1;NP_001191396.1;	-7.4
RAB11FIP5	RAB11 family interacting protein 5	protein_coding	NP_001358201.1;NP_056285.1	-7.4
FOXK1	forkhead box K1	protein_coding	NP_001032242.1	-7.4
TRIM46	tripartite motif containing 46	protein_coding	NP_001243528.1;NP_001243530.1;	-7.5
MLF2	myeloid leukemia factor 2	protein_coding	NP_001369154.1;NP_001369155.1;	-7.5
C1orf122	chromosome 1 open reading frame 122	protein_coding	NP_001136198.1;NP_940848.2	-7.5
IL27RA	interleukin 27 receptor subunit alpha	protein_coding	NP_004834.1	-7.5
DOK1	docking protein 1	protein_coding	NP_001184189.1;NP_001305795.1;	-7.5
PUSL1	pseudouridine synthase like 1	protein_coding	NP_001333045.1;NP_699170.1;XP_	-7.5
CDKN1A	cyclin dependent kinase inhibitor 1A	protein_coding	NP_000380.1;NP_001207706.1;NP_	-7.5

TICAM1	toll like receptor adaptor molecule 1	protein_coding	NP_891549.1	-7.5
MAPK3	mitogen-activated protein kinase 3	protein_coding	NP_001035145.1;NP_001103361.1;	-7.5
CHST14	carbohydrate sulfotransferase 14	protein_coding	NP_569735.1	-7.5
SIRT6	sirtuin 6	protein_coding	NP_001180214.1;NP_001307987.1;	-7.5
SHISA5	shisa family member 5	protein_coding	NP_001258994.1;NP_001258995.1;	-7.5
B4GALT7	beta-1,4-galactosyltransferase 7	protein_coding	NP_009186.1;XP_006714879.1;XP_	-7.5
ZNF444	zinc finger protein 444	protein_coding	NP_001240721.1;NP_060807.2;XP_	-7.5
LIMD2	LIM domain containing 2	protein_coding	NP_085053.1;XP_005257760.1;XP_	-7.5
TNK2	tyrosine kinase non receptor 2	protein_coding	NP_001010938.2;NP_001294975.1;	-7.5
SELENBP1	selenium binding protein 1	protein_coding	NP_001245217.1;NP_001245218.1;	-7.5
ADORA2A	adenosine A2a receptor	protein_coding	NP_000666.2;NP_001265426.1;NP_	-7.5
PIK3C2B	phosphatidylinositol-4-phosphate 3-kinase catalyti	protein_coding	NP_001364263.1;NP_001364264.1;	-7.5
PLEKHJ1	pleckstrin homology domain containing J1	protein_coding	NP_001287765.1;NP_060519.1;XP_	-7.6
NKIRAS2	NFKB inhibitor interacting Ras like 2	protein_coding	NP_001001349.1;NP_001138399.1;	-7.6
COL15A1	collagen type XV alpha 1 chain	protein_coding	NP_001846.3;XP_011516516.1	-7.6
CHTF8	chromosome transmission fidelity factor 8	protein_coding	NP_001034779.1;NP_001035236.1	-7.6
TFAP4	transcription factor AP-4	protein_coding	NP_003214.1;XP_011520935.1;XP_	-7.6
BCAS4	breast carcinoma amplified sequence 4	protein_coding	NP_001010974.1;NP_060313.3;NP_	-7.6
GPX4	glutathione peroxidase 4	protein_coding	NP_001034936.1;NP_001034937.1;	-7.6
OGFOD3	2-oxoglutarate and iron dependent oxygenase dom	protein_coding	NP_078924.1;NP_787098.3	-7.6
ATXN7L3	ataxin 7 like 3	protein_coding	NP_001369237.1;NP_001369238.1;	-7.6
SNRPB	small nuclear ribonucleoprotein polypeptides B an	protein_coding	NP_003082.1;NP_937859.1	-7.6
CYC1	cytochrome c1	protein_coding	NP_001907.3;XP_016868591.1;XP_	-7.6
CTDP1	CTD phosphatase subunit 1	protein_coding	NP_001189433.1;NP_001305440.1;	-7.6
DTYMK	deoxythymidylate kinase	protein_coding	NP_001158503.1;NP_001307831.1;	-7.6
POR	cytochrome p450 oxidoreductase	protein_coding	NP_000932.3;NP_001354491.1;NP_	-7.6
INTS5	integrator complex subunit 5	protein_coding	NP_085131.1	-7.6
RFX2	regulatory factor X2	protein_coding	NP_000626.2;NP_602309.1;XP_01	-7.6
FBXL6	F-box and leucine rich repeat protein 6	protein_coding	NP_036294.2;NP_078831.4	-7.6
DCAF15	DDB1 and CUL4 associated factor 15	protein_coding	NP_612362.2	-7.6
TMEM86B	transmembrane protein 86B	protein_coding	NP_001358942.1;NP_776165.3	-7.6
MYO1C	myosin IC	protein_coding	NP_001074248.1;NP_001074419.1;	-7.6
KMT2B	lysine methyltransferase 2B	protein_coding	NP_055542.1;XP_011525863.2;XP_	-7.6
ARTN	artemin	protein_coding	NP_001129687.1;NP_476431.2;NP_	-7.6
PEMT	phosphatidylethanolamine N-methyltransferase	protein_coding	NP_001254480.1;NP_001254481.1;	-7.6
TACC3	transforming acidic coiled-coil containing protein	protein_coding	NP_006333.1;XP_005247986.1;XP_	-7.6
KLHL25	kelch like family member 25	protein_coding	NP_071925.2	-7.7
TAF6	TATA-box binding protein associated factor 6	protein_coding	NP_001177344.1;NP_001351927.1;	-7.7
TECR	trans-2,3-enoyl-CoA reductase	protein_coding	NP_001308099.1;NP_612510.1;XP_	-7.7
DVL2	dishevelled segment polarity protein 2	protein_coding	NP_004413.1;XP_005256559.1	-7.7
R3HCC1	R3H domain and coiled-coil containing 1	protein_coding	NP_001129580.2;NP_001288579.1	-7.7
NOG	noggin	protein_coding	NP_005441.1	-7.7
SNX8	sorting nexin 8	protein_coding	NP_037453.1;XP_011513631.1;XP_	-7.7
RASA4	RAS p21 protein activator 4	protein_coding	NP_001073346.2;NP_008920.5;XP_	-7.7
TBC1D16	TBC1 domain family member 16	protein_coding	NP_001258773.1;NP_001258774.1;	-7.7
TMC6	transmembrane channel like 6	protein_coding	NP_001120670.1;NP_001308114.1;	-7.7
FAM83G	family with sequence similarity 83 member G	protein_coding	NP_001035088.2;XP_016880442.1	-7.7
MVK	mevalonate kinase	protein_coding	NP_000422.1;NP_001107657.1;NP_	-7.7
RN7SL674P	RNA, 7SL, cytoplasmic 674, pseudogene	pseudogene	.	-7.7
E2F1	E2F transcription factor 1	protein_coding	NP_005216.1	-7.7
TXNRD2	thioredoxin reductase 2	protein_coding	NP_001269441.1;NP_001339229.1;	-7.7
HS1BP3	HCLS1 binding protein 3	protein_coding	NP_071905.3;XP_016860185.1;XP_	-7.7
SPSB2	splA/ryanodine receptor domain and SOCS box cc	protein_coding	NP_001139788.1;NP_001306599.1;	-7.7
CSPG4P12	chondroitin sulfate proteoglycan 4 pseudogene 12	pseudogene	.	-7.7
USF2	upstream transcription factor 2, c-fos interacting	protein_coding	NP_001308079.1;NP_003358.1;NP_	-7.7
CPT1A	carnitine palmitoyltransferase 1A	protein_coding	NP_001027017.1;NP_001867.2;XP_	-7.7
ROR2	receptor tyrosine kinase like orphan receptor 2	protein_coding	NP_001305133.1;NP_004551.2;XP_	-7.7
RASA4B	RAS p21 protein activator 4B	protein_coding	NP_001264264.1;NP_001354696.1;	-7.7
CNN1	calponin 1	protein_coding	NP_001290.2;NP_001295270.1;NP_	-7.7
CDC20	cell division cycle 20	protein_coding	NP_001246.2	-7.7
H2AC19	H2A clustered histone 19	protein_coding	NP_001035807.1	-7.7
CORO2A	coronin 2A	protein_coding	NP_003380.3;NP_438171.1;XP_01	-7.7
H2AC18	H2A clustered histone 18	protein_coding	NP_003507.1	-7.7
LOXL1	lysyl oxidase like 1	protein_coding	NP_005567.2;XP_011519857.1;XP_	-7.7
PYCR1	pyrroline-5-carboxylate reductase 1	protein_coding	NP_001269208.1;NP_001269209.1;	-7.7
DOCK6	dedicator of cytokinesis 6	protein_coding	NP_001354759.1;NP_065863.2;XP_	-7.7
YIF1A	Yip1 interacting factor homolog A, membrane tra	protein_coding	NP_001287790.1;NP_065203.2	-7.7
RTL8C	retrotransposon Gag like 8C	protein_coding	NP_001071639.1	-7.8
ZNF787	zinc finger protein 787	protein_coding	NP_001002836.2;NP_001338611.1;	-7.8
DCXR	dicarbonyl and L-xylulose reductase	protein_coding	NP_001182147.1;NP_057370.1	-7.8
MOB3A	MOB kinase activator 3A	protein_coding	NP_570719.1;XP_011525985.1;XP_	-7.8

AMOTL2	angiomotin like 2	protein_coding	NP_001265612.1;NP_001265614.1;	-7.8
CDC25B	cell division cycle 25B	protein_coding	NP_001274445.1;NP_001274446.1;	-7.8
HDAC5	histone deacetylase 5	protein_coding	NP_001015053.1;NP_001369322.1;	-7.8
ABHD8	abhydrolase domain containing 8	protein_coding	NP_078803.4	-7.8
SRC	SRC proto-oncogene, non-receptor tyrosine kinase	protein_coding	NP_005408.1;NP_938033.1;XP_01	-7.8
ZC3H18	zinc finger CCCH-type containing 18	protein_coding	NP_001281269.1;NP_653205.3;XP_	-7.8
STXBP2	syntaxin binding protein 2	protein_coding	NP_001120868.1;NP_001258963.1;	-7.8
EXD3	exonuclease 3'-5' domain containing 3	protein_coding	NP_001273752.1;NP_060290.3;XP_	-7.8
NF2	neurofibromin 2	protein_coding	NP_000259.1;NP_057502.2;NP_86	-7.8
EFEMP2	EGF containing fibulin extracellular matrix protei	protein_coding	NP_058634.4	-7.8
TFAP2A	transcription factor AP-2 alpha	protein_coding	NP_001027451.1;NP_001035890.1;	-7.8
SERPINH1	serpin family H member 1	protein_coding	NP_001193943.1;NP_001226.2;XP_	-7.8
EEF2	eukaryotic translation elongation factor 2	protein_coding	NP_001952.1	-7.8
HYAL2	hyaluronidase 2	protein_coding	NP_003764.3;NP_149348.2;XP_00	-7.8
CBX2	chromobox 2	protein_coding	NP_005180.1;NP_116036.1;XP_01	-7.8
ABCB9	ATP binding cassette subfamily B member 9	protein_coding	NP_001229942.1;NP_001229943.1;	-7.8
PDE4A	phosphodiesterase 4A	protein_coding	NP_001104777.1;NP_001104778.1;	-7.8
ZNF646	zinc finger protein 646	protein_coding	NP_055514.3;XP_005255767.1;XP_	-7.8
SNORD3B-2	small nucleolar RNA, C/D box 3B-2	snoRNA	.	-7.9
KDELR1	KDEL endoplasmic reticulum protein retention re	protein_coding	NP_006792.1	-7.9
TRIM8	tripartite motif containing 8	protein_coding	NP_001332879.1;NP_112174.2	-7.9
SELENOO	selenoprotein O	protein_coding	NP_113642.1	-7.9
LOC112268288	uncharacterized LOC112268288, transcript varian	lncRNA	.	-7.9
TRAF7	TNF receptor associated factor 7	protein_coding	NP_115647.2;XP_005255684.1;XP_	-7.9
CD81	CD81 molecule	protein_coding	NP_001284578.1;NP_004347.1;XP_	-7.9
LBX2	ladybird homeobox 2	protein_coding	NP_001009812.1;NP_001269359.1	-7.9
MRTFA	myocardin related transcription factor A	protein_coding	NP_001269589.1;NP_001269590.2;	-7.9
ZNF282	zinc finger protein 282	protein_coding	NP_001290410.1;NP_003566.1;XP_	-7.9
SCARA3	scavenger receptor class A member 3	protein_coding	NP_057324.2;NP_878185.1;XP_01	-7.9
CCDC85A	coiled-coil domain containing 85A	protein_coding	NP_001073902.1;NP_001335441.1;	-7.9
ESS2	ess-2 splicing factor homolog	protein_coding	NP_073210.1;XP_005261339.1;XP_	-7.9
DNAJB2	DnaJ heat shock protein family (Hsp40) member I	protein_coding	NP_001034639.1;NP_006727.2	-7.9
ADCK2	aarF domain containing kinase 2	protein_coding	NP_443085.2;XP_006716233.1;XP_	-7.9
KEAP1	kelch like ECH associated protein 1	protein_coding	NP_036421.2;NP_987096.1;XP_00	-7.9
XAB2	XPA binding protein 2	protein_coding	NP_064581.2	-7.9
TRPV2	transient receptor potential cation channel subfami	protein_coding	NP_057197.2;XP_005256733.1;XP_	-7.9
GAS1	growth arrest specific 1	protein_coding	NP_002039.2	-7.9
VPS18	VPS18 core subunit of CORVET and HOPS comp	protein_coding	NP_065908.1;XP_011520145.1;XP_	-7.9
NRGN	neurogranin	protein_coding	NP_001119653.1;NP_006167.1	-7.9
SLC44A2	solute carrier family 44 member 2	protein_coding	NP_001138528.1;NP_001350540.1;	-7.9
NDOR1	NADPH dependent diflavin oxidoreductase 1	protein_coding	NP_001137498.1;NP_001137499.1;	-7.9
BCAT2	branched chain amino acid transaminase 2	protein_coding	NP_001158245.1;NP_001181.2;NP_	-7.9
SLC38A10	solute carrier family 38 member 10	protein_coding	NP_001033073.1;NP_612637.1;XP_	-7.9
CIRBP-AS1	CIRBP antisense RNA 1	lncRNA	.	-8.0
FARSA	phenylalanyl-tRNA synthetase subunit alpha	protein_coding	NP_004452.1	-8.0
DNPH1	2'-deoxynucleoside 5'-phosphate N-hydrolase 1	protein_coding	NP_006434.1;NP_954653.1	-8.0
TBC1D10A	TBC1 domain family member 10A	protein_coding	NP_001191169.1;NP_114143.1	-8.0
DUSP7	dual specificity phosphatase 7	protein_coding	NP_001938.2	-8.0
ARRDC1-AS1	ARRDC1 antisense RNA 1	lncRNA	.	-8.0
BCKDK	branched chain keto acid dehydrogenase kinase	protein_coding	NP_001116429.1;NP_001258855.1;	-8.0
CIAO3	cytosolic iron-sulfur assembly component 3	protein_coding	NP_001291728.1;NP_071938.1	-8.0
MSRB1	methionine sulfoxide reductase B1	protein_coding	NP_001369193.1;NP_001369194.1;	-8.0
NFKBIB	NFKB inhibitor beta	protein_coding	NP_001230045.1;NP_001356628.1;	-8.0
C6orf132	chromosome 6 open reading frame 132	protein_coding	NP_001157918.1	-8.0
KIF1C	kinesin family member 1C	protein_coding	NP_006603.2;XP_005256481.1	-8.0
SKI	SKI proto-oncogene	protein_coding	NP_003027.1;XP_005244832.1;XP_	-8.0
PDGFB	platelet derived growth factor subunit B	protein_coding	NP_002599.1;NP_148937.1	-8.0
MAN2B1	mannosidase alpha class 2B member 1	protein_coding	NP_000519.2;NP_001166969.1;XP_	-8.0
ANKRD9	ankyrin repeat domain 9	protein_coding	NP_001335580.1;NP_001335581.1;	-8.0
SCYL1	SCY1 like pseudokinase 1	protein_coding	NP_001041683.1;NP_065731.3;XP_	-8.1
NIBAN2	niban apoptosis regulator 2	protein_coding	NP_001030611.1;NP_073744.2;XP_	-8.1
GDPGP1	GDP-D-glucose phosphorylase 1	protein_coding	NP_001013679.2;NP_001309740.1	-8.1
ZNF777	zinc finger protein 777	protein_coding	NP_056509.2;XP_005250037.1;XP_	-8.1
NUDT18	nudix hydrolase 18	protein_coding	NP_079091.3;XP_011542952.1	-8.1
CDK2AP2	cyclin dependent kinase 2 associated protein 2	protein_coding	NP_001258778.1;NP_005842.1	-8.1
TAGLN2	transgelin 2	protein_coding	NP_001264152.1;NP_001264153.1;	-8.1
RHBDD3	rhomboid domain containing 3	protein_coding	NP_001316465.1;NP_036397.1;XP_	-8.1
HLX	H2.0 like homeobox	protein_coding	NP_068777.1	-8.1
ZNF511	zinc finger protein 511	protein_coding	NP_665805.2	-8.1
ALDOA	aldolase, fructose-bisphosphate A	protein_coding	NP_001121089.1;NP_001230106.1;	-8.1
LINC01521	long intergenic non-protein coding RNA 1521	lncRNA	.	-8.1

ACOT7	acyl-CoA thioesterase 7	protein_coding	NP_009205.3;NP_863654.1;NP_86[illegible]	-8.1
RABEP2	rabaptin, RAB GTPase binding effector protein 2	protein_coding	NP_079092.2	-8.1
MXD3	MAX dimerization protein 3	protein_coding	NP_001136407.1;NP_112590.1	-8.1
ACTN4	actinin alpha 4	protein_coding	NP_001308962.1;NP_004915.2;XP_	-8.2
TCEA2	transcription elongation factor A2	protein_coding	NP_003186.1;NP_942016.1;XP_00[illegible]	-8.2
ZNF579	zinc finger protein 579	protein_coding	NP_689813.2;XP_016881898.1;XP_	-8.2
TYRO3	TYRO3 protein tyrosine kinase	protein_coding	NP_001317193.1;NP_006284.2;XP_	-8.2
H1-2	H1.2 linker histone, cluster member	protein_coding	NP_005310.1	-8.2
GPRIN1	G protein regulated inducer of neurite outgrowth 1	protein_coding	NP_443131.2	-8.2
SLC25A42	solute carrier family 25 member 42	protein_coding	NP_001308473.1;NP_848621.2;XP_	-8.2
PHLDA2	pleckstrin homology like domain family A membe	protein_coding	NP_003302.1	-8.2
MAP1A	microtubule associated protein 1A	protein_coding	NP_002364.5	-8.2
MAP2K3	mitogen-activated protein kinase kinase 3	protein_coding	NP_001303261.1;NP_002747.2;NP_	-8.2
NT5C	5', 3'-nucleotidase, cytosolic	protein_coding	NP_001239306.1;NP_055410.1;XP_	-8.2
NSMF	NMDA receptor synaptonuclear signaling and neu	protein_coding	NP_001124441.1;NP_001124442.1;	-8.2
PER1	period circadian regulator 1	protein_coding	NP_002607.2;XP_005256746.1;XP_	-8.2
SDF4	stromal cell derived factor 4	protein_coding	NP_057260.3;NP_057631.2;XP_01[illegible]	-8.2
TESK1	testis associated actin remodelling kinase 1	protein_coding	NP_001305159.1;NP_006276.2	-8.2
EPN1	epsin 1	protein_coding	NP_001123543.1;NP_001123544.1;	-8.2
RNF26	ring finger protein 26	protein_coding	NP_114404.1	-8.2
MAST3	microtubule associated serine/threonine kinase 3	protein_coding	NP_055831.1;XP_005259880.1;XP_	-8.2
OCEL1	occludin/ELL domain containing 1	protein_coding	NP_078854.1;XP_005260136.1;XP_	-8.2
H3C11	H3 clustered histone 11	protein_coding	NP_003524.1	-8.2
SLC39A13	solute carrier family 39 member 13	protein_coding	NP_001121697.2;NP_001317174.1;	-8.2
TECPR1	tectonin beta-propeller repeat containing 1	protein_coding	NP_056210.1;XP_005250310.1;XP_	-8.2
CLIP2	CAP-Gly domain containing linker protein 2	protein_coding	NP_003379.4;NP_115797.2	-8.2
LONP1	lon peptidase 1, mitochondrial	protein_coding	NP_001263408.1;NP_001263409.1;	-8.2
PHRF1	PHD and ring finger domains 1	protein_coding	NP_001273510.1;NP_001273511.1;	-8.3
FAM90A1	family with sequence similarity 90 member A1	protein_coding	NP_001306911.1;NP_060558.3;XP_	-8.3
XYLT2	xylosyltransferase 2	protein_coding	NP_071450.2;XP_005257629.1	-8.3
MFSD13A	major facilitator superfamily domain containing 1[illegible]	protein_coding	NP_079065.2;XP_006718036.1;XP_	-8.3
THAP4	THAP domain containing 4	protein_coding	NP_001157828.1;NP_057047.4;XP_	-8.3
SNORA105A	small nucleolar RNA, H/ACA box 105A	snoRNA	.	-8.3
CCDC102A	coiled-coil domain containing 102A	protein_coding	NP_149989.2;XP_011521771.1	-8.3
WNT10B	Wnt family member 10B	protein_coding	NP_003385.2;XP_011537024.1;XP_	-8.3
ANAPC2	anaphase promoting complex subunit 2	protein_coding	NP_037498.1	-8.3
BCL2L12	BCL2 like 12	protein_coding	NP_001035758.1;NP_001269445.1;	-8.3
PRKCSH	protein kinase C substrate 80K-H	protein_coding	NP_001001329.1;NP_001276031.1;	-8.3
FAM110A	family with sequence similarity 110 member A	protein_coding	NP_001035812.1;NP_001276074.1;	-8.3
GSK3A	glycogen synthase kinase 3 alpha	protein_coding	NP_063937.2	-8.4
NRSN2	neurensin 2	protein_coding	NP_001310608.1;NP_001310609.1;	-8.4
U2AF2	U2 small nuclear RNA auxiliary factor 2	protein_coding	NP_001012496.1;NP_009210.1;XP_	-8.4
TRAPPC5	trafficking protein particle complex 5	protein_coding	NP_001035926.1;NP_001035927.1;	-8.4
PRICKLE3	prickle planar cell polarity protein 3	protein_coding	NP_001294908.1;NP_006141.2	-8.4
FPGS	folylpolyglutamate synthase	protein_coding	NP_001018088.1;NP_001275732.1;	-8.4
PIEZO1	piezo type mechanosensitive ion channel compone	protein_coding	NP_001136336.2	-8.4
OXLD1	oxidoreductase like domain containing 1	protein_coding	NP_001034931.1;NP_001291923.1;	-8.4
PTPN23	protein tyrosine phosphatase non-receptor type 23	protein_coding	NP_001291411.1;NP_056281.1;XP_	-8.4
RNF44	ring finger protein 44	protein_coding	NP_055716.1;XP_005265897.1;XP_	-8.4
INF2	inverted formin 2	protein_coding	NP_001026884.3;NP_071934.3;NP_	-8.4
ZNF319	zinc finger protein 319	protein_coding	NP_065858.1;XP_005256126.1;XP_	-8.4
IPO4	importin 4	protein_coding	NP_078934.3	-8.4
LLGL2	LLGL scribble cell polarity complex component 2	protein_coding	NP_001015002.1;NP_001026973.1;	-8.4
PTDSS2	phosphatidylserine synthase 2	protein_coding	NP_001316473.1;NP_001316474.1;	-8.4
NOL4L	nucleolar protein 4 like	protein_coding	NP_001243727.1;NP_001338609.1;	-8.4
MGAT4B	alpha-1,3-mannosyl-glycoprotein 4-beta-N-acetylg	protein_coding	NP_055090.1;NP_463459.1;XP_02[illegible]	-8.4
DUS1L	dihydrouridine synthase 1 like	protein_coding	NP_071439.3;XP_005256450.1;XP_	-8.4
CSNK1G2	casein kinase 1 gamma 2	protein_coding	NP_001310.3;XP_005259555.1;XP_	-8.5
SLC9A3R1	SLC9A3 regulator 1	protein_coding	NP_004243.1	-8.5
DMWD	DM1 locus, WD repeat containing	protein_coding	NP_004934.1	-8.5
SLC39A3	solute carrier family 39 member 3	protein_coding	NP_653165.2;NP_998733.1	-8.5
FOXRED2	FAD dependent oxidoreductase domain containing	protein_coding	NP_001095841.1;NP_001349970.1;	-8.5
SDC1	syndecan 1	protein_coding	NP_001006947.1;NP_002988.4;XP_	-8.5
RAB20	RAB20, member RAS oncogene family	protein_coding	NP_060287.1	-8.5
MYADM	myeloid associated differentiation marker	protein_coding	NP_001018654.1;NP_001018655.1;	-8.5
PRELP	proline and arginine rich end leucine rich repeat p[illegible]	protein_coding	NP_002716.1;NP_958505.1	-8.5
TNKS1BP1	tankyrase 1 binding protein 1	protein_coding	NP_203754.2;XP_006718788.1;XP_	-8.5
DPP9	dipeptidyl peptidase 9	protein_coding	NP_001352916.1;NP_631898.3;XP_	-8.5
MAGEE1	MAGE family member E1	protein_coding	NP_065983.1	-8.5
SMPD1	sphingomyelin phosphodiesterase 1	protein_coding	NP_000534.3;NP_001007594.2;NP_	-8.5
CCDC9	coiled-coil domain containing 9	protein_coding	NP_056418.1;XP_016882067.1	-8.5

C19orf25	chromosome 19 open reading frame 25	protein_coding	NP_689695.2;XP_005259563.1;XP_	-8.5
PELP1	proline, glutamate and leucine rich protein 1	protein_coding	NP_001265170.1;NP_055204.4	-8.5
PTGES	prostaglandin E synthase	protein_coding	NP_004869.1	-8.5
SMTN	smoothelin	protein_coding	NP_001193946.1;NP_001193947.1;	-8.6
EPS8L1	EPS8 like 1	protein_coding	NP_060199.3;NP_573441.2;XP_00	-8.6
KRBA1	KRAB-A domain containing 1	protein_coding	NP_001277116.1;NP_115923.2;XP_	-8.6
ANKRD13B	ankyrin repeat domain 13B	protein_coding	NP_689558.4;XP_005257977.1;XP_	-8.6
LOC101928274	uncharacterized LOC101928274, transcript varian	lncRNA	.	-8.6
MBD6	methyl-CpG binding domain protein 6	protein_coding	NP_443129.3;XP_005268674.1;XP_	-8.6
MEIS3	Meis homeobox 3	protein_coding	NP_001009813.1;NP_001287988.1;	-8.6
MRI1	methylthioribose-1-phosphate isomerase 1	protein_coding	NP_001026897.1;NP_001316501.1;	-8.6
CARM1	coactivator associated arginine methyltransferase 1	protein_coding	NP_001357017.1;NP_001357018.1;	-8.6
EXOSC4	exosome component 4	protein_coding	NP_061910.1;XP_011515436.1	-8.6
TRAPPC6A	trafficking protein particle complex 6A	protein_coding	NP_001257820.1;NP_001257821.1;	-8.6
AMH	anti-Mullerian hormone	protein_coding	NP_000470.3	-8.6
UAP1L1	UDP-N-acetylglucosamine pyrophosphorylase 1 li	protein_coding	NP_997192.2;XP_006717380.1;XP_	-8.6
EME2	essential meiotic structure-specific endonuclease s	protein_coding	NP_001244299.1;XP_016878514.1;	-8.7
FHL3	four and a half LIM domains 3	protein_coding	NP_001230807.1;NP_004459.2;XP_	-8.7
ARL6IP4	ADP ribosylation factor like GTPase 6 interacting	protein_coding	NP_001002251.2;NP_001002252.2;	-8.7
H1-3	H1.3 linker histone, cluster member	protein_coding	NP_005311.1	-8.7
TRABD	TraB domain containing	protein_coding	NP_001307413.1;NP_001307414.1;	-8.7
RRP9	ribosomal RNA processing 9, U3 small nucleolar 1	protein_coding	NP_004695.1	-8.7
PCNX3	pecanex 3	protein_coding	NP_115599.2;XP_005274044.1;XP_	-8.7
SRF	serum response factor	protein_coding	NP_001278930.1;NP_003122.1	-8.7
MEN1	menin 1	protein_coding	NP_000235.2;NP_001357180.1;NP_	-8.7
CAVIN3	caveolae associated protein 3	protein_coding	NP_659477.2	-8.7
SREBF1	sterol regulatory element binding transcription fac	protein_coding	NP_001005291.1;NP_001308025.1;	-8.7
GMIP	GEM interacting protein	protein_coding	NP_001275927.1;NP_001275928.1;	-8.7
BAP1	BRCA1 associated protein 1	protein_coding	NP_004647.1;XP_011532451.1;XP_	-8.7
H3C10	H3 clustered histone 10	protein_coding	NP_003527.1	-8.7
UQCC3	ubiquinol-cytochrome c reductase complex asseml	protein_coding	NP_001078841.1	-8.7
IGFBP4	insulin like growth factor binding protein 4	protein_coding	NP_001543.2	-8.8
CDC20P1	cell division cycle 20 pseudogene 1	pseudogene	.	-8.8
TSPAN4	tetraspanin 4	protein_coding	NP_001020405.1;NP_001020406.1;	-8.8
INAFM1	InaF motif containing 1	protein_coding	NP_848606.3	-8.8
CADM4	cell adhesion molecule 4	protein_coding	NP_660339.1;XP_005258677.1;XP_	-8.8
MTFP1	mitochondrial fission process 1	protein_coding	NP_001003704.1;NP_057582.2	-8.8
DTX2	deltex E3 ubiquitin ligase 2	protein_coding	NP_001096064.1;NP_001096065.1;	-8.8
TARBP2	TARBP2 subunit of RISC loading complex	protein_coding	NP_004169.3;NP_599150.1;NP_59	-8.8
E2F2	E2F transcription factor 2	protein_coding	NP_004082.1;XP_005245805.1;XP_	-8.8
MEGF8	multiple EGF like domains 8	protein_coding	NP_001258867.1;NP_001401.2	-8.8
MZT2B	mitotic spindle organizing protein 2B	protein_coding	NP_001317211.1;NP_001317213.1;	-8.9
CDC42EP4	CDC42 effector protein 4	protein_coding	NP_036253.2;XP_005257239.1	-8.9
SLC25A11	solute carrier family 25 member 11	protein_coding	NP_001158889.1;NP_001158890.1;	-8.9
CCNF	cyclin F	protein_coding	NP_001310467.1;NP_001752.2	-8.9
C8orf82	chromosome 8 open reading frame 82	protein_coding	NP_001001795.1	-8.9
STRN4	striatin 4	protein_coding	NP_001034966.1;NP_037535.2;XP_	-8.9
MAFG-DT	MAFG divergent transcript	lncRNA	.	-8.9
MVP	major vault protein	protein_coding	NP_001280133.1;NP_001280134.1;	-8.9
CCDC124	coiled-coil domain containing 124	protein_coding	NP_001129675.1;NP_612451.1	-8.9
FCGRT	Fc fragment of IgG receptor and transporter	protein_coding	NP_001129491.1;NP_004098.1	-8.9
RARA	retinoic acid receptor alpha	protein_coding	NP_000955.1;NP_001019980.1;NP_	-8.9
COQ8B	coenzyme Q8B	protein_coding	NP_001136027.1;NP_079152.3	-8.9
GNB1L	G protein subunit beta 1 like	protein_coding	NP_443730.1	-8.9
ATG9A	autophagy related 9A	protein_coding	NP_001070666.1;NP_076990.4	-8.9
ABCA2	ATP binding cassette subfamily A member 2	protein_coding	NP_001597.2;NP_997698.1;XP_00	-8.9
SOGA1	suppressor of glucose, autophagy associated 1	protein_coding	NP_542194.2;NP_954650.2	-9.0
MAFK	MAF bZIP transcription factor K	protein_coding	NP_002351.1;XP_005249908.2;XP_	-9.0
AGAP3	ArfGAP with GTPase domain, ankyrin repeat and	protein_coding	NP_001036000.1;NP_001268229.1;	-9.0
PREX1	phosphatidylinositol-3,4,5-trisphosphate dependen	protein_coding	NP_065871.3;XP_011527236.1	-9.0
RRP7A	ribosomal RNA processing 7 homolog A	protein_coding	NP_056518.2	-9.0
FAAP20	FA core complex associated protein 20	protein_coding	NP_001139782.1;NP_001243874.2;	-9.0
CD276	CD276 molecule	protein_coding	NP_001019907.1;NP_001316557.1;	-9.0
ERBB2	erb-b2 receptor tyrosine kinase 2	protein_coding	NP_001005862.1;NP_001276865.1;	-9.0
TAGLN	transgelin	protein_coding	NP_001001522.1;NP_003177.2	-9.0
RPS6KB2	ribosomal protein S6 kinase B2	protein_coding	NP_003943.2;XP_005274221.1;XP_	-9.0
NLRX1	NLR family member X1	protein_coding	NP_001269072.1;NP_001269073.1;	-9.0
TRPM4	transient receptor potential cation channel subfami	protein_coding	NP_001182156.1;NP_001308210.1;	-9.0
ENG	endoglin	protein_coding	NP_000109.1;NP_001108225.1;NP_	-9.0
SETD1B	SET domain containing 1B, histone lysine methylt	protein_coding	NP_001340274.1;XP_006719359.1;	-9.1
CERCAM	cerebral endothelial cell adhesion molecule	protein_coding	NP_001273689.1;NP_057258.3;XP_	-9.1

FSD1	fibronectin type III and SPRY domain containing	protein_coding	NP_001317358.1;NP_077309.1	-9.1
SPC24	SPC24 component of NDC80 kinetochore comple:	protein_coding	NP_001303960.1;NP_001303961.1;	-9.1
PDLIM7	PDZ and LIM domain 7	protein_coding	NP_005442.2;NP_976227.1;NP_99	-9.1
WRAP53	WD repeat containing antisense to TP53	protein_coding	NP_001137462.1;NP_001137463.1;	-9.1
PRADC1	protease associated domain containing 1	protein_coding	NP_115695.1	-9.1
HOXA3	homeobox A3	protein_coding	NP_109377.1;NP_705895.1;XP_00	-9.1
EFNB1	ephrin B1	protein_coding	NP_004420.1	-9.1
ZBTB7A	zinc finger and BTB domain containing 7A	protein_coding	NP_001304919.1;NP_056982.1;XP_	-9.1
FHOD1	formin homology 2 domain containing 1	protein_coding	NP_001305131.1;NP_037373.2;XP_	-9.1
ECI1	enoyl-CoA delta isomerase 1	protein_coding	NP_001171500.1;NP_001910.2	-9.1
RAB1B	RAB1B, member RAS oncogene family	protein_coding	NP_112243.1;XP_016873867.1	-9.1
CCND3	cyclin D3	protein_coding	NP_001129489.1;NP_001129597.1;	-9.2
SYNGR2	synaptogyrin 2	protein_coding	NP_001307452.1;NP_001350707.1;	-9.2
PLXNA1	plexin A1	protein_coding	NP_115618.3;XP_011511210.1	-9.2
ULK1	unc-51 like autophagy activating kinase 1	protein_coding	NP_003556.2;XP_011537100.1;XP_	-9.2
CHERP	calcium homeostasis endoplasmic reticulum protei	protein_coding	NP_006378.3	-9.2
SHISA4	shisa family member 4	protein_coding	NP_937792.2	-9.2
GUK1	guanylate kinase 1	protein_coding	NP_000849.1;NP_001152862.1;NP_	-9.2
GCAT	glycine C-acetyltransferase	protein_coding	NP_001165161.1;NP_055106.1;XP_	-9.2
MYBL2	MYB proto-oncogene like 2	protein_coding	NP_001265539.1;NP_002457.1	-9.2
ATAD3B	ATPase family AAA domain containing 3B	protein_coding	NP_001304167.1;NP_114127.3;XP_	-9.2
CTBP1	C-terminal binding protein 1	protein_coding	NP_001012632.1;NP_001319.1;NP_	-9.2
EPHX1	epoxide hydrolase 1	protein_coding	NP_000111.1;NP_001129490.1;NP_	-9.2
ZBTB46	zinc finger and BTB domain containing 46	protein_coding	NP_001356670.1;NP_079500.2;XP_	-9.2
SEMA6C	semaphorin 6C	protein_coding	NP_001171532.1;NP_001171533.1;	-9.2
PPM1F	protein phosphatase, Mg2+/Mn2+ dependent 1F	protein_coding	NP_055449.1;XP_011528858.1;XP_	-9.2
ZC3H7B	zinc finger CCCH-type containing 7B	protein_coding	NP_060060.3;XP_011528318.1;XP_	-9.2
RAB11B	RAB11B, member RAS oncogene family	protein_coding	NP_004209.2	-9.3
MRPL28	mitochondrial ribosomal protein L28	protein_coding	NP_006419.2;XP_005255098.1;XP_	-9.3
TBC1D10B	TBC1 domain family member 10B	protein_coding	NP_056342.3;XP_011544091.1;XP_	-9.3
SHROOM1	shroom family member 1	protein_coding	NP_001166171.1;NP_597713.2;XP_	-9.3
TIGD3	tigger transposable element derived 3	protein_coding	NP_663771.1	-9.3
PDZD7	PDZ domain containing 7	protein_coding	NP_001182192.1;NP_001337973.1;	-9.3
ZNF865	zinc finger protein 865	protein_coding	NP_001182534.1	-9.3
PPP1R14B	protein phosphatase 1 regulatory inhibitor subunit	protein_coding	NP_619634.1	-9.3
NATD1	N-acetyltransferase domain containing 1	protein_coding	NP_690878.2	-9.3
ARHGAP23	Rho GTPase activating protein 23	protein_coding	NP_001186346.1;XP_006722054.1;	-9.3
KDM4B	lysine demethylase 4B	protein_coding	NP_001357022.1;NP_001357023.1;	-9.3
PEX16	peroxisomal biogenesis factor 16	protein_coding	NP_004804.2;NP_476515.2	-9.3
TNXB	tenascin XB	protein_coding	NP_001352205.1;NP_061978.6;NP_	-9.3
PTBP1	polypyrimidine tract binding protein 1	protein_coding	NP_002810.1;NP_114367.1;NP_11	-9.3
DBN1	drebrin 1	protein_coding	NP_001350470.2;NP_001351080.2;	-9.3
CRYAB	crystallin alpha B	protein_coding	NP_001276736.1;NP_001276737.1;	-9.3
KIAA0930	KIAA0930	protein_coding	NP_001009880.1;NP_056079.1;XP_	-9.3
ITPK1	inositol-tetrakisphosphate 1-kinase	protein_coding	NP_001136065.1;NP_001136066.1;	-9.3
AIF1L	allograft inflammatory factor 1 like	protein_coding	NP_001172024.1;NP_001172025.1;	-9.3
BCL3	BCL3 transcription coactivator	protein_coding	NP_005169.2;XP_011525499.2;XP_	-9.4
C7orf50	chromosome 7 open reading frame 50	protein_coding	NP_001127867.1;NP_001127868.1;	-9.4
EHMT2	euchromatic histone lysine methyltransferase 2	protein_coding	NP_001276342.1;NP_001305762.1;	-9.4
SH3GLB2	SH3 domain containing GRB2 like, endophilin B2	protein_coding	NP_001273974.1;NP_001273975.1;	-9.4
COASY	Coenzyme A synthase	protein_coding	NP_001035994.1;NP_001035997.2;	-9.4
TMEM25	transmembrane protein 25	protein_coding	NP_001137506.1;NP_001137507.1;	-9.4
TIMM17B	translocase of inner mitochondrial membrane 17B	protein_coding	NP_001161419.1;NP_005825.1	-9.4
SSH3	slingshot protein phosphatase 3	protein_coding	NP_060327.3;XP_016873431.1	-9.4
PDGFRB	platelet derived growth factor receptor beta	protein_coding	NP_001341945.1;NP_001341946.1;	-9.4
TMEM259	transmembrane protein 259	protein_coding	NP_001028198.1;NP_219488.1;XP_	-9.4
ZFP41	ZFP41 zinc finger protein	protein_coding	NP_001258085.3;NP_776193.3	-9.5
RNF187	ring finger protein 187	protein_coding	NP_001010858.2	-9.5
BSG	basigin (Ok blood group)	protein_coding	NP_001309172.1;NP_001719.2;NP_	-9.5
KCTD11	potassium channel tetramerization domain contain	protein_coding	NP_001002914.1;NP_001350571.1	-9.5
PACS2	phosphofurin acidic cluster sorting protein 2	protein_coding	NP_001094383.2;NP_001230056.1;	-9.5
ZNF446	zinc finger protein 446	protein_coding	NP_001291382.1;NP_060378.1	-9.5
C1QTNF2	C1q and TNF related 2	protein_coding	NP_001353433.1;NP_114114.3;XP_	-9.5
PTK7	protein tyrosine kinase 7 (inactive)	protein_coding	NP_001257327.1;NP_002812.2;NP_	-9.5
DLGAP4	DLG associated protein 4	protein_coding	NP_001035951.1;NP_001352550.1;	-9.5
LASP1	LIM and SH3 protein 1	protein_coding	NP_001258537.1;NP_006139.1	-9.5
PELI3	pellino E3 ubiquitin protein ligase family member	protein_coding	NP_001091980.1;NP_001230064.1;	-9.5
STARD10	StAR related lipid transfer domain containing 10	protein_coding	NP_006636.2	-9.5
ALDH4A1	aldehyde dehydrogenase 4 family member A1	protein_coding	NP_001154976.1;NP_001306147.1;	-9.5
CEP131	centrosomal protein 131	protein_coding	NP_001009811.2;NP_001306157.1;	-9.5
GPR17	G protein-coupled receptor 17	protein_coding	NP_001154887.1;NP_001154888.1;	-9.5

MAP3K6	mitogen-activated protein kinase kinase kinase 6	protein_coding	NP_001284538.1;NP_004663.3;XP_	-9.5
WIZ	WIZ zinc finger	protein_coding	NP_001317324.2;NP_001358518.1;	-9.6
ADCK5	aarF domain containing kinase 5	protein_coding	NP_777582.4;XP_006716590.1;XP_	-9.6
TST	thiosulfate sulfurtransferase	protein_coding	NP_001257412.1;NP_003303.2	-9.6
OGFR	opioid growth factor receptor	protein_coding	NP_031372.2	-9.6
CSK	C-terminal Src kinase	protein_coding	NP_001120662.1;NP_001341917.1;	-9.6
COL9A2	collagen type IX alpha 2 chain	protein_coding	NP_001843.1;XP_006710428.1;XP_	-9.6
DECR2	2,4-dienoyl-CoA reductase 2	protein_coding	NP_065715.1	-9.6
BRMS1	BRMS1 transcriptional repressor and anoikis regu	protein_coding	NP_001020128.1;NP_056214.1;XP_	-9.6
NUCB1	nucleobindin 1	protein_coding	NP_006175.2;XP_016882334.1	-9.6
DDX49	DEAD-box helicase 49	protein_coding	NP_061943.2;XP_011526385.1;XP_	-9.6
LRRC45	leucine rich repeat containing 45	protein_coding	NP_659436.1	-9.6
B4GALT2	beta-1,4-galactosyltransferase 2	protein_coding	NP_001005417.1;NP_003771.1;NP_	-9.6
PLCD1	phospholipase C delta 1	protein_coding	NP_001124436.1;NP_006216.2;XP_	-9.6
OLFML3	olfactomedin like 3	protein_coding	NP_001273281.1;NP_001273282.1;	-9.6
SH3GL1	SH3 domain containing GRB2 like 1, endophilin A	protein_coding	NP_001186872.1;NP_001186873.1;	-9.6
CALM3	calmodulin 3	protein_coding	NP_001316850.1;NP_001316851.1;	-9.6
CAPN5	calpain 5	protein_coding	NP_004046.2;XP_011543527.1;XP_	-9.6
GRINA	glutamate ionotropic receptor NMDA type subuni	protein_coding	NP_000828.1;NP_001009184.1	-9.6
LOC100130111	uncharacterized LOC100130111	lncRNA	.	-9.7
PLA2G15	phospholipase A2 group XV	protein_coding	NP_001350480.1;NP_036452.1;XP_	-9.7
PRKACA	protein kinase cAMP-activated catalytic subunit al	protein_coding	NP_001291278.1;NP_002721.1;NP_	-9.7
RFLNB	refilin B	protein_coding	NP_874364.1	-9.7
RIPOR1	RHO family interacting cell polarization regulator	protein_coding	NP_001180451.1;NP_001180452.1;	-9.7
MARK4	microtubule affinity regulating kinase 4	protein_coding	NP_001186796.1;NP_113605.2;XP_	-9.7
PSKH1	protein serine kinase H1	protein_coding	NP_006733.1	-9.7
MICOS13	mitochondrial contact site and cristae organizing s	protein_coding	NP_001295169.1;NP_001352690.1;	-9.7
KATNB1	katanin regulatory subunit B1	protein_coding	NP_005877.2	-9.7
JMJD4	jumonji domain containing 4	protein_coding	NP_001154937.1;NP_075383.2;XP_	-9.7
PEX10	peroxisomal biogenesis factor 10	protein_coding	NP_001361354.1;NP_001361355.1;	-9.7
AGTRAP	angiotensin II receptor associated protein	protein_coding	NP_001035284.1;NP_001035285.1;	-9.7
SLC25A22	solute carrier family 25 member 22	protein_coding	NP_001177989.1;NP_001177990.1;	-9.7
ZNF628	zinc finger protein 628	protein_coding	NP_149104.3;XP_005259428.1;XP_	-9.8
MYLK3	myosin light chain kinase 3	protein_coding	NP_001295230.1;NP_872299.2;XP_	-9.8
FAM53B	family with sequence similarity 53 member B	protein_coding	NP_055476.3	-9.8
NCS1	neuronal calcium sensor 1	protein_coding	NP_001122298.1;NP_055101.2	-9.8
SYDE1	synapse defective Rho GTPase homolog 1	protein_coding	NP_001287839.1;NP_149014.3;XP_	-9.8
GRWD1	glutamate rich WD repeat containing 1	protein_coding	NP_113673.3	-9.8
KHK	ketohexokinase	protein_coding	NP_000212.1;NP_006479.1;XP_00	-9.8
PLCB3	phospholipase C beta 3	protein_coding	NP_000923.1;NP_001171812.1;NP	-9.8
MBOAT7	membrane bound O-acyltransferase domain contai	protein_coding	NP_001139528.1;NP_001139554.1;	-9.8
AKT1	AKT serine/threonine kinase 1	protein_coding	NP_001014431.1;NP_001014432.1;	-9.8
FAM124A	family with sequence similarity 124 member A	protein_coding	NP_001229241.1;NP_001317451.1;	-9.8
WTIP	WT1 interacting protein	protein_coding	NP_001073905.1;XP_006723077.1;	-9.8
DHX37	DEAH-box helicase 37	protein_coding	NP_116045.2;XP_005253647.1;XP	-9.9
CLCN7	chloride voltage-gated channel 7	protein_coding	NP_001107803.1;NP_001278.1;XP	-9.9
CLPP	caseinolytic mitochondrial matrix peptidase protec	protein_coding	NP_006003.1	-9.9
BRF1	BRF1 RNA polymerase III transcription initiation	protein_coding	NP_001229715.1;NP_001229716.1;	-9.9
ADRM1	adhesion regulating molecule 1	protein_coding	NP_001268366.1;NP_001268367.1;	-9.9
PPP1CA	protein phosphatase 1 catalytic subunit alpha	protein_coding	NP_001008709.1;NP_002699.1;NP	-9.9
FBXO27	F-box protein 27	protein_coding	NP_849142.1;XP_016881779.1	-9.9
HTR1D	5-hydroxytryptamine receptor 1D	protein_coding	NP_000855.1	-9.9
RECQL4	RecQ like helicase 4	protein_coding	NP_004251.4;XP_011515686.1;XP_	-9.9
GPS1	G protein pathway suppressor 1	protein_coding	NP_001308018.1;NP_001308019.1;	-9.9
PPP1R13L	protein phosphatase 1 regulatory subunit 13 like	protein_coding	NP_001135974.1;NP_006654.2;XP_	-9.9
PLOD3	procollagen-lysine,2-oxoglutarate 5-dioxygenase 3	protein_coding	NP_001075.1	-9.9
CLPTM1	CLPTM1 regulator of GABA type A receptor forv	protein_coding	NP_001269104.1;NP_001269105.1;	-10.0
NECAB3	N-terminal EF-hand calcium binding protein 3	protein_coding	NP_112508.3;NP_112509.3;XP_00	-10.0
HSF1	heat shock transcription factor 1	protein_coding	NP_005517.1;XP_005272372.1;XP_	-10.0
R3HDM4	R3H domain containing 4	protein_coding	NP_620129.2;XP_011526718.1;XP_	-10.0
FBXL18	F-box and leucine rich repeat protein 18	protein_coding	NP_001308142.1;NP_001350370.1;	-10.0
MLST8	MTOR associated protein, LST8 homolog	protein_coding	NP_001186102.1;NP_001186103.1;	-10.0
UNC93B1	unc-93 homolog B1, TLR signaling regulator	protein_coding	NP_112192.2;XP_011543592.1;XP_	-10.0
DDX54	DEAD-box helicase 54	protein_coding	NP_001104792.1;NP_076977.3	-10.0
SLC9A7P1	solute carrier family 9 member 7 pseudogene 1	ranscribed_pseudogen	.	-10.0
ELK1	ETS transcription factor ELK1	protein_coding	NP_001107595.1;NP_001244097.1;	-10.0
ARHGEF1	Rho guanine nucleotide exchange factor 1	protein_coding	NP_004697.2;NP_945328.1;NP_94	-10.0
PPP1R9B	protein phosphatase 1 regulatory subunit 9B	protein_coding	NP_115984.3	-10.0
LYPLA2	lysophospholipase 2	protein_coding	NP_009191.1;XP_005245785.1;XP_	-10.1
RUVBL2	RuvB like AAA ATPase 2	protein_coding	NP_001308119.1;NP_001308120.1;	-10.1
GFER	growth factor, augmenter of liver regeneration	protein_coding	NP_005253.3	-10.1

NUMBL	NUMB like endocytic adaptor protein	protein_coding	NP_001276908.1;NP_001276909.1;	-10.1
ACD	ACD shelterin complex subunit and telomerase rec	protein_coding	NP_001075955.2;NP_075065.3;XP_	-10.1
BAG6	BAG cochaperone 6	protein_coding	NP_001092004.1;NP_001186626.1;	-10.1
PGAM5	PGAM family member 5, mitochondrial serine/thr	protein_coding	NP_001164014.1;NP_001164015.1;	-10.1
CTNNBIP1	catenin beta interacting protein 1	protein_coding	NP_001012329.1;NP_064633.1;XP_	-10.1
CASZ1	castor zinc finger 1	protein_coding	NP_001073312.1;NP_060236.3;XP_	-10.1
MAF1	MAF1 homolog, negative regulator of RNA polyn	protein_coding	NP_115648.2	-10.1
ALDH3B1	aldehyde dehydrogenase 3 family member B1	protein_coding	NP_000685.1;NP_001025181.1;NP_	-10.1
CNP	2',3'-cyclic nucleotide 3' phosphodiesterase	protein_coding	NP_001317145.1;NP_149124.3;XP_	-10.2
VASH1	vasohibin 1	protein_coding	NP_055724.1;XP_016876576.1;XP_	-10.2
MAPT	microtubule associated protein tau	protein_coding	NP_001116538.2;NP_001116539.1;	-10.2
RHOB	ras homolog family member B	protein_coding	NP_004031.1	-10.2
CLN3	CLN3 lysosomal/endosomal transmembrane prote	protein_coding	NP_000077.1;NP_001035897.1;NP_	-10.2
BAIAP2	BAR/IMD domain containing adaptor protein 2	protein_coding	NP_001138360.1;NP_006331.1;NP_	-10.2
FADS3	fatty acid desaturase 3	protein_coding	NP_068373.1;XP_011543325.1;XP_	-10.2
COPE	COPI coat complex subunit epsilon	protein_coding	NP_001317398.1;NP_009194.2;NP_	-10.2
SLC2A6	solute carrier family 2 member 6	protein_coding	NP_001138571.1;NP_060055.2;XP_	-10.2
PIMREG	PICALM interacting mitotic regulator	protein_coding	NP_001182157.1;NP_061886.2;XP_	-10.3
GSDMD	gasdermin D	protein_coding	NP_001159709.1;NP_079012.3;XP_	-10.3
PRKAR1B	protein kinase cAMP-dependent type I regulatory	protein_coding	NP_001158230.1;NP_001158231.1;	-10.3
SERTAD3	SERTA domain containing 3	protein_coding	NP_037500.2;NP_976219.1;XP_00	-10.3
ACTG2	actin gamma 2, smooth muscle	protein_coding	NP_001186822.1;NP_001606.1	-10.3
SEZ6L2	seizure related 6 homolog like 2	protein_coding	NP_001107571.1;NP_001107572.1;	-10.3
RARG	retinoic acid receptor gamma	protein_coding	NP_000957.1;NP_001036193.1;NP_	-10.3
MMP24OS	MMP24 opposite strand	protein_coding	NP_001341932.1;NP_001341933.1	-10.3
CAPN1	calpain 1	protein_coding	NP_001185797.1;NP_001185798.1;	-10.3
CBX8	chromobox 8	protein_coding	NP_065700.1	-10.3
SLC19A1	solute carrier family 19 member 1	protein_coding	NP_001192135.1;NP_001192136.1;	-10.3
GRN	granulin precursor	protein_coding	NP_002078.1;XP_005257310.1;XP_	-10.3
LLGL1	LLGL scribble cell polarity complex component 1	protein_coding	NP_004131.4;XP_011522151.1;XP_	-10.4
DGKQ	diacylglycerol kinase theta	protein_coding	NP_001338.2;XP_011511713.1;XP_	-10.4
HSPA6	heat shock protein family A (Hsp70) member 6	protein_coding	NP_002146.2	-10.4
WDR81	WD repeat domain 81	protein_coding	NP_001157145.1;NP_001157281.1;	-10.4
CYB5R3	cytochrome b5 reductase 3	protein_coding	NP_000389.1;NP_001123291.1;NP_	-10.4
DUSP27	dual specificity phosphatase 27, atypical	protein_coding	NP_001073895.1;XP_011508448.1	-10.4
BIN1	bridging integrator 1	protein_coding	NP_001307561.1;NP_001307562.1;	-10.4
FAM3A	FAM3 metabolism regulating signaling molecule A	protein_coding	NP_001164603.1;NP_001164604.1;	-10.4
YIPF2	Yip1 domain family member 2	protein_coding	NP_001308368.1;NP_001308369.1;	-10.4
B4GAT1	beta-1,4-glucuronyltransferase 1	protein_coding	NP_006867.1	-10.4
ZNHIT2	zinc finger HIT-type containing 2	protein_coding	NP_055020.1	-10.4
TOR2A	torsin family 2 member A	protein_coding	NP_001078816.2;NP_001127902.1;	-10.4
PLEKHM2	pleckstrin homology and RUN domain containing	protein_coding	NP_055979.2;XP_005245847.1;XP_	-10.4
HOXC10	homeobox C10	protein_coding	NP_059105.2	-10.4
SHKBP1	SH3KBP1 binding protein 1	protein_coding	NP_612401.2;XP_006723537.1;XP_	-10.4
ZNF48	zinc finger protein 48	protein_coding	NP_001201835.1;NP_001201836.1;	-10.4
MDK	midkine	protein_coding	NP_001012333.1;NP_001012334.1;	-10.4
MYPOP	Myb related transcription factor, partner of profilin	protein_coding	NP_001012661.1;XP_016882231.1;	-10.4
VARS1	valyl-tRNA synthetase 1	protein_coding	NP_006286.1;XP_005249419.1;XP_	-10.5
MPG	N-methylpurine DNA glycosylase	protein_coding	NP_001015052.1;NP_001015054.1;	-10.5
FNDC4	fibronectin type III domain containing 4	protein_coding	NP_073734.1;XP_005264556.1	-10.5
PIN1	peptidylprolyl cis/trans isomerase, NIMA-interacti	protein_coding	NP_006212.1;XP_011526370.1	-10.5
SYNGR3	synaptogyrin 3	protein_coding	NP_004200.2	-10.5
SMO	smoothened, frizzled class receptor	protein_coding	NP_005622.1;XP_024302659.1	-10.5
VEGFB	vascular endothelial growth factor B	protein_coding	NP_001230662.1;NP_003368.1	-10.5
ECSIT	ECSIT signaling integrator	protein_coding	NP_001135936.1;NP_001135937.1;	-10.5
MRPL38	mitochondrial ribosomal protein L38	protein_coding	NP_115867.2	-10.5
NR1H2	nuclear receptor subfamily 1 group H member 2	protein_coding	NP_001243576.2;NP_009052.4	-10.6
CHTF18	chromosome transmission fidelity factor 18	protein_coding	NP_071375.1;XP_005255527.1;XP_	-10.6
ARF5	ADP ribosylation factor 5	protein_coding	NP_001653.1	-10.6
ARSI	arylsulfatase family member I	protein_coding	NP_001012301.1	-10.6
RHOBTB2	Rho related BTB domain containing 2	protein_coding	NP_001153508.1;NP_001153509.1;	-10.6
TBKBP1	TBK1 binding protein 1	protein_coding	NP_055541.1;XP_005257916.1;XP_	-10.6
ZNF574	zinc finger protein 574	protein_coding	NP_001317448.1;NP_073589.4;XP_	-10.6
DEPP1	DEPP1 autophagy regulator	protein_coding	NP_008952.1	-10.6
PTPA	protein phosphatase 2 phosphatase activator	protein_coding	NP_001180326.1;NP_001258761.1;	-10.6
SERTAD1	SERTA domain containing 1	protein_coding	NP_037508.2	-10.6
TMEM161A	transmembrane protein 161A	protein_coding	NP_001243695.1;NP_060284.1;XP_	-10.6
HOMER3	homer scaffold protein 3	protein_coding	NP_001139193.1;NP_001139194.1;	-10.6
PGLS	6-phosphogluconolactonase	protein_coding	NP_036220.1;XP_011526199.1	-10.6
PACSIN3	protein kinase C and casein kinase substrate in neu	protein_coding	NP_001171903.1;NP_001171904.1;	-10.6
GRAMD1A	GRAM domain containing 1A	protein_coding	NP_001129671.1;NP_001306963.1;	-10.7

THAP7	THAP domain containing 7	protein_coding	NP_001008695.1;NP_085050.2	-10.7
UBE2M	ubiquitin conjugating enzyme E2 M	protein_coding	NP_003960.1	-10.7
LY6E	lymphocyte antigen 6 family member E	protein_coding	NP_001120685.1;NP_002337.1	-10.7
GASK1B	golgi associated kinase 1B	protein_coding	NP_001026870.2;NP_001121896.1;	-10.7
MARCKSL1	MARCKS like 1	protein_coding	NP_075385.1	-10.7
ZNF598	zinc finger protein 598, E3 ubiquitin ligase	protein_coding	NP_835461.2	-10.7
KRT8	keratin 8	protein_coding	NP_001243211.1;NP_001243222.1;	-10.7
ASPSCR1	ASPSCR1 tether for SLC2A4, UBX domain conta	protein_coding	NP_001238817.1;NP_001317457.1;	-10.7
SPATC1L	spermatogenesis and centriole associated 1 like	protein_coding	NP_001136326.1;NP_115637.3;XP_	-10.7
LENG9	leukocyte receptor cluster member 9	protein_coding	NP_001288711.1	-10.7
RAB40C	RAB40C, member RAS oncogene family	protein_coding	NP_001166134.1;NP_001166135.1;	-10.7
KMT5C	lysine methyltransferase 5C	protein_coding	NP_116090.2;XP_005259395.1;XP_	-10.7
DISP2	dispatched RND transporter family member 2	protein_coding	NP_277045.1;XP_011520427.1;XP_	-10.7
NAB2	NGFI-A binding protein 2	protein_coding	NP_001317234.1;NP_005958.1	-10.8
SMAD6	SMAD family member 6	protein_coding	NP_005576.3;XP_011519863.1	-10.8
CAPN15	calpain 15	protein_coding	NP_005623.1;XP_011520922.1;XP_	-10.8
HSPA1B	heat shock protein family A (Hsp70) member 1B	protein_coding	NP_005337.2	-10.8
ATN1	atrophin 1	protein_coding	NP_001007027.1;NP_001931.2	-10.8
KRT80	keratin 80	protein_coding	NP_001074961.1;NP_872313.2;XP_	-10.8
USP5	ubiquitin specific peptidase 5	protein_coding	NP_001092006.1;NP_001369517.1;	-10.8
SUN2	Sad1 and UNC84 domain containing 2	protein_coding	NP_001186508.1;NP_001186509.1;	-10.8
EMC10	ER membrane protein complex subunit 10	protein_coding	NP_778233.4;NP_996261.1;XP_00	-10.9
ZNF428	zinc finger protein 428	protein_coding	NP_872304.2	-10.9
CD151	CD151 molecule (Raph blood group)	protein_coding	NP_001034579.1;NP_004348.2;NP_	-10.9
SGTA	small glutamine rich tetratricopeptide repeat conta	protein_coding	NP_003012.1;XP_011526480.1	-10.9
PTGES2	prostaglandin E synthase 2	protein_coding	NP_001243264.1;NP_079348.1;NP_	-10.9
ADA	adenosine deaminase	protein_coding	NP_000013.2;NP_001308979.1;NP_	-10.9
ABHD17A	abhydrolase domain containing 17A, depalmitoyla	protein_coding	NP_001123583.1;NP_112490.3;XP_	-10.9
CORO1B	coronin 1B	protein_coding	NP_001018080.1;NP_065174.1	-10.9
TK1	thymidine kinase 1	protein_coding	NP_001333592.1;NP_001350777.1;	-10.9
H2AC4	H2A clustered histone 4	protein_coding	NP_003504.2	-11.0
NCAPH2	non-SMC condensin II complex subunit H2	protein_coding	NP_001171940.1;NP_055366.3;NP_	-11.0
THAP8	THAP domain containing 8	protein_coding	NP_001318031.1;NP_001318032.1;	-11.0
BOLA1	bolA family member 1	protein_coding	NP_001307954.1;NP_001307955.1;	-11.0
ABI3	ABI family member 3	protein_coding	NP_001128658.1;NP_057512.2;XP_	-11.0
IGFLR1	IGF like family receptor 1	protein_coding	NP_001332932.1;NP_001332933.1;	-11.1
PALM3	paralemmin 3	protein_coding	NP_001138500.2;NP_001354256.1;	-11.1
ILVBL	ilvB acetolactate synthase like	protein_coding	NP_006835.2;XP_005259774.1;XP_	-11.1
SIVA1	SIVA1 apoptosis inducing factor	protein_coding	NP_006418.2;NP_068355.1;XP_01	-11.1
HSPA2	heat shock protein family A (Hsp70) member 2	protein_coding	NP_068814.2	-11.1
TSEN34	tRNA splicing endonuclease subunit 34	protein_coding	NP_001070914.1;NP_001269261.1;	-11.1
MRPS12	mitochondrial ribosomal protein S12	protein_coding	NP_066930.1;NP_203526.1;NP_20	-11.1
NOC2L	NOC2 like nucleolar associated transcriptional rep	protein_coding	NP_056473.3	-11.1
TMEM115	transmembrane protein 115	protein_coding	NP_008955.1	-11.1
FLNA	filamin A	protein_coding	NP_001104026.1;NP_001447.2	-11.2
CLTB	clathrin light chain B	protein_coding	NP_001351055.1;NP_001351056.1;	-11.2
HPS6	HPS6 biogenesis of lysosomal organelles complex	protein_coding	NP_079023.2	-11.2
NDUFS7	NADH:ubiquinone oxidoreductase core subunit S	protein_coding	NP_001350531.1;NP_077718.3;XP_	-11.2
POLD1	DNA polymerase delta 1, catalytic subunit	protein_coding	NP_001243778.1;NP_001295561.1;	-11.2
RIN1	Ras and Rab interactor 1	protein_coding	NP_001350488.1;NP_001350489.1;	-11.2
SF3A2	splicing factor 3a subunit 2	protein_coding	NP_009096.2	-11.3
PIAS4	protein inhibitor of activated STAT 4	protein_coding	NP_056981.2;XP_011526362.1;XP_	-11.3
CACTIN	cactin, spliceosome C complex subunit	protein_coding	NP_001074012.1;NP_067054.1;XP_	-11.3
FZR1	fizzy and cell division cycle 20 related 1	protein_coding	NP_001129669.1;NP_001129670.1;	-11.3
GIT1	GIT ArfGAP 1	protein_coding	NP_001078923.1;NP_054749.2;XP_	-11.3
SLC7A5	solute carrier family 7 member 5	protein_coding	NP_003477.4;XP_006721349.2;XP_	-11.3
SOX12	SRY-box transcription factor 12	protein_coding	NP_008874.2	-11.3
NUDT22	nudix hydrolase 22	protein_coding	NP_001122084.1;NP_001122085.1;	-11.3
COL6A1	collagen type VI alpha 1 chain	protein_coding	NP_001839.2	-11.3
CAND2	cullin associated and neddylation dissociated 2 (pu	protein_coding	NP_001155971.1;NP_036430.1;XP_	-11.3
SIPA1	signal-induced proliferation-associated 1	protein_coding	NP_006738.3;NP_694985.29;XP_0	-11.3
TRMT2A	tRNA methyltransferase 2 homolog A	protein_coding	NP_001244923.1;NP_001317968.1;	-11.3
TRIP6	thyroid hormone receptor interactor 6	protein_coding	NP_003293.2	-11.4
FASN	fatty acid synthase	protein_coding	NP_004095.4;XP_011521840.1	-11.4
CFAP410	cilia and flagella associated protein 410	protein_coding	NP_001258369.1;NP_001258370.1;	-11.4
MIEF2	mitochondrial elongation factor 2	protein_coding	NP_001138372.1;NP_631901.2;NP_	-11.4
PHLDB3	pleckstrin homology like domain family B membe	protein_coding	NP_942147.3;XP_005259226.1;XP_	-11.4
ALKBH4	alkB homolog 4, lysine demethylase	protein_coding	NP_060091.1;XP_005250521.1;XP_	-11.4
ZNF362	zinc finger protein 362	protein_coding	NP_001357141.1;NP_689706.2;XP_	-11.4
PKN3	protein kinase N3	protein_coding	NP_001304855.1;NP_037487.2;XP_	-11.4
SNORD3B-1	small nucleolar RNA, C/D box 3B-1	snoRNA	.	-11.5

MAP7D1	MAP7 domain containing 1	protein_coding	NP_001273294.1;NP_001273295.1;	-11.5
KLC2	kinesin light chain 2	protein_coding	NP_001128246.1;NP_001128247.1;	-11.5
COL1A2	collagen type I alpha 2 chain	protein_coding	NP_000080.2	-11.5
DMPK	DM1 protein kinase	protein_coding	NP_001075029.1;NP_001075031.1;	-11.6
TUBB4B	tubulin beta 4B class IVb	protein_coding	NP_006079.1	-11.6
LIMK1	LIM domain kinase 1	protein_coding	NP_001191355.1;NP_002305.1	-11.6
LRRC15	leucine rich repeat containing 15	protein_coding	NP_001128529.2;NP_570843.2	-11.6
NCOR2	nuclear receptor corepressor 2	protein_coding	NP_001070729.2;NP_001193583.1;	-11.6
PIK3R2	phosphoinositide-3-kinase regulatory subunit 2	protein_coding	NP_005018.2	-11.6
CTDSP1	CTD small phosphatase 1	protein_coding	NP_001193807.1;NP_067021.1;NP_	-11.6
ST3GAL2	ST3 beta-galactoside alpha-2,3-sialyltransferase 2	protein_coding	NP_008858.1	-11.7
ARSA	arylsulfatase A	protein_coding	NP_000478.3;NP_001078894.2;NP_	-11.7
GPR162	G protein-coupled receptor 162	protein_coding	NP_055264.1;NP_062832.1	-11.7
BCORL1	BCL6 corepressor like 1	protein_coding	NP_001171701.1;NP_001366379.1;	-11.7
H4C12	H4 clustered histone 12	protein_coding	NP_003532.1	-11.7
PCOLCE	procollagen C-endopeptidase enhancer	protein_coding	NP_002584.2;XP_024302553.1	-11.7
LOC101927752	uncharacterized LOC101927752	lncRNA	.	-11.7
PRAG1	PEAK1 related, kinase-activating pseudokinase 1	protein_coding	NP_001074295.2;NP_001356688.1	-11.7
TLE5	TLE family member 5, transcriptional modulator	protein_coding	NP_001121.2;NP_945320.1;NP_94	-11.8
ST6GALNAC4	ST6 N-acetylgalactosaminide alpha-2,6-sialyltrans	protein_coding	NP_778204.1;NP_778205.1;XP_01	-11.8
HSPA1A	heat shock protein family A (Hsp70) member 1A	protein_coding	NP_005336.3	-11.8
PCBP4	poly(rC) binding protein 4	protein_coding	NP_001167571.1;NP_001350814.1;	-11.8
BMP8A	bone morphogenetic protein 8a	protein_coding	NP_861525.2;XP_006710679.1;XP_	-11.8
H4C11	H4 clustered histone 11	protein_coding	NP_068803.1	-11.8
CELSR2	cadherin EGF LAG seven-pass G-type receptor 2	protein_coding	NP_001399.1	-11.9
FOXP4	forkhead box P4	protein_coding	NP_001012426.1;NP_001012427.1;	-11.9
CDIPT	CDP-diacylglycerol--inositol 3-phosphatidyltransf	protein_coding	NP_001273514.1;NP_001273515.1;	-11.9
SDSL	serine dehydratase like	protein_coding	NP_001291922.1;NP_612441.1;XP_	-11.9
UNC119	unc-119 lipid binding chaperone	protein_coding	NP_001317095.1;NP_005139.1;NP_	-11.9
ROGDI	rogdi atypical leucine zipper	protein_coding	NP_078865.1;XP_006721010.1;XP_	-11.9
FSTL3	follistatin like 3	protein_coding	NP_005851.1	-11.9
UBALD1	UBA like domain containing 1	protein_coding	NP_001317396.1;NP_660296.1	-11.9
NLGN2	neuroligin 2	protein_coding	NP_065846.1;XP_005256801.1;XP_	-11.9
FLOT2	flotillin 2	protein_coding	NP_001317099.1;NP_004466.2;XP_	-11.9
SLC10A3	solute carrier family 10 member 3	protein_coding	NP_001135863.1;NP_001135864.1;	-11.9
NUDT14	nudix hydrolase 14	protein_coding	NP_001305309.1;NP_803877.2	-12.0
H2AC16	H2A clustered histone 16	protein_coding	NP_003502.1	-12.0
TMEM54	transmembrane protein 54	protein_coding	NP_001316651.1;NP_001316652.1;	-12.0
PLTP	phospholipid transfer protein	protein_coding	NP_001229849.1;NP_001229850.1;	-12.0
RAB43P1	RAB43 pseudogene 1	pseudogene	.	-12.0
LFNG	LFNG O-fucosylpeptide 3-beta-N-acetylglucosam	protein_coding	NP_001035257.1;NP_001035258.1;	-12.0
SSNA1	SS nuclear autoantigen 1	protein_coding	NP_003722.2	-12.0
JUNB	JunB proto-oncogene, AP-1 transcription factor su	protein_coding	NP_002220.1	-12.0
EMD	emerin	protein_coding	NP_000108.1;XP_024308117.1	-12.0
CUEDC2	CUE domain containing 2	protein_coding	NP_076945.2	-12.0
ZNF581	zinc finger protein 581	protein_coding	NP_057619.1;XP_006723303.1;XP_	-12.1
RPS2P46	ribosomal protein S2 pseudogene 46	pseudogene	.	-12.1
SLC25A39	solute carrier family 25 member 39	protein_coding	NP_001137252.1;NP_001308169.1;	-12.1
ARL4D	ADP ribosylation factor like GTPase 4D	protein_coding	NP_001652.2;XP_011523084.1	-12.1
MGRN1	mahogunin ring finger 1	protein_coding	NP_001135761.2;NP_001135762.1;	-12.1
DDR1	discoidin domain receptor tyrosine kinase 1	protein_coding	NP_001189450.1;NP_001189451.1;	-12.1
COL5A1	collagen type V alpha 1 chain	protein_coding	NP_000084.3;NP_001265003.1;XP_	-12.1
ZNF668	zinc finger protein 668	protein_coding	NP_001166139.1;NP_001166140.1;	-12.1
VASP	vasodilator stimulated phosphoprotein	protein_coding	NP_003361.1;XP_005259256.1;XP_	-12.1
DGKZ	diacylglycerol kinase zeta	protein_coding	NP_001099010.1;NP_001186195.1;	-12.2
RAB3D	RAB3D, member RAS oncogene family	protein_coding	NP_004274.1	-12.2
PKD1	polycystin 1, transient receptor potential channel i	protein_coding	NP_000287.4;NP_001009944.3;XP_	-12.2
TCF7L1	transcription factor 7 like 1	protein_coding	NP_112573.1;XP_006712172.1	-12.2
CC2D1A	coiled-coil and C2 domain containing 1A	protein_coding	NP_060191.3;XP_005260030.1;XP_	-12.2
TMEM184B	transmembrane protein 184B	protein_coding	NP_001182000.1;NP_001182001.1;	-12.2
PGAP6	post-glycosylphosphatidylinositol attachment to pi	protein_coding	NP_067082.2;XP_024306134.1	-12.2
CCDC86	coiled-coil domain containing 86	protein_coding	NP_077003.1	-12.2
FLYWCH1	FLYWCH-type zinc finger 1	protein_coding	NP_001294997.1;NP_065963.1;NP_	-12.2
RASD2	RASD family member 2	protein_coding	NP_001353654.1;NP_001363444.1;	-12.2
LTBP4	latent transforming growth factor beta binding pro	protein_coding	NP_001036009.1;NP_001036010.1;	-12.2
CRAT	carnitine O-acetyltransferase	protein_coding	NP_000746.3;NP_001244292.2;NP_	-12.3
ESRRA	estrogen related receptor alpha	protein_coding	NP_001269379.1;NP_001269380.1;	-12.3
GPX1P1	glutathione peroxidase pseudogene 1	pseudogene	.	-12.3
NDUFS8	NADH:ubiquinone oxidoreductase core subunit S8	protein_coding	NP_002487.1	-12.3
RITA1	RBPJ interacting and tubulin associated 1	protein_coding	NP_001273144.1;NP_116237.1;XP_	-12.3
SPINDOC	spindlin interactor and repressor of chromatin binc	protein_coding	NP_612480.1;XP_005273839.1;XP_	-12.3

MARCHF2	membrane associated ring-CH-type finger 2	protein_coding	NP_001005415.1;NP_001005416.1;	-12.3
ZNF580	zinc finger protein 580	protein_coding	NP_001156895.1;NP_057286.1;NP_	-12.3
JUND	JunD proto-oncogene, AP-1 transcription factor su	protein_coding	NP_001273897.1;NP_005345.3	-12.3
ORAI2	ORAI calcium release-activated calcium modulato	protein_coding	NP_001119812.1;NP_001258747.1;	-12.4
ADAM15	ADAM metallopeptidase domain 15	protein_coding	NP_001248393.1;NP_001248394.1;	-12.4
RHOT2	ras homolog family member T2	protein_coding	NP_001339204.1;NP_001339205.1;	-12.4
CCDC85C	coiled-coil domain containing 85C	protein_coding	NP_001138467.1;XP_011535007.1;	-12.4
MKNK2	MAPK interacting serine/threonine kinase 2	protein_coding	NP_060042.2;NP_951009.1;XP_02	-12.4
RFX1	regulatory factor X1	protein_coding	NP_002909.4;XP_011526467.1;XP_	-12.4
SALL2	spalt like transcription factor 2	protein_coding	NP_001278375.1;NP_001278376.1;	-12.4
SCO2	synthesis of cytochrome C oxidase 2	protein_coding	NP_001162580.1;NP_001162581.1;	-12.4
UBXN6	UBX domain protein 6	protein_coding	NP_001164562.1;NP_079517.1;XP_	-12.4
CDC34	cell division cycle 34, ubiqiutin conjugating enzyn	protein_coding	NP_004350.1;XP_005259747.1;XP_	-12.4
ZNF205	zinc finger protein 205	protein_coding	NP_001035893.1;NP_001265087.1;	-12.4
CHMP1A	charged multivesicular body protein 1A	protein_coding	NP_001076783.1;NP_002759.2;XP_	-12.5
SBNO2	strawberry notch homolog 2	protein_coding	NP_001093592.1;NP_055778.2;XP_	-12.5
VPS51	VPS51 subunit of GARP complex	protein_coding	NP_037397.2	-12.5
DDAH2	dimethylarginine dimethylaminohydrolase 2	protein_coding	NP_001289936.1;NP_001289937.1;	-12.5
MFSD12	major facilitator superfamily domain containing 1:	protein_coding	NP_001274458.1;NP_778148.2;XP_	-12.5
GYS1	glycogen synthase 1	protein_coding	NP_001155059.1;NP_002094.2	-12.5
FDX2	ferredoxin 2	protein_coding	NP_001026904.2	-12.5
COL3A1	collagen type III alpha 1 chain	protein_coding	NP_000081.2	-12.5
SLC46A1	solute carrier family 46 member 1	protein_coding	NP_001229295.1;NP_542400.2;XP_	-12.6
SOCS3	suppressor of cytokine signaling 3	protein_coding	NP_001365861.1;NP_001365862.1;	-12.6
UBE2SP1	ubiquitin conjugating enzyme E2 S pseudogene 1	pseudogene	.	-12.6
PTOV1	PTOV1 extended AT-hook containing adaptor pro	protein_coding	NP_001292034.1;NP_001292037.1;	-12.6
RNF126	ring finger protein 126	protein_coding	NP_001352947.1;NP_919442.1	-12.7
PPP1R12C	protein phosphatase 1 regulatory subunit 12C	protein_coding	NP_001258547.1;NP_060077.1;XP_	-12.7
SLC27A4	solute carrier family 27 member 4	protein_coding	NP_005085.2;XP_016869711.1;XP_	-12.7
HDAC11	histone deacetylase 11	protein_coding	NP_001129513.1;NP_001317565.1;	-12.7
VSIR	V-set immunoregulatory receptor	protein_coding	NP_071436.1	-12.7
PPP1R37	protein phosphatase 1 regulatory subunit 37	protein_coding	NP_061994.1;XP_006723222.1	-12.8
BCL9L	BCL9 like	protein_coding	NP_001365142.1;NP_001365143.1;	-12.8
H3C1	H3 clustered histone 1	protein_coding	NP_003520.1	-12.8
BAHD1	bromo adjacent homology domain containing 1	protein_coding	NP_001288061.1;NP_055767.3;XP_	-12.8
MIIP	migration and invasion inhibitory protein	protein_coding	NP_068752.2;XP_005263544.1;XP_	-12.8
NPRL3	NPR3 like, GATOR1 complex subunit	protein_coding	NP_001034565.1;NP_001070818.1;	-12.8
NOSIP	nitric oxide synthase interacting protein	protein_coding	NP_001257889.1;NP_001350578.1;	-12.8
SNORD3D	small nucleolar RNA, C/D box 3D	snoRNA	.	-12.8
PITPNM1	phosphatidylinositol transfer protein membrane as	protein_coding	NP_001124320.1;NP_004901.2;XP_	-12.8
PLK1	polo like kinase 1	protein_coding	NP_005021.2	-12.8
ADAT3	adenosine deaminase tRNA specific 3	protein_coding	NP_001316462.1;NP_612431.2	-12.8
COL6A2	collagen type VI alpha 2 chain	protein_coding	NP_001840.3;NP_478054.2;NP_47	-12.8
HSD3B7	hydroxy-delta-5-steroid dehydrogenase, 3 beta- an	protein_coding	NP_001136249.1;NP_001136250.1;	-12.8
POLRMT	RNA polymerase mitochondrial	protein_coding	NP_005026.3;XP_005259637.3	-12.8
RAI1	retinoic acid induced 1	protein_coding	NP_109590.3;XP_016879514.1;XP_	-12.9
ZNF512B	zinc finger protein 512B	protein_coding	NP_065764.1;XP_011527232.1;XP_	-12.9
ALKBH7	alkB homolog 7	protein_coding	NP_115682.1;XP_005259715.1;XP_	-12.9
CAMK2N2	calcium/calmodulin dependent protein kinase II in	protein_coding	NP_150284.1	-12.9
DCHS1	dachsous cadherin-related 1	protein_coding	NP_003728.1	-13.0
TGFB1	transforming growth factor beta 1	protein_coding	NP_000651.3;XP_011525544.1	-13.0
AGRN	agrin	protein_coding	NP_001292204.1;NP_001351656.1;	-13.0
DGCR2	DiGeorge syndrome critical region gene 2	protein_coding	NP_001167004.1;NP_001167005.1;	-13.0
HMG20B	high mobility group 20B	protein_coding	NP_006330.2;XP_016881633.1;XP_	-13.0
TRIM28	tripartite motif containing 28	protein_coding	NP_005753.1;XP_024307077.1	-13.0
GRK2	G protein-coupled receptor kinase 2	protein_coding	NP_001610.2;XP_011543075.1;XP_	-13.0
HDGFL2	HDGF like 2	protein_coding	NP_001001520.1;NP_001335098.1;	-13.1
REXO1	RNA exonuclease 1 homolog	protein_coding	NP_065746.3;XP_011526446.1;XP_	-13.1
FAM189B	family with sequence similarity 189 member B	protein_coding	NP_001254537.1;NP_006580.2;NP_	-13.1
ADAMTSL4	ADAMTS like 4	protein_coding	NP_001275536.1;NP_001275537.1;	-13.1
PLXNB2	plexin B2	protein_coding	NP_001363793.1;NP_001363794.1;	-13.1
FAAP100	FA core complex associated protein 100	protein_coding	NP_079437.5;XP_006722174.1;XP_	-13.1
SCAP	SREBF chaperone	protein_coding	NP_001306973.1;NP_036367.2;XP_	-13.1
THRA	thyroid hormone receptor alpha	protein_coding	NP_001177847.1;NP_001177848.1;	-13.2
LRP5	LDL receptor related protein 5	protein_coding	NP_001278831.1;NP_002326.2;XP_	-13.2
NOP53	NOP53 ribosome biogenesis factor	protein_coding	NP_056525.2	-13.2
CPLANE2	ciliogenesis and planar polarity effector 2	protein_coding	NP_112169.2;XP_011540428.1	-13.2
TWF2	twinfilin actin binding protein 2	protein_coding	NP_009215.1	-13.2
PAFAH1B3	platelet activating factor acetylhydrolase 1b cataly	protein_coding	NP_001139411.1;NP_001139412.1;	-13.2
FAM78A	family with sequence similarity 78 member A	protein_coding	NP_203745.2;XP_011516869.1;XP_	-13.2
DAGLA	diacylglycerol lipase alpha	protein_coding	NP_006124.1;XP_016873727.1;XP_	-13.2

SNPH	syntaphilin	protein_coding	NP_001305163.1;NP_055538.2;XP_	-13.2
TMEM250	transmembrane protein 250	protein_coding	NP_001243455.1;NP_690046.3;XP_	-13.2
FAM234A	family with sequence similarity 234 member A	protein_coding	NP_001271426.1;NP_114428.1;XP_	-13.3
METTL26	methyltransferase like 26	protein_coding	NP_001035250.1;NP_001035251.1;	-13.3
SLC2A8	solute carrier family 2 member 8	protein_coding	NP_001258640.1;NP_001258641.1;	-13.3
LZTS3	leucine zipper tumor suppressor family member 3	protein_coding	NP_001269462.1;NP_001352547.1;	-13.3
RIMBP3	RIMS binding protein 3	protein_coding	NP_056487.1	-13.3
BICRA	BRD4 interacting chromatin remodeling complex	protein_coding	NP_056526.3;XP_005258890.1;XP_	-13.3
MCRIP2	MAPK regulated corepressor interacting protein 2	protein_coding	NP_001318158.1;NP_001318159.1;	-13.3
PNPLA6	patatin like phospholipase domain containing 6	protein_coding	NP_001159583.1;NP_001159584.1;	-13.3
TONSL	tonsoku like, DNA repair protein	protein_coding	NP_038460.4;XP_011515350.1;XP_	-13.3
ATAD3A	ATPase family AAA domain containing 3A	protein_coding	NP_001164006.1;NP_001164007.1;	-13.4
TGFB1I1	transforming growth factor beta 1 induced transcri	protein_coding	NP_001035919.1;NP_001158191.1;	-13.4
FASTK	Fas activated serine/threonine kinase	protein_coding	NP_001245390.1;NP_006703.1;NP_	-13.4
C1QTNF6	C1q and TNF related 6	protein_coding	NP_001352807.1;NP_114116.3;NP_	-13.4
SELENON	selenoprotein N	protein_coding	NP_065184.2;NP_996809.1	-13.4
NINJ1	ninjurin 1	protein_coding	NP_004139.2;XP_011517018.1	-13.4
PFKL	phosphofructokinase, liver type	protein_coding	NP_001002021.2;NP_002617.3;XP_	-13.5
OTX1	orthodenticle homeobox 1	protein_coding	NP_001186699.1;NP_055377.1	-13.5
HEXIM2	HEXIM P-TEFb complex subunit 2	protein_coding	NP_001290365.1;NP_001290366.1;	-13.5
CDIP1	cell death inducing p53 target 1	protein_coding	NP_001185983.1;NP_001185984.1;	-13.5
SLC25A1	solute carrier family 25 member 1	protein_coding	NP_001243463.1;NP_001274316.1;	-13.5
ZNF213	zinc finger protein 213	protein_coding	NP_001128127.1;NP_004211.1;XP_	-13.5
SHARPIN	SHANK associated RH domain interactor	protein_coding	NP_112236.3;XP_016869376.1;XP_	-13.5
NPEPL1	aminopeptidase like 1	protein_coding	NP_001191801.1;NP_001191802.1;	-13.5
ATG2A	autophagy related 2A	protein_coding	NP_001354900.1;NP_001354901.1;	-13.6
TEAD3	TEA domain transcription factor 3	protein_coding	NP_003205.2	-13.6
SLC27A1	solute carrier family 27 member 1	protein_coding	NP_940982.1;XP_011526302.1;XP_	-13.6
ADGRL1	adhesion G protein-coupled receptor L1	protein_coding	NP_001008701.1;NP_055736.2;XP_	-13.6
MLLT1	MLLT1 super elongation complex subunit	protein_coding	NP_005925.2;XP_011526323.1;XP_	-13.6
SNORD3C	small nucleolar RNA, C/D box 3C	snoRNA	.	-13.6
ATP13A2	ATPase cation transporting 13A2	protein_coding	NP_001135445.1;NP_001135446.1;	-13.6
NDUFB7	NADH:ubiquinone oxidoreductase subunit B7	protein_coding	NP_004137.2;XP_011526341.1	-13.6
STK11	serine/threonine kinase 11	protein_coding	NP_000446.1	-13.6
HID1	HID1 domain containing	protein_coding	NP_085133.1;XP_005257283.1	-13.7
H3C8	H3 clustered histone 8	protein_coding	NP_003525.1	-13.7
DNLZ	DNL-type zinc finger	protein_coding	NP_001074318.1	-13.7
PLIN3	perilipin 3	protein_coding	NP_001157661.1;NP_001157666.1;	-13.7
AP2A1	adaptor related protein complex 2 subunit alpha 1	protein_coding	NP_055018.2;NP_570603.2;XP_01	-13.8
RPL13P12	ribosomal protein L13 pseudogene 12	pseudogene	.	-13.8
CCDC24	coiled-coil domain containing 24	protein_coding	NP_001336056.1;NP_001336057.1;	-13.8
REEP4	receptor accessory protein 4	protein_coding	NP_001303893.1;NP_001303894.1;	-13.8
DAB2IP	DAB2 interacting protein	protein_coding	NP_115941.2;NP_619723.1;XP_00	-13.8
REPIN1	replication initiator 1	protein_coding	NP_001093165.1;NP_001093166.1;	-13.8
TMEM141	transmembrane protein 141	protein_coding	NP_116317.1	-13.8
HCFC1R1	host cell factor C1 regulator 1	protein_coding	NP_001002017.1;NP_001002018.1;	-13.8
SYNPO	synaptopodin	protein_coding	NP_001103444.1;NP_001159680.1;	-13.8
SPSB3	splA/ryanodine receptor domain and SOCS box cc	protein_coding	NP_001311010.1;NP_543137.2;XP_	-13.9
IRAK1	interleukin 1 receptor associated kinase 1	protein_coding	NP_001020413.1;NP_001020414.1;	-13.9
GAL3ST4	galactose-3-O-sulfotransferase 4	protein_coding	NP_078913.3	-13.9
OBSL1	obscurin like cytoskeletal adaptor 1	protein_coding	NP_001166879.1;NP_001166902.1;	-13.9
CTSD	cathepsin D	protein_coding	NP_001900.1	-13.9
MICALL1	MICAL like 1	protein_coding	NP_203744.1;XP_005261848.1;XP_	-13.9
SLC43A2	solute carrier family 43 member 2	protein_coding	NP_001271427.1;NP_001271428.1;	-13.9
SPHK2	sphingosine kinase 2	protein_coding	NP_001191087.1;NP_001191088.1;	-13.9
PPP6R1	protein phosphatase 6 regulatory subunit 1	protein_coding	NP_055746.3	-14.0
EXOC3-AS1	EXOC3 antisense RNA 1	lncRNA	.	-14.0
NAA80	N-alpha-acetyltransferase 80, NatH catalytic subui	protein_coding	NP_001186945.1;NP_001186947.1;	-14.0
NDRG4	NDRG family member 4	protein_coding	NP_001123959.1;NP_001229762.1;	-14.1
ARMC5	armadillo repeat containing 5	protein_coding	NP_001098717.1;NP_001275696.1;	-14.1
INPP5J	inositol polyphosphate-5-phosphatase J	protein_coding	NP_001002837.1;NP_001271214.1;	-14.1
C1orf159	chromosome 1 open reading frame 159	protein_coding	NP_001317235.1;NP_001350454.1;	-14.1
YIF1B	Yip1 interacting factor homolog B, membrane traf	protein_coding	NP_001034760.1;NP_001034761.1;	-14.2
TMEM129	transmembrane protein 129, E3 ubiquitin ligase	protein_coding	NP_001120738.1;NP_612394.1;XP_	-14.2
SEPTIN9	septin 9	protein_coding	NP_001106963.1;NP_001106964.1;	-14.2
CHPF	chondroitin polymerizing factor	protein_coding	NP_001182660.1;NP_078812.3;XP_	-14.2
RNA18SN3	RNA, 18S ribosomal N3	rRNA	.	-14.2
LOC101060179	uncharacterized LOC101060179	protein_coding	XP_003959981.1	-14.2
UBE2S	ubiquitin conjugating enzyme E2 S	protein_coding	NP_055316.2;XP_011525054.1	-14.2
FAM102A	family with sequence similarity 102 member A	protein_coding	NP_001030331.1;NP_976050.1	-14.3
RABL6	RAB, member RAS oncogene family like 6	protein_coding	NP_001167459.1;NP_001167460.1;	-14.3

KCTD17	potassium channel tetramerization domain contain	protein_coding	NP_001269613.1;NP_001269614.1;	-14.3
ITPKA	inositol-trisphosphate 3-kinase A	protein_coding	NP_002211.1;XP_011519824.1	-14.3
FURIN	furin, paired basic amino acid cleaving enzyme	protein_coding	NP_001276752.1;NP_001276753.1;	-14.3
TSR3	TSR3 ribosome maturation factor	protein_coding	NP_001001410.1	-14.3
MED16	mediator complex subunit 16	protein_coding	NP_005472.2;XP_016881609.1;XP_	-14.3
MRPL4	mitochondrial ribosomal protein L4	protein_coding	NP_057040.2;NP_666499.1;NP_66(	-14.3
CLSTN2	calsyntenin 2	protein_coding	NP_071414.2;XP_016862511.1	-14.4
LRCH4	leucine rich repeats and calponin homology domai	protein_coding	NP_001276863.1;NP_002310.2	-14.4
CBX4	chromobox 4	protein_coding	NP_003646.2;XP_011523701.1	-14.4
TUBB2B	tubulin beta 2B class IIb	protein_coding	NP_821080.1	-14.4
EPS8L2	EPS8 like 2	protein_coding	NP_073609.2;XP_016873620.1;XP_	-14.4
MFSD10	major facilitator superfamily domain containing 1(	protein_coding	NP_001111.3;NP_001139541.1;NP_	-14.4
PDLIM2	PDZ and LIM domain 2	protein_coding	NP_001355049.1;NP_067643.3;NP_	-14.5
GRK6	G protein-coupled receptor kinase 6	protein_coding	NP_001004105.1;NP_001004106.1;	-14.5
SH3BP1	SH3 domain binding protein 1	protein_coding	NP_001336984.1;NP_061830.3	-14.5
PAXX	PAXX non-homologous end joining factor	protein_coding	NP_001316607.1;NP_899064.1	-14.5
MYL9	myosin light chain 9	protein_coding	NP_006088.2;NP_852667.1	-14.5
TNFAIP8L1	TNF alpha induced protein 8 like 1	protein_coding	NP_001161414.1;NP_689575.2;XP_	-14.5
APRT	adenine phosphoribosyltransferase	protein_coding	NP_000476.1;NP_001025189.1	-14.5
METRNL	meteorin like, glial cell differentiation regulator	protein_coding	NP_001004431.1;NP_001350782.1;	-14.5
H3C7	H3 clustered histone 7	protein_coding	NP_066298.1	-14.5
CLUH	clustered mitochondria homolog	protein_coding	NP_001353590.1;NP_001353591.1;	-14.5
RAD23A	RAD23 homolog A, nucleotide excision repair prc	protein_coding	NP_001257291.1;NP_001257292.1;	-14.5
MPST	mercaptopyruvate sulfurtransferase	protein_coding	NP_001013454.1;NP_001123989.1;	-14.6
PRRC2A	proline rich coiled-coil 2A	protein_coding	NP_004629.3;NP_542417.2;XP_01(	-14.6
LMNB2	lamin B2	protein_coding	NP_116126.3	-14.6
LOC112694756	uncharaterized LOC112694756	protein_coding	NP_001352233.1;NP_001352234.1;	-14.6
PIM3	Pim-3 proto-oncogene, serine/threonine kinase	protein_coding	NP_001001852.2	-14.7
ARL2	ADP ribosylation factor like GTPase 2	protein_coding	NP_001186674.1;NP_001658.2	-14.7
CLEC11A	C-type lectin domain containing 11A	protein_coding	NP_002966.1	-14.7
NFIC	nuclear factor I C	protein_coding	NP_001231931.1;NP_001231933.1;	-14.7
REX1BD	required for excision 1-B domain containing	protein_coding	NP_001093888.1;NP_001093889.1;	-14.7
FBXW5	F-box and WD repeat domain containing 5	protein_coding	NP_061871.1;XP_005266146.2;XP_	-14.8
PKN1	protein kinase N1	protein_coding	NP_002732.3;NP_998725.1	-14.8
FAM89B	family with sequence similarity 89 member B	protein_coding	NP_001092254.1;NP_001092255.1;	-14.8
INTS1	integrator complex subunit 1	protein_coding	NP_001073922.2;XP_011513562.1;	-14.9
PYCR3	pyrroline-5-carboxylate reductase 3	protein_coding	NP_001316795.2;NP_075566.3	-14.9
ZC3H3	zinc finger CCCH-type containing 3	protein_coding	NP_055932.2;XP_006716599.2;XP_	-14.9
BCR	BCR activator of RhoGEF and GTPase	protein_coding	NP_004318.3;NP_067585.2	-14.9
STUB1	STIP1 homology and U-box containing protein 1	protein_coding	NP_001280126.1;NP_005852.2	-15.0
CXXC5	CXXC finger protein 5	protein_coding	NP_001304128.1;NP_001304129.1;	-15.0
CCDC22	coiled-coil domain containing 22	protein_coding	NP_054727.1;XP_005272656.1	-15.0
BOP1	BOP1 ribosomal biogenesis factor	protein_coding	NP_056016.1	-15.1
TSSC4	tumor suppressing subtransferable candidate 4	protein_coding	NP_001284587.1;NP_001284588.1;	-15.1
LRFN3	leucine rich repeat and fibronectin type III domain	protein_coding	NP_078785.1	-15.1
TRMT61A	tRNA methyltransferase 61A	protein_coding	NP_689520.2	-15.1
OSR2	odd-skipped related transciption factor 2	protein_coding	NP_001135934.1;NP_001273770.1;	-15.1
H2AX	H2A.X variant histone	protein_coding	NP_002096.1	-15.1
PLD3	phospholipase D family member 3	protein_coding	NP_001026866.1;NP_001278240.1;	-15.1
RRAS	RAS related	protein_coding	NP_006261.1	-15.1
WFS1	wolframin ER transmembrane glycoprotein	protein_coding	NP_001139325.1;NP_005996.2;XP_	-15.2
MAZ	MYC associated zinc finger protein	protein_coding	NP_001036004.1;NP_001263204.1;	-15.2
TOMM40	translocase of outer mitochondrial membrane 40	protein_coding	NP_001122388.1;NP_001122389.1;	-15.2
NECTIN2	nectin cell adhesion molecule 2	protein_coding	NP_001036189.1;NP_002847.1	-15.3
NELFB	negative elongation factor complex member B	protein_coding	NP_056271.3	-15.3
CCDC106	coiled-coil domain containing 106	protein_coding	NP_001357396.1;NP_001357397.1;	-15.4
MAGEA3	MAGE family member A3	protein_coding	NP_005353.1;XP_005274733.1;XP_	-15.4
DBP	D-box binding PAR bZIP transcription factor	protein_coding	NP_001343.2;XP_016881877.1	-15.4
BTBD2	BTB domain containing 2	protein_coding	NP_060267.2;XP_011526429.1	-15.4
SLX1A	SLX1 homolog A, structure-specific endonuclease	protein_coding	NP_001014999.1;NP_001015000.1	-15.4
LOC102723996	ICOS ligand	protein_coding	NP_001350699.1;XP_006723962.1;	-15.5
KLHL26	kelch like family member 26	protein_coding	NP_001332910.1;NP_001332911.1;	-15.5
HSPB1	heat shock protein family B (small) member 1	protein_coding	NP_001531.1	-15.5
SLC16A3	solute carrier family 16 member 3	protein_coding	NP_001035887.1;NP_001035888.1;	-15.5
ENDOG	endonuclease G	protein_coding	NP_004426.2;XP_011516649.1	-15.5
FKBP8	FKBP prolyl isomerase 8	protein_coding	NP_001295302.1;NP_036313.3	-15.5
ATP6V0C	ATPase H+ transporting V0 subunit c	protein_coding	NP_001185498.1;NP_001685.1	-15.5
EVI5L	ecotropic viral integration site 5 like	protein_coding	NP_001153416.1;NP_660288.1;XP_	-15.6
ZNF768	zinc finger protein 768	protein_coding	NP_078947.3;XP_016879154.1;XP_	-15.6
NTHL1	nth like DNA glycosylase 1	protein_coding	NP_001305122.2;NP_001305123.1;	-15.6
NACC1	nucleus accumbens associated 1	protein_coding	NP_443108.1;XP_005259778.1	-15.7

REEP6	receptor accessory protein 6	protein_coding	NP_001316485.1;NP_612402.1	-15.7
LMOD1	leiomodin 1	protein_coding	NP_036266.2	-15.8
EGFL7	EGF like domain multiple 7	protein_coding	NP_057299.1;NP_958854.1;XP_00	-15.8
MPP2	membrane palmitoylated protein 2	protein_coding	NP_001265299.1;NP_001265300.1;	-15.8
SLC30A3	solute carrier family 30 member 3	protein_coding	NP_001305878.1;NP_001305879.1;	-15.8
MIF	macrophage migration inhibitory factor	protein_coding	NP_002406.1	-15.8
PLEKHG4	pleckstrin homology and RhoGEF domain contain	protein_coding	NP_001123199.1;NP_001123200.1;	-15.9
SLC9A3R2	SLC9A3 regulator 2	protein_coding	NP_001123484.1;NP_001239002.1;	-15.9
H1-4	H1.4 linker histone, cluster member	protein_coding	NP_005312.1	-15.9
ISOC2	isochorismatase domain containing 2	protein_coding	NP_001129673.1;NP_001129674.1;	-16.0
MRPL41	mitochondrial ribosomal protein L41	protein_coding	NP_115866.1	-16.0
BAK1	BCL2 antagonist/killer 1	protein_coding	NP_001179.1;XP_011513081.1;XP_	-16.0
TSPAN9	tetraspanin 9	protein_coding	NP_001161792.1;NP_006666.1;XP_	-16.0
ARVCF	ARVCF delta catenin family member	protein_coding	NP_001661.1;XP_005261299.1;XP_	-16.1
TAX1BP3	Tax1 binding protein 3	protein_coding	NP_001191627.1;NP_055419.1	-16.1
PAK4	p21 (RAC1) activated kinase 4	protein_coding	NP_001014831.1;NP_001014832.1;	-16.1
ZBTB7B	zinc finger and BTB domain containing 7B	protein_coding	NP_001239335.1;NP_001243384.1;	-16.2
IGSF8	immunoglobulin superfamily member 8	protein_coding	NP_001193594.1;NP_001307176.1;	-16.2
TMUB1	transmembrane and ubiquitin like domain containi	protein_coding	NP_001129516.1;NP_113622.1	-16.2
LRWD1	leucine rich repeats and WD repeat domain contai	protein_coding	NP_001304650.1;NP_690852.1	-16.2
SLX1B	SLX1 homolog B, structure-specific endonuclease	protein_coding	NP_076949.1;NP_835145.1	-16.2
NUDT16L1	nudix hydrolase 16 like 1	protein_coding	NP_001180381.1;NP_001357514.1;	-16.2
HGH1	HGH1 homolog	protein_coding	NP_057542.2	-16.3
TMEM134	transmembrane protein 134	protein_coding	NP_001072118.1;NP_001072119.1;	-16.3
CACFD1	calcium channel flower domain containing 1	protein_coding	NP_001129247.1;NP_001229298.1;	-16.3
RIMBP3B	RIMS binding protein 3B	protein_coding	NP_001122107.1	-16.3
TCF3	transcription factor 3	protein_coding	NP_001129611.1;NP_001338707.1;	-16.3
RNH1	ribonuclease/angiogenin inhibitor 1	protein_coding	NP_002930.2;NP_976317.1;NP_97	-16.4
RPS2P5	ribosomal protein S2 pseudogene 5	pseudogene	.	-16.4
ATG4D	autophagy related 4D cysteine peptidase	protein_coding	NP_001268433.1;NP_116274.3;XP_	-16.4
TBL3	transducin beta like 3	protein_coding	NP_006444.2	-16.4
NOC4L	nucleolar complex associated 4 homolog	protein_coding	NP_076983.1;XP_016875449.1	-16.5
CDT1	chromatin licensing and DNA replication factor 1	protein_coding	NP_112190.2	-16.5
GLIS2	GLIS family zinc finger 2	protein_coding	NP_001305847.1;NP_115964.2	-16.5
MRPL12	mitochondrial ribosomal protein L12	protein_coding	NP_002940.2	-16.5
SRM	spermidine synthase	protein_coding	NP_003123.2;XP_016857668.1	-16.5
NACC2	NACC family member 2	protein_coding	NP_653254.1;XP_011516523.1	-16.5
GATA2	GATA binding protein 2	protein_coding	NP_001139133.1;NP_001139134.1;	-16.6
SAC3D1	SAC3 domain containing 1	protein_coding	NP_001354414.1;NP_001354415.1;	-16.6
DPF1	double PHD fingers 1	protein_coding	NP_001128627.1;NP_001128628.1;	-16.6
H2AC13	H2A clustered histone 13	protein_coding	NP_003500.1	-16.6
PPP1R16A	protein phosphatase 1 regulatory subunit 16A	protein_coding	NP_001316371.1;NP_001316372.1;	-16.7
POLR2E	RNA polymerase II subunit E	protein_coding	NP_001303252.1;NP_001303253.1;	-16.7
NECTIN1	nectin cell adhesion molecule 1	protein_coding	NP_002846.3;NP_976030.1;NP_97	-16.7
GPR137	G protein-coupled receptor 137	protein_coding	NP_001164351.1;NP_001164352.1;	-16.8
HCFC1	host cell factor C1	protein_coding	NP_005325.2;XP_006724878.1;XP_	-16.8
TUBB2A	tubulin beta 2A class IIa	protein_coding	NP_001060.1;NP_001297244.1	-16.8
SLC25A10	solute carrier family 25 member 10	protein_coding	NP_001257817.1;NP_001257882.1;	-16.9
FLNC	filamin C	protein_coding	NP_001120959.1;NP_001449.3	-16.9
ZNF385A	zinc finger protein 385A	protein_coding	NP_001124439.1;NP_001124440.1;	-17.0
MUC1	mucin 1, cell surface associated	protein_coding	NP_001018016.1;NP_001018017.1;	-17.0
MXD4	MAX dimerization protein 4	protein_coding	NP_006445.1	-17.0
SIX5	SIX homeobox 5	protein_coding	NP_787071.3	-17.0
HSPG2	heparan sulfate proteoglycan 2	protein_coding	NP_001278789.1;NP_005520.4;XP_	-17.0
AGPAT2	1-acylglycerol-3-phosphate O-acyltransferase 2	protein_coding	NP_001012745.1;NP_006403.2	-17.0
PIDD1	p53-induced death domain protein 1	protein_coding	NP_665893.2;NP_665894.2;XP_00	-17.0
H2AC14	H2A clustered histone 14	protein_coding	NP_066544.1	-17.0
MPND	MPN domain containing	protein_coding	NP_001153318.1;NP_001287791.1;	-17.0
MAP3K11	mitogen-activated protein kinase kinase kinase 11	protein_coding	NP_002410.1	-17.1
AKT1S1	AKT1 substrate 1	protein_coding	NP_001092102.1;NP_001092103.1;	-17.1
GNB2	G protein subunit beta 2	protein_coding	NP_005264.2	-17.1
H3C14	H3 clustered histone 14	protein_coding	NP_066403.2	-17.2
MVB12A	multivesicular body subunit 12A	protein_coding	NP_001291476.1;NP_612410.1	-17.2
BCAM	basal cell adhesion molecule (Lutheran blood grou	protein_coding	NP_001013275.1;NP_005572.2	-17.2
H3C15	H3 clustered histone 15	protein_coding	NP_001005464.1	-17.2
DOHH	deoxyhypusine hydroxylase	protein_coding	NP_001138637.1;NP_112594.1;XP_	-17.3
TIGD5	tigger transposable element derived 5	protein_coding	NP_116251.4	-17.3
TRIM47	tripartite motif containing 47	protein_coding	NP_258411.2;XP_005257844.1;XP_	-17.3
NUBP2	nucleotide binding protein 2	protein_coding	NP_001271430.1;NP_001271431.1;	-17.3
SBF1	SET binding factor 1	protein_coding	NP_001352748.1;NP_002963.2;XP_	-17.3
ERF	ETS2 repressor factor	protein_coding	NP_001287964.1;NP_001295331.1;	-17.4

NEURL1B	neuralized E3 ubiquitin protein ligase 1B	protein_coding	NP_001136123.1;NP_001295106.1;	-17.4
CDR2L	cerebellar degeneration related protein 2 like	protein_coding	NP_055418.2;XP_006721915.1	-17.4
UNC13D	unc-13 homolog D	protein_coding	NP_954712.1	-17.4
FBXL19	F-box and leucine rich repeat protein 19	protein_coding	NP_001093254.2;NP_001269280.1;	-17.4
AURKAIP1	aurora kinase A interacting protein 1	protein_coding	NP_001120701.1;NP_001120702.1;	-17.4
FAM174C	family with sequence similarity 174 member C	protein_coding	NP_060384.3	-17.5
PIP5K1C	phosphatidylinositol-4-phosphate 5-kinase type 1 ;	protein_coding	NP_001182662.1;NP_001287778.1;	-17.5
TTLL12	tubulin tyrosine ligase like 12	protein_coding	NP_055955.1	-17.5
HSPBP1	HSPA (Hsp70) binding protein 1	protein_coding	NP_001123578.1;NP_001284529.1;	-17.5
TELO2	telomere maintenance 2	protein_coding	NP_001338775.1;NP_057195.2;XP_	-17.5
OLFML2A	olfactomedin like 2A	protein_coding	NP_001269644.1;NP_872293.2;XP_	-17.5
BCAR1	BCAR1 scaffold protein, Cas family member	protein_coding	NP_001164185.1;NP_001164186.1;	-17.6
DVL1	dishevelled segment polarity protein 1	protein_coding	NP_001317240.1;NP_004412.2;XP_	-17.7
RNPEPL1	arginyl aminopeptidase like 1	protein_coding	NP_060696.4;XP_005247093.1	-17.8
IMPDH1	inosine monophosphate dehydrogenase 1	protein_coding	NP_000874.2;NP_001096075.1;NP_	-17.8
NPTXR	neuronal pentraxin receptor	protein_coding	NP_055108.2	-17.8
ARHGDIA	Rho GDP dissociation inhibitor alpha	protein_coding	NP_001172006.1;NP_001172007.1;	-17.8
CENPB	centromere protein B	protein_coding	NP_001801.1	-17.8
TSPAN17	tetraspanin 17	protein_coding	NP_001006617.2;NP_001353420.1;	-17.8
KREMEN1	kringle containing transmembrane protein 1	protein_coding	NP_001034659.2;NP_114434.3;XP_	-17.9
SEMA6B	semaphorin 6B	protein_coding	NP_115484.2;XP_011525941.1;XP_	-17.9
FZD2	frizzled class receptor 2	protein_coding	NP_001457.1	-17.9
SLC4A2	solute carrier family 4 member 2	protein_coding	NP_001186621.1;NP_001186622.1;	-17.9
PTRH1	peptidyl-tRNA hydrolase 1 homolog	protein_coding	NP_001002913.1;NP_001332906.1;	-17.9
SPATA2L	spermatogenesis associated 2 like	protein_coding	NP_689552.2;XP_005256336.1	-18.0
TMEM201	transmembrane protein 201	protein_coding	NP_001010866.1;NP_001124396.2;	-18.0
TEDC1	tubulin epsilon and delta complex 1	protein_coding	NP_001128347.1;NP_001128348.1;	-18.0
FDXR	ferredoxin reductase	protein_coding	NP_001244941.2;NP_001244942.2;	-18.1
CEP170B	centrosomal protein 170B	protein_coding	NP_001106197.1;NP_055820.2;XP_	-18.1
LIMS2	LIM zinc finger domain containing 2	protein_coding	NP_001129509.2;NP_001154875.1;	-18.1
PAQR4	progestin and adipoQ receptor family member 4	protein_coding	NP_001271440.1;NP_001271441.1;	-18.1
NAT14	N-acetyltransferase 14 (putative)	protein_coding	NP_065111.1	-18.2
MTSS2	MTSS I-BAR domain containing 2	protein_coding	NP_612392.1;XP_005256293.3;XP_	-18.2
NFIX	nuclear factor I X	protein_coding	NP_001257972.1;NP_001257973.1;	-18.2
CERS1	ceramide synthase 1	protein_coding	NP_001277194.1;NP_067090.1;NP_	-18.2
RABAC1	Rab acceptor 1	protein_coding	NP_006414.2	-18.2
ABCD1	ATP binding cassette subfamily D member 1	protein_coding	NP_000024.2	-18.2
ATF5	activating transcription factor 5	protein_coding	NP_001180575.1;NP_001277675.1;	-18.2
KHSRP	KH-type splicing regulatory protein	protein_coding	NP_001353228.1;NP_001353229.1;	-18.2
TUBB3	tubulin beta 3 class III	protein_coding	NP_001184110.1;NP_006077.2	-18.3
LMNA	lamin A/C	protein_coding	NP_001244303.1;NP_001269553.1;	-18.4
PITX1	paired like homeodomain 1	protein_coding	NP_002644.4	-18.5
CNN2	calponin 2	protein_coding	NP_001290428.1;NP_001290430.1;	-18.6
GPSM1	G protein signaling modulator 1	protein_coding	NP_001139110.2;NP_001139111.1;	-18.6
SLC52A2	solute carrier family 52 member 2	protein_coding	NP_001240744.1;NP_001240745.1;	-18.6
MMP11	matrix metallopeptidase 11	protein_coding	NP_005931.2	-18.7
MAP2K2	mitogen-activated protein kinase kinase 2	protein_coding	NP_109587.1;XP_006722862.1;XP_	-18.7
HS6ST1P1	heparan sulfate 6-O-sulfotransferase 1 pseudogene	pseudogene	.	-18.8
MOSPD3	motile sperm domain containing 3	protein_coding	NP_001035186.1;NP_001035187.1;	-18.8
NCKAP5L	NCK associated protein 5 like	protein_coding	NP_001032895.2;NP_001354977.1;	-18.8
CIC	capicua transcriptional repressor	protein_coding	NP_001291744.1;NP_001366409.1;	-18.9
THEM6	thioesterase superfamily member 6	protein_coding	NP_001349929.1;NP_057731.1	-18.9
PTMS	parathymosin	protein_coding	NP_001317262.1;NP_002815.3	-18.9
GNA11	G protein subunit alpha 11	protein_coding	NP_002058.2	-18.9
GAS2L1	growth arrest specific 2 like 1	protein_coding	NP_001265659.1;NP_001349914.1;	-18.9
NOTCH1	notch receptor 1	protein_coding	NP_060087.3;XP_011517019.2	-19.0
FBRSL1	fibrosin like 1	protein_coding	NP_001136113.1;NP_001354800.1;	-19.1
RFNG	RFNG O-fucosylpeptide 3-beta-N-acetylglucosam	protein_coding	NP_002908.1;XP_011521889.1	-19.1
SLC6A8	solute carrier family 6 member 8	protein_coding	NP_001136277.1;NP_001136278.1;	-19.1
RBM42	RNA binding motif protein 42	protein_coding	NP_001306042.1;NP_077297.2	-19.2
RPUSD1	RNA pseudouridine synthase domain containing 1	protein_coding	NP_001311015.1;NP_001311339.1;	-19.2
CORO7	coronin 7	protein_coding	NP_001188401.1;NP_001188402.1;	-19.3
PIGQ	phosphatidylinositol glycan anchor biosynthesis cl	protein_coding	NP_004195.2;NP_683721.1	-19.4
EVA1B	eva-1 homolog B	protein_coding	NP_001291691.1;NP_060636.1	-19.4
GCHFR	GTP cyclohydrolase I feedback regulator	protein_coding	NP_005249.1	-19.4
RBM38	RNA binding motif protein 38	protein_coding	NP_001278709.1;NP_059965.2;NP_	-19.5
MFSD3	major facilitator superfamily domain containing 3	protein_coding	NP_612440.1;XP_011515108.1;XP_	-19.5
TNRC18	trinucleotide repeat containing 18	protein_coding	NP_001073964.2;XP_016868217.1;	-19.5
CBX6	chromobox 6	protein_coding	NP_001290423.1;NP_055107.3	-19.5
RNA18SN1	RNA, 18S ribosomal N1	rRNA	.	-19.5
LZTS2	leucine zipper tumor suppressor 2	protein_coding	NP_001305028.1;NP_001305029.1;	-19.6

ATP5F1D	ATP synthase F1 subunit delta	protein_coding	NP_001001975.1;NP_001678.1	-19.6
PHLDA3	pleckstrin homology like domain family A membe	protein_coding	NP_036528.1	-19.7
WDR18	WD repeat domain 18	protein_coding	NP_001359014.1;NP_001359015.1;	-19.9
PKMYT1	protein kinase, membrane associated tyrosine/thre	protein_coding	NP_001245379.1;NP_001245380.1;	-20.0
DGCR6L	DiGeorge syndrome critical region gene 6 like	protein_coding	NP_150282.2	-20.1
MEX3D	mex-3 RNA binding family member D	protein_coding	NP_001167589.1;NP_976049.3;XP_	-20.1
SORBS3	sorbin and SH3 domain containing 3	protein_coding	NP_001018003.1;NP_005766.3;XP_	-20.2
L1CAM	L1 cell adhesion molecule	protein_coding	NP_000416.1;NP_001137435.1;NP_	-20.2
RAVER1	ribonucleoprotein, PTB binding 1	protein_coding	NP_001353103.1;NP_597709.3	-20.2
BAD	BCL2 associated agonist of cell death	protein_coding	NP_004313.1;NP_116784.1	-20.2
RIMBP3C	RIMS binding protein 3C	protein_coding	NP_001122105.1	-20.2
GAA	glucosidase alpha, acid	protein_coding	NP_000143.2;NP_001073271.1;NP_	-20.2
RPS19BP1	ribosomal protein S19 binding protein 1	protein_coding	NP_919307.1	-20.2
ZYX	zyxin	protein_coding	NP_001010972.1;NP_001349712.1;	-20.3
CARD10	caspase recruitment domain family member 10	protein_coding	NP_055365.2	-20.4
MIDN	midnolin	protein_coding	NP_796375.3;XP_005259728.1;XP_	-20.4
LZTS1	leucine zipper tumor suppressor 1	protein_coding	NP_001349813.1;NP_066300.1;XP_	-20.5
TNXA	tenascin XA (pseudogene)	ranscribed_pseudogen	.	-20.5
SAMD1	sterile alpha motif domain containing 1	protein_coding	NP_612361.1	-20.6
PARD6A	par-6 family cell polarity regulator alpha	protein_coding	NP_001032358.1;NP_058644.1;XP_	-20.6
SSBP4	single stranded DNA binding protein 4	protein_coding	NP_001009998.1;NP_116016.1;XP_	-20.7
PNPLA2	patatin like phospholipase domain containing 2	protein_coding	NP_065109.1;XP_006718328.1;XP_	-20.8
ADAM11	ADAM metallopeptidase domain 11	protein_coding	NP_001305862.1;NP_002381.2;XP_	-20.8
PPL	periplakin	protein_coding	NP_002696.4;XP_006720965.1;XP_	-20.8
RHOF	ras homolog family member F, filopodia associate	protein_coding	NP_061907.2	-20.8
FBRS	fibrosin	protein_coding	NP_001098549.2;XP_011544218.1;	-20.9
ETNK2	ethanolamine kinase 2	protein_coding	NP_001284689.1;NP_001284690.1;	-20.9
SAPCD2	suppressor APC domain containing 2	protein_coding	NP_848543.2;XP_011517482.1	-20.9
CTU2	cytosolic thiouridylase subunit 2	protein_coding	NP_001012777.1;NP_001012780.1;	-20.9
CSPG4	chondroitin sulfate proteoglycan 4	protein_coding	NP_001888.2	-21.0
DUSP8	dual specificity phosphatase 8	protein_coding	NP_004411.2;XP_011518234.1;XP_	-21.0
RGS19	regulator of G protein signaling 19	protein_coding	NP_001034556.1;NP_005864.1;XP_	-21.1
SPNS1	sphingolipid transporter 1 (putative)	protein_coding	NP_001135920.1;NP_001135921.1;	-21.1
GDF6	growth differentiation factor 6	protein_coding	NP_001001557.1	-21.1
TOR4A	torsin family 4 member A	protein_coding	NP_060193.2	-21.2
ZDHHC8	zinc finger DHHC-type palmitoyltransferase 8	protein_coding	NP_001171953.1;NP_037505.1;XP_	-21.2
NCLN	nicalin	protein_coding	NP_001308392.1;NP_064555.2	-21.2
P3H4	prolyl 3-hydroxylase family member 4 (inactive)	protein_coding	NP_006446.1;XP_006721703.1;XP_	-21.3
TEDC2	tubulin epsilon and delta complex 2	protein_coding	NP_079384.2;XP_011520969.1	-21.6
BRAT1	BRCA1 associated ATM activator 1	protein_coding	NP_001337555.1;NP_001337556.1;	-21.6
H1-5	H1.5 linker histone, cluster member	protein_coding	NP_005313.1	-21.7
SLC12A9	solute carrier family 12 member 9	protein_coding	NP_001254741.1;NP_001254743.1;	-21.8
EHD2	EH domain containing 2	protein_coding	NP_055416.2	-22.0
PLEC	plectin	protein_coding	NP_000436.2;NP_958780.1;NP_95	-22.0
ZNF703	zinc finger protein 703	protein_coding	NP_079345.1	-22.0
DYNC2I2	dynein 2 intermediate chain 2	protein_coding	NP_443076.2;XP_011517481.1	-22.1
KIAA1522	KIAA1522	protein_coding	NP_001185901.1;NP_001185902.1;	-22.1
ICOSLG	inducible T cell costimulator ligand	protein_coding	NP_001269979.1;NP_001269980.1;	-22.1
ATP6V0E2	ATPase H+ transporting V0 subunit e2	protein_coding	NP_001094062.1;NP_001276919.1;	-22.2
TPRN	taperin	protein_coding	NP_001121700.2	-22.2
ALDH16A1	aldehyde dehydrogenase 16 family member A1	protein_coding	NP_001138868.1;NP_699160.2;XP_	-22.3
METTL7A	methyltransferase like 7A	protein_coding	NP_054752.3	-22.3
CASKIN2	CASK interacting protein 2	protein_coding	NP_001136115.1;NP_065804.2	-22.3
H3C13	H3 clustered histone 13	protein_coding	NP_001116847.1	-22.5
EPHB4	EPH receptor B4	protein_coding	NP_004435.3;XP_016867305.1	-22.5
MIRLET7BHG	MIRLET7B host gene	lncRNA	.	-22.6
MBD3	methyl-CpG binding domain protein 3	protein_coding	NP_001268382.1;NP_001268383.1	-22.7
GIPC1	GIPC PDZ domain containing family member 1	protein_coding	NP_005707.1;NP_974197.1;NP_97	-22.7
EHD1	EH domain containing 1	protein_coding	NP_001269373.1;NP_001269374.1;	-23.0
UBTD1	ubiquitin domain containing 1	protein_coding	NP_079230.1	-23.0
GPAA1	glycosylphosphatidylinositol anchor attachment 1	protein_coding	NP_003792.1	-23.1
A4GALT	alpha 1,4-galactosyltransferase (P blood group)	protein_coding	NP_001304967.1;NP_059132.1;XP_	-23.2
C9orf16	chromosome 9 open reading frame 16	protein_coding	NP_077017.1;XP_011517306.1	-23.3
TMCC2	transmembrane and coiled-coil domain family 2	protein_coding	NP_001229854.1;NP_001284540.1;	-23.4
METRN	meteorin, glial cell differentiation regulator	protein_coding	NP_076947.1	-23.4
MAP1S	microtubule associated protein 1S	protein_coding	NP_001295292.1;NP_060644.4;XP_	-23.4
BORCS6	BLOC-1 related complex subunit 6	protein_coding	NP_060092.2	-23.6
FSCN1	fascin actin-bundling protein 1	protein_coding	NP_003079.1	-23.6
CREB3L1	cAMP responsive element binding protein 3 like 1	protein_coding	NP_443086.1;XP_006718443.1	-23.7
CAVIN1	caveolae associated protein 1	protein_coding	NP_036364.2;XP_005257299.1	-23.8
CDC42EP1	CDC42 effector protein 1	protein_coding	NP_689449.1	-23.9

PKP3	plakophilin 3	protein_coding	NP_001289958.1;NP_009114.1	-24.3
CPTP	ceramide-1-phosphate transfer protein	protein_coding	NP_001025056.1;XP_005244858.1;	-24.5
ZBTB42	zinc finger and BTB domain containing 42	protein_coding	NP_001131073.1;NP_001357271.1	-24.5
GAMT	guanidinoacetate N-methyltransferase	protein_coding	NP_000147.1;NP_620279.1	-24.6
KLF16	Kruppel like factor 16	protein_coding	NP_114124.1	-24.7
GALK1	galactokinase 1	protein_coding	NP_000145.1;NP_001368914.1	-24.7
ID1	inhibitor of DNA binding 1, HLH protein	protein_coding	NP_002156.2;NP_851998.1	-24.8
TRIM29	tripartite motif containing 29	protein_coding	NP_001317311.1;NP_036233.2;XP_	-24.8
IER5L	immediate early response 5 like	protein_coding	NP_982258.2	-24.8
BAIAP2-DT	BAIAP2 divergent transcript	lncRNA	.	-24.9
LMF2	lipase maturation factor 2	protein_coding	NP_001350745.1;NP_149977.2;XP_	-25.3
PLPPR2	phospholipid phosphatase related 2	protein_coding	NP_001164106.1;NP_073574.2;XP_	-25.7
QPRT	quinolinate phosphoribosyltransferase	protein_coding	NP_001305178.1;NP_001305179.2;	-25.9
PRXL2B	peroxiredoxin like 2B	protein_coding	NP_001182665.4;NP_001182666.4;	-25.9
TP53I13	tumor protein p53 inducible protein 13	protein_coding	NP_001333006.1;NP_001333007.1;	-26.0
ZNF358	zinc finger protein 358	protein_coding	NP_060553.4;XP_005272517.1;XP_	-26.0
RPPH1	ribonuclease P RNA component H1	RNase_P_RNA	.	-26.1
APBB1	amyloid beta precursor protein binding family B m	protein_coding	NP_001155.1;NP_001244248.1;NP_	-26.1
RXRA	retinoid X receptor alpha	protein_coding	NP_001278849.1;NP_001278850.1;	-26.3
CHMP6	charged multivesicular body protein 6	protein_coding	NP_078867.2;XP_005257725.1	-26.4
SLC2A4RG	SLC2A4 regulator	protein_coding	NP_064446.2	-26.6
SCAF1	SR-related CTD associated factor 1	protein_coding	NP_067051.2;XP_005259179.1;XP_	-26.6
CASTOR2	cytosolic arginine sensor for mTORC1 subunit 2	protein_coding	NP_001138536.1;XP_016868063.1	-26.8
CDH3	cadherin 3	protein_coding	NP_001304124.1;NP_001304125.1;	-26.9
HS6ST1	heparan sulfate 6-O-sulfotransferase 1	protein_coding	NP_004798.3	-27.1
ZDHHC12	zinc finger DHHC-type palmitoyltransferase 12	protein_coding	NP_001304944.2;NP_001304945.1;	-27.1
SNTA1	syntrophin alpha 1	protein_coding	NP_003089.1;XP_005260574.1;XP_	-27.3
PPDPF	pancreatic progenitor cell differentiation and proli	protein_coding	NP_001340352.1;NP_077275.1	-27.3
SCRIB	scribble planar cell polarity protein	protein_coding	NP_056171.3;NP_874365.3	-27.6
CD320	CD320 molecule	protein_coding	NP_001159367.1;NP_057663.1	-27.7
H1-10	H1.10 linker histone	protein_coding	NP_006017.1	-27.8
TTYH3	tweety family member 3	protein_coding	NP_079526.1;XP_011513837.1;XP_	-27.9
RPS6KA4	ribosomal protein S6 kinase A4	protein_coding	NP_001006945.1;NP_001287731.1;	-27.9
MCRIP1	MAPK regulated corepressor interacting protein 1	protein_coding	NP_001087236.1;NP_001275727.1;	-29.4
PLCD3	phospholipase C delta 3	protein_coding	NP_588614.1;XP_011522555.1;XP_	-29.5
PTCD1	pentatricopeptide repeat domain 1	protein_coding	NP_056360.2	-29.5
RNA28SN3	RNA, 28S ribosomal N3	rRNA	.	-29.5
TSC22D4	TSC22 domain family member 4	protein_coding	NP_001289972.1;NP_112197.1	-29.7
ID3	inhibitor of DNA binding 3, HLH protein	protein_coding	NP_002158.3	-29.9
C20orf27	chromosome 20 open reading frame 27	protein_coding	NP_001034229.1;NP_001245358.1;	-29.9
LOC728392	uncharacterized LOC728392	protein_coding	NP_001155843.1	-29.9
SCAMP4	secretory carrier membrane protein 4	protein_coding	NP_001316468.1;NP_001316469.1;	-30.0
AHDC1	AT-hook DNA binding motif containing 1	protein_coding	NP_001025053.1;NP_001358857.1;	-30.5
FUZ	fuzzy planar cell polarity protein	protein_coding	NP_001165408.1;NP_001339191.1;	-30.7
LOC107984334	uncharacterized LOC107984334, transcript varian	lncRNA	.	-30.7
PKD1P4	polycystin 1, transient receptor potential channel i	pseudogene	.	-31.0
SCAND1	SCAN domain containing 1	protein_coding	NP_057642.1;NP_361012.2	-31.2
ITGB4	integrin subunit beta 4	protein_coding	NP_000204.3;NP_001005619.1;NP_	-31.5
IRF2BP1	interferon regulatory factor 2 binding protein 1	protein_coding	NP_056464.1	-32.0
PRR12	proline rich 12	protein_coding	NP_065770.1	-32.1
RASSF7	Ras association domain family member 7	protein_coding	NP_001137465.1;NP_001137466.1;	-32.1
ADGRA2	adhesion G protein-coupled receptor A2	protein_coding	NP_116166.9;XP_011542783.1;XP_	-32.5
LRFN4	leucine rich repeat and fibronectin type III domain	protein_coding	NP_001350453.1;NP_076941.2	-33.4
ZNF219	zinc finger protein 219	protein_coding	NP_001095142.1;NP_001095924.1;	-33.8
EEF1A2	eukaryotic translation elongation factor 1 alpha 2	protein_coding	NP_001949.1	-33.9
TSPO	translocator protein	protein_coding	NP_000705.2;NP_001243459.1;NP_	-35.0
ADGRB2	adhesion G protein-coupled receptor B2	protein_coding	NP_001281264.1;NP_001281265.1;	-35.3
GPC1	glypican 1	protein_coding	NP_002072.2;XP_011509278.1	-36.1
CCDC85B	coiled-coil domain containing 85B	protein_coding	NP_006839.2	-37.3
RNA45SN2	RNA, 45S pre-ribosomal N2	rRNA	.	-37.8
COL1A1	collagen type I alpha 1 chain	protein_coding	NP_000079.2;XP_005257115.2;XP_	-40.9
RNA45SN1	RNA, 45S pre-ribosomal N1	rRNA	.	-41.7
SCARF2	scavenger receptor class F member 2	protein_coding	NP_699165.3;NP_878315.2;XP_01(	-44.5
KRT17	keratin 17	protein_coding	NP_000413.1	-44.6
APOE	apolipoprotein E	protein_coding	NP_000032.1;NP_001289617.1;NP_	-47.5
VASN	vasorin	protein_coding	NP_612449.2	-47.9
TNS1	tensin 1	protein_coding	NP_001294951.1;NP_001294952.1;	-51.5
RNA45SN3	RNA, 45S pre-ribosomal N3	rRNA	.	-53.1
TENT5B	terminal nucleotidyltransferase 5B	protein_coding	NP_443175.2	-57.5
SCARNA2	small Cajal body-specific RNA 2	guide_RNA	.	-58.5
RNA28SN1	RNA, 28S ribosomal N1	rRNA	.	-62.9

MRGPRF	MAS related GPR family member F	protein_coding	NP_001091985.1;NP_659452.3;XP_	-64.5
RNA28SN2	RNA, 28S ribosomal N2	rRNA	.	-64.8
NXN	nucleoredoxin	protein_coding	NP_001192248.1;NP_071908.2;XP_	-77.2
MIR3648-2	microRNA 3648-2	miRNA	.	-112.2
